# Identification of transcriptome and fluralaner responsive genes in the common cutworm *Spodoptera litura* Fabricius, based on RNA-seq

**DOI:** 10.1186/s12864-020-6533-0

**Published:** 2020-02-03

**Authors:** Zhong-Qiang Jia, Di Liu, Ying-Chuan Peng, Zhao-Jun Han, Chun-Qing Zhao, Tao Tang

**Affiliations:** 1 Key Laboratory of Integrated Pest Management in Crops in Eastern China (Ministry of Agriculture of China), College of Plant Protection, Nanjing Agricultural University, Nanjing, 210095 People’s Republic of China; 2 Institute of Plant Protection, Hunan Academy of Agricultural Sciences, Changsha, 410125 People’s Republic of China; 3 Present address: Institute of Entomology, Jiangxi Agricultural University, Nanchang, 330045 China

**Keywords:** *Spodoptera litura*, Fluralaner, Transcriptome, GABA receptor, Cytochrome P450 enzyme, Resistance, Agricultural pests

## Abstract

**Background:**

Fluralaner is a novel isoxazoline insecticide with a unique action site on the γ-aminobutyric acid receptor (GABAR), shows excellent activity on agricultural pests including the common cutworm *Spodoptera litura*, and significantly influences the development and fecundity of *S. litura* at either lethal or sublethal doses. Herein, Illumina HiSeq Xten (IHX) platform was used to explore the transcriptome of *S. litura* and to identify genes responding to fluralaner exposure.

**Results:**

A total of 16,572 genes, including 451 newly identified genes, were observed in the *S. litura* transcriptome and annotated according to the COG, GO, KEGG and NR databases. These genes included 156 detoxification enzyme genes [107 cytochrome P450 enzymes (P450s), 30 glutathione *S*-transferases (GSTs) and 19 carboxylesterases (CarEs)] and 24 insecticide-targeted genes [5 ionotropic GABARs, 1 glutamate-gated chloride channel (GluCl), 2 voltage-gated sodium channels (VGSCs), 13 nicotinic acetylcholine receptors (nAChRs), 2 acetylcholinesterases (AChEs) and 1 ryanodine receptor (RyR)]. There were 3275 and 2491 differentially expressed genes (DEGs) in *S. litura* treated with LC_30_ or LC_50_ concentrations of fluralaner, respectively. Among the DEGs, 20 related to detoxification [16 P450s, 1 GST and 3 CarEs] and 5 were growth-related genes (1 chitin and 4 juvenile hormone synthesis genes). For 26 randomly selected DEGs, real-time quantitative PCR (RT-qPCR) results showed that the relative expression levels of genes encoding several P450s, GSTs, heat shock protein (HSP) 68, vacuolar protein sorting-associated protein 13 (VPSAP13), sodium-coupled monocarboxylate transporter 1 (SCMT1), pupal cuticle protein (PCP), protein takeout (PT) and low density lipoprotein receptor adapter protein 1-B (LDLRAP1-B) were significantly up-regulated. Conversely, genes encoding esterase, sulfotransferase 1C4, proton-coupled folate transporter, chitinase 10, gelsolin-related protein of 125 kDa (GRP), fibroin heavy chain (FHC), fatty acid synthase and some P450s were significantly down-regulated in response to fluralaner.

**Conclusions:**

The transcriptome in this study provides more effective resources for the further study of *S. litura* whilst the DEGs identified sheds further light on the molecular response to fluralaner.

## Background

The common cutworm, *Spodoptera litura* Fabricius (Lepidoptera: Noctuidae), is a destructive polyphagous pest with a broad host plant range of more than 100 species of crops and vegetables [1]. It is widely distributed around the world and has evolved high resistance to most conventional insecticides, including carbamates, pyrethroids and organophosphates [2, 3]. As a result, farmers are using more insecticides leading to serious environmental pollution [4]. Therefore, exploring alternative and environmentally-friendly insecticides is one important approach to reduce the economic loss from *S. litura*.

Fluralaner, as a novel isoxazoline insecticide, shows excellent insecticidal activity to agricultural pests including *S. litura*, the rice stem borer *Chilo suppressalis* Walker, the fall armyworm *S. frugiperda* Smith & Abbot, the corn earworm *Helicoverpa zea* Boddie, the potato leafhopper *Empoasca fabae* (Harris), the western flower thrips *Frankliniella occidentalis* (Pergande) and the two-spotted spider mite *Tetranychus urticae* Koch [5, 6]. It exhibits no-cross resistance to the conventional GABAR targeting insecticides, such as phenylpyrazoles (e.g., fipronil) and cyclodienes (e.g., α-endosulfan) [7]. Fluralaner was found to significantly reduce the weight of ticks on fluralaner-treated dogs [8]. Similarly, fluralaner postpones the development and reduces the fecundity of *S. litura* and significantly affects several detoxification-related genes [9]. However, the effect of fluralaner on *S. litura* at the transcriptomic level remains unclear.

The transcriptome has been widely used as a powerful tool for exploring the molecular mechanisms of genes that respond to insecticides and toxins. For instance, Cui et al. (2017) explored the responsive genes of the Asian corn borer (*Ostrinia furnacalis* Guenée) to flubendiamide [10]. Song et al. (2017) found that immunity-, metabolism- and Bt-related genes in *S. litura* midgut were responsive to Vip3Aa toxin, highlighting that trypsin was possibly involved in Vip3Aa activation [11]. Xu et al. (2014) found that in the carmine spider mite, *T. cinnabarinus* (Boisduval), 10 gene categories were putatively involved in insecticide resistance [12]. Marco et al. (2017) found that the defensome family genes of the mosquito, *Anopheles stephensi* Liston, responded to toxicants during insecticide exposure [13].

In the present study, the transcriptomes of control and fluralaner-treated *S. litura* were sequenced with the Illumina HiSeq Xten (IHX) platform (Illumina, Inc., San Diego, CA) and assembled according to the reference genome [14]. Hundreds of new genes were annotated by the NR, GO, COG and KEGG databases. The differentially expressed genes (DEGs) between control and fluralaner-treated *S. litura* were identified. In particular, detoxification enzyme genes and insecticide-targeted receptor genes were investigated, and fluralaner responsive genes relating to detoxification and development were also analyzed.

## Results

### RNA-seq, sequence assembly, transcript analysis and functional classification

The *S. litura* transcriptomic data were generated from 9 different libraries on the IHX platform. After removing the reads with adaptor and low quality, 70.97 Gb clean reads were produced with Q_30_ > 94.15%. After alignment, clean reads (85.71–88.22%) were successfully mapped to the *S. litura* genome, of which 85.83% mapped to exons and the others mapped to intergenic regions or introns. Finally, 16,926 genes were assembled in the *S. litura* transcriptome. Subsequently, 16,572 genes, including 451 new genes, were successfully annotated according to COG (6142), GO (7887), KEGG (6812), and NR (16,554) databases. In the transcriptome, 12,707 annotated genes were > 1000 bp long whilst 3850 annotated genes were between 300 and 999 bp.

**Fig. 1:**
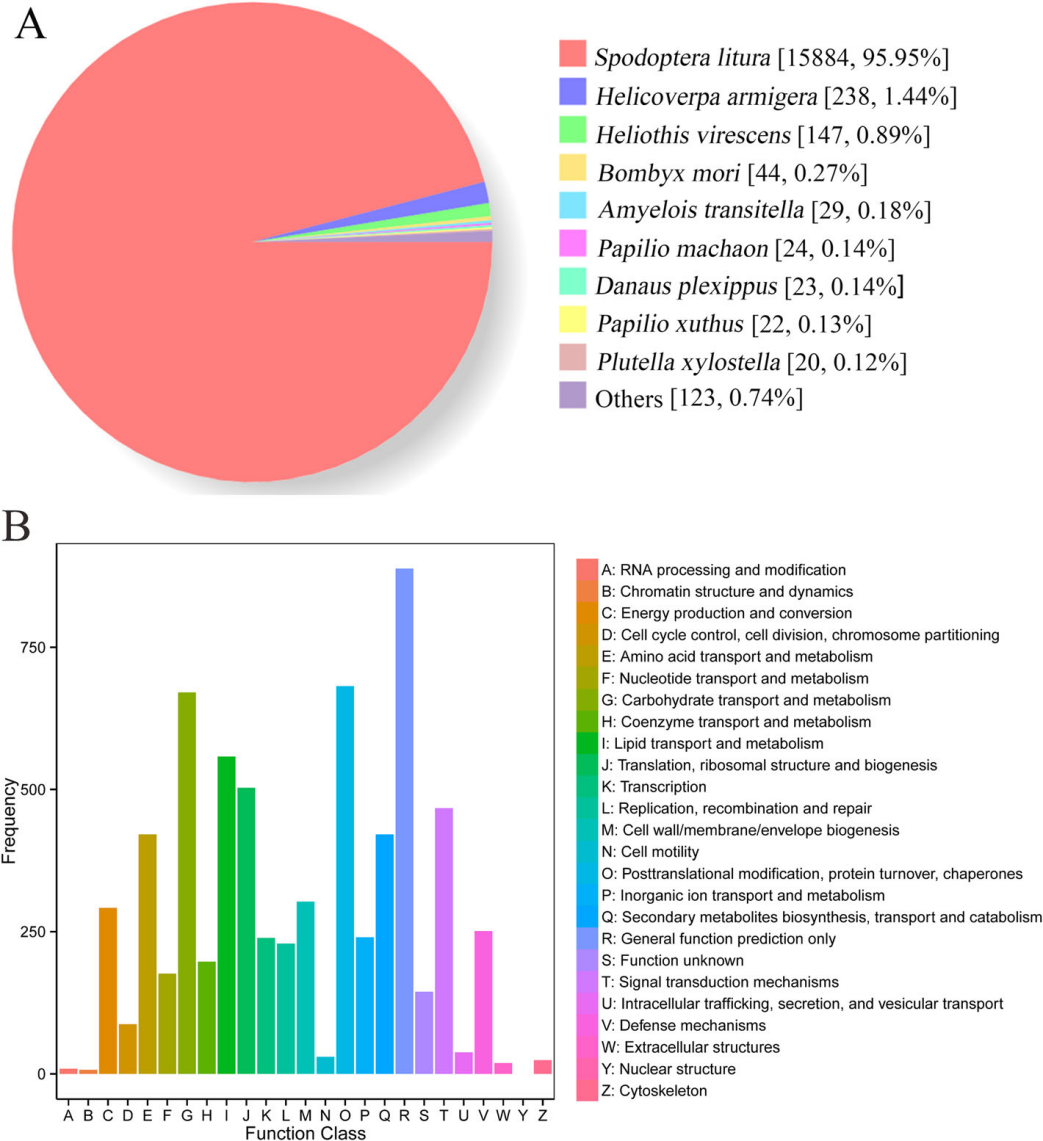
Functional classification of annotated genes according to different database. Note: **a** species distribution of homology search of genes annotated by NR database; **b** distribution of COG functional classification of all annotated genes

**Fig. 2:**
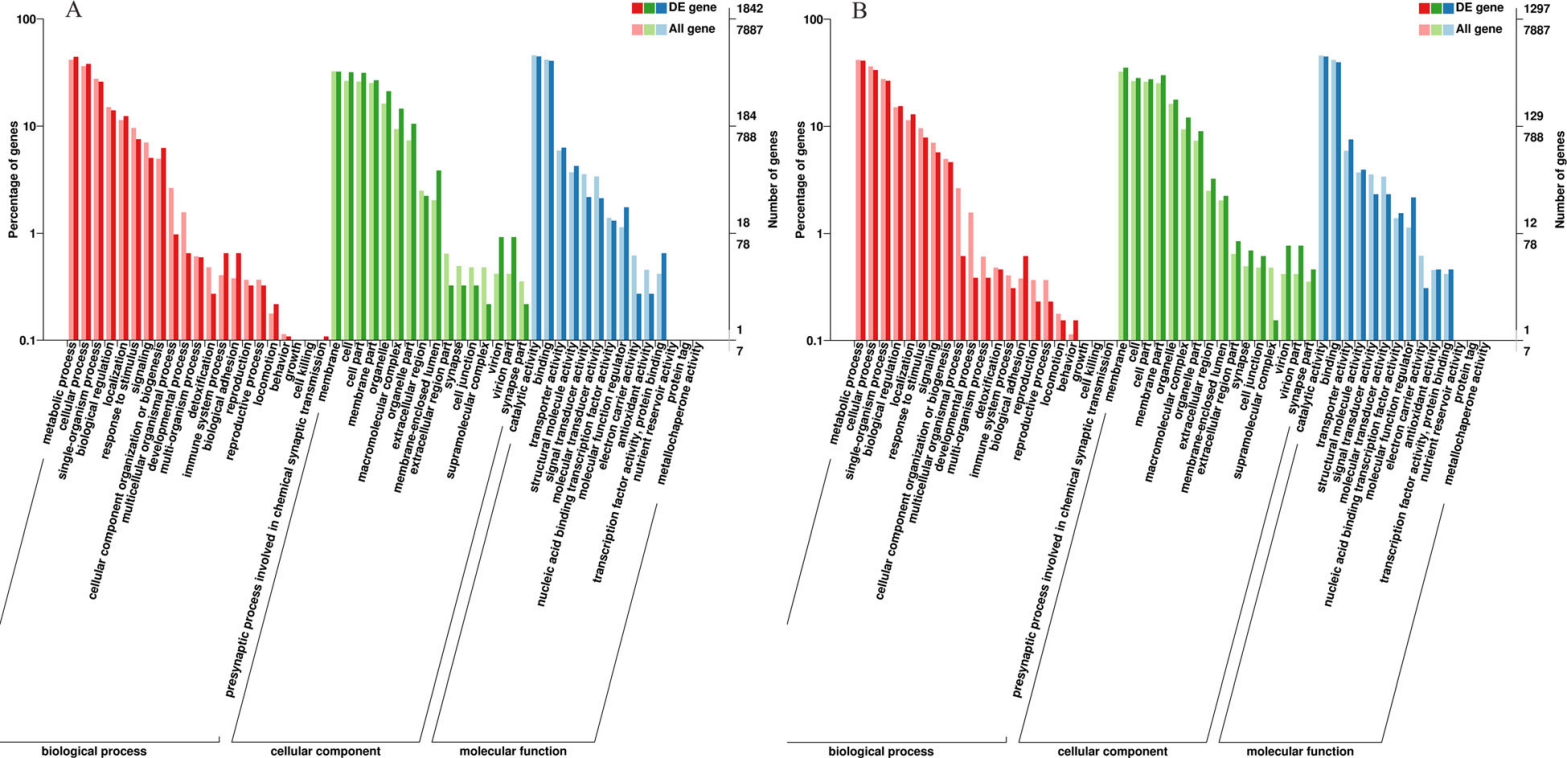
Functional classification of DEGs according to GO database. Note: **a** represented the DEGs and all genes after the exposure of LC_30_ fluralaner, **b** represented the DEGs and all genes after the exposure of LC_50_ fluralaner, respectively

Through comparing the *S. litura* transcripts against NR databases, most annotated genes (≥ 99.26%) were highly homologous to Lepidopteran genes. To be specific, 15,884 (95.95%) genes annotated by NR databases were homologous with those of *S. litura*, followed by 238 (1.44%) and 147 (0.89%) genes that were homologous to those of *H. armigera* Hübner and *Heliothis virescens* (Fabricius), respectively. Moreover, homologous genes identified by significant Blast hits from nine insect species are shown in Fig. 1a. According to the COG database, 6142 genes were annotated and classified into 25 specific categories and each gene was classified into at least one category (Fig. 1b and Additional file 1). According to the GO database, 7887 annotated genes were primarily divided into three categories of “cellular component”, “molecular function” and “biological process”, and were further classified into 51 functional sub-categories, including 21, 16 and 14 sub-categories in the “biological process”, “cellular component” and “molecular function” categories, respectively (Fig. 2 and Additional file 1). According to the KEGG database, 6812 annotated genes were involved in 154 pathways with “purine metabolism” (174 genes, 2.55%), “carbon metabolism” (154 genes, 2.26%) and “peroxisome” (143 genes, 2.10%) being the largest three pathways (Additional file 2).

### Detoxification enzymes and insecticide-targeted genes

Insecticide metabolism and resistance are mainly related to detoxification enzymes and insecticide-targeted genes of insects. In this study, the main detoxification enzyme genes (P450s, GSTs and CarEs) and insecticide-targeted genes (GABARs, GluCls, VGSCs, nAChRs, AChEs and RyRs) identified by BLAST searches against the NR database were analyzed. Compared with those from *B. mori*, *Drosophila melanogaster* Meigen, *Apis mellifera* Linnaeus, *H. armigera*, *Plutella xylostella* Linnaeus, 114 P450s genes were identified, 107 of which (Additional file 3) were clustered into CYP2, CYP3, CYP4 and mitochondrial (CYP M) clades consisting of 44, 46, 10 and 7 genes, respectively (Additional file 4); 30 GSTs genes encoding proteins of 125–246 amino acid residues (Additional file 5) were identified and distributed into 6 clades: ε (16), σ (5), δ (4), microsomal (2), θ (1) and unclassified (2) clades (Additional file 6); 19 CarEs genes (Additional file 7) were identified (Additional file 8) as well. The identified insecticide-targeted genes included 6 putative ionotropic GABARs, 2 GluCls, 2 VGSCs, 15 nAChR subunits, 6 AChEs and 1 RyR that were annotated by the NR database (Additional file 9). However, phylogenetic analysis identified 5 ionotropic GABARs, 1 GluCl, 12 nAChR subunits and 2 AChE genes (Additional file 10 and Additional file 11).

### Expression profile analysis of fluralaner responsive genes

**Fig. 3:**
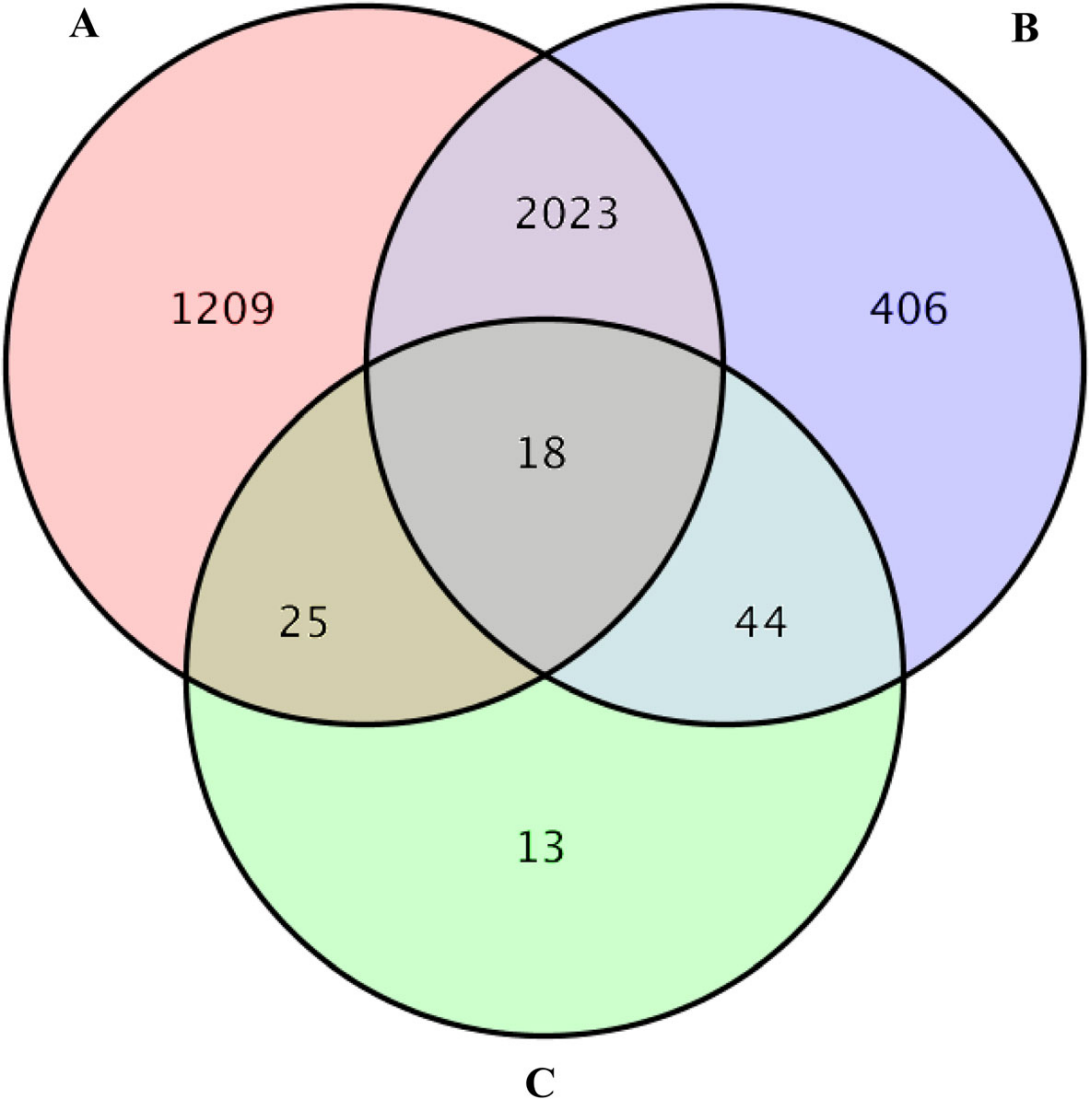
Venn diagram of the DEGs among different treated groups. Note: **a** represented the DEGs between the control and LC_30_ group; **b** represented the DEGs between the control and LC_50_ group; **c** represented the DEGs between the LC_30_ and LC_50_ group

In general, the transcriptome can represent the response of gene expression upon exposure to chemicals [10, 15]. To determine the influence of fluralaner on the expression profile of genes, the change of genes was analyzed using the Fragments Per Kilobase of transcript per Million fragments mapped (FPKM) method [16]. A total of 3725 DEGs were found in LC_30_ and LC_50_ fluralaner-treated groups. The Venn diagram shows the number of shared and exclusive DEGs at each group, and a total of 3275 (1695 up-regulated and 1580 down-regulated) or 2491 (1500 up-regulated and 991 down-regulated) DEGs were found after exposure to LC_30_ and LC_50_ fluralaner compared with the control group, respectively. 100 DEGs were shared between the LC_30_ and LC_50_ treated groups (Fig. 3). 1209 and 406 DEGs were exclusive to LC_30_ and LC_50_ exposure to fluralaner, respectively, whilst 2041 genes DEGs were shared between all treated groups (Fig. 3).

Subsequently, the DEGs in LC_30_ and LC_50_ fluralaner-treated groups were analyzed by GO, COG and KEGG databases. 1842 and 1297 DEGs annotated by GO databases were divided into 50 and 48 sub-categories, respectively. The major sub-categories were “membrane” with 592 (32.14%) and 455 (35.08%) DEGs, respectively, “catalytic activity” with 823 (44.68%) and 580 (44.72%) DEGs, and “metabolic process” with 814 (44.19%) and 531 (40.94%) DEGs (Fig. 2). 1238 and 880 DEGs were annotated by the COG database and divided into 24 categories. The “general function prediction only” was the largest category with 187 (15.11%) and 131 (14.89%) DEGs, followed by 151 (12.20%) and 128 (14.55%) DEGs in the “posttranslational modification, protein turnover, chaperones” category (Additional file 12). 1728 and 1207 DEGs were annotated by the KEGG database and classified into 128 and 124 pathways with 62 and 41 DEGs being in the pathway “purine metabolism” in LC_30_ and LC_50_ fluralaner-treated groups, respectively (Additional file 13).

As shown in Additional file 14, there were 43 and 30 DEGs of P450s after LC_30_ and LC_50_ exposure to fluralaner, respectively. Of these, 16 genes in both LC_30_ and LC_50_ treatment were overexpressed and annotated as CYP304A1 (gene9509), CYP49A1 (gene2558), CYP4C1-like (gene3595, gene9175 and gene9174), CYP4D2-like (gene11881), CYP4G15-like (gene13430), CYP4V2-like (gene3598 and gene10859), CYP6A13 (gene9333), CYP6B7-like (gene4502), CYP9E2-like (gene17041, gene1218, gene12726 and gene15894) and CYP12A2-like (gene11879). Six GSTs DEGs were found in both fluralaner-treated groups with only the “gene7753” (glutathione *S*-transferase 2-like) being significantly overexpressed. In addition, 3 CarE genes were up-regulated after LC_30_ exposure, and were annotated as “venom CarE-6-like” (gene8407), “CarE 1C-like” (gene5053) and “liver CarE B-1-like” (gene15885), which were not significantly changed after LC_50_ exposure. In exposure to both LC_30_ and LC_50_ fluralaner, a single chitin synthesis related gene annotated as “probable chitinase 10” (CHT10) (gene6005), and two juvenile hormone (JH) synthesis related genes annotated as “JH esterase-like” (JHE-like) (gene1042), and “troso-like protein” (Tsl-like) (gene8419) were down-regulated; the “JH acid *O*-methyltransferase-like” (Jhamt-like) (gene2891) and “Jhamt-like isoform X2” (gene2887) genes were up-regulated.

### Verification of the DEGs by RT-qPCR assay

**Fig. 4:**
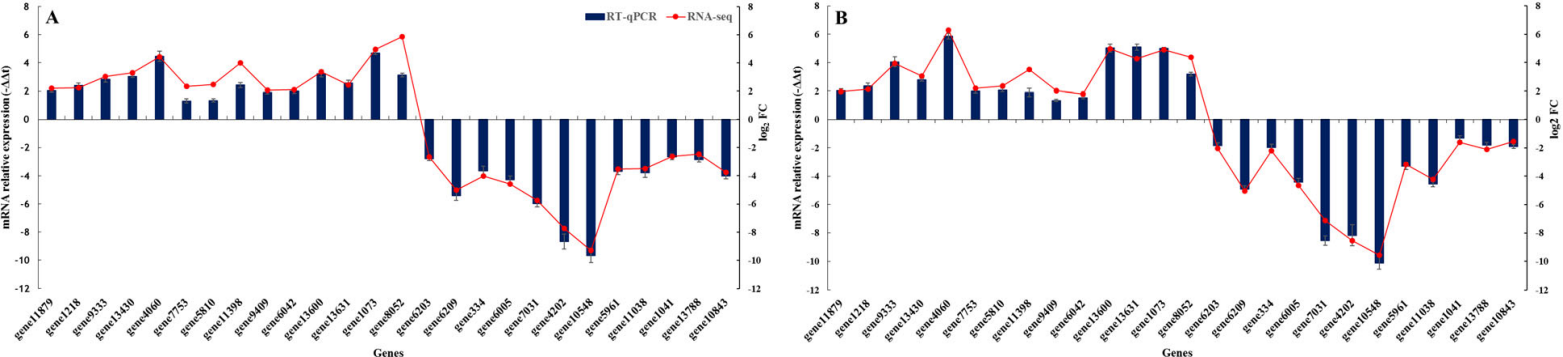
Validation of the DEGs by RT-qPCR. **a** represent the mRNA relative expression of DEGs after LC_30_ fluralaner treatment, **b** represent the mRNA relative expression of DEGs after LC_50_ fluralaner treatment. The left and right Y-axis indicates the mRNA relative expression levels based on RT-qPCR and the log_2_ FC based on DGEs’ analysis, respectively. The error bars represent the means and SE of three replicates

In order to confirm the quality of the *S. litura* transcriptome and the results of DEGs, fourteen up-regulated genes, including four P450 genes (gene11879, gene1218, gene9333 and gene13430), three vacuolar protein sorting-associated protein 13 (VPSAP13) genes (gene5810, gene11398 and gene9409), two pupal cuticle protein (PCP) genes (gene13600 and gene13631), one heat shock protein (HSP) 68 (gene4060), sodium-coupled monocarboxylate transporter 1 (SCMT1) (gene6042), protein takeout (PT) (gene1073), low density lipoprotein receptor adapter protein 1-B (LDLRAP1-B) (gene8052) and GST (gene7753) gene, and 12 down-regulated genes, including 3 P450s genes (gene13788, gene10843 and gene5961), 2 esterase genes (gene11038 and gene1041), 2 sulfotransferase 1C4 genes (gene6203 and gene6209), and 1 proton-coupled folate transporter (gene334), chitinase 10 (gene6005), gelsolin-related protein of 125 kDa (GRP) (gene4202), fibroin heavy chain (FHC) (gene7031) and fatty acid synthase (gene10548) gene, were selected for validation by RT-qPCR assay. Our results demonstrated that most of the selected up-regulated and down-regulated genes (71–92%) showed the same expression trends, respectively, compared with the transcriptomic results (Fig. 4).

## Discussion

In the present study, the transcriptome of *S. litura* untreated and treated with fluralaner was assembled based on the *S. litura* genomic database resulting in 16,572 genes being annotated according to NR, COG, GO, and KEGG databases. The number of annotated genes were almost consistent to those (16,161 genes) in a *de novo* transcriptome of *S .litura* [17], whereas more than those (11,692 genes) based on sequence homology of *S. litura* [18] and the reference genome (15,317 genes) [14].

In insects, insecticides often induce the increase of metabolic activity of P450s, GSTs and CarEs [19, 20]. P450s are members of an important metabolic enzyme superfamily, which are involved in the metabolism of xenobiotic compounds such as insecticides and plant secondary metabolites [21]. Generally, there are four large clades of insect P450 genes that are CYP2, CYP3, CYP4 and CYPM [22]. In the present study, less genes of CYP3 and CYP4 clades and almost equal number of genes of CYP2 and CYPM clades were identified when compared to those from the reference genome (61, 58, 11 and 8 genes of CYP3, CYP4, CYPM and CYP2 clades, respectively) [14]. The CYP3 and CYP4 are the largest clades and are mainly responsible for xenobiotic metabolism and insecticides resistance [22]. In this study, 90 genes accounting for 84.11% of the total P450 genes identified were clustered into the CYP3 and CYP4 clades, which were consistent with those containing 86.23% P450 genes in the reference genome [14]. In this study, 16 P450 genes belonging to the CYP4, CYP6, CYP9 and CYP12 sub-families were overexpressed in fluralaner-treated groups (Additional file 14). Similarly, butene-fipronil, phoxim, cycloxaprid and chlorantraniliprole could significantly enhance the expression of CYP304A, CYP49A1, CYP4C1 and CYP9E2 in *Drosophila melanogaster* Meigen, *B. mori*, *Periplaneta americana* (Linnaeus) and *Plutella xylostella* Linnaeus, respectively [23–26]. Also, CYP4V2, CYP6A13-like and CYP12A2 were overexpressed in the insecticide resistant populations of *Bemisia tabaci* (Gennadius), *Aphis glycines* Matsumura and *M. domestica*, respectively [27–29]. Conversely, reduced CYP6B7 was found to increase larval susceptibility of *H. armigera* to fenvalerate [30]. Methanol was found to up-regulate the expression of CYP4D2 in *D. melanogaster* larvae [31] and CYP4G15 is probably important for the metabolism of endogenous compounds in the central nervous system of *D. melanogaster* [32]. We therefore speculate that up-regulating expression of P450 genes belonging to the CYP4, CYP6, CYP9 and CYP12 sub-families are likely involved in fluralaner metabolism in *S. litura*.

GSTs are a class of multifunctional detoxification enzymes that play an important role in the metabolism of insecticides [33]. The total number of GSTs genes identified in this study was less than those in the reference genome (47 GSTs) [14] but was more than those in *B. mori* (23 GSTs) (Additional file 6). In both fluralaner-treated groups, six GST DEGs were detected and only “gene7753” belonging to the σ clade was up-regulated, to 5.10- and 4.61-fold in LC_30_ and LC_50_ treatment, respectively. The σ clade of GSTs play protective roles in processing xenobiotic compounds in insects [34, 35]. Similar to the *A. aegypti* GST-2 gene, which exhibited more than 8- fold mRNA levels in resistant strains than those in susceptible strains [36], the *S. litura* GST-2-like gene (gene7753) was over-expressed after exposure of fluralaner (Additional file 14) indicating that GST-2 may participate in metabolizing fluralaner. CarEs can hydrolyze a diverse range of carboxylates. In *T. cinnabarinus*, the expression levels of CarE-6 increased up to 1.64-fold under fenpropathrin-exposure [37] and the protein level of CarE-1 was significantly increased in a chlorpyrifos-resistant strain of the small brown planthopper, *Laodelphax striatellus* Fallén [38]. Under LC_30_ fluralaner-treatment in *S. litura*, venom CarE-6-like (gene8407), CarE 1C-like (gene5053) and liver CarE B-1-like (gene15885) were up-regulated by 2.64-, 4.06- and 2.57- fold, respectively (Additional file 14), which indicates that these CarEs may be involved in the metabolism of fluralaner.

Fluralaner mainly acts on ionotropic GABARs [39, 40], therefore the identified ionotropic GABAR subunit genes would be useful for exploring the relationship between fluralaner and the *S. litura* GABAR. As shown in Additional file 10, three genes (gene14064, gene14068 and gene15324) were classified as resistance to dieldrin (RDL) subunits whilst the others were assigned to ligand-gated chloride channel homolog 3 (LCCH3) (gene11829), CG8916 (gene11828) and an unclassified clade (gene6139) (Additional file 10). However, it was notable that gene6139, annotated as a GABAR δ subunit, was possibly not a typical GABAR gene according to the phylogenetic tree analysis (Additional file 10). Similarly, “gene9312” was annotated as a GluCl subunit, “gene1980” and “gene2657” were annotated as nAChR subunits, and “gene555”, “gene13723”, “gene15777” and “gene4992” were annotated as AChE subunits in the NR database but not in the phylogenetic tree (Additional file 10 and Additional file 11). Therefore, we speculated that the phylogenetic analysis is very necessary while annotating the new genes. Notably, the 8916 subunit is considered as a GABAR-like gene in the phylogenetic tree analysis (Additional file 10), in agreement with a previous study in *C. suppressalis* [41], despite there still lacking functional evidence to date [42]. Previous studies have found that insecticide-treatment can affect expression of RDL in various insect species [43, 44]. The over-expression of RDL1 (gene14064) and RDL2 (gene15324) after LC_30_ and LC_50_ exposure of fluralaner were similar to the relative mRNAs expression levels of GABAR from *Leptinotarsa decemlineata* (Say) treated with a sublethal concentration of fipronil [45]. GluCl mediates inhibitory synaptic transmission [46] and is identified as the secondary target of fluralaner [39]. Similar to many other species, such as *D. melanogaster* [47], *Apis mellifera* Linnaeus [46] and *Tribolium castaneum* (Herbst) [48], only one GluCl gene was identified in the *S. litura* transcriptome (Table 1 and Additional file 10).

**Table 1:** Putative insecticide-targeted genes identified in *S. litura*

Insecticide class	Target	Gene name	Gene number	Length (bp)
Cyclopentadienes; Phenylpyrazole	GABAR	RDL1 subunit	gene14064	8257
		RDL2 subunit	gene14068	7331
		RDL3 subunit	gene15324	1633
		8916 subunit ^a^	gene11828	1823
		LCCH3 subunit	gene11829	1566
	GluCl	GluCl	gene3536	1645
Pyrethroid	VGSC	VGSC	gene491	9853
		VGSC	gene9046	6872
Neonicotinoids; Sarcotoxin	nAChR	α1 subunit	gene8842	5075
		α2 subunit	gene8844	3665
		α3 subunit	gene9676	2010
		α4 subunit	gene9375	4199
		α5 subunit	gene7363	2055
		α6 subunit	gene7613	3853
		α7 subunit	gene7328	3269
		α8 subunit	gene8875	1809
		α9 subunit	gene863	1414
		β1 subunit	gene7327	2158
		β subunit	gene8228	1792
		β subunit	gene1694	1307
		β subunit	gene8227	2050
Organophosphorous	AChE	AChE1	gene1175	2608
		AChE2	gene9347	5592
Diamides	RyR	RyR	gene11613	16,054

Note: ^a^ the 8916 gene was considered as a GABAR-like subunit in *Drosophila* [47]

Sublethal doses of fluralaner was found to reduce larval body weight, decrease pupation and emergence, and cause notched wings of adults in *S. litura* [9]. The DEGs of CHT 10, JHE, Tsl and Jhamt genes in the present study may mediate these effects in response to fluralaner exposure. The expression of CHT 10, JHE, Tsl and Jhamt were changed in the fluralaner-treated group (Additional file 14). As is known, CHT are important enzymes for chitin degradation and reconstruction, and play important roles in the shedding of the old cuticle and peritrophic matrix turnover in insects [49]. JHE is the primary JH-specific degradation enzyme and indirectly regulates the JH titers [50]. Tsl is not just a specialized cue for Torso signaling but also acts independently in the control of body size and the timing of developmental progression [51]. Jhamt can transfer a methyl group from *S*-adenosyl-L-methionine to the carboxyl group of JH acids to produce active JHs in the corpora allata in insects (Jhamt in *D. melanogaster*). In line with the present study, knockdown of CHT10 induced lethal phenotypes, developmental arrest and high mortality in *Nilaparvata lugens* (Stål) [49]. Also, depletion of *B. mori* JHE by CRISPR/Cas9 resulted in the extension of larval stages [50] and Tsl mutation could lead to developmental delay in *D. melanogaster* [51]. Furthermore, overexpression of Jhamt resulted in a pharate adult lethal phenotype in *D. melangaster* [52]. Therefore, the transcriptional change of these related genes may contribute to the abnormal growth and development of *S. litura*.

## Conclusions

In conclusion, transcriptome analysis of *S. litura* based on the reference genome was provided in the present study. The identified DEGs may help uncover the possible molecular mechanisms underlying responses to fluralaner in *S. litura*. In particular, P450s may be involved in the detoxification of fluralaner *in vivo*. This study, therefore, may facilitate the identification of genes involved in fluralaner resistance and may offer useful information for exploring novel insecticides to control *S. litura*.

## Methods

### Insects and chemicals

A laboratory strain of *S. litura* was reared as described in our previous report [9]. Fluralaner (a.i. > 99%) was purified from Bravecto® (Merck & Co. Inc., Isando, South Africa) as described in Sheng et al. (2019) [53]. The 3rd instar larvae of *S. litura* were chosen for the experiment and three treatments (45 larvae per treatment) were used in the present study. Larvae in the control group were fed with artificial food containing acetone and in the fluralaner-treated groups with artificial food containing different sublethal concentrations [LC_30_ (0.59 mg of fluralaner per kg of artificial food, mg·kg^− 1^) and LC_50_ (0.78 mg·kg^− 1^), respectively] of fluralaner dissolved in acetone. After 24 h, 15 alive and normal *S. litura* from each treatment were randomly collected, equally divided into three 1.5 mL Eppendorf tubes, respectively, immediately frozen in liquid nitrogen and stored at − 80 °C until use.

### Total RNA extraction, cDNA library construction and IHX sequencing

Total RNA was isolated from whole body of *S. litura* from each group (the control and fluralaner-treated groups) by TRIzol™ Reagent (Invitrogen, Carlsbad, CA). The concentration and integrity of total RNA were measured using NanoDrop 2000 (Thermo Fisher Scientific, Waltham, MA) and with the RNA Nano 6000 Assay Kit by Agilent Bioanalyzer 2100 system (Agilent Technologies, Santa Clara, CA), respectively.

RNA sequencing libraries were generated from each sample and the sequencing was carried out by BioMarker (Beijing, China) following the manual of the NEB Next® Ultra™ RNA Library Prep Kit (NEB, Ipswich, MA) in Illumina. Briefly, 1 μg of total RNA from each sample was enriched using magnetic beads with Oligo (dT) and broken into short fragments, which was carried out using divalent cations under elevated temperature by adding NEB Next First Strand Synthesis Reaction Buffer (5x) (NEB). These short fragments of mRNA served as templates for synthesis of the first-strand complementary DNA (cDNA) with random hexamer primers and Moloney Murine Leukemia Virus (M-MuLV) Reverse Transcriptase (NEB). Using buffer, dNTPs, RNase H and DNA Polymerase I, the second-strand cDNA was subsequently synthesized. Remaining overhangs were converted into blunt ends via exonuclease/ polymerase activities. After adenylation of 3′ ends of DNA fragments, NEB Next Adaptor with hairpin loop were ligated for hybridization. The purified cDNAs were subjected to end repair by adding an adenosine triphosphate (A) base to the 3′ end and connected with sequencing adaptor. Suitable fragments of cDNAs were extracted by the AMPure XP system (Beckman Coulter, Beverly, CA). Three microliter of USER Enzyme (NEB) was used for size selection, and adaptor-ligated cDNAs at 37 °C for 15 min followed by 5 min at 95 °C before PCR. Then PCR was performed with Phusion High Fidelity DNA polymerase (NEB), Universal PCR primers, and Index (X) Primer (NEB). PCR products were purified with the AMPure XP system (Beckman Coulter) and library quality was assessed on the Agilent Bioanalyzer 2100 system (Agilent).

The clustering of the index coded samples was carried out on a cBot Cluster Generation System (Illumina) using TruSeq PE Cluster Kit v4-cBot-HS (Illumina) according to the manufacturer’s instruction. After cluster generation, the prepared libraries were sequenced on an Illumina platform and paired end reads were generated.

### Mapping and annotation of the clean reads

Raw reads in the fastq format were first processed by in-house perl scripts. Subsequently, clean reads were obtained from raw reads by removing reads with low quality, adaptor and poly-N. The intergenic regions and introns were identified by comparing the reads to the general feature format (GFF) file of the reference genome [14]. The clean reads with a perfect match or with only one mismatch were mapped to the reference genome of *S. litura* [54] by the Hisat2 software [55]. The mapped reads were assembled into the transcriptome by the StringTie method [56] and all the genes were functionally annotated and classified by Blast [57] according to the databases of Clusters of Orthologous Groups (COG) [58], Gene Ontology (GO) [59], Kyoto Encyclopedia of Genes and Genomes (KEGG) [60] and Non-redundant protein (NR) [61]. In addition, new genes from the unannotated sequences encoding more than 50 amino acids were identified and analyzed by Blast using the above mentioned databases.

### Identification of detoxification enzymes and insecticide-targeted genes

Genes encoding detoxification enzymes including P450s, GSTs, CarEs, and insecticide-targeted genes including ionotropic GABARs, GluCl, VGSCs, nAChRs, RyR and AChEs, were identified from the *S. litura* transcriptome. Referring to the previous studies [10, 21, 62], all of the confirmed protein sequences were aligned with *S. litura*, *B. mori, D. melanogaster* and *A. mellifera*, then the phylogenetic trees were mapped with 1000 bootstrap replications using the neighbor-joining method to evaluate the branch strength of each tree using MEGA 7 [63]. The phylogenetic trees were further annotated via the EvolView online tool [64]. Referring to previous publications [46, 48, 65], the phylogenetic tree of cys-loop ligand-gated ion channel proteins was constructed using Clustal X [66] and TreeView software [67].

### Identification and validation of DEGs

The relative expression levels of genes among the control, LC_30_- and LC_50_-treated groups were analyzed using DEGseq method [68], which provided statistical routines for determining differential expression in digital gene expression data using a model based on the negative binomial distribution. The fold change (FC > 2) and false discovery rate (FDR < 0.01) from corrected *P*-value were used as a judgment standard for the significant differences in gene expression between two samples [15]. The DEGs were annotated, classified and analyzed by databases of COG, GO, KEGG, and NR. The RT-qPCR assay was performed to validate the randomly selected 26 DEGs using TB Green™ *Premix Ex Taq*™ II (Tli RNaseH Plus) (Takara Biotechnology (Dalian) Co., Ltd., Liaoning, China) on a Quant Studio™ 6 and 7 Flex Real-Time PCR System (Life Technologies Corporation, Carlsbad, CA) according to the procedures from Liu et al. (2018) [9]. The housekeeping gene of EF-1α was selected as an internal standard to eliminate variations in mRNA and cDNA quantity and quality [69]. Three technical replications per biological replicate were conducted and the relative mRNA expression levels of genes were analyzed using the 2^-△△Ct^ method [70]. The primers used in this study are listed in Additional file 15. All values were expressed as means ± standard error (SE) and the statistical figures were constructed by Microsoft Excel (Microsoft, Redmond, WA).

## Supplementary information

**Additional file 1: Text S1.** Gene annotation with COG and GO databases.

**Additional file 2.** All pathways annotated by KEGG database.

**Additional file 3. **Cytochrome P450 nucleotide sequences of the *S. litura* transcriptome.

**Additional file 4. **Neighbor-joining phylogenetic analysis of the P450s genes from *D. melanogaster, H. armigera, P. xylostella,* *S. litura* and *B. mori*.

**Additional file 5. **Glutathione S-transferase nucleotide sequences of the *S. litura* transcriptome.

**Additional file 6. **Neighbor-joining phylogenetic analysis of the GST genes from *D. melanogaster, A. mellifera,* *S. litura* and *B. mori*.

**Additional file 7. **Carboxylesterase nucleotide sequence of the *S. litura* transcriptome.

**Additional file 8. **Neighbor-joining phylogenetic analysis of the CarE genes from *D. melanogaster, H. armigera, P. xylostella,* *S. litura* and *B. mori.*

**Additional file 9. **Nucleotide sequences of annotated insecticide-targeted genes of the *S. litura* transcriptome.

**Additional file 10. **Neighbor-joining phylogenetic analysis of the cys-loop ligand-gated ion channel superfamily genes from *S. litura* and other species. The subunits sequence used are as follow: Am-alpha1 (AAY87890.1), Am-alpha2 (AAS48080.1), Am-alpha3 (AAY87891.1), Am-alpha4 (AAY87892.1), Am-alpha5 (AJE70263.1), Am-alpha6 (AAY87894.1), Am-alpha7 (AAR92109.1), Am-alpha8 (NP_001011575.1), Am-alpha9 (AAY87896.1), Am-beta1 (AAY87897.1), Am-beta2 (AAY87898.1), Am-GluCl (ABG75737.1), Am-RDL (AJE68941.1), Am-GRD (AJE68942.1), Am-LCCH3 (AJE68943.1), Am-CG8916 (NP_001071290.1), Am-HisCl (ABG75739.1), Am-pHCl (ABG75741.1), Am-CG6927 (ABG75747.1), Am-CG12344 (ABG75746.1), Dm-alpha1 (AGB96296.1), Dm-alpha2 (NP_524482.1), Dm-alpha3 (NP_525079.3), Dm-alpha4 (NP_001097669.2), Dm-alpha5 (NP_995708.1), Dm-alpha6 (NP_995674.1), Dm-alpha7 (NP_996514.1), Dm-beta1 (NP_523927.2), Dm-beta2 (NP_524483.1), Dm-beta3 (AAF51485.1), Dm-RDL (NP_523991.2), Dm-LCCH3 (NP_996469.1), Dm-GRD (CAA55144.1), Dm-CG8916 (AAF48539.4), Dm-HisCl1 (AAL74413.1), Dm-HisCl2 (AAL74414.1), Dm-NtR (NP_651958.2), Dm-pHCl1 (NP_001034025.2), Dm-pHCl2 (NP_651861.1), Dm-CG6927 (AAF45992.1), Dm-CG7589 (AAF49337.2), Dm-CG12344 (NP_610619.2), Bm-alpha1 (XP_021203289.1), Bm-alpha2 (NP_001103397.1), Bm-alpha3 (NP_001103387.2), Bm-alpha4 (NP_001166816.1), Bm-alpha5 (NP_001166811.1), Bm-alpha6 (NP_001166813.1), Bm-alpha7 (NP_001166818.1), Bm-alpha8 (NP_001166817.1), Bm-alpha9 (ABV72691.1), Bm-beta1 (ABV72692.1), Bm-beta2 (ABV72693.1), Bm-beta3 (ABV45510.1), Bm-RDL1 (ADM88014.1), Bm-RDL2 (NP_001182629.1), Bm-RDL3 (NP_001182630.1), Bm-LCCH3 (BAT57341.1), Bm-GluCl (BAO58781.1), Bm-pHCl (BAX77827.1).

**Additional file 11.** Phylogenetic analysis of the AChE genes.

**Additional file 12.** Functional classification of DEGs according to COG database. Note: A represented the DEGs and all genes after the exposure of LC_30_ fluralaner, B represented the DEGs and all genes after the exposure of LC_50_ fluralaner, respectively.

**Additional file 13.** Functional classification of DEGs according to KEGG database.

**Additional file 14. **Changes of DEGs related to detoxification and development of *S. litura* after exposure of fluralaner.

**Additional file 15.** Primers for RT-qPCR validation.

## Data Availability

All data are presented within the article and the additional supporting files.

## Abbreviations

AChE: Acetyl cholinesterase
bp: Base pair
CarE: Carboxylesterase
cDNA: Complementary DNA
CHT10: Chitinase 10
COG: Clusters of Orthologous Groups
DEG: Differentially expressed gene
GABAR: γ-aminobutyric acid receptor
GluCl: Glutamate-gated chloride channel
GO: Gene Ontology
GRP: Gelsolin-related protein of 125 kDa
HSP: Heat shock protein
JH: Juvenile hormone
Jhamt: Juvenile hormone acid O-methyltransferase
JHE: Juvenile hormone esterase
KEGG: Kyoto Encyclopedia of Genes and Genomes
LC_50_: Median lethal concentration
LDLRAP1-B: Low density lipoprotein receptor adapter protein 1-B
nAChR: Nicotinic acetylcholine receptor
NR: Non-redundant
PCP: Pupal cuticle protein
PT: Protein takeout
RT-qPCR: Real-time quantitative PCR
RyR: Ryanodine receptor
SCMT1: Sodium-coupled monocarboxylate transporter
Tsl: Troso-like protein
VGSC: Voltage-gated sodium channels
VPSAP13: Vacuolar protein sorting-associated protein 13
