# Supplementary material for: Identification of transcriptome and fluralaner responsive genes in the common cutworm Spodoptera litura Fabricius, based on RNA-seq
Source: BMC Genomics. 2020 Feb 3;21:120. doi: 10.1186/s12864-020-6533-0 (PMC6998375; doi:10.1186/s12864-020-6533-0)
Supplement: Supplementary file 1 — Additional file 1: Text S1. Gene annotation with COG and GO databases. [file 12864_2020_6533_MOESM1_ESM.doc]

**Additional file 15: Text S1.** Gene annotation with COG and GO databases.

1). Annotated with COG database.

Among the 25 specific categories, the “general function prediction only” (889, 14.47%) was the largest category, followed by “posttranslational modification, protein turnover, chaperones” (682, 11.10%), and “carbohydrate transport and metabolism” (671, 10.92%), whereas only a few genes were annotated to “RNA processing and modification” (9, 0.15%) and “chromatin structure and dynamics” (7, 0.11%) categories. No genes were classified into the “nuclear structure” category.

2). Annotated with GO database.

The “biological process” category contained 21 sub-categories, followed by “cellular component” and “molecular function” categories with 16 and 14 sub-categories, respectively. In the “cellular component” category, the “membrane” and “cell” groups were most abundant with 2,543 (32.24%) and 2,091 (26.51%) genes, respectively, whereas the “virion”, “virion part” and “synapse part” sub-categories contained only 33 (0.42%), 33 (0.42%) and 28 (0.36%) genes, respectively. In the “molecular function” category, genes were mainly distributed in “catalytic activity” and “binding” sub-categories with 3,597 (45.61%) and 3,273 (41.50%) genes, respectively. The “protein tag” and “metallochaperone activity” sub-categories contained only 4 (0.05%) and 2 (0.03%) genes, respectively. It also showed that “metabolic process” (3,157 genes, 40.03%) and “cellular process” (2,807 genes, 35.59%) sub-categories were dominant in the “biological process” category, whereas the “cell killing” and “presynaptic process involved in chemical synaptic transmission” sub-categories contained only 3 (0.04%) and 2 (0.03%) genes, respectively. In summary, the “biological process” category possessed the largest amount of genes, and the “molecular function” category had the least sub-categories.
