# Supplementary material for: Identification of transcriptome and fluralaner responsive genes in the common cutworm Spodoptera litura Fabricius, based on RNA-seq
Source: BMC Genomics. 2020 Feb 3;21:120. doi: 10.1186/s12864-020-6533-0 (PMC6998375; doi:10.1186/s12864-020-6533-0)
Supplement: Supplementary file 3 — Additional file 3. Cytochrome P450 nucleotide sequences of the S. litura transcriptome. [file 12864_2020_6533_MOESM3_ESM.docx]

**Additional file 3**. Cytochrome P450 nucleotide sequences of the *S. litura* transcriptome

>gene10298

TTAAAGTATGTTCAAATTTATTCTTTTTGGAGAAAAAAAGGTGTTACCGGACCTAAACCGTATCCATTTGTTGGAAATTATGCACCGTTTATATTTGGAAGAGTAAGTGAAGGAGATTTGTTACGGAAAATATATGAACAATATCCAAATGAAAGGTTTGTTGGAATCTACAAAGGGGTCCGACCTGTTCTTCTTCTCAGAGACCCCGAATTGATTAAACTTGTGATGGTCAAAGACTTCAAATACTTCCCCGACAGATGCATTGAGAAGACCTTACAGATTGCCAAGAGCGTCTTCACATTAAAAGGAGATGAAGAATGGCACGCAAAAAGAAAATTGATGACACCAATGTACACAACATCCAAACTAACCAAAATACTTCCTTGTATTCAAAAATCCGCTGATAATTACGCAAAATACGTGAAGTACCTGATCGATAATAACATTGATCATGAGATCCATCATCTACAATCCAAGTTCATGCTGCAAAACGTTGGAATCTTAACATTTGGCGTAGAAATCGACCCTTTCTTTGGTGATAACGAACTTGAGACCGCTTTGATAGAAACAACGATCCATCCGCATCTAATAAATTCGCCAATACATATCGCCAACTACGTGTGTCCACCATTATTGAAAATGTTAGCACCTCTCCTACAAAAAATATATCTGAGAAGACTGATGAATTTCGGACATTACATAACGTCATTAATTAACAAGCCACGAGACGGTAACGATCTGAGTCACATCTTCTTGAACCATATGGTGAATCTAAAGTCTCGTGATGAGAAAGTGCTTAACAGTAAACTGCAAGCAAATGTAAATAACACAGACATTATAGAACAGACCTTGACTATTGCTTTAGCCAGTTACGAGACACCGTCTATTCTGTCAGGACTGATCCTTTACGAACTGGCTCTTCATCAGGACATTCAAGAGAAATGTTACCAAGAAATTACGACTGTGTTTGAAAAACACGACGGACAACTGTCCTTACAAGCTCTAAGTGAGATGAAGTACTTGGATATGATCTTTGATGAAACTCTGAGACTTCATCCTATGGCTCATGTCCTTGATCGAAGACCTTCTGCTAAATATACTTTTCCAGACACGAATGTGACCGTAGATAAAGATGTGTTTATTTTTATACCCACCCATGGATTGCAGCTGGATGCAAAATATTACGAGAACCCCTTAAAGTTCGACCCGGATAGATTTTTGCCGGAAAATAAGGATAAATTACAATCGTATACTTATTTGCCATTTGGATTTGGACCAAGAGCGTGTTTGGGTATAAGAGCATCAAAGGTGTTAGTATTATCAAACATATCTTTATTTCTTAGGAGGTTCAAGGTGTCACCGTCTGCTAAGACCAAACCAGTACTGAAGTTAGATCCAAAGAGGCTGCTCGCTAGTGTACCGTTAGGTGGAATTTGGATAAAAATAGAACAAAGGAATTGA

>gene10483

AACATTACCTGACGGTAGACTTCTCAGTAAAAAGAAAAAGCTAAATAACAAAAATGTATCTTAGCCAAGTGCGTAATTTTTTACCGTACTCAAGACTAGGATTGATGAACATCCAGAACAGATGTTACGAGAAAGTTGCTTTAAAGACTACGAAATCTCAAGAAATGGAGAACTCTAAGGCAAGCATGCAGTCTGTGGGTTTAACTCCTAAACAAATCGAAATGTTTCCAACAGCCAAGGTGCATATACCGATGGCGCTCACTAGTAGAGAGCCAATTATTTTACCTTTCGATGAAGTACCTGGGCCGAAATCTTTAAAGTACATATCTAATTTCCGTCAGTATCTTTCGGTAATAGGAACTCAGATCACAGTAAGTGTTCTCACGATGATGCTTAATGTGGGCACTTATGTGAATGCAAAGAAGCCACTGAAAAATCTCTCTGCACTCTTTGATGAATACGGTCCCGTAGTTCGATTCGTTAGCCCTGTGGGTGGGGACATAGTTTTAATAAATCACCCAGAACATATTCAAAAAGTGTACGCAATGGAAGGAAACTATCCCGTTAGATCGAATTTGGATTCTTTGGAAAAATATAGAGCTGAGCACAGGAATCAAATTTACGGCGGAACGTTTACCGCTTTCGGCGAGGAATGGCATCGCCAGCGGGAAGTTGTAGTAGCTCCTCTACATGAATCGGTGAGCAAACACATGGGTGGCATCAATGAAGTGTGCGAGAATTTTACCTTGAAGATATATAACATACGAAACTATCAAGACGAAGTTACGGACAACCTGTATAAGGAGATTTATAAGTGGTCCTTTGACTGTATGGGATTGATCCTTTTCTCGAAGAAGTTTACCATGTTGAATACGGAGGTGGTATACAGTCAGTGCGACATGTCGTGGTTGTACCACAGCCTGGAGAAAGCCACAGATGCTATAATCAAATGCGAGTCCGGACTGCAGTTCTGGAAACTACTGCCAACACCTACTTGGAGTTCCTTGGTCAAATATTGCGACAGCTTAGACAATTTGATCGGAAAGTATGTCTTGGAGGCAGAACAGGCCGTTGGTGTTCAACTCAAGAATGTAGGCCCTAATGTATCTGATGAATCATTTGAACTTGAAAATTCATTGGTCAATGCAATGTTGGTGGGTGAAAACAAAATGAGTGCTGAAGATATAGCAACAATTATTATGGATATGCTTCTAATTGGAGTTAACACTATTACATCTTCAATGTCTTTTCTTTTGTACCACATAGCGAAATACCAAAGGGCACAAAAGATATTATTCAGCGAAATTGACAAAGTTGGTGGCGACCTCAATATAAATAACATGTCGGCGATTATTGAACAAACTCCATATCTTCAAGCATGTATAAAAGAGTCTTTACGACTGGTTCCACCAATTCCTGTGCTAACCAGAATCCTACCAAAAAATATAACTATTGACAGATACAATATACCTCGTGGTACCCTTATTATTATGTCTACTCAAGATGCATCTCTCAAAGAAGGGAACTATGATGATGCATCAGTGTTCTACCCTGAGCGGTGGTTGAAGGGTGATGCAAAGGAATACCATGCTTTTGCATCTATACCATTTGGTTTTGGAGCCCGTAAGTGTTTGGGCCAGAACATAGCAGAGACAATGCTCTCTTTGCTTACTATCAGGGTAAGCCAATATCCTTACTTTTTTAAATATATTGGATCTTTGTTTGTATTTATAGAAAAAAATTCAACAGATTTCGTAGCCTCATTTACTAGCTTTACAAAATTTTTAGATTCTCCAAAAGTTTAAGCTGGAATACCATTATGGAGATATTGGATCAACAAGAAGTTTTATATCAAGACCTACTAAGGCACTTAAGATTAGATTCGTGGA

>gene10840

GTTAGTGTTAGTGTTTGTGGTATGTTTGGTGGTACTCAGTGTATGGCTAAGGGTTAGGTCGAAGAACCTAATGCCTCCAGTTGTCCCTGGGGCTCTTCCACTAATAGGACATACGCATCGACTCACTGAATTTGAAAAAATATGGAATATATTGGAACAAACAAGTATATTTACATTGAATAATGGAGGAATTTCCGTTATAAAAATCTGTGAATATAACATATATATACTTACGGATCCGGATCTCAGTCTCATGGCGTACAATATTTGCTTGAACAAAATGTACTTCTACCATTTCACTGATGATCTTGTGGCTAATGGACTCATTACGTCTGAAGCCCAAACTTGGAAAATACACCGAAAGTTAATAAATCCAGTGTTCAATCAACAAAACTTAAACAATTTTATAGACGAAATGAATGTGCAAACAAAACGGCTCGTCTCTGAATTATCTACTATGCTTGGAAAACCAGTAAATATACGCAAACCTCTTGTCAAATATACAATGAATATTACTAGTCGGACTACTTTAGGATTAACTGCAGAAGATCAAACTTTAATCGAAAAGGATTATGCGCACGCATTTGAAGTTTTATCGGTATTATATTATGAGAGGGCTACGAAACCTTGGCTGCACTTACCATTCTTCTTTAAAAAAAGTGCATTAAAGCGTAAACAAGACAAACTTACGGAAACAGTGAAAAATATTCTTACCCCCATAATACAAAAAAGGAGATTGGAAATGAAGATGAATTCGTACATCAACAACGAAAATGATGATAAATTCCAACCTGTGTTAAATCGTCTATTATATCTAGCCGATGAACAAAACGCTTTCACTGACACCGAGATACGGGAACACTTGAACACTTTTGTGATTGCTGCTTACGACACTACTACAGTATCGCTCACACTCATACTGATGATGATTGGTTGGCATAAGGATGTACAGGAACGGATTTACAATGAAATACAAGAAGTATTAAATAATGAAGACAGAGACTTCACAAAGAATGATTTGCCAAAATTAGTGTATGTGGAAGCGGTAATCAAAGAAACATTGAGGTTGTACCCAACTATACCGTACATCGGTCGCCAAGTTAATCGAGACATCGTATTAGGAAAATATACCTTACCGGCTGGTAGTACGTGTGGAATTTCATTGTATGGCATCCATCGTCACCCTATGTGGGGAAAGGATGCTGATCAGTTCATACCAGATAGATGGCTCGATCCTGCTCGGCTGCCGAATCACCCAAATGCTTTCGTAGCCTTTGGTGTAGGCAAAAGATATTGTATAGGTAAACAATATTCCATGATGGCGATGAAAACAGCAATAGGACATATAGTACGCGAGTACCACGTGCACAGTGATCTTAGCAGGCTGAAATTTCAATATGAAGTTGTACTGCAACCGACGAAGGGTCATCGAATTACATTTACAAAAAGAAAATAATTTTCGAAAATCTTTGAAAAAAAAAGATGTAGCGAACGTTAGGTCGGATAAGAGGACCCAGTATGAGTTTGTTATGTTTGGCGAGATCGATCCATTACCACATTTAGCTCTATAATTGCTTGGTTTAATGGAGCTGGCCAACCAGAGCGCCGAACGTGGTAACATTTCAATCTCGTTAATCGTAAAACTAAGCCCTCTGGGTGTGATCATGATTACCCATTAGTTTAACTTTGTAACGACAATAGGGTAGATGACTAATTTTTATTTTAATGTCCGCCTGGTAGATTCTTTCAAAACATTAGACACG

>gene10842

CAGCATGTCGGTGATCGCATCCATTTTAATCTTTTGCATTGTGTTTATATTGTGGTTCTGGTTAAGGCCCAGAAGCCCAAATGCGCCCCCGTGTTATCCTGGTGCATTGCCATTTATAGGCCATGGACATCAAATTTTTGGAAATCGTAAACACCTATGGAGATACTTAGAAAAAATATTTCATTATTCTTTGGAGAAGGGTGGTGTTCTTGAACTACGGATTGCAACGCACTGTGTTTATGTTTTAACTGACCCTGATGAAATTCTAACAGTTTCGAATACCTGCTTGAACAAAGCGTACTTTTATGAATTTGGAAAAGAATATTTCAAAAATGGATTGATCACTTCTAAAGCATCAATGTGGAGGAATCATCGCAAACTTCTAGATGTGGCCTTCACTCAGAAAGTTCTGGATACATTTGTTGATGAAATGAATGCACAGACTCGGGTCTTAGTCACTAATTTAAATGCTCAAGTTGGTAAAGGACCATTCGACGTGCGCCACTACTTGATAAATTATACGTTGAAGACTGTCAGTCGGACATCGCTAGGACTAGAAGCTAAAGACCAGACAATAATAGACGAAGCGTACGCTGATGCATTAGAAGATTTGATTAAAGTTTACTGTGAGAGAGCTCAGAAAGTGTGGTTGCATCTCTCTTGTATCTACGATAGAAGTTCTTTGAGGAAGAAACAGGACTACCTGCTGAAGATCATGAATAATATTATAGATGCTGTTATTGTACAACGCAAATCGGATTTAAAGCTCAATAACAATGAATCTACACAATATGACCAACCAAAAAAAGGTAAATTCAAACCAGCTTTAGATCAAATGCTACAGTTAGCTGATGAGCATCATGTCTTCAATGATGAAGAGATTCGAGAACACCTGGATACCTTCGTGGCAGCTGCTTATGATACAACTTCTGGAACCCTAATTTATGTATTATTAGCCATAGGATCACACCCAGAAATACAAGAGAAGATTTTAAAAGAAGTACAAGAAGTTCAACCGAACAAAAACGAAGATATCTCGAAATATGATCTTTTGAAATTGGTGTATGTAGAAGCAGTGATCAAAGAGGCACTTCGGTTGTACTCGCCATTGCCTGGAGTCGCAAGGAAAATTGAGAAAGATGTTAAATTAAAAAACTACACATTGAGAGCAGGCAGCACTTGCATTCTAAGTCTATATGGGGTCGGACACCACTCCATGTGGGGTCCCGATGTAAATGAGTTCAAACCAGAGCGGTGGTTTGATATCGCAAACTTTCCAACTAACCCTAATGCATTTGGTGCGTTTGGAATCGGCAGACGAAACTGCATTGGTAAACAATTCGCCATGTCGCTGATGAAGTTAACAATCGTGTATCTCGTGCGAAATTATCATATTTCAAGTGATATAAGTAAACTTGAATCCGAATATGATATAGTGCTCAAAGCAGTGTCAGGGCATCTAATAAGTATTACTTCTAGAGTATAAGGCATAAAAAGATATAATAATCAAAAATAAATGTTAGAACTATATTTGTATATTTTTGGTATCTTCAGTATCTGTTGATTTCAATGTTTTACTTGAATTAATATCTAAATCTTTGTGTACAGAGAATATTGCAATATTCCAATGCCATTGACCTGCAGTCATA

>gene10843

TATGTGTTAGTGCTAGCTGTGATAATTGGTGTCTTGCTTTCATTATGGTACTGGACAAGAAATTATTCTCGTTCTCCTCCAATATATCCAGGGGGATTACCGATTATTGGACATCCTTTTATGGCCATGAAGCACCGCAAAGATTTGTGGGGTTTAATGCAAACTGTTGCTGATTACGGCTTGGAAAACGGAGGTATAATCCAAATTTGGGCAGGACCTGTCACAGCTTATGTTGTGAGTGATCCAGAAGTCGTCGGAAACATAGCAAACACATGTATGGAAAAGCCATTTCAATACAATTTCTTAATTGATAGCATAGGAAATGGATTGATAACATTGAATGGACCCACGTGGAGGATACACAATAAACTACTAAGTCCGTCTTTCAACCAACAAGTATTAAACACGTTTCTACCTGAAATGAATGTACAGACTCGAAATATAGTCGCACAAATGACTACGGTGGCTGGAAAGGGACCAGTTGATATCAAGGAATTTATAACTCAATATATACTAAGATCAGTTTGTAGAACTTCACTGGGATTGGAATCTAAAGATCAAGATATAATCGACAATGGTTACGCGAAAGCGCTAGAAGAAATCTTGAGCATTGTATGCTACCGTGTTTTAAATGTGCATGTATATCTTTCATGTATTTACAACTGGACGTCAATGAGAAGAAGAGAATTGGAACTAGTCAAAATCGTTAAGAATATGATAAATCCTGTAATTCAAAAACGCAAATCTGAATTAAAACCTACAAAAATGTATAATAACGATAATTCAACAACAGTTTCAGGTAAATTCAAACCGACGCTAGATTTGATGCTGGATTTGTCTAATGAACAAAACGTCCTCTCCGATGATGATATTAGGGCGCATCTGAATACTTTCGTAGCAGCTTCTTATGACACAACTTCAGCAGTATTGCATAATGCGCTGATGGCGCTTGGATCATATCCTGATGTGCAAGAACGAGTTTATGAAGAGGTTCAAGACGTTTTCCAAAATAATGAGGAGTTGACGAAACACGATATGTCTAAACTTGTTTACTTAGAAGCTGTGATAAAGGAGGTATTGAGGGTATATGTCGCAGTTCCATTGGTGGCAAGAAAAGTTGATGCCGATATTGTACTGCCGAAATATACATTGAGAGCGGGAAGTATATGTATCCTATCAATATATGGCCTTCACCGTCACCCCTCGTGGGGACCAGACGCGAAAGAGTTCAAACCGGAGCGATGGCTGAATCCCGATACATTACCTACTAACCCTAACGTATACGCTCCGTTCAGTTTTGGCAAGCGAAACTGTATTGGGAAACAGTACGCCATGATGAGCCTGAAGACGTCGATAGCGCATATAGTGAGGAAGTTCCGCATTACTGCTGATATCAATATTTTGAAGTGGAGATACGAAGTTGTATTAAAACCTACAACAACTCCTCTTGTCAATTTAACATTAAGGTATTAA

>gene10844

ATGTGGAAGATACACCATAAATTATTAAGCCCAGCGTTCAGTCAGCAAGTCCTGAATACGTATTTGAATGAAATGGACGTACAAGGTCAAAATCTAGTCTCGCAATTGGCAACAGTGGCGGAGAAGGGACCAGTTAATATCACTGACTTCTTATTTAAACATGTCCTAAGAACAGTTTCCCGTACGTCACTGCGGCTGGATGCTAAAGATCAGGATATGATTGACAATGACTACGTTGAAGCGGTTGAAGAAATAGGAAACATTATAATTCATCGCGCTTTAAGCCCACTGTTACATCTTTCATTTTTATTCAATAGGACCGCAATGAAGAGAAGGCAAGTGGAACTAAGTAGAGACAACAGGAAGCTGTTAGATCCTATAATTCAGAAACGAAAATCTGACTTAAAATCAACTAAACATACAGTCAATAATAATAAAGAGTATTCAGTACCAGGTAAATTCAAACCTATATTAGATTTGTTGTTGCATTTATCTGATGAACAATATGCCCTCTCCGATGATGAAATCAGGGCGCATCTGAATACTTTTGTGGCAGCTTCATTCGACACAACTTCATCAGTACTTCAGACCGTGTTGTTAGCGCTTGGATCATATCCTGATGTACAGGAACGAGTTTTTAATGAGATCCAAGATATTTTTGAAAATAGAGAAGAGGTGATTAAACATGACCTGGCAAAACTCGTTTACTTGGAAGCAGTGATAAAGGAGGTATTGAGGGTTTATAATGTAGTTCCTATGATAGCGCGGAAACTTGACAAAGATATTGTACTCCCAAAATATACTCTACGAGCTGGAAGCTCCTGTATCCTGTCGATATACGGCCTTCACCGTCACTCCTCGTGGGGCCCAGATGCGAAGGAGTTCAAACCGGAGCGATGGTTGAATCCCGAAACTTTACCTACTAATCCCAACGTATACTCCCCGTTTGGTATTGGCAAGCGAATTTGCATTGGAAGACAGTACGCCATGATGTCCATGAAGACGTCGCTTATCCATATCGTACGAAATTTCCACATTTCTGGTGATATCAGTTCATTGAAGTGGAATTTTGAAGTCGTACTAAAAGCAAAAAATCCTCTTATTACTTTGACGTTAAGGTCTTAA

>gene10845

ATGACTGTGTTGGTGCTAGCTATGATATTTAGTGTAGTGTTTGCAATATGGTGGTACTGGTGGAGACCTTGTCCTCGATCACCTCGCACGTATCCTGGTGCATTGCCCATCATTGGGCACATTGTTGAGGCAATAAAATATCGCAATGACATATGGAGTTACATGGAACATGTTGCTGATGATATAACAGATGAGTGTGTTCAGTTAAAGATGGGTCCCCATATCATTTATGTTGTAAGTGACCCAAGTGAAATCAGTGTTATAGCAAACACATGTTTGAAGAAATCATTCTTTTACAAGTTCTTGGTTGACCTATTAGGAAATGGACTTCTGATAGCAGATGTGCCCACATGGAAAATACATCGTAAGCTTCTGAATCCTGCATTCAACCAGAAAGTACTGAACACATTTGTAAATGTTATGAATGTAGAAGCACGAATTTTAGTCACACAATTGAAACCTGTAGCGGGACAGGGACCAGTTGATGTCAAACAATTTATAATTAAATATGTTTTAAGATCGGTTTGCAGAACATCTTTGGGATTGGAAGCTGATGATCAACACATGATTGACAAGGAATACGCGCAAGCAATCGAAGAAACCATAGCAATAGTAATAAAACGAGGTTTAAACGTGGCATTACATCCTCCATTTATTTACAACATAACTGCAATGAGAAAAAGAGAACAGGCATTGATCACGAAGATAAAAAACATTTTAAATATCATAATCCAAAAACGAAAATCTGATTTGAAATCCACCAATAATAAATATATGACAGACTTCAATGATGAGTCAGTGAATGGTAAATTCAAACCTATACTAGATCTGATGCTGCATTTATCTGATGAACAACAAGTTTTATCTGATGATGATATTAGACAACATATTGATACTTTTGTATTTGCTTCTTACGACACAACTTCAGCAATGTTACAGACCACATTACTAGTGCTAGGATCACATCTAGATGTACAGGAACGAGTTTATAAAGAGATTAAAGAAATTTTGGGAAAGAATGAGGAATTGACTAAATACGACTTACCAAAATTGGTTTACTTAGAAGCTGTGATAAAAGAGGTTTTGAGGCTATATTCTATAGCTCCATGGGTTTCGAGACAACTTGATACTGATGTTGTTTTCCCAAAATATACTTTAAGAGCTGGAAGTACATGTATTCTGGTATTATACAATCTTCACCGTCACCCCTCGTGGGGACCAGATGCGAAGCAGTTCAAACCGGAGCGATGGCTGAATCCCGATACATTACCTACTAACCCCAACGTATACGTTCCGTTCGGAATTGGCAAACGAAACTGCATCGGGAAACAGTACGCTATGATGACCCTAAAGACGTCGGTAGCGCATATCGTACGAAATTTCCAATTATTTGCAGACATTAGTCATTTGAACTGGAAATATGAAGTTGTATTAAAACCAACAAAACCTCTCGTTAGTTTTACGTTAAGATCGTAA

>gene10846

ATGACTGTGTTGGTGCTAACTATGATATTTAGTGTAGTGTTTGCAATATGGTGGTACTGGTGGAGACCTTGTCCTCGATCACCTCGCACGTATCCTGGTGCATTGCCCATCATTGGGCACATTGTTGAGGCAATAAAATATCGTAATGATATATGGAGCTATATGGAACGTATTGCTGATTATATGACAGACGAGTGTGTGCAACTTCGGATGGGTTCCCATAAAATCTATGTTGTAAGCGACCCCGATGAAGTTGGTGTTATAGCAAACACGTGTTTGAATAAATCATTTCTTTACAAGTTTCTAATCGATGTATTAGGAAATGGATTACTATTATCAGACGTACCCACATGGAAATTACATCGTAAGCTACTGAATCCAGCATTCAACCAGAAAGTATTGAACACATTTGTAAATGTTATGAATGTAGAAGCACGAATTTTAGTCTCACAATTGAAACCTGTAGCGGGACAAGGACCAGCTAATGTCAAGGAATTTCTAATTAAATATATTTTAAGATCGGTTTGCAGAACATCATTGGGATTAGAAGCTAAAGATCAAGATATGATTGGTAAGGAATACGCACAAGCAATAGACGAAATCATAACAATAGCAGTAAAACGAGGTCTAAACGTAGCATTACATCCTTCATTTGTTTACAACATAACTTCGATGAGAAAAAGAGAACAGGAACTGGTGACGAGGATAAAGAACATTTTAAATAGCATAATCCAGAAACGTAAATCTGATTTAAAAATTACCAATTCAACAAATTACGATGATGGCTCAGTTAATGGTAAATTCAAACCTATCTTAGATCTGTTGCTGCATTTATCTGATGAACAATATGTCCTCTCAGATGATGAAATCAGGGCGCATCTGAATACTTTCGTAGCAGCTTCTTTCGACACAACTTCATCAACACTTCAGACTGTGTTGTTGGTGCTTGGATCATATCCTGATGTTCAGGAACGAGTATATAGAGAAATAAACGAAGTATTCGGAAATAAACAAGAATTGACTAAATATGACTTACCAAAATTGGTTTACTTAGAAGCAGTGATAAAAGAGGTTTTGAGGATATATTCTATAGCTCCTTGGGTATCAAGACATATTGACACTGATATCGTTTTCCCAAAATATACATTAAGAGCTGGGAGTACATGTATTCTGGTATTGTACTACCTTCACCGCCACTCCTCGTGGGGACCAGATTCCAAACAGTTCAAACCGGAGCGATGGTTGAATCCTGACACTTTACCTACTAACCCTAACGTATATGCTCCCTTTGGCATTGGGAAACGAAACTGCATCGGAAAGCAGTACGCTATGATGACCCTAAAGACATCGGTTGCGCATATCGTTCGAAATTTCAAATTATCTGCAGATATTAGTCAATTGTACTGGAAATATGAAGTTATATTAAAACCAATTAACCCAACTCTCGTTAGTTTTACGTTAAGATCTGAATTTAAAATCTTTATGTACAAATAA

>gene10847

TTGTAAGTCTGTGTTAAATAAAAAAATATAAAGATGTTAGTCGTGCTATTTGGTGTTACAGCTGTATTATTGTGGTACTGGTGGAAGCGTCATCCTCGTTCACCTCCGTCGTTTCCCGGTGAACTGCCGATCATTGGACATGTCTTTGCAATGTTAGGATATCGAAATGATATATGGACCTTCATCAAACGAGTTTGTGATTACTCTTTGGAAAACGGTGGATTAATTCGTCTTAACGTCGGTTCTCATATTATATATGCGACTACATGGAAAACACATCGTAAGCTATTACATCCAGCCTTCAATCAGCAAGTTTTAAACACATTTATAAACGAAATGAACGTACAAGCTCGAAATCTCGTCTCACAATTAACTGCAGTGGCTCAAAGTGGATCAGTTGATATTCACCAATTTGTGATTAAATATGTCTTAAGGATCGTCTGTCGAACATCATTAGGACTAGAAGCTAAAGATCAAAATATTATCGACAACAATTATGCAAATGCTATCCAAGAAATTTTGAAAATATTCTGCTATCGTGGCATTCATCCTTGGTTATATCCACCGTTTATATATAAAAGAACTGCTTGGAAAAAGAAAGAAGACGACTTAATAAGAGATCTTAAGAACATGATAAATACTGTTATCCAAAAACGGAGGTTTGATTTAAAGGCTAACAACTTTGTTACAAATAATGGCGTTGATTCAATAACGGGTAGATTTAAACCAGTCCTAGATCTTCTACTGCATTTAGCTGATGGAGAACACGCCTTTACTGATGATGAGATCAAAGAACATCTGGATACTATTGTAATAGCCTCTTACGATACAACATCAACGGTACTACAAACTATATTGTTAGTGCTTGGAACATATCCTGAAGTACAGGAACGAGTTTATAACGAGGTCCAAGAAGTATTTCAAAATAGTGAAGATTTATCTATGCTTGATTTATCAAAACTCGTTTACTTGGAAGCGGTAATAAAGGAGGTATTGAGGGTATACAGTACAGTTCCTATAGTTTCAAGAAAACTGGACACCGATATTGTACTGCCAAATTACACCTTACGAGCTGGAAGTACATGTATCCTGTCGATATATGGCCTTCACCATCACTCCTCGTGGGGACCAGATGTGAAGGAGTTCAAACCGGAGAGGTGGCTGAATCCTGATACTTTACCTACTAACCCCAACGTATACGTTCCGTTCAGTATTGGCAAGAGAAACTGCATTGGGAAACAATACGCTATGATGAGTCTAAAGACGTCGCTTGCACATATCGTGAGGAAGCTCCACGTTTCTGGTGACATTAGTAATATGAAGTGGAAATACGAACTAGTGTTGAAACCAGCAAAACCAGCTCTTATCGAGTTTACTATAAGATCTTAATTTATGTACATATTAAAATTGTTTACATT

>gene10848

TTTTTGGAAATGTATATAGCAGTAGGATTGGTGTTTTTATGTTTCGTGGTGCTAACTGCGTGGTCGACGTGGTTCAGGCAGAAACCTGATGCACCTCCTTTGATGCCAGGCTCACTCCCGATTATAGGACATTCACATCTCATAATCGGAGACCGAAAACATCTCTGGCGCTTTATACGGCATGTCAACGAATCATCACTCGAGCATGACGGAGCAATTGAAGTATGGTTTGGCCCAAACATATATTACATTATCACGGATCCAGAGGATTGTCTTACTCTAGTCAACGCATGCTATAGTAAACCTTACATCTATGGTTTTGCAAAGGAATTTTTAAATAATGGCCTGATTACCGCGGATGTTTCTATTTGGAAAAATCATCGTAAATTATTAAATCCTGCATTCAACCAGCAAGTGTTGAATACTTTTATCGATGAAATGAATGTGCAGTCTCGAAATCTCGTTGCAAATTTGAAGAATGAAGTTGGACAAGAGCCTTTTGATGTTCGACACTATTTAATTACGTTTACGCTATCAACTATTAGCCGAACTTCACTAGGTCTAACAGCTCAAGAACAAACGCAAATAGACAGAGACTATGCGGTGGCTATTGAAAATTTGTTGGCTCTGTACTGTGATAGGTTCCAAAAAGTATGGCTGCATATTGGTTGTATATTTGACTGGAGTGCATTGAAACGAAAACAAGACAAATTAACGACAACACTGAAGAATATTATGAATCCTATTATACTTAAAAGAAAATCAGAGATGAAGACTAAAATAGAAACTACGCATTATGACGACACACCCGGAAAATTTAAACCAGTTCTAGATCAGATGCTTCAAATGTCCCATGAGCAAGATGTATTCTCAGTTGATGACATCAGAGAACACTTAGATACATTGGTAGCAGCATCATATGACACAACCTCTTCAGCTTTAAGCTTCATACTATTGGTGATTGCGACTTATCCAGAAGTACAGGACCGAATATATAACGAGATACAAGAAGTATTGCAAAACAAAGACGACGATTTTTCAAAACACGACCTACAGAAACTTGTATACTTAGAAGCAGTTATGAAAGAAACAATGAGACTGTACCCCGCTGTTCCAATTGTAGCCAGACAAATTGACGTGGATGTTAAATTGAAAAACTGGACGGTACGCGCCAACACAACTGGTATAATTGGTGTGCACTCTCTTCACCGCCACCCTCTCTGGGGTCCTGATGCAAATGAATTCAAACCAGAGCGCTGGTTGGATCCTAGCCGCTTGCCTGATAATCCCGTCCTCTTTGCTGCCTTCGGTATTGGCAAACGAAACTGTATAGGAAAATTGTTCGGGATGCTGATGATGAAGACCGCACTAGCACACATAGTACGACAATATCACATTTCTGGGAGCATCCATAATGTAGAATGTGAATTTGATGTAGTTTTGAAACCTGTAACTAATCAACATATCCAATTAAAATTAAGGTCATAGGGTTGTACCAAAAAGGCTGTATCATAGATTAAGATTATAGGACTTAAAGTAATTATTAAATAATATGTGGCGGTTAATTAGATGATAATATTATTATTATAATGTAATATATATGTCTATTTAAAATCACGCTAGTGTGTTTTAAAAGAAATTATTTTTACTGTTTCTATTTATTTTATCCTGTCACTGTCATCCTGTCCTCCTTACACATTCTGATTATATACCATCACGTAGGTATGTATCATATTTTTTTGTGTCTTTGTCTAACCATACAATCTTGCTACCATTCGTGCGTCACTGCATGGGTGCCCACTGAAAATCAGTGCTGAGTGACTTGTATGCCTAGCTAACAC

>gene10851

ATGGCAGCAACGACTGTCACAACCGAGGCCTGGGCTCGCTGCCATTGCAGCGATTTATGGGGTTTAATGCAAAATGTTGCTGATTACGGCTTGGAAAACGGAGGGATAGTCCAACTTTGGGCAGGACCTATCATAGCTTATGGACCCACGTGGAGGATACACAATAAACTACTAAGTCCATCGTTCAACCAACAAGTATTAAACACGTTTCTACCTGAAATGAATGTACAGGCTCGAAATATGGTCTCACAAATGACTACTGTGGCTGGAAAGGGACCAGTTGACATCAAGGAATTTATAACTCAATATATACTAAGATCAGTTTGTCGAACTTCACTGGGATTAGAATCTAAAGATCAAGATATAATCGACAACGGTTACGCGAAAGCGCTAGAAGAAATCTTCAGAATTGTATGCTACCGTGCTTTAAATGTGGATGTATATCTTTCATGTATTTACTACTGGACGTCAATGGGAAGAAGAGAATTGGAACTAGTCAAAGTCGTTAAGAAAATGATAAATCCTATAATTCAAAAACGCAAATCTGAATTAAAACCTACAAATATTGATAATTACGATAGTTCAACAACAGGTAAATTCAAACCTACGCTAGATTTGATGCTGGATTTGTCTAATGAACAAAACGTCCTCTCCGATGATGATATTAGGGCGCATCTGAATACTTTCGTAGCAGCTTCTTATGACACAACTTCAGCAGTATTGCATAATGCGCTGATGGTGCTTGGATCATATCCTAATGTGCAGGAACGAGTTTATGAAGAGCTTCAAGACGTTTTTCAAAATAATGATGAGTTGACGAAATACGATATGTCTAAACTTGTTTACTTAGAAGCTGTGCTAAAGGAGGTATTGAGGGTATATGTCGCAGCTCCATTGGTGGCGAGAAAAATTGATGCCGATATTGTACTGCCGAAATATACATTGAGAGCGGGAAGTATATGTGTCGTATCAATATATGGCCTTCACCGTCACCCCTCCTGGGGACCAGACGCGAAGGAATTCAAACCGGAGCGATGGCTGAATCCCGATACATTACCTACGAACCCCAACGTATACGCTCCGTTCAGTTTTGGCAAGCGAAACTGTATTGGTATGATCACTTGA

>gene10852

ATGGTGTTGCTGTGTCCGTCAATCAGCGGTCTCAGGTGGCTCCTAAGAAAGTGTGAAGATTATGCGGCGGCTCATGGGCTCAGGTACAATGCTAAAAAGAGTGAACTACTTGTCTTTAAAGCGGGCACCAAATGTTACTCGAATGTGCCACAAGTTACCATTTCTAATTCACCATTGACTAGAGTATCTCATTTCAAGTACTTGGGTCACTGGGTCACCGAGGATCTCAATGAGAACATGGACATTGAACGTAAATTTAAACCAATTCTTGATCAGATGCTTCAAATGGCAAGTGATCATGGAGTATTCTCAGATGAAGACATCAGAGAACACTTAGATACTCTCGTAGCAGCATCGTATGATACAACTTCTTCAGCGATGACCCACATGTTGCTGGCGATTGCAACTTACCCAGAAATACAGAACAGATTATATAATGAAATACAAGAAGTTCTGCAGAACAAAGATGATGATTTTACTAAACATGATCTACAGAAGCTGGTCTACTTAGAGGCAGTAATGAAAGAGTCGTTGAGATTGTACAGCGCTACTCCACTTATAGGGAGAAATATTGATGTGGATGTTAAATTAAAAAACTGTACAATACGTGCCAACAGCACTTCTATCATTGGTGTGTACGCTCTCCACCGTCATCCTCTATGGGGTCCCGATGCAAATGAATTCAAACCAGAGCGCTGGTTGGATCCTAGCATTTCTTCATTACTTGAGAAGCCTGCCCTCTTTGCTGCCTTTGGTATTGGCAAACGAAACTGCATCGGAAAATCGTACAGCATGCTGATGATGAAGATAGTACTAGCACACATTGTTCGACAATATCATATTTCTGGCGACATTCACAAAGTAGTATGTGAATTTGACATAGTTTTGAAACCGGTATCAGGACACCATATTGGTTTAAAATTAAGGTCTTAAGATTGTAAAAATAATTGCTCTGTAATGCCTAATGATGGAGACACAGAAAAGTAGAAAAAAATTGTTTATTTTGTAAGTTGTTGAAATATATTCTTAAGTCATGTTCGAGTTCTTTACTGTAATCTATATAGATAGACTATCTACTTCCGCGCGGTTTCACACGCTCT

>gene10853

TGTGCGATTTCCAAATAGTCGGGCTCAGAACGTGGTTAGACTTTGACAATGTACATAGCAGTGGGGTTAGGGTTGTTATGTGTCGTGCTCGCTGTGTGGTCGACGTGGTTCAGGCAGAAACCTGATGCACCTCCTATGATGCCAGGATTTCTTCCGATAATAGGACATTCACATCATCTTATCGGAGACCGTAAACATCTCTGGAAGTATATACAATACTTAACCTTAACATCACTCAAGTATGGCGGAGTTGTAGAAGTATGGTTTGGCATAAAGAGACATTATGTTCTAACCGATCCAGATGATTGCCTTAAACTGGCCAATACATGTTATAGTAAATCTTACATCTATGACTTTGCAAAGGACTTCCTAAACAATGGATTAGTAACAGCAGACGCGGCCACATGGAAAGTGCATCGCAAATTATTAAATCCTGCTTTTAACCAACAAGTTTTGGATACATTCATTAACGAAATGAATATTCAAGCACGAAATGTTGTCTCAAATTTGAAAAATGAATTAGAAAATGAGTCTTTTGATGTTCTGCGCCACATAGTCAGCTTCACGCTATCAACTGTTAGCCGAACATCTCTTGGGTTAACAGATGAAGACCAAAAGATAATAGACACGGATTATGCTGCAGCTATCGACTCCATATTTGCTCTATACTGCGACAGATTCCAAAAAGTTTGGTTGCATCTCAATTTTATTTTTAAGTGGAGCGAAATAAAACGCAAACAAGATAGACTATTGAAAATTTTGCATGATATTATGATTCCGCTGATATTGAAAAGAAAATCGGAGCTGTCGACTAAAAGAGAACCTACGTATTATGAAGATAGGCCCGGTAAATTTAAACCAATTCTTGATCAGATGCTTCAAATGGCAAGTGATCATGGAGTATTCTCCGATGAAAACATCAGAGAACACTTAGATACTCTCGTAGCAGCATCATATGATACAACTTCTTCAGCGATGACCCACATGTTGCTGGCGATTGCAACTTACCCAGAAATACAGAACAGAATATATAATGAAATACAAGAAGTTCTGCAGAACAAAGATGATGATTTTACTAAACATGATCTACAGAAGCTGGTCTACTTAGAGGCAGTAATGAAAGAGTCGTTGAGATTGTACAGCGCTACTCCACTTATAAGTAGAAAAATTGATGTAGATATAAAACTAAAAAACTGTACAATACGTGCCAACAGCGCTTCTATCATTGGTGTGTATGCTCTCCACCGTCATCCTCTCTGGGGTCCCGATGCAAATGAATTCAAACCAGAGCGCTGGTTGGATCCCAGCATTTCTTCATTACTTGAGAAGCCTGCCCTCTTTGCTGCCTTTGGTATTGGCAAACGAAACTGCATCGGAAAATCGTACAGCATGCTGATGATGAAGATAGTACTAGCACACATTGTTCGACAATATCATATTTCTGGCGACATTCACAAAGTAGTATGTGAATTTGACATAGTTTTGAAACCGGTATCAGGACACCATATTGGTTTGAAATTAAGGTCTTAAGATTGTAAAAATAATTGCTCTGTAATGCCTAATGATGGAGACACAGAAAAGTAGAAAATAATTGTTTATTTTGTAAGTTGTTGAAATATATTCTTAAGTCAAGTTCGAGTTCTTTATTGTAATCTATATAGATAGAAGAAATATAATAAAAGAGACATACATATAGATAATAAAATTGTAGAAAA

>gene10858

CAGTCATGATTTTTGCAGTGCTTATTGTGTTATTCTGTGCGTTGCTCGCGTTATGGAACAAACTGAAACCGCGTGGTCCATCGCCTCCAGTTTATCCTGGAGCATTACCGATATTAGGACACGCTCATCAATTATATGGAGACCCTATACACTTTTGGGTGATTCAGAAGAAAATGTATCAATTTAGCTTAGACAATGGTGGTGTCTCAGAAATACGATTTGGTCCTCATTCTGTTTACATGGTAACTGATCCAGATGACAGCCTAGTCGTAGCCAACACGTGCCTGAACAAACCATACTACTACGAATTTGCCAAGGAAATGTTCAACCGAGGACTCGTCACAGCTAGTTTAACAACATGGAAACCCCATAGACGACTGTTGTACCCAGCTTTTAATATGCAAGTCTTAGCAACATTCGTATCGGAATACAATACTCAGGCCCGGAATCTGGTCAAAGCGTTAACTTCTGAAGTTGGAAAAGAACCGTTCAATGTACGCCATTACTTGGTCAACCAATTGTTGAAAACTGTTTGTCAAACTTCACTAGGATTAGAAACCAAAGACAAATCCATTGATGAAGACTATGCAAATGCTACCGAGGACCTCTTCATGTTATTTGTGGAAAGATTGAAGAAACTATACTTGCATTTACCTTGGATATTCGCTTGGAGTTCTCTAAAGGCGAGACAAGATCAGCTGCTGAAGATCATGAATGATGCAATGTTTAGGGTGATTGATAGACGAAAAGCGGAATTGAAAGGCAATGTGGACAAAAACAAAACAGATATCAGAGATGTTAAATTCAAGCCACTTCTTGATCAACTGCTAGAACTATCTGACACACAAGATGCTCTATCAGATGCTGAGATTAGAGAACATTTGGACACTGTAGTGGCAGCCGCTTATGACACTACATCCATAGCAGTTACATACGCATTGATATTATTAGGAACTTATCCAGAAGTACAAGATCGTGCTTATAAAGAAGTTCAATCAGTACTTGGAGACGAAGATAAAGACTTTTCCAAAGATGATCTGCAGCGGTTAGTGTATTTAGAAGCAGTTTTAAAAGAAAGTATGAGAATGTACCCATCTGTTCCTTGTGTAGCGAGAAGTATAGAAGCCGATGTAAAGTTAAAAAACTACACATTGCCAGCTGGTGCATCTTGTATGATATCAATATATGGACTGAATCATCATGCAATTTGGGGTCCCGATGTGGATCAGTTCAAACCGGAGCGTTGGCTGGACCCATCAACCTTGCCTGACAACCCTAATGTTTTCTGTAGTTTTAGTATAGGTAAACGCAACTGTTTAGGTCGTCTATACGCCATGCTGGTGATGAAGACAATGTTAGCGCACGTACTTCGCAAGTTTCTGATCATCAGTGATATAAACAGCATCACAACACAATATGACATCCTAATAAAACCAGCTGATGATTCACAAATACGTTTGGAATTAAGATCGGGATAGTAATATTTTTATTTAGTATAAAGTCACCATTAAAATTGGGCTACTTTAATTATA

>gene10859

TAAAACCTGCGCAATACAATCAGTATCCCAGTCTCAATCTTCAAAACAAAAAAAAAACAAAAACTTCAAAAAAAGTTGTACCCTCAACTGACGTCACCAGGGTTACTAAACCAGTTATAGTCATAATTTGTTAGCAATCACTTCATACATAAATAGAAGATTTTGTGTTATTCGGGCTCAGATCGTTGTTAGGCTTGCAAAATGTACATAGCTGTGGGGTTGGTGTTATTATGTATCGTGGTGCTCGCTGTGTGGACTACGTGGTTCAGGCAGAAACCTGATGCACCTCCTATGATGCCAGGTTTTCTCCCGATAATAGGACATTCACATCTCATTTTCGGAGACCGTAAACATCTCTGGTACTTCATAAAACATATCAACGAATTATCTTTCGAGCATGGAGGAGCTATCGAGCTTTGGTTGGGCGCCCAGAGGTTTTATGTATTATCCGATCCTGAAGATTGTCTTACACTGGCCAACACGTGCTACAGCAAACCTTACATCTATGACTTTGCTAAGGATTTCTTAAACAATGGACTGATTACAGCTGAAGCCCCTGTTTGGAAATCTCATCGTAAATTATTAAATCCGGCATTCAACCAGCAAGTATTGAACACATTCGTCGATGAAATGAACAAACAAGCTCGAAATCTTGTTTCGAATTTGAGCAATGAATTGGGCAAGACGCCTTTCGACATCAGACATTACTTGGTCAACTTCACACTATCTACTGTTAGCCGTACTTCACTAGGTCTAACAGCCAAAGAACAGAAGATAATAGACCGAGAATACGCTGAGGCTATTGAAGATTTATTGGCTCTGTACTGTGACAGAGCCCAGAAAGTGTGGCTGCATATTGGCTGTGTGTTCAAATGGAGTGAAATGAAACGCAAACAAGATAGGCTGACTAAAACTTTGAAGAATATCATTAATCCCGTTATATTGAAAAGGAAATCCGAAATGAAGAACATACCAGAACCTACGCAATATGAATACACAAATGGTAAATTCAAACCAGTGCTGGATCACATGCTTCAAATGGCACATGAGCAAAATGTTTTCTCAGACGAAGACATCAGAGAGCACCTAGACACTTTAGTAGCAGCATCATATGACACGACCTCTTCAGCCATGACCTTTATGCTGTTGGTGATTGCAACTTACCCAGAAATACAGAGTCGAATTTATAATGAGATACAAGAAGTACTGCAGAACAAAGACGATGACTTTTCAAAACACGATCTGCAGAAACTTGTGTACCTAGATGCTGTTGTTAAAGAATCAATGAGGGTGCACACTGCTGTCCCGTTTATGGCTAGAAAAGTTGACGTGGATGTTAAACTGAAAAACTGTACGCTGCGTGCCAACAGCACTTGTGTTCTCGGTGCACATGCTCTCCACCGTCACCCGCTCTGGGGTCCCGATGCAAATGAATTCAAACCAGAGCGCTGGTTAGACTCCAGCACCTTACCAGATAATCCTGTCCTTTTCGCTGCCTTCGGTATTGGCAAACGAAACTGCATAGGAAAATTGTTCAGCATTCTGATGATGAAGACAGCACTAGCACACATAGTACGACAATATCATATCTCTGGCAGTATTCATAATATAGATTGTGAATTTGACGTAGTTGTAAAACCGGTAAAAGGTCACCTTATTGGTTTGAAATTAAGATCTTAGAATTTTATCAAAATGGCTGTAAATGATAGAGTGAATGTGAAAAATAGGAAATAGTAATTTATTTCATAAGTTGTTTAAATGTATTTTATATGCTATTATACAATCAAAATACTTTTGGTTGCGTAGTTAAAACTAGAATGAAGTAAAATAAAAATAATTATATATATGCCTATTGACTTGGTAACAGTCTTATAAA

>gene10860

GAAAATGTACATAGCAGTGGGTTTAGTGTTGTTATGTGTCGTGCTCGCTGTGTGGTCGACGTGGATAAGGCAGAAACCTGATGCACCTCCTATGATGCCAGGCTTTCTCCCGATAATAGGACATTCACATCTTCTTATCGGAGACCGTAAACATCTCTGGAAGTATATAAAATACTTTACCTTAACATCACTCAAGTATGGCGGAACTTTAGAAATATGGTTTGGCTTAAAAAGACATTATGTTCTAACCGATCCAGATGATTGCCTTAAACTGGCCAATACATGTTATAGTAAATCGTACATCTATGACTTTGCAAAGGACTTCATAAACAATGGATTAATTACAGCAGACGCGGCCACATGGAAAGTGAATCGCAAATTATTAAATCCTGCTTTTAACCAACAAGTTTTGGATACATTCATTAACGAAATGAATATTCAAGCACGAAATGTTGTCTCAAATTTGAAAAATGAATTAGAAAATGAGTCTTTTGATGTTCTGCGCCACATAGTCAGCTTCACGCTATCAACTGTTAGCCGAACATCTCTTGGGTTAACAGATGAGGACCAAAAGATAATAGACACGGATTATGCTGCAGCTATCGACTCCATATTTGCTCTATACTGTGACAGATTCCAAAAAGTTTGGTTGCATCTCAATTTTATTTTTAATTGGAGCGAAATTAAACGTAAACAAGATAGACTATTGAAAATATTGCATAATATTATGATTCCGCTGATAGCGAAAAGAAAATCGGAGCTGTCGACTAAAAGAGAACCTACGTATTATGAAGATAGACCCGGTAAATTTAAACCAATTCTTGACCAGATGCTTCAAATGGCAAGTGATCATGGAGTATTCTCAGATGAAGACATCAGAGAACACTTAGATACTCTCGTAGCAGCATCGTATGATACGACTTCTTCAGCGATGACTCACATGTTGCTGGCGATTGCAACTTACCCAGAAATACAGAATAAAATATATAATGAAATACAAGAAGTTCTGCAGAACAAAGATGATGATTTTACTAAACATGATCTACAGAAATTGGTCTACTTAGAGGCAGTAATGAAAGAGTCGTTGAGATTGCACAGCGCTACTCCACTTATAGGGAGAAATATTGATGTGGATGTTAAATTAAAAAACTGTACAATACGTGCCAATAGCACTTCTATCATTGGTGTGTATGCTCTCCACCGTCATCCTCTCTGGGGTCCCGATGCAAATGAATTCAAACCAGAGCGCTGGCTGGATCCTAGCATTTCTTCATTACTTGAGAAGCCTGCCCTCTTTGCTGCCTTTGGTATTGGCAAACGAAACTGCATCGGAAAAACGTACAGCATGCTGATGATGAAGATAGTACTAGCACACATTGTTCGACAATATCATATTTCTGGCGACATTCACAAAGTAGTATGTGAATTTGACATAGTTTTGAAACCGGTATCCGGACACCATATTGGTTTGAAATTGAGATCTTAAGATTGTAAAAATAATTGCTTTAAATCCTAATGATGGAGATAAAGAAAAGTAGAAAGTAGTTGTTTATTTTGTAAGTTGTTGAAATACATTCTTAAGCCAAGTTCGAGTTCTTTATTGTAATCTTTATAGATAGTTATAAAGAGACAAATAATAATAATATAATCGAAGAAAGGTCATAACTGTTCATTGAA

>gene10862

TGTATGTATTAGTGTTACTCGTGCTACTTAGCGTTGTGTACCATGCATATTGCTGGTGGAAGCCTCGTTCACCTCCAATATTTCCTGGCCAGTTGCCGTTCATTGGACACGGTTGGATATTTCTTAAATACCGCAATGATATGTGGAGTTATTTGAAAAGTATCGGTGAATACGTATTGGAACATGGAGGAATAATTCAATTTCGGGCGGGTCCTCACATAGTTTATGCTGTGAACGACCCAGAAGTCGCTGGCATCATAGCAAACACGTGTTTGGATAAACCATATTATTACGAGTTTATGAGCGATGGAATAGGAAATGGATTGGTAACGTTAAATGGGGATATGTGGAAGATACACCATAAATTATTAAACCCAGCGTTCAGTCAGAAAGTCCTGAATACGTATTTAAATGAAATGGACGTGCAAGGTCAAAATCTAGTCTCGCAATTGGCAACAGTGGCGGAGAAGGGACCAGTTAATATCACTGACTTCTTATTTAAACATATCCTAAGAACAGTTTCCCGTACGTCACTGCGGCTGGAAGCTAAAGATCAGGATATGATTGACAATGACTTCGGTGAAGCGGTTGAAGAAATAGGAAACATTATAATTCATCGCGCTTTAAGACCACTGTTACATCTTTCATTTTTATTCAATAGGACCGCAATGAAGAGAAGGCAAGTGGAACTAAGTAGAGACAACAGGAAACTGTTAGATCATATAATTCAGAAAAGAAAATCTGACTTAAAAGCAACTAAACATATAGTCAATAATAAAGACGATTTTGTACCAGGTAAATTCAAACCTATACTAGATCTGTTGCTGCATTTATCTGATGAACAATATGTCCTCTCCGATGATGAAATCAGGGCGCACCTGAATACTTTCGTGGCAGCTTCATTCGACACAACTTCATCAGCTCTTCAGACTGTGTTGTTAGTGCTTGGATCATATCCTGATGTACAGGAACGAGTTTTTAATGAGATTCAAGATGCTTTTGACAATAGAGAAGAGCTGACTAAATATGACCTGACTAAACTCGTTTACTTGGAAGCAGTGATAAAGGAGGTATTAAGGGTATATAATATAATTCCTTTAATAGCGCGAAAACTTGACACTGATATTGTACTCCCAAAATATACTCTACGAGCTGGAAGTTCCTGTATCCTGTCGTTATACGGCCTTCACCGTCACTCTTTGTGGGGACCAGACGCGAGGGAGTTCAAACCGGAGCGATGGTTGAATCCCGACACTTTACCTACTAACCCCAACTTATACGCTCCGTTTGGTATTGGCAAGCGAATTTGTATTGGAAGACAGTACGCCATGATGTCCATGAAGACGTCGCTTATCCATATCGTACGAAAGTTCCACATTTCTGGTGATATCAGTTCATTGAAGTGGAATTTTGAAGTCGTACTAAAAGCAAAAACTCCTCTTATTACTTTGACGTTAAGGTCTTAAATTAATAAAAGGCT

>gene10863

TCTCAAAATGTACGTAGCATTGGGATTAGTGTTGTTATGTTTCGTGGTGCTCGCTGTGTGGACGACGTGGTTCAGACAGAGACTTGATGCACCTCCTATGATGCCAGGCTTGCTCCCGATTATAGGACATGCACATGCCCTTATCGGAGACCGCAAACATTATTGGAAATTTATTCAGAATATCAACGCAACATCATTTGAACGTGGTGGAGCCATTGAATTCTGGATGGTCTATATGAGATATTATATTTTAACCGATCCAGATGATTGTCTTACACTATCTAACACATGCTTTAATAAACCTTACGTCTACGACTTTGCCAAGGACTTCTTAAATAACGGATTTGTGACAGCGGATGCTTCTATATGGAAAGCTCATCGCAAATTATTAAACCCTTCGTTCAACCAGCAAGTCATATACACATTCATTGATGAAATAAACTTGCAAGCTCGAAATCTTGTCTTGAGTTTGAAAAATGAACTGGGAAGAAAGCCTTTCGACGTCCGGCCCTACTTTATCAGCTTCACAGTTTCTACTGCTATTCGATCTAGTCTTGGGCTAGAAGCCGAAGACCAAAAGAATGTAGACCGAAAGTATTGCGAGACTATAGACGATTTGTTTGCTCTGTACTGTGACAGAACCCAGAAAGTGTGGTTGCATATCGATTGGGTGTTTAACTTAAGCGAAATGAAACGCAAACAAGACAAACTTACAAACTCCTTGAAAAATATAATTTGTCCTATTATACAGAAAAGGAAAGCCGAGATAATGACTAAAATAGAAACATCGTCGTTTGAGAATAAATCCGGTAAATTTGAACCAATTCTCGATCAGATGCTTCAAATGGCAAACGAGCAAAATGGGTTCACAGATGAAATTATCAGAGAACACTTAGATACTTTTATAGCAGCATCATATGACACGACGTCTTCATCTATGGTCTTCACGATGTTGGCAATTGCGTCATATCCAGAAATACAGAATAGAATTTATAACGAAATACAAGAAGTTTTGCCGAACAAAGACGATGACTTTTCAAAACAAGACCTACAAAAACTTGTGTACTTAGAAGCAGTTATAAAAGAATCATTAAGATTGTACAGTATAATTCCAGTTGTAGCGAGAAAAGTAGAAGTAGATGTTAAACTGAAAAACTATACGCTGCGTGCCAACAGCACTTGTATCATTGGTTTGTATGCTCTCCATCGTCATCCTCTCTGGGGTCCCGACGTAAATGAGTTCAAACCAGAGCGCTGGTTGGACCCCAGCAGATTACCTACAAATCCTAACCTCTTCGCTCCGTTTAGCATCGGAAAACGAAATTGCATAGGCAAAACATTCGGCATGCTGATGATGAAGATAGCACTAGCACACATAGTACGACAATATCACATTTCTGGGAGCATCCATAATATAGAATGTGAATTTGATATAATATTGAAACCCGTATCAGGTCATCAGATTGGATTGAAACTAAGATCGTGAAGATAAACAACAACGGAGTATCGTATAATTTTAACGACAATAATGCCCCAAAATATTTTTATTTAATTCTTAAATAATAATTGGATGACCCAGCTTTACTCGGAATTTCTTTAGAAAAAAAAATAGAACAAAACATAAACGTTTGGACCAAGTATTGAACCCGAGTGTTCTACAGTGGGAGGCATTTCCCTGCTAAGCTATGGCGCTATGACGAAATTTAGCATGGAAAATAATAATCTTCCTAAAAAAAAAATCTCCAGCGATCTGTGGCCGACTATAAAAATGGATCTTCGAATAACAAAACTAAACTTCTGAATAGAATATTGAACTTCCGACAAAATTACTTATGCCCGAATATCGAAACTATACTCAATTTTATTTTTAAAACGCCAGAGATGGCACGATATTTAAACGCAACTTAAAATTGAGTACCCGTTCAG

>gene10962

CGATGTAATAGGGATATCAAACTATTTGGAAAACTTACAGTCGCTGACGAAACACAGGACGTAAGCAACTAGAAAAAGCTGAAGCATCGAGAATGTTCGCGGTGGCGTTAGTGTGCATCGCGTGCGCAGCGTACTGGGCGTGGTTACGCATGCGTAATGCCACCGAGCCACCCTTCTTCCCAGGAGGATGGCCCATCGTGGGACATGCACCACAACTACTTGGAGATAGTTGTAAACTATGGGACGGCATCAATGACCTAGCGAGACAGAGCTACGACATTGGAGGAGTGGTGTCGGCCAGCATTGGACCACGCACCGTGTACATTGTAACCGACCCAGACGACAGTTTCACTATAGCGAACACGTGCTTGGAGAAGGACGGGTTCTACGACTTCGCTAAACCGTGGCTAGGCGAGGGTCTAGTCACTGGGAAATATTCAATATGGAAGAATCATCGAAAATTCTTAAATCCAGCGTTCAGTCAGATACTGCTGGACACATTCATGGGAGTCTTCAACAGTCAGTCCAGGAAGCTGGTGAAGGAACTGGAGAAGGAGGCTGGGAAGGGTCTCTTTGATCACTGGACTTATACACGACATAATGCCTTGGAGACTATTTGCTTGACAGCGTTAGGAGTGGACTTCACAGACTCGAACCTGAACAGCCAGTACGTGGAGGCGACGGAGGTGATCCTGAACACCATCGTAGAGCGGTTTATGAAGTTCTGGTGGCACAGTCCTTACACCTTCGCATGGAGTAACGTGAAGAAGAAGCAGGACGAGTGTTTGAAGATATTGCACAACATGTCTAACATGGTGCTGCAAAGAAGGAAATTCGAGTTCAATGGAAATGTTTGCGCTGAGAGAAGTATTATACCTGGAACCAGGTTCAAAGCTTTTATGGATCTCCTTTTAGAGCTGTCTGTTGAGAAGGGAGTATTCAACGACAGAGAGATCCGAGAGCACGTGGACACTATGATAGTTGGCGGCCATGACACCTCCGCTAACGTGCTCATGTTCACCATGATACTCCTTGGGTCACATCCTGAGGTTCAAGAGAAGGCTTACGCTGAAGTACAAGAAGTTCTAGGAGGTGACAGAGACGTGGAGAAGACAGATCTGTCTCAGATGGTGTACTTGGAAGCTGTGCTGAAGGAAAGCATGAGAATATTCACCATCGTACCAGTGCTTGCTAGGAAACTCGACAAGGATGTTAAACTGAAAAACTACACCCTCTCTGCTGGTCGAACCTGCTTCCTATTCGTGTTTGGACTGCACAAGCATCCGATCTGGGGCCCAGACGTGAACGAGTTCATACCGGAACGCTGGTTGGAGCCGGGCAGACTGCCTGACAATCCTAATGCGTTTGCTGCTTTCAGTGTCGGGAGACGACAGTGTATAGGGAAGGCGTACGCGCTAATGTCTATGAAGACGACCTTGGCTCACTTGGTCCGCAGTTACAAGATCAAGGCAGACCACACGAAGGTGAGGCTCAAGCTGGACGTCATGCTGAAACCAGATTCAGGTCACTGTGTTAGCATCGAGAAGAGGACGTAGCGCGTAGTGTACCTACAGTCAGCTAAGCTATTAATTGAGTACACAGAATAAAATATAGGTTCGTTACAAAGCATGCAATTTAGTGTCATAGTAGCTACCGTCCTTGTACTGAAGCAATCTTTTGTCTTACCTAGAAATTTGACTGCATGTAAATGAAAGAAAAAGATTCAGTCACTATTAGGATCACATCCATCGCCATACTGCGACATTTAACACATTACGGTATCAAAGTATCATTCGCTAGTTAGTACGCGATTATATACACTGCTGATGTGCATGCGTATCGTTTAGATTTAGGTAAATGGATGCTATTCTTAATCAAAAATAAATTATTTCGACTGTTCAATATAATAAAATATTTCAA

>gene10964

ATGTGGTTGGCGTATGTGCTCCTGTGTCGGTTGTACTTCCCAGTGAACGAGCAAGTGGCGACGTGCCCAGGGAAATGGCCGCTCATTGGTCACGCTCACAAACTCGTAAACAAGGAAGTTGTGACGGAGCCAGAAGATGTGTCCACAATTGTCAACAAATGTCTGGACAAGATGTTCGTGTACAAGTTTGTCGAACCAATGTTAGGGAAGACGTTGATTGCAGCTGAAGTACCGACATGGAAACGAAACAGAAAATTTGTAGACCTTTGCTTCAAGCAGCAGTTATTGGACGACTACCTCCAACTGTTCAATGAACGCGCTGAGCGGTTTGTCGAGACACTTGCCGAAGACGCTGGTCAAGGTGACGTCGACCTTAAGAAGAAAATCACAAGAAGTATTTTAGAAACTTCTGTCATGACCACTTTCGGGATTAATCTGGATGGAAAAAACGAAATGAACGATAACTACGTCAAAGCATTAAACGACTGTCTGAGCATTATCTCTGACCGGATCTACAAGCCTTGGCTCATCATAGATACTATCTTCAACTTATCCAGTGACAGAAAAAAATTAGAAAAGGCACTTGAAACTGTATTTCAGTTTTCGAAGGAAACAGTGCTGTTAAGAAAGACTGAACACTTACAACGCTTGCAAAAGAAAGAGGACCTTAACCACAAAGGTGGGTTTCAAAGCGTCTTAGATGTGATTTTGGAAAACTCAATGACTGAAACTGAGAGTGTATTCAACGATGTTGAGTTGAGGAATACAATGGACAACATGATAATCGCTGCCTTCGATACCACTATCTATCAGATGCTCTACGTTTTGATCTGCATCGGTAGCAGCCCTGAAGTACAGGACAAGATTCTTGAAGAAATAAACTCAGTTTTGGGTAAAGATAAACAACTTAAAAGCAACAATCTATCTCAACTCGTATATTTAGATGCAGTTGTTAAAGAAGCTATCAGATTATATCCAGTTGGTCCACTGATTGGACGACACACAACAGTACATACGAAGTTGCGTAATGTAACAATACCAGCAGAAAGTCCCGTTATTGTGCATATATGGGCCATAAATAGAAGCAAAAGATACTGGGGGTCGGATGCGGAGGAATTTAAACCAGAAAGATGGTTAGATTCCAAGACTATGCCAAAGCATCAAGCTGCATTTGCCACCTTCGGGCCGGGAAGGAGGGGTTGTGTTGGTAAAACATACGCATTGATGTATATAAAAGCAACCGTTGTATCATTATTGCGTAAGTACAAGATTACAGCTGATCATAAGAAAATGAAATTGGAATGCAAAGTGATGCTCAAACCTTTATCAGGACACTTGATCAAAATAGAGAAAAGGGATAAATAG

>gene10969

ATGTATAACAAGACCGATGATTGTGTCCGTACAATGTTGAGCTTACTGCTATTCGTTTGTGTGGTGTGCATTGCCAGTGTTTGGTCCAAGTATGCTCGGGGTAGGCCCAAGTATCCCCTGCCTCCTGCTATGCCTGGAGCCCTGCCAATCGTTGGAGTTCTTCATCAGGTGCTAAAGAACTATTCTCGACGTTGGGATTTCCTCAAGAGCAAAGCTGAGGAGTGCGCAAAGTTAGGCGGAGTAACTTACGCTCATTTCGGTAGCGAGTTATATTATGTGATAACAGATCCTCAAGACGCGTTGACAGCCAGCAACCAGTGCCTGAAGAAACATTACGCATTTGATTTGGCTAAAGTCTGGATGGGAAATGGGCTGGTACTGTCATCAGGAGACATTTGGAAGCGTCATCGCAAGCTACTGAGTCCGGCGTTCACTTTGCCGATAATCCATAGTTTTTTAGATGTATTCAACAGTCAAGCCAAGAAACTCGCCAGTTCCATGGAACCGCACGTCGGGAAAGGCCTTTTTGATCCATTTCTAAATTTAAAATTGAATGCATTGGAAACTTTCTGTGTTGGTACATTAGGTATAGAAGCTCTAGATGACGTGAACTTCACTGAGAGGTATATGGAGTCAGTGGATGACATTGTGACGTTGTGGATCAGTAGAGTCGTGAAGGTTTGGCTGCTCAGTGACGTCGTGTGGAAGCTGACTGGACTGAAGAAGAAGGAACAGGAACTTGTGGATACTTTACATACTATGACTAATAAAGTTCTGCAACGAAAAAAAGCTGCTCTCAAAAACAAGGGCGTGGAGAAAGTTATTGAATCTCAAACTTCAGGTATTAAATACAGACCATTTCTAGACCTTCTTCTGGAACTGTCTACCAACGGTGCGTTCACTGATGAAGAGATCAGGGAAGAGACGGACACTATCATAGCAGCTGGATATGACACCACTTCCAATCAGATTACTTTTGTACTGTTGCTCCTTGGTGCTTACCCTGATGAACAAGAAAAGGTTTATGAAGAACTTTTACGAGTTCTCGGACCAGACAGAGACATAGAAAAAGATGATATAAATAAATTAGTTTATACAAACGCTGTTATAATGGAGTCTCTCAGGTTGTTTCCGTCGATACCATCTTTGTTTAGAACTGTGGAAACCGACGTTAAACTGAAAAATTACACAATGCCCGCTGGCAGTTACTGTGTTATCTTCCCAATAGCTGCTTCTAGTGTTGATCCTTCTTGGGGACCAGAACCTGATAAATTCAGACCAGAAAGATGGCTACATGGCGATTTTAGGCACAATAAGGAGTATTCTGCATTTGGCTTAGGAAAGAGAGCTTGTATAGGTCGAACGTACGCCATGATATCAATGAAGGTGACGTTAGCACATTTCTTGAGACAGTACCGAGTGAAGGCAGACCTGAGCAAATTGAAGCTGAACTTTGACTTCTTGTTGAAACCTATTTCAGGACACGATATCAGTATTGAAAGTAGAAGAAAATAA

>gene10970

CATGACAATTGTATTATTAACAACTGATAAAAACATGTCTTCAGTGATAATATTTTATAGTAAAATTGAAATACATCTAACTGACGATAGTACTAAAGCTTGAGCATCTACATATAAAAAATTTCAACATATTTCCATAATTTCAAGGTTTTTTGAAAGAATAAAATATCGTGAAGTGTATAGAAGTTGTAGTAGTATTTGAAATGCTTAGTGTTGTGCTTCTGTTTGTGGTGTGGTGCGCATGCGTGGTGCTGGTGAGGCGGCTGCGGCCAGCTCATCCGCTACCACCATCTTACCCTGGAGAGCTGCCGATCTTGGGACATACACATTTGATTAGCGGTGATACTGAAAAAATATGGGAGATTTTGAAAGAAATGTCAGAGAAATGTGTGGAAAATGGTGGAGTCTTACACGCAAAAATTGGAACACAAATATATTACTTGGTAACGGATCCTGATGATGCGTTGACGGTGGCCAACAACTGCCTCCACAAACATTTCATTTACGATTACGTGAGGACCTGGCTTGGCAATGGATTGCTTACATCTTCAGGAGAATTCTGGCAACGGCACCGCAAGCTCCTTAACCCATCGTTCACAGTGCCGGTGATCCACAACTTCCTCGGCATCTTCAACCACATGGCGAAGCAGTTAGTCAGCGAGTTAGAGGTCCACGCAGACCGGGGACCCTTCGAGATCTCCCCCTATTTGAGGGTCATATCGTTCCAAACTTTCAGCCGAACCGCGTTCGGCATAGCAGATGACAGCGTGAAAGAATTTGCAGAGAAGTATATGGAATCATCAGACCAAGTGATGAACATGGTGGTGTACAGGTTCCAGAACTTCTTGTTGCATAGTGACCTGATCTTTAAGCTGACTGGTTTGAAAAAGAAGCAGGACCGTCTGATTAAGATACTGGATGATATGGCAAATCAGGTAATAGAGAGGAAAAAGGCTGAAATGAAATTGGCCAATGAGAAGAAAGCTAATGAATGGTCAACGACAAATACTAGATACAAGCCATTCATGAGCCTGTTATTGGAACTGAAGAATGATGATACTCTTACCGATAAGGAGATAAAAGAAGAAGTGGATACATTTATAGTAGCAGGTTTTGACACCACGTCCAATTGTCTCACTTGTCTACTAGTCCTGCTTGGAACATACCCTGAAGTGCAAGAAAAGATGTATAATGAAATAATAGAAGTTCTAGGTCCAGACAGGGATATTGAGAAGGATGATATCAAACGACTGGTGTACACTGAGGCTGTTATTAAAGAGGCTCTTAGGGTGTTCCCTATTGGACCTGCCCTACTAAGATATGTGGATAGGGATATTAAACTTAAAAACTACACAATGAGAGCAGACAGTCAGGCAGTGATCCTCGCTCGAGGGGCCCACAGGAGTCCTATATTTGGTGACGATGTGGAGACATTCAGACCTGAACGATGGCTGGACTCCAGCATAACGGCTGTGGGGAACAATGCTTTCTATGGATTTAGTTTAGGAAAACGATCGTGTATTGGAAAAGCTTACGCAACGGTTTCAATGAAGACGACTCTCTCTCATCTCTTACGGAAATATAAATTAATATCTAATGTAAATGACATGAGGTTCAAAGTCGATATGGTACTAAAAACGACCACAGCGCCGGTACGCATAGTGAGAAGAAAATGAAACATTATTATTTGATAGAAAGAAAAGCGTGGTCGAGTTTTAGAATAAAAAATAATAATAAAAAATATTGATAAATGTTTAATAAAATAAATTTTAA

>gene11878

ATTCGTGTAGTCGCTCTAGTGAATGAAGAACTAATTTATCATTCAATTACATTACAAACTAAAACAAATTGCGCATATTCGAAATATTTCGATCAAAAATCTTCGATTACGTATTATAGAAATTGATGCAATTAATATTTTCGTTGTTCAACACGTACGTAATACGTTTATCAGAGTGTAATCACTGTTGTGGGTGCCATGTGATATCTTCGCCTTTAAAAGTAGATATATTCATTGGACGATAACATCGCTGAGGTATACTGTGAACACCACTCAGTGACTAACTATACAAACATGCAGACGACTAAAGTGAAGCCGTTCGCGTTGCTCAAACTAGTATCTATCAACAAGAAAATTACGAGGAGAGTAGCTGTAGCAAGTACTACAGTCAATTCGGACACAAATTTGAAATCATGGAAGGAAATTCCTGGACCTCCATCCTTGCCAATTATTGGCCAATTACACAATTTCTTTCCTGGAGGTCAACTACATAACATCGAAATAGTGGACACTACTTTGAAATTATTCCAGTTGTATGGTCCAATTGTAAGACTTGATGGACTATTAGGAAAGGCACCCATGATAGTACTGTTTGACCCAGAAGCTTCTGCCCATGTATTACGAAGTGAAAACTGGCTGCCGATTCGACCTGGATTCGTATCACTTGAATATTATAGAAAAGTATTCAAGAAGAAGTTTAATAAGGATCACAATGTTCAGACTACTGGTCTTATCACTGATCACGGTGAAGTATGGAAAGAGTTTAGGTCCACTGTCAATCCAGTGATGTTGCAACCGAAAACAATCAAACAATATACCACAGTACTTGATGAGGTGGCGCAAGATATGGTCGCAAGGATGAAAGCCAATCGCAATGAAAAGAATATGATTAAAAATGATTTTGATAAAGAAATGAACTTATGGGCCTTAGAATCTATTGGTACGGTGGCTCTTGGCTCCCGACTGAACTGCTTCGATCCGAACCTTCCTGCAGACTCTCCGGAGTGGCAACTGATCCAATGCGTCCACGATCTGTTTGTTACTGCCAATAAACTGGACTTCAAACCAAGTTTATGGAGATATTTTGCTACACCGACATACAAAAATGCCATGAAATTGTATGAGCATCATGAAAACCTAACAAAACACTTCTTGAAGAAAGGAAAGGAGCAACTAAAGAACAATTCTGGTGGAGAGAATGGTGTACTGGCAAAATTATTGGCGATCAATGAAGAAGTAGCTTACATAATGGCCAGTGACATGTTATTTGCTGGTGTAGACACGGCTGCAAATACAGTAACCGCAACACTTTATCTTCTGGCAACAAATCCAGAAAAGCAAGATAAGTTAAGAGAAGAATTACGGTCGGGCTCTGATCGTAGATACCTGAGGGCATGCGTCAAAGAATCATTACGAGTGATGCCTGTAGTATCAGGCAATATACGAAGAACCACCAAGGAATACAGTTTGATGGGTTACAAGATACCTAAAGATATGGAGGTGGTGTTCGCTCACCGAGACATGTCGCTGTTAGAGGAATACTTCCCAAGGGCCAAAGAGTACATTCCAGAGAGATGGATAACCAGTAAAGATGATCCCCTTCACTATGGAAACACACATCCATTCACGCATCAACCGTTTGGATTCGGCGCCCGAAGCTGTATAGGTCGTCGCATAGCGGAATTGGAGATGGACATCTTCGTAGCTCGCTTGATCGAAAACTTCCAAGTAGAATGGTTCGGTCCACCACCGAAGATACAACAAGAATCTCTCAATTACATCAAGGGACCTTTCAACTTCATATTTAACGATGTTAAGAAATAAAATAATTATAATTTTAATGTTAATGAAATGTATATACCTACTGTTATAATAATGTATGAAATTATAGATAAGTAATGAATATGTTGTGGCGTTCTCAATATGTAATTTACATTTATATATAGTTATTGTGTGACTGCCTCGTTGGCCAAATGGTC

>gene11879

ACCGAAAAACAGAATGGGTAACGTTACAATAACAGAACTATATTAGAAGCCGCTGGTCTATGTCGCTTTAAAAAATAAAAAATAAACATTCCAATTTCGAATTGGGTATTTATCGAATGACAAAACAAAAAAAATACGCGCCATTTTAAAAACAAAAATATTCACGCGTTCGAAATTCGAAACAAATATTTAGATTCACGTGTACCTATTATTATTAGCCAGAGATATAGATAATAAAATTGTGAGTGTACCAAAGTGCCTATTTAGGTACAAGTGACATGATAACGAATTTTAAATAAATAATAATACTAGTCTGTGATTGTGTTAATTCTCTGATCAACAACATAAACATGCAGACGACGAAGTTGAGACCGCTCTTCCAGTTGATATCTATTAACAAGGAAGTCATAAGGACTGTAGCAGTCGCCAGTACAGCGGTGAAGACAGAAGCCTTGAAACCATGGAGCGAGATCCCAGGACCACCATCATTGCCCATCATTGGGCAAATACATCATTTCCTTCCCGGAGGCACTTTCGGTGACATGAAAGACCCGGAAACTATAATGAGACTGCTCGACACTTATGGACCAGCTGTAAGGCTGGACAGCATGCTGGGCAGGCCACCTCTGCTCTTCTTAAGCGATCCTGATAGCGCGGAACTTGTTTTACGGAGTGAAAACTGGTTGCCGTACCGTCCTGTTTTTCAGTCACTTGAGTTTTATAGAACCAATTACAAAAAAGATAAAGGACGCGTAACAGGACTTATTTCGGATCATGGCGAAGTATGGAAAGAGTTCCGATCAACCGTCAATCCGGTGATGTTGCAGCCTAAGACGATCAAACTATATGCCAAAGTACTGGATGAAGTGGCTCAGGATATGATAGTTAGGATGAAGGCCAACCGTGATGAAAAGAACATGATAACAAAAGATTTCGATAAAGAAATGAACCTATGGGCTTTAGAATCTATTGGTACGGTGGCTCTTGGCTGCCGACTAAACTCCTTCGATCCAAACCTTCCTGCAGACTCTCCGGAATGGCAACTGATTCAATGCGTCCACGACCTCTTTTCTACAGCCAATGAGCTTGATTTTAAGCCAAGTATCTGGAGATATTACGCAACACCGACTTTCAAACAAGCTATGAAGCTGTATGAGCATCATGAGAACTTAACTAAATACTTCATAAACAAAGGAAAAGAACAATTGAAGACCAAACCTGATAATGAGAAGGGAGTATTAGAAAAACTGTTGGAAATAAATGAAGAAGTAGCTCATATTATGGCCAGCGACATGTTATTTGCGGGTGTTGATACGGCTGCAAACACAGTAACAGCAACACTATACCTTCTGGCCAAGAACCCAGACAAACAAGCAAAATTAAGAGAAGAAGTAACCTCCAATGTGGAGAAGAGACCGTACCTTAGGGCCTGCATCAAGGAGTCTCTAAGACTTTTGCCTGTGGTATCAGCAAACGCTAGGAGAACCACAAAGGAGTACAATATACTGGGATACCAACTTCCTAAAGGCGTAGACGTCATCTTCACTCATCGAGAGATGTCCTTAATGGAAAAATACTACCCAAAAGCCAAGGAGTATATTCCAGAAAGGTGGCTGACCAACAAGGATGATCCATTGCACTATGGAAATGCCCATCAATTCGCCTACGGGCCCTTTGGTTTTGGCGCACGAAGCTGTATAGGTCGTCGCATAGCAGAACTAGAAATGGACACATTTGTGGCGCGATTAATAGAGAACTTCCAAGTCGAATGGTTCGGACCCCCACCGAAAGTCGTACAAGAAGCTCTCAACTACATCAAGGGTCCCTTCAACTTTGTATTTAAAGACATTAAATGAATAAACTAATTTATTTGTTTGGTTAAGTTTATTATAGGAACGATATTGATATTTTTATGTTTTACAAATGTATT

>gene11881

CTGCAATGTCTTGTTGACTCGCATTTTGATTACAAACCTCATCGAATGAGGTTGAATAGCACGTGCTTGTGTTGTGTTAAAAATGATTTTGTTATTAGTATCTGTTGTTTTAGTGTTTGCTTTGTTAGTGTCTTGGATTAGTTTAGTTAGACAAAGTCGCCGGTTTAATGTAGACGGGCCATCTCCACTGCCCTTAGTGGGAAATGCACATTTGTTTGTAGTTAAATCTTCTGAGTTCTTGAACTTGGTTCAAAGACTATCCGAAAAGTATGGCAAAGTGTTCCGGGTACATTTTTTCTCGACGCCTTACGTGGTGATCTGCCATGCCAAGCAGGCTGAGGAATTAGTCTCGAGCATAGAGCATATAACAAAAGGCAGGTCCTACAGCTTCCTGACATGCTGGCTCGGTCAAGGCTTACTCACTTCCACAGGTCAAAGATGGAAGACCCATCGCAAGTTCCTGACTCCCGCATTCCACTTCAATATCCTTCAGAACTTCCTCCCCGTGTTCTGCAAGAACCAGCGCGTGCTGACGGAGAAATTGCGAGGCCTTGCCGATGGCCGCCCGATTGATATGTTCCCTATTATTGCTTTGGCTGCCCTTGATAATGTCACAGAATCCATTATGGGCGTCTGCATGGAAACCCAAAAGGATAGCCAGTCTGAATACGTGAAATCCATTGAAGAATTGTCAGCAATAGTAACAATGAGGATGCAAATACCATTCTTCGGTGAAGACGCCATATTTAATCTGCTGCCGTACAAGACGAGACAAGACAAAGCATTGAAAGTGTTACATGGACAGACAAACAAAGTAATTGAAGCAAGGAGACAGGAACTGAAGAAGGCAAAAATAACTGCTCTTCCTGATAATAATGATATTGGCATAAAGAACAAGCATGCGTTCTTGGACTTGTTGCTGCTCGCTGAAATTGATGGCAAAAAGATTGATGACGAGAGCGTGAGAGAAGAGGTCGACACATTCATGTTTGAGGGCCATGACACAACAACTTCGGGCATTGTATACACACTTCATTGTCTCTCTAAACGCAGAGATGTCCAAGAGAAAATCTTTGAGGAACTGAAGACTATCTATGGAAATGAAATGCACAGGGACCCGACTTACCATGAGCTTGCACAAATGAAATACTTGGAGCTGGTCCTCAAGGAGTCGATGCGGCTGTTCCCGCCTGTGCCTCTGATTGAGAGAAGAATCATGAAGGATTGTGAGGTGGGAGGTTTGAAACTATTGAAAGGTACTTCAGTCGTGATGAATATCTACCAGATCCAGCGGCAGCCAGATTTGTATGATGATCCTTTGGAGTTCCGTCCTGAAAGATTCGAGGCTCCTCTGAAAAACCCCTTCAGCTGGCTGGCGTTTAGTGCTGGTCCTAGGAATTGTATTGGTCAAAAGTTCGCGATGATGGAACTGAAGATCACTATCTCGGAGATTATCAAGCACTTCTACATATTACCAGCAGCCCAGGAACCAGAGTTGAGTGCTGATTTAGTTCTGAGGTCCAAAAATGGAGTCCAAGTAAAGTTTATGCCGAGAAAATAATGTGATATTATTTGTTGTGAATAGTACTTAGGTTAAGTTTAGATCTATATTTTTGTACTTATATGTGTGTTGAGTCAATATAGTACAATGTACGTTATTTTGTAATATGGGAAGGAATAAATGAATTTTAAGGCAAA

>gene11882

GTTGTCCTGTTGACTCGCATTTTGATTACAAGCCTCATCGAATGAGGTTGCATACTGGTGTTAGTGTACTTTAAAAATGATTTTGTTTTTAGTATCCGTTATTTTAGTGTTAGGTTTATTAGTGTCATGGATTAGTTTAGTTAGAGAGAGTCGCCGGTTCAATGTCAATGGGCCGATGCCCTTACCTATTATCGGCAATGCTCACATGTTCATTTCTGAATCTACAGAGTTCTTGAACTTACTTGCAAAGTATTCTGAACAATATGGGAAGGCGTATAGAGTACATTTTTTGGCTGTGCCCTATGTGATCATTTGCGACGCTAAATATGCTCAGGAAATAGCATCGAGTCAGGAAATTATTTTCAAAGGGGGACCTTATAGCCTGATGACATGCTGGCTTGGCCAGGGTCTGCTCACTTCTGCTGGTCAAAGATGGAAGCGTCACCGCAAGTTCCTGACTCCCGCATTCCACTTCAACATCCTGCAGAACTTCCTTCCTGTGTTTTGCAAGAACCAGCGCGTGCTGACGGAGAAGTTGCGAGGCCTTGCCGACGGGCGCGCGATTGATATGTTCCCTATTGTCGCTTTGGCTGCTCTTGATAATGTTACAGAATCCATTATGGGCACCAGTGTCGACGCTCAAGGACATGAAAGCGAGTCTGCCTATGTGAAGTCAATTGAAGAAATGTCAGCCATATTAGCGATGAGGATTCAAATACCAATTCTTGGTCCTGACGCCATTTTTAATCTAACACCGTTAAAGAGCAGGCAAAGCAAAGCGTTGAAGGTATTACACGGACACAGTACGAAAGTAATTGAGGCGAGAAGACAAGAGTTGAAGAAGGCTAACATCACTACTCTTAACAATAGCAACGATTCCGGAATTAGGAACAAGCATGCGTTCCTGGACTTGTTGCTGCTTGGTGAAATTGATGGCCAAAAAATTGACGATGAGAGCGTGAGAGAAGAGGTCGACACATTCATGTTTGAGGGTCACGATACAACTACTTCGGGTATTTCATACACACTCTACTGTCTCTCTAAGCGCAGAGATGTGCAGGAGAAGGTATATGAAGAGCTGAAGACTATTTTTGGGGATGACATGGAAAGAGACCCGACTTACCAAGAACTTGGACAGATGAAATACTTGGAGCTGGTTCTTAAAGAGTCGATGCGCTTATTTCCACCTGTGCCTCTAATTGAACGAAGAATTACAAAGGACTGCGAGGTCGGAGGTCTCAAATTAGTTAAAGGCACATCTGTCGTCCTAAATATCTACCAGATTCAACGTCAACCAGATATGTTTGAAGATCCTTTGGAGTTCCGTCCCGAAAGATTCGAGGAGCCTCTAAAGAACCCCTTCAGTTTTCTGGCATTCAGCGCTGGTCCTAGGAATTGTATTGGTCAAAAGTTCGCGATGATGGAACTGAAGATCACTATCTCAGAGATTATCAAGCACTTCTACATATTGCCTGTTGATGAAACACCTCAGTTAAGTGCTGATCTTATTTTGAGATCGAAGAATGGAATCAAAGTAAAGTTTATGCCAAGAAAATGAATGGATGATGGATTCTATACAAATCATGTATACGTAAATCTAGATATACCTACTCTAAGCTCTAAATTGATCATATTTTTGTAAATACTTACTTATGTTTTAATTATTATCAAGTTATTTTTTAATGGAATAAAGTGTTGGAA

>gene11883

GCTCATTCAGTATTTAAGACGTGTCTTACTTCCTACGTGTTATTCGTTTTCCATCCATTTATGTACTTGCAAAGTACTTCTTTAAAGAGAAGACCTTGAGAAATATCAGAGCAATTTAGTACTTATTAGCAAAGGAATAAGGTTTCTGGTACAAATATTAATAGTCGGACTAGTAAATTGTTGGTTTACATACAATACTAATTTTAGCATAGACTGGTTGCGTTAAGTTGAGATCCTTTTCGTCAATCCTGCGTACTGATCGGTCGTCAATGTCTTAATAATATTAAAAAAAAAAATACTACATAGTGCCAGTGTTTCCAAAATGATTTTGTTTTTAGTATCTGTTATTTTAGTGTTAGGTTTATTAGTGTCATGGATTAGTTTAGTTAGAGAGAGTCGCCGGTTTAATATCAATGGGCCGATGCCCTTACCTATTATCGGCAATGCTCACATGTTCATGGCTAAATCTACAGAGTTCTTGGACTTGGTCACACGCTATTCGGAAAAGTATGGCAAAGTGTTCCGTGTGCATTTTCTGTCGGTTCCGACTGTGATCGTCTGTGATGCTAAATTCGCTCAGGAGATAGTATCGAGTCAGGAGCACATCAGCAAAGGTGGACCCTACAAACTGCTGGAATGTTGGCTTGGTCAAGGTCTACTCACTGCTGGAGGTCAAAGATGGAAGAGTCATCGAAGGTTCCTGACGCCCGCGTTCCACTTCAACATCCTGCAGAACTTCCTTCCCGTGTTCTGCAAGAACCAGCGCGTGCTGACGGAGAAGCTCCGAGGACTCGCCGACGGCCGCCCGATTGACATGTTCCCTATTGTCGCTTTGGCTGCCCTTGATAATGTTACAGAATCCATTATGGGTATCAGCGTCGACGCTCAAGGACATGAAAGCGAATCTGCCTATGTGAAATCAATTGAAGAAATGTCTGCTATAGTAGCCATGAGAATACAAATTCCAATTCTTGGTCCAGACGCAGTGTTTAATTTAACCCGGCATAAGGCAATTCAAACGAAAGCATTGAAGGTATTACACCAACACACTAAAAAAGTAATTGAAGAGAGAAGGCAACAGTTGAAGAACGCTAACATAACTAGTCTTAATGCAAGTAACGATGTCGGAATAAGGAACAAGCATGCGTTCTTGGATTTGTTGCTGCTTGGTGAAATTGACGGTCAAAAAATTGACGATGAGAGCGTGAGAGAAGAAGTCGACACATTCATGTTTGAAGGTCACGACACAACGACTTCTGGTATATCATACGCACTCTACTGTCTGTCTAAGCGTAGACATATTCAGGAGAAGATATATGAAGAGTTGCAAACTATTTTCGGTGACAATATGGACAGAGACCCAACGTACCAGGAACTTGGCCAAATGAAATACTTGGAGATGGTCCTTAAGGAGTCGATGCGGCTGTACCCACCCGTGGCTTTCATTGAGCGAAGGATAACAAGAGACTGTGAGATCGGAGGCCTAACAATGGTCAAAGATACATCAGTCATTCTAAATATCTACCAGATTCAACGCCAACCAGACATGTTTGAAGATCCTTTGGAGTTCCGTCCCGAAAGATTCGAAGAGTCTCTAAAGAATCCCTTCAGTTTTCTGGCATTCAGCGCTGGACCTCGGAATTGTATTGGTCAAAAGTTCGCGATGATGGAACTGAAGATCACTATCTCGGAGATTATCAAGCACTTCCACATATTACCTGTTGATGTAACGCCCCAGTTATGTTCTGACCTCATTCTGAGATCAAAGAATGGTGTCAATGTGAGGTTCATGCCAAGAAAATAAATTGTTTGCGAAAAATTAATTGATAATAATTGATAAGATTAAAGTATTTTAAGTACCATGTAATTGTAAGCATTCATATCATATCATGTGTTTATTAATACCAAACTTGTATTTTGAATAATATAATGTTGTAAAATA

>gene1203

ATGACAAAGCAAATATTTAGTAAGATGCTCTGCGGATAGATAAGGCAAGTCGCAACCTGTCGGGTCTAAATATACTATTATCGTATCAGTTGTAAACACCGAGCGTATTATAAAAAGGAGCATTCGAATTGAGCGTTCATCAGTTACAGCTGAACATTCAACGAGATTAGACAAGTTGAGACAACCAAACATGTTGTCCACAATCATACTCGCCGTAGCCATCGTGGTACTAACGCTTACGTACCTCAGAGGCAAATACAATGAAAACTACTGGAAGAAAAGAGGAATAGTTTTCTACAATAGGAATAAAACCCTTGGCCCTCTGTGGGAGTACATGACTGGAGAAAGACCATTGTTCCAAGTGTTCAACGAACTGTATTGGAGATATGAAAAGGAACCTGTTGTTGGATTGGGCTCGCTCGACTCGCCCGGACTTTATGTAAAGGATCCAACCAATATTCAGTTCATCACACAAACTGACTTCCAGAACTTTAGCCACAGAGGAGTTACAGTTTTAGAAGAAGATTACCTGGCCAATAATGTTCTGTTTTTACACGGTCAGAAATGGAAAGTAATCAGACAGAAGTTGACACCGATTTTCACAACAGCAAAGCTGAAAAACATGTTCTACATTATTGACAAAAGCGCCCAGGACTTCATAGAACATTTGAAGCAAAACCCAGAAAAACTAAAAGGTGACACATACGAACATTTAAGTCATTTCTGTACCGCTGCTCTCGCGGCCGCTGTATTTGGAATCACGACAAAATCCACGTTTGATTCTCCGTTCCGTGTAATGGCAAAACAAGCATTAACTCCTACATTATTTTCAAACATTAGGTTCGCAATAAATAACGTCAGTGATAAAGTCTTTAGAGCATTGAGACTAAAATTCTTCAAAGAACAAGAGGGCTTCTTTATTGGAGCTATTAAAGAGGTACTCAAACAACGCAGATTAGATACTACTGCCCGACACGACTTCATAGATTTGTGTATTGATCTTCAAAAGAATGGTCCAATGGTGGATAGCGATACTGGATACACATTGGAACCTACAGACGAGCTACTGGCGGCTCAAGCGTTCTTCTTCTTCTTAGCTGGCATTGACCCCACTACTGCTGGAGCATTTGGACCTTTGTATGAATTGGGTAAACACCCAGAAATCCAGAAGAAGGTACAAGATGAGGTAGATGCTGTCTTCGAGAAATACGGCGGCAAGCTGACGTATGATGCAGTACTGGAACTGAAGTACACCGACTGTGTATTCAATGAGGGTCTTCGTTTGCATCCTCCAATTGGCTTCAACACGCGGCAGTGTGTGCAAGACTCCGTTCTGCCTGTCGGCAATGTGCCTGTTGAGAAAGGTACGAAGATATTCATACCTACATATGAGTTGCACCACGATCCTAAATATTTCCCGGAACCTGAGAGGTTCGACCCTGATCGCTTTGCTAGAGGAGAGGTGAGTGATACAATATATATGCCGTTTGGTATTGGCAAAAGATTGTGCATTGGTGTACGATATGCTCGTATGCAAATCCTGACTGCTTTCGCTCATCTCCTAAGAAACTATACACTGGTTACGCACATTAAAAAAGGCGGTGTACAGTATAGAAAGGAGCAATTCCAAGTGCGGCTCGATAACTGTGATATCGAGTTGGTGCCCAGGGAACAGTGATGGAAAAACGAATGTAGCTAGTATAATGGACTTGAAGATAGGTTACTTATGTTGTGAAATTGTGAAGTGTTTTTTACGTGAATAGCAAAACAATTTTAAATAATTGGTAGTAATACATGTACTAATGTACATATTTGTTTAATTATTCATACACTAAAAAAATTACAGTAAGTGTTTGTAAGCTGTTGTTTATATTGTTTATATTTTGTGTATCTATAAAAAGCCGCAATACGGTCTTATGATTTGTTTATAATATATATAGCTATGAATGAATGAAATGAATAAAATGTGATTGTATTTAAAA

>gene12037

GTAGTGAGACACACTCAGTGTCGTTTGGGGATAGGACAATTTGTCTACCCCGCTAGTGAAGGTGATTCTTAACAAGAGTGTAAAATCATCCAAACATGAGCTACACGACTGCAGAGAGCGTGGTGCCCACCAGCACCTGGGCGGCCACCAACTTGTTCTACGTTCTGGTGGTACCAGCCCTGATCCTCTGGTACGCGTACTGGCGCATGTCCAGAAGACATTTCTATGAACTCGCAGCAAAACTGCACGGACCCCCTGGCCTACCTCTGCTCGGAAACGCGCTAGAATTCACCGGTGGATCTCACGACATCTTCAGAAATGTAATCGAGAAGAGTATTCCTTTCGATGGAGAATCCGTAGTGAAAATTTGGATCGGACCCCGACTTCTGGTGTTCTTGTACGATCCTCGTGATGTTGAGCTGATCCTCAGCAGTCACACACACATTGACAAGGCTGATGAGTACAGATTTTTCAAGCCTTGGCTCGGAGACGGTCTTTTGATCAGTACTGGCCAGAAATGGAGGTCTCACCGCAAGCTGATTGCCCCCACTTTCCACTTGAACGTTCTCAAGAGTTTCATTGATCTGTTCAATGCCAACTCCAGGGCCGTTGTTAACAAGCTGAAGAAGGAGTCAGGAGAATTCGACTGTCATGACTACATGAGTGAATGTACCGTAGAAATCTTACTTGAGACTGCTATGGGAGTCAGCAAGACTACACAAGACCAGAGCGGATTCGAATACGCTATGGCTGTAATGAAGATGTGTGACATCCTTCACTTGAGACATACTAAGATCTGGCTCAGACCAGACCTCCTCTTCAAATTTACCGACTACGCTAAGACTCAAACTAAACTCCTTGATGTCATCCACGGCCTGACCAAGAAGGTTATCCGAAGGAAGAAGGAGGAATTCAACTCTGGCAAGAGGCCCACTATTTTAAATGACTACAGTTCCACCGCTGAGGAAACCACCAAAACTACTTCCGTCGAGGGTCTGTCATTCGGTCAAGCGGCCGGTCTTAAGGACGATTTGGATGTTGATGACTCTGATGTAGGACAGAAGAAACGTTTGGCCTTCCTTGACCTCCTCCTCGAAAGTTCACAGAGCGGTGTTGTTATTTCCGACGAGGAAATCAAAGAACAAGTGGACACTATTATGTTCGAGGGTCACGACACAACTGCCGCTGGAAGCAGTTTCTTCCTGTCGATGATGGGTATCCACCAGGACATTCAAGACAAAGTTATCGAGGAACTTGACAAAATCTTCGGTGACTCGGATCGTCCAGCCACTTTCCAGGACACTCTTGAAATGAAATACCTGGAAAGGTGCCTCATGGAAACACTCAGAATGTTCCCGCCAGTGCCTATCATCGCACGTCACTTGAAGCAAGACATTACTCTTCCTTCATGTGGTAAGCAAGTGCCTGCTGGTACAACCGTAGTAGTAGCAACATACAAACTGCACCGTCGCCCCGACGTGTACGAAAACCCTACCAAGTTCGACCCCGACAACTTCCTGCCTGAGAAATCAGCGAATCGCCATTACTACGCATTCGTGCCATTCTCCGCTGGACCCAGAAGTTGTGTCGGTCGTAAATACGCCATGTTGAAGCTGAAGATTATTCTATCTACGATACTTAGGAATTTCCGCGTCTACTCTGATCTTAAAGAGTCCGACTTCAAACTGCAGGCTGACATTATCCTCAAGCGAGCTGAAGGCTTCAAAGTTCGCTTGCAACCACGCAAACTGGCCAAGGCGTGCTGATTAGTTACATTATAATTCCTAATTTTAATTTTTATGTAAATATGGACGCATGGTTCTCGCATTTTTACTAAGTCCGTGCTGATAGCACGGAATAAAATATTTTCATAACATATTGTTAATGTCAAGAAAGATGTTAAATCGGTGGCAGACCTTTAGACACGGGAAGGTGTTGATAATGGTGCAACAGTTGGCACGTTTGCTCAATATTATTTAACTATGCGGTTATTTTAAGTACTTTGTAAATAGACATTTAAATTATACCCTTTTTACGTTGTACGAATAAGACGAACCAATAAAGTATAGGTAACAAATAAA

>gene1205

CATTCAACGAGATTAGACAAGTTGAGACAACCAAACATGTTGTCCACAATCATACTCGCCGTAGCCATCGTTGTACTAACGCTCACATACCTCAGAGGCAAATACAATGAAAGATACTGGAAGAAAAGAGGAGTAGCTTTCTATCAAAGAAATAAAACCGTCGGCCCTCTGTGGGAGTTCATGACAGGAGAAAGACCATTGTTCCAAGTGTTCAACGAACTGTATTGGAGATATGAAAAGGAACCTGTTGTTGGATTGGGCTCGCTCGACTCTCCCGGACTGTATGTAAAGGATCCAACCAATATTCAGTTCATCACACAAACTGACTTCCAAAATTTTAATCACAGAGGAATCACAGTTTCTGACGAGGATTACCTGGCTAATAATGTTTTGTTTCTACACGGCCAGAAATGGAAAGTAATCAGACAGAAAATGACACCACTTTTCACAACAGCGAAACTGAAAAACATGTTCTACATTATTGACAAAAGCGCCCAGGACTTCATAGAGCATTTGAAGCAAAACCCAGAAAAACTAAAAGGTGACACATACGAACATTTAAGTCATTTCTGTACCGCTGCTCTCGCGGCCGCTGTATTTGGAATCACTACAAAATCCACATTTGATTCTCCGTTCCGTGTAATGGCAAAAGAAGCATTAACTCCTACATTGATGTCCAACTTCAGGTTCGCAATAAATAACCTCAGTGATACACTCTTTAGAGCATTGAGACTAAAATTCTTCAAGAATCAAGAGAAGTTCTTTATTGGAGCTATTAAAGAGGTACTCAAACAACGTAGATCAGATAGTACTGTTAGACACGACTTCATAGATTTGTGCAAAGACATTCAAAAGAATGGTCCAATGGTAGACAACGAAACTGGATACACATTGGAACCTACAGACGAGGTGCTGGCTGCTCAAGCGTTCTTCTTCTTCTTAGCTGGCGTTGATCCTACAGCAGCTGGAATGTTTGGACCTGTATTTGAATTGGGCAGACACCCAGAAATTCAAAAGAAGGTACAGGATGAGGTAGATGCCATCTTCGAGAAATACGGAGGTAAACTGACGTATGATGCAGTACTGGAACTGAAGTACACCGAATGTGTATTAAATGAAGGTCTTCGGTTACATACTCCAATTGGCTTCAACTCGCGGCAGTGTGTGAAGGACTCGGTTCTGCCTGTCGGCAATGTGCCTGTTGAGAAAGGTACGAAGATATTCATACCTATGTATGAGTTGCATCACGATCCTAAATATTTCCCAGAACCTGATAAGTTCGACCCTGATCGCTTTGCTAGAGGAGAGGTGAGTGATACGATATACATGCCATTTGGTATTGGCAAGAGGTTGTGTATTGGTGCACGATATGCTCGTATGCAGATCCTGACTGCGTTAGCACATCTCCTGAGAAACTACACTCTGGTCACACACATCAAAAAGGGTGGTCTGCGGTATAGAAAGGAGCAATTCCAAGTACGACTCGATAACTGTGACATCGAGTTGGTTCCCAGGCAATAGTAAATAGCCAAAATTACTTTAGCTGGTACAATGGACTTGTAAATAATTTATTGTAAAACTGTGAAGTGTTTACTATGTGAATTATGAACTTGTTGCTAATTGTTATGTATTTACACATATCTTTATGAGTAATAGTAAGAGTGTGTACGTTAATATTTGATTTATTTTGTGTAAATACATATGTACATAAAGCCGTAAAATCGGTCTTACAAAGATTTTATTGTGATATAGTTATAAGGCATCTGAATAAATTAATATGATTAAATTG

>gene1206

TAACGAGGTTAGACAAGTTCAGACAACCAAACATGTTGTCCACAATCATACTCGCCGTAGCCATCGTGGTACTAACCCTCACGTACCTCAGAGGCAAATACAATGAAAACTACTGGAAGAAAAGAGGAGTAGCTTTCTACCAAAGAAATAAAACCGTCGGCCCTTTGTGGGAGTTCATGACAGGAGAAAGACCATTGTTCCAAGTGTTCAACGAACTTTATTGGAGATATGAAAAGGAACCTGTTGTTGGATTGGGCTCGCTGGACTCGCCCGGACTTTATGTAAAGGATCCAACCAATATTCAGTTCATCACACAAACCGCTTTCCAGAATTTTAGTCATAGAGGAATCATAGTTTCTGAGGATGATTACCTGGCTAATAATGTTTTGTTTCTACACGGCCAGAAATGGAAAGTAATCAGACAGAAAATGACACCACTTTTCACAACAGCTAAACTGAAAAATATGTTCTACATTATTGATAAAAGCGCCCAGGACTTCATAGAACATTTGAAGCAAAACCCAGAAAAACTAAAAGGTGACACATACGAACATTTAAGTCATTTCTGTACCGCTGCTCTCGCGGCCGCTGTATTTGGAATCACTACAAAATCCACATTTGATTCTCCGTTCCGTGTAATGGCAAAAGAAGCATTAACTCCTACATTGATGTCCAACTTCAGGTTCGCAATAAATAACCTCAGTGACACACTCTTTAGAGCATTGAGACTAAAATTCTTCAAGGATCAAGAGAAGTTCTTTATTGGAGCTATTAAAGAGGTACTCAAACAACGCAGATCAGATAGTACTGTGAGACACGACTTCATAGATTTGTGCATGGACATTCAGAAGAATGGTCCAATGGTAGACAACGAAACTGGATACACATTGGAACCTACAGACGAGGTGCTGGCTGCTCAAGCATTCTTCTTCTTCTTAGCTGGCGTTGATCCTACAGCAGCTGGAATGTTTGGACCTGTATTTGAATTGGGCAGACACCCAGAGATCCAAAAGAAGGTACAGGATGAGGTAGATGCCATCTTCGAGAAATATGGAGGTAAACTGACGTATGATGCAGTACTGGAACTGAAGTACACCGAATGTGTATTAAATGAAGGTCTTCGGTTACATACTCCAATTGGCTTCAACTCCCGGCAGTGTGTGAAGGACTCGGTTCTGCCTGTAGGAAATGTGCCTGTTGAGAAAGGTACGAAGATATTCATACCTATGTATGAGTTGCATCACGATCCTAAATATTTCCCAGAACCTGAGAGGTTCGACCCTGATCGCTTTGCTAGAGGAGAGGTGAGTGATACGATATACATGCCATTTGGTATTGGCAAGAGGTTGTGTATTGGTGCACGATATGCTCGTATGCAGATCCTGACTGCGTTAGCACATCTCTTGAGAAACTATACAATGGTCACACACATCAAAAAGGGTGGTCTGCGGTATAGAAAGGAGCAATTCCAAGTGCGACTCGATAACTGTGACATCGAGTTGGTTCCCAGGCAACAGTAAATGGCCAAAATTACTTTAGCTGGTACAATGGACTTGTAAATAATTTATTGTAAAACTGTGAAGTGTTTTCTATGTGGTTTTTGAAAATAATCTAAATGTTATTTATTTACACATTTTTTCTA

>gene1207

ACGCACATGGCTTTATAAGCCATTTATGCCGCGCTCTCTGCCAGTTGCCATTTGAACTTGAAGAGAGATATCTTCTAAAATGTTGTTAGTGTTACTAGGCGTTGTTGGTGTAATACTTGCATTAATATACCTAAAGGGAATATATAATCAGCATTATTGGAAGAAACGTGGTGTAGCCTTCTATGACAAGCACAAGACAGGAGGACCTCTATGGCAGTTCGCAATCGAAGATCGGTCATTCTTCCAAATCTTGAACGATATCTATTGGAACTATGAAAAAGAACCAGCTGTCGGTCTGGGCTCATTCCATGACCTGGGACTATTCGTGAAGGATCCGACCAACATACAACACATCGCACAAGTTGATTTTCATTCATTCAATCATAGAGCTATTGCATTTACCGAGGATGATGTCTTGGCACACAATGTGCTAATGCTCAACGGTCAGAAATGGAAGCTAGTCAGGCAGAAAGTGACACCAATTTTTACAACTGCCAAACTTAAAAACATGTTTTACATCATCGACAAAAGTGCTCAGGATTTTATAGAGTATTTAAGAGATAATCCAGAAAAGTTGAAAGAAGATCTGTTTGATACTTTGGGTAATTACTGCAATGCTGCCATTGCTGCTGCTGTATTTGGAATCCATACACGCTCAACTTTCGATTCTCCATTCCGTATGATGGCTAAAGAGGTTCTGACTCCTACTTTTTTCACAAATTTGAAGTTTGCGATAAGTGGAGTAAGTGAATCACTCTTCAAATTTTTAAAACTCAAGTTATTCAAAACAGAAAAAGAGAAATTTTTCATCGCAGCGATCAAACAAGTGATTAGGCAACGAGAAAAAGAAAACGTGAAGAGACACGACTTTATAGATATATGTGTAGAGCTCCAGAAGAATGGGACAATGGTGGACCCAGATACTGGGTATGCGCTGGAGCCGACAGATGAGCTGATGGCTGCTCAGGCTTACTTTTTCTTCTTGGCAGGTACTGACCCTACAATCGCTGGAATATTCGGTACTTTAATGGAATTGGGAAGAAATCCCGATATCTTGAAGAAGGCACACGAGGATGTGGATAGAGTATTCGAGAAATATAATGGTCAACTGACGTACGATGCAGTTGGAGAAGTGAATTATTTGAAAAATGTATTGAGTGAGGTCTTTAGAGTGCATCCTCCGATAGCTTTTGTAATGCGACAGTGCGTGCAGGATAGTGTTCTGCCTGTAGGCAATATTCCTGTTGAGAAAGGTATAAAGATATTTATGCCTATATTTGACTTGCATCATGACCCTAATTATTTTCCGGAGCCTGAGAAATTTGATCCTGACCGCTTCGTGAGGGAAGGAGAGATAAATGATGCGACTTATATACCGTTTGGTAAGGGTGGTCGATATTGTATTGGAGCGAGATATGCTAATATTCAGATCATGACTGGGTTGATCCATATTCTAAGGCATTATACAGTACGTACATTTGTGAGAAAAGGAGGCGTTAAGTACAATAAGGAGCAATTTCAAGTGCGTTTGAGAAATTGTGATGTTGAGTTGGTGCCTAGAAAATTGTGATTGGTGCAAGCGAGTGTAATTGCTATTGTGTTTCGAGCAGAGGTTACTTTTACCTGTACGCGTAGAAGTATAGTAGTTAGTTCGTAATTTAGTTTCGACAGTACTGTTTTTATATATTAATTTATTATAGTATCTACAGTTATCCGTAGATAATTTTGTGAATTCTTAGAATATTTTTTTAATATATAGAGAAAATTTCTACAAATAAATTAAAGTTTACATGTGTATTTATTTATGCATGCTTAGCATAACATCTTGCTTATTAATTTCTGAGAAATTGGACGAAGTAGGTATGTACCTCTATATTAAGTCCATATTCTTTTAACTCTATCCTACTTATGAGAATTTTGAAATCGAAATTTTCAGTAATGCTTTTTCAAATCCAAAGGTTAAGAGCAGTCTACGAAATATTAAAATAAAGAACAATTTTTAATATCTAATTAAATATCATATCCACTTTTCACCAGTTATATTACTAGCTTCATGTAATGTGTACAATGGGCTATAATATTGTATAAGATTATGCAGTACAGTGACTGCCTCATTGGCCGAGTGGTCGCAAGTGCGACTGCGGG

>gene12080

TCATTGAAGAAAGCACGTCGTATTGGTTGCTACAGCAAAATGGCGCCGTTTGTCGACGACACCGTTTATGTGGATTGGACCACACGTTTCGTGTTCTACGCTCTGGTAGCAGTTGTGTCTGGTCTATGGATGATACATAGATGGCAGCAGCAATCCAGATTATTCAGGTTGGGCAACGTTCTGCCAGGTCCTGAATGCGTACCGTTTTTTGGTAACGCTCTCCTTGCTTTGGGAAAAAAGCCAGACCAATTGGTGAACATTGGTCTTGAATACGCCGAAAGGTATGGCACCGTAATCCGAGGTTGGCTTGGCTCCAAATTGATAGTGTTTCTCCTGGATCCCGAAGACATTGAAATCATCCTCAACAGTCAAGTTCACATCGACAAAGCTCCAGAATACCGATTCTTTAAACCATGGCTTGGAGAAGGTCTTTTAATCAGTACAGGAAACAAATGGCGGTCCCATCGCAAAATGATTGCCCCAACGTTCCACATCAACATTCTGAAATCATTCGTTGGTGTTTTCAACCAAAACAGTAAGAATGTTGTCGAAAAACTGAGAAAGGAAGTTGGAACAACGTTCGATGTTCACGACTACATGAGCACTGCGACAGTGGATATTTTGCTAGAAACTGCAATGGGGATTACAAGAAAAACTCAAGATGAATCCGGTTTTGATTACGCTATGGCTGTTATGAAGATGTGTGACATTATTCACCAAAGACACTACAAATTCTGGCTGCGATTTGATACAATTTTCAAATTCAGCTCTTTCTTTAAAAAGCAGAAACAGCTTTTGGGTATAATACACGGACTTACTAATAAGGTGATAAAGAACAAAAAACAAACGTACCTTGAGAACAAAGATAAAGGTATTATACCACCAACACTGGCAGAACTGACGCAAAATGAAGAAGATGGAAGTGTTCTAGCCAATGATGCTAAAACACTTTCTGATGCGGTGTTCAAAGGGTACCGCGATGACTTGGACTTCAATGACGATAATGATGTTGGTGAAAAGAAAAGACTGGCTTTCTTGGATCTCATGATTGAATCAGCTCAAAATGGTACTCATCAGTTAACTGATCACGAAATCAAAGAAGAAGTAGACACTATTATGTTTGAGGGACACGACACTACGGCTGCTGGTTCCAGTTTCGTCCTATGTCTGCTGGGTATCTACAAAGATGTTCAAGCAAAGGTTTACAATGAACTGTACGATATTTTCGGTGACTCTGACAGGCCAGCCACTTTCGCCGACACTTTGGAAATGAAGTACCTTGAGAGGGTCATATTGGAAACTTTGAGGTTGTACCCACCAGTACCAGCCATCGCAAGGAAATTGAATACAGATGTTAACATTGTTACCAACAATTACCTCATTCCTGCAAGCACCACCGTAGTTATCGGAACGTACAAGGTCCACCGCAGCCCCAAACACTACAAAGACCCTGATACCTTCAACCCTGACAACTTCCTACCAGAGAACATGGCCAACAGGCACTATTATAGCTACATACCCTTCAGCGCTGGACCCAGGAGTTGTGTCGGTCGCAAGTACGCCTTGCTGAAGCTGAAGATTTTGCTATCAACGATTTTGAGGAACTACGAATGCACATCCACAGTACCTGAGAAGGACTTCAAACTTCTTGGAGACATTATTTTGAAAAGATCAGACGGCTTCAGACTGCAGATTGCTCCTAGGATAAGAAAACCAACAAATGTAGCATAGATTTATACTTAGTAATTTAGTACCTATTGCATGAATATGTACGTAAAATGACCTAATGCTTTCATTATGGTAGTCATGTTCTACTGAAACAAAGACTATATTAATATTCATTTATTTGTACATCAATACAATACTTAATTATTGACGTGAGTACCAATACTTATAATGCTAAGAA

>gene12081

GTTTAGTACATAGGTTAATTGTGATTGGACCACGGGTAGATAGGCTTGACTGAAATATACTTAGGATTAAATAAAAATAAATCACAATGACGTCGTTGGTCGACGAGACCGAGGTGCCGAGCTACAACACAAGACTGATCTTCTACCCACTCCTGCTCCTCGCATCCAGCATCTGGATCCTATACAGATGGCAGCAGAAGTCACGCATGTACAGATTCGCTGAGAAGATCCCTGGACCTGCTAGTATACCTTTCGTTGGAAATGCACTTATTGTGCTCAGGAAGAAACCCAGTGATCTGGTAAAGCTCGCCCTTGAGTATGGAGAAAAGTTCGGCAACGTCGTCAGAGTGTGGATGGGATCAAACCTTATCGTCTTCCTTACCGACCCAAATGACGTTGAGATCATCCTCAACAGTCAGGTCCACATCGACAAGGCCACTGAATATAAATTCTTTAAACCCTGGCTTGGAGAGGGTCTCCTCATCAGCACAGGTGATAAATGGCGTTCCCACAGGAAAATGATCGCTCCTACATTCCACATCAACATCTTGAAATCTTTCGTTGGCATCTTCAACCAGAACGGCAAAAATGTCGTAGAGAAAATGCGTCCAGAAATGGGCAAGGAGTTCGATGTCCATGACTACATGAGCGGTGTCACCGTCGACATTTTGCTGGAAACTGCTATGGGAATTACCAGGAAGACCCAAGATGAATCTGGATTTGACTACGCTATGGCTGTAATGAAGATGTGTGACATCATCCACCAGAGGCACTACAAACTGTGGCTTCGTTTCGACGCCATCTTCAACAATTCACCCTTCTTCAAGCAACAGAAACATCTCTTGAACATCATCCATGGTCTTACCAACAAGGTTATCAAGAGCAAGAAGGAAACATACCTAGAAAACAAAGCAAAGGGTGTCGTCCCACCAACTCTGGAGGAACTCACCAGGACTCCTGATGAGAGCGTCCTTGCCAACAACGATAAGACTCTTTCCGAAGACGTGTTCAAGGGATACCGTGATGACTTAGACTTCAACGACGAAAATGACATTGGTGAGAAGAAACGTCTTGCTTTCTTGGACTTGATGATCGAATCATCCCAGAATGGAACCAACAACATCAGTGACCACGAAATCAAGGAGGAAGTCGACACTATCATGTTTGAGGGACACGACACCACTGCCGCTGGCTCCAGTTTCGTGCTGTGCCTCTTGGGTGTCCACAAGCACATCCAAGACAAAGTTTACAATGAGCTGTATGAGATCTTCGGAGACTCTGACCGGCCCTCTACCTTCGCCGACACCCTGCAGATGAAGTACTTGGAAAGAGTCATCCTTGAATCCCTCAGAATGTACCCACCAGTGCCCATCATCGCCAGGAAACTGAACCGTGACGTGAAAATCGCTACCAACAACTACGTGCTGCCTGCTGGTACTACCGTCGTGGTTGGTACCCTGAAGATCCACCGCAACCCTCAGTACTACAAGGACCCCAACACCTTCAACCCTGACAACTTCTTGCCTGAGAACACCTCCAACAGGCACTACTACAGCTACATTCCATTCAGTGCTGGACCCAGGAGTTGCGTTGGTCGCAAGTACGCTCTTCTGAAGCTGAAGATCCTTCTGTCGACCATCCTCCGTAACTACAAGGTGACGTCTGATGTGACTGAAGACCAGTTCGCCCTGCAGGCTGACATCATCCTCAAGAGGACTGACGGCTTCAGGCTGAAGATCCAGCCCAGACAGAGGGTCCCAATCCCCACAGTCGCTTAGATCTACCAAAGAAAATATAAAACGATGATCTTATCTTCCCGAGATAAGACAGTAACTGCGATATTAAACACTCATTATGACATTACCTTATACTACTATAAGTCTTATTTATTAATTTAAGGAATATTTAAAATGTTCCTAATAATTCTCAATGTTTATTATCTCACTCCAATGGAAGAAGGTTCATAAAACACGGCCATTTATCGCTTACAACTAAAGAGTCATGTCAATGGTGTTGTTGCGCATTTACAAAACTTAAATTATGAAATGCCTACCTATTAATAAATTATTCATAATTAAC

>gene12082

AGTAATAGTTTTAAGGCCTACCAGTGAAAACGTTACAAACAGGATTTTAGGGGGAGCGATTTATTATAGTCAGCAAAATGACATCGGTCGTCGACGTTGCGGTTGACCAGAATGCCAATACCACAAGGATGATATTCTACCCGCTGCTGCTATTAGCGTCCGCGATATGGATGTACCACCGATGGCAGCATCAGTCCAGGATGTCCAAAATGGGAAACATGCTCCCAGGACCCACAGCTCTCCCGTTATTCGGAAACGCACTTATGGCTCTGTTTAAGAAGCCTGAACAATTCATTAAACTTGGCTTCGAATACGGTGACAAATATGGCTACGTGGTCCGTGGCTGGTTGGGTTCAAAACTTCTGATCTTTCTCGCAAATCCAGAAGATGTGGAAGTTATCTTGAACAGCCAAGTGCATATTGATAAGGCTTCGGAGTATAGATACTTTGAGCCTTGGCTTGGAGAAGGTCTCCTCATCAGTTCAGGTGCTAAATGGCGCTCACACAGAAAAATGATCGCTCCTACATTCCATATAAATATCCTGAAATCTTTCGTCGATGTTTTCAACCAGAACAGTAAGAGTGTGGTTGAGAAGATGAAATCTGAAATCGGGAAGACTTTTGATGTTCATGACCACATGAGTGGCGTTACAGTCGATATCTTATTGGAAACTGCTATGGGCATCACAAAGAAGACTCAGGATCAATCTAGCTTCGATTACGCGATGGCTGTGATGAAAATGTGCGATATTATCCACCAGAGGCATTACAAATTCTGGTTGCGACTGGACAGTATTTTCAAATTCCACCCATTATATAAAACACAACAGAAACTTTTGGACATTATACACGGACTTACTAATAAGGTCATTAAGCTAAAAAAGAAAATCTACCAGGAAAACAAAGCTAAGGGTATAATTCCCCCTAGTCTCCAAGAGTTGACTGATGGCAAGGCTCTCAGTAACGATACAGTTTTGGCGAACAATGCCAAAACATTATCAGACACAGTCTTCAAAGGTTACCGTGACGATCTCGATTTCAACGATGAAAATGACGTCGGAGAGAAAAAACGTCTGGCCTTTTTGGACCTGATGCTTGAATCAGCTCAAAACGGCACCAACCAGATCAGTGACCACGAGATTAAAGAAGAAGTGGACACCATTATGTTTGAGGGCCACGACACCACTGCAGCTGGCTCAAGCTTCGTGTTATGCCTGCTAGGTCTCCACCAGGATGTTCAGGCGAAAGTTTATGATGAACTGTACCAGATCTTCGGTGACTCAGACAGGCCTGCCACCTTCAACGACACTCTACAAATGAAGTATCTGGAGCGAGTTATCCTTGAAACTCTTCGGATGTACCCTCCTGTACCCATCATTGCTAGAGAGTTGAAGAGAGACGCTAAAATCGTGACTAACAACTACGTGTTGCCGGCGGGAGCTACCATTGTCATAGCAACGTACGGCATCCACCGCCACCCTCAACACTACAAGGACCCTGATACCTTCAACCCTGACAACTTCCTACCAGAGAACATGGCCAACAGGCACTACTACAGCTACATACCCTTCAGCGCTGGACCCAGGAGTTGTGTCGGACGCAAGTACGCTCTGTTGAAACTAAAGATTCTTCTATCAACCATCTTGAGAAACTACAAAACAATTTCTGACGTCCCTGAGGAAGAGTTCCAGCTTAAGGGAGACATCATTCTGAAGAGATCAGATGGATTCAGGATTAGAATCGAACCAAGAGTGAGGGTACCATCCAACGCAGCCTAACTTATTTGTAAAGCCTAGTACTATCTACAGATATGTTGTTCACTTCATGGTCGATGTAAGGACTCATAGAGCTAAGCCTGATATTTTTTTAAGACAGATATTTAAAACAATACCAGAAAAAAGATGAGGACACAATCTGAATCTCAAAGAGACCTCGAAAAATTGAACTTATCAAAAGTAACTTTTTTATAAATATAATATTTAAATAAATGAGAACTTATATTTTAATATTTATGATTCAAAAAATGTGTGTTATACCTAAATTTATTATACGATATGCATTCGTTATTCACGATACATTAATATAATGGCTTTTATGACATGTCATTAAAGCTATTATTACTTGAAAAAATACGATTATTCAAAAATATGTAAAGATTATATCTATTTTCGATTATTTCTCAGTAAAATTATTAGCAGTGCGTGAAGATATCGTTAATTTAAAACCTGATTACTAATTTTGGTAACCATAATTGTTAGACGATTTAGTTGTGTCATCGTGAGGTCAATTGATTAATTATGTCTTTAAATAAAAGTTAAAAAATA

>gene1218

GTTCCGACCCGCGGTACGAGTAAGTAACGAGGATAAAAGTATTAATTTTTAAAAGTGGTGCTACCGTGTAGGACCCCAAGCTTAGTGTTGTGACTAAACAATCATGATAATCCTATTGATCTGGGTGACGGTGCTGGTCGCCGCCGTGGTGCTGTACTTCAGACAGCTCTACTCCTCATTCAATCGCCATGGCATCAAGCACATTAAGCCAGTACCTATCTTCGGCAACATGGGCGGCATTCTGTTACGCATTGACCATATTTGTGATAATATTGTGAAGCTGTACAATGACTTCCCTGAGGAGAGGTTCGTCGGTAGATACGAGTTTGTGAGTGAGCTGATCCTGGTCCGTGACATCGAGCTCATCAAGAAGATTGCCGTCAAAGACTTCGAACACTTCCTCGACCATCGCTCTCTATTCAGCAACAGTGAATCCTATTTCTCCAGGAACCTGTTCTCTTTGAGAGGTCAAGAATGGAAGGACATGCGGTCTACTTTGAGTCCAGCGTTCACCAGCTCAAAGATGCGTTTGATGGTGCCTTTCATGGTAGAAGTCGGAGAACAGATGATGCAGTCACTCCATCAAAGTATAAAACAATCCAAAGATGGCTCCATAGACATAGAATGCAAGGACCTGACAACCCGCTATGCTAACGATGTGATTGCGTCCTGTGCCTTCGGTCTGAAGGTGGACTCGCACAATGACAAGAACAACACTTTCTATGCGCTGGGCAAGGAGACCTCCTCGTTCAATTTTCGACAGATGATGACCTTCTTCTTGCTGATTAATGCACCAGCTTTAGCCAAGTTATTAAAGTTGGATTTCCTCTCGGAGTCATCAAAGGAGGCGTTCAAGAAGCTGGTACTTGGTACAATGGAAAACCGAGAGTTAAAGAAGATCATCAGACCCGACATGATTCATTTGTTGATGGAAGCTAAGAAAGGCAAACTGACTCATGATGAAATCAAATCCAATGATTTAGCAGCTGGATTTGCGACTGTAGAAGAATCAGCTGTCGGACAGAAAGAAATTAATAGAGTATGGACTGACGAGGACCTCGTAGCACAAGCAGTACTGTTCTTCATAGCCGGTTTTGAGACCGTATCATCAGGAATGTCCTTCCTTCTCTACGAGCTGGCTATGAACCCTGATGTTCAGGATAGGTTGGCGCAGGAAATCAAGGAGAATGATGCTAAGAACGGCGGCAAGTTCGACTTCAACTCCATACAGAACATGGTCTATATGGATATGGTTGTGTCAGAGCTTCTTCGACTTTGGCCTCCTGGCGTAGCCCTTGACAGACTTTGCACTAAGGACTACAATATGGGCAAACCTAACGCTAAAGCAGAGAAAGATTTCATTCTCCGCAAAGGTAGTGGAATCTGGATTCCAGTTTACCCAATCCACCGAGACCCTCAACACTTTCCCAACCCTGACAAGTTCGACCCAGAGCGCTTCTCGGAAGAAAATAAACACACGATTAATCCTGCCGCGTATATGCCCTTCGGTGTCGGACCTAGGAATTGCATTGGGTCCAGATTTGCGCTTTGTGAAATAAAAGTAATGGCGTACCAGATCTTACGAGAGATGGAGGTGTCTCCTTGCGCAAAGACCTGCATACCAGCCAAGTTGTGTAAGAACACCTTCAACCTGAGGCTCGAAGGAGGACATTGGTTAAAATTCAAACCGAGGACGAATTAAGGATTTTCGACTTTATTACAAAGAGATTTTGTCTACCTGTAGACAGTGCTGTAGGCACTATAATGATCAAAGATATTTTTGTTAGTGACACTACTTAATAAGGTTTTATTGTGTATCTAAAAGAGAGTAGAAAATCTATTTGTCAATTTGGCGTAATATAAAGTTGTCCACTAATGATACTGTAAGCATTTTGTACCTTTAAAAGTTTTAATAGTCTGGTATATTTAAGTATTGGTTTGCTAACGACTGATGTGTACAATAATAATTTTGTTTTACACCAAAGGTGTTAACACGTTAAACGCCATTGGGGTTAGCGGCGACCGACATTAGATGAGGGGTTCTAGGTGACCAACAACGTGAGCGGAGGGATAGCCTTCAATAGTTTTCTATTGGCAGTCAAAGCTTTAATATGATATTGATGATGATGTGATTTAATTAATACAATTTGAAAAACATTGTTTAGAAGTGAGTTGCAACTGGATATAATTTTACGGTGCACCCGATTATGGGGCAATTGAACAGTTGCCTACATATAATGATATTTTGTATAGTTGTAACTAAGATTATTTTATTGTGATATACGATAGGCTAAGGTTATTTCATTACAAAAGTACGTAAGTAAAATAAATTAACACAATATCATCTGTTGTTTC

>gene1219

TCCGACCCGCGTTACAAGTAAGTAACGAGAATAAAAGTATTAGTTTTTAAAAGTGGTGCTACCGTGTGTGACCCCAAGCTTAGTGTTGTGACTAAATAATTATGATGATCCTATTGATCTGGGTGGCGGTGTTGATCGCCGTAGTGTTGCTGTACTTCAGGCAGATCTACTCCTCATTTAATCGCCATGGCATCAAGCGCATTAAGCCAGTACCTATCTTCGGCAACATGGGCGGCATTCTGTTACGCATTGACCATATTTGTGATAATATTATGAAGCTGTACAACGACTTCCCTGAGGAGAGGTTCGTCGGTAGATACGAGTTTGTGAGTGAGCTGATTATGGTCCGTGACATCGAGCTCATCAAGAAGATTGCCGTCAAAGACTTCGAACACTTCCTCGACCATCGCTCTCTATTCAGCAGCAGTGAATCCTTTTTCTCCAGGAACCTGTTCTCTTTGAGAGGTCAAGAATGGAAGGACATGCGGTCTACTTTGAGCCCAGCGTTCACCAGCTCAAAGATGCGTTTGATGGTGCCTTTCATGGTAGAAGTCGGAGAACAGATGATGCAGTCACTCCATCAACGTATAAAACAATCCAAAGATGGCTCCATAGACATAGAATGCAAGGACCTGACAACCCGCTATGCTAACGATGTGATTGCGTCCTGTGCCTTCGGTCTGAAGGTGGACTCGCACAATGACAAGAACAACAACTTCTATGCGCTGGGCAAGGAGACCTCCTCGTTCAATTTTCGACAGATGATGGCCTGCTTCTTGCTTGTTAATGCTCCAGCTTTAGCTAAGTTTTTAAAGTTGGATTTCCTCTCGGAGTCATCAAAGGAGGCATTCAAAAAGCTGGTACTTGGTACAATGGAAAACCGAGAGTTAAAGAAGATCATCAGACCCGACATGATTCATTTGTTGATGGAAGCTAAGAAAGGCAAACTGACTCATGATGAAACCAAATCAAATGATTTAGCAGCTGGATTTGCGACTGTAGAAGAATCAGCTGTCGGACGGAAAGAAATTAATAGAGTTTGGACTGATGAGGACCTCGTAGCACAAGCAGTACTGTTCTTCATTGCCGGTTTTGAGACCGTCTCATCAGGAATGTCCTTCCTTCTCTACGAGCTGGCTATAAATCCTGATATCCAGGAGAGGTTGGCGCAGGAGATCAAGGAGAACGATGCTAAGAACGGCGGCAAGTTCGACTTCAACTCCATACAGAACATGGTCTATATGGATATGGTTGTGTCAGAGGTGCTTCGACTTTGGCCTCCTGGCGTTGCCCTTGACAGACTTTGCACTAAGGACTACAATATGGGGAAACCTAACCCTAATGCGAAGAAAGATTACATTCTCCGCAAAGGCACTGGAATCTGGATTCCAGTTTACCCAATCCATCGAGACCCTCAACACTATCCCAACCCTGACAAGTTCGACCCAGAGCGCTTCTCGGAAGAAAATAAACACACGATTAATCCTGCCGCGTATATGCCCTTCGGTGTCGGACCTAGGAATTGCATTGGGTCCAGATTTGCGCTTTGTGAAATGAAGGTAATGGCGTACCAGATCCTACGAGAGATGGAGGTGTCTCCTTGCGCAAAGACCTGCATACCAGCCAAGTTATGCAAGAACACCCTTAACCTGAGCCTCGAAGGAGGACATTGGTTGAAATTCAAACCGAGGACGAATTAATATATCGGATGATTAATATCATTTGTTAATTTGTTAATTAAAGGGCATTGCCTAAAATTGGTGCCTTTCTTTTTTC

>gene12413

GCATGCTCGGTCAATTCGTCAGTAGTGTACTCGTGGGCCTCGGATAGAGTGAACATTATACCCGTGTATGTATTCGATCCAAACAAAATGTTCCTCATATTAGTGATAATTGGTTTAGTGGCACTTTATTTCTACGGAACGCGGACATTTAACTACTGGAAATCAAGAGGCATTAAACATGATAAACCAGTACCTATTTTTGGAACGAACTTAAAACAGTTCATGCAACAGGCCAGTATGTGCATGATGGCTACTGAAATGTACAACAAATATCCAGAGGAAAAAGTTGTCGGATTCTACAGAGGTACTGATCCAGAATTAGTGATAAGAGATCCAGAAATTATTAAAAGGATCCTTACGACAGACTTTCAATGTTTCCACTCAAGAGGCCTTACTTACCACAAGACTTGTGTCGAACCCCTGTTAAGGAATATTTTCTTTGCTGACGGAGACTTATGGCGTTTGATCCGACAGAGATTTACTCCTGCTTTCAGTACAGGCAAGTTGAAGGCAATGTTCCATATCATCACAGACAGAGCAGAGAAATTGCAGGTTATCACAGAAGAGGTCGTTGATAGGGAATATTATGACGCCAGAGAACTCATGGCCAGATACACAACAGACTTTATCGGTGTCTGCGCTTTCGGGATTGACATGGACTCTTTGAGCGATGAAAACTCTCACTTCCGAAAATTAGGTAAAAGAATTTTCGAAAGAAGGTTCAGAGATGCAGTAGCCGGTGCCTTGAAGATTATGTTCCCTGAAGTGTTCAAGCACATGCATTTCCTAGCACCCGAGTTAGAAACCAACATGAAACATTTAGTGCAGACTGTGCTTAAGAACAGAAACTACAAGCCATCAGGTAGAAACGATTTCATTGATCTAATGTTAGAGCTCAGAGAAAAGGGAAAAATTATTGGAGAGTCTATTGAACACAAAAATCCAGATGGTACTCCAAAAATTGTGGATTTGGAAATGGACGATTTACTAATGACAGCTCAAGCGTTTGTATTCTTCGGAGCTGGGTTCGAAACGTCATCCACTGCCTCAAGTTACACTTTACATCAGTTAGCGTTCCACCCAGAATATCAGAAAAAGGTACAAGAAGAAGTGGACACTGTGTTAGCCAAACATAACAATAAACTGACCTACGATGCTATCAAGGAGATGAAATATTTGGAGATGGCATTTTATGAGTCCATGAGAATGTATCCATCGGTTGGGTACTTGATCCGACAGTGCTGTGTACCTAAATATACGTTCCCAGAAATTGATTTGACGATCGACGATGGCCTTAAAGTAATGATACCTATACAAGCTATCCACAGAGATGAGAAGTACTTTAGAGAACCAAACAAGTTTGACCCTGAAAGGTTTAGTGACGGCACAAAGGAGGATATTAAGAACTTCGTGTATTTGCCTTTTGGAGAGGGCCCACGATCCTGTGTTGGTGCCAGACTAGGACAGATGCAGTCAATGGCTGGTCTTGCAGCAGTTCTACAGAAGTACTCTGTAGAACCAGCAGCTTGCAGTCGCCAGGAACCATTGCCTGATCCAGCTGGAATTGTCTCCGAAGGTTTTGTTGGCGGTCTACCGCTTAAAATAAGGAGACGAGTGAAATAGCTTCAAAAACCTTACTATTATTTTTAATATAGACTGACTTTTTATCTTTACGTAAATACTGCTTTAAAGAAGGAGTATTATTACTGTTTTTATTAGCTCAAAAGTAACTTTTATTGTGATGTAAATTAAGATTGAGTTTACCAATTGACCATAAAATATTACATAACAATGACGCCATGACCTTGCTAAAATTGATGTAATTATTTTTATTTACTTGATTATAGTATCTAAATTATTTATTAAAGGGATAAAAAAGACCTGTTAATTTGTATAGTAATTGTAATAAAATACTTACCATTGATA

>gene12725

ATGATTACAGAAGTCATAGCCTATTTGCTAAGTTCTCTTATATTTTACGTTATATATGAATATAAGAAGATACATTATAATTTTAACAAACATGGAATTAAATTCAAGCCTGGCTACCCGATATTTGGAAATACCTTTAACAGTACGTTCCTTTTCAAACATCTTATCGAGGATATTGATGCAGTGTATACGGCATTCCCTCAGGAAAGATACGTTGGTTTTATAGAAGGCATGAAGCCTATCATATTAGTTAGGGATCCTGAGCTCATGAAGGTCATTACAATTAAGGATTTCCACTACTTTGTGAATCGCAAGGAGATGTTCCCGAAAGAGATCGAGCCTTTACTGGGGTCTAGTCTTTTGAATATGGAAGGCGATGAATGGCGTAAGATGCGTAGTCGGCTTAGTGGTGCGTTCAGTGGCTCCAAAATGAAATGTATGCTGCCCTTCATGGTGGAAGTTAGCAAAAATATTTCACGTTATTTAGACGAACATCAACTTGAAGACATAGATGTGCTGGACCTGATGCGCCGCTACAACACAGACGCCGTGGCTTCCACTGGCTTTGGTCTCCACGTCAACTCAATAAGAGATAGAGATAATAAGTTCTTTACTATTGGTAAAAAAGCGGTGACCTTCACATTTTGGAGACGAATGTACTATTTTATAACGATACAGTTTCCAGCTGTGGCTAAAATTATGCAATTTTTAGGTATCGAACTTCTTTCATCAGAAGGCACCGAGTTCTTCAGAAATATTGTTTCCGATACAATAGCTTATAGAAAAAAGAATAATGTGGTGAGACCTGATTTCATTCATCTACTAATGGAGGCCGCACAAGATTTAACTTTAGATGAAATCACTGGTCAAATATACTTCGCTTTTCTAGCAAGTTATGAGTCTAGTTCAAGTACTCTGATGATGTGCATCCATGAACTAGCTCTCCGCCCAGATATAGCAGAAAAATTGTACCAGGAGATCAGAATAAAGCAGGAGAAATTTGGAGATCTAAATTATGAATGTATTATGGAGTTAAAATACATGGATTGCGTGTTAAATGAGGCGGCGAGGAAATGGTCTGTGGCTGTAGTTATGGACAGAGTTTGTACTGAGTCCTACATGTTACCTCCTCCAAGAAAGAATGGTATACCTTATTTGGTTCAGCCAGGGGACGTGGTTTATAATGTGGTGAACTCAATCCACATGGACGAGACGTACCATCCATCACCAGACACCTTCAATTCTGACAGATTTTCTGACGCAAACAAGTATAACATCAAGTCCTTCACACACATGCCTTTTGGAATGGGACCTAGAAGTTGTCTAGGAATGCGATACTCAATGCTGAAAATGAAAGTGCTACTATACCATATAATACTAAACTACAAAATAGTTCGGTGTAAAAGAACCTCCGATCCTCTTCATTTGCAACCACTAGACTTCAGCGTCAGGGCTATTGGAGACACTTACGTGCAATTCCAGAGAAGACCATGAACGAGCGATAAAATGAACAATAGACTTTTTGTTATT

>gene12726

ATAACACAGTACCCTGGTAGCCCGCGGAACACATCAACATGATATTGGAAATATTAATATTTGTGATAACAGTGATTGTTGCATACTATTTCTATAGTAGTTGGAAAATAAATAACTACTTCAAGCAACGAGATGTGAAGTACATTCCCGGTGTGCCGTTTTTTGGCAATATATTTCATTCTACTTTTCTAATCAGGCATTTTCTGGACGACCTTCAAAGTGTTTACGATGCTTTCCCTGATGAAAAATACGTTGGCTTCCTGGAGAACTTGGTACCGGTACTGATGATAAAGGATCCGGAACTGATCAAGTCTATCACTATCAAGGACTTTGACCATTTCACTGATCATAAGGAGTTCTTCACGACAGACTCCGAACCTTTGTTCGCGGGCAGCCTGCTGATGATGAAAGGTGATAAATGGCGCCAAATGAGAACGACTTTAAGTCCAGCGTTCACCGGGTCTAAAATGAAGATGATGCTACCTCTAATCGTGGATAGTGCTGACAATATTATCGAGTATTTGAATGACCATCAAACTGAAGATATTAACATAGACGACCTGATGCGCCGGTACACGAGTGACGTCATAGCGACTACTGCTTTTGGTCTGAAGGTCAACTCTTTGAAAGACAGAGATAATGAGTTCTACAGAGTTGGCAGCTCTCTGTTTGAATTCACCCTCACTCAGAGATTACTGATGTTCAGCACTCTATTCCCTTCAGTCTCAAAGAAACTTGGAACTCGTCTATTTCCGGACACGACTTATAATTTCTTTAGGACAATTGTTTCATCTACATTGGAATATAGAAAGAGAGAAAAAGTGGAGAGACCTGACATGATTCAACTACTCATGGAAACTCCAAAAGAATGGACTCCAGATGAACTGACAGGCCAAGTATTCATATTCTTTGCTGCTGGCTTCGAAACATCTGCCAGTGGACTTGCCATGGCTATCCATGAGCTAGCATTACACCCTGACATCCAAGAGCGATTGTACCAGGAGAGCAGCCAGTTTAAGAACGATAAGGAACTTACATTTGACAAACTCAGCCAACTGAAGTACTTGGAATGTGTGATTAATGAAACTCTGAGGATATGGTCCCCTGCTATCTTTATGGACAGAACCTGTGTGAAGACTTATGAACTGCCTCCACCCAGAGAGGGCGGAAAACCATGCATTGTTAAACCAGGTGACGTTGTTTACAACATGGTGAACTGTCTACATATGGATCCGAAGTACTTCCCTGAGCCAAAAGTCTTCAATCCTGACAGGTTCTCAGAAGAGAATAAGCATAACATCCAGCCTTGTACCTTCGCACCATTTGGGGGAGGTCCAAGGATTTGTATTGGAGTAAGGTTTGCTATGATGGAGATCAAGGTTCTGCTGCATCACATTATATTGAACTTCAAGATTGTAAAAACGAAGAAAACTTTGGACCCCATCAAGTTGAAGCCTCATATGTTTAACATTAGGGCTATGAACGGTACCTGGGTTAGCTTCGAGAAACGACAGTAATTTGTACACAACCAAGTATGTAAACAAGTTGGCAAAACGATGAATTGTTTTTTGTAACGTTAAG

>gene12757

TCGTACACGCAGACATACTTGCGACGTCTTACACGCAACACGTTTGTGACGTGAAGATTAATCTTTTTAATAATTTTGTTTATTTACTTCGAAGATTATACCAATATTAAGATAATGATCATCCCACTGATTTGGTTGGCCGTCTTGATCGGCGTGGCAGCGCTGTACTTCCGCCGGGTGTACTCCAGCTTCAGCCGACAAGGCATCAAACACTTCAAGCCAGTGCCTGTGTTCGGTAATTTGGCCAAAATGCTAATGCGCACCGAACATTTCTGCGACCATATTGTCAACCTCTACCAAAGCTTCCCTGAGGAACGGTTCGTTGGAAGGTACGAATTTGTGAAGCAGATAATTTTCGTTCGCGACCTCGAACTGATTAAGAAGATGACTGTGAAAGACTTCGAGCACTTCCTTGACCACCAATCCATATTGAGTACCAGCGACTCTTACTTCTCCAGGAACCTCTTCTCTTTACAAGGTCAGGAATGGAAGGACATGCGCTCTACACTGAGTCCAGCGTTCACAAGCTCCAAAATGCGTCTGATGTTGCCGTTCATGGTGGAAGTTGGGGATCAAATGATGCAGATGCTTCACCGGCAAATCAGGGAGTCCAAAAATGGCTCCATAACTGTGGACTGCAAAGACCTAACTACCCGCTATGCTAATGACGTGATTGCGACCTGTGCCTTTGGTCTGAAGGTAGACTCCCAGACTGACAAGACCAACAGCTTCTATATGCTTGGCAAGGAGTCTTCTGAATTCGGTTTCCGAAAACTGATGTCTTTCCTCATCGTCTCCAATGCACCGGCTTTAGCAAGGCTTTTGAAATTGGATTTCCTCTCGGAGGCATGCAAGCAAGGGTTCAAGAAAATTGTGCTCAGTACGATGCAGACCCGAGAGATGAAGAACATTATCAGACCAGACATGATTCATTTGTTAATGGAAGCTAAAAAAGGTAAACTGACCCACGATGATGTTAAAACAAATGATGTCGCCGCTGGATTTGCTACTGTTGAAGAATCAGCTGTTGGGAAGAAATCAGCAGATAGAGTATGGAGCGACGAAGATCTTGTTGCTCAAGCAGTCCTCTTCTTCATTGCTGGATTTGAAACTGTTTCAACTGGAATGATCTTCCTACTTTACGAGCTAGCTGTAAATCCTGATGTACAGGAGCGGCTGGCGCAGGAGATCAAGGAGGTTGACGCTAAGAACGGCGGCAAGTTTGACTTCAACTCCATACAGAACATGGTCTACATGGATATGGTGGTGTCAGAGACTCTCCGTCTGTGGCCACCTGCAGTCACTCTTGACAGAATTTGCACCAAGGACTACAACATGGGCAAACCTAATCCGAAAGCTGAGAAAGATGTCATTCTCCGCAAAGGCACTGGGGTCTGGATCCCCACATACGCCTTCCACCGTGATCCTCAATACTTCCCCAATCCTGACAAGTTCGACCCTGAGCGATTCTCAGAAGAAAACAGGCACACTATTAACCCGTTCGCTTACATGCCCTTCGGTGTTGGACCTAGGAATTGCATTGGGTCAAGATTTGCGCTTTGCGAGATAAAGGTTATGGTGTACCAGATCCTCAAAGAGATGGTGCTCTCCCCATCAGAGACGACCTGCATACCAGCTAAGTTATCAAAAGACACATTCAGCATGAGGCTGGAAGGAGGTCACTGGCTGAAGTTTTCCCCAAGGAATTAGAACAAAATTATACTGACCATGATAAAACTATAGACATTGGTGTAGGTTTGAAACGCGGTGTTCAAATAAATTGTAGTTTGCAATACTTTATGTTTGATATATAAGCTTTGATTAAGTACAAAATGTGAACAAAATGGAAATAAATATATTTGTTTGTTGTTTG

>gene12758

CTCAGTCTCGGAATACTGGCACGGACGCCGGTTGTTCCTTTCGTAAACATTTTGCTCTGCGATTTTACAAGGTAAGATCCATCATGCTGCTGTTACTGACGTGGGTAGTGGTGATCGTCACAGCCTTAGTGCTGTACCTTCGCCAAGCCTATGGCTCCCTACGCCGCGATGGCATCAACCATCTGCCAACCGTCCCATTCTTCGGCAACATCTTCTGGGTTATGTTCCAGAAAGAGCACTTTGTCGATCTTATAATGAAGATTACTAATGCATTCCCAGATGACAAGATAGTAGGAAACTATGACATGATGACTCCTATACTAATTATTCGAGATCTGGAGATGTTGAAGCGTATCACAGTCAAAGATTTTGAGCACTTCATAGACCGTCGCAGCTTCACCACTGAGTTGGATCCTATGTTTGGGAGAGGTCTGCTGCTCCTGCACGGTGACGAATGGAAGGCAATGCGATCTACAATGAGTCCAGCCTTCACCAGCTCCAAGATCCGGCTGATGGTTCCATTCATGGAAGAGGTTTGCTTAGAGATGGTCAAAGTCCTCAAACAGAGAATCAAAGATTCTGGAACTTCTTACTACGACATGGAGTGTAAAGAAGTTATGACGAGATACGCGAATGACGTCATTGCCTCCTGTGCCTTCGGCCTGAAAGTGGAATCTCTGACAAGAGACAGTGAGTTCTACGTGCACAGCAAGTCCATCACCAAGTTCACTTTTTGGAGGTTCATGAAGTTTATGTTCTACAGGCTTCTACCATCCGTAGCCGGGATGTTGCAACTCTCGCTGGTGTCACAGAAGACAACAGACTACTTCACAAACGTAGTCCTTGGTACAATGAAGGACAGGGAGAAGAACAAGATTGTTCGGAATGATATGATCAACATCCTGATGGAAGTAAAGAAAGGACAACTTACTCATGAGAAAGAAGGTAAAGACGCTGATGCTGGGTTTGCAACCGTTGAAGAGTCACACGTTGGAAAGAAACAGCATAATTATGAATGGACCGACACAGACCTAGTAGCCCAAGCAGCATTATTCCTCTTCGCTGGCTTCGACACAATTTCCACAGCGATGTCGTTCATACTGTACGAGCTGGCAATAAACCCTGACGTGCAGGACCGGCTGGTGCAGGAGATTAGGGAGTATGACGCCAAGAACAATGGAAAGATTGACTACAACAGTATACAGAGCATGACTTATTTGGATATGGTGGTGTCAGAGGGTATGAGACTATGGCCGCCGGCTCCCTTCATGGATAGAATATGTGTGAACGACTACAACATTGGAAGACCCAACAAAGAAGCTACGAAGGATTTAATCATTCGCAAGGGGCAGTGTGTGGTGATCCCGGCGTATACTCTCCATCGAAATCCTGAATACTTCCCGAACCCTTCAAAGTTCGACCCGGAACGGTTCTCGCACGAAAACCGGGATAAAATCGTCCCGTTCACTTATTTACCGTTCGGGTTGGGTCCTAGGAATTGTATTGGATCTCGTTTTGCCTTATGTGAGGTAAAAGTAATGCTGTACCTGCTGCTCCGTGACCTAAAACTGACCACTTGTGATAAAACCTGCATCCCTGCGCGGTTGAGCAAGAATGGATTCGAAATGCTACTCTTTGGAGGTGCCTGGGTGCGATTGTCTGTTCGCACTGAATAATAAAAATATAACAAAAATAATCTGTAAAGAATGTAAATATCTAATATTGTACGTAAGTTTTATATT

>gene12768

TCAGTTTCTAATATAGTCAAACTCAAACGTTAGTTCACTAAAAGCGCGCCAACACGTGTTACATACATTGTACTATTCCTAAAATCGATAAACAAATACGCAGCCAATATAAATGAAAAAGACTGATTCCAATTTTAAAAATCGAACGTGTTTATGATTTCTTGGAAGCCAGTGACCAGTTTTTAAATACTGTTTTGTGGAATTCGGGTTTTGTTGACGGAAAAAACAAAATAAAATATCTAATTTTGTTTATTTTGTTCTTAATAACAAAACAAAGAGATGTGGTTGTTGATCGTGATCTTAGTGACGTGCCTCTTTTATTATTCCCGCCATCGCTTGAGTTACTTCTCGTCCCGGGGTGTACGTACGTTGCCTCCGGTCCCGTTTTTGGGGAATTTGACAGCTGTCACTCTTGGGCGGGAAAATTTCGTGGAAGCGATCGCGGCGGGATACAATGCCTTCAAAGATCAAAGATACTTCGGCTTGTACCAGTACTTGGTGCCGACCCTCATACCTCGGGATCCGGAGCTGATCCGTCAGATCATGGTGCGTGACTTCAACTCGTTCATCGACCGAGGAGTACACATCGATGCTGACTGCGATCCACTGTTCGGGAGAAACCTCATTATGTTGACTGGATCAAAATGGCGATCAATGCGTGCCTCCTTATCTCCCGCGTTTTCTGGAGCTCGCTGCCGCAGTATGGTGCCGCTCATGTCCGAGAGCGCGTGCGCAGTCCTCAACTATTTACAGGAAAAGATAACTGGCGAACAAGTCATCGATATTAATACTATAACAATGTCCTATGTTAACGACGTGATAGCATCATGCGCGTTCGGCTTCGCGTCTGATTCTCTCAAGGATCCCAACAACTGCATCTTTAGACTAGGGAAGAAGGCAGTCATACAGGACACTACGCAAGTCATGAAGTTCTTCGGCTACGAGAATATGAAGAGCATTATGAAGTTTCTAGGAGTTAAATTAATTCCCACGCAAGACGCGGAACAGTTTTCTCAGCTATTCAAATCTGCACTAAAGGCTCGTAGAGAAAACTTAGTGAAACCTAGACCTGACTTCATTCAAGTTCTAGTCGACGCTGCTAATGGGAAACTGAAGGCTGATTGTGAGAATGACGGCAACAACAACGAATGCGCAAAGACCGCTGAGAAATTCACAGACGACGATCTAGTAGCTCAAGCAGTTCTCTTCTACATAGCTGGTTACGACACCACAGCAAACCTGATCTACTACTTCTTGTATGAAATGGCCATCAACCCTGCAGTGCAGGAGAAGCTTCATGAAGAACTGGACACCTTGCCTCCTGAAGACCAGATCAACGATATGTATGAAGCGGTTCAAGAACTAGAGTATTTGGACATGTGTGTGAATGAGGTTTTGCGATTATGGCCTTTAGTTGGGTCCGCTGATCGGCGTTCGGTCGCTACTTACGACTTTGGTCCTACTCACCCTGGAAGCAAAGATCGTCTTGTCGCGCCAGCAGGTATACACGTCTGGCTACCAATATACTCGATACATCGCGACGAGAAGTTCTGGCCAAATCCAGATGTCTGTATTCCAGAACGTTTCTCAAAGGAGAGGAAAGCAGAGATAGTTCCCTATACGTACATGCCATTCGGAAATGGACCCCGACATTGCATTGGTTCCCGCTTCGCAGTTCTAGCAGCGAAAGTGTTCCTCGTGAAATTCCTGAAGCATTACAAGACGAAGGCAATACGAGCATCACCACATCTCTCTCCACGCGCTTTTATTCTAAGACCCAAAGACGGATACCAACTTATTGTAGCACCTAGGACCTAGACATTAAGTACAAATTACAATAAATACATTCATAAATATACATTAAATTATTTGTTCG

>gene13428

GACGTATGTCCGAGGTAGGGTAGTTAGTGCTCTGTATTGTGTTAGGAACGACGCTTTGGAAGAATGTATTGGCCGCTGTACTTAATAGGCGGTGCACTGGTAGCCTTCTGGTTGCTATGGAGATACAAGAACAGGAGGATGATACAGATGGGCAATAAGATGCCAGGTCCACCAACGATACCACTCTTGGGGAATGCGCTGATGTTCATGTGCCAGCCAGAAGCTTTTATCAAAGTACTAAAGGATTTGATGGCAGAATACGGGAATGTGACGAGATTCTGGCTCGGTCCTGATCTGAACATTGTCATCAGTAATCCTGATGACATAAAGCTTCTACTGTCAAATACAAAGACCAGTATCAAAGGGCCGCAATACAAGTACATGGCTGATGTGCTTGGAGGCGGAATACTGAGTGGATCAGGCCCAACATGGAGACGACATAGAAAGATAGCGAACCCCAACTACGGTAAGAGAGCCATTGAAGGATACAGCGAGGTGTTCAACCAAGAAGTGGATCTCTTGCTGAACAAGTTGAGGTCTATGCCGAGAGAGCAGTTCAATGTCTACGAACATGTTGTTAGGACCACAAGCTATGCAGTTTGTCGAACACTGATGGGTTTGACAAAAGACCAGACGATGAAGTTACCACACATTGAAGAACTGATAGACACGAGTCCATGCCTTTACGACATAGTATTTCGTAGGATGACAAAATGGTACCTGCAAATCGATCCCGTTTTCTGGATGACCAGTGACCATTACTCCCAGAAAAACTTTGTAAAAATGATGACGGACTTTTGTGAAGTCATTCTGCAGCATAGGACGGAAAGGCTGAAGACTTTGGAGGACAGTAAGAGGGATCTGATGAACTCTGAAGATGATTCAAGTGTTAATACTGAACTCAGTGTTATCGATAGGTTCCTACTCTCACAGGAGCTTGATAAAGATGAGCTGGTGCAAGAGACCTTTACTATATTTACTTCGAGTCAAGAGGCTACTGCAAAAATATCGTCTTACATCCTCCTTATGATGGCTTATCACCCAAAATGTCAGGAGAAACTTTTTGCAGAAATAAAGAATATTATCGGTGATGAGGACAGATATGTCACTGATGATGATTTGAAACGAATGCCGTACTTAGAGATGGTGTTCAAAGAAGTGCTGAGACTGTACCCCATAGGGGCTATGTTGCAGAGAACTATTAACGAAGATATTGCTATTAGTAACTACACACTACCCGCTGGTTGTTCTTTCGTGGCGCCAATATTCCACTTACACAGGGATCCCAGATTCTGGACTGAACCAGATCGCTTCGACCCAGAGCGGTTCAACCCTGAAAATGTGAAAACACGCAACCCTAATGCTTACATACCATTCAGTTTGGGACAGATGGACTGCTTAGGACGATTCTTCGGGACTAAGTTAGTGAAAACACTTTGTGTGCGTGTACTAAGAGAATTTGAACTTTCTTCAACTGAAACTTACGATGATCTACGTGTGATTATATCTATTTCTGTCGCATCATTGAATGGATTTCCTATCATCTTGAATCCAAGAAAAAAATGAACCTAACTTTATTGTCATAGCAATATGTATACCAGGTTTTATTTATAAAATAAATGATAATGTT

>gene13430

ATGGACTGGCCACTATTGTTCCTGTACATAATAGGCGGTGCACTGGTAGCATTCTGGTTGCTATGGAGATACAAGAACAGGAGGATGATACAGATGGCGAACAAGCTGCCAGGCCCGCCCACGCTGCCACTATTAGGAAATGCGCTCGTGTTCATGAACCGGCCAGAAGAGATACTGAACAAAATAGGAGAACTCGTGGAGACGTACGGTGATGTTTTTAGGTTCTGGCTGGGACCTGAACTTAATATTGTTGTTAAAAACCCAACAGATATCCGTGTTTTATTGTCAAGTACCAAATTGAATCAGAAAGGGCCAGTATATGATTTCATTTTGCCATTCCTCGGTGGTGGCATTATAACTGGAGGACCGACCTGGCGCTTGCACAGGAAGATAACGATTCCATCGTACAGCAAGAAGACCGTGGAGTACTTCTTTCCCGTCTTCAACAAGGAGTGTGAGGAGCTGGCCAAAGTCATCTGCCAGAAGGGTCCGACGACCTTCGATTGCTACGAAGATGTCCTCAGAAGCACCACGCAGAGCGTTAACCAAACGGTGATGGGGCTATCTAAAGAAGATTCAGTAAACTTAACCAGATTGGAAGAATTTATATTTAAAACTCACGACATGTATAATCTTATGTTTGACAAAATGACGAAATGGTGGTTGCATGTGCCGCCTATTTACTGGTTGCTTGGCAAGAAGAAGCAACAGGATTACTACTTGAAGATGATAGATGACTTGACGGAAGATATCGTGACACGGAGGAGGAAAGCCTTGGAAGTGTCCCAGCCGAGCGAGGAGTGCATGGGTGTTGTGGACCGATTAATCCTCTCTGGAGAGCTCACTCACAAGGAGATCAAGGAGGAGACCATCACATTATTTACTAGTAGTCAAGAGGTAGCCGCTAAAATAGCAGCGGGAGTTCTCATGTTCCTAGCTCACTTGCCGGATTGGCAGGATAAAGTATACAAGGAGATAATAGAAGTAGTCGGAGCTGACGGTCCAGTCACAGATGAGCAGCTGAAGCAGCTGGAGCAGCTGGACATGGTGTACAAGGAGACATTGCGGTACTTCCCAATAGCAGGGATGATACAGAGGACTGTGACGGAAGATATTACTATTAGAGATGGAAGCATCAGCGTCCCAGCTGGCACATCGCTGGTGATACCGTTCCACAACCTCCACCGCGACCCTCGCTACTGGGAGGACCCCAATAAAGTGATGCCGGAGAGGTTCCTCCCAGAGAACGTGAAGAAACGAGACCCTAACGCCTTTCTACCTTTTGGTTTGGGACCTATGGACTGTTTAGGTCGAGTTTATGGAACGGCCCTCATAAAAACGATAGTGGTGTGGGTGTTGCGGTACGCTAAGTTGGAACCCGCCATCTCCCTCGACAATATAAGGCTGAATATCGCTATCTCTGTCACCAGCTACGATGGTTATAACATTAAAGCCAGTCCTAGAAACGGGAGAATAAATGGACTATCATAAAAGGTCGTCAATGTAATTTGTGTGAATGTTAGAATGTCACTGACATATAGTTCTTAAAATCTGCATAGTGAAGTGATAATGGACAAGCTACGTCTCTCGAAGTACTGACGGGTGAGGGGCAAGGAAATTGTTCGAGCGGCATCTGTGGACTGGAAGGCGCAACGTTGATAGACCCGCTAAGTGGATTAACGACATTATCAGTGTTAATATTGAATCCAAGTGGTGGTTCGTAGTAAGGATCGTATGGTTTTAAGCTGCCTGATAATTTATTTATGTATTTAATTTAATATGCTTCGTGCACTCGATGTACAATAGGTGGACTTAATGCCGCAGGATTTTTCTCCTAGTCTGACCTGATAATTCGAGTATTTATGTGACCATTTCAAAATTCTTTAGTAGCTGTGATTGTAAACTGAGACAAGCTTGCCTGCCAATACAGCAGGGCCTCATGTCTGCTATAATTATAATTGTTGTCATTCCAACACAATCGTATTGTCATTTTGCTATAAGTTCCTAATATGCTAAATGCATAAAATTGGATGATTGACAGGTCAATGGTCTCGTAATGGAAATGTAGTAGAATGACATTGCCAATTTGCCATAATGCCATTATTATAAAACTTTGTAGTAGAAATAGTTTTCGAAAAACTACTAGGTACTAAATTTGAGCATTTGTTCATGCCAGATGTTATTTATGTTTTATTTTACAAAATATCTATCAGTATATTATTATGTTTTCGGCTTACTCACGTT

>gene13432

ATGGACTGGCCACTATTGTTGCTGTACTTAATTGGCGGTGCACTGGTAGCGTTCTGGTTGCTATGGCGTTACAAGAACAGGAGGATGATACAGATGGCGAACAAGCTGCCAGGCCCGCCCACGCTGCCACTATTAGGAAATGCGCTCGTATTCATGAACCGGCCAGAAGAGATACTGAACAAAATAGGAGAACTCGTGGAGACGTACGGTGATGTTTTTAGGTTCTGGCTGGGACCTGAACTTAATATTGTTGTGAAAAACCCAACAGATATCCGTGTTTTATTGTCAAGTACCAAAGTGAATCAGAAAGGGCCAGTATATGATTTCATGGTGCCATATATCGGTAGTGGCATTATAACAGGAGGACCGACCTGGCGCTTGCATAGGAAGATAGCGATTCCTTCGTACAGCAAGAAGACCGTGGAGAACTTCTTTCCCGTCTTCAACAAGGAGTGTGAGGAGCTGGCCAAAGTCATCTGCCAAAAAGGTCCGGCGACCTTCGATTGTTACAAAGATGTCCTCAGAAGTACCACGCAGAGCGTTAACCAAACGCTGATGGGGCTATCAAAAAAAGATTCAGTAAACCTAACCAGATTGGAAGAATTTATATTTAAAACTCATGACATGTATAATCTAGTCTTCGACAAAATGACAAAATGGTGGTTACATGTGTCGCCTATTTACTGGTGGCTTGGCAAGAAGAAGCAACAGGATTACTACTCGAAGATGATAGATGACTTGACGGAAGATATCGTGACACGGAGGAGGAAAGCCTTGGAAGTGTCCCAGCCGAGCGAGGAGTGCATGGGTGTTGTGGACCGATTAATCCTCTCTGGAGAGCTCACTCACAAGGAGATCAAGGAGGAGACCATCACATTATTTACTAGTAGTCAAGAGGTAGCCGCTAAAATAGCAGCGGGAGTTCTCATGTTCCTAGCTCACTTGCCGGATTGGCAGGATAAAGTATACAAGGAGATAATAGAAGTAGTCGGAGCTGACGGTCCAGTCACAGATGAGCAGCTGAAGCAGCTGGAGCAGCTGGACATGGTGTACAAGGAGACATTGCGCTACTTCCCAATAGGAGGGATGATACAGAGGACTGTGATGGAAGATATTTCTATTAGAGATGGAAGTATCACCCTCCCAGCTGGTACATCGCTGGTGATACCGATCCACAACCTCCACCGCGACCCTCGCTACTGGGAGGACCCCAATAAAGTGATGCCGGAGAGGTTCCTCCCAGAGAACGTGAAGAAACGAGACCCTAACGCCTTTGTACCTTTTAGTTTGGGACCTATGGACTGTTTAGGTCGAGTTTATGGAACGGCCCTCATAAAAACGATAGTGGTGTGGGTGTTGCGGTACGCTAAGTTGGAACCCGCCATCTCCCTCGACAATATAAGGCTGAATATCGCAATCTCTGTCACCAGCTACGATGGTTATAACATTAAAGCCAGTCCTAGAAACGGGAGAATAAATGGACTATCATAAAAGGTCGTCAATGTAATTTGTGTGAATGATAGAATGTCACTGACATATAGTTCTTAAA

>gene13433

TGTAGATATTTATCAAGTAAATAAATGTTATTGTTGTTGTTCAGGTCTATGTGTTGATGCAATAAATTAAGAATGAACAGAAGTCATTAAAATGGCATAGGTAGTATAATTAAAGTGTTAGTGTTGGAATTGTGTAATCAAGGGTGACAATTTTAAAATAACCAGCCATGTTCTGGCTAACCATCACCCTGATCATCACAGCCGGATTCTTAGGTTGGCAGTACTGGAGATGGAGACACAGACGTATGCTGAAACTTGCAGCGAGATTGCCTGGTCCACCAGCATTGCCCATACTTGGGAATGCCCTCAGTTTCATGTTCAATCCTGGAGAGTTCCTGAACAAAGTCGACGTATTCATGGAGAAGTATGGTGAAGTCTTCAGGTTCTGGCTGGGTCCAGAACTCAATATTGTTGTGAAAAACCCAACAGATATACGTGCCTTACTATCCAGTAACAAAGTGAACCAGAAAGGACCATTGTATGAGTTCCTGGTACCTTTCATCGGCTATGGCATTTTATCTGGAGGTCCAATTTGGCGCGCTCACAGAAAAATAGTGACTCCTTCGTACAACAAGAAGTGTGTGGATAACTTCTCTCCAATCTTCAACAGGGAGGCTGAGCAGCTGGCCAGGGTTATCTGCAAGAAAGACCCGAAGAGAAGTTTTGATATCTACAAAGATGTCGTCAGATGTACCACGCAGAGTGTTAACCAAACACTGATGGGACTATCTAAAGAGGATTCCCAAAACCTGTACAGAATGGAAGAGATGTTATGGGCCACGCAAAAAATGTACGGTCTAATCTTCGAGAAGATGACAAAATGGTGGTTACACATACCGATTATTTACTGGCTGCTTGGCAAGAAGAAGCAGCAGGACTACTACGTGAAGTTGATAGATGACTTGATGGAAGACATCGTGAGACGGAGGAGGAAAGCCTTGGAGGTATCCGTGCCTGGTGAGGAGTATATGGGTATCGTGGACAGATACATCCTTTCTGGAGAACTTACCGAGAAGGAGATTCAGAGGGAGACCATGACTCTGTTTACTACGAGCCAAGAAGCAGCTGCTAAGATGGCATCAGGAGTGATCATGTTCCTAGCTCATTTGCCGGATTGGCAGGATAAAGTGTACAAGGAGATGATGGAAGTGGTCGGAGCTGACGGTCCAGTCACCAACGAGCAGTTGAGGCAGCTGGAGTACCTGGACATGGTGTACAAGGAGACACTGCGGTATTTCTCCATAGCTGCCCTGATACAGAGGACTGTAGTGGAAGAAATTACTATTCAAGACGGTAGTATAACCCTCCCAGTGGGCACATCGCTGGTGATACCGATCCACAACCTCCATCGCGACCCTCGTTTTTGGGAGGATCCTCACAGGGTGATGCCGGAGAGGTTTCTCCCGGAGAACGTGAAGAAACGAGACCCTAACGCCTTCGTACCTTTCAGTTTGGGACCTATGGACTGTTTAGGTCGAGTATACGCAACAGCTCTCATCAAGACGATAGTGGTGTGGGTGCTCCGGTACGCAAGGTTGGAGCCAGCTGGGAACCTTGACAATATAAAGCTCAACGTCGCCATCTCCGTGTCCTGCGCTGATGGATATAATATTAAGGCTAGACCTAGGAACGGTAGAATAAATGAATTAACATGA

>gene13786

TGAAGTGTCAACAGTCGACAATAACACGTTAGCCGAAATGAACGGGCTTATCGTTCTCGCGTCATTACTATTCGTAATATTTTCTTTAATTTATTACATATCGAGTAAAAAACTCAGCTACTGGAGAAAAAAGAATGTGCCACACGTCGACCCATTGCCTATTTTGGGCAATTATGGGGACTACATTTTGCAAAAAGAATACCCGGGAAAGGTGGTCCAGAAATTGTGCCACAAGTTTAAGAATGAACCATACTTTGGAGCTTACTTCGGCACATTGCCCGTCCTAGTAGTGAAAGACCCCGAGATCATCAAACATGTGTTAACAAAGGACTTCTACTACGTCAGCGGCAGGGAATCTTCCCATTATTATGATCAAGAGGTTATGACTCAAAACATGTTCTTCGGTTCAGGCGACCGCTGGAAAGTATTGAGACAAAATCTAACGCCTCTCTTCTCTTCACTAAAGATGAAAAATATGTTTTATTTGATTGAACAGTGCACAATCGGCTTTGAAGACATGCTGAACAAGGAACTTAAAATGTCTAATGTCGTTGAAGTAAGAAGTGCAGTTGCAAAGTATACAATGGACAGTATTTGTTCCTGTGCTTTTGGAGTAGAGGCTCACACGATGACAGAAAAAGAAAACAACCCCTTCCAATTCATGGCTAACGAAATATTCGAGTCATCGAAATATCGGGGCTTCAAAATTATTTGTAGAGCTGCATGGCCATCCATTTTCTATGGTCTTGGATTCAAATTATTCCCTAAGACTATTGACAGATTCTTCTCCAACCTATTGACCGGTGTCTTCGAAAGCCGAAACTACAAGCCATCGCCAAGGAATGATTTTGTCGATCTCCTCTTGAATTTTAAGACAGAGGATTATATAGTTGGGGATAGTTTAAGTAATGTGAAAACAGGGGAAGAGAAGAAGGTGAAACTGAAAGTAAGTGACGAGGTGCTGATTGCGCAGAGTGTTATGTTCTTCTCTGCTGGTTTCGAGACGTCGGCGACGACCACGAGTTTTACTTTGTTTGAGTTAGCAAAACACCCAGAGCTGCAGGAACGAGCTGCTGCGGAGGTGGACGAGTTTTTACGTCGTCACAACAACAGACTGGTGTATGATTGTGTGACGGAGCTGCCTTACTTGGAGGCTTGTATGTACGAGGCCCTGCGGATGTACCCAGTGATAGGCAACTTGACCCGTGAAGTCATGGATGACTACGTTCTGCCTACAGGCTTAAAGTTAGACGCAGGTGTCCGCGTCCATATACCAGTGTATGACATGCATTATAATCCCGAATACTTCCCTGAACCTAAAATGTACAACCCGGAACGTTTCATGCCTGGAAATAAGGAGCACATCAACCCTAATACGTTCTTTGCATTCGGATCTGGACCCAGACTGTGTATAGGAATGCGCTTCGCAAAGATGCAAGTATTGTCAGGTCTCATCACCTTCCTGAAGAAGTACAAAGTGGAATTGGCAGACGGAATGTCAAGAGAGTTAGAGTTTGACCCCAAGACATTCTTGACCCAACCTCTGGACAACTGCATCGCATTAAAATTGACTGAACGTGAAGGATGGCAGCAAAGGAATTACTTAAGAACTTAATTTGTATGTAGACGATAACTACCAAATAATAAATTGCTAACACGTTTGTAGTTTATAGTAA

>gene13787

ACGAAGTACTCACAATTATCACACGCCTCCAAACATGATCACGCTGTATCTGTTGCTCGCCATACCACTGGCTCTGTACGGGGTTTACCTCGTATCAAAACGAAAATTCCGGTACTGGGAACAGAAGAAAGTGCCACACCTACCACCGAAACCAATTCTGGGTAATTTCTCCGAATACATTCTCCAGCAGAAATACTATGGACATGTGGAACAGGAAATCTGCGACAAGTTCCCTGAAGAACCTTATGTTGGCTCTTACTTTGGCACGGAACCGGCCCTCATCGTACAAGATCCTGAATTCATCAAGACTGTCATGACAAAGGACTACTATTTCTTCAGTGGCCGTGAAGCTTCTGCATACAGTAAAAATGAACCGTTAACGCAAAACCTTTTCTTCACTTATGGTGACAGGTGGAAGGTACTGCGTCAGAATCTTACGCCTTTGTTCTCATCCGCCAAGATGAAGAACATGTTCCACTTGATCGAGAAATGTGCCCGTATCTTTGAGAACATGATCGATCATGATGTACAGAAATATAAAGATATTGAAGTCCGAACCCTGACAGCAAAGTTCACTATGGATGCCATTGGAAATTGTGCTTTTGGAGTTGAGACCTGGACCATGGTAAAGACTGAAAATAATCCATTCACAAAAATTGGTGATGTTATCTTCGATACTGCCAGGCTGAGAGCATTAAAGGTTGTGATGAGAAGTATTTGGCCAGCCATTTTTTATGGTTGTGGAGGTAAATCACTCCCCGCTGACGTTGATAACTTTTTCTACAATCTGATGACTGGTATTTTCAAAGGACGCAACTACAAGCCGACACCAAGGAACGACTTTGTTGACCTCCTATTAAAGTTCTACAATAATAAAACGGTGACTGGAGATAGTATGAAAAACATGAAGGGTGATTCAGAAGAGAAAGTTACTTTAAAAGTGGACGATGAATTTTTGATTGGACAATGTTTCCTGTTCTTTGCTGCCGGGTATGAGACGTCAGCAACTACAATGAGTTACACTTTATATGAGTTGGCAAAGAATCCGAAAGCTCAAGAGTTAGCGATTCAAGATGTGGATAATTACTTACGTCGCAATGATAACGTCTTGAAGTACGAGTGTGTTACCGAGTTGCCTTACGTGGAAGCTTGTGTTGATGAGGCTCTCCGTCTATACCCACTGCTGGGAGTCCTGACTCGGGAGGTCATTGAGGACTATACATTCTCGACAGGATTGAAATTGGAAAAAGGACTCCGCGTACATTTACCGGTGTACCACATGCAACACAACCCTAAACACTTCCCGGAACCAGAACAATATCGACCGGAGCGGTTCCTGGGTGAGGAGAAGAATAACATCACGCCGTACACATACTTCCCATTCGGCGAAGGACCTCGACTATGTATCGGAATGAGGTTCGCAAAGATGCAGATAACAGCAGGAATTATAACGATACTCAAGAAATATCGCGTCGAACTTGCACCGGGAATGAGCGAGAAACTACAGTTCGAACCGCGGTCTATAATCACTGCACCTATTGGAGGTATCAGACTGAAATTCATTGAAAGAGAAGGATGGCAACAACGAGTGTTTAAGGCGCCAAGTGAAGCTTAAGCTATAATTAAGTAAGGCCAATATGTCATAGGCCTAAGTGTGAATAAAATGGTGTACTAAGACAAGTACATAAGAACTCTGTTCATGTCAATATACGGCTCGGCTGAATAACGTAACTGTTTGACTAGTTTAAAGCTACTAAAGCCAAATTGGTTAGATGTAGTAAAGTGCACGCCTGATTATGAGCCCCTGGCGAGCTCGAAATTAGTCCAGTTAACTCGTCGAGTTGTTACGTGAGTAAGCTTTAGTATGTTTTTCTACCTCTATAATCATTACTTCATTACATACCAGAAAATATGATAATAAAAAAGCATTATAAAAATATAGTGATTGAAACATTGTGATATTGAAACGTTATTTTTAAGTTGTATAATTGTATATTGGTAGTTTTTTATATTGTTTTTGTTGTGACAC

>gene13788

CGCCGGCAGTTGCTAACAACACCGTAACCGAAATGAACGGCCTCATCGTTCTCGCGTCGCTAGTAGTCGTAATATTTTCTTTAATTTATTATCTTTCGAGAAGAAAACTCAGCTATTGGAAAAAAAAGAATGTGCCGCACGCTGACCCATTGCCAATTTTGGGCAACTACGGGAAATATATTTTACAAAAAGAATACCCGGGAAAAATGATCCAGAAATTGTGTCAGAAGTTTAAAAGTGAACCCTATTTTGGAGCCTACTACGGTACGGAGCCTGTCCTAATAGTTCAAGACCCCGAGATCATCAAGCATGTCTTAACAAAGGACTTCTACTACGCTAACGGTAGGGAATCGTCCAACTATTCCAATGGAGAAGTCATCACACAGAACTTGTTCTTCAGCGCAGGTGACAACTGGAAAATAGTTCGACAAAACTTGACTCCACTCTTCTCCTCTCTTAAGATGAAGAATATGTTCCATTTAATTGAAAAGTGTACAATCGGCTTCGAAGATATGCTGGACGAAGAAATTAAAATGTCGAATGTCATTGAAGTGAGAAGGACAGCCGCAAGGTATACTATGGACTGCATATGTTCCTGTGCTTTCGGAGTGGAGTCTAACACAATGTCACAAAAAGAAAACAACCCCTTCCTACTCATGGCTGACGAAATATTCGAGTCTTCGAGATATCGGGGCTTCAAACTTATTTTTAGAGCTGTATGGCCGTCAATATTCTACCGTATCGGGCTTAAAATATTTCCCTCGACTATTAATAGATTCTTCTTCAAGTTATTGATTGGTGTTTTTGAAAGCCGAGAATACAATCCATCATCAAGGAACGACTTCGTGGATCTGCTCCTGAATCTGAAGAAGGAAGATTATATAACTGGAGACAGTATAAGCAATATGAAGACAGGTGATGATAAGAAGGTGCATTTGAAGGTGGATGACGAGCTTCTGGTGTCGCAATGTATTCTATTTTTCTCTGCCGGCTTCGAGACTTCGGCGACAACTATGGGATTTACTTTGTATGAGCTAGCTAAGCACCCGGAGGTGCAGGAAAAAGCTGCTGAGGAGGTGGATGCGTTCATGCATCGTCACAAGAACAAGCTAGTGTATGATTGTGTGACGGAGCTGCCTTACTTGGAGGCTTGTATGTATGAGACTCTGCGGAAGTACCCTGTGCTAGGCAACTTGACCCGCGAGGTCATGGATGATTACGTTCTGCCTACGGGGTTGAAGTTAGATATAGGTGTCCGTCTGCATATCCCAGTGTATCATATGCACTACAATCCCGAATACTTCCCTGAACCTTACAAGTACAAGCCGGAGCGATTCCTACCCGAACACAAGAATGAGGTCAACCCTAACACGTTCTTTCCATTCGGATCTGGACCCAGGCTGTGTATAGGAATGCGCTTCGCAAAGATGCAAGTATTGTCAGGTCTCATCACCTTCCTGAAGAAGTACAAAGTGGAATTGGCAGAGGGAATGGCAACAGAGTTAGAGCTTGACCCCAAGACGATATTGACCCAACCTCTGTACAATTGTATTGGATTAAAATTGACTGAACGTGAAGGATGGGAGCAAAGAAGATTTGTACGAACGTAATTTGTACGTATTTATCAATAAGGCTTAGGTCTCACTACGGCTCAACCGCGGTCTCTTAATATGTACCTAGTGCAGCCATTTTTAAACCGTGCATGTAAGTTAAACATGAAATAGGATACAAGTGGCCGAAGTTCGACTAAATTTTAAATGGATATATTACTGCATAATAACTCATATAACATAACATAACATCACGCCTGCCTGACTGCCTCGTTGACCGAGTGG

>gene1390

GCCGTTCCCACCGGTGTTGTCACTCTAAAAAATCTTTTTTTTTAAATAAAGATGTTGAGTGTACTATTTTTCATAACCGTCGCCATCTTGGTGGCGTATAAATTTTATTCTAAGAACACCGTTACTTATGAAAAAGTAAATAAATACGGAGAAAATAAAGTTATGGTTTTGAAAGAGGCGCCGGGGCCTACGCCATTACCGATTATCGGTAATTTACATCTGTTGGGCAAGCATGAATCCCCATTTCAATCTTTCACCGATCTTGCGAAAGACTATGGGGATATCTTTAGCTTGAAACTGGGAACAGCGAAATGTTTAGTAGTCAACAATTTGGAGTTAATACGCGAAGTTCTGAATCAAAATGGGAAATTTTTCGGCGGTCGACCCGACTTCCTACGCTTCCATCAACTCTTTGCCGGAGACAGGAATAATTCACTAGCCCTGTGCGACTGGTCGAACCTGCAGCTCCGCAGAAGAAACCTAGCTCGTCGCCACTGCGGTCCCAAGCAACACACTGACAACTACGCCCGCATCGGTGACGTCGCCACCTTCGAGTCCGTCGAACTAATTCAAACCCTTAAAGGTATAACCAGAAACTCCGACGAAGCTATCAACTTAAAACCGATCTTAATGACGACAGCAATGAACATGTTCTCCCACTACATGTGCAACGTTAGATTTGACGCGGAAACCGATAATGATTTTAGAAAAGTCGTCGACCATTTCGACGAAATCTTCTGGGAAATCAACCAGGGATACGCCTTGGATTTCCTGCCTTGGCTCAAGCCGTTCTACAAGAAGCACTTGGACAAACTCTCAAACTGGTCTTCTGACATCCGTTCCTTCATTCTATCAAGGATTGTCGAACAGAGGGAGATGAATTTGGACATCGAAGGGCCTGAAAAGGACTTTTTGGACGGTCTCCTCAAAGTTTTGCACGAAGACCCGACGGTCGACAGGAACACCATCATATTCATGCTTGAAGACTTCCTTGGCGGTCACTCTTCTGTTGGTAACCTTGTTATGTTATGTCTCACAGCTGTAGCCAGAGACCCTGAAGTAGGCAGGAAGATCAGAGCAGAATTAGACGCTTTGACTAAAGGCAAACGACCTGTCACTCTCCTAGACAGACACAGCATACCATACACAGAAGCTACGGTTCTAGAATGCCTAAGATACGCTTCATCACCGATCGTACCTCATGTAGCTACAGAAAACGCAACCGTTGCTGGTTATGGAGTCGAAAAAGGTACAATAGTCTTCATAAACAACTACGAACTGAATACGTCAGAAGAATACTGGGAAAGGCCAGAGGAATTTGATCCTTCAAGATTCTTAGAGAAGACTAAAGTCAGAGTGCGCAGGAATTCTTTATGTGATTCTGGAATGGAGTCGGACGGCGAGAGACCTTCGAAGCAAGCTGAAGCTAACGTGGAGAAAGAGGTATGGTCTGTTAAAAGAAACATCCCTCATTTCTTGCCATTCAGTATTGGAAAGAGAACCTGCATTGGTCAGACTCTAGTTACAACAATGAGTTTTGTAATGTTTGCGAACATTATGCAGGAATTTGAAGTCGGGGCTGAGAGTCTTGAAGATTTGCGTCAAAAACCGGCTTGTGTAGCGCTACCTAAAGATACGTACAATTTGTATTTGATACCACGTAAATATTGATTTGGTTTTTGAAATTATTTTGGGCTTGATTTCTTTTTTAGGTTCTTTTTAGTTTGAGAATCTTATGTCTATTAAATTCCTAAAAAAAATCGAGGTAGAAATGAAGGGAACGTTTCCACTTCAAAAATTAATTTCTTCAGTACAATTTTTCGATTGTAAGAGGTTTTTAACGTAGTTGTTTTTCGTATTAACTTTTTCGCTATAGATTCTCTTTCTAAGAAAATAAACAAAAAAGAATCAAGAAGGGTTCGCTCAAAAGTTAATTTATTTTTTCGAAATAATACGATTACTTACTTGTCTTAAATAAGTTATTTTTTAACATATTTTTCGGTATTAATATTGACATTACGTGGGAATTACAAAATTATTCAAATAGTACAATTTAATTTTAAAAAATACGTAGAATTGAGACCAAAAATGACGATTGCGTGATTGTGGAAACAATACATTGCCTTTAAATTATTATTAAGTCTTTTGGAATGAAAACAAAACTTGAAATATTAATAATTATGTAGAAAAAGAAAACGTGAGGAATTTTTTAATCAAAATATGCTGATACATTGACTAAAAATGCTAGTAGTTAAAATCAAATGTTTATTAAAAAAAAATAGTGACGTAAAAAATAGAGGTAGATTTTGGTAGCAATGGTATCTTTTGCGATAATTCTTTCTTTTAAATTATTTTTTTACGAGTTGTACTTACCTAGGTATACATAGTTTGATTAACAAGATCCTTACGTTTGTACTATTTAGCTTAAATGAAGTAATTATCTAAAAATTGTAACCATAATGACAAAATTAGTTCCTTTAAAAAAATATTTTTTTCATAATGTTACAATAACAATTAGGATTTTATTTTTAATGGCAATAATTGGGGTATAAATAAAAAATAAACGACTGTATCATGCTTGGTGGACTAATGCTTGTATTTAGTAGAAAAAAAATGTTTCTGATGACGAAATAATGCTAAAAAAATGGCAATCCATTTGAAAACGCAATTTTTGAATCACTGAAACATTTTTTGTTTAGAATTGTAGTATTGTAGTTTTCATTAAACGTAGTGAATTGATCATCTCTTAGAATTCCTTTTAAATACATATACGAATATAATAAACTCATCAACATTGGCTGTTTTAAAAAATAATGAAACCGAGAGCGCAGTTAGACAGTTGACCGTAGCGACATCTAGTAGGGAGTAGACAAAACTTTTCTTTTATGTAATTTATAATTTACTTCGCTCTTCGGTTAATTTGGATAAATGAATGTTTGATTTTGTATTAACTTCTAGAACTGGTATAATCATTAGAAATAGTAATTAAAATATAGACTTAAACGGGGGAAAATTATGTAAAACACTCTTATTAAGAATTAGATTTTTTTA

>gene14070

TTGCCGAGTAGGCCAGTGGATATTTTTACCTCACGCGTACTATACACTGTGCTCTCTGTAAAAATATCGATCATCGAAAATGATCCCTACGTTTCTACTACTGTTTGTGGTCACGTTGATATTGTACGGCTTTATCTCGACTGTAAAGCCGAGAAAGTATCCGCCGGGTCCAGTATGGTTTCCATTTATTGGAAGCAGTGGTATTTTGCAGAAGATGACAAAGAAGTATGGATCGGAATGGAGAGCTTGTTTAGAACTATCCAGACAGTATTCCACGAATGTTCTAGGCATGAGATTGAGCACTGAACTATTAGTAGTCGTTTTTGGTGAAAAGAATGTACGTCAAGTATTCAATGAGAAAGAATTTGATGATAGGCCTGATAATTTCTTTGCGAGGTTACGTTGTCTTGGATACAAGAACAAGGGTATAACTTTTGCTAACGGTGAAGTGTGGAAGGAGCACAGACAGTTTGCAGTGAAAAACTTAAAATACGTGGGCTACGGTAAAACTGTAATGGAAAAAGAAATACAAAATGAGCTGTCCAGTTTATTGGAGCAAATAAAGGAGAATAACGACAAACCAATTAATATAGTTAATTTATTGGGCGAATCTGTGATTAACGTTTTATGGAAATTTGTGGCAGGCGAACGTATAAAAGAAGAGAAACTGAAACATTTATTGGATCTTTTTAGGCAGCGAGGGAAGGCTTTCTCAGTTGCCGGGGGCATGTTAAATCAAATGCCTTGGTGTAGATTCATTATACCTGAATTGAGTGGGTACTCACTTATAAAAAAATTGAACGGAGAAATTTCCAAAGTTATAGAGGAAGCTATTGAAAAACATAAAAAGAAAGAAGTAGAAGGTCATGATTTTATCTACAAGTTTCTGAATGAAATTGATGCGAATAAAAACGCGACATTTACAGAGGAACAATTGAAAATAGTATGCTTGGATTTCTTTATTGCGGGGTCACAGACTAGTAGTAACTTCCTGGGCTTCGCTTTTCTCAAGGTGTTAAAATCTCAAGACATTCAGGAGAAAATTTTTAACGAAATAGACACAGTAATTGGGGATAGACCACCATGTTGGAATGACAATGAAAGATTGATATACACGTCTGCATTCATTCAAGAAGTTCATAGATACTACCCTGTAGTTTGCATGGCCGGTCCTAGGTCCCTGCAAGCAGATACATCTATTGACGGGTATCGTATCCCTAAAGGTGCCACGGTACTCATGTCTTTGGCCGATATATACTTTGACCCAGAGTTGTGGGACGAGCCACAAGTGTTTAGACCTGAAAGATTCATTGATGAAAGTGGGATGTTGAAAAATTCAGAACACATATACCCATTTGGATCAGGCCGGAGACGCTGCCCCGGGGACTCTTTAGCGAAGTCGTTCATCTTCATCACGTTCGTGGGTATACTGCAAAAGTACAGGATTCATGTCAGCAATGGTACGGTACCGTCTGACATACCGGTCATAGGCATATTGTCTTCGCCGAGACCCTACACAGCTGAGTTTACATTAAGGAAATGAAATTCAA

>gene14950

ATGTGGTTGGCCACGTTAACAATTTTTGTAGCCGTCTGCTTGTACATCTACTATGACACTCATAAACCTAAGAAATTCCCACCGGGCCCGAAGTGGGTTCCTATCCTTGGTTGTGCCAGCGAAGTGTATAAACTTCGAGAAAAAACTGGCTATTTATACAAAGCTGTGAGAGAACTCTCTCTGTCCTATTCTAAAGATGGCTCCCTGCTTGGTCTAAGAATCGGAAAAGATAGAATCGTAATGGTAAACAGTCTAGAAGCTAACAAAGAAATGCTCTTCAATGAAGACATTGACGGAAGACCCAAAGGCATCTTCTATCAAACAAGAACTTGGGGAGAGAGAAGAGGAGTACTCCTCACTGATGGAGAGCTGTGGAAAGAACAGAGAAGATTCCTGTTACGGCATTTGAAGGAGTTTGGATTCGGTAGACGAGGAATGGAGGAAATCGCAAGATCGGAAGCGCGGCATATGGTCAACGATGTCATGACAGTTGTTGGTGACAATGACGGGAAGACAGCCATTATACAGATGCACAATTTCTTTAATGTGTACATTTTAAACACGCTGTGGACGATGATGGCAGGCATCAGGTACAAACCTACCGATCCTCAGATGAAGGTCCTCCAAAGTCTTTTATTTGACTTATTCGCCGCAGTTGACATGGTCGGTACTCTCTTCAGCCACTTTCCTATCCTGAGCATTTTGACTCCTACTTTATCAGGGTATAAAGACTTCGTACGAACGCACAAAAGAATATGGAAATTTCTTCGAGAGGAGTTAGCTAGACATAAGGACCGGTTTGATTCTACACAAGAAGATAGGGATTTCATGGATGTGTACATAAGAGTTCTCAAAGAGAAAGGAGAGGTGGAGTCATATTCGGAAGGTCAGCTCGTGGCCATGTGTATGGACATGTTCATGGCTGGCACGGAAACTACTAGTAAGAGTATGAGCTTCTGCTTCAGTTACTTGGTAAGGGAGCAAGAGGTGCAGAAGAAGGCTCAGAAAGAGATTGACAGAGTAGTCGGTAAAGACAGGACACCCAGTTTGGATGACCGTCCCAATATGCCGTACAACGAGGCAATAGTGCACGAATGTGTACGACATTTCATGGGGCGGACATTTGGAGTACCTCATCGTGCCCTAAGAGACACCACTCTAGCTGGATACTTTATTCCAGAGGACACGATGGTGCTGAGCAACTTCACAAACATCCTGATGGACGAGGAGATGTACCCAGAGCCTTATGCCTTCAAGCCAGAACGATTTCTTGCTGAAGATAAGATCTGTCTACCAGACCATTACTTTCCCTTTGGTCTGGCCAAACACAGGTGCATGGGAGACGTATTAGCAAAATGCAATATTTTCGTGTTTACCACCACCATGCTGCAAAAGTTTTCTTTCCTACCAGTTCCTGGAGAACCTCTACCTTCACTAGATCATGTTGATGGGGCTACTCCATCCGCAGCTCCCTTTAAAGCTCTGGTGGTACCAAGGAAGCAGTAGAAGTCTCCATAATCCAAGTTAGGACCAACTCCTCGTCAGAAATGAGAGACGAACGAGCGAATAGAAAAGTCAGCGTATGACATTGATAACTTTCGTAACAATTATGTTAGGCT

>gene15344

GGAGTTCACACTCAGTCAGATTTGCAGCAAGTTTGCATATATATATAGGAACCTATATTATAAAATTTATATATAGGAACTGTTAAAAAATTGTCAACCATGTTCGCAATTCTCTTGTTTATGGTGGTACTATTCTCCAGTTTATGGCTGTTGAACAAATGGCGAAAAGTCAAAAGTTACTGGTTAGAAAGGGGCGTGCCTCATTATCCTCCACATCCCATTTTGGGAAGCCTCACCTTCTTGCAAAGGGAGAATCCTTCTGTATGGATGCGAAGAGTATACAACGACTTCAAGGCTCCTTACGTCGGCATGTGGCAATTCTGGCGACCAGCTCTGGTGGTGAACAGTCCTGAGATAGCTCGCAGGATCCTCGTGAAGGACGCTGATGTGTTCAGGAACAGATTCCTGAGTTCTGGCAAGAGTGACCCCATTGGAGCTCTGAACGTTTTTACTGTTAACGACCCTCTCTGGTCGAAGCTGAGAAGACGTCTGACGTTGGTGTTCACTGCTGCAAAACTGAGAAGTCTGAACGGCATCACCATCGCCAAGACCAAGGATTTCGTCAGACGCCTGGAAATAGAATCTAAAAAACCTGAACTCATTGACCTTAGAAAAATCTGCACCGACTTCACAACTGATGTCATTGGAGAATCAGCGTTTGGCATCACCAGCAACTCCCTGGCAGACGGCGACAGCATCATGAGAAGAGTCACCAGAGAGTTCCAGGCCTTCAATTTGCATCGAGGGCTGTCTTGGTCCAGCATCTTCTTCTTCCCTGAACTGGTCGATATATTCAGATTCAGTTTCTTCCCCAAAGACACGATGAAGCTGCTTCGCAAAATATTCCGTACTGTCGTGGAACAGAGAGGAGGATATGACAAGGATGTCAAGGATGCTCGGGACCTGCTTGATGCTCTGCTAAAGATCAAAAAGGAAGCTGACATGGAAAATGAAGAAATGACAGAAGACATTTTACTCGCTCAAGCTGCAGTGTTCTTGCTGGGTGGCTTCGACACTTCTGGTGCTGCATTAACCTGGACCATGTATGAATTGGCTTGGAACCCACAATATCAGGAAAAACTATACGAAGATATTTTGAACATGAAAAGGAAAATTGGTGACCGTGGTTTCGACTCTACCGTGCTTGCTGAGGTCGGATACCTGGACTGTGTTATTAAGGAAACCCTCAGAATCTACCCTCCAATGGGCTGGTTGGACCGTATCGCCACTAAGGATTACCAGATTGATGAAAAGCTAACTATCCCTGCTGGAACTGCTGTCTACGTGAACGGTGTCAGCATGCAGATGGACCCTGAGTACTTCCCGAACCCCGAAGTCTTCAACCCTGACCGGTTTCTACCGGAAAATGAACGGAGTATCACACCATATACTTTTATGCCTTTTGGAGAAGGACCGAGGAATTGTATAGGAATGCGTTTCGCCTACCAGACTCTCCGTCAAGCATTAGCAGAGATCCTACTAAAGTACGAAATCAAGGTCATTCCAGGCACTTTGAAACCCAGTGAAATAGAGATCGAGAAGAATGGCATGTTCTATATGCCCAGCGGGAAGATGAGCGTGCAGTTTGTACAGAGAGACAATGAATTTGTTAATTAGTGTTATGTTATAGAATATATTAGTTTTAAACTATTTATACTTTGCTTTATTAGTTACTTATAGTCACATACGTTTTTACGAAATAAATGTAAAAGTATTTATGTATCA

>gene15345

TCGGTACCGCGTAACATAATGTACTATATCTTTGTATTACCTCTCTGTATCATATCGTTATGGTTGTACTTAAAATGGCTGTCCATGAAGAACTATTGGAAGAAGCTCGGAGTTCCACACGAACCAATTCATCCACTATTTGGAAGTATGTCGTTTTTGCAGAAGAAAAATCCGGGTACATGGATGATGGAGCTCTACCAGAAATACAAGTCGTCTCCATACGTAGGCATCTGGATATTCTGGCGCCGTGGCCTAGTCATCAATGATCCTGACCTGTCGCGCAAGATTCTCGTGAAGGATGCTGATATCTTCAGGAACAGACTGCTGGCTTCTGGCAAGTCCGACCCGATGGGTGCCCTCAATTTGTTTACTGTTGATGATCCATTATGGACATCGCTCCGCAGGCGCCTGACGTCGGTGTTCACTGGCTCCAAGCTCCGAGGTTGGCAGGAGCTGTACCAATCTAAGGTGGAAGATTTGATCTACAGGATCAACTCAGACAATGAGAAGGGGATCATCAATGAATTGAGGCCCTTATTCGCTGATTACGCGACAGACATCATCGGTGAATCATCATTTGGGATCCAGTGCCAATCCACGAGGAACTCCACTGGTCCACTACGGTTGATGACCAAGGAGTTCGAGAAGTACAGCCAGTGGAGAGGCATGGCTTGGTCTTCCATATTCTTTCTACCAGAAATGGTCGATATACTAAGGTTTTCATTCTGGCCAAAGAATACAGTATCATACTTTAGAAAGGTTTTCAAAGTAATGGTTGAGGAAAGAGGAGGGTTTGAGAAGGACATAGATGGCAAGAGGGACTTGCTTGATGCGCTGCTGAAGTTGAAGATAGACGCTAAGAAGGAAAATCAAGATATGGACTTGGACATACTGATTTCAAACGCGATGATCTTCCTACAAGGAGGGTACGACACCTCCTCCACAGCCCTCACATACACAGTTTATGAACTTGCCTATCATCCTAAATACCAGAAAATATTATATGATGAGCTAGTGGAAGCAGAGAAGGCACTGAACGGGAAACCGTTTGACGCGGAGAGTCTCGCTAAGCTGCCGTATTTGGATGCTGTCATCAAAGAATCCCTGCGAAAATACCCCATTATGGGGTGGTTGGACCGCAAAGCTTTAAGAGACTACCAGGTCGACGAGAACCTGACCATACCAGCCAACACAGTGGTCTACGTCAACGCCATGGGCTTGCACTATGACCCCAAGTACTTCCCCAACCCTGAGGAGTTCAGACCTGATCGATTTTTACCTGAAAATGCGAATAACATCGTACCTTATTCTTACTTACCTTTCGGAGAAGGACCGAGGATGTGCATTGGTAAACGTATAGGAATGAACACAGTAAGGTACGCGTTAGCGGCTGTGGTACTCAACTTCGAAATCCTCCCATTGGACAAGTTCCCACTGCCCAACGACATCCCGATAGAGAAGAAAGGACTGTTCTACCATCCTGCTGTACCACTCTCTGTAGAATTTAAAAGAAGGAAATAAAAATACTACAAACTGC

>gene15812

CCTTAGTGAGTGCGTCGCGTCGCGTCTTATACCGAGTAAACAATGTTGTTCACGCTCGTATTGACAATAACAGTGTTGTGTTATGTGCTAATACATAGAAATTTTTTCAATAAAACTCATTGGAAGAAGAAAAATGTCGTGCAAACCAACTTCAATCTGATGCTGAAGTTCTTGTTTGGGAAGCAGTCCATGCCGGAGATACTGAAGGACATTTATAATGATCATCCTGATGCGTTATACGTGGGAACAATAGCTGGGACCACACCAACGCTGATCCTTAAGCACCCTGACGACATTCAAGCGGTTTTAGCTGGTGATTTCCAAAGCTTCCACAGCCGTGGCATCAAATTTGGACCACATGATCTATTAGCCGATAATCTGCTCTTTATGAATGATGTTAAAAGATGGAAGCTCTTGAGGCATAAACTCAGTCCAGTTTTCACAACCTTCAAGCTTAAGTATATGTTCAAAATCATTGAGAAAGTTGCTCGAGACTTTGTTGAAGTCGTTGAAGATGATATCCATCTGCGACAAGACCCATTCAACATTCTATACACTTACACAACTGCATCTATTGGAGCATCAGTGTTTGGTATAGATACACAGAACAATCAAAACGCTATGGATTCTCCGTTCCTGGAGATGACGAAGAAGACCCTGAAGCCATCTTTGTATGCAAATATTGCTGCTTTGCTTGCGAATACAAGCCCTAAACTATATGATTTTTTGAACATCAAAGTGTTTGGAGAACATGAGGAATTTTTCATTGGTGCTGTGCGAGCTGTTCTGGAAGAAAGACAACGCAAAGCTATTAGAACACATGATTTTATTGACCTCTGTTTAAGCTTGCAAGTCCACGGATATATGCATGACTTTACAACAAATTATAGTTTGGAGCCAACTGTTGAAATTTTGGCTGCTCAAGCATTCTTCTTTTTCCTGGCTGGCACTGATACCTCAGCTAATACTATGCATTATACTCTCTTAGAACTATCCAATAATCCCGAAGTGTTAGAAAAAGTGCACGAAGAAATTGATAAAGTATTCGATGAATGCAATGAACAGTTTACTTACGATGAAATAGAGAAGTTACAGTATTTGGATCAAGTGGTAAATGAAGCGATGCGGAAGTATCCTACAATAGGAGTCATCCAAAGACTCTGTACTAAAGACACAGTACTGCCGTCTGGAGTAGCTATAGACAAAGATGAAATTGTAATGATATCGCCATTTGCAATGCACAGGGATGAAAAGTATTTCCCAGAACCTGACAAGTTTGACCCAGAAAGATTTTCGCCAGAAAATGTGTCTAAAATACCGAAGTATGTGTTTTTGCCATTTGGAGAAGGAAACCGAATTTGTATTGGTGTCCGGTTCGCTCGCCTCCAAGTAAAAGCAGGACTCGCGTGGTTGTTGCGACGCTTCACATTGGGAGAACAGAATTGCACTCCGACATTTGAACGAAGTCCGTTTGGCCTTAGAAGTCCTACAGATCGATATGAACTGAAACTTAGAGATTAACGCCCAAATACTGGCAATACTACAAAATTTTAGTTGTACTCAGATACGTTCCATCTGTCATCATCATCATCAACAGCCTATAATTCCCCCTGCTGAGAAATGCCTCTTCTCACACGGAGAAGGT

>gene15894

TAAAATTCAAATCAGTTGACTGATAGGTAGCTTCCTGGTAGTACGCGTATCGTGCATGAATTCGTAATAAATAACAATATTTATTAATAAAATTCGAAACACCGGCGCTAGTCGTAATCAAGGAATTACCACATTTGATCGTGAAAATGGACATATTAATGGATCAACTTAAGGGTGGCTTAAAGTCATTGCTTCTAATGATGATGGAGGATTGGAAAATGTATCTGTGCCTTACTATTTTAACTAGCTTGTATCTTTATTATACAAGCACATTTAATTTTTTCGAAAAACGTGGTATTCCATACAGAAAGCCAGTTATATTTTTGGGAAACTTAGGCCCTAGATTGAGAGGCAAACAATCATTTCACGAATTCCAATTGGAAACGTATAATTATTTTAAAGGAAATCGCTTTGGAGGAATATTTGAAGGAAGAAGACCACTGTTGACAATTCTTGACCCTGACTTAATCAAAGCAATAACAATCAGGGACTTCGATCACTTCACAGACAGGATGGCTATGAATGCCAAAGAGCCGAAGTTCTGGAGCCGATCTCTTTTGAATTTAAAGGGCTCAGAATGGAAAGCGGTTAGAAGTACATTAACACCCGTCTTCAGTTCATCACGTCTTAGACATATGCTTCCGCTCATTGAAATGTGTTCTAAACAAATGGTTGAGTTTCTGAATCAATATGATAAGAAGGATGTAGAGATGAAGCAAACAATAGGCCACTTTACGTTAGAAGTAATTGGGGCATGTGCATTCGGTGTCAAATGTGATGCCTTGTCGGATGAGAATGCGCACTTCTTCAAAGTAGCAGAGAAGTTTGACTACATGCCGATGCATAAGAGAATATTGCTGTATTTTATCTTAATATTCGTGCCTCAGCTAATAAAATACTTGAACTTTTCGTTTCTGAATTTGGACTCTTCAAAGGAGTTGGTGAGAATTCTGAATGTTGCAAAGGATGAGCGACAGAAATCGGGCGTTAAGCAAAACGATTTTCTTCAAATCCTTATTGACTTCTCCGAAACCGAAAAATCTGAATCTGAGAAAACAAAGTCTACTGTTCATTTAGATGACGCGACCGTGGACGCTCAGCTGCTATTATTCCTCATAGCTGGGTATGAGACCTCCAGTACTTTACTTTCCTTCGCTATATACGTCCTGGCTACCAAACCTGAGATACAAGACAAGTTAAGGTCCCATATCATTGAGATGACAGAGGGAAAAGAAGTGGATTATGATCTCCTTGCGAAGTTGCAATATCTTGATGGATTTTTGTTGGAAACATTACGTATATATCCCCCTGTATCTCGTGTGGACAGGGTGTGCACAAAGCCATATAATCTTCCTGGAACAAACGTTGTTATCAAACCTGGCGAAGTGGTGGCTATACCTCTGTATGGTATTCAAATGGACCCTGAACATTACCCCGAACCGAAAGAATTCAAACCAGAGAGATTCCTAAATGAAAACAAGAGTGAGAGAGCATCTCATTTGTATATGGCGTTTGGTGTCGGCCCTAGAAATTGTATTGGTCTGAGATTCGCGATGTTATCAGCAAAACTAGCGATGGTTGACCTCGTGAAGAAGTTTAGATTTTCGCCATGTGAGAAGACAGAAGACCCAATACAATTTGACAGAAGGTCACTTCTGCTGAAGGCGCGTGGTGGTTTATGGGTTCGTGTAGAAGCCATATAATTTAGGTATCCCATGAGTATTTTTGATATACACAATATTTATATACATATTACTCATTGAAAAATAAATTAAGTTATATTTTATATTTATTGTACAACTTTATAGTACAAAGTAATTATGTTGCATTTAGAAAAAGATTGTTATTAACCAAATAATATCGACATGGAATTAAAATACGAATTAAATAATATAAACACTGATCAAGTATAGAAGGGTACCTTATGAAAACTACAGTATTTATTAGTATTTAATTTAAATTTATTGATTAAATGTATATTCATCACAAAATACTAGCCATATTTTACATTAAGAGTAGTGGATCTTCTTTGACCAAGTAATATTTAATATCTGTGATGCCTCACATTAGTTCATATAAATCCTTTTTTATATCTATGAGGCAGTATTCTTTTGTCTATAACCACTACTTCGATTACGATTTCGAAAATGCTGCTATAAAGAATCCAATAGAATCCTTGTAAATAGGATCTACTTTCCTAAGTTCATTGTCTTCTGGTCCAAATCTCTGTACCATTAATCTGAAATAGTACATTATAGATGAGTATAACATATTAGTGTGATAATTAAATTCCAATAATATATGTAGAGAAAACAAAATGTTTTACAAAATATATTTTAATTAATTACAAAGATTCTTGCCAACTTGCCAATATTGTAAGCAATTATTTATTTACGTTTTAAAAATAAATGTTTTAACCTA

>gene16257

ATGTATGTGTTAGTGCTAACTGTGATAATTGGTGTCTTGCTTTCGTTATGGTACTGGACGAGAAACTATCCTCGTTCTCCTCCAATATATCCAGGGGGATTACCGATTATTGGACATCCTCATATAATCATTAAGTACCGCAAAGATTTATGGGGCTTTTTGCAAAACATTGCTGATTACAGTGTGGAAAACGGAGGGATAGTCCAACTTTGGGCAGGACCTGTCACAGCTTATGTTGTGAGTGATCCAGAAGTCGTCGGAAACATAGCAAATACATGTTTGGAAAAGCCATTTTTTTACAATTTCTTAATTGATAGCATAGGAAATGGATTGATAACATTGAATGGACCCACGTGGAGGATACACAGTAAACTACTAAGTCCATCGTTCAACCAACAAGTATTAAACACGTTTCTACCTGAAATGAATGTACAGTCTCGAAATATGGTCGCACAAATGACTACTGTGGCTGGAAAGGGACCAGTTGACATCAAGGAATTTATAACTCAATATATACTAAGATCAGTTTGTCGAACTTCACTGGGATTAGAATCTAAAGATCAAGATATAATCGACAATGGTTACGCGAAAGCGCTAGAAGAAATCTTCAGAATTGTATGCTACCGTGGTTTAAATGTGTATGTACATCCTTCATGTATTTACAACTGGACGTCAATGAGAAGAAGAGAGTTGGAACTAGTCAAAATTGTTAAGAATATGATAAATCCTATTATTCAAAAACGCAAATCTGAACTAAAACCTACAAAAATGTATAATTACGATAGTTCAACAACAGTTTCAGGTAAATTCAAACCTACGCTAGATTTGATGCTGGATTTGTCTAATGAACAAAACGTCCTCTCAGATGATGATATTAGAGCGCATCTGAATACTTTCGTAGCAGCTTCTTATGACACAACTTCAGCAGTATTGCATAATGCGCTGATGGTGCTTGGATCATATCCTGATGTGCAAGAACGAGTTTATGAAGAGGTTCGAGACGTTTTTCAAAATAATGAGGAGTTGACGAAACACGATATGTCTAAACTTGTTTACTTAGAAGGTGTGATAAAGGAGGTACTGAGGGTATATGTCGCAGTTCCATTGATGGCGAGAAAAATTGATGCCGATATTGTACTGCCGAAATATACATTGAGAGCGGGAAGTATATGTATCCTATCAATATATGGCCTTCACCGTCACCCCTCCTGGGGACCAGACGCGAAGGAATTCAAACCGGAGCGATGGCTGAATCCCGATACATTACCTACTAACCCCAACGTATACGCTCCGTTCAGTTTTGGCAAGCGAAACTGTATTGGGAAACAGTACGCCATGATGAGCCTGAAGACGTCGATAGCGCATGTAGTGAGGAAGTTCCGCATTACTGCTGATATCAATGTTTTGAAGTGGAGATACGAAATTGTATTAAAACCTACAACAACCCCTCTTGTTAATTTAACATTAAGTTCCGGGGACGAAGGACTTGGGGAAGTCTCCGTCATCCCGAACAGGACAGGACTACACGTGGTGGTGGTGGTGCACGTTCACGAATGCCTCCAAGGTAAAGCATACGGCAGATGGACACTCCACTTGCCTGTGCGTCTCCAGGTGGAATCTAGGAGGCGGAACGTCACAAATGGGGCCGCGAATAAAGATTCCTACATTTTCACGTCCACTTGGGAGACAGGACATCG

>gene17041

TGCAAGGACCTGACAACCCGCTATGCTAACGATGTGATTGCGTCCTGTGCCTTCGGTCTGAAGGTGGACTCGCACAATGACAAGAACAACACTTTCTATGCGCTGGGCAAGGAGACCTCCTCGTTCAATTTTCGACAGATGATGACCTTCTTCTTGCTGATTAATGCACCAGCTTTAGCCAAGTTATTAAAGTTGGATTTCCTCTCGGAGTCATCAAAGGAGGCGTTCAAGAAGCTGGTACTTGGTACAATGGAAAACCGAGAGTTAAAGAAGATCATCAGACCCGACATGATTCATTTGTTGATGGAAGCTAAGAAAGGCAAACTGACTCATGATGAAATCAAATCCAATGATTTAGCAGCTGGATTTGCGACTGTAGAAGAATCAGCTGTCGGACAGAAAGAAATTAATAGAGTATGGACTGACGAGGACCTCGTAGCACAAGCAGTACTGTTCTTCATAGCCGGTTTTGAGACCGTATCATCAGGAATGTCCTTCCTTCTCTACGAGCTGGCTATGAACCCTGATGTTCAGGATAGGTTGGCGCAGGAAATCAAGGAGAATGATGCTAAGAACGGCGGCAAGTTCGACTTCAACTCCATACAGAACATGGTCTATATGGATATGGTTGTGTCAG

>gene17221

TGCAAGGACCTGACAACCCGCTATGCTAACGATGTGATTGCGTCCTGTGCCTTCGGTCTGAAGGTGGACTCGCACAATGACAAGAACAACAACTTCTATGCGCTGGGCAAGGAGACCTCCTCGTTCAATTTTCGACAGATGATGGCCTGCTTCTTGCTTGTTAATGCTCCAGCTTTAGCTAAGTTTTTAAAGTTGGATTTCCTCTCGGAGTCATCAAAGGAGGCATTCAAAAAGCTGGTACTTGGTACAATGGAAAACCGAGAGTTAAAGAAGATCATCAGACCCGACATGATTCATTTGTTGATGGAAGCTAAGAAAGGCAAACTGACTCATGATGAAACCAAATCAAATGATTTAGCAGCTGGATTTGCGACTGTAGAAGAATCAGCTGTCGGACGGAAAGAAATTAATAGAGTTTGGACTGATGAGGACCTCGTAGCACAAGCAGTACTGTTCTTCATTGCCGGTTTTGAGACCGTCTCATCAGGAATGTCCTTCCTTCTCTACGAGCTGGCTATAAATCCTGATATCCAGGAGAGGTTGGCGCAGGAGATCAAGGAGAACGATGCTAAGAACGGCGGCAAGTTCGACTTCAACTCCATACAGAACATGGTCTATATGGATATGGTTGTGTCAG

>gene2347

ATTTGCAGTAGTGTCACCTTTAACTTATTTCTTACCGTTATTTATTCAACTTGCGTCAGTATTTGGTATTAAAAATGTATTTTAAAATCGTTGCGCTCATACAACATACATAATTCACAGACAATTACCAATTCAATGAGATTTAGCCGTAACATAGGAAGATCAGGTAACGGTTCCCAATAGAGGAACTTAATTCAGTTTGTTTGGGGACGCAGGCGGGTCAAGTTCCGATTCCTTCGAAGAGGTGGATATTGAGTTATCATTTTACCGACAATAAGATAATATGATTTGTAACAGGAATAGAACTTTACTAAAACTCATAAAATTCAATAAGCATTTATCACACGATGCGTCTCCATCACCCAAGTCGATTGAATTTATGCCGCGTCCAAAATCTCTGCCTATTGTAGGAACTAAATTGGATTTCATAGCAGCAGGAGGTGGCTCTAAATTGCACGAATATGTCGACTTTCGTCATAAACAATTAGGTCCCATATTTTGTGATAAATTAGGAGGAAGTATAGATTTAGTATTTGTTAGTGATCCCGCACTAATAAAAACACTATTTCTAAATCTTGAAGGAAAATATCCCATACATATATTACCAGAACCATGGGAATTGTACGAAAAGCTTTATGGGGCCAAAAGAGGCTTATTTTTCATGAATGGAGAAGAATGGTTAGAAAATCGAAGAGTTGTGAACAAACATTTATTAAAAGAAAATTCAGAAAAAATATTTCATAATCCAGTCACAAATACTATCAACCAATTGGTACAGAAATGGATTATTGAAGCGAAAAAGGGCTCTTATGTGCCAAACTTAGAGACCGAATTTTATCGACTGTCTATTGATGTGATAATATCAGCAATGCTAGGAAGTTCTATCTTCCATAAGTTTAGCGTACACAGTGATGCTTTATTGACAGCATTTGCGGAAGAAGTCAAAAAAATTTTTCAGACCACAACGAAATTGTATGGGTGGCCTGTTAACATGTGTCAGAAGATGAATTTAAAAGTGTGGAGAGATTTCAAAAAATCTGTTGATATATCACTATTCCTAGCTAACAAAATTGTCGAGGAGATGATAAATAATAAACAACCTCAAGACGGACTGATTAATCTTCTTATTGAAGAAAATCTTAAACCAGAAATCATAAAAAGGATTATCGTAGATTTTGTCATCGCTGCTGGTGATACGACATCTTATACTACAATATGGACATTGTATCTATTGTCCAAGAACAAAGATGTGAGACAAGAACTATTTAAAAGAAGTTCTATTGCAAACTATGCGATAAAGGAGTCTATGAGATTATATCCCGTTGCACCGTTTTTGACCAGGATTCTGCCCAAAGAATGCACCTTCGGAGCTTACAAACTTAAGGGAGGCACTCCAATAATAGTTTCCATTTACACATCTGGAAGAGACAAGCAATATTTTAGCAGAGCTACAGAATACCTGCCTTACCGTTGGGACCGCAATGATATAAGAAGGAACAATCTTGTGAACCATGTGTCTTCTGCGTCATTGCCATTTGCTCTTGGGGCCCGGTCATGTATAGGCAAAAAGCTAGCAATGCTACAAATGACAGAACTTATAACTCAGGTGGTACAAAACTTTGAATTTGAATGTATAAACAAAGATGAAGTTACTTCCAAAACATCTCAGGTACTTGTGCCCAGCCAAGAAATCAAGTTGTCTTTCTCGTTGCGAAAACCAGAATGTAGTGAATAGACCTACATAATATTATCATTTATAT

>gene2437

CGTTCATATTAGACAAACTTATCTAACGAATCAAGGTCAAACTTATCATTTTCATGGTCAATTCGAATTCTATAGTACACAGCTCCTCTATTTACATCGGGCGGGTCAGTTTGTTATTCTGAGAATTTACACGCAAATTATAGTATTAAACAATGATAATGAGAAATAGAGCATTGAGTGAGTGTTATACTTTGTTTTTCATCAGATTTCATTCGTCCTTTCAAAATGTTCGACGAAGTTATTCTTCGGCACCGTCTGCACAAATTAAACCATACGCGTCCATACCGGGACTGTCCTCGTTGCCGATTCTTGGTCCTATACATCATTTCTTACCTGTGATTGGACAATTTGGACGAGGAGTAAATATATTTGACTTGATGTCTACCTTGCATGAAAAGTATGGGCCTATAGTAAAACTGCAAGGGGTGTTATCTAGAGCTGACTTTGTTATTTTGTATGAACCAGAACACATTGACCAGGTCTACAGGAGCCAGGAAGCAAATCCATTACGACCAGGGTTTCAAACTCTTGAGTATTACAGAGAAGTGATCAAAAAAGGATCTCTGGATGGAGTATACGGACTAACAACCGCACAAGGGGAGAAGTGGCGCGATTTTCGTACAAAAGTCAATCCACCATTGTTAAAATTGAAATTGGTGAAAGTTTATGCACCACCGCTTGGTGAGATCGCTCAAGATTTAGTACAGAGGATAAGGCGATACAAAGATGACACCCAATATCTGGCGAAGAACTTAGACCTTGAAATTACGAAATGGTCGTTAGAATCAGTAGCCTATGTGGCTTTGGGGTCTAGATTGGGGTGCCTTGGAGATGAAGTCACAAAGGACCATCCAGGATTTACACTGCTGCAATGTAGTAAGGATATAATCGAATACGCTTTTCAATTAGAATTTTTTCCCAGTGTGTGGAAATACTTCGCTACACCTGCTTATAAAAAGATTATCAGCACATATGACAAGCAATGGGAAATAAGTACAAATTACATTGAAGAGGCAAGAAAGCGAATTAACGCAAGAGGACATGACATTCCTGAAGAGGAGAAGAGTATAGTCGAAAGACTATTGGCTATTGACGAGAAGGTTGCCATCTTGATGGCTAATGAAATGCTGTTTGCTGGTATTGATACGGTTGCTTTTACAGCAATCAGTGTAATTTACAATCTAGCGACAAATCCGGAAAAACAACAGAGACTTCGAGAAGAAATTCGTTCACCTGACCCACATAAACGATATCTTCGAGCTTGTTTGAAGGAAACCCTTAGAATGCGGCATGTCATCCCGGCTAATTTGAGGAGGACAGATAGAGATCACTTTGTGGCTGGTTATCATATTCCTGCTGGGGTGGATGTAATAGCCCCAAATGAATACTTGTCAGGTTTGGAAAAATATTATCCGAGGCCCAAAGAGTTTCTCCCCGAGAGGTGGTTGGCAGATAAATCTGATCCTATTTACTATGGGAATGCACCTGCTATTGTGACTTTACCATTCGGTTTCGGTATCCGAAGTTGCATTGGACGTAGAATTGCTGAATTAGAGATTGAAACTCTAATGAAAAAGCTGTTTGATGAGTTTGAAGTAACATGGGAAGGACCACCGATAAAAGTTGTGACAAAAATCAGTAACATGTTTCTTGAGCCTTATAACTTTAGATTTAAGAGTGTTAAGTAATTTATTTCACTTTTATATATTATAGTTAATGCAGTA

>gene2558

TGAATGCATTATCGACTAGCTCGGCCGTACGACAACACTGTCTGTAGTCAATTAAATTAAAAAAATAACAAATAATATTTTTTATCTACGAAAACAAAATGGCTGTTTGCGTGTGAAGTGAAGTTATCGTTCATTTATTTTTATTTTTTGTTTCTGTTTTGTTATTATTGAGTGAAAGTGTTTTATTTGTATTAAAATGCCTAAACCAACTATTTTGATTCGCCAATCTCTATTCCACCAACCACGACGGCATATCGCCGGCTCTTCATCTCAAAGGACTTCCACATCTCCTCAGCGTCGGAATGTGGCTGCTCCAAGGCTGGCCACGTCTGCTGCTCGACCCTTCAACGAGATCCCAGGACCGATCGCCCTGCCTATGCTGAGACATTCGGCTCATGTATTGCCTAGAATTGGTAGTTACCATCATACTGTTGGACTGGGTTTGCTGGAAGGTCTTCGCGATAAGTATGGGGACCTGGTCCGGCTGGCGAAGGCATCCAGGTCTAGGCCTGTGCTGTACGTCTTCGATCCTGAGATGATGAGAGAGGTATATGAAAGTTACGCTACAGAGCCTCCACGCTTCGAAAAATCCCCGCTTTGTCAACATAGGAAGGTTGTGGGCCACTGTCCAGTTCACGGAGATGAGAACAAAGCTATTTGGGCGGCGATTCGCGCGCTGTTACAAGATGGCAGCCTACTGAAGTGCTACGACCAAGCCTTCGATGGTATTGCTGCTGATATCACGAGGAGACTGGGAGACTTGCGTCATGCAGAAAATGCTTTGAACGAAGAATTGGAGACTGAAATCTACCGGTGGGCCATCGAGACTATCGGTATGATGATCTTCGGCATTCGACTGGGATGCCTTGACGGTGCTGTGCATATACCTACTGCTGATAACCAGAAACCAGAAAAAACCTCAATGGATGACGAGATCCACGACCTCTGTTCCTTGGCGAAGCGTTCCCTAGAAGAACTGACCCCTGCAGAACGGCTGGTACGATGCAGCCGAGACATAGCTGATGGCAGCTTCCTTGTCAGGAGTGAAGGGACCCTGAACCCTGAGAATCCCACCTTCAACAGTGCTCTAAAGGCCTTTGACAGACATTTTACGCTCACAGAACATTTCCTGACGAAAGCACTAAACTCCCTCAACTCCGAAGAGTTGAGGCCTGAACAGGTTTTACTCGACAAGTTGCGACCATTGCAGAGGCGAATCCTGCCTCTAGCTGCTGATATATTACTCGCCGGCGTTCACCCTCTCGCCCAAACAGCTCTGAACATGTTTTACCAACTATCTCTTCACGCGGCTCGTCAGCAAAGGGCACATGACGAGGTGCACTGGTCCATAGCATCAAGGGAGGCTGGGTGTGACACACCCGAACTGCCCTACGTGGCTGCCTGCGCCAGAGAAGCTATGAGACTCCACCCAGCCACTGGAGGAGTAGTGAGGAGGAATAAGGAACCTATCATTGTTGGAGGCTATGAAATTCCTGCTGGGGTGGACATAGTCCTCGCTCACGGCGTAACAAGCAAATCTGAGAAGCAATGGGGAAGGGCAAAGGCCTTCGTTCCAGAGAGGTGGTGCAGTGAAGGTTGGGAGCCTTTAAAAGCTTCCAGAGCCCATCCACTAGCCTCCATGCCCTTCGGAGAAGCCTGTCCAGGTACCGGCGCAGTTGGAAAAATGCTATCGTCACTAACAACTAGAGTGTTGGACAAATACAGGCTAGAATGGCACGGACCTGCCCCGAGTATGGCCACCGTAGGAGTCAACAGAATGCATCCGCCTTATTATTTCGTTCTACAGAATGCTGCTTGAATATTCATTGTGATATAGGTTTAAGTTTTAGACTAGATTCATCACTTTTTAGTGAGAATGTATGTTAGTGATGGACTGTGGACTGTTGTTTTGTTATAGGCTAGAAATTTTAGCTGGATGTAATAAAAATTACTGTTATTGATTTTTATTGACGCATATTACATTCATTGTAATACGTAAAACTGTTATAAGTAACGTAAATTCCATACTTAACTGATTTTTGACATGTATCCTCCTAAATGACGTATGTATGTACAACTTTATTATTATTAATTTTATTCATTAACCATTTATTTCAACCGATTACTTAGATCATACATAATTTACACACACATAACAAACCATAAAACAATATAAAAGACTAACAACATTAAAATAACCCCGATCCCAATTCAAAACATTAATTTCACATTAGCCAGTACATGAACCATACAACAGTGGTTTTATGTACTGACAGTCAAAATGTTCAGCAAAGGCGCTCGGGATACTGTTGCGACTTACCCCGCACTCTTTTCACCAATGACGTGCACGTGTATTTATAGAACAATCAAAATCTGCAATCTTTATCTTCCAAACAACGATAATAAAATTTCAGAAGACAATGCGTTAGCTAATTAACTCGAATAAACCCTTTGAAGCCAGTTTCATAAAGCCCCGTTTTCTAGCAAAAGTGACGCTGCCAATTTTGGCATCCAATCAATCATTTTGGTTCAATACGCGTAGGTACCCGTTTTCCAACCTAGCCGTGTCCTGATTAAGACAGATTTGAAATTGAGAACAAAAAGATTAATTAATCTAATTAAGTTAATGAAAGTTTTCTAGACTACCTCTGACGAAGTCTTTGGGAAAGATTTTAAATGTGCTTAAGTAATTATAGTCCTAACAAATTAACTGTTCTTAGGAAGACTTTGTTTCTTTGAAAGACGAGCCCTTCGGTCGTATAATTATCTTTTCTAGTTGTCTGACTGTTTTTGACGACCTTTAGGGAATTATTCAATTTTCTATTCTACGTATTCACAAACTATTACATCAAGAAACCCCGAATATCTTCATAAAATTTTCAAATTTATTGTTTTGTAATAAAGTACAAAATCTATTAAATAAATTTGACTAATAAGGATAGCTTGATGTGTTAGTGTCTGTAAATAATTTACAGTGTGAATTTCATATACCTAACCATTTTCCCACGACAAAACGAATTGCCCTCAGTAGGATTATCAAAATACAGAACAAATCGATTCACCGGTCGACTTCCTTTCGCGCCATTGACCTCACCTACGAACATGTTACCATTGGTTTCAAGTCGAATTCGAGTTAATTTTTTACAGTAAAAATTTAGCATAAACAGTTTGTAATATCAAGAGAATGTCATTCATTAATATGTTTCAGTTGTACAGAAATTGTCAGAATTAACAATAAGTAATGTCTATTTTCCCTGCTTTGTGATAACTTTGTAAAATTCATATGGTCTTAGGACAATATTTCATTATGTGCCTATTACATAATTATGTTTTATAAAAGTTCCTTAGTAAGAATGTGTTGGATTTGTAACGTTTTTAATGTTATAATAATTTAATTTAATATAACAATGATTTGTATGTTCTTATAGGTATATCATTTATGATTAAAATAAGACATTGGTTATT

>gene3045

CCTCTTGCAACAAGCTTACAGTCTCCGACGATAGCTCAACGCGGTCGAACGCAACATGTTTTTCGAAAGTTTTCTTCTTAATCTCTCAGTCATCGTCGTGCTCATAATCGCCCTAATCATCGACTATGTGACCAAATTCTTCAGCTACTGGTACGTCCGGCATGTGCCTTACAAATCATCCATCCCCTTCTTTGGCGGCGACTACCACAGGGTCCTCGGAATCAGGAGCAGCACTGAAGATGCCAACATACTATACGCCAAACACCCCAAAGATAAGTATGTCGGCCGTGTCAAATGCAGGATTCCCGACCTGATAGTGAAAGATCCCGATACAGTAAAGAGGATGTTGTCAACGGATTTCGCAAATTTCCATAGTCGAGGTCTCTCCTTGGACAAATCTCAGGATGTTTGTTTACGAGACAATTTGTTTTACGCCGACGGAGAAAAGTGGACCCTATTGCGCGAAGGCTACGATTCTTTATTGAGTAACATGAACTGTGAATTGGAGAATTTGAGTGACTGTTTGCCGGGAACAAATGGTGATGCTAACGTTCAAGAGATTTTGTCGAAGGTATTGGATAAAGTCTTCGAGGATTTACTTCTTGGTAGCGGTGGAGGGTCAGTTGTAAAGGAAGTGAGATCAGCAATACAAAGGCGTACTTTGATAGAGAGATGTAAGAGTTATCTTAAAGACATTTTCCCGTCTTTGTACATTATGTTTGGGTTAACGACGATCTTTGGAGAGCCCTCATATAAAACAAAGAGTGTAATCGAAAAGTCAAAAGTACTCTCACAAATTAAGAACTTGGGTAACGTGTATCAACTTGGGATCAAAGAAAAGAATAGAAAGCGGCATTCCGATGTCGAATTTGCTTATTCAATAATTTCCTCATTCATAACCGAGGGGTACATACCGTGCCTGAATGCATTGACCGCATTGATTTATGAACTAGCATTGAATCCAGAGGTGCAAGAGAAATCTAGGAATTCGGTTGCAAAACACAATAGAGATGAATATTTGGACGTGGCTATTAAAGAGGCTTTGAGGCTGCACCCTCCATACTCAATAATTTCACGACAATGTACGAAAACATATCACCCTGAAGGTGGAATACTGATTGATAGGAAAATAACTATCAATGTTCCTGTGATGGCTTTGCACCGCGATGAAGAAAACTACATCAATGCGAATTCTTTCAATCCTGATAGATTCTTGGATGATGAAGGAGCATCGAAGCATTGTTACGCTTATCTGCCTTTTGGAGCCGGTCCCAGGAAATGCATCGGTGAGCAGTTGGCCTTGAAGTTGATTAAAAGTATAAGCAAGGCAGTTCTTAATAAGTACGAAATTGAGCCTTGTGCCAAGACACCATCAAAATTGACGATGGTGGACCATAACTTTGGGAGAGTAGCTGATAGGGATATCTGGCTCAGATTTAAGGTGAGAGCTTAAAGAAAGTAAGATTGTTTTGTGTTCGAGTAAAAAGCAGTGAAATTAGAATGTAGAATGTATATTATAGTATTCTTTGTGTTTTTTGATAAATGACAATTAATTTTAGTATATTATGTTGTTGATGAATTAACTCCATGACAGAATCAAACAAGAAAATGTAGCTACAATTTACTTTATTTTATTTATTAACGAATAAAATAAAAATGTTGGTTTTATA

>gene3586

CAACGGTAGTGTGTTTTGATTCGTGGGAAGCGAAATACATGTATTCAAAGTATTTTATTTTTATACGAAGTTGTTAGTGATTTTATGTGACTTTTTCCTAAAATGATTTGGCAAATATTTGTGGGTGTTGTGATAATATATGGTTTAGTACTTCGTTGGCAAGCAAGACATAGGATTAAGTTCGCTAAGTTATACAGAAATGACATAAAAGCCGCGCCTATACTCGGACATTCAGTACATTTTATCAAATGTAAAAATAACACTGATCGATGGAAGGTTCTAGAAAAATTCGGTCGCGAGGCGGTGAAGCAAGGCGGTATGACAATAGGTTGGAATCTACACAGGTTGTACTTTATCGTTGCCGATCCAGTAGTAGCTGAATGTGTTATGAAACATTGCCTAGACAAAGATGATTCAATGAAATTAACACTAGCACTGATTGGAAACGGCAGCAGTGTTGCTGATGTACCAATCTGGAGACCTCGTCGTAAGATCCTAGTATCAACGTTCAGTCAAAAGAATCTATTGGCCTTCACCGACATATTCGCTAGAAATAGTGAGATTATGGTGCAACAACTGCAAAAAGTGGCAGGAACTGGAATCATATCAATATCTGAATACATAATGGCTTATGCGATGGATTCCGTTCGTGAAACGTCTCTGGGCTTGAATGCTAATTCACAACTGAATCCAAACCAAGATCTGATAAAAGCATTTGCGGGCAGTTGTACTCTGATAGCAGACCGCATCATGAAGCCATGGCTTCACTTCGAGCCTTTATATAAACTGACAGCACACCATGCCAAATATATGGAGTATGTGGATACTGTCTTAGGATACATAATGGAGATGATTCAAGCTAAACGCAAGGAACTCGCTCAAAAGAAATTTAATAAGAATGATGACGAAGACAAAGACCGCATCAAAAGTTTCCTAGAACTCATGATAGAATCCTCAGGAGGAGACAAGGGGTACACTGACAAGGAGCTCCTGGAGGAGATAATGGTAATCTTCACGGCAGGTACGGACACCTCAACCCTCGCGTCTTCATTCGCTAGCATGTTACTGGCGAGACACCCTGAAGTGCAAGAGAAAGTCTACCAAGAGTGCAAAGATGTATTTGGAGACTCATACCTACCAGTTACAGCTGAAGAACTGAGCCGACTCAAGTATATGAGTGCAGTTATAAAGGAGACTCTGCGATTGTATCCCCCAGGACCAATCTTGTTGAGAAAAGTTGATAACGACGTGGTTCTTCCATCAGGAGATATTTTACCGAAAGGCACAGGCATCTTTGTATCTATATTTGCTGCTAACCGCAACCCTCGCTACTGGGGAGACGATGCAGACCAGTTCCGACCTGAACGGTTCATTGACACGCAGCTCAAGCATCCCGCTGCTTTCTTGTCCTTCAGTTATGGTCCTCGGAATTGTCTAGGATACCGCTATGCAATGTTATCAATGAAGTCAGTTTTATCAAATATTGTTCGCCGGTTCCGAATACTGCCAGTCTCCAAATCAGAGTTGGGAAAGCCACTGGACATAAATTATGATATCATGCTGCGGTCTGCAGATGACTACAAAGTAGTACTGGAACCAAGAGTATGATCTTGGTATTTCAATAATTCCCTTATATACAATTTGTTTTTTTATTAATTGTTGAAAATATGAAAATAAATTTATTTATTACCGTTCTGTTTCGTTACACAATAAATTGAACAAACTTGGTGCGTTTCATTAACATCTAAAATAATGACCCTTTAGGTGCACTTATAGTGTCAATAAGAGATTAAAGTGTATGTTACT

>gene3595

ATGTTGGTGGAATTGTTAATATTTTTAGTGATAGTGTTATTGTTATATGACCGTTGGTCTAGGAGAGAAGTGTACAAATTAGGTAAACAGTTGAGCTGCAGAGTAAAAGCCTACCCTTTGATTGGACACAGTTATTTGTTTTTGGGTAACAACCACAAACGAATGGAAGCATTACAAATTCTCGGTAGAGATGCTTTAAAGAATGATAATGGTTTGTTCCCGGCGTGGCAGGGAAATTATCTGTATACAATGATAGTAGACCCTGAGGCTGCGGAGATAGTACTGAAGACTTGTCTCGAGAAAGATGATATAATGCAAATGGCTACACTGTTTGTTGGAAAGGGCAGTATATTCGCTCCTGTTTCAATATGGCGACCTCGTCGTAAGGTCTTGGTGCCAACCTTCAACATGAAGAACTTGAAATCTTTCGTGACAATATTTGTAAGGCACAGCACCATACTTGTGCAGGAGTTAGGAAAAGTGGTTGGGGCTGGCCCACTGTCGGCCTTTAAACATTACACAGCTTATACCATGGACGCTATTGCTGAATCCACTTTAGGATACAAAATGGACGCCCAAAAGTCTTCTGATCATCCTTTCTTAAAAGCATTTAACTATGGACTTCCGAAGTGCGCAGAACGCCTGTGCCAACCATGGTTACACAGTGACTTGATATACAACAATCTACCAGTGTACGCAAGGCTGGTGAAACTTAAGGAATATTTATGGGGTTTTATGATTGAGTTAATCAAGGCTAAAAGAAAAGAAATTAAATACAGCAAACAAAGTAAAGACACTGAAAATTCACCAGAAGGAAATATTCAAACATTTTTGGAATGTTTAATACGATCATCAGGAGGAGACGATGGATACACAGATATAGAAATCTTAGAAGAACTGATGGTTATCATGGTAGCTGGTAATGATACTTCTGCTGTTGGAACATCTTTTATATCGTTGTTACTCTCGAGGCATCCAGAAGTGCAAGAGAAAGTTTATGAAGAGTTACAAGAAGTGTTCGGTGACTCCGACAGGCTGGCCACCTTCGAAGACCTGCCTCGGTTGAAGTACCTTGATGCAGTGATCAGGGAGACACTACGACTGTACCCACCAGTACCAATAATCGTTAGAAAAGTGGAAAATCATGTGCCGTTGCCGTCAGGCATCACACTCGTACCTGGAACCGGAGTTTTAGTTAATATCTGGGCTTTACATCGCAACCCTCGCTACTGGGGAGACGACGCAGAGCAGTTCCGACCTGAACGGTTTCTTGACACGCCACTCCAGCATCCCGCTGCATTCATGGCTTTTAGCCACGGACCCAGAAACTGTGCAGGTTACCAATATGCAATGCTGTCAATGAAAACAGTATTGTCGACGGTACTACGACGCTTCAAGTTCCTGCCAGCTGTAGCAAGTGACGGCAGCTTCCCTCCTCTGCGACTTACCTTCGACCTCATGATGAAGGATGTAAACAACTTCCAAATACGTTTAGAACCTAGAATTAAGTGTTAATGCGAACATTCTTCCCACCAGCTGCTATGTATTTCATCTGTGTGTAAATAGTAATGAAACGTCAAATTCTTACGTGAAGTAGCCATGCTTCGGGATGAAAAGCCTGGCTCCAACAGAGTGGCCTAACAGAAAACCATGTTTATAAGGTTACAGGAGGATTTTATTTATTTATGAAAAATAAAACATGTTG

>gene3596

TAAAGTTCATATATTCATGGACATCTTAAACCCTTTATTAATCTCTTTATATTAAAAAAATATCTATAAATTCACATCAAGTCTTAATGTAAACATTATCTAAGTGTATGTTAAAAGTGATACAAAATGTTGGTCGAATTAATATTACTAGTGGTCATACTCCTGCTGATACACCAACGTTGGACAAAGAGGGATATATATAAGTTGGGGGAACAATTGAATTCTGGAATAAGGACATTTCCACTTATTGGACACATTCATATATTCATCGGTGATAATGTAAGACTAATGAAAGCCTTCCAGGTGGTTGGCTTGGATGCACTGAAAAAAGAAAATGGCTTGTCATCACTCTGGTTTGGAAATTATTTGTATACCATAGTTGCTGATCCTGAAGCGGCGGACACAATTTTAAAAAACATTTCGGAAAAGGATGACATCGTTAGATTGGCTTCAGATTTTATTGGAAATGGTTCTATATTTGCTCCAGTACCGATTTGGCGTCCACGTCGCAAAGTATTAATACCGATGTTTAGTATGAAGAATTTAAAATATTTTTCTACAATATTTGCAAGTGAAAGCCAAATCCTGGTTGAGGAACTTGGAAAAACCGTTGGAAATGGTGCAATTCCAGTATTTAAATACATATCCGCTTATACGATGGATTCTATTGCGCAAACTTCCATGGCTTATAAAATGCATGCCCAAAGGAATTCTGATCACTTTTTCTTGAAAGGATTTGATATGGGACTCAATAGCGTAGTTGAGCGTATTTGTCAGCCATGGCTACACAGTGATACGATATACAAGAATCTTCCACGGTATTCAACGTTGATGAAGCATAAGAAGTTGCTATGGGACTTTATGCTTGAGTTGATTAGAAATAAACGAAAGGAACTTGAAGAAACCAACATGACCCAGCACCAACACGAAAATTCTTCAGATACTGTCCATAAGACGTTTTTGGAGAGTCTTATAAAATTGTCTGGTGGTGATGATGGGTATACGGATGATGAAATAGTAGAGGAACTACTGGTGATCATGGTGGCTGACACTGACACCTCAGCAGTCGCAGCCTCCTTCGTGACTTTGCTACTCTCCAGATATCCCGAAATACAGGAGAAAGTTTTTGAAGAATTACAAGAAGTATTCGGAGACTCAGACAGGTTGACCACCTTCGAGGACCTGCCTCGACTGAAGTACCTTGAAGCAGTGATCAGGGAGACACAACGGTTATACACCACCTCACCGATTGTAACCAGGAAAGTGGATAAAGAACTTTTATTACCTTGTGGTATAAAGCTGGTGCCTGGCACTGGTATCATGATCAACATCTGGGCTTTACACCGTAACCCTCGTTACTGGGGAGACGACGCAGACCAGTTCCGACCTGAACGATTCCTTAACCTGGATCTCAAGCACCCAGCTGCCTTTATGCCATTCAGTTATGGACCTAGGAACTGTGTTGGTTATCAATATGCAATGATATCGATGAAGACAATGTTATCTACACTACTAAGAAGATACAAGATCCTGCCAGCAACCAAACCTAACAGTAATGCTGAGTTCCCACCACTGCGCCTCAAGTTTGATCTTATGATGAAAGATGCAGACGGTTTCCAAATATGTATCGAGCCTAGATCAAAACGAGCTTAAATAAATTAGATTTAGATTAGTTTCTCATGAGTCAGTAAATCTAATATCTACCATTGTGGTTTTCTTCATATATTATTCGTATATTTATACATATTTCAACTAAAAGGTTTTTTCTGTTAATCATTTGTGATGTTATTAATATAAGTTTACTGATATTACATATATTTGTTTTTGTGTGGGGTATCGCTGAAAGTAAAAAATTTATCTTAAAAATTTGTTCACAATTATATAGCTATTTTATCCTTAACTGTCACAGACATTGTAATTATCATGATTAGCTGCCTCATTGGCCGACTGGTTGCATGTGCGACTGCCGGGCAAGGGGTCTCAGGTTCGATTTTTGGGTCGGGCAA

>gene3598

CGAATGTCGGTTGTGGTCGTGTTACATTTTCATCGCGATTAAGGTGCTTTTGTTAGGTGGTGCAAGTACATAGTTTTTTAGTAAAATGCTGTGGCAATTACTGGCTGTGCTTCTTTTAGTCTGGTCAGTGTATAGGTGGCGCAACAGAAGAAAGTATGCCTTAGGCAATAAGCTGGCCAGGAAGATCATAGCTCTCCCGTTGATTGGTCACGCTTACACGTTCTTGGGGACTGATGAGGATCGTTTCAAACAGTTTGAAGCAATTGGGCGTGAGTCCTACGAGACGGGGGGCATCTTAGCACAATGGCAGGGACACTTACTCTATCTCATGGTAACAGAACCAGAAATAATAGAAGTAATCCTCAAGACGTGCTTGGAGAAAGATGACCTTATGAGGATGTTCAGAGTTCTGCTAGGAAACGGCAGTATTTTTGCACCTGTCTCAATATGGCGTCCTCGGCGAAAGATCTTGGCTCCGACGTTCAGCCAGAAGAACCTCAATTCATTCGTTGATGTGTTTGCCCGACAGAGCAAGGTCATGTCTGATCAGTTGCATATTGCTACACAGAAGGGACCTGTGTCTATGTGGAAGTACATCTCTACTTACACCATGGATTCTGTTTGTGAGACAACTCTAGGTATAAATATGAACTCCCAGCTGGAACCAGAGCTACCCTTCCTCCAAGCCTTTGAGAATTGCTGCCGTATCGATGCTAAACGTATTTGCCAACCCTGGCTGTACAACGACACAATAAACAAAATCTTCTGCAGAAGTGCCTTTGATTCTTATGATCGCAGTAAGAAGTTAATTTGGAAGTTCATGGACAAGATAATAACTTCAAAATGGGCGTTACTAAAGAAAGAGACATCAGAATCAAATAGTGAAAAGCCGAAAGACAGTTGGAAAACGTTCTTAGAGTTGCTGATTGAGTCTTCAGCAGAGTACGGCAACCTGGAGCTGAGGGAAGAGACCCTGGTCATCGTACTGGCTGGTACTGATACTTCGGCAGTCGGATCTGGTTTCACTACACTCTTACTGGCCAAATACCCTGAAGTACAAGAAAAGGTTTACGAAGAGTTACAAGAGGTGTTTGGTGACTCAGACAGGCCAGTGACCCATGAGGACCTGCCTAGACTGAAGTACTTGGATGCAGTGATCAGGGAGAGCCTTCGACTGTACCCACCTGTACCTTTGATCACCAGGAAAATTGAGAAAGATGTCGCTTTGCCGAACGGTGTGACATTAGTAGAAGGATGTAGTGTATTAGTCAGTATCTGGTCCGTCCATCGCAACCCTCGCTACTGGGGCGACGACGCGGAGGAGTTCCGACCTGAACGGTTCCTTGACACCACGCTCAAGCATCCAGCTGCCTTCATGCCGTTCAGTTATGGCCCCAGAAGTTGTTTAGGGTATCTTTACGCTATGATGTCAATGAAGACCGCTATGGCGACTCTGGTACGTCGGTACAGGATTCGACCTGGTGATGGTCAGACGGGCAAAGAAAGAATACGAGTCAAATTCGGCATCATGTTGAAACCTGTCAACGAATTTATTGTCAAACTTGAACCAAGGACTAAGGGAGAGAAATGAACGCACGCATTTTATATGAACCTTTATATATGCCTGAAATTAAGGAAAAATTGCCATTTTTATCAACCACCCTTAAACAACCTCTTAACTAAGCGTTACTTAGCACGGGGATAACTCTTTATTTGTACTATTATTAGGTGTCAATTATAACGTAAGTTGTGAAAACCAAAATGTCTTTATTTATTGAACACTTGGAAAACCAATAGCTACACAATTTGAATTAAAAGGGTAAGAGTCACTTAATTACTTATTTAAAAGGCCACCTATCCTATCTTAGGGTATACTTAAAATTTACGAGAGGTTGGTAAAAACGGACAGAAGTAATATAAAATAATATAATAAACTACGTTTTACCCGCGGCTTTGTTCGCGTATTCCTGTTTTATCTCATGGAAGCATTTAAAGAACTTATTATAATAAGTACCCTATCACCAGGTAAGTCAGCGTATAGGTACCCTTCCTGCATACCAAATCCAATTTGCAAATCAAAGATCCTTTCAAATTTATAATATTAATGTAATAAATAAATGATATCCCATTTATTTAGTCATTATTATTACA

>gene3599

AAGTTTAACGCGACTATGTTGTGGCTCGTCCTCTTAGTCGTCACCCTCGGGTTTACCTTCAGATGGATCAAAAACCGACGTATACTACAAGTGGCCAGCCAAATAAAATGTGACATCAAAGCTTACCCGTTATTAGGACATGCGTATTTGTTTATTGGAGGCGGCGAGAGTCGCATGCGAACATTCGAGAGACTAGGTCGGGAAGCTATTAAAAATGGCGGCCTCACTAATATGTGGTTAGGAGACAATTATTTTACTGTTGTGGTCGATCCAGTAGATTTAGAAGTGATCCTTAAGTCATCATTGGAAAAGGATGATGTCATGCGGTTTGCGAGAAACCTTATCGGCAATGGAACTATATTTGCACCAGTGTCCATTTGGCGTCCCCGGCGCAAAGTCCTCGCTCCGACCTTCAGCCCGAAGAACTTGAACAACTTCGTGAAGATATTCTCCAGGCAGAGCAGCGTTTTGGCTGAACAGCTGCAGACAGAGGCAGGGCGAGGACCCTTCTCCTTGTGGAAGTATCTCACCAGCTACACCATGGATTCTGTGTGTGAAACTGCCCTCGGAGTAAACGTAAACGCCCAGAAGACGGAAGACGAGCCGTTTCTCAAGGCGTTCGAGAAGTGCTCCATACTGATAGCCGAGCGCATGGTACAACCATGGCTTTATGCTGGCGCTGTATACAAGCTCCTGCCGTACCACGACGCGTTTGAGAAGTCTAAGACTGTCATCTGTGATTTTATTGATAAGATAATTAAGTCGAAGCGAATTGCAATTAAGGAAAACAAAGACGACTCGAATAAAAATTCCAATGATTCGAAGACTTTCTTAGAACTTTTAATAGAGGGGTCTGGAGGTGAACGAGGGTACTCAGACCAGGAGCTGCAGGAGGAGACGTTGGTGCTCGCTGTGGCTGGTACTGATACTTCGGCTGTGGGAGCATCCTTCACCGTGTCTATGTTGGCACGGTATCCTGCTGTGCAGGAACAAGTTTACAAAGAGTTGGTAGAAGTGTTTGGTGACTCTGACAGGCCGTTGACAGCAGAAGACTTGCTCCAACTGAAGTACCTCGATGCAGTCGTCAGGGAGACACTGCGTCTATACCCACCAGTACCCGTCGTTGTAAGAAAAATTGAGAAGGATCTAGAACTTCCATCAGGAATCACATTGGTGCAAGGTTGTGGAGTTCTGGTGCATATCTGGGGCACGCAGCGCAACCCTCGCTACTGGGGAGACGACGCAGAGCAGTTCCGACCTGAACGGTTCCTTGACACGCCACTCCAGCATCCCGCTGCATTCATGGCTTTTAGTCATGGACCAAGAAACTGTTTGGGGTACCAATACGCAATGATGTCAATGAAGACATCAGTAGCCACTATTGTACGAAAGTACCGAGTGTCGATTCCCGGTACGATAAAGAACGGGTCTAGCACAGAACAGAAGCCTATTAGGATAACTTTTGACGTCATGATGAAGGATGCAGACAGGTTTACTGTGCAACTGGAGAGTAGAGGAAAGAAAGAAGAGAAGAGGAGATAGAGGTGTTACTCTTTTATTATCGGGTGATTGGCGCCAAACAATAGTTAGTGTTACTTTATTAGTACTGCTTAATCTACCTTCTTCGTGTGAGAAGAGACTTTTGGTCAGTAGAGGCCACTTATAGGCTGTTGATAATAATCATCTGATGATGATAGAAGTGTAGTTAATTTATTTTATTTATTATCTGAAAGAATACACTTAAGTTCATTTTAGCAAAGCGACGAGTTCGTCAAAAAAGGTTGTCTGGTCTAAAATAATGAATGTCTAACTTACATAAGTAATACATAAGTGCGAATATTATAATTAAATGAATGTAGCTACCTAA

>gene3634

CTCAGTGGAGTCCATTCGTTCGCACGGTCCAGTCGCGTCTCATAATCTCAAAATGTTTTTGCCAGTAATATTATTTGGCTTAGTTGTGCTATTATTGTTCACATACCTCTATAAAACTGGACAATATAATGAAAGCTACTGGAAGAAACGCGGTGTTAAGTTTTACGATAGAAGCAAAGCCATCGGCCCCTATTGGGAGTTTCTCACAACAAAACGAGCTTTGTTTGAGATACTTGGTGATATCTACAAGGAGTATAAAGATGAACCTGCTGTGGGAATTGGGCAGTTGTCAACACCGGCGCTATTTGTTATACATCCAAAGAATGTGCAGCAAGTTCTACAGACTGATTTCCAAGCCTTCCACCATAGAGGAATGGAAAGCGTTGAAGGTGATCAACTTTCCGATAATATTCTCTTCATGAATGGACCCAGATGGAAGCTTATGAGAAAGAGTATGAGTCCATTATTCACGGCCAGCAAATTGAAGAACATGTACTACATCATGGACAAAAGTGCCCAAGACTTTGTGAGTTATTTAAAAGGAAATCCTAAAAAACGTGGGAGTGACATATACGGAACACTTACAACCTTCTGTAATGCAGCAATTTGTGGAGCAGTTTTTGGCATTGGATCCGAATCAACCTTTGATTCACCGTTCATCAAACTAGCGGAAAATATGTCAGCAACAAAACTCAAGAACGTATTAAGATTTGCTATCATTGGAATGAGCCCAAAGATAGCAAATATGTTGGGAATCAAATTGTTCAAGGAACACGAAGATTACTTCATTAGTTCTATTGCAGAAGTTATAAGAAAACGGGAAGCAGAGAATGTAAAGAGACATGATTTTGCTGATCTGTGTATTACTTTGCAGAAGAACGGAACGTTGAAAGATGAGACGACAGGGTGTACTATTGAACCAACGACTGGATTATTATCAGCACAAGCCTTGTTCTTCTTCGCTGCTGGAGTTGAGCCATGTGCTGTAGTATTATTTTCAGTATTAACTCTTCTCTCTTGCCATCCAGAAATATTAGAAAAAGTGCACAAAGAAATAGATGAAAAGTTTGAAAAATACAATGGCCAAATTACTTATGATGTTATCAATGAAATGGAATATGTAGACAAAGTGTTAAGTGAAGCAACAAGGATTCTTCCTCCTAATGGTTATGTAACACGGCAATGTGTTCAAGATACTGTATTAGAAGTGGGTAATATCAAAGTAGAAAAAGGTACGAAAATTTTTACTCCAATTTACCAAATACACCACGATCCGAGATTTTATCCAGAGCCAGTGGTATTTGATCCAGAACGGTTCTCTAAGGACAGAAAGCCTAGCGATGAAATTTTTATGCCTTTTGGTATGGGTAATCGCATGTGCTTAGGAGCTAGGTACGCTAAATTGCAAGTACTAGCTGGACTAGTTCACGCTCTGAGACATTTTACCGTTAAATCTAATGCAAATATGAATAATCTTATTTTTCAGAAACATATACTAAATGTAAAACCTAATAATGTAGATATAAAACTTGTTCCTAGAAATAAAAACTAAGTGCTGATGACTTTAGTGTTTATGTTGTATCATTTTATTGTTGTGTATTCCATAATTTAAGAAACATAATTGTATGATCACATAAGTGGTAATTTAAAGATAAGATTACATTAAAAATGTATTGTTACTATAATTACTTTAGTGATCAATAATTCTTCTATTACCCTGATTTGTCTGATCACTTTTAATACTAAAATCGATAAGATTACGTTAGCACTTAATACGTCTTCTATAATTCTTCCGTTGAATACAACTCTCTGTTTGTTGTTGAACTCAAATGAGTCAATGTGTCTTTATCACATTTTTTTGTCTCATATACCTACAAGATTATTATGAAACATGTTCTTATCATAACTATTGCTCTGCTCATAACGTAACTCAGATGGGTGCTAAATAAGGAAGTACTGAACACAATGTCCGGGATGGTAACGATATGACACTGCATATGACCTATGTGGAGAAAGGGCAGTAGTCGTTACGTTTTTGCTTACATTCAACTAGATATTTAGAAAAGATAGAAGATATTTAGATATTAGACAAAATAAGACTTTGGATTAAAATTAATAAGGGCAAATTTTAGCCGTGAACTGGATGTCAAAAATACTTTTCCATAGAAGAAGTCGCAATAGTACTTGTTCCTTTAGAGTCTATGCAAACACACCAATGAATTAAAAGACAGACGGATAAAGTAATTGGCATCAATAAACACAGCAATAAAGATGAAGTAGTAACGCACAAAGATAAACGGCATTCATGGAGTAAATAAAACTTCCAAGGTCATAAACGAAACCGCGGCGACTTCGAATTTACATGTCAATCGAGGTATCAAAATCACATTATGGACATATACATAAACACATTTATCACTGAAACAGTAAGAGCAAGGGATTTCCGAACAGCAATCTTTGGACCCAAGTATTTAGGACAACCAGCGGAGCGAAACACTTGCAACACTATCTGGACAACACTAACGTGGACATCCCATTCAATTTTCACCTCTATGTTTACCCAATTTAAGTCTTTTGCAACTGCACGTGCTAACCCTGTCAGTAATACAAAAACCAGTTTGCAAGATCTATGCAAATGGATGCGGTACCCTGACACATGTTCTGCGTAAGTGCGGTGTGCGTTCAGAGTGGGATGCCTAGTTATAGTGGTCGGTACTCGTAAGGCATTTTACGATGTTGAGAATGAATGACTTGTTCCGTTGCGAAAATTATATATGTGAGTAGTATGATGCAAAAGTTTGTTTTTGTTGTAACGATTGGTTTCGGTTTCATCGGAAAATGTGCGGAGATATTTAGCGTCTTTGTGTCAGTAAGCATTTACCTGCATACACTAGAAAACTCATAAAATGTTTACAGTTCGTTTCTAACAATTCATGTTAGAGACGGACCAGCTTATTAATTATAAGATAACAGTGAACATCATTTTTGACCTTCTCGTTTTATAGTATTTTGTAGCACCTCCGATCTAATCAGTGCACAAGCAAAGCTAAAGTATAAAATACTAGACATAAGGGTCATATTTGCGTGTAACAAGCTTCCGGCACATTTCGGCACAACAAATAAACGAAGGTAACCTCATTAAGCAAAACAATTAAAAATTGAAACACGAATGTATGCAACATTCGATGTTTTCACAATAATT

>gene3671

TAGTACGTCAACCGTATAACCCGCGTTTATATTCACGCAGTCTATTGAATTTGTTAAATTTCCGTCTACTGAGAAGTTCAAGGCTAATCTAGATACGAAGAAGAAGAACACAATACAAGAACTAACATCAATTCTTTGAAGAAAATTAAACAAGATGTTTTTGTTACTACTGCCATTAGTGTTAGTCCTGGTGTACCACTACGTCACCAGAAACCACGACTATTGGGAGAAACGTAATGTGAAATATGAGAAACCTGTACCTATTTTTGGAACGTTGTACGCTAATCTCGTAGCCAAGAAGAGCATTACAGAAATATCTGTGGAGATGTACAACAAGTATCCAAATGAACAAGCAGTCGGCATATACCGAGGCACAACACCCGAGCTCATCATACGAGACCTAGACGTAGTCAGAAGGATTCTAAACGTGGATTTCGCATATTTCTATCCAAGAGGAATAGGAAGGAACCCCAAAGAAGAACCAGTATTTCTTAATCTTTTCCACGTTGACGGCGACATGTGGAAGCTGTTGAGACAACGCTTGACGCCAACATTCACGACAGCCAAATTGAAGAACATGTTCCCTCTAGTAGTGCAGTGCGCGGAGAAGTTACAGACAGTGGGTGAAAACATCGTGAGTCGTGGCGGAGACTGCGATGTACGTGAACTGATGGCTCGCTTCACCACCGAGTTCATAGGTGCCTGTGGTTTTGGTATTCAGATGGACAGTATCAATGATGAGCACTCCCTCTTCAGAGCACTTGGCAAAAAAATGTTTCAAAGATCATGGAAGAATTATGTAGCTGTCGCATTATATGATCTGTTCCCTGAATTTAGAACTATTATTCAAAAATCTTTACGTGAAACTGAAATAATGGATGCTATATCAGAAATAGTTGAGAACATTCGTAAACAGAGAAACTACAAGCCCATTGGACGTAATGACTTTATAGACTTATTGTTGGAACTCGAGAGTAAAGGTAAAATTGTGGGAGAATCTGTGGAGAAGAGAGACGCCAATGGAAAGCCAGAACAGGTAGAAATGGAGATGGATCTCACTTGTATGGTGGCACAAGTGTTCGTGTTCTTTGCCGCTGGGTTTGAGACGTCCTCATCGGCCACAAGTTTCATGTTACATCAGCTAGCGTTCCATCCTGAAGAGCAGAGAAAGATTCAGGAGAATATCGATCAGGTTCTAGCGAAATATGACAACAAACTGTGCTATGACTCCATCAGTGAGATGACAGCTCTGAGCAATGGATTCAAGGAGGCCATGAGACTGTTCCCCTCTCTGGGCACGTTGCACAGGGTGTGTGCACAGAAATATACAATTCCAGAAATGGGTATCACCCTCGACCCCGGTGTGAAGATCATAGTTCCAGTGCAAGCTATACAAGTCGATGGGAAATATTTTGATAATCCCACAGAGTTTAATCCGGACAGGTTCAACGATACGTCTGCGGACAGACATAAGTTCGCATACCTGCCCTTCGGTGAAGGACCCAGACAATGTATTGGTGCCCGACTAGGCGAGATGCAGTCCCTAGCAGGACTAGCAGCAGTACTACACAAGTTCAGCTTAGAACCAGCAGCGAATACAAAACGCCATCTAGAGGTCAACCATGGAAGTAACACGGTGCAATCAATAAAGGGAGGATTACCTTTGAAATTAAGACTGAGAAGCAAACAAAGTCAGGTTGCTGCCTAGTTTTATTAGGGATGTTGAAATTAGTGCTTGTATTATATCGAAACACACAGGTACTACTAACTCACGTAAATGTTTGAGGTTGCTCGACTGGTTTCAATGCACGACCGGAGTCGATAATCAGGGAACCACTTGAAACTGTTGAACAGTTACGTAAGTTAACCGTGTTTTGTAATTAGTTAAAATAATTTGTTATGTCTTTAAAACTGTTCATGTATTGT

>gene3751

TAGTGCGATACGCGAGGAGGCGCGCGCGCGCAACATGCTCCTGACTTGACTCCTTGTTCTACACGTACATTTGTTGCTAGTGATTAAAATAAAGTGACAAAATGTTTGTGTGGTTGTTACTCCTGGTTTTTGTGCTGTGGATGTTCACTGCGTGGGCTAGAAGGAGACACATGGTGAAGCTGGCTGCAGCCCTTCCAGGACCAAGGGATGAACTGCCTGGAATTGGCCTTGCACATAAGTTCATTGGTGATACTGAAGCTATCATGCATCAGCTGCAACTCTTCAGTTATGAGAGTATGGAGAACGATGGAGTCCTGCGAGGCTGGCTGGGAAACACCCTATTCTTTATCGTAGTTCATCCTGGCGACCTCGAGATGGTGCTGAAGACTTGCCTGGAGAAAGATGATCTTCACAGATTCATCAGGACAGTCATAGGAAATGGAGGAATATTCGCTCCAGTGTCCATCTGGCGTCGTCGTAGAAAGATCCTCGTGCCTGCATTCAGCCCTAAAGTAGTAGAGAACTTCGTCCATGTGTTTGCGGAACAAAGTGATAAGTTAGTGAAGCAGCTGTCACGATGTAGTGGCAAGGGAAAGTTCCAAGCCTGGCCTTTCCTTAGCACCTACACCCTGGATTCAGTATGTGAAACGGCAATAGGAGTAAAAATTAACGCGCAAGAGAATCCGGATTCACCTTTCCTTAGAGCGATGTGTGATATCCTGAATATGATTTGTAAGAGAATTTTCCACTTGTGGCTGCAACCAGATTGGCTGTTCAAGATATTACCGATGTACAAAAAACACCAAGAGTGCATACACGCCATGCACAATTTCACCGATCGGGTTATCGAAAGCAAGAGAGAAGAATTAAAAAGAGAAAAGGAGTCTAAACCAGAGGTGGATCATGAGTTTGATTTAGGGGCATACAAGAAACAAACATTCCTAGACCTACTGATACACTTATCTGGTGGTGAAAGGGGGTATTCCAATGTGGAATTGCGAGAAGAAGTATTAACCCTTACCATCGCTGGTACCGACACCTCTGCTGTTGCCATTGGATTCACTCTCCAGCTACTGGGAAAATATCCAGAGATCCAGGACAAAGTATACCAAGAATTATGTGAAGTTTTTGAAGACGACAGAATTTTAGTCAAAGAAGATTTAATGAAGCTGAAGTATCTAGAGAGAGTAGTCAAGGAGTCTTTGCGGCTGTTCCCTCCCGTGCCACTTATTATAAGAAAGGTCTTAGAAGACATTACGTTGCCATCTGGTCGCGTCCTACCAGCTGGTTCAGGCGTAGCATGCTCAATATGGGGTGTCCACAGAGACCCCAGATACTGGGGTCCAGACGCCGAGAGATTTGACCCCGACAGATTCTTGCCAGAAAGATTTAATCTAGAACACGCTTGCAGTTACATGCCCTTTAGTAATGGTCCTAGGAATTGCATTGGTTATCAATACGCCCTCATGTCAATAAAGACTGCGTTGTCAACAATATTACGGAAATATAGGGTAATTATGGACACCGAAGAAAGCCCTTACCCATATATCAGGGTCAAAATAGATATTATGATGAAAGCAGTTGACGGATACGAACTGAGACTCGAAAGACGAGAAAGAGATCACCACATATGACGTTTCAAAAGCGCGGGAAAAGTGACATTAGCTTTTTATAGGTTCTTATTCCTGCCAGCCGTAAAAATATTTATTAACTTTTTTTGTATTGCGTTATAGTATATACAGCCAGGTTAATATGGATATCTACTATTAGAGATTAAATTAATTTT

>gene3829

CGCCGAGTAGCGAGCTTGTTTTGTTTATGTTATGTGCATAATTTCGCGGGGAGGAGATCGTTTGTAATTCGATGATTTCGTTCCGTGGCGTCTTGCGATCTGGTTGCACGATACGGAGAATTGTAAAAAATGTACAAGTCATCTAAAATATTTTCCAATCGTTTGAAATATGTGAATTTAGTGTCGTACGTAGCGAAGTATGGGCGTGACAAGGCACAGAACTGTGATGTAAGGAGTTTTGAAGAAATCCCAGGACCTAAAAGTTATCCCATAATAGGGACGCTTCATCAATATGCACCTTTTATTGGTGACTACGATGTCGAAACTCTAGACAAAAATGCTTGGTTGAATTATCGCCGCTATGGCAGCCTTGTTCGTGAAACACCAGGCGTCAACGTCTTGCATGTTTATGATCCAGAAGATATAGAAACAGTATTCCGACAAGATCATCGGTTCCCAGCAAGGAGAAGCCATATTGCGATGTACCACTATCGTATGAACAAGCCTGATGTGTATAGAACTGGTGGATTACTGAGCACAAACGGTGAAGAATGGTGGAGACTAAGGAGTACATTTCAAAAGAATTTCACCAGCCCGAAAAGTGTTAAAAGTCACATAGAAAGTACTGAGATTGTGATAAGAGAATTTATTAACTGGATAAAAGAAAGAAATGTATCGCACAATGAAGATTTTTTGCCGTATTTAAATAGATTGAATTTGGAAGTTATTGGTGTTGTAGCATTCAATGAGCGTTTTCAAAGCTTTTCTCCGGAAGAACAAGATCCGACGTCCAGAAGTAGCAAAACAATTGATGCGGCATTTGGTTCCAACTGTGGAATTATGAAACTCGATAAAGGATTTATGTGGAAGATCTTCCAAACTCCTGTTTATAAGAGACTTGCTGATTCACAAACGTATTTAGAAAAGGTATCTACAGATATTTTATACAATCGAATTCATTATTTTGAACAACCTGAAGATGGCGACATTTCTTTGTTGGGATCTTTTCTGAAGCAGCCCAATGTGGACTTAAAGGACGTCATTGGAGTGATGGTTGATATTCTTATGGCGGCTATAGACACGACGGCATATTCAACCAGCTTTGCTTTATATCATATTGGAAGAAATCCCGAAGTTCAGCAGAAAATGTTCGAAGAAATCGCAACTCTTCTTCCCACAAAAGATGCCAAAATAACTCCTGATATTTTATCAAAAGCAACTTACGTACGAGCTTGTCTCAAAGAAAGTTTGCGACTTAACCCTGTGTCGGTTGGCATTGGTCGTTTAACTCAAAAAGATTTTGTTTTAAGGGGTTTTCTAATTCCAGAAGGGACAGTCATTGTAACTCAAAATTTTGTGGCTTCTCGAATGCCACAGTATGTAAAAGATCCATTAAAATTCAAACCTGAACGGTGGATAAGAGACTCGGAGAGTTATGAAAACATTCATCCATTCCTGAGTTTGCCATTTGGTTTTGGACCACGGTCTTGTATAGCAAGGAGATTAGCAGAACAAAATATATGTATTTTCTTAATGCGGTTGATCCGTGATTTTAACGTAACATGGATGGGTGAAGATCTTGGTATTAAAACCTTACTTATAAATAAACCAGATAAACCCGTCTCTTTGTCATTTACTTCAAGAATCGTGTGAATACAAGAAATTATTTCAATAGATTTGTGTCAATATGTTGTGTCGTATGTACATATTGTGTATTTATTTTGATTGATGATGGTAGGTCAATAAAATAATTAA

>gene390

TAAAGATGGATATTATGAAGTAATAGTAGTGTGACTGCTTTCATAATGATAAAGTTAGAACAATTTAGTTTAAATGCAATGAATGGGTGGGATTGCGGAAGATACACAAATGTACGCATTGAGCAGAGACCGGACGAGTCAATCGGCGCGCGAGCACCGCTCCCTGCTTCCATCGAGGGATTGACAAATTGAATTGATGGCGGTGACGTCACGCGTAATACAGATGAACACTGAACATCAAATTATCAGAATGTACTAATGTGTACTTACCTACATGCAAACATCCGATGTTAATAATCGTAACAACAACGTTGTTATCACACCAGACTTTTTAATTAATCGTGGCCCCATTTTGCAAATTAAATACGTGAGTAAAATTAATTTCAACGTTTTGATCGGCGCGGTCTAATTTTATCCCTGTATGCTCGTTACCTTAACACCATGCGAAGTACCATGCACGACTAGCAATATTATGCCAACCTCTTTATTAAGAGAACAATTTGCGTTGCCGTTTAAATTTTATATTGTTTCCTATAGGCTTATGGTTCTAAACATTAGATACACAATTTACGAAATAATACTGCAGAGTGAAATTGAAAACCCTAGCAATGACTTGCATTTTCCTTTAGTCAGTTTCTAATATTAACAAGTACACTCTAAGTAGTGGCATCACACTTGTGACAATAGGGCGCTACAAATACGCATAGTTTCAAACGGTTTTGTCAACAATTAAGATCACCATTGGTGTCGTAAAATGGAATTAAGAAAGCATTCGTGATGGCGAAAGAAATGCAAGGTTTATAACTAGCAATGCAGGGCCTGTCCTCGTCAATAATCAAGGACCTTCAGTAGTAAAACTCGATTAAGTTCCGCCAAAAAATATTTTCAAGGAAATGTCAACCCCCTCCAGATGTGAGTGAGAGGTCTGGCAACATCAATTGAGGTTGTATTTATTTGTACGCATGCGTGTGCACTGCGTCAACTGAGCCGGGTGGCGTATATTACGACATCAACGTCGCTGAAAACGTAACATGGTAGCCCCCACTGCTTCCTCTGTAGTCTTGGACTTAGGGGCTGAACCTAAAGCCATTGACAAAGCGTCCGCGATGAGTAACGCTCGGTGCTACGCGGTGATGCCGGGCCCTAAGCCCCTGCCTATTCTCGGTAACAGTTGGAGATTCGCTATAGGGAAGAACCCTTGGAGAACCCGTGCTTTGGATGTTTCACTGTGGTTCCTGAGAGCTTTGGCAGGTGATGGGGGAGCGGCTAAAGTGTCCAAATTATTTGGACATCCTGACTTAGTGTTTCCATTCTGTGCTGAGGAAACTGCAAGGATTTACAGGCGGGAAGATTTAATGCCACATCGAGCTAAGGCACCGTGTCTCAGTCACTATAAACAGAATTTGAGGAGAGACTTTTTTGGGGATGAGCCCGGTCTAATTGGAGTACATGGGGAGCCGTGGTCTAGATTTAGATCGAAAGTATCCAAAGCATTGATAGCACCGGAGGCAGCAAGGGCTGCAGTGCCCGATTTAGAACATGTGACCGACGATTTTGTTCACAGAATGGAATGCATTCTCGACGATAATCGTGAGCTGCCGAAAGATTTTCTCACGGAACTCTACAAATGGGCCCTTGAGTCTGTGGGCGCTTGGGCCTTGGGGACAAGACTCGGATGTTTAAAGGATAACGATGCTGAAGCAAAGGAGATAATTAGGTGTATTCATGGGTTCTTCCATAGTGTTCCTGTACTGGAACTGTCTGCACCCTTGTGGCGAATATATTCAACGCCGGCTTACAAAACATATATTGAAGCTTTGGATGCGTTTAGAGCGCTGTGTTTGAAAAGGTTGACTGATAAGGGTATTTGTTCTCAAATTGCGAAGGCTTCTGGAGAAAAGGTTGCCACTATATTGGCTTTGGACCTGCTACTGGTTGGTGTGGATACCACAGCAGCTGCTGCAGCGAGTACTTTGTATTTACTGGCCAATAATGAAAGAGCACAGAAAAAACTACAAATAGAAATGGACAACAACCTGCCCAGAGACAGAGGAATGACTAGTAAGGACTTAGACAGGATGCCTTACCTCAAAGCTTGTATAAAGGAATCATTGCGAATCAAACCAGTTATTTTGGGCAACGGAAGGTGTATCCAATCAGATGCCGTGATATCTGGATATTCCGTTCCTAAAGGGTCGCACGTCGTTTTCCCGCATTACATCCTATCAAACGAGGAACGGTACTTCCCGTCGCCGAAGGAATACGTTCCCGAAAGATGGCTACGTGACAATGAAGTTCGGGCGCCTGATCAAGCTCCTTCAGGATCGATAACTAATGCTGATAAGCCAGGATGCTCGTATACAAGAGCGATGGAGGTGTGTCGCAAGCAAAAGGAAGTAGGGATCCACCCATTCGCCTCACTTCCTTTCGGCTTTGGACGACGCATGTGTATTGGAAAGAGATTTGCCGAAGCTGAGTTGCAACTTTTACTTGCTAAGATATTCCAGAAATACAATGTATCGTGGCGGTACGGCGAGTTAACTTATAGTGTAACGCCGACGTACGTGCCGAACGAACCGTTGCAGTTCACACTCCATAAGAGAAACGGTAACAACACTAGCCTATAATTTTACATGTTAAACAAGTAAGGCGAACAACCAAAGACGATTTAATTTAGGCAACGATTTCTATGAGCAAGTTTATATTTAACTTGCAATGTATGTCAGTATGTTAGTTTAGGATTAAACTAAGTTTAAAATGTGCACGGCAGACTTTTTCAAATAAACCCTTTATGTTGGCACCTTTATTTGCATTTCATTATTTATACACTCGTAATAGCTGTTGCAACTGGTAAGTTCAATGTTTGGTTCTAAAACAAAACCTACATTTCGGTCGAAAGAAAGTCAACCTTTACTCATTCAATATTATGGGGATTACGAAATGTCCATAAAGTAACGGTTCATGTAATGTTTGAGTAGAATTTCTTTGCACTTGGACGTGTCATAAGTG

>gene4500

TGTGGAGTATACCAAACACACACAGTCGCTCGCCGCGGACGCGAGCTGACGCGCAGTTTTTGTGCTGAAATACATCCGCGCTTGTTTACATTGTGTTTTAAATATTATTTTTTGATCGTGAAGCTCTAGTGCTGCCCTCTATTGAAATATCTCGCAGTATTATCTGGCAAGATGATCGTCATTGCCGTAGTAGTGGTGCTGCTCATCACTCTGTACTTCTACGGTACGAGGAACTTCAAGTACTGGGAGGACAGAGGAGTGAAACATGATAAGCCACTCCCACTGTTCGGAAACAACCTGCAGGGCTACCTGTTCCGGTCCAGCATGACACAAGTAGCAGACGAGATCTACTGGAGATATCCCAACGAAAAAGTCGTCGGATTCTACCGCTCCTCGCGACCCGAGTTACTCATAAGAGATCCAGAAATTGCGAAACGTATCCTGATCACTGACTTTGCCCACTTCTTCGAGCGTGGCTTCACTCCCCATAAGACAGTGTTTGAGCCTCTGATGCAGAACCTGTTCTTCGCTGAGGGAGATCTCTGGAAGCTGCTCAGACAGAGAATGACACCGGCCTTCACTACAGGCAAACTGAAGGCAATGTTCCCGCTAATCGTTGAACGAGCAGAAAAACTGAAGACACGAGCGCTGACTGCGGCCGCTGAAGGCAAGTCTCTGGACGCGCGCGACTTGATGGCGAGGTATACCACTGACTTCATAGGAGCCTGTGGCTTCGGACTCGACGCCGACTCACTGAACGATGAGGACTCACCGTTCAGACAGCTCGGAATTAAAATATTTAAAATGAGACCTCAAGAAATTATTATAGCCATTGCAAAAGAATTATTTCCAGAACTGGGCAGGAATCTAAAGATATGGACTCGAGTGGAGAATGACATCAACGAGCTTGTCGTAGAGATATTGAAGAAGAGAGACTACAAGCCCTGCGGGAGAGGAGACTTCTTAGATTTAATGTTGGAGTGTAAGCAGAAGGGAGTGATGGTCGGAGAGTCGATACAGAATAAGAAACCCGACGGCACCGCGGAGACAGTCACTCTGGAGTTCACCGACTCGCTGATTCTTGCTCAAGTGTTTGTGTTCTTTGCCGCTGGCTTCGAGACCTCGTCATCAGCGACCAGCTATACTCTACATCAGCTAGCGTATCACCAGGACGTGCAGAAGAAGGCGCAGGAGGAGATCGACCGCGTGTTGGCCAAACACGACGGGAAGCTGTCGTACGACGCAATTAGAGAGATGCACTACTTGGAGAACGTGTTCAAGGAAGGGCTACGTATGTTCCCGTCGCTCGGGTTCCTGTTGCGACAGTGTACGAAGTCATTCACGTTCCCGGAGCTGAACCTGACGATAGAAGAGGGCGTGCGAGCCTTAATTCCTCTCCAGTCGCTGCACAATGACCCGAAGTACTTCCCCGAGCCGCACGTGTTCCGCCCGGAACGGTTCGAGCCGGACGAGTTTGACGCTAACAACAAATACGTGTACTTACCTTTCGGAGGCGGACCCAGAGCTTGTATAGGTGAGCGCCTAGGTCTGATGCAGTCACTAGCCGGGCTGGCAGCAGTGCTGTCTTGCTTCACGGTGGAACCTGCCAAGGAGACAGTGAGGCATCCTGTCGTGGATCCACTCTCCAGTATAGTGCAGAGCATCAAGGCTGGTCTACCACTCATGTTCCGGGAGAGAACTAAACATGCTTGAACTGATGAAATAATTCGTAAAATTTAGTAAGAAATGACTTGGGGCTTGATTCACAACAGTTTTTGATTCAGGAATTTCTTTATAATTCTGTTAGGTCAAGGCCCGTGCAGTTGGAAAGGGATACACGTATATTTGCATCTCTTTTCAACTGTACCTACCTTGAGAAGATATCTCGATTTGAGTGTTGTTATTTTATAGGGGCCTACAGAACATATAATTTGATGTATTTTCCAGGAAAATGCTTGTAATAGGTACTTCTTAAAATATTTTTCAATGAAACCTAATTGGTAGTAATAGAACAGGCCGTAGGTAATAGTAAAATATTTAATAACTAACGTATTATTTAAATAAGTCTACTAAGTGTAGTATTAAGTGAATGTAATATAAGAAACGTTTCCAAATAATTAAAATTGAGGACAATTCAGTTTGATGTCAAGTTTTTTTATCCAATTTCATGCGAGACTGGACATTGTGACGTCATGTTTTGCGCAGAATAAGCGCACTATTTTATACTTACCAGAAAATTATATTACTAAGAAACGAATTTATTGGATGTAATTTTTGAGTAGGAAACCTCAGGCGATACATGGCGATTTAAATCTAAAATAAATAAAGGAGATTACCGTAGATCGTACGGTAATCATATACCAAGGAAGTTAGCCATTATATTTAATTTTATGTAATACCTATATCTACCTAGTATAATTAGTTCTTCAGTTCGTTCAAAGGTCGAAGCTATAGTCCCTAATAACGAACATTTATTTTACCTATCTCTGCCAGTTACTATTATAATTATATTGTAATTTCAATGAAATGCTTTGTGAAACATGTATATTATAGTTATAATTAACTATAATGAATGATTGTCATCGCAGTTAGCGATATAAAACTATTGTTGTACCGTGTAGGTACTGTTTATGTACTAACCTTACAAATATTGAGTGCAATAAAGTATCATTATTTTGTATTGTA

>gene4502

GTGCAAAAACACAGGACATTCGCATTTTGGAAGTCAAACGTTGAACTTGTAGTGAACTGTGGAACTCAGTGATAATTTATTAAATCTCTTTTATATTTATTATTAAGATCACATAAACTATTCGTGACACATTCAACTGGATACAAGTACCTCCCGTTTGTAATAAACTAACCATGATTTTCATCGCTATTGTTGTGGTCCTACTGATCACTCTTTACATTTATGGAACAAGAAACTTCAATTATTGGGAGAAAAGAGGTGTCAAGTGTGACAAGCCATACTTCCTTGTCGGGAACAACTACAAGGAATACTTTGGGAAAGCCAGTATGACACAAGTAGCTGACGAGCTGTACTGGAGGTATTCCGATGAAAAAGTCGTCGGTTTTCTTCGCTCTTCGCGTCCAGAATTAATTATACGAGATCCAGAAATTGCGAAAAAAATATTAATCACAGACTTTGCTCATTTCTTCGAGAGAGGTTTCAACCCTCACAACACCGTATTCGAGCCTCTGATGCAGAACCTGTTCTTTGCTGAAGGAGATATCTGGAAGCTGCTCAGACAGAGAATGACACCGGCCTTCACTACAGGCAAACTGAAGGCAATGTTCCCGCTAATAGTTGAACGAGCAGAAAAACTGAAGACACGAGCGCTGACTGCGGCCGCTGAGGGCAAGTCTCTGGACGCGCGCGACTTGATGGCGAGGTATACCACTGACTTCATAGGAGCCTGCGGGTTCGGACTTGACTCAGACTCACTCAATGATGAGAACTCCCCGTTCAGACAACTCGGTGCAAACATTTTTAAAATAAAAACTATGGATATCGTAAAAGGAGTAGCAAAAGAATTATTTCCCGAAATTTTCAGAAATCTGAAAGTATGGACTCGACTAGAGAATGAGATCAATGAGCTTGTCGGAGAGATTTTGAGGAAGAGAGACTACAAGCCCTGCGGACGGGGTGACTTTATAGATCTAATGCTGGAGTGTAAACAGAAGGGGGTCATAGTCGGCGAGTCAATTCGAAAGAAAAAGCCTGACGGCACCCCTGACACTGCAACACTAGAAATCGATGATGCATTAATTGCTGCTCAAGTGTTTGTGTTCTTCGCTGCTGGCTTCGAGACCTCGTCATCAGCGACCAGCTTCACTCTCCATCAGCTGGCGTATCATCAGGACGTTCAGAAGAAGGCGCAGGAGGAGATCGACCGCGTGTTGGCGAAACATGATGGGAAGCTGTCGTACGACGCAGTCAAAGAGATGCACTACTTGGAGAACGTGTTCAAAGAAGGGATGCGAATGTTCCCCTCGCTCGGGTTCTTGTTCCGCCAGTGTACAAAAGCGTTCACATTCCCGGAGTTAAACTTTTCGATAGATGAGGGCGTGCGCGTGCTGATCCCACTTCAATCAATGCACAATGATCCTAAATATTTCCCTGAGCCTGAAGTCTTCCGCCCAGAGAGGTTCGATCCCGACGAGTACGACACTAACAACAAATATGTTTACATGCCGTTTGGGTCGGGACCCAGAGCTTGCATAGGTGAACGATTGGGTGCCATGCAGTCTTTGGCTGGTCTAGCGGCAATCTTATCCTGTTTCACGGTGGAGCCCGCTAAGGAAACGATCAAGCATCCTGTCGTGGATCCCCAGTCTTCCGTGGTACAGAGCATCAGAGCTGGACTACCTCTAATGTTCCGTGAAAGGAGTTAATAAGTAGATGCTTACGTCTTAATTGTATTAAAAATATCTAAATAAATTATATCTCTAAGTTTAATATTAA

>gene4794

TATAAATAAAATTAAATACGTGCCATTAATGTGATTTAAATAATTAAAAATGTCTTTATTACTAACTCAAGTGTTAAGCCCGCAGACAATAATATTTGTCGTAGTTACAATTATTTACACAATTCTGTGGTTCCGATATAAATTCACTTATTGGTCGAAAAGGGGTGTCGTCGGTCCCAAACCGGAGTTTATTTTCGGAAATATTAAAAAAGTGATTAAACGGGAGGAGCAGTTTTTCCAGCCGTACTATGATAACTACTTTAAATATAAACATTTGCCGTATATAGGCATGTACTCTTTCCATCAACCTGTGTTGTCTATACACGATCCAGATATAGCCAAGCTGATGTTGATAAAGGATTTTGATAGTTTCCAATCGCGTGGTACGTATGCCGGTGGGGTTGGAGACCCATTAGCGGCGAACTTGTTCAATATCTTTGGGAAAAGATGGAAAACCCTAAGGTTGAAGATGACACCAACCTTCACGCCTGGTAAACTGAAGACAATGTACCCGATCGTTGAGGATATAGCCAAACAGGCTTTGGACTATGTGGATGTGTTACACTCTAATGAAGAGACGGTCAATTTCACTGATTTCTATTCGAAGTACGCGATGGAGATTATCGGGAACGTTGGTTTTGGTGTGGAATGCAATGGTTTTGTAAATCCTAAGTCCGAGTTTTATATAAGAGGACATGAATACTTTGATCATCAGTCTCACTACTGGAGACTAATTCGCGCGTTTGCATTTTTCGCGCCAGACACGTTTGACAGTTTGAAGATCAGACGGATCAGTTCGAAAATTGAGAATTTCTTCTGTGGCATTGTCAAGAGGACAGTGGAGTACCGGCAGAAGCATTCTGTTACACGAAACGATTTCTTACAAACACTTATTGAATTAAAAAATGGCCACGTTGTTGATGAACAAGGAGAAGTCAAATATTCGAAAACAGACTTCCCATTCACAATGACTGATGTAGCAGCCAATACTATGCTGTACATGATCGCTGGTTACGAGACCTCAGCCACTACGGGCCAGTTTGCAGCATACCAGCTGGCTCTGAACCCTCACATTCAAGCCAGGGTCAGGAAGGAGGTGGACACGGTACTCGCCAAGTACGGAGGCGAGTGCACATACGAAGCTCAAAACGAGATGGTCTATTTAAACATGGTTTTGGATGAAACGATGCGGATGCACCCATCGATGCGTGCCCTATTTAGGAGATGCAACAAAGACTATAAACTGCCAAACAGTGACCTGGTCATAGAAAAAGGCACGATGGTGTTTATACCGATACACGCGATACATATGGACCCAGAAATCTTTCCCGAGCCAGAGAAATTCAATCCAGAAAGATTTTCACCAGAGAACAAAGCCAAGTTACATCCTTGCCATTGGATGCCATTTGGAGAAGGACCAAAGAAATGTTTAGGAATAAGACAAGGATACATACAGTCAAAGATGGCTTTAATAAAAGTGCTGCACAAGTACGAACTGATATTGGATAAGAGAACAGAGGTGCCAATGAAGATAAAGAACTCTTCCCTTGTGTATGCAGCTCAAGGCGGAGTCTGGTTGAAGCTCAGGAGTTTGGAGTCATAAAAACGTGAAATTGAATGGAAAAGTACATAACATATTTTCTTCTTTTGCGGAAGTCAAGATTACAACTTTAACAAATGAAGAGTCTTCTCGAAGGAAAGGCATGTTCAATATTTAGTTAGAATTTCCCTTGATAGAAAATCAGAACCGATCTGAAATCCGATTTGCTGTCAGCTACATTCATAGACAAAGCAGAGATAGAAAGAC

>gene5958

TGCGCCTGTTTAAATTATGTTATATTATTACATTGATATGCGATAATGCTCGAGCCGGTGCGTAGTTGCGATTATCAAATGTTATCCAAGCAAATTGATCGGAATGGATTTTTTCTTTTTATGGCTAGTAACATTCGTCGCGGGATTTTGGATTTTTAAAAAAATAAAGGAATGGCAGAACTTACCTCCTGGGCCTTGGGGTTTACCGATTGTTGGTTACTTGCCTTTCTTGGACCGTCATCAGCCGCATTTGACTTTGACTAAATTATCCAAGCAATATGGACCGATGTATGGCATTGGTATGGGCAGCGTGTATGCTGTAGTTTTATCTGATCACAAACTGATAAGAGATGCCTTTGCTAAGGACAACTTTTCGGGTCGAGCGCCGTTGTATTTGACGCACGGTATTATGCATGGAAATGGTATTATTTGCGCTGAAGGCAGTCTGTGGAAGGACCAGAGAAAGTTAATAACGATGTGGCTGAAAAGTTTTGGTATGAGTAAGCATAGCGTGTCGAGGGAAAAGTTGGAAAAACGGATAGCTTCGGGTGTCTACGAACTTTTAGAAAACGTCGAAAAGTCTCAAGGGAGCCCAATGGATTTGAGTCACATGCTGACGAATTCTTTAGGAAATGTTGTGAACGAAATTATCTTCGGTTCGAAGTTCCCCCCTGACGATAAAACCTGGAATTGGTTTAGACAGATCCAAGAGGAGGGGTGTCACGAAATGGGGGTGGCTGGTGTTGTTAACTTTTTGCCCTTTATTAGGTTCATTTCCGCATCAACAAGAAAAACAATTGAAGTTTTAACACGCGGTCAAGCTCAAACTCACAGACTGTACGCGAGCATCATCGATCGGCGACGAAAAGTTTTAGGATTAGCACCAGTGAAAGAAGCAGCATATTCCCTTCACGAAAACTTGTTTAACGAACACCCAGACGGCCATATTAAATGTATCAAGTACAGCAAACACGCGTCCGAACACGAAGAACATTACTTCGATCCGAATATTCTGATACCAACGGATGGGGAGTGCATCCTTGATAATTTCCTTCTAGAACAGAAGAGAAGGTATGAGAGTGGAGATGAAGGAGCTAAGTACATGACGGATGAACAAATGTTGTATCTTCTGGCTGATATGTTTGGAGCTGGTCTTGATACTACATCGGTGACGCTGTCCTGGTTTTTGCTGTATATGGCCCTTTATCCTGAAGAACAGGAAATAGTTCGGAAGGAGATTCTATCAGTGTACCCTGAGGATGGCGAGGTGGACGGTTCCAGACTGCCACACTTGATGGCTGCCATTTGCGAGACGCAGCGCATTCGATCGATAGTGCCCGTCGGAATCCCCCACGGATGCCTCCAAGACACTTTTATAGGGAACTACAGAATTCCCAAAGGTGCTATGGTGATTCCATTGCAGTGGGCTCTGCATATGGATCCAGATGTTTGGGAAGACCCTGAAGTATTTAGACCACAAAGATTCCTAGCGGAAGATGGAAGTTTGCTTAAGCCTCAGGAGTTTATACCGTTCCAGACAGGAAAACGTATGTGTCCCGGTGACGAACTCTCGCGGATGTTGGCGTGTGGTTTAGTGGCGCGACTGTTTCGGCGTCGGCGCGTGCGCTTGGCGACCGATCCGCCCTCTACGAAAGATATGCAAGGAACTGTCGGCGTCACCCTCTCACCGCCAACCGTTTCATACTATTGCGACCCTTTATGATTATGATATGATGATTTTTAGTATTTTAGTATTTCGTGTGACAAATAAAATGCTAGCAAACAATTGA

>gene5959

TTATTCGCAAGGCCACGCCAGCGGCGCGCCAGTGCGCAGACGTCGGTCGCACGCGATATACAATCACTGAGTTCTCGGCGTCACATTATCGCGATTTACGATCCGCGCACTAATGCTACGTTTCGTTTACTAAATAACATAAAACAGTTTATTTATATTTTAAAGACAATATAAACAGTCAAAATGATCACAGTTATATATAATTCCAAATTATTGTGGGGACTTTGGTGGGTGTCAAACACCGTCGTATCGCGTATTTCGCTACCGCTGCTGCTGGTGGCGGCCATGGCTTTGCTGGTGCTGCGGCTGGTGTCGTTGCTGACTGAGATGCGCAAGCTGCCTCCCGGCCCGTGGGGTCCTCCAGTCGTCGGCTACCTGCCCTTCCTTGGAGTGCGCCACAAGACCTTCCTGGAGCTTGCTCGTAACTACGGTGCCCTGTTCTCCGCACGACTCGGAAATCAGTTGACAATCGTTCTCAGCGACTACAAACTCATCAGAGAAGCTTTCCGGAGGGAAGAGTTCACTGGACGTCCCAACACCCCGCTCATGCACACTTTGGATGGTCTCGGCATCATCAACAGCGAGGGCCGCCTCTGGAAGAGTCAGCGCCGATTCCTGCACGAAAAACTGCGAGAATTCGGTATGACTTACATGGGAAATGGCAAGAAAATAATGGAAGCCAGGATTAAGAGCGAAGTTTACGAGCTTATCGCCAACCTGCACCGCGCGGAGGGAGCACCCATTGACGCGAACCCTCTCCTCGCTTTGGGCGTGTCTAACGTCATCTGCGGCATTACGATGTCCGTGCGTTTCAGTAACGGTGACGCTCGCTTCGAAAGGCTCAACAATCTCATTGAGGAAGGCATGAGGCTGTTTGGAGAAGTTCACTATGGTGAATACGTGCCTCTGTACAACTATCTTCCCGGCAAAGCACAAGCACAAGAGAGGGTGGCCAAGAATCGTGAAGAGATGTTTGCTTTCTATCAAACTCTGATTGACGAACATCGTGAAACTCTTGATGTCGATAACGTGAGAGACCTAATCGATGTTTATCTGATCGAGATCGAGAAGGCCAAAACCGAGGGCCGCGCCGGAGAACTGTTCGAGGGCAGGGACCATGAACTGCAAATTAAGCAGATTCTTGGCGACCTGTTCTCCGCAGGGATGGAGACTATCAAGTCGTCTCTCTTATGGATGATTGTATTCATGCTGAGGAACCCTGATGTGAAGAGACGCGTACAGGAAGAGCTCGATACTGTGATTGGTCGCGAACGTTTGCCTGCCATCGAAGATATGGCAAATTTACCTTATACCGAGACAACAATCTTAGAAACTCTTCGCATGTCCAACATTGTCCCCCTAGCAACAACTCATTCACCAACGAAGGATGTCCATTTGAATGGCTACAGGATCCCGGCGGGTTCTCAAGTTATTCCTCTCATCAACTGCGTACACATGGACCCCAATTTATGGGACGAGCCCAACAAGTTCAACCCCAGTCGTTTCATGGACGAAACAGGCAAAATCAGAAGACCTGAGTTTTTTATGCCTTTCGGTGTCGGAAGGCGTATGTGCCTGGGCGATGTGTTGGCACGCATGGAAATGTTCATGTTCTTCGCGTGCATGATGCACCAGTTCGACGTTCAGATGGAGACGGGTGATGCTCCGCCATCGTTGGAAGGCACAGTGGGCGCCACCATCGCGCCCCAGGCGTTCCGCGTGAAGTTCAGTCCGCGCGCCATGCCGGCCGCAGCCGCGCCCGACCACCCCCACCTGCGCCACGTCGGCGCGCACTAGACGCCCGCGCCGCGATACAACGCGCTACAATGCGCTACATCGCGCGTCTACGCGCGAACCCATACCCACCAGTATTCAATGATCACTCGTTTTAGAGCGAATCGAAAACCATTTTTAATTAGTCTTAATTATAAAATAACGACATCGCCACAGCGATGTTCGAGGCTGTGCATAAATAAAGTGTTCAATTACATACATAGTAGACCGAACGTTAGATATATTGATGTACCCATCGTTGAGATTATACAATGACGTTTGTGTGAGTTTGCAGTATTAGAATGTTGGTAGACTAGAATCGTAGGAAGTTCAGCAAATCACACCATTACACATTTGTATTTAAGGTTTACTTTCTAAGTTCTAGTACCTATACCAAAATAAAATGTAGAATCTAGGTCATTATTTATTAGTGTCACTTGCGTGAATGTGGTATGTTGTGTGTGATCTCATGATTGTTGCGTTACGGTAGGCTATATGTGTAAGGACTGTAAGTGTATTTGTATGTTAGTGTAATCTGTACCTATCAGGTATGAAGAAACATTCCATGGTAGAGTAAAAACCAGCAACTAGTCTAGAGCGATATCGTCGCTGTGTAATTGAATAACGCCTAACTTTAATGTTCTGTTTTGTACTAAATTCATAGTGTTGCAACCTAGTTGCCCTGTACTGAACTTCAGTCGCATAAGTCTGTAAGCTATATGAGCTCACCGTTTTCTGTTGGACTAATATAAGCTTTGTATATAATGCTCTGTTTTGTTGTAAGTTGTATAGATTAAGTAATAAACTAATTGTGAAAACATTAA

>gene5961

ACTCGGTCAAGTTTCGCCGCGTCCTCCTCCACTTACCAAAAAATATTTAATTTAAATATAAATACCACTTCTAATTTTAGTAAAATACTCCTTTAAGGTTTTGCTTAATTCTTGTTTCAATATTAGTTTTAATTTTTTTCCAAAATGTATTTCTATTATTTTCTTGTTTTTCTTGCCATAAACTTTTTGTCCAGCGCAATCTTGCGCATTTTTATGTATTCTACATTAGTATTTTGGATAATATTTAAAAATGTTAAATACTACAAAAGCCTTCCACCTGGGCCATGGGGATTACCACTGCTGGGAATACTGCCTTTCCTGAACCGCAGACCTCCTCATCTGGTGTACTTAAATATGGCTAAGAAGTATGGAGATGTATTCTCCGTGCAGATGGGCAGCAACCTAACCGTATGCCTTGGTTCTACGAAACTAATGAAGGAGTTCTTTAGTAGGAACGACTCCACCGGCCGACCTCACACTCCTTTGAACAACCTCTTGGGCGGACTTGGTGTTATCACAAGTGAAGGTTCTCTATGGAAAAGACAGCGCGTGTTCCTCCACGAGAAATTCCGCGCTCTCGGTGTCAAGCTCTGGCCCAACACACGTTTCGAAAGAGACATAAATGTTGAAATCGATGAGTTGATGATGGAACTAAACAAATCGGAGGGAAAATCGGTAAACCCAGCCCATGTACTTGGAAGACATATTCATAACGTCATATGCCAGCTCATGATGAGCTTCCGTTTCACGGCTGATGATGCGGAGTTCAGGTTATTTAATGAAAAGGTCTCCCGTGGTATGAGACTGTTCGGCCTAGTACTCATCGGCGAACACGTTAAGGCCTACTTGAAACTGCCAGGAAAAATGGCTGTCATAAACGAAATCAAAAAGAATTTGGCCGATGTGAGTAATTTTCACAAGGAGCACATTGTCGCGCGGATCCAACAAAGTGAACAGTCCTCGGAGCCACGCCAGCCAGCCGACTTGCTGGACTTTTATCTTGATCACCTCGAAGCTGAAAAACAGTTAATGACCAAGAACGAAAAGCATAACATATTCCCTGATGTTGAACCCGTTCAACAAATCGTACAGATCATGAATGATCTATTTTCTGCGGGAATGGAGACGTCCCTGACATCATTATTATGGACCTGCATCATGATGATGAAGCACCCTGAAGTTGCAGAAAAGGTGCGAGCTGACTTGAGAGAAGTCGTAGCACCTGGTGAGCGCGTGACTATGGCTCACCGCTTGCAACTGCCATATATCGAAGCGGTGCTTATCGAAACAATGAGGATGGTGTCAATCGTGCCGTTAGGAACGACGCACGTCAATACTGCGGAATGGAAAATTGGCAATTACACGATCCCCGCAGGTACTCACATTGTACCGTTGATCCACCGCATGAATATGGACCCCGACATTTATATGGAGCCCGAACAGTTCAGGCCCGAGAGATTTATTCGGGATGGAAAATTTGTAATCACAGACACATTCATGCAATTTGGCATAGGACAGCGCATGTGCATAGGCAACCTTCTGGCAAGAATGGAGCTGTTTCTTTTTTTCTCTAATATTATGAATAATTTTGAATTCCGCATGCCAGAAGGAGAAGATATTCCAGGTTTGGATGGAATTCTAGGTGCCACACATGCGCCGTTGCCATTTAAATTAGTCTTTAAGAAACTGGAGGCCTAAGTTCAAATTCTAACACGCTATAGTTACCTGAAGCATATCTCTTACGTATTCAATAGTAAGACTTTGCTTGTTTCTACTTATTTAGGCATCATTAATACTGATTTTTAACTTTGTAATTTTTAAGTTTACGCCTTTTAAGCGCTACTTTTGGTACCTTATTATTAATATTGAATGTTTTAGATTAAGATATTGTTTGTTTTTATAATAAGATTGTTTTGTGCATTTTTGCCATTGTTACCAAAATTTTTTGGATTAGCTATGTAACTTCATAATTTTAATTTCATCCGCCTTTACATTTTACCGTCTATGTTCCATGCATATTATACGATGTAAGTCTTAGATTTTACAAAATCATTTATAAGTTAGATTAGATCATTATTCACTGGCTTTACAACTTTCACTGCTTTGCTTCCTAGGAAATTGTATTGTTAATATCTTTAGCAGTAGTATTGTTCAATCGTGAACAAGGTCTATAAGGGTTGTGTGTACTATTGTACTCAAGTTGTATCTAAGTTAGAAAACGCTTACATTATTAAGTTCCTTAAAAAGGAATTAAATTTTAGTTAAGTTATTTAAGTTTTTAACTTGTGATCAAATATGAGTTCATTATAATGTTATTATGTAAGGTTA

>gene6094

GTAGGGCTTTCTGACTGAGTATTATTTTAGTAAGTGGATCTATTTATGAAGACCGGCCGGCGCGGAGTTAAAGATAGAGGTTAAACTGTTTATTGAGCCGTACAAACGCCGGAAATGTAAACAAACATTCGTTTATTCCCTGCGATTCAACGCGTGTCCGTCCATTAAGACGATTCGCAAATGCATTAAAGGGGATTGTAGTTGTAATTCACATTTTAGAATACAATGGTTAACGTAAGCAATCGATATACGGTTACATTCGAGTCACGGGCGCAGTACATCAACACACATGGCCTTGGAGGCATGGCGTCGCGCGAGCTACTCGGTACACTCGCGTAGGGCTTATCTTAGCGCGCCTTGCCGATATGCTTCACTTGATCACTGACCGCCGCCGCCACCGCAGGCGCTGCCGGCATCGTCGTACATATCGCGGATTGTATTGTTTACATACGAGCGAAATGCCGGCTATTTAAATATACAACGTGAACAAACACTTTTTATTCAAATCTAAGCAGTAAAGTGAACGTGATTCAAAATGTGGTTCTTTGGAATATTGTTGGTGATTTTGCTGTTTCTCGTCCTGTTATTGGCTGTTTTTCATTACACTTCCAAAGGAAGAAAGTATTGGTATTATAAAAATGTTGCCTACAGGGAACCTTGCCCGTTATTCGGAAACTTTGGAGCAACGTTTACGATGCGAAGAAGTTACACGAAAATGTTGCAGTTCTTCTATGATAATTATAGAGACCAGAAATATGTTGGACTTTTTCAAGCTCGGAGACCAACCCTAATGGTGATTGACCTGGACATTGCAAAAACTATATTCTCCAAAGAGTTCCAGTGCTTCAGTGACCGCGTGTCCGTGTCTACTGATACACAGCGTGAGCCTCTCCTGCGTAACCTGGCGAATATGGGAGGCGCGGAGTGGAAGGCCATGCGCCACATAGTTACTCCCACCTTCTCATCAGCTAAGATGAAAGCCATGTTCCCCTTAGTAGCCGAATGTGCACAAACACTGAAAGTTATTCTGCTGAAAGAATGTGAGGAGAATATAAATGTGCCTAGTATGATGACCAGGTTTACTACTGATGTTATCGGGAGTTGCGCATTTGGAGTGGACCCGGGAGCGCTAAAAGATTCCGAATCACCATTTCTGCAGATGTCACAAAAAATGTTTAAGATTGATCGCTCTACTTTATTGAAACGGTATTGTCGAACATTCTTTCCCAGACTGTTTAAATTATTGAATTTAAGATCTTATTCCTCTGATGTGGAGACATTTTTTACTTCTATAATCAATCAAGTTTTGGCTGAGAGGCGATCAGGTGTTCAAAGACATGATTTCCTCCAACTTATGTTAAATGAGCAGAAAACTGACTCCGGCTTCTTAATGACAGATGCTTTAATCACCTCCAATTCATTCATATTTATGTTAGCGGGGCTGGAGACATCAGCAACTACTTTGTCATTCTCTTTGTATGAATTAGCAAAGGACAAAGATATTCAAGATAGTGTAAGAAAAGAGATCATAGAGTGTCAAGAAAGGCATGGGGGTCTTAATTATGATGCTGTGGTTGCAATGCGCTCGGTGTATCAAGTAGTGGCGGAGACGTTGCGGTTGCACCCGCCCACACCTATGACTACGAGGCTTTGCACTTCTCCATACAAACTCGACGAAGACCTTTCTTTAAAAGTCAGAGACCCTGTACTAATACCGATCCATTGCATTCAGCGAGACCCACTATATTTTCCAAACCCAGATAAGTTTGATCCGGACCGATTTAAAGATGATTTAAATCCACCGGGCTTTATGGCTTACGGCGAAGGACCAAGGAGTTGTCCGGGTGCAAGATTCGCCCAGCTGACTGTAGCGGCTGGACTATCGGTGATCTTATCCACTTTCTCGGTGGAACCCTGCGAGAAAACTACTCCGACCATCGAGTACGATCCAAGAAGTGTCATGCTGAAGAACAAAGGAGGAATATGGTTGAAGTTCGTGCCTATCACTAACATAGATAAATAGATTAGATCCTTCCTAATTGATGTTATGTACCGAGAAGTGATAAACACATAGCTTCGGTGTTAGTCCTTAGTCAGCCATTAATCACATTTTCATGGCGTGAACAATGTGATCTAGGAAAAAGGGCGCTTCTGTATATGAAGCTAATGAGATATTCACAAATAGTTGAGGAAAATCGAATTGTAGATACTTATGTAGGCGAGTATTGGTATTAAAAACTATATGGATGGGAAAGTATTTGACCTTTTCGCCTTTGTACAATGGATATATTTTATATCTGTATCATACAAGAGGGCTGAAAGTTACAATACGGAACACTAGCCACGCAAGCCGTGGCTCCCACCTGTCGGTTTTTTTTTAAATACGGCATTATAAGACTTAATTACTGTTTAAGTTTTTAATTAGAACTCCTTTATTGTATGTATATTTTTCTAGCGTGTTATTCAATTGTAGAAAGAATTCTCTTATTTATTCTAATTTGTATTTAGACTACTTAGCTCTAGTTTTAGACTTGTAGATGTTCTGGTTCTGTCATGTATGAGTAATAATGTTCGTCTAATGAGAAATCACGTAGTTTACATTTATATATTGAATACACAATTAATAATAATTATCATTGTCTTTCTCTTCGGCTGCGTGATTCATGCGGATGTAGTTATCGTAACTGTTGTTATATTTCTATTGTTTTTAGGTTACGAACATTCCTTATGTATTTATGTAATTCGTACCAAGTCACCTCTGAAATGTAACGCAAAACATATTTGTAATTTCAAAGTTATCCTCCTATTGTTTTAATTTAAATTGGGAACATTTTGCGACTTGTGTACGAATTGTTAAGTAATATTCAAGGTAACAAGAGCAAAATTGTTATTGATTATTTTTACGAAAGGAGTACTTTATTTTTATAGATTTTAACCAAAATTTACAAACACATTGACACTAATTACGTAGTGTCAATGATCAGTCAAAAAAGTTTGGGACTGTAGATAGAAAATTACGTTTCAATTTATAGTTGCGAACATGTTTCGCAATCAGCCATAACGATTATCATGATGGACATTTATCAATTGATTCGATCCGCCATTTAATTATGGTGCGAACCGAACCAAACAGCCGATGATATCAATCAGCTGCTTCGCTAAACAACCGCTAAACGTGAACACTGAAGTTAATGGATCATGTAATTGCATAAATATAGCCTGCGAACATAGGGGGCCTTTTTATTTCAACGGTTTTATTTAACTTGCCCCAGTGTATGTAATTTAGTAACTGGGTAATTTCCTTAATAAAAAATAAATTATTCCTGCTGTTCAATACACAATCACATTTTCATTAATTGAT

>gene7471

TATCAGATAGGACGTTATCGAAATGTGGTTGTATTTTGGTGCTGTGGCGGTATTTTTGGGTGTTCTACATTTGTTCCTGAATTACAATGCGCAAGCTCGGATGATCAAGAAGCTGCCTGGACCGAAGGATGACTTTATTGTAGGAAATGCATTTAGAATTATCTGTGATCCAGTGGAACTAATGAAACTAGGAAGAACCTTCGCCAAGAAATGGAAAGGAATATATAGGATCTGGGTGTACCCATTTTCTGCTGTAATTATTTATAACCCTGAAGATATTGAGTTGATCATGTCCAGTATGAAGTATGGTGAAAAAAGTATGGTTTACAAAATTTTGCAGCCGTGGTTACAAGATGGACTCTTGCTAAGCAATGGCATAAAATGGCAAGAAAGAAGGAAGATATTGACACCAGCCTTTCATTTTAACATTCTGCGCCAATTCTGCGTCATCATAGAAGAGAACACACATCGTTTGATAGATCAGCTGAAGAAGACAGCAGGACAACCCGTGGATGTGGTGCCTATACTTTCTGACTTCACACTCAATTCTATCTGTGAAACTGCAATGGGCACTCAACTAAATGAACACTCGACGGCCACAACGTACAAAAAAGCGATTTATGATCTTGGAAACATATTTTATCATAGATTCATTAAAATTTATTTGTACCCTGAATTTATATTTAATGCTACATCTTTCGCGCGAAAACAGAGTAAAGCTGTAAAAACAGTACAAAGCTTTACTGAAAAAGTTATAAGACAAAGAAGAGAATATGTGAAAGAACATGGTTTTGATATCTTCAACCAGAATGTTGACGATGATGAAGTCTATGTGTACAAGAAGAAAAAGAAGACTGCCATGTTAGACCTCCTTCTGTCAGCAGAACAAGAAGGTCTCATTGATAAAACTGGTGTACAAGAAGAGGTTGACACTTTTATGTTTGAAGGGCACGATACTACTGCATCAGGACTTACTTTCTTATTTATGTTGCTAGCCAATCATCCAGAAATTCAGGACAAAGTTGTAAGTGAACTCAATGACATATTCGGAGACTCGCAACGTTGGGCATGCATGAACGACTTGCCAAAGATGAAGTACTTAGACCGGTGTATAAAGGAGTCACTGCGTATGTACCCGCCGGTGCACTTCATTAGTCGGAAACTGAACGACGAGACAGTATTAAGTAATCACACAATACCGGCCAGTACATTGTGTCACATACCAATATACGACCTGCATCATCGAGAGGATTTGTTCCCAAACCCTGAAGTATTTGACCCTGACCGGTTCCTACCAGAAAATTGTGAAGGAAGACATCCTTATGCATACATACCGTTTAGTGCCGGACCCAGAAATTGTATAGGCCAAAAATTCGCCATATTGGAAATGAAAATAGCTGCCGCAGCAGTTCTTCGAGAGTTTGAACTAAAAGCGGTGACGAAACAATCCGACATTGTATTCCTTGCAGATCTCGTTCTTAGGAACAATGGACCAGTGCGTGTCAATTTTGTCAAGAGAAATCAATTACAGAGAGTTGAGGCCAAGGACGTGGACGTTATAAAAATGTTCTTGTATTTATTTGTGACGGCGGGGTTTTTGTGTTTGTTGCACTTAATATGTAATTACAATGAAAAAGCGAGGAAGATTAGACAAATGCCGGGTCCAAAGGATTCGTTTATATTAGGAAATGGACCTGCTGTGATGCGATCCTCAGTTGGTCTCATGGACTTGGCGAGAGAATTGGCAAATACTAATTCTGGATTATACCGACTCTGGATACCGCCGTTTGGTGCTGTGAATATTTACAATGCCAATGATGTTGAGAAAATAGTATCAAGCATGAAGTTCAATGAAAAAAGTCAAGTTTACAGGGTCTTGAGGCCTTGGCTAAAAGATGGTCTACTTGTAAGCAAAGGGTCAAAGTGGCAGGAGAGGAGAAAAATCCTGACGCCCACTTTCCATTTCAATATTTTACGTCAATTTTGTCAAGTTTTGGAAGAAAATTCGCAGCGCTTTGTGACAAATCTCAAAAAGGTTGCTGGTCGACCAGTAGATGTAGCTCCTATCATTTCGGAATTCACGCTCAGTTCTATTTGTGAAACTGCAATGGGCACCCGATTGAGTGATTTGAGCACATCAGAGATGAACGCATACAAAGACGCGATATACAACTTGGGATACATATTCTACCAAAGATTTATCAAAGTATTCTATTTTGTGGACTTTATATTCAACCTTTCGCCACTGAGTAGAAGGCAGGAAGGGTATCTAAAGACGGTACATGGCTTCACGAAGAAGGTAATACAAGAAAGGACCGAATACATTGATAAGTTTGGTGTACCAGAACAGGATGTAACAGAAGATGACTACGTGTATAAGAGAAAAAAGAAGACTGCCATGTTGGATTTATTAATTACCGCTAAAAAAGAAGGATTAATTGACGATATTGGAGTGCAAGAAGAAGTCGACACGTTTATGTTCGAAGGTCACGATACCACGGCGAGTGGGTTGACTTTTTGCTTTATGCTGTTAGCACATCATAAAGATGCACAGGACAAAATCGTAGATGAATTGAAAGAAGTCTTGGGAGATTTTAAACGTCCAATCACAATTGAAGATCTACCGAAGATGAAGTATTTGGAACGTTGTGTTAAGGAATCGTTGCGGTTGTACCCTCCAGTGCATTTTATCAGTAGGAGCTTGCACGAAGATGTGATATTGAGTGACTACTTAGTACCAGCTGGAACATTCTGCCATATACATATCTATGACTTGCATCGCCAACCGGATTTATTCCCCAACCCTAACAAGTTCGACCCTGACAGATTCCTGCCTGAGAACAGTGTTGGAAGACATCCCTATGCTTACATTCCCTTTAGTGCGGGACCCAGGAATTGTATAGGTCAGAAATTCGCCATGATGGAAATGAAGATGGCTGTGGCAGAAGTGTTACGAGAGTTTGTGCTGGAGCCGGTCACTCATCCTGACGACATTCGGATCATTACTGACGTTGTACTCAGAAACGACGGGCCCGTCGAAGTCACCTTTGTAAAACGACAGTAA

>gene780

ATCTGGCACTAGCCGAGTGGAGACTTAATTTACATTGTAGTGGAGACGCGAACACATCGGTCGTCGATATGTTCGGGTTTATCGTAGAGTTGGTTCTTGTTTTCATAATATCTTGTTTATCTATTTTCTATCTTTTCGTGCGAAGTAATTATGGATATTGGAAGAAGCGTGGCGTTCCTTACGATGAACCAAAGCTGATTTTCGGGAGTCTTTCCTTCCTCATGAGAAGGAGTTTCTGGGATGTGTTCTACGATCTAGGTAAAAAGTACAAGAAGTATGATTATGTTGGAATCTTCATGTCGTGGAAACCTGCCCTCATGCTGCTCTCGAAGGATCTCTCGAAGAAAGTTCTTGTGAAAGATAGTGACAGTTTCCAAGATAGATACAGCTATTCTGGAATGAAAGATGATCCTTTGGGGTCATTGAATTTGTTCAGTATTAAGAGTCCAATGTGGATGCAAATGAGGAACGAGCTATCGCCGATGTTTACAAGTCTTCGTTTGAAAGGAGTCACGGAACTTATGAACATAAACTCCACGGAACTCGTGCGCAGGGTACAGAAAGATTACATCGAGAAAAATGAACCTGTTGACCTTAAGCAACTGTTTTCTATGTACACATCGGACACAGTAGCGTATACAGTGTTTGGCATCCGAGTGAGTGCTCTGAAAGAACTTGCCTCTCCCCTCTGGGACATCACACAACACATGGTGAAGTGGACCTTCTGGAGAGGATTGGAATTTACTATGGTCTTCTTCCTACCTGTTATTGCTGAGATCTTCAGATTGAAATTCTTCTCGCAGGCTGCTACAGAACATATAAGGAAGTTATTCCATGAAGTAGTAGCACAAAGAAAGAAAACTGGAGAATCCAATGACAAGGACCTCGTCAATCATTTATTAAAGTTAAAGGCGAACTTGAAACTCCCCGTAGGATCCGATGCAGAATTAGCCGATAACTTAATGATGGCTCAGGTAGCAGTGTTCATACTGGGATCTATTGAAACATCATCAACGACATTGTCGTACTTCCTAAATGAGCTGGCTTATCATCCTGAGGAACAGGAAAAACTGTACCAGGAAGTCACAGCGGCGTTAAAAGAGAGCGGAAAAGAAATCTTGGAATATGACGACTTGTTGAAAGTGAAATATCTGACAGCATGCATGCATGAAACGTTAAGAAAATACCCACCAGTACCTCACTTGGACAGAGTATGCAACAAGGCGTACAAACTGACAGAGAATATGACTGTGGAACCTGGTACTCCAGTCTACGTGAACGTGGTGGCCATCCACTATAACGAGGAGTACTACCCTGAACCTGAGGAGTGGAAGCCGGAGAGGTTCATCAACTCCACTGACAGTGATAACCATGATTTCGCGTTCCTGCCATTTGGTGAAGGCCCCAGGTTTTGCATAGGTAAACGCTACGGCATGATGCAAATAAGGACAGCAATCGCCCAGTTGATAACTAAATACAAATTTGAGCCCGCTGCGCCCAAAAAACTCGAGACTGATCCCTACAGCGTTATCTTGGGGCCCAAAGATGGCGGCAAAGTGAAGTTTGTACCTAGAACGTAAATTTATAATCTCTTTTACAGTTCAGTAAATAATTTGTTTTGATTGATGTGACTTAATCAAGTATTTAATTGATAATTGTAAGTATGATTAAATCAAAAATGTAAAAAATA

>gene7938

GTCATAGCGATCGAGACTACTCGCTTACAACTCCCGATGAAATTGTAACATCACTATTGTGTCAAAATTTTAGCGGGAAGAACGTCAATAAATTACGATGATAATCCTAGCCATCATATTTTTGGCGACCTGCGTTTTATGGTTGTATTGGGCCGCAAAAGCCCGACGCATGGACCAAATGACAGCGTCTCTACCGGCACTACCAACTTTACCTCTTATTGGCAATGGTTCCCTCTTCCTAGGAGATACACAACAGATCCTAAAAAATCTATGGACACTCGCAGACTTGGCGTTCAAACATAAGAACTCGGCGAAACTATGGCTCGGCCCTAAATTATACGTAGCCATTGGAGATCCTGAAGACGCCCAAGTAGTTCTAGAAAACTGTTTAGACAAAGATGTAGTGTATCGTTTCCTACGGCCTTGGCTTGGCCACGGACTGTTCGTGGCTCCAGTAAATCTATGGAAAGCGCATAGGAAAGTGCTACTTCCAGTTTTCCATAACAAAATAGTGGAAGAATATTTGGGCGTCATTGCGGAGCAGGCAGAAGTGCTCGTCGAGCGGTTGAATGAACGCGTTGATGCAAAGCAGTTCGATGTTCTCAAGTACATAACTGCTTGCACCTTGGACATCGTTTTTGAAACTGCAATGGGTGAACGTATGGACGTTCAGCATTCTCCAGACACTCCATACCTGCGAGCTCGCCATACTGTCATGGAAATCCTGAATATGCGGCTGTTTAAGGTCTGGCTGCAGCCAGACTGCATCTTCAACCTCACTCCCTACGCCAAGCAGCAGAGGGATAACATCGATCTTACTCACAAGTTCACTGATGAGGTGGTCCAAAAGAAGCGGTTGGAATACGAAAGGAAAAAAGGTGATCTTAAAGACCAAAATGATGGCAAACTTCGCGCAGTCCTTGACTTATTGTTTGGTAGAGAGATCGAGTTCACGGACGAACAACTACGGGAGCACATTGACTCAATCACCATCGCTGGTAATGATACTACGGCGCTCGTCATCGCATACACTCTCGTCCTACTTGGGATTCACCAGGATGCACAGGAAAGGGTGCTACAGGAACAAGAGTCAATATTCGGTTCGTCCACTCGAGGGTCTACGAAAGAAGATTTACAGCAGATGCATTATCTCGAGAGAGTGATCAAGGAGAGCATGAGACTGTACACAGTAGTACCAATCATCGCAAGGAATATAGACAGAGATATTTATCTGCCTACCTGTGGAGTAACAGTGCCAGCTGGTGCTGGTGCCGTGATCGGAGCTTTCGCAATCCACCAGTCAGCGGCATGGGGACCCAAACCTGAAGTTTTTGACCCTGACAGGTTTTTACCAGAAAGGTCTCAAAATAGACACCCAGCCGCCTTCCTGCCCTTCAGCTACGGCTCCAGAAACTGCATCGGACGAAACTTTGGCATGCTTATAATGAAGAGCATTATCTCAACGGTGGTGCGGTCATATAAAATAGAGGCGGACGAAATTGGACCACTCAAAGTAGAAATGCTCCTCTTCCCAATCAACGGACATCAAATTAAAATCTCACGGAGAACTTTATTAAAATAGGATATTGTATTATATTCTAGTGTATAAATAAAACTCAATGTACATACTTAGGACGCATCGTGCATACATGCTCGA

>gene7939

TATACCAGTCAGTTCTATGGTGTTTATAACATATAACTTAAAATATACAAATATTATAATCAAAATAACTAATTAAAAAATGTTGGTCGTTATATTAATATGTATAATTGCAGTTGCTCTTTACATAAAATTCAGATATTCGCGTATTCCTTTGTATAAGTTCGCTGAAACTTGGCCCACTTATGGTGATTTGCCAATTTTGGGACACACCCATTGGTTTATTGGAGGACCTGAAAGAATATTTAACAATATACGCACTTTGATGGATGCAATTAACGCCGAAGGGAAGACTGTAAGCTCGGTGTGGATTGGCCCGTCTTTGTATATGGTGTCATTCAATCCAGACGACGTGCAGAAGATTTTGGAGACTTGTCTTGAAAAGGACTACAGTTATAAGTTCCTTCAAACATGGCTTGGAAATGGATTATTTGTTGCACCAATTGATTTATGGAAGGTTCATAGAAGACTGATGCTACCGATATTTCACAACCGTATCATTGAGGATTACATAGAAGTTTTCGGGGAACAGGGTAATGTCCTTGTGGAGAGATTACAGGAACAAGTCGGCAAGCCGTCATTCGACGTCTATGAGTACATAACTTCATGTATGCTGGACATAGTTTTCGAAACGTCTATGGGCAAGAAAATGGACGTCCAACACAATCCTGACACCCCGTACCTGCGCGCGCGTAAGACAGTGATGTCAATCATTAACATGCGACTGTTCAAGGCGTGGTTGCAGCCAGACGTACTCTTTAATTTGACGTCTCATGCCACTGTGCAGAAAGAAAATATTGACGCCACTCATAAGTTCACTGATGAGGTTATACGCGACAAGATTGCTGACTTCGATGAAGAGAATTGCAAAGAAGGTCGAAAAGACATATTACAACTACTATTGGAGAGAAAATTAGCTTTCAGTAACATAGAGCTACGGGAACACATAGACTCGATCACCATAGCGGGAAACGACACCACGGCACTGGTGATTTCTTATGTTTTACTATTATTGGGAGAACATCAAGATGCACAAGAAGAAGTATATAAAGAATTGAAAATGATTTTTGGTGATTCTCAAAGAAGGCCAACCAAAGAGGATATAACGAAGATGGATTATTTAGACAGAGTTATCCGAGAAACAATGCGATTATACACAGTTGTACCTATAATTGGACGAAAAACTCAGAAAGAATTAAAACTTTCAACGTGCACAGTTCCGGCTGGCGTTGGTTGTGTCGTGGTACCCATGGTGTTGCATCTCTCCAAGAAACTTTGGGGTCCTGACGCTGACAGTTTCAATCCAGACCGATTCCTTCCAGAAAACTGCGCCAACAGACATCCATGTGCCTATATACCTTTTAGTTACGGTACTAGGAACTGTATAGGTCGATACTTCGGAATGCTCGCAATGAAAAGTATTCTCGCCAATATATTAAGAAGTTACAAAGTATCATCACAAGCGTTCGATCGGCTCAAGATTGAGATTCTACTCTTCCCAGTCAATGGTCATCAAATAACTTTAGAAAAACGACTGTAACACATTAATAGATAACATTCATGACTTTATACTTACTTAACCTTTCCTAATTAAAAAAAAGAACATAG

>gene8165

CTCAGTGATAGCGCGTACGACCACAACAACATGCTTCTATCAGTGTACATACTGTGTACATTGTTGCTGTGCATATGTGTGCTGCTATGGAGCCTGGAGCGACGCGAGCGCAACAAATATAGAGGGGTACCGACCTTGCCACTCTTACCTTTCATCGGGAACATACATCAAGTTATAGGAGATACACAACATTTGTTTCACATAGTGGAGAATATAACCGAAATTTGTGAAGAAAAGCAGTCACTTTTTGTGTTTTGGTTGGGACCTTATCCTATTTTAGCGGCCCAACATCCAGATGACGTGAAGATGATCACAAACTCCTTCGTGGAGAAGCCGTATTTCTATGACTTCGGTAGAGCTTGGCTCGGAGACGGACTCATCACTGCACCAGGTTACATATGGAAACAAAGCATCAAGAAATTATCTGGCACATTCACCAGCTCCGTGGTGGACGGCTACCAGGATGTGTTCAATGCTCAGGCGCAAAAGTTGGTGATTAACTTGAAGATTAAAGTGGGGCAGGAACCATTCGATGTTATGCATACGTTGGCGTTTGCCACATTGGAAACTATATGCCAAACGGCTTTAGGAGTTTCAAATATATCAGAAAGTATAGTCACAGAGGAGTACTATGATGCATTTAACCGTTGCCTTGAGCTGATGCTATATAGGGGCGTGAACATTCTGCTGCATCCTGACGCCATATACCGGCTCACTCCTGCACATAAGGAGATGATGAAGTGCGTTGCTGCCCTGCACAAAGTATCTGATAATGTGATGAAAAAGTTACGAGCTGAACAGAACAACGTGAAGACAAGTGAAATGAGAGGAAACCAGAATCAAGGTACAAATAGAGGGCCTAAGTTTAAGGCTTTCTTGGACATTTTGTTGGAGCTGAGAGAAATAGACCCGAACCTTACGGATCATCAGATCAAGTCGGAGGTGGACACCATCATCTTAGCAGGACAGGAGACGGTAGCCACTGCCTTTTTCTACACCCTGCTGATGATTGGGTGTAAGCCAGGAGTCCAGGAGAAGATGTATGCAGAGCTGTACAGTATATTCGGGGACAGTAAGCGTCCGGTGTGCAAGGAGGACCTCGCCCGAATGAGATATTGCGAGGCTATCATCAATGAAACTCTACGATTGTACCCACCAGCTCCTGGCGTCATGCGATATGCTGACAGGAATCTGAAACTTAAATCCTTCACAATCCCGAAGGGCACAATATGTGCTATTAATTTCTGGGGAGCAGGGAGATCGTTGCAAGCTTGGGGTCCCGACGCCAAAGTCTACAGGCCGGAGCGGTGGCTGGAAGACCAGCCCCCTGGCAACCCTGCGGCATTCCTACCCTTTAGTTATGGGAGACGAGCGTGTATTGGTAAGAAATACGCCATGGCGATCTTAAAAACAATGCTGGCGCATTGTGTCCGAGAGTTGGATTTCGCGTCGCAGGCTGACAACTTAAAGCTGAAGATAGACTTGGCTCTACGACCAGTCTCCGGCCATCTCATACAAATTAAATTGCGGGAAACCGAAAACCGTTGCTTGTGAAGTTGTGTGATACATAAATGAGATTATTGTAATAAATGTACCGTATTCGAGTTATTTGTGGCAGATTTTAAAATAAATTA

>gene8361

CCGCGCAACGGTACACCCTAGGATCTCGATAGCGGCGTGAGCGCAGTCGTCGTCGCGGCCTTAACCGCCATGGGTCGTTCTCTCCGCACGTTTGCGGCCTACTCACAGCCGTTCGTAGTGAAAAATGCTCGATATGCAACCAGCGGGTGCCCGTTCTCAAAACGGCAACGTTCCCAAATTGCCCCCACCGCGGAATTAAGTGAAGAAATTTTCTCTAATGCCAAACCTTACTCCGAAGTTCCTGGACCAAGACCAATCCCGATACTGGGGAATACGTGGCGGATGGTGCCTATCATCGGGCAGTTCGACATTTCGGAATTCGCGAAAGTGACCCAACATTTTTTGGACACTTACGGGAGGATTGTGCGCTTAGGAGGGTTGATTGGAAGACCGGATCTGCTGTTTGTGTATGATGCGGATGAAATAGAGCGGATGTACCGACGGGAAGGGCCAACGCCGTTCCGTCCTGCGATGCCTTGCCTCGTCAAATATAAGTCTGAAGTCAGGAAGGATTTCTTCGGAGAACTGCCAGGCGTTGTTGGGGTTCACGGAGAGCAATGGCGCCGTTTCCGTTCGAAAGTCCAGCGTCCCATACTCCAGCCACAGACGGTGAAGAAGTACGTACGTCCCATAGAGATGGTGACAGAAGACTTCATCAAGTACATGGAGAATGCTCGCGATGAGAATGGAGATCTTCCTCATGAGTTCGATAATGATATCCACAGGTGGTCGCTTGAGTGTATCGGCCGCGTCGCCCTCGATACGCGGTTGGGTTGTTTGTCTACGGACTTGACCAGCGATTCAGAAACACAGCGCATAATTGATGCGGCAAAATTTGCGCTTAGAAATGTTGCTGTGTTAGAACTTAAGGCGCCGTATTGGAGATATATACCGACTCCACTTTGGACCAGATATGTTAATAATATGAACTTCTTTGTTGAGCTTTGCAGCAAATACATAAATGAAGCTCTGGAACGGCTAAAAACCAAAAAAGTGACCTCAGAGAACGACCTGTCTCTTCTGGAGCGTGTTTTGAGAAGTGAGGGAGATCCAAAGATCGCTACAATCATGGCACTTGATCTTATTCTAGTTGGAATTGATACGATTTCAATGGCAGTTTGCTCAATCCTGTACCAAGCGGCGACAAGATTGAAGCAACAGGAGAAGATGGCGGAGGAGATAAGGAGAGTGCTACCTGATCCCAGCAAGCCTTTGAGTTACGCGGACTTGGATAAACTGCATTATACCAAGGCGTTTGTTAGAGAAGTATTTAGAATGTATTCTACTGTTATCGGAAACGGCAGAACACTTCAAGAAGATGATGTCATTTGTGGCTACCATATACCTAAAGGCGTTCAAGTGGTATTCCCCACAATCGTGACGGGTAACATGTCCCAGTTTGTCTCCAACCCTGAGGAGTTCAAACCCGAGAGGTGGTTGGAGAGTGACGGTCGTCTGCATCCCTTCGCCTCCTTACCATATGGATTCGGAGCCCGCATCTGTCTTGGTCGAAGATTTGCTGACTTGGAGATACAAATTCTTTTGGCAAAGTTAATCCGTCGATATCGTTTGGAATACCACCACGAGCCGCTCGACTACGCTGTCACCTTCATGTACGCACCGGACGGACCCTTGCGCCTGCGCATGATTGAACGATAAGCCACAAATGTAAAAAATAAACAATAAAATTAATTATTAATAAATACAGGTGTAATTATATTTGCATATTTCTGTCATAAATGATAGATATCCACTATGATTGTTGACTTTGTTAAAACATAATATATAACTAAATTAAATAAAAATAGTTGCATAACATTTGTTAAAATGTTTATTGATGTTTGATTTGAGGCTGTATAAAGTTTTAGCTGCCTCTACATTTTCATAGAATATTGTTATTATAACTGCTAATAGAGGTATTAAACCTACTCAAAAGAAAAAAAAAATCTAAATTAATTTAAAATAAGGTGTTTTCGGTAATTTAAATAACGTTATTGTAAATTAATGTAAGTTAAAAGAT

>gene8362

TAGAGGGGGTCAGCCGAGTTCATGCTATTGTGACTGATTACGTGTCAAAGGGCTGTGTCAAGTGATAAGGACTTTTTGGATATTTCTTCTGTTTTGGCAAAGAGCCAAGTACCTACCTACTAAATAATGAGCGGTCGAATCAGTGTGGTAAAATGGCCGCTACGAAATGTCCGTGGCGCTCGACTTATAACAACGAGTACCCAAAACACAAAACCGACTGAGAGTGCATTAGAAATCAGTCAAAAACCAGCTCAAACATGTCCGGTAAAAGGATTTCGCGCGAGATCCACTCATGCGGCGATCGACACGTCCATTTTTGACATGGCTCCATCTGTCAAGTCGTGGAATGAAGTACCTGGGCCACGTCCATTACCGTTCTTAGGCAACACTTGGCGATTCACACCTTATATCGGAGGATATTCAGTAGAGCACATAGACAAAGTGTGTCTGTCTTTGCGAGCCGAATATGGAAACTGTGTGAAGGTCACTGGTCTGCTTGGAAGACCTGATATGCTGTTCGTCTTCGATGCCAGTGAAGTGGAAAGAGTGTTCAGGGGTGAAGACGCAGCACCGCATAGACCATCGATGCCATCTTTGAACTATTACAAACACACATTGAGGAAAGACTTTTTTGGCGCTGAAGAAAATTGTGCTGGGGTTATTGCAGTCCATGGTGATTCCTGGTCTGCCTTCCGAACCAAAGTATCTCGGGTAGCGTTGAGCGCTGGTGCAGCAGCTCAGTACACGGTACCTGTCTCTGAAGTAGCTGATGCTTTTGTCCAAAGAGTACGACAAATAAGGGATGATAATTCAGAAACGCCGGGCGATTTCCTGAATGAGGTCCACAAATGGTCTTTGGAGTCTTTAGGACTAATAGCTCTGGACACAAGACTGAACTGTTTTGACGCGTCAGAAGACTCAGAGAGTATGCGCCTGATTCACGCCGTACAGACTTTCTTCCTAAGTGTGGGTCAGCTCGAACTGCGCGCCCCCTGGTGGAGGTTATACCCAACTAAAATGTTCAGACAATATGTTAAGGCATTGGATACAATCCTTAGCGTAACTCTCAGTCACGTGGAGAAGGCGCTAAAGGAATGCGAAGCTAACGGCAGCAGCAAGTCACTGCTGCAGGACCTGGTCGCAGCAGCTGGGCCAAGAGTGGCAGCGGTAGCAGCACTAGACCTGTTCCTAGTAGGAATTGACACAACGTCAAATGCAGTAGCTTCAATTCTATATCAGCTATCTCTAAGGCCGTCAGTACAAGAGCGGTTGTACGAAGAAGTTAGCAAAGTTCTTCAAGGTAGACCAATGAAGCCAGGAGACGTTAATCAGATGCCGTATCTGAAGGCATGCGTTAAAGAAGTTATGAGGATGTACCCTGTTGTCATCGGTAATGGAAGGCAGCTGACTAAGGATACTGTCATTTGTGGATATAATATACCTAAGGGGACTCAAGTAATATTCCAACATTACGTGATGGGGAACAGTGACGACTACTTCTCCAATGCGTCAGAGTTTAAACCAGAGAGATGGATGACCAGGTCTCCGGCACAGAAACACCACGCCTTCGCCTCCCTACCATTCGGCTACGGCAAACGAATGTGTCTTGGTAGAAGATTCGCTGAGTTGGAAATACATACAGTAATTTGCAAAATGATACAAGCCTTCAAAATGGAGTATCACCACGAACCAATGGATTACCACATTCATCCAATGTACACACCGAATGGACCTATCAGAATAAAAATGACGGATCGGTAGATCTACTACTTGTATGTTACTTAGTTTTAGCATCGTATTGATCTAGGAATGCTAAAAGCAAGCTAGATTCAAAGCTCAATTATTTAGTTGCAATAAAATTATATTAAAATTCTTTTGAGACATTTTAAATTGACTGTGCTTAACCCGCGGCAGTTGGCAGTCGAAAACTTCATCGGCGCCCTCTGGTATGTCGTGCAATGCTGCTCATGAATACGAGCCCCCAACGTGACACGAAACTAGTCGAGTTGCCTCGTCAAACAGTTTCGTGAAAAAACCGTTAAACAGTTATTTTTTTGTGTTATAGTTTTATTAAATTTGAAGTAATATAATGGTTTTTTCATCTGTCCTAGGGTAGATTATAATGATAAAAATATTGTGTTTGTATATTTTTGTTCACATTCTATTTCTCAGGATTAATTTATTACTGTATACCCAAAACTAAACTTAATCTGATTATTAGTATGTATGACATGACTTTCAGGTGATCTTGTGTTCATTAAGTTACCATTTTATTGTAACATATTATGTAAATATTGTATTTCAAATTTATCCTCGGGATATAATAATGTT

>gene8531

AACACTCTAACCATGATCACGTTGTACTTGTTGCTCGCCATACCACTGGCGCTGTACAGCATCTACCTCATATCAATAAGAAAATACAGATACTGGGAGAAAAAGAAAGTGCCGCATATACCACCAAAACTAATTCTGGGAAATTTCTCCGAACATATTCTTCAGAAGAAATACCTTGGACGTGTTGAACAGGAACTATGTAAAATGTTCCCCGAAGAACCATACGTTGGTTCCTACTTAGGCACGGAACCAACGCTTATTATACAAGATCCAGAATTAATCAAGGCCGTCATGACAAAGGACTATTATTTCTTCAGCGGCCGTGAAACATCAAATTACTCGGAAAAAGAATTGGCAACTAGAAACCTTTTCTTGAGTTCTGGTGACAAGTGGAAGGTGCAACGTCAGAACCTCACGCCTTTATTCTCATCCGCCAAGATGAAGAATATGTTTCATTTAGTTGAGAAATGTGCGCGTGTCTTCGAAACTCTGATCGACCAAGAAGTACAGAAGGGTAAAGATATTGAAGTGAAAGCCCTTATAGGTAAGTTCACAATGGATTCAATTGGAAACTGTGCTTTTGGTGTTGAGACTCGCACAATGGTGAAGACTGAAAATAATCCATTCACGGAAATCGGTAATGTCATCTTTGATACTAATTGGTACAGAGCATTCAAGCTTGTCGCAAGAGGTATCTGGCCAGCCATCTTTTATGGTTTGGGATTCAAATCACAACCTGTTGACGTTGACAATTTCTTTACCAATTTGATGACTGAAACCTTTAAAGGGCGAGATTACAAGCCAACAACGAGGCACGACTTCGTTGATCTTATGTTGAAGTTGTATAACCAGAAAACGGTGACCGGTGACAGTATGTCGAACAGGAACGGTGATGCATCAAAGAAAGTGACTTTAGAAATTGACGATGAATTTTTGATATCCCAATGTTTCTTGTTCTTCGCTGCTGGCTTTGAGACGTCGGCAACTACGTTGAGTCATACTTTGCTTGAATTGGCGAAAAACCCCGAAGCTCAAGAGCTGGCGTTCCAAGATGTGGACAACTATATGCGTCGCAATGATAACGTATTAAAATATGAGTGCGTTACTGAGTTGCCTTACGTGGAAGCTTGTGTTAATGAAGCTCTTCGTCTGTACCCAGTGCTGGGAGTCATAACTCGGGAAGTCATTGAGGATTACACATTCTCAACAGGATTAAAGTTAGAGAAAGGGCTCCGGGTACATTTGCCTGTGTACCACATGCAACACAACCCTAAGTATTTCCCCGAGCCAGAGCAGTTTCGTCCGGAGCGGTTCCTGGGTGACGCTAAACAGAATATTATACCATATACGTACTTTCCTTTTGGCGAGGGACCTAGACTTTGTATTGGAATGAGGTTCGCAAAGATGCAGATTACAGCGGGAATTATAACTTTACTGAAGAAATATCGAGTGGAACTTGCTCCAGGAATGAATGAAAAAGTTGACTTCGAGCCTCGATCTGTGATCACCGCGCTAATTGGAGGAATCCAACTGAAATTCGTACCACGAGAGGGTTGGGAACAGCGTCTATTCAAGGCTGCGTCATAAGTATAGGTTAGTTTTATTTATGTAAACGATATTCATTCATTTATTCATTAACTTCTTCACGACTGTTCGTTTGTCGATTACGAGACCGTAATGTGGACACATTGCATGATTTAATGAATTCATGCGTTCGTCAAACACAATAGGCGGTTTTAACGTTTCCGCTTCATGGACCGTTTTTTTTTTTATTCTTAAGCAAGATGCACGCATTTGTAGGCCGTTTATTGTTTGTTAAGGATGCATGCATTCGTCAAATGACGCAAAGTGGATAATTATCATAAGGATGCTTCAGTTTAAACCCTTTTTAGCTTGCTGACAGACATACAGACTGTCTGTCTATCACCAGGCTATATTTCACAAATTGTCCTAATTTTCACAGATAATGTATAACAAATACTTGAAATAAATGTGTAAAACAGTTTTCCTTAC

>gene8796

TTCTCAGTGCTCCACTCACTCGTCGGCCGCTACTCCGCCGCCTCGCACTCAACATGATCACACTCGCCATCGCGCTAGTTGCGCTCGTCGCACTCTACCTTTACGGCACGCGCAGCTTCTCTTACTGGCAGAAGAAAGGAGTCAAACATGACAAACCAATTGCTTTCTTCGGGAACAACGCAGCAGCCTACCTCATGAAAAAAAGTATTACACAGATGGCTGTCGATGCGTACTGGAAATATCCAAAGGAGAAGGTAGTAGGATTTTATCGATCTACGTCTCCAGAGCTCGTGATTCGTGATCCAGAGATAGTAAAACGCATACTTATAACCGACTTCCAATACTTTTATCCCCGAGGCTTAAACGTGCACAAGACAGCCATTGAGCCTTTGATGCGGAATTTATTTTTCGCCGACGGCGACCTCTGGCGGATGCTGCGGCAGCGCATTACCCCTGCATTCACAAGTGGTAAGCTGAAAGCGATGTTCCCACTGATCGTGGAAAGGGCTGAGAAGCTGCAGGCGCGCGCTCTCTCCGCTGCCACCGCCAACCGCGAGATGGACGCCCGCGACCTGATGGCACGGTACACTACTGACTTTATTGGCGCATGCGGTTTTGGTCTTGATGCTGATTCGCTCAATGAAGAAGACTCCGCTTTCAGAAAGTTAGGAGTATCCATATTTGATTTTAAACCAAAAGACGTTTTTGTAGCCATGCTTAAAGAAATGTTCCCGGAGGCGTGTAAGAATTTAAAATATTTAGAAAGATTGGAAAAGCCGATGTTTGAATTGGTTACAGCAATACTAAGACAGAGGAATTACGAGCCAGTGGGTAGGAATGATTTTATTGATTTGTTGCTTGAATGCAAAAAGAAAGGAAAAATGGTAGTGGAATCTGTAGAAAAAGTAAATCCCGATGGCAGTCCGGAGACAGTTTCCATGGAGTTGACGGATGAACTCATTGCGGCGCAGGTGTTTGTGTTCTTTGCAGCTGGTTTTGAGACGTCTTCCTCTGCGACCAGTTACACACTGCATCAGCTGGCGTATCACCCGGAAGTCCAGAAGAAGGTGCAGGACGAGATAGACAGGGTGTTGGCGAAACACAACAATAAGCTCAGCTACGATGCTATCAAAGAGATGACGTACTTGGAGTGCGCCTTTAAAGAAGGCATGAGAATGTTCCCATCCCTTGGCTTTTTGATGCGTCAATCCGCGCGCAAATACACTATTCCAGAGTTGGACCTCACGATAGATCCAGACGTAAATGTGGTAATTCCCCTGCAAGCCCTGCACAATGACCCGCAGTACTTCGAGGAGCCGCACGTATTCCGTCCTGAACGCTTCCTGCCTGACCAGGTGGACGAGAAAACCAAGTTCGTGTATCTTCCCTTCGGCGACGGCCCTCGGGCTTGCATAGGGCTTCGACTGGGTCTGATGCAGTCGCTGGCGGGGCTGGCGGCGCTGCTGTCCCGCTTCACGGTGGCGCCGGGTCCCTCCACGCTCCGTTATCCGGTAGTAGAGCCCAAGTCCGGCATCGTACAGAGCATAAAACACGGCCTGCCGCTTTCCTTCACAGTGAGGAACCCTGAATCGGTGGCGACGACATTGTGATGGGGAGTACTTCCCGGGTAGTGGCAGTGATGTACACGATGGACAATGTGATTTGATGATTATTTATTTAGTAGAGGATTATTTTAATTATATTTTTATTATCTGTCTCCTCTTTATACTGAGTTGGTGACCCGTTCGGTTTGGGCATGTCCGCCAACACCGCGCACCTGAATTATTGGCCTGTTTTAGTTTTAGTTTTATAATTTACCTTTTATCTCT

>gene8798

TTGGATTGAGGCATTCGCTAGTCAGCAGTCAGCCAGCAACATGATCGTAGTAGCGCTCGTACTTGTGGGAGTAGTGGCCCTGTACCTGTACGGGACTCGCAACCACAACTACTGGAAGGAAAGAGGAGTCAAACATGACAAACCGATACCAATCTTCGGAACCGACGCGAAGCGGTACCTTCTCCAGAGCAGTATCGCACAGAGGGCAGCTGACACCTACTGGAAGTATCCTGGAGAGAGGTTCGTGGGGTACTACCGCGGCTCTATCCCTGAGCTGGTGATCCGTGACCCCGACCTAGTCAAACGAATCATCACCACAGACTTCTCCTACTTCTACTCCCGCGGCCTGTCGCCTGCACACAAAGTGATTGAACCTCTGCTACGTAACCTATTCTTTGCGGACGGTGACCTCTGGCGTCTGCTCCGGCAGCGCATGACTCCTGCATTCACAAGTGGCAAGCTGAAGGCGATGTTCCCATTGATTGTAGAGCGAGCTGAGAAGCTGCAGGCACGCCTACTCGATGCCGCCGCCGCCGGGCGCCCGATAGACGCCCGCGAGCTCATGGCTCGCTACACCACCGACTTCATCGGTGCCTGCGGTTTTGGTCTCGACGCCGATTCACTTAACGATGATAGTTCACCCTTTAGGAAACTCGGCATGAAAATTTTCAAGATAGACTTAAAGCGCTGTATCGTCATATTCTGCAAAACTATTTTCCCCGACTTATTTAAGACCCTTAAATTTTTGGGCCAGGAACTGGAAGACGACATTTTAACTCTCGTAAATCAAATTCAAAAACAAAGAAATTATAAGCCGTCCGGCCGTAACGACTTCATTGATTTCTTGATGGAATGGCAACAGAGAGGAAAGATCCTGGTAGAATCCCTCGAAGAAATGAACCCAGACGGCACACCCAAATCTGTGGAACTGGAGATGGATGACGTACTGGTAGCAGCGCAAGCGTTCATCTTCTTTGCGGCTGGTTTTGAGACTTCCTCGTCAGCGACGAGCTTCACGCTGCACCAGCTTGCTTATCACCCAGAAGTGCAGAAGAAGGCGCAGGCTGACATCGACCGAGTGCTCGCCAAACACAACAACCGCCTCAGCTACGACGCGGTCAAGGAGATGACCTACCTCGACTGGGTGCTGCAAGAAGGCATGCGCATCTTCCCGTCCTCCGGCATGTTACTGAGGCAGTGCGTGCGCCCGTACACCATCCCAGGGACGGACATCACCATCGACAAAGGTGTCAAGATAAACATCCCTCTAGTTGCACTGCACAACGACCCACAATACTTCGACAACCCTAAAGAGTTCCGCCCCGAGCGCTTCGAACCAGAGGAAGTGGCCAAGAGACACAAGTTCGTGTACCTGCCGTTCGGAGATGGACCTCGCTCTTGCATCGGTGGTCGACTAGGTCAGATGCAGTCGCTGGCTGGATTGGCGGCAGTGCTGGCCAAGTTCTCAGTGTCACCGGCGCCGGAGACCAAGCGCGAGCTGGAGTCCGACCCCACCTCCAGCATCGTGCAGAACATAGTGGGTGGCATCCCGCTCATGTTCCACGCGCGCCACGCTCCGCCTGCGTAGCTGCCTAGTCTAGTTACTAACCATTAGTTTGTATTGCGACTGAAACTATTTATTTATCTTGTTTAGGTTTAAGTTGTAGTATAGTGATTTTCTCTTATGCTTCGACTAAAGGTCACTAAAAAGACGACGTACAAAAGTAAACGGTACAAGTGTGAGATTTGTGGCAGACATCACAGGCAGTCGGAGGGAGTGCCGTGATCAGTTTCTTGTACTCGACTTTGTCTTCTGTTTAGATTGGAGATCGAAATTTAAAATATACGTAGGATTTAACATTGTGTAGCTGTGATACCAGTGTGTATAAATGTAGACTGTGTGTTTTGTTGTTGTAAATGGGTATATTACTAAGATAATTCAATTTTATTGTTCTCAATGTTTTAATCTCTTTCGATGAGGTGTTTTATGTTAATGTTATCTTCTTGAAAACATAATTTTCGAAATACACTTTTTAATTCA

>gene9047

TATGTATGTACCTACACCGCCTAAACACCAACTCACTTAGAATTGTTAAGATAACACACACGAGGAGTCGAACAAACTATCATAGCGTGATCCTAATCAAGTTTTCGGTTTGTATATAAAATCGTCGAGTTATTTACAAATCATTCATAAGTGTTCGGACCGAGTGTTTGGCCAGTCACAACCATGTCGGCCTTATATTTCCTCGCAGCAGTCCCAGTGTTAGCTTACGCTATTTATTATTATTTTACGAGGACATTCAACTATTGGAAGAGTAGAAATGTTCCCGGACCAGAACCAACTGCATTTTTTGGAAACATCAAGGAATCGGTTGTTCGCACAAAAAATATTGGCATTGTAATGCATGACATCTACAAAGCATTTCCAAATGAAAAAGTGGTCGGTATGTTCAGGATGACTTCACCTTGTCTCCTTATCCGAGACCTGGACATAGTCAAACATGTCATGATCAAAGACTTTGAAGCGTTTAGTGATCGTGGAGTGGAATTCAGCAAAGAAGGATTGGGACAAAACTTATTCCACGCTGATGGAGAAACATGGACAGCTTTGAGGAATAGATTCACACCCATTTTCACAACTGGTAAATTGAATAACATGTTTTACTTGTTAAACGAAGGAGGTGATTCATTTATAGAGTACTTTATTACTGAATGCCAAAAGAAACAGGAATTTGATATCCAGCCTCTCCTCCAGACATACACCTTGTCCACGATCTCTGCCTGTGCATTTGGTATTAGTTATGACAGTCTTGATGATAAATTGGAGACTCTGAAACTTGTCGACAAATTATTTTCATCTCCAAGTTATGCATTTGAGTTGGATATGATGTATCCAGGACTCCTGAAAGCACTCAACCTTTCTTTATTCCCTTCATCCATACAAAAGTTCTTCGACAATCTAGTGAAAACTGTTATTGCGCAAAGAAATGGTAAACCATCAGGTCGACATGATTTCATGGACCTTATCTTGGCACTCCGTGAAATTGGAGAAGTCTCAAATGCAAAACATGACTCTGCGAAGCCTGTGGAAATAACACCCGAGGTGATGGCAGCACAAGCATTTGTATTTTATGCCGGTGGCTATGAAACCAGTGCTACTACGATGACGTTCATGCTTTACCAACTAGCAATGAATCCAGATATTCAAAATAAGTTAAGGGCAGAAATTGACGATGTTATACAAGCAAATAATGGACAAGTAACATACGAGTCTATTAAGGAAATGAAGTACCTAAACAAAGTATTTGATGAAACTCTACGAATGTACTCAATTGTGGAACCTCTTCAGAGTAAAGTGGTAAGGGATTACCAAGTACCCGGTACTAACCTGACTTTGGAAAAGTACACCATTGTACTTATATCCCCAAGAGGCATACACTACGATGAGAAATATTATGATAACCCTGAACAGTTCAACCCTGACAGATTTTACCCGGAGGATGTGGGCAAGCGCCATCCGTGTGCTTACTTGCCATTTGGACTTGGACAGCGAAACTGCATCGGATTGAGGTTTGGCAAACTCCAGTCACAATTATGCATAGTCAAGTTTCTGTCCAAGTTCCAAGTGGAGCCATCAAAGAATACTGCAAGGAAGTTGGAATTTGAACCCTGCCGTACTATTATCGGACCCAAAGGAGGAATTCGTTTAAATATTGTTCCTAGAAAGTTGAAGGCTTGAAGACTTTCAAACTGTTGACATTGTCATAAATTATTACAGCAGGCCAGCAAGTTAACTTACACCAGTTTGTCAATTTTTTCACTGGTTTTTCGACTATATATATAAAGTAATACAGTTGAAATCTAATAAAGATATTTGATAGAACGGAGTAGGCAAACCTGCTGGCTACTGGTACATAGAATATCGCACACATTTTGATCTAAAAGAATTAGTTTATATCATATATGGCCATTATCACCATTAATGTTATTTATTTAAATGTTAAAGACTTATGAGGGCCTATCAACAACCATCGGTCACAGTTCCAGTCATTTGTTAAATGTATAATAGTCATTGCTCATTCGCAAATCATTCATTATATTATAAATAAACTATTC

>gene9048

TACAAATCATTCATAAGTGTTCGGACCGAGTGTTTGGCCAGTCACAACCATGTCGGCATTATATTTCCTCGCGGCAATCCCAGTGTTAGCTTACGCTGTTTATTATTATTTTACGAGGACATTCAACTATTGGAAGAGTAGAAATGTTGCCGGACCAGAACCAACTGCATTTTTTGGAAACATCAAGGAATCGGTTGTTCGCACAAAAAATATTGGCATTGTAATGCATGACATCTACAAAGCATTTCCAAATGAAAAAGTGGTCGGTATGTTCAGGATGACTTCACCTTGTCTCCTTATCCGAGACCTGGACATAGTCAAACATGTCATGATCAAAGACTTTGAAGCCTTTAGTGATCGTGGAGTGGAATTCAGCAAAGAAGGATTGGGACAAAACTTATTCCACGCTGATGGAGAAACATGGACAGCTTTGAGGAACAGATTCACCCCCATTTTCACAACTGGTAAATTGAATAACATGTTTTACTTGTTAAACGAAGGAGGTGATTCATTTATAGAGTACTTTATTACTGAATGCCAAAAGAAACAGGAATTTGATATCCAGCCTCTCCTCCAGACATACACCATGTCCACGATCTCTGCCTGTGCATTTGGTATTAGTTATGACAGTCTTGATGATAAATTGGAGACTCTGAAACTTGTCGACAAATTATTTTCATCTCCAAGTTATGCATTTGAGTTGGATATGATGTATCCAGGACTCCTGAAAGCACTCAACCTTTCGTTATTCCCTTCATCTATACAAAAGTTCTTCGAAAATCTAGTGAAAACTGTTATTGCGCAAAGAAATGGTAAACCATCAGGTCGACATGATTTCATGGACCTTATTTTGGCACTCCGTGAAATTGGAGAAGTCTCAAATGCAAAACATGACTCTGCGAAGCCTGTGGAAATAACACCCGAGGTGATGGCAGCACAAGCGTTTGTATTTTATGCCGCTGGCTATGAAACCAGTGCTACTACGATGACGTTCATGCTTTACCAACTAGCAATGAATCCAGATATTCAAAATAAGTTAAGGGCAGAAATCGACGAAGTTATACAAGCAAATAATGGACAAGTAACATACGAGTCTATTAAGGAAATGAAGTACCTAAACAAAGTATTTGATGAAACTCTACGAATGTACTCAATTGTGGAACCTCTTCAGAGTAAAGTGGTAAGGGATTACCAAGTACCCGGTACTAACCTGACTTTGGAAAAGTACACCATTGTACTTATATCCCCAAGAGGCATACACTACGATGAGAAATATTATGATAACCCTGAACAGTTCAACCCTGACAGATTTGACCCGGAGGAGGTGGGCAAGCGCCATCCGTGTGCTTACTTGCCATTTGGACTTGGACAGAGAAACTGCATCGGAATGAGGTTTGGCAGACTCCAGTCACAATTATGCATAGTCAAGTTGCTGTCCAAGTTCCAAGTGGAGCCATCAAAGAATACTGCAAGGAAGATGGAATTTGAGCCCTGTCGTTTTATTATCGGACCCAAAGGAGGGATTCGTTTAAATATTGTTCCTAGAAAGTTGAAGGCTTGAAGACTTTCAAATTGTTGACAGTGTCTTAAATTAGCCGTTGTCATATAGCAGGCCAACAGGTAATTTTAGACCAGTTTTCCAAATTTTTCACTGAATTTGACTATATATATATATAGTCCAAAAATATATTATATAATATATATATGTATATAATAATACAGTTGAAATTTAATAAAGATTTTTGATAGAACGGAGTAGGCAAACCTGCTGGCCACTGGTACGTAGAAGATCGCGCACGTTTTGATCTAAAACAATTAGTCATATCATATATGGCCATTATCACCAGTAATGTCATTTATTTAAATGTTAAAGACTTATAAGGGGTTAGCCTATCGACAACCATCGGTCACTCATTTATAAAATGTATAATAGTCATTGCTCATTCGCATATCATGCATATCATTCATTATGTTTTAAGTAAACTATTCTTAATTTAATAATGTAGAATATAAGTTAGTTTTTTTGTTACATAAATACATTTGTTATACAAAA

>gene9049

TTCACGAAATCGCTAACTCATAACACATTTTTCACTAATTGTGTTAGGAGCTTCAAAAATGTCGTTCCTTTATATTGCTGCCGGATTAAGCGCATTAGTTTACGCGTTGTACTATTACTTCACAAGAACATTCAACTACTGGAAGAGCAGAAATGTTCCCGGACCGGAACCAATCCCGTTCTTCGGGAACATCAAGGAATCAGTTCTACGGCAGAAGAATATAGGCATCATAATACATGATATATATAAGGCCTATCCAAACGAGAAAGTGGTTGGACTTTTTAGAATGACTTCTCCTTGCCTCCTCATCCGAGATTTGGACATTGTTAAACATATTATGATCAAAGACTTCGAGGTGTTCACTGACCGTGGAGTGGACTTCAGCAAAGAAGGACTGGGACAAAATTTGTTCCACGCTGATGGAGAAACATGGACAGCTTTGAGAAATAGGTTCAGTCCCATTTTCACCACAGGTAAATTGAAAAACATGTTTTACTTAATGAATGAAGGAGCTGACTCATTTGTGGACTACGTTACTACAGAATGTCAAACAAGACAAGAGTTTGAAGTGCACCTTCTCCTGCAATCGTATACTGTATCTACTATTTCCGCCTGTGCCTTTGGAGTTAGTTATGACAGCCTCCAAGACAAGATGGAAGCCTTGAAAATTGTAGATCAAGTTATTTCATCACCAAGTTACATCGTCGAGTTAGACTTTATGTACCCAGGACTCTTACGATCACTTAACCTTTCTCTTTTCCCGAAAGTGGTGCTGCATTTCTTCGCAAATCTTGTTGACAATATTATTTCTCAGAGGAATGGTAAACCATCAGGACGAAATGATTTCATGGACCTCATACTAGAACTTCGTCAAAAGGGAGAAGTAGAAAGTACCAAATACGGACACAGTGGCAAGACTCTAGAAATAACGTCAGACGTGATTGCGGCGCAAGCTTTTGTATTTTATGTGGCTGGGTATGAGACTAGTGCAACCACTATGGGCTACATGTTGTACCAACTAGCGTTGAATCCAGACATCCAAAAGAGGTTGACCGAGGAGATTGATGAATCACTGAAAGCAAACAATGGACAAATAACATACGACACTATTAAAGAAATGAAGTACTTAAACAAAGTATTTGATGAAACTCTACGAATGTATTCGATTGTGGAACCTCTACAGAGGAAAGCTGTAAGAGAATACAAAGTTCCCGGTACTGACTTGACAATAGAAAAGGACACGATAGTACTTGTATCTCCGAGAGGAATTCACTATGATGAAAAATATTACGACAACCCTGAAGTATTCAACCCTGACAGATTCGACCCGGAGGTGGCGGGCAACCGCCATCCGTGCGCCTACTTACCATTTGGAATTGGACAACGAAACTGTATCGGCATGCGGTTTGGCAGACTTCAATCTCAACTATGTATAACGAAGTTGTTGTCCAAGTTCCAAGTGGAGCCGTCTAAGAATACCGCTAGAAACTTGGAGGTGGAACCTCACCGCGGTATCATCGGGCCAAAGGGAGGAATCAAATTAAATGTTGTTCCGAGAAAGATAAAATCTTAAAAACCTATTGGATTCCAACCGGAAAATATGATAAAACCCTCCATAAATAAAAACCGAAATATATCCAGAATCGTGTCTGTATAAAAGAAAATATGTATAGGACTAATATAATGGTTTGAAGTCAGTCTTACAATTTATTCTGAAATCGTCAGTGGTTGAAGTGAAAGGTATAGGTAAAGTAAAAGTTTTACTTGATTATTATACTTATACCAAAATTCTGAAAAAAACAATGCATCTCTATTTAGATGAGCCACGATTTTGTAAAGTGATTATACTGGGACGAAACACCATAATAAACCCAGAAAACGCAGCCTTCTTTTGGCGTCTAAACATTCCTTTGGAAATTAGATTTTTTAAGTATGTACCTAGTGTATGTGTTTGGAAATACCTATGTACTATGTACAGAAAAACGAAGAATTACCACAAGTCATTATTACGTTAGGTGTGGTTATCAAACAAGGTATATATTGGATGTAGTAATCCGACCAGATTATGAGACTGACAACGTGAAAAGATAAGTTGATAAGTTTGCAACAATGTATGTATAGAATGGAACCGAAACATATTATCTAATCGTTGATTATTTAATGTTCTGATTATAACGTGTGTGTATGATACTATTAGATTTATAGTACTTTACTGGTTTAGGGAAAATTGTACCTTATGCTATAAACTTTATGTTTCAAATTCCAAAGAAACCATTGCAGCTATTGTAAATATTTAGTTTTTAACTTTTGTGATGGTATATGCCTAAATTCTATATTATAAATTATATAATAAAACTTATACAAAAATTA

>gene9050

CTATAATAACGGTGCTCGACGAGTTAATTGAATTGTAAAATATTTCAGCTGCAGAGCCTATTCTTTTCTTATAGGAAATACGAATAAATATATTATCTCTTATTACTAGTGTGATATTACAGTTGTAAACATATATTTTTGAATCACACTAAGCAGTTACGCAATCGGCGTGACCTTGAGACCCTACTGAGCCACGAGACCGTTTCAATTAAGTACCGTACTCGGCAATGCGTGTACGACGCAATTGTAAAATTGGGTGAGTCAATTGTTAATTCACGTCCTACTCAGTACATACAGTAACACCGAACTAAATAAATAAATTGAGTATTTTTAACGTCTTTAAGTATTGTTCGTGATGTTACTAAACATTACACATCTTTTATTCTTCTACTAGTTTTTGCCAGCGGCCTCGCCCGCGTTCACGTTCGAAAAGTCTATGTTTTATTCCAGATTATAATCTACATCTATTCCAAATTTCACATCGATACTTACAGCCGTTTTGACGTGAAGGAGTTACAAATAAATATACAAACTCATTCATAATATTAGTAGGATTAGGTTGGCAAAGATTAACTGAAAGTGCCATTTTAGCGAGATGTATTATTACTTATATTGTATAGAATTAGCTTAAACTGTTAAACATCGCGAGGACACCGGTGACCGCAACCTATTGTTCTTTAATTGTGTCTATAATGACGGTACTCAACGAGTGAAACTAATATTCCAAGGCTAATCACTACTGTTAATCTATTTACAAGAACAATGTTTATCAAAGCGTACAAAAAAGACAAATAATGTAGGTATATTTATCAAAAACCCGCGTGATAATATTAGTACATAATTCGTACAACACTTTATTAACATAAACACCACATGTTTTTATCTATCGTAGAATTTTTTGTTAAATTTAATAACAATATAGAGGCAAGAATTTGATCTCAACATCGTTTTTTCTACGTCTGTTTTAAACAGGTATGGTAGGCCTGTCTTAATTTAAAAATTCCTTTTTGATGTTACATCATGACATCGCCACGCCTATTTATCAAAGAAAGAGATGGAAATACTCTACTATTTACCATAATACCCATTCATCAAGTACCATTTTCCTCGATGTAAGGTTAAATTATACCTTTTGTCTTTCCATGGGTTATGTAATGTGCCATTTACGACACAAACAAACATACATGTGACTATTGACATTTTCGAAGTTAATCAGATATTGATAAAAAATATTATAAGCCTTGATAAACACGTTTATTAAAAACAAAACGTAGGTATTATATTTAGGTAACAATAACAACAACAAACGTGTCTGTCCAACAATATAACAACAGATCTAATGGTTCATTCATTCATTCATTCAATTCACATTATTCGGTGACTGAGTTGTGTACTCTCTGCAATGTGCGGAATAAACTTATTACTAGTGATAATAGTGTTATGTGGTTGTTTATACTATTACTTTACAAGGACGTTTAACTATTGGAAGAAGAGAAATGTTCGTGGACCAAAACCAACAGTCTTTTTCGGAAACATCAAAGATGCAGCTCTGCGAAACAAAAATCACGGCATTGTCATTCAAGAGATATACGACATGTTTCCGAATGAAAAAGTCGTTGGTATTTATAGAATGACGTCACCTACGCTACTGGTACGTGATTTAGATATAATCAAACATATCATGATCAAGGACTTCGATGCTTTCACTGATCGTGGGCTGGACTTCAGTAACCGAGGTTTAGGACAAAACTTATTCTTTGCTGATGGAGACACATGGACAGCTTTAAGGAGTCGATTTACCCCAATTTTTACAACGGGTAAATTAAAAAATATGTTTTATTTGATAAATGAAGGCGCCAATAATTTTGTGGATTATGTTAGCACTGAATGCCAAGTAAAAGAAGATTTTGAAGTGGTCTCCCTTCTTCAAACATACACGTTATCTACGATCTCATCCTGTGCCTTTGGAATTAGTTATGATAGTCTAAATGATAAAGTAGAGACACTAAAAATTGTTGACAAAATACTTGCGAGACCTAGTCATGCACTTGAATTAGATATGCTGTATCCAGGACTTTTAAAGTCGCTTAACTTATCCATCTTTCCAAGAGAAGTACAACAATTTTTCAAAAATCTTGTGGATCTCATAATCACTCAGAGGAATGGGAAACCTTCGAACAGGAACGACTTCATGGACTTAATACTCGAACTTCGTGAAATGGGAGAAATAAATAGCACGAAACATGGAAACACTGCGAAGCCAATTGAAATAACTCCAGAAGTAATAGCGTCACAAGCTTTTACATTTTACGTAGCTGGCTATGAAACCAGTGCTACTACAATGTCGTACATGCTTTACCAACTAGCAATGAATCCAGACATTCAAAATAAGTTAAGGGCAGAAGTTGACAAAATAACCCAAGCTAATAATGAAGAAATAACATACGAAACCATTAAGGAAATGAAATATCTGAGCAAAGTCTTTGATGAAACTCTGCGAATGTATTCGATTGTGGAACCTCTGCAGAGAAAAGCTACAAGAGACTACCAGGTACCTGGAACTGACCTGGTAATAGAGAAAAACACGATGGTACTCGTATCCCCGAGAGGTATACACTATGATGAGAAATATTACGATGATCCTGAAGTATTCAACCCTGACAGATTCGCCCCGGAGGTGGCGGGCAACCGCCATCCGTGCGCTTACTTACCGTTTGGAATTGGACAACGAAATTGTATCGGTATTCGGTTTGGAAAACTGCAGTCTCAACTGTGCATAGCGAAGATTTTATCTAAGTTTGAAGTGGAACCTTCGAAGAATACACCAAAGAAGTTAGAAGTGGAACCAGACAGGGTTCTCATTGGACCAAAAGGGGGAATACGTCTGAAATTAGTGCCAAGAAAAATTAAGACGTAGATTAAAGAAGAAATAAGGTCGATTTTTAAGCCTGCTTCATACGGCGGGTTACAAGAGCAACTGTCGAGCATGGGGTCTCGGGCTAAATTTTCCAAGCTAAGCAAATTATTATTGGGCCTTTAAAAATCCAGCTCAGCCTTTGCTGAGCATATTGAGCTGAGCTTTCAGCCGTCTTGTTGTTTACCTCTAATATCATAATAGCCTGGACCCTGAAATCGTGATATGTCTTCTGCCTATTCTTAAATATAACAAGAGACTCGTCCGTAATTGGCAAAAAGTGAAGGTTAAAATATAATACGAATTCGAAGAATTACTATACTGTAATATTGTGTACCTATATTTCGTTATATACATATAGTTTACGGCACAATATTATAAATTTTAATTTGTACTTTAGTTTGCAGAATAAATAAGCTATTTTGTAGTGTTGTTGTTTTATATCAAAATCAAAATAAACCGATGTAATTATATCTAGACGCTGTTAATAAATAATAACAAATAGTTTTGTTAAAAATACAAAGTGCATTTAATAGTAAATTCTTAACACAAAATGTTTTAAAATAATAAAACAAACTGAGTAATCGAAACCTTTTGAGAGGTTGTATTCTTAGAAAAAAGTATTATTATCTATTTATATACTTTGACAAAACCTGTGAATAATGTCGCATTGTTGTTCATTGATATTACTTAGTTTTTAGTATATGTATTGTATTTCTGCATACATTAATCTTAAATTTTGTAAAACTAAGTTACATTCTCAGCTAATACAATAAATAACAATAGTATAAGTAACTTATGAAGTTTCTACAACAATAAAAGAGTTTTGTAATACATAGTTGATTATTTTTCTTTCAAGTCTAAAGAACACAATCTATTGAAGTTCTGAGTGCATTCCTATAGTATAGATATATCAAACACTACTAAAGAGCAGACAATGAAAGATACAATTCGTCTATTTCGCAACAAAATGATTGCAAACAAAGTGATTATAAGTGTCGCGTAGCCATCCACATAGTAGCAAAATGTTACATAAAAAGATAATTGCGAATGTCACAGAGATTCAAAAACTCACTAAAATCAACAAGTCTTCGCCATTTGCTTCAAACAGCGAATAGGCATTACAATATCACGTAATACAAAGCTATTATCTCTGAACTGTAGTTAACTTAAAACAAATTTGGTGATAACGCACACTAACACTGATAA

>gene9051

AACAGTTCTATGCATATCACAGAACCCACAGATTGTGCAAAGAACTCCTCAAAATGTTGCTGTTATATCTCCCGATAGTGCTGACATTTTTCATATACATATTGTATTTGTACTTCACAAGGACTTTTAAGTACTGGAAAAGCAGAAAAGTTCGCGGGCCAGAACCAACCGTGTTCTTTGGTAACATCAAGGAATCAGCTCTTCGCCAAAAAAATATTGGCGTGATAATGCAAGAAATCTACAATAACTATCCCAACGAAAAAGTTGTCGGTGTTTACAGAATGACTTCTCCTTGTCTTCTTATACGAGACTTGGATATCGTTAAACATATCATGATCAAGGATTTCGAAGTGTTCGGTGATCGCGGCGTTGAATTCAGCAAGAAAGGACTGGGACAAAACTTGTTCCATGCTGATGGAGATACATGGACAGCTTTGAGGAATCGGTTTAGTCCAATTTTTACAACGGGTAAATTAAAAAATATGTTTTATTTAATGAACGAAGGCGCTAATAATTTTGTGGAGCATATCAGCACGGAATGCCAAAAGAAGCAAGAGTTTGAAATACATTCTCTTCTCCAAACGTACACAGTGTCTACTATATCTGCTTGTGCTTTCGGAGTTGGCTACGACAGTCTAGATGACAAGTTGGAGACGCTTAAACTCGTGGATCAGATAATTTCAGCACCAACATACGCTAATGAATTAGACATGATGTACCCAGGACTTCTCACATCCCTCAATCTTTCTATTATTCCGGTACCTATACAGAAATTTTTCAAAAATCTTGTCGATAACATCATATCTCAAAGGAATGGTAAGCCATCGGGAAGAAATGATTTTATGGACCTAATACTAGAACTTCGTGAAATGAGAGAAGTCAATTATACAAAATATGGAAATTTTGTGAAGCCTCTAGAAATTACTCCTGAGGTGATGGCAGCGCAAGCTTTTGTATTTTATGTGGCTGGGTATGAGACAAGTGCAACCACTATGGCCTACCTGATGTACCAACTAGCGCTGAATCAAGATATCCAAAACAGGTTGATAGCTGAAATTGACGAAGTAATTCAAAGGAATACTGGAGAAGTGACATACGACATGATTAAGGAAATGAAATATTTGAACAAAGTATTTGATGAGACTCTACGAATGTACTCAATTGTAGAACCACTGCAAAGAAAAGCCACAAGAGACTACCAAGTGCCTGGGACTGACCTGGTAATAGAAAAGGGTACAATAGTTTTGATATCCCCGAGAGGAATTCACTATGATGAGAAATATTACGACAATCCCGACGTATTCAACCCTGACAGATTTGACCCGGAAGAGGTAGGCAAGCGCCATCCGTGTGCTTACTTACCTTTTGGACTCGGACAAAGAAACTGCGTTGGCATGCGATTTGGCAGACTTCAGTCTCAGTTATGCATTGTAAAGGTCTTGTCCAAGTTCCGCGTGGAACCGTCGAAGAATACAGACAGGAATTTAAAGGTTACTCCACACCGAGCTATCATTGGACCAAAAGGAGGGATCCATCTTAATATTATTCCTAGAAAAATCAAAGCTTAGGGTAAAGATTGTAGTCTAAAAAATCTACTGAAATATACGTAGATGTATGTACAGTACAATTAATTATGTTAGACTTATAACTTAAAACAAGTTCGTTAAACTGTATAGTTTATTTAAATGGTTCATTTTAACCTCGTAATATCCTTGAGATTCTTTTTAAATGATACTAAAAGTAAGTTTACCAATTTTAAGGTGGATGCCTAGATAGACCTCGCCATACATTTTTCAGGTGTGGAAATCTCCGTAAATAAACAGTTTCTGTAGAATGAGAGCAATTCTAATGAGACTTTATAATGTTATGTTCGCGTATGGCCCAAATTCTATTTTATTATATTTGCACAAAAGACTGTTCATGTTCGAAATGAATTTTATTTGTAAATAAATATTTTTAGCATAGTATTAGGAAAAATCATGTTGTAACATATTGATAGCCGATGCTCCGACTGAAAATAAATAAATGAGTTTACA

>gene9123

CATTTAAAATTTGCGACGCCACACATATCTGATGACATATATACATTTCTGGAAAGTCCAACTGTGATAGCATCAATTTTAAATAGACTGATAGTGATCGATAATATAGATTCTTATCAGCTGTGTTTGTTGTTTAGACTTCTGTATTTTGAAAATAAACTATCAATAGAGAAATTTTGAAAACAACAGTAAATGAGTATTTAAGTTTAAAGCTGAAAAACAATTTAGTCCATAATCATAATCAAGATCTGAAAAGAAAATACTGACCACGAAATTTTTTACATAAAGGTGCATAATTTCGAATAATGTAGTCTTAAACCAAGCATTAATTTTTGGTTGCGAACTATTTTTGACCTGAGAATAATAAACTGTAGACAATACGTCTTTTTTCCAAAATACTTCGATGCATATTGCACACGTATCTTTGCACTCATCCGACTACGTGTACAATGGCTAATCCAATCATCAAAATATCTACTACCACTAATTGTTCCCATTATCAGTGCAACGTCGTATCGTAGGCTCTTCGGTCATATATAAACAGAATTACGTCGAACACGCCTATCATTAACACTAAATTAGCCACTACGGTGACATCTACAAAAATGTTTATTTATCTGCCAACTTTAATAACTTTCGTTTGCGCTCTGTATCTTTATTTCACTCGAACTTTTGACTTTTGGAAAAAGAGAAATGTGAAAGGACCATGCCCAATACCATTTTTTGGTAACTACATTGATGTATTCTTCAGAAGGAAACATATTGGCGTTCTATACCATGATATTTACAAACAATATCCAGATGAGAAAGTAGTGGGTTTATACAGAATGATGTCACCAACTTTGTTGATTCGTGATCTTGATATAGTGAAACAAGTGCTGCAAAAGGATTTTGAATCTTTTCCTGATCGTGGAGTGTACTACAGCAAGCAAAAGTTGGGAAACAATCTCTTCCACGCTGACGTCGAAGTGATGAAAGGACTAAGAAAACATATGACTGCTGCATTCACACCAAACAAGTTCAAGGCTAATTTCAATATGCTTGCTAATCGAGCGGAGCAGTTTCTTGAATACATGGGAAGAGTTAGCGACGAAAATGGAGAAGTAAATATACTGCCGGTTTTTAGAAAATATGGTGCTGACTCTATCATGATGGCTGCTTTCGGTATCGATTTGAAGCCTTATGATGAAAACAACTTAATATGTGATGTGTTGGATGAAGGAATTCAGTATCCTAGATATCTTCTTGAGCTAGAGCTACTTTTCCCTGGCAGTCTTACAAGATTTGATTTATCAATATTTCCTGATAGAATATCTCGGTATTTTAAACAAATAATTGAAGCTGGTGCGACATTAGGAGTCACGGAGAAAACCGAAAGAAATAGAGCTATTGATATTATGATGGAATTAAAAAGACAAGGAAGTATTAATGCCTCTAGGAAAGAAAATGGCGAAAAGGAACATTGCTTGGAAATCACAGATGAGATGTTAGCTGGTCAAGCATTTATCTTCTACTTTGCTGGTTATGGTAATAATTCATTACTATTATCTTATGCATTATATTACTTGGCCAAAAACCCTGAAAAACAAGATATACTAATACAAGAAATTGATGAAGTTATGATGAAGCACGATGGTAAATTTTCTTACGAATCGTTAAAAGAAATGAAATACCTAGAGATGGTTTTTGAAGAGACTTTACGCTTGCGTCCTATGACAAACGCTGTTGTCAGGAATGCTGCAAGAGATGTGCAGTTAGAAGGCACAGATATCATCATTCCAAAAAATACTATCCTAGCAATATCTCCATACTCATTCCATCACGATGAGAAATATTTCCCTGAACCAGAGAAGTTTAAACCTGAGAGGTTTTCTACTGAAAATATGAGGGAACAACATCCCTGTGCTATGTTATCCTTTGGACTTGGGCCAAGGAGTTGTCTTGGTTCAAAGTTCGCTCTACTCCAGTTTAGTATTTGTATGGTGAAGTTGTTATTGAAATACAGAGTAGAATGTACCAAGGACACTCCAGAATCACTCACGTACACTCCCACGCGACTCCTACTGACTCCAAATGAAAGAATATATCTCCGATTAGTGAATAGAGATAAGTAATAGTAATGTACAATAATCCAATTAGGTCTTTTAAATAAGTAAATCCTGTAAATAAACTTAGATATAATTATTAAATTATTA

>gene9140

ATGATTACAATAATAACATGTGTTTCAACAGTATTGTGTTTAATTATTTGGTTTCTATTAAAATGGAGGAAAGTGAAAAAATTTTGGGCTGACAGGGGCGTTCCTCATTATTCCCCGCATCCCATTTTCGGTAGTCTCGCGTTTTTGCAGCGAAAAAATCCTGCAGTATGGATGAAGCAAGTTTACAACGAGTTCAAAACACCATACGTCGGTATGTGGGCATTCTGGAGACCAGGTCTGGTGATCAACTGTCCAGACTTGGCCAGGAAGGTGCTGGTCAAAGACCATGAAGTGTTCAAGAACAGATTTCTGAGTTCTGGCAAGTCAGACCCCATTGGGGGTCTTAATTTGTTCACTGTAAATGATCCAACCTGGTCCTTTCTAAGACGACGTCTAACAATACTCTTTACGGCAGCTCGACTGAGAAGCCTAAACAGCTTACTATCAGCTAAATCCAATGATATGGTAAAGCGGATCAGAGATGACATGTCCAACGAAGATCCTCTGAACTTGAGGGTCCTATGCTCAGACTTCACAACAGACGTAATAGGAGAAGCTGCGTTTGGTCTCACCAGTGAGTCAGTCAGGACTGGTCATAGCCTGATGAGGAGAATCACCAAGGAGTTTGTCAAGTTCAACCTTCATAGAGGATTGTGTTGGTCCAGTATTTTCTTTTTTCCTGAAATAGTTGATGTTTTTAGATTTTCCCTATTCCCGAAAGATTCATTAGAGGTTTTACGTCACATATTCAGAACTATAATAAACCAGAGAGGAGGTTATGAGAAGGAGGTCAAACAATGCAGGGACCTCCTTGATGCTCTACTGAAGATCAGGCAAGAAGCTGCCGAGGATAACGAAGAGATTAGTGAGGATTTGTTACTTGCTCAGGCCGCCATCTTCCTTCTGGGAGGCTTCGACACTTCAGGAGTAACATTGACGTGGACTTTGTATGAACTGGCTTGGAACCCACTGTGTCAGGAGCGACTGTATCAAGAGCTTTTGGACGCAAAACAAAAGAACGGTGACAAGGATTTGGACGCTAGTTCTCTTGCTGAACTGACATATATTAACTGTGTTATAAAAGAGGCATTACGCAAATTCCCTTCAATGGGTTGGTTGGACCGCATTGCATCACAAGACTATAAAATCGACGAGAATCTAACTATACCCAAGGGAACAGTGGTGTATGTGAATGCGGTGGGAATGCAGCAAGATCCTCAGTACTTCCCGAACCCTCAGATATATAATGCTGACAGGTTCCTTCCTGAAAATGAGAGAAATATTACTCCTTACACGTTTTTACCTTTTGGAGATGGACCAAGAGGTTGCATTGGTAAGCGCTTCGGCTATCAGACAGTTCGATGTGGATTGGCAGCCATTATACTCAATTATGAAATACGGGCCCTACCCAATATGCCGAAGCCAAATGAGTGTCATATTGAAAAGAATGGACTCTTCCTAGGTCCAGACAAGAAACTTTCTATAGAATTTAGACTTAGGAATTAATGTAACATGTTTTATGGTAAACAGATGTGTTCATAATGAATGTATTAAGTAGTTTTCGTCTTTTGCAAGACTTGTGAGTCCTGTGTTATTAAG

>gene9153

CGCAGGCGCGAGCCGCGAGCGCTATGTCTCGCGAATTTAACTATTATTAAAATAATTTTCACTATTAAATAAAAAAATGTACAAGTCCGCCCCTGCGTGAGTGAAAGAGGATTGCAGTTTTGAATTATATTGTATAATATTTTCTTAGTAAAATAATGTTGCATACTTAATACCAAAATGTTTGGGTTCAAACGATCGTCTGTGCAACTTGTGGTGATACTGGCTTGGCTTTTGCGATATTACACCTTCGGGTCGATAGTAATATGGTGTATAGTCGCGTGTGGTTTGAGTTTGGCTATTGTTTTGGCTGTGACGACGTATGACCATGACTACTGGACGTCCCGTGGGGTGTTCTCCCCGCCTGCGTGGCCGGTCGTGGGCCACATCCCGTCCGTGGTGATGTTCAAGGAGCAAGGAGGCATGTGCTTCAAGAGGATATACGATACCTATCAGGATGAGAGATTTTTAGGCTGCCACCAGTTCTACCAGCGCACTCTAGTGGTCCGCGACCCTGAGCTGATCCGAAGAGTGTGCGTCAATGACTTCCAGCACTTCACAGACCGAGGGTTCTTCTTCAACAAGGACGTGGATCCTCTCGCTGGGTCTGTGCTGTTCTTGAGGGGGAATGAGTGGAAGAGGCTCAGAGCTAAGATATCGCCTATATTCTCACCCAACAAACTGCGTGGCATGTTCCCGCTCATAGAGAACACAGCGGTAGAATTCGTGACAAAAGTTCAAGATTTGCTGACACAGTCTAAAAATGAACCGAACAAAAATGGCCCAGTGAAGACAGAAGGTCAATCGAATGGTGTGGAACAAATCTCCGCTGTGGTAAACTCTGAGAAGCTGGTCGGTGGCTATACAGCTGATGCCATAGTGCCTTGCGCTTTTGGTTTAAAAAGTAACGTGATGTATAATGAAGATGATCCCTTCGCTGTAGCATTGCATGCGTTCTACGAGATGTCGCTGTTTAATATATTTGAGAAGACGATGCGTCAGTTTTGGCCCGCTTTTGTATTGTTCTTTCGAATGAGAATCATACCAAAGAAGACACATGACTTCTTCTACAACATCGTCACGACGGTGCTGAGGGCGCGGGAGAATGGCGATCAAGAAAAGCGAGGGGATTTCATTGACATGATGATGGCGTTAAGGAATGATGAGTCCAACAATAATTGTAAGAAGGACCAAGAAGATGTCGAAATCACTGACATGGTGATATCAGCAAATGCTTTTATCATCTTCCTTGGAGGGTTTGAGACGACATCTTCAACTCTAGCCTTCCTATTCCTGGAGTTAGCAGCCAACCAGCAGGTGCAGGAGAAGATGAGGGAAGAGATCAGGCAGGTCGTGGACAAACACGAAGGGAAGATCACGTATGAACTGCTGCAGGAGCTGGTTTATATGGAGATGGTGATTCAAGAAACTCTTCGCCTGTACCCTCCATTCCCGAGCATCCAGCGTATGTGTACCAAGGAGTACACAATCCCCGACACCAACATCGTGGTGGAGAGAGGGACTATAGTGCTGTTCCCAACGCTGGGCATACAGAGGGATGAACAGTACTTCGAGAACGCGTCTGCGTTCATTCCTGAGCGCTGGTCTGAAGGTAGCCCGCAGCCGCCGCCAGGAGTCTACATGCCCTTTGGAGACGGGCCGCGGTATTGTATAGGTAAAAGATTCGCCCTTATCCAAATGAAATGCTGTCTGCTACGAGTACTGCAGCATGTGAGGATTACTCCAGCTCCTCGTCCTGATGCTGCAGGGAAACTATCAGTCCAACCTCGACTGGAACCCTTCACTGCTGACCCTCGGTCCCCACTAACGCTTCACCCCGCTGACAGCCTGGTAACTCTTAGCTTGTTATGACGAAATTCTGTCTTCGTTTGTAGAGACCAGCACTTCAACGTATTCTAAAGATTTTAAACCTTCATATTTAGTAAAAGAGTTGAAAATTGATGAAGTAAACTGACAGTCCAGCTTCGAGTGGATCCTTTCAATCCCTCGGTCCCCACTAAGTCTTCATCTCGTTGACTGCTTAGTTGCTCTTACCCTGTTATATCTCCCGTAACAGGCTAAGAGCAACTTTACAACTTACGCAATAAAGTTTAAAGTCGATTCAACAAACAGTTTGGACTTAGTCTGCCGGTATATGGATATCCAAAAGATTTTCAACTCTACTATTCAGATAGTAAAGTATAAAATTGATTAAAGAAAGATCTTTAGAACACTTTCTACTGGTATGTGCACGATTTGAATATCTGTGACGATTTAAATATTGTTAATAAGTCAACTATTTTTTCGTAATTACTATGTGAATGTTTCACATTCCAACAAATTAAAATAATTGCATAAGTTTCATGTAAAGAAGTTTTTAAAGCATCGATGGACCTATTTAAAGTTTTTTTTTAAAAATTCATTATTATGACTTATGACATCTCGCCGGCTCTGCCTCGAAGATTGTAAAAGAATATTACTTTTTTTATTGTTTGTTTCAATCATTTAATTTCGTTTTGTAAATAGTGAAATATAGTCTAGCTAAGAGCCAAATATTCATACTAATCATTGAAAAAATAAAATGTGAAAATGTCTTTGTGTTCATAACTCTTGAACGACTGTACCAATTTTGATAAATCTATTTTTATACAGAATATAGCTCTTGAAGTATCTTATACATTTACGAAGTCATGTCTTATTATTAATAATTTGTTACTGAAATGTTGAAAATGTAAATAAAAATTGATTTTAGCCTGTATGTATTAGTATACGGCTTTGTTAAAATAAGGATTAGCTCATAAGTGATAGTTTTATTATTATTATCTTGTCGTTTCTGTAA

>gene9174

TCGTGTGGAGTTCACGCACTCCTGCGCACGTAGAGCGCGCTACTATTCGCGGAGTGGTTCTAGTGTTGTGGTTTATTTAATTACTTGTTTGAATTCCAACTGCCGGTTGGTTGAAGACCAGCAACATGATATTTGAGTTGCTGCTGGCGGCGTTGGTGGCGGCGGTGGTATGGCTGATATTTTTCCGAGATGATGATAACCCCGTGGACAAACTCCCCGGACCTCCGAGGAAACCATGGATCGGGAACGCCTGGGAGCTGCTCCAAATGCCCTCAGATAAGTTATTGGACATATTGGTCGACTACACGAACAAATATGGCGACCGCTATGTGTTCAAGGTGTTCAGTCTCCGAGTGCTGCATATATCTGGACCTCCTGATGTTGAGACGGTGCTCTCGCATTCGAAGAATATCAAGAAGAGTGCTCCTTATGACTTCCTAAAGGGATGGCTGGGCACCGGACTCTTGCTTAGTACTGGTCTCCACTGGCACAAGCGCCGTAAGATTCTGACTCCAACCTTCCACTTCAACATCCTGAAGAACTTCGCCCACGTAATCGAGGAGAAGACACAGGATCTGGTGAAGATGCTCAAGGACAAGAACGGAGCTGATGTGAGCTTGATGCCCACAATCAGTGATTTCACTCTCTTCACTATTTGTGAAACGGCAATGGGGACAAAATTGGACGACGATAAGACATCAGCCACAGTGGACTACAAGAACGCCATCCTTAACATCGGCATGCAAATGTTAGCGAGGATCACCCGGTTTTGGCTGCATAACGACTTCGTCTTCAACAACTCTTCACGAGGAAAAGAATTTAACAAAACCTTGGAAGTAGCTCGAGCCTTTGCTGATAACGTCATCATGGATAGGAAGGCCCAAAGAACTCAGAATAAGAGAGAAGAAACTGCTATTGCTGATGCGTCTGATGGCATTGGAACTAAGAAGAGATTGGCTATGTTGGATCTCTTGCTGGAGGCTGAAGAGAAAGGCGAGATTGATATGGATGGTATCAGGGATGAGGTTAACACTTTCATGTTTGAGGGACATGATACAACAGCCTTAGCTCTTACTTTTGGCTTGATGCTGTTGGCTGATCATGAAGATGTACAGGAGCGCATCTACGAGGAATGCCAAACGATCCTGGGTGACTCAGAACACGTGACGATGTCCAATCTGTCCGACATGAAATATTTGGAGGCAGTCATCAAGGAAATACTGCGATTGTACCCCAGCGTGCCTTTCATTGGACGAGAGATCACTGAAGATTTTAAGCTGGGTGACATAACAGTGAAGAAGGGTACGACAGTGGACGTCCACATCTACGAGCTGCACCGTCGCGCTGACATGTTCCCGGAGCCGGAGAAGTTCATGCCGGAGCGGTTCCTCGGCACGGAGATGAAACATCCTTACGCTTACGTGCCGTTCAGCGCTGGACCTAGAAATTGCATTGGACAAAGATTCGCGATGCAAGAAATGAAAACCACACTTAGCGAGCTGGTCCGTCACTTCAAAATTGTACCTAAAGTCAAGGGAGCAAGGCCTAGAATCATGGCGGACTTGGTTCTCAGACCAGTGGATCCCATCTATGTCAAGTTTATTCCTCGCTAAATTATGTGTAATTTGTTCACATTAAGACAGTGGTGTGTCATTGTTGCTGAGATTTGATTTGACCTATATTCCAACTCTTGCACATCGTGCTGTTAAAGTGTTTACTCAATTTTCAGCTTTATTATTTTGTGTTACAGTTGTAAATCCAATATTTTTTGGCGGTTTATGTTTAACATGCTAGACAGTCTGTACGTACCTTTCAATATCACTGATCACTAATACTGTTACAGCTCGACTATGCATTACCATGAACCAACTTGACAATCTAATCATTAGATAATAATAATAGTGCATACAGGGATAGTCGCCGGTTTGTGTCTCCATTACATTTAGATTAAAGTTGTCT

>gene9175

TTGTGTTCGGACAAAAATATTACTAAACTCTAACAAATAGACAGTTAATAATAACTTACATATAAATATATGTATATGTTAGTTTGACAGGTCTTCAGTGTGTGTGTATGGCTGTGTGCCGGTGTTTTATGTAGTGTTTATTATTGTTTGTGTTATCAGTCAGTGATAGCGAGCGTGTTGAGAAACGATTAATAAAAGATATATCATCCCTACTGAAGCAAGAAATAAGTAATAACAATGATCGCCACATTGCTATTGGTGGGTTTCATCGGGCTGGTGATCTATAGACTCCTGGCTGAGGAGGAGAATCCTTTGGACAAATTACCTGGACCACCACGGAAGCCGATCGTAGGATGTGTCTTCGAATTCCTGAATCTTACTCCACGTGTAATGTTCAAGAAAATGCGACTGTACAGTAAGGTGTATGGAGGCCGCTACATTGTTAAAATATTACGAAGAAGGATTCTACATCTGCACAATGTTAATGATGTTGAGATCGTGTTGTCTCATCCGAGGAACATAAAGAAGAGTAAGCCATACAGCTTCTTGGAAGGCTGGCTTGGCACTGGACTGTTGCTCAGTTCTGGTGCAAAATGGCACAGACGTCGCAAGATCCTGACTCCAACTTTCCACTTCAACATTCTAAAGAACTTCACCAACGTGATGGAGGAGAGGAGTCGAGGACTGGTAGACAAGCTGAAGGAGTACGACGGCAAGGAAGTCAACCTCATGCCGGTCATTAGTGATTGTACCCTGTATACTATTTGTGAAACCGCAATGGGTACTCAATTAGACTCTGATTCTTCTACAAAGACCCAAGAATATAAGACAGCTATCCTGCAAATTGGGGGGCTACTTATGGGTCGTCTCACAAGAGTGTGGCTGCACAATGAGTATATCTTCCGAAAATTCCCAATGGGTAAACAGTTTGAGAAATGTCTAGAGAAAGTTCATCCTTTTGCTGATAACGTGATAATGGAGAGGAAGAAGAATTGGAAACCTGGACAGAGTAATGCAGGAGAAGATGATGTAGGTGGTAAGAAGAGGTTGGCCATGTTGGACGTATTGTTAGAAGCTGAGAGGAAAGGAGAAATTGATTTAGAGGGAATAAGAGAAGAAGTTAATACGTTTATGTTTGAGGGACACGACACTACAGCAATGGCTCTCGTTTTTGGACTTATGTTGCTAGCAGACCATCCAGAAGTACAGGAACGGATTTACGAAGAATGTCAGACGATCCTGGGTGATTCAGACACATCTCCAACGATGTCTGATCTGGCTGAAATGAAGTATCTGGAGGCAGTTATCAAAGAAATCTTAAGACTGTACCCGAGTGTGCCGTTCATTGCCCGGGAGGTGACTGAGGACTTTATGTTAGGAGATGTCCTAGTAAAGAAAGGTACAGAAGTATCAATCCACATATACGACCTGCATCGTCTGCCAGAATTGTACCCAGACCCGGAGGCTTTCAAGCCGGAACGGTTCCTCAATCAACAACCGACACATCCCTACGCCTTTGTGCCTTTTAGTGCTGGACCTCGGAATTGTATAGGTCAAAGATTCGCGATGTTGGAAATGAAATGCATGCTGAGTGGAGTCTGCCGTAAGTTCAAGCTGTCACCCATTGTCCCGGGAGAAAGGCCAAAACTGCTCGCCGATATGGTGTTAAGACCAGTCGGACCTGTCTGTGTGAAGATGCATCAAAGATAGGTTTTAAGATAAGCATTACTGAATAGTTGTGAATTTTGTGATACATTAAAATTCTTTATTGTAAGTCAAAATATAATGTTATTATTATAGTTGTGATACTTTTACAAAAATCAAATTATGAGTTAATAAAAAAAGCAAGAATGACTACGTTTTAAAATTGA

>gene9203

ATACCTCTATATAGATAGAGTGAGCAGTCAATACGCTGTGCTGTGTGTGCGAACATTAAGGACGATTAGTCAACGCGAGTCGCGCAGTGAATCAATATGATTTTTCTGTACCTCATACTAGTGTTCATATTTCTCTTTTACTATGTTGTTTCACGTAAATATAATTACTGGAAAATTAGAAACGTGCCATATACGGAGCCAACATTTTTATTGGGAAATTTTACTGATATGTTTCTCGACAAGAAGTTTAACGGAGAAATAATCGACGATATTTGCAAGCAGTTTCCAGACAACAAGTACGTTGGAGCTTTCAACGGTATTGAACCTGTCCTAATTCCTAAAGATCCAGAAATAATCAAGTTGATCGTGACCAAGGACTTTTATTACTTTCATGGACGAGCGTTATCAAAATACAATGACAAGGAACCGGGTGGCTTGAACCTCTTCGGTACATTTGGTGATAATTGGAGGGTTATCCGGCAAAATATGACTCCATTGTTCACATCAGCCAAAATGAGAAACATGTTCCATCTTATAGAAAAATGTTCCCACGAATACGAAGCTATGATAGATCGAGAAATTAAGATTTCCAAGGAGCACGATGTGATGCCGATGACTACAAGGTTCACTATGGACTGTATTGGAGCCTGCGCTTTTGGTATAGATACTAAAACAATGACAGAAGGAGAGGACGATAATCCGTTCAGAAGGATAATACGTTCTTCAAAACCTAAGAAAAGTATGATTTATCAGGGTATGCTGCGATCTTTGTATCCTGAAATCTATTATGCTCTTGGTTTTAAGATGCCTAGCACCCAATTGGATTTTCTGCATAAGTTGGTCACTTCTGTGATGAAACAACGGGATAACAAACCTTCTGAGAGGAACGATTTTGTAGACCTCATTATGAGTTGGAAACAAAAGCATCAAATAGTGGGTGATAGCATCAGGAATATGAAAACTGGTGAACATGAAAAAGTGGTATTGGAAGCGAGCGATGATTTACTGATAGCTCAGTGCTACGTTTTCTTTGCTGCTGGTTTTAAAACATCAACTACTGCTCTAAGTTACACTTTATATGAAATCGCAAAACATGAAGATGTTCAGAATAAAGTATTTAATGAAATAGACTCGTATTTAGCTCGTCATAATAACAAAATAAACTACGATTGTGTTTCGGAGCTGCCATACTTGGACGCTGTCATAGATGAGACATTCCGGGTCTACCCAGTTCTCAGTGTAATACCAAGGGAGCTGATGGAGGACTACACAATGCCCGATGGAACGAAGTTGGATAAAGGTTTAACTGTACAATTGCCTGTATACCATCTTCACCATAACCCAAAATTCTACCCAGAACCTGAAGTATTTCGTCCAGAACGATTTATGGGTGAAGAGAAAAAGAAAATCAATCCGTATGTATATTTACCGTTTGGAGAAGGACCAAGAGTATGTATAGGTATGAGGTTCGCAAAGATGCAAATGTTGGCTGGTCTGATCACGATGTTGAAGAAGTATCGCCTGGAGTTAGCTGATGGTATGCCGACGAAGCTGAAGTTCAAGGCCCAGTCCTTTATCACGCACCCAGTAGGAGGAATCAGGATCAAATTTATTGAAAGAGAGGGATGGGAGAGTAGGGTGTTTGCTAAGAGTAGCTAAATTCATGGACAGTTGGCATCCCATCATGGATTTGTTCAGGCGTGTTTATGGAAATTGTAGTACCATTTTTTTATTGACAGCTTAGTTATCTGGAGAATTTGACACACATGAACAAAGCCATGATTTTTTAAAACGACGGAGTAAGAACTAAGGTCTTTAAATCAAAATTGGAATGATTTAAGCATAATGTTATATTGAATTATTTTTTAGTAAATCTTGGTGTGTCAAGCACTATAATTTTGTTTTTAAATGGAACTGAGATTTATCAGCTCAAATTTTTTAATAGTATGTGACTGTTTTTTTATTATTTGTATTGTTTACCTTAACATTGGTATTGTATTAGTTCCCAGCTAGCTGTAATCGAGGTTAATGACTAAGATTTTATTTTAATTGGTATCTATTGCAGATAAAAAGGTTTTAATAGTGACAATGTTTATAAAATACTGTAATTTTATTTTGTGAGATATCAGCATATTTAACAAAACATCTTTAGTGCTTGTATTAATTATTATATCAATTATACTTTAATACACAAAGC

>gene9331

TATTCAACCACATTGTATATTCAAACAATTGATAAGTCGCTCGTGTTTATTCGCGCGTCCAAAAATACATTACGATGGTATTATTACCATTAACATTAGTACTAGTCACTCTAGTTCTGGCGTGGTACTTCATTGGAAAGTACAATGAAAATTATTGGAAGAAGCGTGGTGTAACATTCTACACCAAAAACAAAGTAGTGGGACCCATGTGGGACTTCATGACATCACAAAATGCCCTGTTCGAGATCTTCGGAAAACTGTACAAAGAATACAGAAAAGAACCAGCGATCGGCATCGGTCAATTCTTAACTCCATCACTGTTTGTCATTGACCCGAACAATGTGCAACAAGTGCTATCAGGTGACTTCCAATCTTTTAATCACCGAGGAATCGAAAGCGTCGAAGGAGACACTTTGTCAGACAACATTACCTTTATGAGTGGTCCAAGATGGAAGCTGATGCGATCAAATATGACTCCATTGTTTACGGCAAACAAATTGAAGAACATGTATTACATCATGGACAAAAGTGCTCAAGACTTCGTGCAGTATTTAAAAAATAACTCCAAGTTGCAGCGTAGTGATTTATTCGAAACGCTTATGATGTTCTGTAATGCTGCTGTTTGTGGAGCTATCTTCGGTATTGGATCAGAGTCAGTCTTTGATACACCTTTCCTCAAGCTTGCCAAAAACTTATCACAATCGAATATACTGGTAAGAATGAAATTCATATTCTACACTTTGAGCCCGAAAATTTATACGGCGTTAGGTTTTAAACTTTTCAAAGAACATGAAGAATTCTTTGTTAAATCAATTGGTGAAGTGTTGAAACGAAGAGATCAAGAAAATGTTAAGAGACACGACTTCGCTGACATTGCTTTAGCTATACAGAAGAACGGTACCATGAGAGACCGTACAACAGGATACGAGATAGAGCCTACGACGGGTATACTCTCTGCACAGGCATTGTTCTTCTTCACTGCTGGTGTAGAGCCTTGTGCTAATGCGATCTATGCGACGTTGTATCTCCTGAGCTCTCATCCAGATATACTAGAAAAAGTGCATCAAGAAATTGATGAACATTTTGAAAAACATAGCAACAATATAACTTATGACGTAATTTGTGATCTGGAGTATACGGAAAAGGTATTAAGTGAGGCGATGAGAATCTTCCCTCCCATTGGATTTTTGACAAGACAATGTGTTCAAAATACTGTTTTGCCGATCGGTAATATTCCAGTGGAGAAAGGAACTAAAATATTCACACCTATTTATGATATCCATCATGACCCAGAATTGTATCCAGACCCAGAAGTGTTTGACCCTGAGCGGTTCTCCAAGGATAGGAGGCCTAACGACGACATTTACATGCCATTTGGGATGGGTAACAGGACGTGTATAGGTGCCAGGTACGCCAAGTTGCAAGTGCAGGCTGGCTTGGTGCACGTCTTACGTAACTTCACTGTTCAACTCTCGAAAACCAGTGGAAAGTTGAAATTTGCGCATGATCCTCTTAATGTAAGACTCAGAAATATCGGTTTTGAGTTGATTCCTCGAGCAATTAAATAGACTTTCACGTATCAAAGAACAGGGGGAACTCCGCCTCTAAATTAATACTTAGTTAAATTTTACAACTAGTGATACCAAATATTTTTATGTGAAGGACATTCCTGTTATGTGGGAGGTGGTATTTTCCCCAAATTCACCATAACTGACTGATGGGGGGCATTGACTGATTACTGATGTAAAAAAATGGCTTTTTGTTCAACTGTACAATGTATTATTACGTGTATTATGTATATTGTAGTACTGTAAGATTAACTGGTAATATCATTATAATGTAGTCTGTACGCACTGTTATTTTGTTTTGGTTTAAAAAAACTTGTCAACTTAGTAAATGTTATGCGTTTATAAAATATTGT

>gene9332

AAAAAGATAACTGATCAGCAGGTAGGTATTTTGACTAATTTAAACCGAATAACAATGATTTTCAAAAATGTTATTTAAATAGCAATCAAAATAATGACTCAGCAAAAATAAGAATGATAGAACCGGTGAAAACTAGAGATATCTTCGGAACACTTATCCGTGGCTACCACTGATAACAGCTGAGTATAAATAACGAGCAAAATATTATTATCTTCATATTATGTAAAACCACTAACGAATTAGCAAGTGTTTGTTGTGATTTTTGTAATAACTCGTAACAGTGAAGACACAATGTTATTCCTACCATTAAGTCTTATAGCAGTGGGCTTACTACTGGCTTGGTACCTAATAGGAAAGCGCAATGAGGGCTACTGGAAAAAGCGTGGTGTAAAGTTCTATAGCAAAAATAAAGTGGCTGGACCATTCTGGGATTTCCTAACATCACAACGTGCATTTTTTGAAATCTTGGGCGATCTATACGAAAAATACAGACAAGAACCAGCTATCGGTATTGGTCAATTTTTAACTCCGACACTGTTTGTCATCGATCCGAAGAATGTGCAGCAAGTATTATCAGGTGACTTCCAGTCTTTTCACCACAGAGGCATTGAAAGTATTGAAGGGGACCAATTGAGTGATAACATTATTATGATGAATGGTCCAAGATGGAAGCTGATGCGACAGAATATGACTCCATTGTTTACGGCAAACAAATTGAAGAACATGTATTATATCATGGATAAAAGCGCCCAAGACTTCGTGCAGTACTTGAAAGACAATCCAAAGACCCATGCTGGTAATTTATTTGAAACTCTTATGATGTTCTGTAATGCTGCAGTTTGTGGAGCTATCTTCGGCATTGGATCAGAGTCAATCTTCGACACACCTTTCCTGAAGGTTGCCAAAAGCTTGTCACAATCCAATCTTAGTATCAGGTTAAAATTCATATTAGTCAATCTCAGCCCAAAGTTGTTTAACCTATTAGGATTAAAAGTTTTCAACGAGTACGAAGATTTCTTTATTGGCTCAATAAGTAAAGTGATGAAATCTAGAGAACAAGAAAAAGTAAAGAGACACGACTTTGCTGAAATTGCTTTAGCTATACAGAAGAACGGTACCATGAGAGACCGTACAACAGGATACGAGATAGAGCCTACGACGGGTATACTCTCTGCACAGGCATTGTTCTTCTTCACTGCTGGTGTAGAGCCTTGTGCTAATGCGATCTTTTCAACATTAATTCTTTTAGGCAGTCACCCAGATGTACTTGAAAGAGTTCATCAAGAAATTGACGAACATTTTGAAAAGCACAGCAATAACATAACTTACGATGTTATTTGTGATATGAAATACATAGATATGGTGCTGAGCGAAGCAATGAGAGTATTCCCTCCAATTGGTCACTTGACTAGACAATGTGTTCAAAACACAGTTCTGCCTGTTGGTAACATCCCAGTAGAAAAAGGGACAAAAATGTTCACCCCAATTTATGCTATTCATCACGACCCAGAATTGTATCCAGATCCAGAAGTCTTCGACCCGGAGAGATTCTCTAAGGATAGGAGGCCCAGAGATGACATTTATATGCCATTTGGTATGGGCAACAGGACATGCATCGGTGAGAGGTATGCCAAATTACAAGTGCAGGCTGGTTTAGTTCATGTCTTGCGAAACTTCACTGTTAAACCGCAGACACATCAAAAAGTAATATTTGCTCATGATCCACTTAATGTGAGACCAAGAAATATCGATGTGCAAATCATTCCGAGAAACATTTAAAAGGTTTCTTGGATATGTTGAGGATTTTAATTTTATTATTTTAAATTCTTATGGTTGACTTCGCCTGGTGTTGTATATCTAGAGGAGGAAACAATAAGCTTTCTTTTACTGGAACACTATATGAGACTGTTGCGTGATATGATCATGAAAAAACATCGTGACATATAAAAAAATATAATTCTTCACCTGCCATGTCTTTATTGTGATTAATTAGACACTCAAGTTGTCTAACTAAAGGAAGACATGTTGTATGTTGAAGATGGTTGGCATGATAAAGGGCCAATGAAGGCGCAATAGTAATACTGACAGGGCATAGCTTCAACACAATCATGCGCGAGCGGTGCACGTCATTAGTGAACAGGGTGCGGGGCAGCCGCAATAGTATCCTGAGCGCCTTTGCTGAACGTTTTGACTGTCAGTACATGAACCACTGTTGTATGGTTCATGTACTGGCTAATGTGAAATTATTGTTTTGAGTTGGGAGATTATTTTAGTGATGACTGACTTGTGATTGTGATTTTTAATATGTGATTGTGACTTTTATATTGTATTATGGTTTGTTATGTGTAATAGATGCGTAATTAATGATCTATATAATTGATTGAAATAAATGGTAATAATAATAATAGTACAAATGGTTTAGTGTGATCAATATGATAATCAATTTACATGTTTGTTTTTTATAAAATTGTTATCGATTTAGTTTATTTGTAAATACTGATAAAAATTTAGACTTAAGT

>gene9333

TTATTTACATAACGTTTAACATCCGAACGAGTAACAAGTGAACAAGTGGTTTTTATTAATATTCGAGACAAAATGTTGTTTCTACCTTTGAGTTTGATAGCAGTGGGGTTAGTACTGACTTGGTACCTAATAGGAAAGCACAATGAGAGGTACTGGAAAAAGCGAGGTGTAAAGTTCTACAGTAAAAATAAAGTGATTGGACCGTACTGGGATTATTTCTTAACGCAACGTGCATTGTTTGAGGTCTTAACAGACTTGTACAAGAAATATAGACACGAACCAGCTATCGGTATTGGTCAGTTCTTAACACCGTCAATCTTTGTCATCGATCCGCAGAATGTGCAGCAAGTTTTATCAGGTGACTTCCAGTCTTTTAACCATAGAGGCCTTGAAAGTATTGAAGGAGACCAATTGACAGACAATATTCTTATGATGAATGGTCCAAGGTGGAAGTTAATGCGACAGAATATGACTCCATTGTTCACGGCAAACAAATTGAAGAACATGTATTACATCATGGACAAATGTGCTCAAGACTTTGCGAACTATTTGAAAAACAACCCCAAGACACGTGATGGTAATTTGTTTGAAACCCTTATGATGTTCTGTAACGCTGCTGTTTGTGGGGCTATCTTTGGCATTGGATCAGAGTCAATCTTCGACACACCTTTCCTCAAGCTCGCCAAAAGCATATCGCAATCCAATTTTAAAATGAGAATGAAGTTCGTTATTTTTAGTATCAGTCCAAAGTTGTTTAAATTATTGAGATTACAAGCTTTCAAGGAGATTGAAGACTTCTTCATTGGTTCCATAAGTCAAGTGATGAAATCAAGAGAAAATGAAAATGTAAAGAGACACGACTTTGCTGAAATTGCTTTAGCTATACAGAAGAACGGTACCATGAGAGATCGTACAACAGGTTGCGAGATAGAGCCTACTACCGGCATACTCTCTGCACAGGCATTTTTCTTCTTTAGTGCTGGTGTAGAGCCTTGTGCTAATGCAATTTTTTCAACATTAATTCTTTTAAGCAGTCACCAAGATATACTAGAACGAGTCCACCAAGAAATTGACGAACATTTTGAAAAGCACAGCAACAATATCACCTATGACATTATAGCTGAAATGAAGTACACAGATAAGGTGCTGAGTGAGGCAATGAGAATATTCCCTCCAATTGGTCACTTGACTAGACAATGTGTTCAAAATACAGTCCTGCCTGTTGGTAACATACCAATAGAGAAAGGGACAAAAATGTATACCCCAATTTATGCTATTCATCACGACCCAGAATTGTATCCAGACCCAGACGTGTTCGACCCGGAGCGATTCTCCAATGATAGGAAACCTAAAGACAATATTTACATGCCATTTGGCATGGGCAACAGGACATGCATCGGTGAGAGGTATGCCAAATTACAAGTGCAGGCTGGTTTAGTTCATGTCTTACGGAACTTCACTGTCAAACCGCAGAAACATGTAAAGGTAACATTTGCTCATGATCCACTTAACGTGAGACCGAGAAATTGCGATGTCCAATTAATTCCCAGAAATATCAAATAGTTATTTTAATTGTATTTTAACTTATTGCAATCATTCTATTGGTTGCGGTTCGCATCGTGTACTGTGCAAGTTCTATCATGTGCTTGATAGAACTTGCTCAGTCTAATATACAAGGAGACAAAATGTTGTATATTATGATATTTAAACTAAAGGAGTATGTCTACTTTGTAGTTTGTAGTAGAGGGACACCTCTACTGCCGATGTTTATTGATTAATAATTCTAATTGACGATGTTGTGATAGCCTAGCAATAGTTCAATATATTCAATTTGTTATATTCATTGGAATAGAGATTGTGAAAATAATTAGAACATTGTTAATCACTTTAAATGCTTTAGCCAACATCTCTATGTTGTGTAATTTTATACTTATTTGAAGACATTATTAGGTCACTATATTATGTCATATTGCAGTACTTACTAGTGTTAAGTTTGTATTTTGAAGTAGTTTTTAAGAATCAAATTTAACTCGTAATTCGTAATGTTTATAA

>gene9334

TCGAGAAGTCGGCTGCCTTTATACAGTAATAACTTGCATACAAAAAAGTAACAATGTTGTTGTTACCAATTACTTTAGTCCTAATTACTCTAGTACTAGCATGGTACTTCGTTGGGACATACAATGAAAATTATTGGAAAAAACGTGGTGTGGTCTTCTACAGCAAAAACAAAGTCTTAGGACCAATGTGGGATTTCTTAGTATCACCACGTCCCCTGTTCCAAGTCTTTGGAGATTTGTATAAACAATACAGACATGAACCAGCCATTGGTTTGGGCCAGTTATTAACTCCAGCGATGTTTGTCATCAATCAGAAAAATGTTCAGCAAGTTCTATCAGGTGACTTCCAATCTTTTAATCACAGAGGCATTGAAAGTATTGAAGGTGACCAACTAAGTGATAATATTACCTTCATGAATGGTCCAAGATGGAAGTTGATGCGATCAAATATGACTCCATTGTTCACGGCAAATAAATTGAAGAACATGTATTATATCATGGACAAAAGTGCTCAAGACTTCGTGCAATATTTGAAAAACAATCCCAAGACGACCGAAGGTAATTTATTTGAAACTCTTATGATGTTCTGTAATGCTGCTGTTTGTGGTGCTATCTTCGGCATTGGATCAGAGTCAATCTTTGATACACCTTTCCTCAATGTTGCGAAAGGCTTATCCCAATCAAATTTATCGATGAGGTTGAAATTCGTTACATTTCTTCTAAGCCCGAAAGTGTGTTCAATTTTGGGTTTAAAACTTTTCAAAGAACATGAAGATTTCTTTGTTAAATCAATTGGTGAAGTATTGAAACGAAGAGATCAAGAAAATGTTAAGAGACACGACTTCGCTGACATTGCTTTAGCTATACAGAAGAACGGTACCATGAGAGACCGTACAACAGGATACGAGATAGAGCCTACGACGGGTATACTCTCTGCACAGGCATTGTTCTTCTTCACTGCTGGTGTAGAGCCTTGTGCTAATGCAATCTATGCAACGTTATATCTTCTTAGTGTACACCCAGAAATATTAAAAAAAGTGCACCGAGAGATAGATGAACATTTTGAAAAATACAATAACGATGTAACCTACGACGTCATTAATGAAATGGAGTATACAGAAAAGGCTCTGAGTGAAGCGATGAGAATATTCCCTCCAATTGGATTTTTGTCAAGACAATGTGTTCGGGATACTGTCTTACCTGTTGGTAACATTCGAGTACAGAAAGGAACTAAAATGTTCACTCCAATTTATGATATCCATCATGATCCAGAACTGTACCCAGATCCAGAAGTGTTTGATCCGGAACGATTCTCCAAGGACAGGAGACCCAATGACGATATTTATATGCCATTTGGGATGGGTAACAGGACGTGTATAGGTGCCAGGTACGCCAAATTGCAAGTGCAGGCTGGCTTGGTGCATGTCTTACGTAACTTCACTGTTACATCGATGCAAACAGATAAAACGGTAACATTTACGCACGACGCATTTAATGTGAGACTAAAAAATGTTGATGTTCAGCTTATTCCCAGAAATGTGAAATAGATCGATTTGTGACTCAAAGTGGCTGCTTACCAAATGGAACTGTGTTAAACCGAATGACTTCGTAAGTTGTACCCATGTATGTAGAGGTAAATAATTGAGCTTTTATGTTTTAAACGATTTTTTTGATTATTCTTAGTTCAACGAAACGCATGAAGCTTAAAATTTAAAAAAATAATGTGTAAAAGTTAAATTTCATTCACTCCAGGATATAATTCCCATGGTAGGTATA

>gene9509

CCTCGGGATGTGGTTCTATTTGTGCAGTTAGCGCGTGGATCTGTCTGTCAACACAGTCGCTCTTAATTATATTAAGTGCTTCTGTTATTAATAAATAATTTATTTAAATATTTATTGAATATCTTTAACCGGTTCATAATGATTGTCCTAACAATTTTGGCCGCGTTATTTTTACCTTATTTAATTATTTATTTGTACAAAAATGCGTATAACAAGCCGAAGAATTTTCCACCAGGCCCACCGAGTCTGCCAGTATACGGTGCCTTCTGGATAGTTCTGGCCCATGGGTTCACTGACCTCGCAACAGCTTTTAAGAAGCTGGGAGAGAAATACAAAACGAAGATCATAGGTCTCTATATGGGACCTGTTCCAACCATAGTGCTGAATGACCCTGAGCTGATCAAGGAAATGCTAAACCGGGAGGAGTTCGACGGACGGATGGACATCATAATATTCAGATTGCGGTCATTTTGGAAGAAACTGGGTATATTCTTCACGGACGGATACTTCTGGCATCAGCAAAGAAGATTTTCACTCCGTTATTTGAGAGACTATGGGTTTGGAAGGCGGTGTGAAGACCTGGAGTCAGTAGTTGCGACTGAAGTCAAGGAGATGATAGACCTGAGGATTAGTGGACCGAAGTATCCTGCTGAAATGGATATCGTCAAGGGTGACCTAGTCTACATGCCATACTTCTTTGCAATACCGTTCCTTAATGGTATGCTCCAAATATTCTCTCGCAGCACTCTGCCAAGGTCGGAGTACAAAGCACTATGGGCTCTGACCCGTGGTACCGTCTTGTTCCAACGGAGCTCGACTGATATGGGAGGTGCCCTCTCCTTGACTCCTTGGCTGAAAGACATTCTGCCAAACTACAGTGGGTACAATGATCTGGTGAAAGGAAATGAATACCTGTTGAATTTCTTCAAGAAATTAGTAAACGAAGCTATGGAAACCCATGACGAGACTTACGACCGTCACTTCTTAGACATGTACATCACGAAAATGAAACAAGAATTGCGGGAGAAAGACAAGACTACATACTCAGTGGACCAGCTGATCCTAACGTGCACAGACTACACATTCCCCGCGGCGTCAGCTGTGCAGTTCGTGCTGACGATCCTTGTGGAGCGTCTGCTACTGCAGCCTGAGATCCAGGACAAGATCCATGAGGAAATCGACCGCGTCGTCGGCAGAGACCGTCTCCCGAATTTGGATGACAGAAGAAATATGCCATATTTGGAGGCTTGCATCCGAGAGACTATGCGGTTCGATACCACAGTACCTCTGTCGGTGCCTCACCGAGCAATGAAGGATACCACCTTAGCAGGATACGATGTGCCAGAGGGCACGATGGTGAGCGCTAACCTGACGCTACTACACATGGACAAGGATATTTGGGGTGACCCCGAAAACTTCCGTCCCGAAAGATTCCTCAAGGACGGAGAGCTTGACCTCGCCAGCGACAAGTCATTGCCATTTGGAGCAGGCAGAAGATTATGTGCTGGTGAGACCTATGCTAGACAGTCCATGTTCCAAGTATTTGCTGGCTTCATGCAAACTTTCCATGTATCCACCGCTGACGGCAAACCGCTGCTGAAGCCTTCTAAAAGGATCCAAGGCATCATCACCACCATTCCTGAGTTCTGGGTTAAAGTTACTGTCCGAGAATGAAAAACAACAAACAAGAATTTTGCCACAAAATTCAAAAATAGGTGTCTATGATCTCTCTTGTTTTTCGCCAAAATGTAATCAAAGGTTTGGATGTTTACTTTAACTTAAACTTTAGTTACTTTTTAACCCAAAAAAAAACTATTGTTTCCGTTATCTACGTACCTGTATTAAAAAATAATGTGATCACATTTTTGGATTTCTGTACCTCCTTTCTATTTTAATGATCTACTTTACAGAACTACTATAAACTGCCTATATGTATTTCCCTTAAGTTCAATTACCATCTAATAATTAATAACAATAATGATGACCGAACTCCATGAAACTACACACAGTAGACACAATCGACTTACCAAGATTAATTTATGCATAACGTAAAATAAGTTTTGACAGCTCAAAATGACATTGACATAGTTGAAGAGTTAGCCTTGACAGAGGCGACTCATTCAAGATATATTATTTCAACCTGTTTTTGTTTAAATGCGTATACTGTTAGGTATTATTACTTAACATAATAATCCAAGTAACGATATCTCATTATAAGCATATCTCATTAGCATTT
