# Supplementary figures and images for: Identification of transcriptome and fluralaner responsive genes in the common cutworm Spodoptera litura Fabricius, based on RNA-seq
Source: BMC Genomics. 2020 Feb 3;21:120. doi: 10.1186/s12864-020-6533-0 (PMC6998375; doi:10.1186/s12864-020-6533-0)

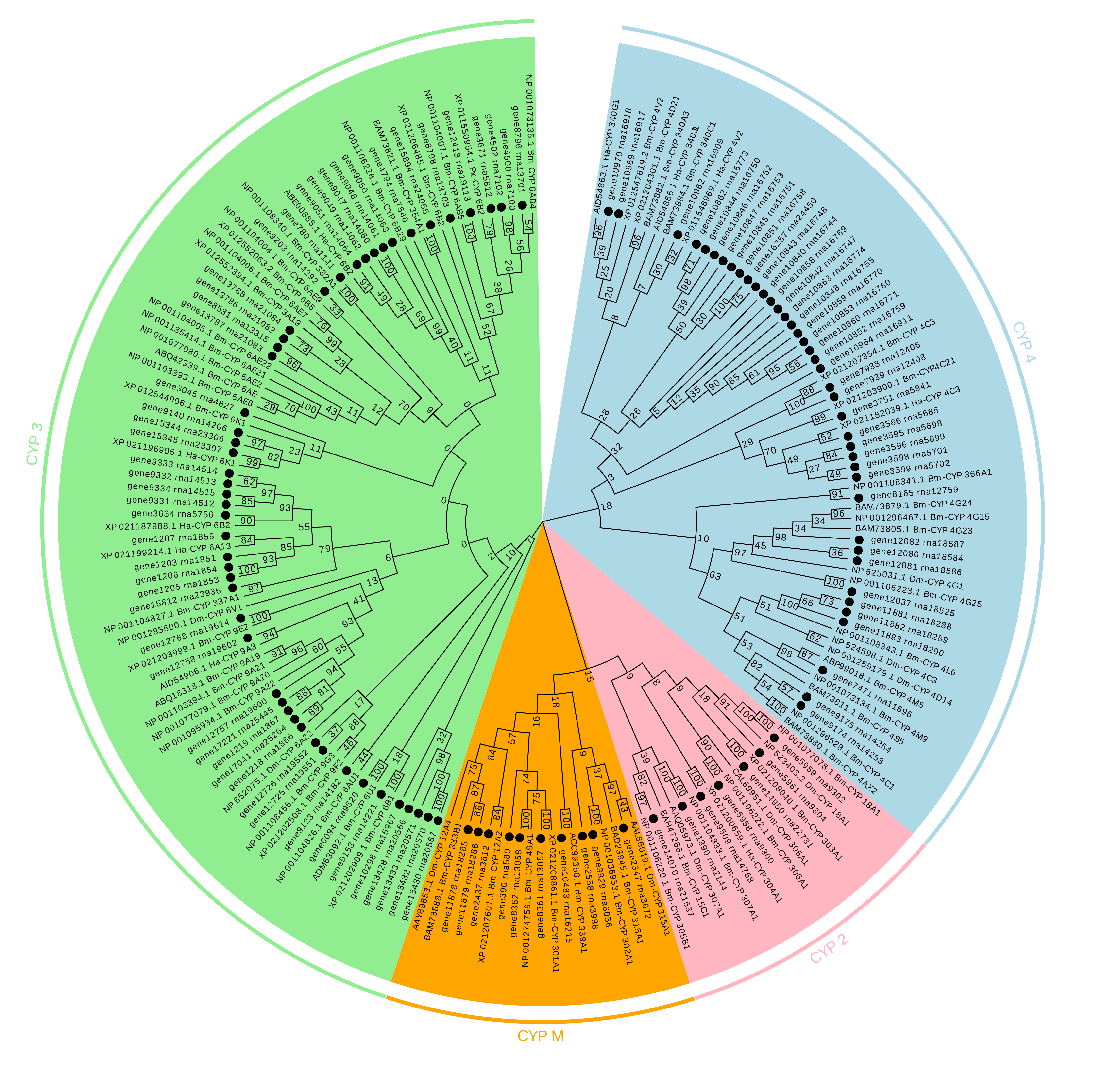

Supplement: Supplementary file 4 — Additional file 4. Neighbor-joining phylogenetic analysis of the P450s genes from D. melanogaster, H. armigera, P. xylostella, S. litura and B. mori. [file 12864_2020_6533_MOESM4_ESM.png]

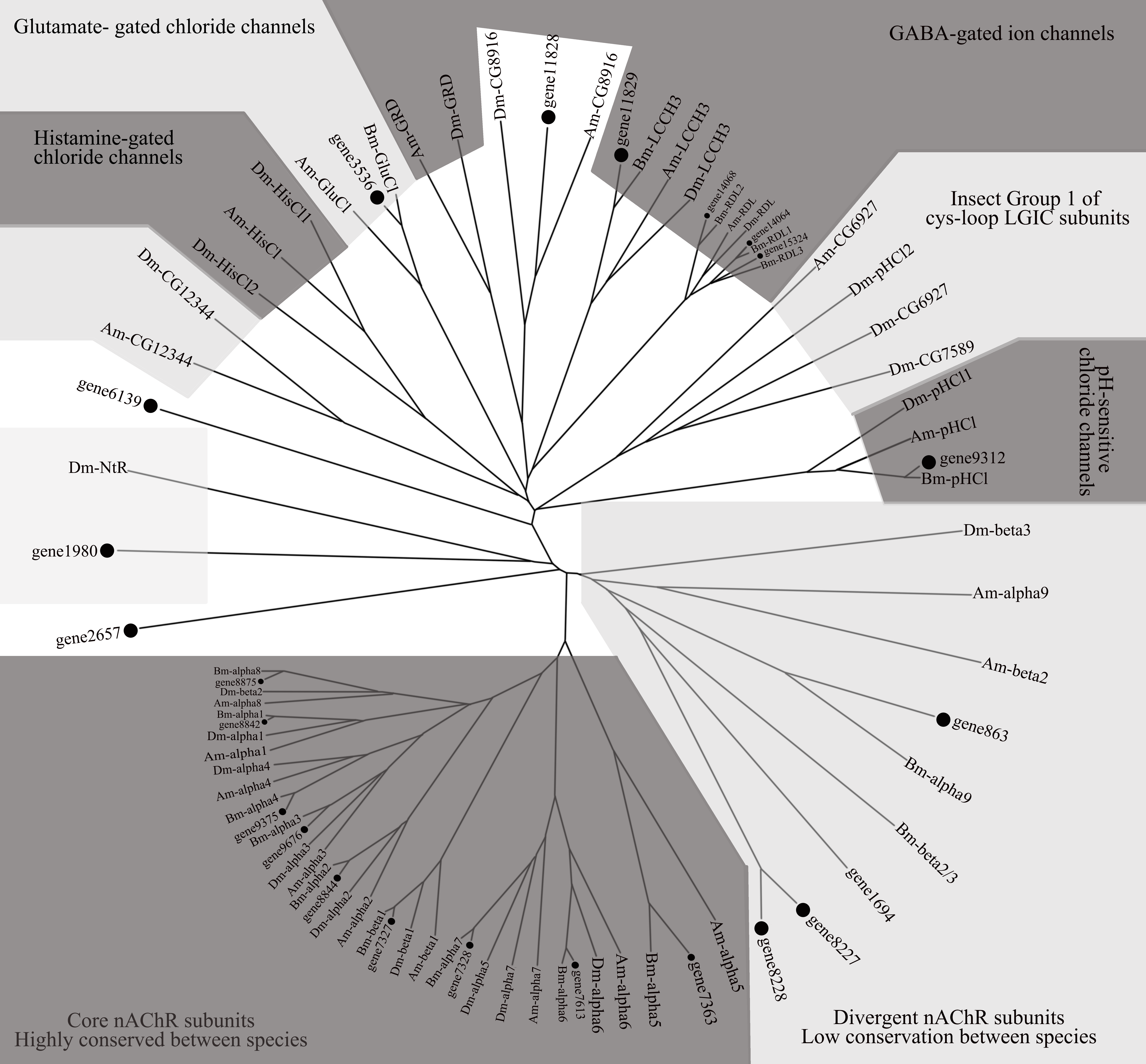

Supplement: Supplementary file 10 — Additional file 10. Neighbor-joining phylogenetic analysis of the cys-loop ligand-gated ion channel superfamily genes from S. litura and other species. The subunits sequence used are as follow: Am-alpha1 (AAY87890.1), Am-alpha2 (AAS48080.1), Am-alpha3 (AAY87891.1), Am-alpha4 (AAY87892.1), Am-alpha5 (AJE70263.1), Am-alpha6 (AAY87894.1), Am-alpha7 (AAR92109.1), Am-alpha8 (NP_001011575.1), Am-alpha9 (AAY87896.1), Am-beta1 (AAY87897.1), Am-beta2 (AAY87898.1), Am-GluCl (ABG75737.1), Am-RDL (AJE68941.1), Am-GRD (AJE68942.1), Am-LCCH3 (AJE68943.1), Am-CG8916 (NP_001071290.1), Am-HisCl (ABG75739.1), Am-pHCl (ABG75741.1), Am-CG6927 (ABG75747.1), Am-CG12344 (ABG75746.1), Dm-alpha1 (AGB96296.1), Dm-alpha2 (NP_524482.1), Dm-alpha3 (NP_525079.3), Dm-alpha4 (NP_001097669.2), Dm-alpha5 (NP_995708.1), Dm-alpha6 (NP_995674.1), Dm-alpha7 (NP_996514.1), Dm-beta1 (NP_523927.2), Dm-beta2 (NP_524483.1), Dm-beta3 (AAF51485.1), Dm-RDL (NP_523991.2), Dm-LCCH3 (NP_996469.1), Dm-GRD (CAA55144.1), Dm-CG8916 (AAF48539.4), Dm-HisCl1 (AAL74413.1), Dm-HisCl2 (AAL74414.1), Dm-NtR (NP_651958.2), Dm-pHCl1 (NP_001034025.2), Dm-pHCl2 (NP_651861.1), Dm-CG6927 (AAF45992.1), Dm-CG7589 (AAF49337.2), Dm-CG12344 (NP_610619.2), Bm-alpha1 (XP_021203289.1), Bm-alpha2 (NP_001103397.1), Bm-alpha3 (NP_001103387.2), Bm-alpha4 (NP_001166816.1), Bm-alpha5 (NP_001166811.1), Bm-alpha6 (NP_001166813.1), Bm-alpha7 (NP_001166818.1), Bm-alpha8 (NP_001166817.1), Bm-alpha9 (ABV72691.1), Bm-beta1 (ABV72692.1), Bm-beta2 (ABV72693.1), Bm-beta3 (ABV45510.1), Bm-RDL1 (ADM88014.1), Bm-RDL2 (NP_001182629.1), Bm-RDL3 (NP_001182630.1), Bm-LCCH3 (BAT57341.1), Bm-GluCl (BAO58781.1), Bm-pHCl (BAX77827.1). [file 12864_2020_6533_MOESM10_ESM.png]

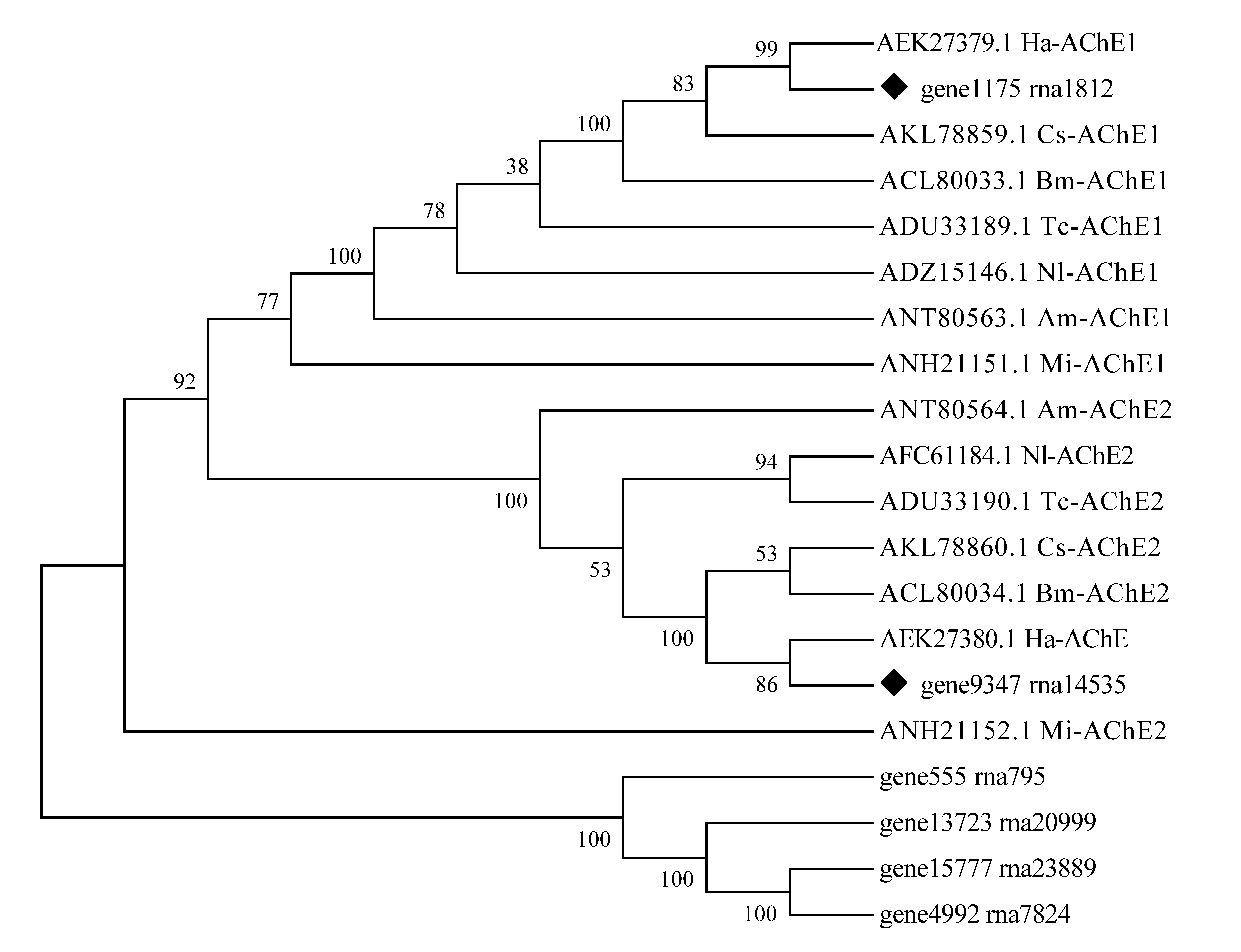

Supplement: Supplementary file 11 — Additional file 11. Phylogenetic analysis of the AChE genes. [file 12864_2020_6533_MOESM11_ESM.png]
