# Supplementary material for: Identification of transcriptome and fluralaner responsive genes in the common cutworm Spodoptera litura Fabricius, based on RNA-seq
Source: BMC Genomics. 2020 Feb 3;21:120. doi: 10.1186/s12864-020-6533-0 (PMC6998375; doi:10.1186/s12864-020-6533-0)
Supplement: Supplementary file 5 — Additional file 5. Glutathione S-transferase nucleotide sequences of the S. litura transcriptome. [file 12864_2020_6533_MOESM5_ESM.docx]

**Additional file 5**: Glutathione S-transferase nucleotide sequences of the *S. litura* transcriptome

>gene10017

ATTGTTGTTGACACTATGGCTCCCGTTTACCAATTGAAGTTTTTTAATTTTATGGGCGTTGCTGAGCCCATTAGGTACTTATTTGCCTATGGAGGTATCAGCTACAAGAATGTCGAAGTCAACAAGGATTCGGACTGGCCAGATGTACAGAAATCTGTACCCTTTGGCAAGCTGCCAGTTCTGGAGGTAGACAAGAAGGTGCTCTACCAGTCTGTGGCTATCAGTCGCTACCTTGGAAAGTTAGTAGGCTTGGTACCCGCTGATGACTTGGATGCAGCTGAACTAGATGCTATTGCGTTGTCTGTATACGATTTTGGCGCAAAGGTCCACGAATGGTACCACGAGAGCCAGAAAGAAAGGAAAGAATACTTGCAAGTGGAATTAGAGGACATTTGGCTATCGAAATATCTTCGCGAATTTGAAGATATTGTTCAGAAGAAAGGAAGCTATTTTGGTGGCCAGAAAATAAGCTGGGCTGACTTATACTTCGTGGGCATCATCGAGTCCATTGAAGGCATGTGGGGTTCCAAGCTCTTGGAGAAGCAGTATCCATTGCTGTACAGGATTTACGTCCATGTCCACGAGATCCCTGCCATTGACACCTACGTCAGTAACAGACCTCATTACAAATACTGAAAATAATATTTCCACACTCGAATAAATATGTAATGTACTGAATTTATAAAA

>gene10018

GCAGTGACTACCGCAATTGACACAAGCACGGCGCCCATTATTCAAGGTCAAAATGCCGAAAAAATTATCGTACTTCAATTTGAACGGTCTTGCCGAACCCATAAGGTACATTCTTCATTACACCAATCAAGAGTTTGAGGATGTTAGATACGATGTGTACAGTTGGCCAGACCCGAAAATTAAAGCAAGTCTTCCCTATGGTCAGTTCCCAGTATACGAAGAAGATGGCCGTGTTCTTACTCAAAGCCTGGCCATCACCAAGTATGTGGCAAGAGGAACAGATCTAGTACCAACTGACCCTTGGGATAATGCTTTCATAGAGAGTGTCGTATTCTCCATCATGGATTACTGGAAAAATGTCGTGCCAACTGTGAGAGAACCCGATCCAGCAAAATACACAGAATTAAGAAATGAAATAATCGAAAAGAACATTCCATACTACTTCTCAAGATTTAACAAACATTTAGAAGATAATGGTGGATTTTTGAATGGCAAGCTCTCCTGGGGTGACTTCGTATTCTGTGGGGTGATTGAAGCCAGCGACATGTTTCTGGGCATCGAATTGAGCAAGGATTATCCAGCTGTTGGTACTTTGGTGCAACACATCAGGAATTTACCGGGAGTGAAGGAGTATGTCAAGGCTATAGGACCCTACACCATTGACCTTGCTAGATTTAAGTACAAGAAATGAAGTGTTTTTATATATAAATATTATACGAATTGTATCCATGTTCAAGGAGAATAAAATATGAAAAATGA

>gene10019

TTGCTCAGTACGCTCTAATCATTAAATCAGTCGAGAACAATATTTCGCGGTACTATTCAACAATGGCCAAAAAACTGCATTATTTTAATTTTATCGCTATAGCTGAGCCTATAAGGTATATTCTTCATTACACTAAGCAAGAATTTGAGGATGTCAGGCATGATCATAGATTTTGGCCAAACCCAGAGTTTAAAGAGAAATTACCCTTCGGACAATTTCCTCTTTATGAAGAAGGAGATCGTATGCTAACCCAATCACTGGCTATCGCAAAGTATGTGGCTAGAGGCACAGATCTCATTCCATCTGATCCTTGGACGCAAGCTGTTTTGGATGCAGCTGTATACACAATCTACGATTATTGGTCAAAGGTTGTGACTTTCATCAGAGAAAAAGACCCTGAAAAAAAGAAAGAATTGAAGCGTGAACTCTTGGATGAAACTATAGACTATTTCTTCTCAAGACTTGACAAAGATCTGAAGGAAAACGGTGGCTACTTTAGTGGAAAGTTATCCTGGGTGGAGTTTGTCCTATGCGGCCTAGTAGAGGCAAGCAACTACTTCTTGGACACTGAATTGGAAAAGAAATACCCAAGGGTCGAAGCTTTAATTAAAAAGATAAAGAGTCTACCGGGAGTCAAAGAGTATGTTGCTGCGAGAGGGCCTAATGTTTTTAAACAATGATTAATAAATTATTTCAGAAAAACATACCGAAGTACCTACCGGTAGAAAATGCATTTAATAATGTTTTTTATTTATCTGCCTGTATATGTAATAGAAAAGTTTACAAGTTCATGGTTCATCTGACACAAATATACAAAGCTGAAATAAACATATTAAATACATAA

>gene10093

TTGGAGGGAGATCTGGGTGTTTTGTCCTCGACATGCGTCATGCATTCGAAGCTGCTATATAACAGTTGACCTCCCTCCTCTCGCAGTTATTCACTCCGACAGTCCCGTACCGGCTGCACGCGCTGTGTTTCTTGTCAAAATCATAGTTTAAAAAAAAACCACAATGCCCATCGATCTATACTACGTGCCAGGCTCCGCTCCCTGCCGCGCCGTGCTCCTCACCGCCAAGGCTCTTAACCTCAACCTGAACCTCAAACTAGTGGATCTGCACCATGGAGAACATCTTAAGCCAGAATATATCAAGCTTAACCCTCAACACACAGTGCCCACCCTAGTGGACGATGGGTTCTCAATCTGGGAGTCGCGCGCCATCATCACATACTTGGTGAACAAATACGGCAAGGGCAGCAGCTTGTATCCTGAGGAACCCAAAGCCAGGGCCCTGGTAGACCAGCGCCTGTACTTCGACATCGGCACTCTGTATCAGAGATTTGCTGATTACTTTTACCCACAAGTGTTCGGTGGCGCCCCCGCTGACAAAGACAAGCTGGCCAAGATTGAAGACGCTCTGCAACTCCTGAACACATTCCTCGAAGGTCAGAAGTTCTGTGCTGGTCCCACCCTGACCCTCGCTGACCTCAGTCTCGTCGCCAGCGTGTCTAGCTTCGAAGCATCTGATGTGGACTTCAAGAAATACCCTAACATTAAGAGATGGTATGAAACAGTGAAGAGCTCAGCGCCTGGTTATTTGGAGGCTAATGAGAAAGGCCTGGATGCCTTCAAGGGTCTCGTCAACAGCATGATGAAGAAGTAACTGGAGTTTACACGAGATGTGTACTCCATTCCTTTATGCAGATGACAATAATGGACGAAGGTGCAATAAGTGCTCAATTAAAAATTAAATTGGGCCTTTATTGGGCCTTCGTTCATCCAGAAAATAACACCAAATTACAAAATGTTTGCACTTTTCTTTGGCTTCTACTATCCATATCTTGCCATGGATTTGGAATGATTTCTTCCAATTTTTGGATAAATGAATGGAGCTCTCCTATTACTCTACACAGCACGAATGCTGTCACTTTCACAAAATGTTGTTTTTTCAAGTTACACCGTTTTACATTCTGATTTTATTTAAGTTTAGGTTATAAGGCACAAATAAAAGTTTAAAATATA

>gene10094

GTGTAACAAGGCTAATAGCATTGAGTCAAGGCTGGCCGGTTTCTGTATGTATATTGTCATCGACTGCCGCAGTTATTATCTACATTGAACACGGAGAGCACGACTGACACAAAATGGCTTTAGCTCTATACTACACCGCTGGGTCCGCGCCATGCCGTCTGGTCCTGCTAGTAGCGGCAGCCCTCAACATCCAGCTGGATTTGAATCCCGTCAACTTGCGAGCGGGAGAACAGTTCAATCCAGAATTTTTGAAGCTGAACCCCCAACACACAGTGCCAACCCTCGTCGACGGTGACTTCTCACTTTGGGAGTCTCGCGCCATCAGCAGATACTTGGTCTCCAAATATGGCGACGAGAACAACAGCCTGTACCCAAGTGATCCTCAGGCCAGGGCTGTAGTTGATCAGAGATTGGACTTCGATTTGGGCACATTGTATCCAAGATTTGGAGGGTATTTTTATCCACAAATCTTCGGTGGAGCGAAGGCTGATGAAGCTCTTCTGAAGAAATTGGAAGAAGCTCTAGTGTTCCTGAACACATTCTTAGAGGGCAACACATATGCTGCCGGGGATAAGTTGACCTTGGCTGACCTCAGTCTTGTGGCCACAGTGTCTACGATTGACGCTACCGGCATCGTCAGCTTGGACAAATATCCTAATGTTCAGAAATGGTTCGAACTGGTGAAGACATCGGCACCTGGATATGATAAGGCCAACCAGGCCGGTATTGATGAATTCAAAGCTCTGCTAGCTTCATTCAAAGCAAAAACTGAGTTGTAAGTTCCTCAATAGTAATAAGTAAAACTTCGCATTTATTTATAAATTGTAATACGTTATCGCTTGTTTCTTTTCCAACTTTACGGAACTTTAGTTTCAGCTTTGATTCATTTAATTCTAAGCGCCTACGAATATTTTACACAAAGCGACTGAAACCGTTATAATACTATTGTATTGTACTCGAAATAAAACACTAAAAATATA

>gene10811

GTGTATCAGGATGATAAGCGAATGGCAATGTTTGGAACAAGTTCAGTTCGTAATACATCGCCGTCGACGCCAGACGTCATGACGATTATAATACACAAAACTGATGTCAGTCCGCCGGCTCGCGCGACGCTCATCCTCGTAGACTTACTCGGTCTTAAAATCGAAACTCGCGAAGTGAATCTACCTACAAGACAACAATTCAACCCAGAGTACTTAGAGAAGAATCCATTACACACAGTTCCATTATTAGAAGATGACGGTCTAGTTCTTCAAGACAGCCATGCAATCATGATATACCTAATGTCAAAATATGGTATAGAACACGAGAGTTTGTACCCTAAAGATTTGGCAGCAAGGGCGATAGTCAATCAACGTTTGTTTTTTGACGCGTCCATACTATTCCCAAGGTTGAAGACTGTTATTTATAGTACTGTGAAGCATGGAATACCAATGACAGAAGCACAGGAGACAGATATTAAGGAATGTTATGATGTTGCTGAGAAGTATCTTACGGATCAATACATTGCGGGCGATACTTTGACCTTGGCCGATATCTCGTGTGTCACCACTATATCATCATTAGACTGTATTGTACCCATACATTCGAAATATAGAAGGCTTCACGATTGGTGGAGCCGACTGAAGAAGGAACCCTGGTACCAGAAGATTAACCAACCAGGATTGGCGCTGTTTGGCAGATTTATCAAAGGCTTTTTGTGATCAATATTGTTTTCAAATAAAAAATAATTTTAAGTGAATAAATGTAATGAATTGTGAATGTAAATGACTGTTATTTGCTTCTGTAAGCGTTT

>gene11126

CTACTACGTTTCTATGATAGATGAGACATTAAATTGAAAAAGTATACATGGTTTAAAAAAATTCGTATTTTTAAATAAATTAAACAGAATGGCGTGTGCTACTCCGTGTTCATTCTTAATGCAGAGTTACTTTCTGTACTCAGCGTTACTGGCGTTACAATTATTAGCATTGGCGCCTTTAGCAGGGATGGTGTGCAATCCAGAAAAAATTCAGCGTGCAAATATAAGTGACTTGAAGAACCTAACACCGTTCTGGTTGGTGGCAGCCCTGTACATGACGACTTCTCCAGAACCGATAACTGCTAGATCACTCCTTCGCGTTTATGTCATAGCTCGAATAGTGGCCGCCATGGGTTATATTTACAAACTACCCAAAATGCTCACGGACTCAGCGTTCTTCGTGTCATTTCTTATAACAGGTTATATGGGAGCAAGTGTAGTATATGCGTATAGGGAAGCGCTGTAGAGACCGCAATCGTTTCACAAAAATGTTTGTGCATTTGAAATTCATATGAACAATGTGTCGTAAG

>gene136

CACAGCCAGACACTTGAACGTCTTGAACTTATTCATCAAGGCCTTGTCAAGTCAAGCAACTTTCTCTTCTTTGCACTTTTAACTTTAATTTTGTGCAAATGTCTGTAAAACTCTATCATTTTCCTATCAGTGGACCTTCCCGTGGAGCTCTACTGGCAGCTAGAGCAGTAGGAGCCCCGGTGGAAGTTGAGATTATTAATTTATTCCAAAAAGAACAGTTAAAAGAAAGTTTTTTGAAAATAAATCCTCAACACTGTATCCCCACGTTAGACGATGATGGTTTCATTTTATGGGAGAGTCGAGCCATTGCAGGCTACTTGGTCGATAAGTATGGGAAGGATGATCACGTGTACCCAAAAGATTTGAAAAAGCGCGCATTGGTCAATCAAAGGCTGTATTTCGATAGTTCTTCTTTGTATGTCAAGATTAGGGCAATTTGTTTCCCAATATTGTTTCTTGGGGAGACAGAGATAAAGAAGCCATTGAAGGATGATCTGAATGTCACTCTGGGTTTCCTAGATCAGTTTCTGAAAGACAGTAAATGGGTTGCTGGTGACCAACCTACTATTGCAGACACATCTATCTATGCTTCTGTGAGCAGTATCCTGGCTGTTGGTTGGGACATATCTGGATTCCCATCCATTGTAAGATGGGTAGGTCAATGTTCTTCATTACCTGGTGCAGCAGAAAATGACGAGGGAGCTAAAGCATTCGGAGATGCTGTGAAAAAGAATCTGCATTAAAAAACATATTTTTTTACAAAACATGTATATTGTTACAAATTTTAATATATTATTTTTATACTTATGTAATA

>gene15408

TGTAAAATGTTATCTTGAATCTTTTAAACAACTAAACAAATTTAAACGTAAATTCAATGCACATAAAGTTCATAATTGATATGATGGACTAAGTTTTTACTCTATCAATATCAAAACAATTTTAATAAAAAAAAATCTGTTATAAAAACATCCGAGCAGCCAGTAACTCAGTACAAAGTTCACAAAAAATTAACTTCCTATATGAAATTTAAAATGTCTTTAAAATTATACTGCGATCTTATGTCCCAACCCTCGAGGGCTCTTTATATCTTATTGAAATCTATCAAATGTAATTTCGAGCCGAAATTCGTCAATTTACGACAAGCTGAACATTACACTGAAGAATACTCTGCAATAAATAGGTTCCAGAAGGTGCCAGTGATAGATCATAATGGATTTATTTTAACAGAGAGTGTTGCCATTCTCAAGTATCTATCCAGAGAGAATGTGATACCAGAAAGTCTGTACCCTAAAGAGTCTAAAGCTCAGGCCAGGGTTGAAGAGTTCCTGGAGTGGCAGCATGCGGGGCTTAGGTTGCATTGTGCTATGTACTTCAGGGTTAAATACTTGAATCCAGTGATGTTTGGCAAACCCGCTGATCCTGTATCTCTAGCTGGGTATGAGAGCCGCATGGAAGCAGCTTTGACGGAGTTCAATGACAAGTGGCTGGGCAGAGGAACAGACTTTATTAATGGAAATACTCCTACCGTGGCAGACTTGCTGGCGGCTTGTGAACTGGAACAACCTAGGATGAGTGGCTATGATCCTTGTGCGAACTTTAAGAATATACAGGTGTGGTGGAAAAAAGTGCGGGAACATTTCAACCCATACTATGACGAGGGACATGTCATAGTCAATAAGATTATTAAGAAAAATACTCCGGTAGCCTCTAAGATTTAATAAAAAGCTCAATTATGTATGTAAGAAATTGATGATTATATTCGTATTTTTTATCTATGAGACTGTTATATTCCAGTAAAGGCTTCGTAAGTCCAAAACTGGTTTTGTAACGGAAAGAAATATGTATCTTATCTTATACTATTATATAAAGCTGAAGAGTTTGTTTGTTTGAACGCGCTAATCTCCGTAGCTACAAGTC

>gene32

AACATATTTTTAGATAGAACAATATTATTTTGAGATACGAATGTGAGACATTATCTGAGCCAGGGGTCTTGTCATGCATAATGTAGGAGCTAACAGGTAACAGAATCCTAAACGTTATACATACCGGTAGAGTGTGACTTTGTAAATTCAGACTAATTTACAAGTGTAAATAGTTATGTACATTATTCCTTTTCGTCATAACCTTAGTGGGATCCTTTCTGGCCAGTGGTTTCTTGGAGTAAAATTATAAACGGCGTTATAAATGTATATCTCTCGAGGCAAATATTTAAACAGTTATCAAGTTTTGTGTCTCTTTAAAAAACGTGCACTCAGCCATACAATGGAGAAAGCAGTGAAGCCGATCCTGTACGGAGATGAAGCTTCACCGCCAGTAAGGTTTGCCATGATGACAGCATCTTTAGCGAACGTTGAGGTTGAATTCCAAAAAATCGACCTTTTCCAAGGAGAGAATCGAACTGAATTCTATAAAAAGATAAACCCCTTTCAGAAGGTGCCAAGTCTTTCTGTAAACGGGCAAAATATATGCGACAGTCATGCCATAGCTGTGTATTTCTGCAGAAAGAGTTACAACAAAGAGCTGTACCCTGATGATATCATTCTTAAAGCCAAAATAGATGAATGGCTTTACTTTGATGCCGGCATACTGTTTCCAATTGATAGTGCTATATTTAGCGACTTTTTTGCCGGTAAATGGCCAGCTAACGAAGTATTGATTAATAAGTGGTATTTCGCTTTGGACCATTGCGAGCTCGTTTTAGAGAAACAGAAGTGGCTTACTGGAGATAAGATACGCCTTTGTGACATTTGTTGCGGGACGACCATAAGTTCTCTTGAAATACTGATTCCTCTCCTGGAACGACACCAACGTTTGAAGGAGTGGATGAGGGAATTAAGAAATCTTCCTTGTTTTGAAATAAATACGCGAGGTTTAAAACGCTTACAAGTCTTTGTAGATGCTGTAAAAACTAGTAATACACAAGTGAATTGATGATGTTGTGCTGGCAGTTGTTTCGTAGAAATAAATGAAT

>gene34

CAGTTATAGTTGATAGACAATTTGATGAAAAACATTTTGCAATATCTATAAAATATAATACAAAAAATGCATACTTAATTAATTCAACACTCACTCTAAATTATAAAGTACCTAATTTTTAGTATATTCACATGGGGCTTTCAGCGAGTAAAAAGATTGCAACTGGCTTAAAGCACTGCACGTTATGGAAAGCAGACAGAAGTCCTGGGTGTCGAGCCGTAATGATGGCGCTAGACGCCATGAACTTGAGCATAACGGAAGTAGACATCAACATAGATAAAGGAGAACATAGAACACCAGAGATGCTTGCCCTAAATCCATTACAAACATTACCAGTACTTAAGGACCGGGAGCTTGTGCTATGCGACAGTCACGCTATTTGTGCGTACTTAGCTGGCCGATACTGTGACTCCGGCCGCCTGTTGCCGAAGGATCCTGGCGGCCGTTCCATAACAGACCAGCACATGCATTATAATAGTGGAGTTTTATATCCTCGATTCCGCGCAGCTGCTTATCCGATCCTATACGAAAATTGCAATTTTCTAATGCCACAACAAATACAAGACATCGAATGTGCTTATTGTGATTTGGAGTGCATGTTGGTTGGTAAAAGCTGGTTTGGTGGTAGTTGGGCAACTCTAAGTGACATTATCTTTGCGTCTACAATCAGCACCCTCAATATTTTGGTGCCTGTTGACAAATACAAATTTCCAAGACTCTCGGGGTGGTTATATCGCGTGTCAGAAGAATTATTTTACGTTACAGCCAATAGGAAAGGACTCTGCGAGTTTTCGAGAAGAATAGATTGCGGCTGTGTTAAAGATGACAAAGAATTCAAGTGTCCAAGAACCTCCATGAGACGACGGACGCTAGCGGCCGATTCATAATAATACCTTGGGGCTTGCCAGAAGTTAAGGGCGGGTGCAGTTTGCCGAAAGTATTCTAATGGGCCTGTATCGCCGTTTCCTGAACTAAAGCAGGTAACAATCTCCTGATAAATGTACCTACTGTTCATTTCGTTTCGTTAATGAGTTAAAATTTAATTGTATTGTATAATTTGAAATGTAAATCGCATTAACTGTTTGTGAATTTATTACAACTTCCAGAAATAATACATTGAAGTTATCAAAAATCTACAATCCATTAGGTAGGTACATGCCCGTTAGACTACTTGCTATGACCTTGGCAAGTATCAAGGAATGTATTTATATTTCATGCTTATGCGAAATAAATGAGTGAT

>gene3508

TTATGTCCTTTAATAGTATTATAGCGTGTCTGAGATCATTAAATCCTAGTCTAGGAACGAGAGATATCGTGTGTAGAGCGCTTATCTGAAATTATATTTTCTTAAAAAACTAAATAATTGTGAAAATTCTCAAAAATGGCGTTAACATTGTACAAAATGGATGCCAGTCCACCAGTACGAGCAGTATACATGGTAATCGAGGCGTTGAAGCTGCATAATGTGAAATATGTTGAAACTGACCTCTTGGCGGATGATCACCTTAAGGATGACTTTCTGAAGATAAATCCACAACACACAATACCACATTTAACCGATGGAAACTTCCAACTATCGGACAGTCATGCGATAATAACATATTTAATGAACAAGTATGGGAAACATTCTGATTTGTATCCAAGTGACCCGCAGCAGAGAGCTATGATTGACCAAAAACTGCACTTCGATAGCGGTATCCTCTACCCAGCTTTGAGGGAGAATGACGAACCTATTTTCTTCGGAACCGCTACATCTTTGAAACCCGAAGGTGCAGCCAAAATCAGATCAGCGTACAATTTCACGGAGAAGTTTTTGACTGGAACACAATGGCTGGCTGGAAACAAACTCACTGTAGCTGACATAAGCTGTGTAGCAACTGTAAGCACACTCAATGAGTTACTACATATTGATGAAGCAATGTACCCCAACATCTGTGCGTGGGTAAAACGTTGTGCAGAACATGAATTTTACAAGAACGGAAACCAGCCAGGGCTTATGGAGTTTCGTCGGCTTCTGAAAATATATTTGGCACGTTAAATTGACACAGAATGAGATGTTGATCTCTAATGTTGAATTATAAAATCTCATTCTGTGTTGACAGAATGAGATTTTATAATTACTATCAACTGTAATTACCATACTTCGTGTAAATAAACGTTATTCTTATGATAATATATATTCAACGTCGCAAATTCAACGTGATTTTAGCAAAGGTGACATTGGGTGTTTATTAGCGTATTACATACTAAAAGGTTTTCTTTTTGGATCACGTTGACTGGATTTT

>gene3509

ACTCTACTCCACAACTTGGTACGATACGCTTGTGTGAGTGACCGCGTTCTCCAACACTTTGATAACACTTGGACCTAAGAGTTCGTCAACAATGGTGCTAACTCTGTACAAGATAGATGCGAGTCCACCAGTAAGGTCTGTGTTCATGGCCATAGAGGCTTTGGACCTCAAGGACGTACAAATGGTTGACGTCAACTTGTTCGAAGGAGAACATTTGAAGGAAGAATACTTGAAGGTGAATCCCCAGCATACAGTGCCGATGCTCGCCGACGATGAGTTTTACATTTGGGACAGCCATGCGATTGCAACTTACTTGGTGACCAAATACGGTAAGACCGATGATCTGTACCCGGCCGACCCACAAAAGAGGGCGATCATTGACCAACGACTCCACTTCGATAGTGGAATACTGTTCCCGTCTTTAAGGGGCACTGTTGAGCCAGTACTTTTTTGGGGAGAGAAATCGTTCCGTCAAGAAAATTTGGATAAGATTAAAAAGGCGTATGAATTTTTGGAGAATTTCTTAACTTCTTCATCCCCATGGCTGGCTGGTGATAAAGTGACACTCGCAGACATTTGCTGTGTTTCTACAGTGAGCTCTATGGATGTGGTTCTGCCTATTGATGCTGATTTGTATCCGAATCTTGTATCCTGGAAGGATCTCTGCTCTCAACAACAGTTTTATGTTGCGGGGAACATCCCTGGCTTGGAACAATTCCAAGAGCTGGTAAAATTTAAGTTGCGTTAAATGCTGGAGACTAGCGCTATT

>gene3510

AGCAATCAAAGTGTTTAAAGTTCTAAAGTCGTTTTTTGTGCAATATTTTAGAGTACATATTACTAATTTGTTTGTTGTCGTAGGGATGATATCTAGATTTTTTTACCAGCCCTGCAGGTCTATAATGGGCATGAAAGTGTACAAGAGAGATACCAGTGGACCTTGCCGGTCGGTGTTTATGGTACTTGAGCTGTTGGGCATAAAAGATGTTGAATACATACAAATGAATTTGATTCAACGCGAGCACTTCAGTGAGGAATACTTGAAGATGAACCCACAACATACAATTCCAACGTTGAAAGATGGCGATTTCGTAATTTGGGACAGCCACGCAATTGTGACGTATTTAGTTAACCAATACGCAAAAGACGATTCCCTCTATCCCAAGGACCCCAAAAAACGCGCCATTGTGGACCAAAGGTTACATTTTGATAATGGTGTTCTCTTTGCAGCATTGAAAACTACTATGGTTCCAATTTTATACAACGGTGAGACAGCATTCAGGCAAGAAAATCTAGACAAAATAAAAGAGGGTTATGCGTTTATTGAGAAATTCTTCTCTGGACCCTGGCTTGCAGGGGAGTCAATAACACTGGCTGACATTTGCTGTGTGTCTAACATCAGCTCTCTGAATGAAATTTTCCCCATAGATAAGGCCCTGTACCCCAAATTATCGGCCTGGTTTGAGAGATGTTCAAAACAAGACTTCTACATCAAGAAGAACTTGCCTGGCCTGCAAGAATTTCAAGAACTATTAAAAGTGAAAATTGTATCCTAAAATTGATCATTCATTCATGAAGACGTGTTTTGTTAAGATATTGTTCTGTGAAAATAAATATCTGTTCTTTTAC

>gene3734

GTCAGTATCGAGTGAGGCGTACAGTCGTGAGTTGTGTCTTTTAAATTGTAATACTTGACAGAAAAATCTCCATAACAAATATGGGTGTGAAACTGTACACTTTGGATATGAGCCCGCCAGTGCGTGCCTGTATGATGGCTTGTGAGATCTTCAATGTACCATTCGAGAAGATCCCTGTTGATTTAATGGCAGGTGACCATCTAACTCCTGAATATTTACAGAAAAATCCTCTCCACACAGTCCCTGTTATGGAAGATGGTGATGTCACGTTACATGACAGCCATGCAATAATGGCGTACTTAGCTGACACCTATGGAAAAGATGAATCCTGGTATCCAAAAGATGTCAAGAAACGGGCTTTTGTGAACCAAAAATTATTCTTCGATACAGCTATAATTTTTCCTAGACTACGAAACATCACATACTTCATCGTGTTGAAAGGAAAGACGACTATAGAACCAGAATTGCTGGAGGCCGTTGCAGAAGCTTACGACTTCATAGAAGCATTCCTGTCCCGTACAAAATACATCGCTGCAGATCATGTCACCATCGCTGATATTGGAATATTGGCAGTTATGACGTCTCTCGAACACATTCTACCAGTGGAACCTAATAAATACCCAAAAACATCCGCCTGGTTAGAAAACTTGAAAACAGCCCCATACTGCAAGAAATGGAATGAAGAAGGTTCCAATGCCTTGGGTGCGTTTGTTAAGAGCAAAGTTACTTTGTAACATCCTTCGAATAATAATATGTATTGACCAATAATCTTAATAATATAATTATGACTAAGTGTTAATAAAGAATTTCC

>gene3735

ATCGACAGTGACGTACGGTAGACTACTAATTTACATATTTACGTAAAAAATAATTGCAAACATGGTTGTGAAACTATACACCTACAACATGAGCCCGCCGGTGCGTGCATGCATGATGGCTTGTGAGATCTTCAATGTACCATTCGAGAAAATACCAATTGACATTCAGGCTGGTGAACATCTTACACCAGAATTTTTAAAGAAAAATCCCATCCACCAAGTCCCTGTTATGGAAGATGGAGATTTAACCTTACATGACAGCCACGCCATACTGCCGTACCTCGCTGACACCTACGGAAAGGATGACTCCTGGTATCCCAAAGACGTTAAAAAACGTGCGCTTGTTAACCAAAAACTTATCTTCAACGCAGTTATTATTTTCGCTAGAGCTCGTAACATTACAGGCCAAGTAGTGTTGACAGGAAAGAGGACTATTGAACCAGAATTGATAGCAGCTGTTACAGAAGGCTACGAGTTCATGGAAGCATTCCTGTCCCGGACAAAATATATCGCTGGAGACCATGTCACAATCGCTGATGTAGCTATTTTGGCTGTTATGACGTCTCTCACACAAATTATAAAATTAGACGCTCAAAAATACCCAAAGACATTGGCCTGGTTAGAAAACCTGAAAACAGCGCCATATTGCAAGAAATATGATGAAGCAGGTGCAAATGAATTAGCAGCGTATGTTAATAGCAGAGTTGCATAAATTCCTGTATTTCTTATTATTCTATAAATCAGTGTCTTTTTAAGTTTTATAAGATAAGATCAAATATAGCAAGTGT

>gene3736

ACCAAATCGACTGAGACGTACGGTAGACTACTAATTTACATATTTACGTAAAAATAATTGCAAACATGGTTGTAAAACTATACACCTACGACATGAGCCCGCCGGTGCGTGCATGCATGATGGCTTGTGAGATCTTCAATGTACCATTCGAGAAGATACCAGTCGACCTTCAGGCTGGTGAACATCTTACACCAGAATTTTTAAAGAAAAATCCCATCCACCAAGTCCCTGTTATGGAAGATGGAGATTTAACATTACATGACAGTCACGCAATACTGCCGTATCTCGCTGACACCTACGGAAAAGACGACTCTTGGTATCCGAAAGACGTTAAGAAACGGGCGCTTGTTAACCAGAAACTAATCTTCGACGCAGTTATTATTTGCGCTAGAGCACGAGATGTTACTGGACAAGTAGTGATGACAGGAAAGAGGACTATTGAACCAAAATTGATAGCAGCTGTTACAGAAGGCTACGAGTTCATGGAAGCATTCTTGTCCCGGACAAAATATATCGCTGGAGACCATGTCACAATCGCTGATGTAGCTATCTTGGCTGTTTTGACGTCTCTCACACAAATTATAAAATTAGACGCTCAAAAATACCCAAAGACATCAGCCTGGTTAGAAAATCTGAAAATAGCGCCATATTGTAAGAAATATGATGAAGCAGGTGCAAATGAATTAGCAGCGTATGTTAATAGCAGAGTTGCATAAATTCCTGTATTTCTTATTATTCGTTAAATCATTGTCGTTT

>gene3737

ATGGTAATAAAATTATATACAACAGAACTAAGTCCGTCTGTACGGGCTTCTATAATGGCGTTTGAGATATTTCAAGTTCCATATGAAAATATAGAAGTTAATCTATCCGATAAGTCAATGTTGGATCCTGAATTTTTAAATAAAAATCCAATGCGAACAGTTCCTGTTCTTGAAGATGAAGACTTTATATTGCATGATGGTCATGCGATATTAATGTATTTAGCTGATGCATACGGCACTGAAGATTCATGGTACCCAAAAGATTATAAGACGAGAGCGCTAGTCAACCAAAAACTATCATTTATCAATGAAATTATATTTTCTGGATTCAAAAAGATTGCGTACTCCGTAGTAGTTGAAAGAAGAAAAACGTTGATGCCACAATGGATAGAAACCATTGAGGAAGGTTATAGTATAATGGAAAAGTTTCTAAGCAAAACAACCTACATTGCCACAGATGATGTTACAATCGCTGATCTCTCAGCTTATAGTAATATGTCGTGTCTAATGTATGTTATTCCTGTGGATAGACAGAAGTACCCGAAGACACTTAAATGGTGCCACGTCATGGAGATGCAGCCCTACTGTAAAGAGTTCAACAACAAATCGGCGGCTACTTTCGGAGATCTGTATAAGCTAATTATGTCACATTAG

>gene558

TAATCTTACACTAAGTCGATTGTGTAACAATTATCACCATGTCAATACACAACACTGCGATAATAGTTACATATAAGGCCATGGCCATTTATGTTCAGCCAGTACTTCGACCACATAGCAACAGTCACTATGGTCATCAGGATATACAAGAAAGACGCTAGTCCACCAGCAAGGGCTGTCCTGATGGCTGCAGAGATCATCAACTTGAAGTGCGAAATCCAAGATGTTGACTTATCGTCAGGACCACACCTTTCTCCAGAATATCTTGAGAAGAACACTCTACGTTCAGTACCATTTTTAGAAGATGGTGACTTCTACCTGGCTGACAGTCATGCCATCATCACATATTTAGTATCGAAGTATGGTGCTGAACATCGATCGAAGTGGTACCCTTGTGATTTGAGTGTAAGAGCGACTGTGGACCACCGAATGTATTTTGATACTTCTGATTTATTTCCTCATATCAAGAATATTGTGAGTGTGATTGTGACGCAAGGGGTAGGTCCTAATGCTGAACAGATAAAGGCAATTAATGCTGCTTATGAAGTTTTGGACAAATATTTGCAGAAGACGAAGTTTGTGGCAGCCAATCATATGACTCTAGCTGATGTGTCGTGTGTGGCGAGTGTGTCTTCTCTAAACGTACTATGTCCTGTCGATAGCAAGTATGTTCATCTCCTAAAATGGTGGGATACGTTGAAAGAGGAAGAGTGGTACAAAAAGGCAAATGAACCCGGATTGGCTAAGTTAGAAGCGTATGTTAAGTCGAAATGGAATAAGTAAAAGTGATTATTTCATTGAATATTTCGTTTTTTAATAAACTTTTTAAGTAATGTAAA

>gene559

CGCTGGGCGCATCCTTCAGTATCGTACTACGGTTCACTATCACACAGTCAACATGCCTCTCAAAATCTACAAGTTGGACGCGAGTCCCCCAGCTCGGGCGGTGATGATGTTAGCCGAGTTCCTGAAGCTCAAGCACGAAGCTGTGGACGTCAACCTGATGACTGGAGATCACCTCACACCAGAGTACTTAAAGAAGAATCCACAACACACTGTACCACTGCTGGAAGATGGTGACTTCTATGTAGCTGACAGTCACGCCATTAACACATACTTAGCCTCAAAATATGGCGGTGCACAGTCAGCGCAGCTATACCCTACAGACCTCCAAGTGAGAGCGACCATCGACTCAAGGCTGTACTTCGACATCTCAGCCATCGCAGGAAACTCGGGTGCTATAGTTAGTGCACTTCTTCGTGGAGATATCACATCCCCCACTAAAGAACAAACCGACAAGCTGAACTCCGCCTATGAAATTTTGGACAAATTCTTACAGAAGACCAAATTCGTGGCAGCAGATCATCTGACAGTGGCTGACATTTCACTGGCAGCTAGTGTATCATCAGCATCCCTTCTGGTTCCTATTGACGAGAAGTACAGTAAATTGACGGCCTGGTTCAACACAATCCAACAGGAGGACTGGTACAAGAAGGCTAACGTACCAGGACTAGAAGGATACAAAGCATTCGTGCAGTCCAAACTGAAGTAGATTGTAATTGTAAGTTCCTATCTATCTGTTTATGTTATTATTTGAACTGAATAAATTATTATTTCAATGTAAAAAAA

>gene6249

TGAGATTACATGTATTATGTATTGTATAGTGTTCTTTTGGATTTTTGTGAAATCACCCGATTGCTGCATTAGTTACTTAGTTCGATAACTTCGATCGAAGTGAACGATTTTTTTCAGTAAACAAAATGGCGCCACTGTTGTACAAAAGCAACACGAGTCCTCCAGCGAATGCAGTGCGGATGTTGGCTGATATCATTGGTCTGGAGCTTGACCTCAAAGATGTCAGCATACCTAACATGGAACATAAGTCGCCTGAGCATTTGAAGCGCAATCCTATGGGAACTATCCCGACATTGATAGATGGCGACTTCATCATCTCTGAAAGCCACGCAACCATGAAGTACCTGCTATCAGTGTATGGTGACTATGAACTCCGCGAGTCATTATACCCGAGCGACGTGCGCACGCGCGCCCTCGTCGACCAGTGCATGTTCTTCAACGCTGGCGTCTTCTTCCTGAAGCTGCTGGGCTGTGTTTTACCAGCTGTATTCGGAGACTTGGACGGTCCTACACAGCAGCATAAAGCAGAAATTGATGCGGCATATAGCGTGTTGGAGGCATATTTGAAAGATCATAAGTACATTGCAGCCGACCACTTAACTCTCGCGGACCTGAGCGTTGGTGCAACTGCCATATCCATTCAGGTTGCACATAAGCTGGATGCTACGAAGTTTCCCCTGACGGCTGCGTGGATGTCCAGTTTAGAAGACCACCCATCATTCAAGAAGATCTTACTACCAGGTGTGCAACTTTTAAATGAATTCGTCAATGCGGCCTGGCAGAAGAATAGAAAATAATTAATATTATGAATATTTGAATTAATTAATTGATTGTATTTTTTG

>gene6250

ACGATTATATATCCACTACATAGACATGTATAAGTTATATAATAGTATGTTGTACTCGGCAGTTGGTAGCAGACCTTGCTTCTAAACACACGATTACAAAATGGCGCCGATACTGTACAAAATAGATGGCAGTCCACCCGCCAACGCGGTGAGGATACTCTCAGACATCATCGGGCTGGAACTCGAAGTCAGGGACGTCAACTTTGGTGTCCTCGAACACAAGTCTCCTGAACATTTGAAGCGCAACCCTATGGGCACAGTACCAACGCTGATCGATGGAGACTTCACCATCTCTGAGAGTCACGCCACCATGAAGTACCTTCTCTCAGTGTACGGCGGCGACAAGAGCGAGTCGCTGTACCCGAGCGACGTGCGCACGCGCGCGATAGTGGACCAGTGCATCTTCTTCAATGTCGGCATATTCTTCATTAGGCTCAAAGTTGTTGTTTTACCTGCCATATTCGGTGACTTGGACGGTCCTACAGAACAACACAAGGCAGACATTGACGAAGCCTACGGCATCGTGGAGGCGTACCTGTCTAAGAACAAGTACATCGCTGCTGACCACCTTACTATTGCAGATTTGAGTGTTGGTGCCACCGCCATCTCTATGCAACCTTTGCATAAACTGGACGCTGCCAAGTTCCCCCTGACGGCTGCGTGGATGGACAAATTAAAAGAGCACCCTTCAGTTCAGAAGTACCTGGTACCCGGCGCCAAGGGCCTCGGAGAAATTGTACACGCGGCCTGGGAGAGGAACAAGAAGAAGTAGATTGTATAATAAAATTTAAATAATTA

>gene6251

ATTTATCATTATGTAATTTGTTGATAAAAATTGTCACGCTATTTATTTCATATTGATTACCGATTGTGGTTGTGTTTTTGCAATATGGCGCCGATCCTGTACAAGTTGGCAGCGAGTCCGCCCGCGTGCGCCGTGCGCATGGTGGCTCACATCATTGGACTCAAACTGGACTACAAGGAACCAGATATTACTAAGATGGAACACAAGTCCCCCGAGTACTTGAAGTTGAACCCGTTGGGGACGATACCGGTGTTGATAGACGATGACTTCATCCTGTCTGACAGCCACGCAATAATGATCTACCTGCTATCAAAGTACGGCGGTGAGCACGGCGAGCGACTGTACCCGAGCGACATTCGCACGCGAGCTGTCGTCAACCAGGTCATGTTCTTCGACACCGGGATACTCTTCGTCAGGATCAAAGTTATTGCTCTGCCAACTATAATGGAAGGTATGAAAGCACCTACGCAGAAGCACCTGAATGACTTAGAGGAGGCCTACGGCATGGTGGAGGCCTACGCGTCGAGGTACAAGTACATCGCGGCCGACCACCTCACCATCGCCGACCTGTCGCTGGCCATGACCATGGGAGCTGCACAGGTTCTGCACAAACTGAACCATAAGAA

>gene6252

GTTGTAGTAATTCGTTGTACTTATTACGTAAGTAAATATCAAAAGTAGTTTTGTTTTCGCAGTTTTAAAACCGCGTGTGCAATGCGGCAATGCGCAACCGGTATCGTGCGTAACAAAGATCAAGGTCACCACGTGCTTTTTCTTTTTCATTTTTAGGTTATAATCATGCTGTCATGTTTATCTGTTTTATCAGAAACATTATTTAATCGTTTCATTGTGTTTTTTATCGTGAAAGTGAACAGTGGAGTGTCGTGGAACTGTGCTATTAATTGTTAAAGTTGAAGCTGAATTAGAGCCATGTCGGTGTCTCGTCTGCTCCTCCACCGGGCTCCCGGCAGCCCTCCAGCGCGGGCCGTGATGATGCTCAGCGACATGCTAGGTCTGCAAATGGAATATAACGACGTAAAATTCTTGCAACTAGAACACAAGTCCCCTGAGTTCAAAAAGTTAAATCCCATGGGTACAGTGCCGGTTCTCCAAGATGGTGACTTCATTGTTTCTGAAAGCCACGCCATAATGAAGTACCTACTAACTAAGTACGGCGGCGACAAGCGTGAGCTGCTGTACCCGAGCGACGTGCGCACGCGCGCTGTAGTGGACCAGTGCATGTTCTTCAACGCTGGAGTCTTCTTCGCTGCTTTTATGTCAGTCGGGCGAGCAACGTTCACGGGCAGTATATACAAGCCAACAGCCCAACACATACAGGAAATAGAGACCTCGTACTCCGTGGTAGACGCCTACCTGCAGGACAGGCCCTACGTGGCCACCGACCGACTGACCTTAGCTGACCTCGCCGTCGGAGCAACGGCCTCGACCGCTCAGGTCTTCATTAAAATGGATGCTGATAAATTCCCCCGCTGCGCTGACTGGATGTCTCGCCTACATGAGGAGCAAGTATTCAAAACTGTCATGGCGCCCGGCGTCGCTTTCTTTGCGAAAGTCATCAACAAAATCTGGGAGCACAATAAGTCGAAACTAGAAAAGAAATAAATCAATATTTATTGTTTATGAAA

>gene7753

TATAAATTTACCTGTTTGCGATAAAAATATCAGTGCTTTAGCAACATTCGTTAGGCGTTCTAGAGAATTAGTAGAAAGTGTACTCTATTCTATTACATCTATTGAATTAATCATGCCGAAATACGTATTCCACTACTTTCCAATAAAGGCTCTGGGCGAGTCGGTTCGTCTGCTGCTGGCATATGGTGGCGATGGGTTCGAAGACCATCGGATAGATTTGGACGACTGGCCGAAGTTTAAACCAAATACTCCCTTTGGACAGATGCCAGTCATAGAGTTTGACGGCAAGCAGTACGCTCAGAGTATCGCCATCGCTCGGTACTTGGGTAACAAGTACGGTCTGGCTGGAGATACCCTGGAAGATAATCTAGAGATTGACCAGAACGTCTACCTCATCAATGATCTTCGTATAAAGGCGGCATCAGCACATTACGAAAAGGACGATGTCATTAAGGAGCAGAAATATAAAGAGTTCTCTAAAGGAGTTTTCCCAGACTCCCTAGAAATGCTAAACGCTCTCTTTGCTAAGAACAACGGTCACGTCGCTTTAGGAAAGTTGACATGGGCTGACTTCATGTTTGCGGGTCTATTTGATTACTTGAGTGCTATGATGCGTATGCCTGATCTTGGACAGAAGTACCCCGCCTTACAGCAGGTTAAGGACAGAGTATACTCCTTTCCCAAGGTCAAGGCTTATGCTGATGCAGTTCCTGCTTAGAATATAGATCATAATTTACCTGTGTTAAAGTAATAGTAGGTAGGTAAGCATAGTTTAAGATAAAACACCTCCAATTTTTACACTTAGTTTAAACTAAATTGTAAGTAAATAAATGTTTAATTGATGCA

>gene7755

TGAGATTACCGATTCGTCACGTATTCCTTTCAAAAATCGAAAATCTACATACCTACTCGGCCCAGCTCGGCGGCTAACTTCTGACTACTTCTACCGGCCGGCAACCGTCGGCGGGTTAGTATTGGAGAGAGCGCCTTCCCGACGACACTGCTTCCGTGCTCCCAAAAATAACTAACCATGCCTGAGGTCGTGTTCTACTACTTCCCCGTGAAGGCTCTGGGCGAGTCCGTCCGCTTGCTGCTGGCTTACGGTGGCCAGGAGTTCGATGACCGTCGTATCCCACAGACAGAATGGCCTGCATTCAAGCCAAAGACCCCATTTGGTCAGATGCCAGTTCTGGTGATTGACGGCAAGGAGTACGCTCAAAGCTTGGCCATCAGCCGGTACCTCGGTAACAAGTACGGAGTCGCTGGAGACTCCTTCGAGGATGCTCTGGAGATCGACCAGAACGTGGACCTCATTAATGATCTGCGTGCAAAGGCTGCAGTAGTACAGTATGAACCTGATGAGGCTGTGAAGGAAGCTAAATACGCTGACTTCGTTAAGAACGTATTCCCAGACCTTTTAGAGAAATTGAACGCTATTTTTGTCAAGAACAACGGCCATGTTGCTTTAGGAAAGTTGACCTGGGGAGATTTCGTGTTCGCGGGCATGTTTGACTACCTGAAGGTGATGCTGCGTATGCCTGACTTGGAGAAGAAGTACCCAGTGTTCCAGCAGGTGATAGACAACGTGTACTCCATTCCTAAAGTCAAGGCGTATGCAGACGCCGCGCCCCCCACCGACTTCTAAATTTAAATTCCTGAAACAACACGTACAAAATATAGAGAGTCTATTTTGAAACTATACGT

>gene9126

ATTAAGTTATCAGCTGTTGTAAAACAGATAAACAAAATGACAACGACCTCCCTCAAATATTTTATGCAAACAAAACTGATAACTGTTGAAGTGAAGTGTATTTTTGTGAATTAAAAAACCTTAACATTATTTACGAGTTTTAATTTAAGAATTTGACAATATTTTATAAGACAAATAAAGCAAAAATGCCGCCAATCCTCTACAAAGCAGATGCCAGTCCCCCAGCGAGAGCAGTGATGATGGTCATAGATATTTTAAGGATAAACGTTGAACAGAGGAATTTGAATCCAGTATTGCGAGAACAAGATGCACCAGAGTTTGTAAAGAAAAATCCCATGAGAACCATCCCTTGGTTAGACGAAGGCGACTTCTGGTTGGCTGACAGCCACGCCATTATGATTTATTTAATAGAAAAATATGGTAAACCAGAACATTCCAAGTTGTACCCGACTGACAGCAGAAAGCGCGCCACTGTTCATCAGAGGCTGTTCTTCGATTGTGGGATCCTGTTCCAAAGACTAAGAGCTGTGATGGCACCAACATACATGGGAAGACTGACAGAGATGTCAAAAGGCATGATCAGGAACATTGTGGACGCATACGCAATGCTTGAAGATTATTTATCAGAGAACTTGTATGTGGCCGACGAAGTCATTACGATTGCGGATTTAAGCATAGCCTCTACAATGGCAACACTGGTTGGATTAGTTGCAATTGATGAACAAAGATTCCCCAAATTAACACAATGGTACAACAATATGAATAAACAGGATTATTGTATAAGAATCAACATTCCCGGCGGCAAAGAACATTCCGATGGACTGAAGGCTTTGATGGCAAATACTAAGTTTCACCAAAAATCTAAGATATAATCTTATGATAAAGTATTATAATGGTAAAGGTTTGTTAAAGGAGATTTTTACTGAGATTTTGAAAAATCAAAACAGCGAAACTGACATAAGATAAAAT

>gene923

AGTCAGTGTACTCGGTTTGACAACAACAACACCTACTATTGGTTGACGAAATACTGTAGCTATACGATAACAAGTAACGAAGCGCTGTGTTACAGTGAACAAGCGACTCGGTCAGTGTGACTGTTATATCCCAGCGCGCCGCTCCACTCGGTCGGCTACGAATTTAATAATCGGAAACGTAGCAACTGTGTTTTATTGAAAAATGGCACCGCTACCATTGGATGATCCAGCGGTACAATCGTATCTGCTGTATTCCAGCGTCTTGGGCCTCAAAGTTCTGGGTATGGCATTTTTGACAGCCAGAGTGCGATACGCTAAGAATGTGTTTGCTAACCCAGAGGACGCTGCAGCGAAGAAAGGAAAAGTGAAGTACGATGACCCCGACGTGGAGCGAGTTCGTCGCGCGCACCTCAACGACTTGGAGAACATTCCCGTGTTCTGGGTGCTGGGCGCGCTGTACCTGACTACTGCGCCCTCCGCCTGGCTCGCCACCACGCTGTTCCGTGTGTACACAGCTGGCCGAGTGATGCACACTCTGGTCTACGCTGTCAAGCCACTGCCTCAGCCAGCGCGTGGCATCGCCTTCGGCATTCCTCTCATGATCACATTCTACATGGGAGTCAAAGTAATTCTACACTACTCCAGTGCGCTGTGATTTGTTAATAAGAATGTATTGGACTGTATTAGACGCGTGATCAATAAAAAAGTAATGTGTACTCATAACATTATCAAACTCGGAGTTGTAAACGAATGTATGTTAGATAAGATGTGTAATCTTATCTAATCCTTTACTGTCGCTGTACGCAACTGTAAAGTGACAAGTACATAGGTATTACTGTTGTATTTTTTTTACAAGTGCTTAATAAAGAAATGTTTGCCCTATA

>gene9807

GCTTCTCAGTATCACTCAGGATTCCAACAAGTATATAAAATTGGATAAGCGAAATTCGATACCATAGTGAGATAGGTCGTGTAGGCTAGTGTTTCTAACCGCTAGCAATATACCGGTATAGGTAGGTATACATTGAATACAACAAGTGAAAGAGTGTCTCGTATCTCGACCCTCGCACAAACATTCAATTCATTTCTGTGTCAAACTCCGCACACGATCCACGGTACTGATAGAACTGCACAATGGTCCTAAAACTGTACTACTTGGAAGATGGGCCGCCTTCCCTCTCATGCAGGCAAACCTTGGAGGCGCTGGGGGTACCCTTTGAGATCGTCAACGTTAGCTTTTACCATGGCGAGCATATGACTGAGGAATTTGCAAAGAAAAACCCACAAAAGGAACTGCCAGTACTGGAAGACGATGGTTTCTTTTTAAGCGAGAGTGTCGCCATGATGCAGTACATATGCGACAAATTCAAGCCGAACAGCTCGTTGTACCCGACAGACTCAAAAGCAAGGGCTATAATAAACCATCGGCTGATGTTCACCATGACTACATACTACGCTGAGCTGTTAAATTATTTTATTATGCCGGTTTATTTCAAATACGAGCGTACTCCAGAGGCGTTGAAGCGTGTGCACCGTGCTCTAGACTTGTTTGAAACGTACCTGGAGCGCGAGAACACCAGCTATTCTGCTGCCAACCACCTCACCATCGCTGACTTCCCTCTCATCAACGCCACTATGGCTATGGAAGTCACTGACTTCGACTTTTCTAAATACAAAAGGATAACAAAATGGTACAATGACTTTAAGAAGAACCAGCCCAAACTATGGAGCATCACAGCCGAGGGTATGAGATTGATGAGGAAATGTGTAGAAAACCCACCAGACCTATCACACTTAAACCACCCTATACATCCTGCGAGGAAAGCCGAATAGAACAGCTAAACGAAAACTATTCTGAACCATTTTCTCTAATACTTTTGTAATTTTCGAAGAGAGGGCGCTGAATTCAATTTTTGTAGGAATAAGAATGAAGTGCATATATTTTATCAAATGTTGAAAAGAATACTGCTTTACTTTTTTTCTTTTCGAAACATCTACATGTCTACTTAATACATTTTGTAAATTATAA

>gene9953

ACAGAGTTATAAATCAGTATTTCTGTGGACTTCAGTTGAAACAGTGACTTTCGTTCGTCGTCCGTAGAAAGCTTAATCGGAACTACGCGAGTTGACTTAACTTTGTTCGGTCATCTGTAGGTTATTTCTGAAATAAAATTTGGTGATTTAATTTTGAATTAAATTTGGTTTGTGCTAGTTTGATTTAAAATTATTCAAACTGGGCCAATGCATGGACAAAACTGTTGTAGAAAAAATACAAAAGGCAAAGTTTAATAGCAATTCGCTAGACGGCAACAACTAAGTTTTTATATAAAACTATTTGTTGAAATAATTAATAAAATCATGAAAAGCTTAGCATGTTTAATTGCTGTATGTCTGGTAATAAACCTTCCTAGCAACGCAGCAGCACGTTCTAAGAGCAAGATGCCAAACCAGCCAATCAAGGTGTACTACCTTCCACCTTCTCCACCATGCCGGTCCATCATCATGGCTGCCAAGGTGATTGGTGTCGACCTGGATCTGGTCTTAACTAATATCATGGAAGGACATCATATGACGCCGGAATATCTTAAGATGAATCCACAACACACGATTCCGACCATGGACGACAGTGGATTCATTTTGTGGGAGAGCCGCGCAATACTGGCTTACCTCGCCAATGCCTATAGTCGCGACGACACTTTATACCCGAAGAACCCTCGTCAGCGAGCTATTGTCGACCAGCGCCTGAACTTCGACCTTGGTACCCTTTACGTACGATATTCTGCTCTCTATTTACCCATGCTGTTTCGTGGTGAAGAGTATGATGAACAGAAGGCTGACCAGTTAGACGAGGCCCTTGGCTGGCTGAACACCTTCCTCGATGGACGGGCCTTCGTCGCTGGTGACAATCTTACCATCGCTGACATTTCTATCATCGTTACCATCACAAATCTTGAGGCTTTTGGCTACGACATGAGCGGCCATCCAAACCTCATGAAATGGTTCGAAAGGACCAAGAAAGCTCTTGAGCCATATGGATACGAAGACGTCGATGTAGCTGGAGCTAAGATGCTTGCAAATTTCTTAAAGAAAGATTAGGAGATTCATTATTTATGTATTTTTTTATTTGTTTATCTGTTATAAATGTTTAGATTTCTTTTTTTACGGATACACAGGTTTTGAATGTTTATTATTTATTAATTGAACATGTTTTATGTAATTTGTTTATTATGTCCTTGTAACAAATAGATCTGTTTCAATTATA
