# Supplementary material for: Identification of transcriptome and fluralaner responsive genes in the common cutworm Spodoptera litura Fabricius, based on RNA-seq
Source: BMC Genomics. 2020 Feb 3;21:120. doi: 10.1186/s12864-020-6533-0 (PMC6998375; doi:10.1186/s12864-020-6533-0)
Supplement: Supplementary file 7 — Additional file 7. Carboxylesterase nucleotide sequence of the S. litura transcriptome. [file 12864_2020_6533_MOESM7_ESM.docx]

**Additional file 7**: Carboxylesterase nucleotide sequence of the *S. litura* transcriptome

>*Spodoptera_litura*_newGene_925

GTCCATTGTCAGTATAATATCGTTGGCATTAGTTCAGGTATGGTGAAGTTGTATACCTCATATGAAAGCTTAGGCAATGTAGATGTTTTTAGTAAAAAGTCCACAATAAGTGTCATAGCAAATTTTTATGTTAGAGAACCATAATAGGTAAACTTGAAAAATAAATCTTATGTTATATGAATTTGAGATAAATGTGTCTAAAATAATTTTATTATTGTGCATTTTTTTATTACCCTCTAAAAGTTAAATATTTTAATAACTGTACATTTTCCGCCTAGCCCTTTGTTTGACAATAATATTGGAAATGATCTGTTCTATAACCCCATACGCAATTATGATCTAAAAACAACGCAACTGTATTTTTATTTTTTTAAGGGGAGAAAATCATCGAATTACTGCTCCCGCCTGGGTGAGGCGGGACTGCGGGAGGGAGTGTCAGACTCTTTCTGACTAAAAACCACCCCGTTCCTTCTCCTGCACGTCGAGTCGGAGCCCCGGTTACCCGTTAGGTTCTCCGTGACTGTATAGGAAAATATGTTGCGTAACAAATCGCGCGTCTAGTGTAATCTAGTGGATATGAGCTCTTTACATAATAACAGTCGTAATACATATATAGATGCAGTAGAGTTATCTGAGAGCGTCTTAAGTCACAATCATTTATCGCAGTCAAAATAAATTTTATCAAAGAACACTTGCACTTATCTAATTACTAAGGCTTATCGAAAGACGAATTTTTATCTACTAGTGTTGTACTGAAAATAATTTACGCTGTTATCAAATATTCGATTGTATATATTTATCTAGGACACGGTGGATACAATGGCGGTGTGCCCTGCTTGGGCGAACACCGCCGTGTCCTCGAGGATGTGGCCGACGATGGCGACTTCTCGCGTCCAGCTCTGGTTCAGCCCATGGTATGGAGCGAAGGGGACTGGGATGCCGTCTCCTCCTTCTGCGAAACAGTCATGCTAGCTAAGGAGGAGGCGAGGCGCCTAATTCCTCACACCTCAGCCGTCGCGAGAGACTCCGGGTGTCGCGGATCATGAGATGATGTCTGGCAACGTAATCACGGGCCCACGGACGGCAACCACAAATAACTCACCGCCTGAGCAGAATCAGACCTGAGCATATGGCGCGTCACATTCAGCGCGCCTCGAAGAGCCATCAAACTACTGCAGATGAGGCTCAGTGGGGCTGATGCCGTATACGGAGCTGCGGAAGACTTAACGGGTTACTGGGGCCACTGTTAGAAGTGCAGGAGTAGAAACGGGGTGGTTTAAGTCAGTAAAAGTCGGACACTCCCTCTCGTCGCATCCGGGCGGGAGAAGTCATTGGATGGAAAATCATCCTATAAGAAAAACAATATTAGCTCCAATTTTCTATATCAGATTTGGAGTACGAGTCATTATTTTCATTGGAAGGTTTTTTGTCTGTGGTGACAGACAAACGGATTTTTGTTAGGTTTTTTTTCAAACTCTTATATTTTAACAGTAATTGTAATAATATTTTTTTAATAAATTGCCTATTAACATATTCATTTGTAAGTGATAAAACGTTAAACATTTGTATCTCCATGGTTTATCGGAACAAGGACGAATCAAGTCATTTACAAATTCTAACTTTAGAAAAACCTGCCTGAACAACATTTAATCATTCAATTTATCAATGCACTGATTTTTTTCAAAATAATCCCACTTTCTTTAATTAATTTAAATTAAAGTCGAATACAAACACATACCGTTTTCGATACGTGGTCACATCACTAAAACTGAAAATATATGCGACGGTATCAAAATATCTGTAGCGTATTTTATTAGTGTACTAGCAACCTCACGAATCTAACGTCGAATGTTTTTCAGAAGATAAGAATTTTACATTATATAAAATAGTAGCAAAACAAACATGTGGAAGATACTAATATTGCTAAGTGTTGTGACGTTAGGTTATTGTGACGATGGTGAGTGGAAACAAGTGCGCACTGCACAAGGAGAGGTGCGCGGACGCAAGGACCCTGCCGGTGGACTCTACGCATTCCTTAATATTCCATATGCTAAAGTGCCTGTTGGAACTGACAGATTTAAGGCACCTCTTCCTGGACCGATATGGCTGGAACCACTAGATGCCATCGACAAAGGCATCATATGTATGCAACCGCCTTCACCATTCTTTGACACTAGTTCCAAAGTAATGCAGGAAGATTGTCTTATCACTAATGTTTATGTTCCTGATACTGATGATAAAAACCTACCTGTTGTAGTATACATTCATGGAGGTGCATTCGAAATAGGATTTGGAAACATGATGACACCCAAACACTTAGTCAGAAACAAGAAGGTTATTGTAGTCAATTTCAATTATCGTCTTGGAGTACACGGATTCTTATGTCTGGGCACTGAAGACGCGCCAGGAAATGCAGGACTGAAGGACCAAGTGGCTCTACTTCGCTGGGTGAACAAGAATATTGCAAGTTTTGGTGGTAACCCAGACGATGTTACTATAGCTGGCTACAGTGCAGGCTCAGTCTCAGTCGACTTGTTGATGCTTTCGAAATCGGCAAAAGGACTGTTCAATAAAGTCATAGGTACTGGAGAGTGGAGTAAGCGTGGGTTTTATAGCCATTCAAAGAAATCCCATAGAAAACGCTAAAAGCTACGCCAAAATGTTAAACTTTACTAAAGTTAATGACTTTAATTTAATGCAAGAATTTTACAAAAGTGCTCCAATGAAATCAATGTTCGTGAGCACATTTTTAGGTAGAAAAGATTCAACTGTGCTATTTTCACCTTGCATAGAAAGGAGAAAAGGTAATCAGGCCTTTTTAGATGAATCACCCTATAATATTATCAAGAGTGGCAAATACAACAAAATCCCTATGCTAATCGGCATCAGTAACATGGAAGGATCTTTGCAGATGCACCATGTGGGAATATGGAAAGACTCTATGAATACCAAATTCAGTGATTTCTTGCCAAATGATATAAAATTTCAGAGCAATGACGAAAGAGAAATGGTGAGCAATAAAATTAAGGAATTTTATTTTGGCGAACAGCCAGTAGGAGGGGAAACCATACTAGCTTACCTGGACTATTTCGGTGATATTATGTTTGGCTATTCTACTTTGAGAGCAGTTAAATTACATTTGGAAGCTGGGCACAAGCAATTCTATTTGTATGAATTCAGTTTCGTGGATGATAGTGTGCCTGTCATACCTAACACAAACGAACGTCGTGCACAACATTGCGCTCAAACTATGGCTGTACTCGAAGGACCTGTAGATGAAGATACTTTATCTGAAGAGTACAGAAATTTAAAGTCTGTTATAAGAGAAATGTGGTCAAACTTCGCTATACACGGGACTCCTGTGACTGAAGATTCGTCGCTGCCTACATGGCCTACGACAGGTGCAGGTGGGAGTCCATACATGTCTATTGGAAAAGCAGTGGAGATAAAAGAATCCTTTTTGGGAGAACGTGGGAAATTCTGGAACAAAATCTATGAAAAATACTACAGCGCCCCAGTGGCAGTGGCGCCACCTCCACCACAAGCATATCACAATGAACTGTAAATGAAACAAGCTGCTAATTTTTGTATATATTTTTTTAATAAAGATTTAAAAAAAGTTATTTAAATCTTAATTCA

>gene11105

CCTGACCGTTGCGGACGGTGTCCTGAGAGCACAGTGCCGGCCTAAGTATGGTGCAAGTGAGAGTGAATGAAGGTCTGCTGGAGGGAGAGCGAGTGGACAACCACTACGGGGGCTCCTTCTACAGCTTCAAGGGAATACCGTACGCACAACCACCACTCGGAGATCTTCGGTTCAAGGCTCCTCAACCACCTCTACCATGGGAAGGTATCCGGAGTGCGAAGGAGTTTGGATCAATATGCTACCAGAAAGATTTCGAATCGCAAACCAAGACAACAGGAAGCGAAGACTGTTTGTTCCTCAACGTTTATACTCCAGAATTGAAACCCCAAAATCCTTTACCCGTAATGTTCTTCATCCATGGCGGAGGATTCTACTCTGGCAGTGGCAACGACGACATGTACGGTCCAGAGTTCCTCGTCCGGCAAGGAGTCATCCTAGTCACGATCAACTACAGACTTGAAGTACTGGGTTTTCTTTGTCTAGACACTGAAGAAGTACCGGGCAATGCAGGAATGAAAGATCAAGTTGCAGCTTTACAATGGGTTAATAAAAATATTTCAAACTTCGGTGGTGATCCCAACAATATTACGATCTTTGGTGAGAGTGCTGGCGGTGCAAGCGTCGGCTACCATTTAGTTTCGCCCTTGACCAAAGGTCTTTTTCAAAGAGCGATTTGTCAGAGTGGAATCACAACTTGCTGGTGGGCTAAAGTTTTTGAACCTCGATTGAGAGCACTAGCATTAGCAAGACAATTAGGATATAATGCGGAGAAAGATGGTGAAGACCTTTATGAATTTTTCAAATCTCAACCAGTTGAGAAACTCGCTGGAGTAAAAGTACCATTATCTTACTCAGAACAACATCGAGCTAGCTTCGAATTATATTTTGGCATTGTAAGCGAAAAACAATTTGGCAACAATAAATATTTCTTTCATGGTAATATATTTGACTATTTGAAGAATGGAATCCATGAAGGTGTGCAAGTTATTACTGGCTATACTGCAGATGACGGTGTCCTACTACATGTGTTTGACAAAGATGGAAAAATGTTAGAACAGGCCAATGAATTAGATACCTTTTTCACTCCCAGACATATTGCATTGAATTGTTCTCTAAAAGAACAAATTGAGGCCGGACATAAGATCAAACAATTTTACATGTGTAACGAAGATATAGACACAGACTGTGAAAGACTGAGTCGCTTTCAAACTACAGAAATGTTTTTATATGGAATCATAAAATGGATGAAAATTTGCTCACAAAGAAATAAGACTTATCTTTATAAATTCACGTGTAAGAGTGAAAGAAACGCTGCAGTCCGCTTGATGGGACTGGAAGCTGTGACACAAGGTAAAGAAGTAGCTTGTCACATGGATGACTTGTTTTATATTTTCAACAGCAAGGTATTAGATGCAAAAGTCGATATGGATTCGAAAACATTCCAAATGATTGACAATGTGACAAAATTATGGACCAATTTTGCTAAATATGGAGATCCGACTCCCGATAACAGTCTTGGTGTTAAATGGAACCCTTACACATTGGACAAAGAAGAATATTTGGACATCGGCAATGACCTCCAAACTGGATCTGCGCCGGATCGCGACGAGTTTTTATTTTGGGAGAGTATCTTTAAAGAATTTACTCCTGAATATGTCGATAAAGTTCATTAACATTGATGTTCATTATAACATTAT

>gene11107

GTGGCAGTCTTAGTCTTGTTTGGGGCTCTGACCGTTGCAGACGGTGTCCTGAGAGCACAGTGCCGGCCTAAGTATGGTGCAAGTGAGAGTGAACGAGGGTCTGCTGGAGGGAGAGCGAGTGGACAACCATTACGGAGGCTCCTTCTATAGCTTCAAGGGAATACCGTACGCGGAGCCACCAGTCGGGGATCTGCGGTTCAAGGCACCGAAACCTCCAAAGGCTTGGGGCGGAGTTCGTTCTGCTAAGGAATTTGGCCCGAAATGCTACCAAAATGACCTTTTCATGAACACTGGAATAGTTGGTGAAGAAGATTGTTTGTACCTAAACGTGTACACACCTGAGATCAAACCTGACAAGGCTTTACCAGTAATGTTCTGGATACATGGAGGAGGCTTCTTCTGTGGTAGTGGTAACGATGACCTTTATGGTCCAGAGTTCCTTATCAGGCACGGTGTCATCCTTGTCACCATCAACTACAGAGTAGATGTGCTCGGTTTTCTTTGTTTGGACACAGAGGATATTCCTGGCAACGCAGGCATGAAAGATCAGGTACAAGCTCTTAGATGGGTGAACAAAAATATTGCTAGCTTCGGTGGTGACCCCAACAACATAACCATATTTGGCGAAAGTGCCGGTGGAGGCAGCGTCAGTTACCATTTAATATCTCCGATGTCTAAAGGTTTGTTTAAAAGGGCCATTGCTCAGAGTGGGGCGTCTACTTCGCCCTGGGCCCAAGCTGTTGAGCCTCGCGAAAGAGCTTTAGCATTGGCTAGAAGCCTTGGATTTTATTCTGAGGACGATAAAGAATTGTATGAATTTTTCAAAAATCAACCAAAGGAGTCAATAGTGTGTATTCCTGTAGCACCAACATTCTCCGAAGCCTTCAGAGGTGGGTTAGAAATCAACTTTACTGTTGTAAATGAAAAGAAATTTGGAGATAACGAGAGATTCTTTTACGGAGACATGCTTGATGCAGTGTCAAACGGTGTTCACGAAGGAGTAGAAATCATGACGGGGACTACTACAGAGGAAGGTCTCATTATTTTCGGAGACCTAGAGGTTACTAAAAAGTTATTAGAATCAGCAAGAGCATTCCCAGAATCCTTCATTAGTAAACAACTGGCATTCACCGTCCCATTGAATCAGCAGCTAAAGATGGGAAGGGAATTCAGAAAGTTTTATTTCAAAGACCAGGTCAATATCCCAAATGATTGGGATAAATGGATGAATTTCGTTTCAATGCAGATGTTCGGATACCCAACAATGCAGTGGGTGAGACATTGTGCTAAAGCGAAGAAACACAAGCTGTATGTGTATAAGTTTTCTTGTAAGTCTAAGAGAAATATGATAGCTGTTTTGAGAGGACTCACTGAGATGCTTGGAGACAGACAAGTTACGTCTCATGCTGATGATATAGCCTATCTTTTTAACCCTAAACTGATGAAATTAAAAGTGGACACTGCTTCCGAGGAGTTCAAGCTGATAGAAAGAGTGGCAACGCTTTGGACCAATTTTGCTAAATACGGAAACCCAACGCCTGACGATTCTCTGGGAGTTAAGTGGGCACCGTACACCCTGGAAAAACAAGAATATCTGGACATTGGAAACGAGCTAAAAGCGGGCACCGCGCCTGATGCTGAAGAAACACAATTCTGGGATAAATTGTATGAAAAATATGTATTGTGATTTTATCTAGACTTTCCATAGGATTGGTGGTGTTCCCTTTAGGGTCGAAAAATATAGCATTCTTATATATTACACGGATATATATGTTAA

>gene14196

TTCGAAGCAGCGCGATCATCTGCATGCGGTCACGTCAAACCGGTTTCATTTATTAGACTACTTTAGAAAAATGTTTAAGCCTATTGTGATATTCGTTGCTTTAAATTTTCTTTTAACGATTTATGTCAGTGATGTGAAGAGCGAGAAACAGCCGGTTGTACGGACCCCGCTAGGCGAAGTGGCAGGGTACTACATGCAAACTAGAGGAGGGAGGCAAATAGCAGCATTTACAGCTATCCCCTTCGCGGTTCCTCCCGTCGGAGACTTGAGATTTAAGGCCCCAGTTCCTGTAGAACCATGGCAAGACACTTTAGATGCGACCAAAGAAAGCCCTGTGTGTGTTCAGAGGAACCCGTACACTCGGCAAGAAGAAATCGTCGGACAAGAAGATTGTTTGTATCTTAACATCTATACACCAATAACGGATAATGATGTGGGCGAGAAAAAGAAATTGTTACCAGTCATGGTATTCTTACACGGAGGTGGTTGGATGTGTGGTAGTGGCACCACTGAAATGTACGGACCGCAGTTTCTTCTAGATAGAGATGTTGTATTTGTTGCGACCAACTACAGATTGGGTCCACTAGGCTTTCTCTCTACTCAAGACGAGAACTGTCCGGGCAATAATGGCCTCAAGGATCAGCAAGAAGCTTTGAGGTTTTTACAAAAGACCGTAGAGAGTTTTGGTGGTGACCCGAACTCTGTAACAATATTCGGAGAGAGCGCTGGAGGGGCCAGCGTCCACTACCATATGATATCAGAGAGCAGCGCAGGACTATTTCATAAAGGAATATCAGAGTCAGGTACGGCTTTAGTGCCGTGGGCCGAAGCCCCGCCTGGAGAAGCCCTCCGGAACGCATTCCTTTTAGCAAAGTATCTGGACTGTCCTCAGGCACCTTCTGCTAAAATGATCGAATGTCTTCGCACTAAGGACAGCTATGACATCATCGGCACGGAATTTAGTTTTTACAAATGGGATTATGAACCAATGACTCCTTTTAAAGCTGTGGTGGAACCAGACCTTCCCGGGGCATTCTTGACGAGGAGTCCTCGTAAAGTACCAAACGTGCCTGCAGTCCCTTGGATGACTGGCCTTACCAAAGACGAAGGCTGCTTGAAATCAGTTTGGATTACAGCGTTTGAAGGCAGATACAACGAGTTTATGTCGGGCTTCGAAACTATTGCTCCAGTCACATTTTACTACGAAAACTCGCCGTATGCGGAGGAAATAACTCAGAAGATCCGTAATAAATATTTTAAAGAAAATGACTTACAGGCTACACAACAAGGAATTCTTAATATTTACTCAGATTCGTATTTCGCTTATCCAGCCATAGAAGCTATAGAGTTAGTATTCAACTACACAAAGAATCCTGTGTACCTTTACGAACTGACATATCGAGGAGCCAACAGCTTCTCACAAATATTCGGTGATCCGCAAGGAGATTATGGTGTATGCCATGCTGATGATCTTATGCATTTGTTCCCTATCGGGTTTCTTTCAAACCCCTTTTCACCTGAAGATCTAAAGATGAGTGAAACCATCGTGACTCTATGGACCAATTTTGCAACTACGGGGAATCCTAACAAACCTGTACCCGTGCCATTCAAGTGGGAAACAGCGACTTCTGGAAGTAAAATGGAGTACCTCGAATTAGGTTTTAAGCCTCACATGAAGAAAGATCTGGCGCCCCGATCTCGCTTCTGGGGCGAGTTACCCCTATGGCAAAATATGCGCAGTCGCCGATATATGGATGAATTGTAAATATTAATAATAAAACAAATATAATATTTTATGTAAGTATATTTTGTACCTATTTCTTAAAAAATAAATCTTACAAGTAATTAATCTAACGTTACAACCTACCAACCACTGAATTGTTTA

>gene148

CACGGTCTATAGCGGAGAGCGCGCGTGCGCTGTGTTAAAAAAAGAAAAGTGTATTTGTGTGTGTATAAACATGTTCGTGCAAGTTTTGTTTGTGTCTTTCCTTGCGGCGTATGCGCATGGTTTGCAAGTGAATACTACTGAGGGTATTGTTGAAGGATCAAGAGCAGTTGATGGCGATTATTTTGCGTTTTACGGTGTTCCGTACGCCGGGCCGACGTCTGGGCAAAACAGGTTTAAGATGCCGATGCCCCCAAAACCATTTCCTGGAGTGTTTCGAGCCACCGACAGCAACATATTTTGCGCCCAACCCTCCTCCCGAGGCCTCATTGGAACCGAAGACTGCCTGACATTGAGCATCTTCACAAGAAATGTCACTTCACCAAAACCAGTCGTCGTCTTCCTCAACTCTGATGAGTACACCAATACAAACAACCAACCCATGTCCTACAGAAGATTTGTGGAACAAAATATTGTTTTCGTGACTATGAATTTCCGATTGTCCATTTTCGGATTTCTTTGTCTCGGCGTCCAAGAAGCACCTGGTAACGCAGGCTTAAAAGACGTACTTCAAGGACTCAAATGGATTAAATCAAACATTGCGAACTTTGGAGGAGACCCAAATAATGTTATTCTAATGGGGCATGCGTCTGGTGGTGCGCTGGTCGATCTCCTCACGCTGTCTCCTCAAATTGAGAATCTAGCGCATAAAGCTATAGTACTCAGTGGTTCTGCGTTAGCTCCATGGGCTGTAGGTTACGATCCTGTTGGATACGCCAATCTATTGGGAACAAAACTTGAGTACGACCACAAAACTCCCGAAGTATTAGCACAACGTCTAATCAATACGGACATTAATGTTTTACACACTGCATTGAATGACTTCAAGTTCTTTAATAACACGCCATTGTTCGCTCCTTGTGTGGAGAATCCGAAACTTAGTCCAAACCACACAGTTCTTGGTGACGCTCCTATCAACATCCTGAGATCAGGTAAATACGTTCAGATTCCCGTTATTTACGGTTACACCAACAGGGAAGGAACTATGAGGGCTGAAGAGGCTGACTTTGGAAATTGGTTGACTTTAATGCAAACCAACTTTACCAGTTTTCTGCAGGTTGATTTAAAACTAGCCAATAACAAAACTGCCGTGGCAGCATCCATCCGTGACTTCTACTTTGGAAGCAGTGCAATCAGTATGGGAACTATTGAAGACTTTTTGGATTATCAAGGAGACACGCTAGTCCTCGTCTCTACCATCAGAGTAGCTAAGGAAAGAGCTCTAACTTCTAAGAGCGAAGTGAGATTAATGGAATTTGCTTATCTGGGAACCCTAAACTCCAATTGGATTCACAACCAGATTCCTCTGAGTGGGGCAGCGCATGGAGCCTTCCTAAATTATTTGTTCGGGTATGACCTACGTCCTGCTGATGAAGCAGTCAGCAGGGCACTCGTTGGACGTGTGTCAGCCTTTGCTTACAATTGGTCAAACAATACATGGGCAGCCGTTACAAAAGACTCCACAAATTACCTCTACTACGGTGGTAATGGGGCTCCAACGGCCAACAACACTTCTATCTACGTGGAGGAACCCAGGTTCAACCCCCATGTCCAGCGCATGTCCTTCTGGAACAACCTTTATGAGAAGTTCTATGTTGCTCCTACGCAGCCTAGTTCCTCAAATATTATTGTCAGTTCTTTATTATTTATTATGCTCTGTCAGATGGTATTGCGTTTGTATTAAGTTAGAGTATTGCCAATGTTAAAAAATCGACTGTCATATTTTTTGTAAATGTGAGAAAATTAGACTTTTTGTTCAGGTCCGTGTACAAAGTGTTATTTGTTCATTGCAAACCATTAACATTGGAGTCAACAAAGTTATGAAGTAAATTTATAGATATCATGGTATTAGGTATGTGAGTGATGTATTCATGATGCAAAAAAATAATCACTAGTTTTCCATATTTTTGCTTATTTGAACAAAGAACTACATTTTCAGACATCATCACTTCTGTAAAAACTTATTTTATTGTATTTACATGCCAATGCAGCTAACACGAAGGTCAGGTCAGGTCAAGGTCTGTCTTTGATTTATCGACAAATAGTTGTCACAAGTATTTTTGTCGCCATATTTAATTAAGTAAATTAGGTTCTAAGTCAATCTTTAATTAAGGAAAGTGATTGAAAGTGTGTACGAAGTGGACATATTGAAATAAACGTAAATTATATTGAAATAAGAACCAATATCGTAAATAAAATATCTGTCTAAGTATTGCAATAAAAAAAGAAAGAGTATATAATCACATTTATTTTGTTAAAGTAGAAAAGATTAAACTTATAAACTTCATCATTTAACCGCTTTATTGATTGAAATTTCAGAGTCAAATGTGTGTGTCAGTTAGTAAATGTCGACCTCATCGTTCGCTTGATAAAATTGACGTGTAAAAGTGCTGCCTTGTAGCCTATTTGCAGAAATAAATTCACTTGTTTCACTTGTGTATAAGGGTCTTTTTTCAAAATACCGTAGAGACTGAAATTAGACTCGATATTAGTCCTATTAAAATGAGATTTAATAACATCACTTGTAAAAATGTAACGTAAGTAGGTACTATGTTTATATTATTAACGAATAAGTTATTATTACGCAATGTATGGAATGACTCCAAGTCAAGTCAAGAGTGATGTGGCTAAGAGCTATATTCATAGATATAATAAAGAATAACTGTTTTATATATTTGACTCGTTGACCGAGTGGTCGCAAATGC

>gene15885

ACCAATTCTACGCGGTATTAAGTGTCGATGCATTGACAATGGTGCAAGTTCAAGTAAACGAGGGTTGGTTGGAGGGAGAGGTGGTACAGAACGAGTATGGCGGCTCCTACTACAGTTTCAAGGGGATCCCGTACGCACAACCTCCACTTGGTGAACTGCGGTTTAAGGCCCCTCAACCGCCAAAGCCCTGGAAAGATGTCCGCAGCGCGAAACAATTCGGTTCAAAATGCTACCAAAATGATGCGTTCTTTGAAAAAGGAAAAGTGTGTGGTTCTGAGGACTGCCTCTATCTCAACGTGTATACACCAAGCATCAAATCTGACAAACCTTTACCAGTCATGTTCTGGATTCATGGAGGAGGTTTCGTATCTGGAGCTGGTGATGACGACGTGTACGGCCCCAAGTTTCTCGTAAGACAAGATGTCATCCTTGTCACGTTCAATTATCGACTTGAAGTTCTTGGTTTCCTCTGCTTAGACACTGAAGATGTGCCTGGAAATGCTGGTATGAAGGATCAGGTAGCAGCCCTCAGGTGGGTGAACAAAAATATTGCCAACTTCGGAGGTGACCCAAACAATGTAACAATTTTCGGTGAGAGCGCTGGGGGGGCCAGTGTCACTTATCACTTGATATCACCAATGTCCAAAGGATTGTTCCGGAGAGCGATAGCCCAAAGTGGTACCAATGTTGGGTATTGGGCTCAAGCGTACAAACCTCGTGAAAGAGGCTTTGCGTTGGCAAGGCAATTAGGTTTATACTCCGATGATCCTAAACAAGTATACGAGTTCCTAAAAGAACAACCTCTCGAGGCTCTTATTAAAACTGTTGTACCGATAACATATTCAGAGAAAGCACGAACCTACAATGAGATATATTTTAATGTGACTGCTGAAAAACAGTTTGGAAACAACGAACGATTTCTCTGCGGAGATGTACTTGATTCTGTGTCGAATGGTATTCACGAAGGAGTTGATATTATGACAGGATACACAGCGGATGAGGGTATAATGAGCTTGGCTGTCTTTGGAGATATATCTGCAAGTTTAGAACAAGCTAGAAACTTTCCGCAGTTTTTCATTTCGTATCCAATGTCTCTCACATTGTCCACTAACGACCAACTGGAGCTTGGGAAGAGAATAAGAGAATACTATTTTAAGAATTCGATTTCGGTACCAGATGATTGGGAGAAGTTGGAGAAATTTTATGCAGCTGACCAATTCGTGTTTCCTGCAAACAGATGGATTAAATTGTGTGCTCAATCTAAGAAGAACAAGACATATTTTTATAAGTTCACTTGCGTATCAGAATTGAATATAATATCTCAGATGATGGGCGTAGGTAATTTAATTAAGGACAAACCAGTGGTAGGTCACGCAGATGACTTATTTTATTTGTTTACGCCGAATAGTTCACCAATAATGGACAGGAATTCGAACACATTCCAACATGTGGAAAAGGTCACGAAACTTTGGACAAACTTCGCTAAATTTGGAAACCCCACACCTGACGATTCATTGGGCGTTACTTGGACTCCGTATTCAGTGGCCAACAAGGATTACCTAGATATTGGCAACGAGCTCAAACCTGGTCAGGCGCCGGACGACGAGGAAGTACAATTTTGGGAAAACGTCTTAACAGAATTCAAACAGAAATTGTATTGATTAGATATATTACTTTGTAAACAATACATTATATAAA

>gene15886

GCATTGACAATGGTGTAAGTTCAAGTAAACGAGGGTTGGTTGGAGGGAGAGGTGGTACAGAACGAGTATGGCGGCTCCTACTACAGTTTCAAGGGGATCCCGTACGCACAACCTCCACTTGGAGAACTGAGATTTAAGGCCCCTCAACCGCCAAAGCCCTGGAAAGATGTCCGCAGCGCGAAACAATTCGGTTCAAAATGCTACCAATATGATGCGTTCTTTGAAAAAGGAAAAGTGTATGGTTCTGAGGACTGCCTCTATCTCAACGTGTATACACCAAGCACCAAACCAGACAAACCTTTACCAGTCATGTTCTGGATTCATGGAGGTGGTTTCGTATCTGGAGCTGGTGATGACGACGTGTACGGCCCCAAGTTTCTCGTAAGACAAGATGTCATCCTTGTCACGTTCAATTATCGACTTGAAGTTCTTGGTTTCCTCTGCTTAGACACTGAAGATGTACCTGGAAATGCTGGTATGAAGGATCAGGTAGCAGCCCTCAGGTGGGTGAACAAAAATATTGCCAGCTTCGGAGGTGACCCAAACAATGTAACAATTTTCGGTGAGAGCGCTGGGGGGGCCAGTGTCACTTATCACTTGATATCACCAATGTCCAAAGGATTGTTCCGGAGAGCAATAGCCCAAAGTGGTACCAATGTTGGGTATTGGGCTCAAGCGTACAAACCTCGTGAAAGAGGTTTTGCGTTGGCAAGGAAATTAGGATATAACTCTGACGATCCTAAACAAGTATACAAGTTCTTAAAAGAACAACCTCTCGAGAGTCTTATCAAAGCTACTGTACCGATAACATATTCAGAGAAAGTACGAACCTACACTGAGTTTTATTTTAGTGTAGCTGCTGAAAAACAGTTTGGAAACAACGAACGATTCTTCTGCGGAGATGTACTTGATTCTGTCTCGACTGGTATTCACGAAGGAGTTGATATTATGACAGGATATACAGCGGATGAGGGTATATTGAGCTTGGCTATCTTTGGAGATATATCTGCAAGTTTAGAACAAGCTAGAAACTTTCCGCAGTTTTTCATTTCGTATCCAATGTCTCTCACATTGTCTACTAACGACCAGCTCGAGCTTGGGAAGAGAATAAGAGAATACTATTTCAAGAATTCCATTTCGGTACCAGATGATTGGGAGAAGTTGGAGAAATTTTATGCAGCTGACCTATTCGTGTTTCCTGCAAACAGGTGGATTAAATTATGTGCTCAATCTAAAAAGAACAAGGCATATTTTTACAAGTTCACTTGCGTATCAGAATTGAATACGATATCTCAGATGATAGGCGTAGGTGATTTAATTAAGGACAAACCAGTGGTAGGTCACGCAGACGACTTACTTTATTTGTTTACGCCGAATACTTCACCAATAATAGACAGGGATTCGAAAACATTCCAACATGTGGAAAAGGTCACGAAACTTTGGACAAATTTCGCTAAATTTGGAAATCCCACCCCTGACGATTCATTGGGCGTCACTTGGACTCCGTATTCAGTGGCCAATAAGGATTACCTAGACATTGGCAACGAGCTCAAACCTGGTCAAGCGCCGGACGACGAGGAAGTACAATTTTGGGAAAACGTCTTAACAGAATTCAAACAGAAATTGTATTGATTAGATATTACTTTGTAAACAATATATTAGATAGAAATGTTATTATTTTTGACTTTGTTTAATG

>gene1782

GAGTTTGGTGAAGGTGACCCAGTGGGTCTTTGGGGTCCAGTAGTAGAGCCAGATTTTGGGCAGGAGCGATACCTCCCTATAAATCCATTAGTTGCAATTAAAGAAGGTAAAATGCACACGGTACCCCTCATCGTGAGTCAGACTACTGACGAGTTTTTCTGGAAAGCATTCCCGGTCTTACAAAACGAAACGCTTTTGAAGACAATGAATGAAGAGTGGGAGAGAGTCGCTCCGATCTCGTTCATACTACCGAAGAAGAACCGTGGCGCGGCGGTACAGAAACTTAAAGAGGTGTACCTTAAGGGGAAGAAGTTGACGAATGACAAACAAAGTGAGAAGGCTCTTGGACAACTTTACGGAGACTCCGTCGTTGGGTTTGGGGTGCACAGGCTAGCTAACCTAATGTGTCGTCACTCCAAGCATCCAGTGTGGTACTCAGAATTCGCGTACGTTGGGAACAACTCCCACTATGAGGACTCTCACGGCAAACCACAAGGAGCGGCGCACCACGACGACCTGCTGTACCTGTTCACGCTGAGCTACCGGTTCCCCGTGATCTCGCTGGACAGCAAGCACTCGCACGTCGTCGACGAGATGACGGCGCTCTGGTACAACTTCGCCAGATACGGAGACCCGAACCCGCGCGGTGACACGCCGGAGCTGGGCAAGCTGTCGTGGCCCGCCATGACCGCCGCGGACAGGAGGTACCTGCACCGCGGACAACAGCTAGAGGTGCGACAGAACATGTTCGAGGACCGGTACAAGATTTGGGACGACTTGTACCCCATACAGTACTGATCAGTAGTACCATAAATTCTTATTGTTAACAGTTA

>gene1784

TGTGTCCAACAGCGTGACACACAATGTTGTACGCGCTGCTGTGTGCGTTGTGTGTGTGCGCGGTGCACGCACACCAACACAAACATCAGACAGAGCCAGCAGAAGTGAAGCCGGCCGCGCCACTGACACACAGCGTGTCGGGCACGTTCCGCGGCTCGTGGATGGAGACGCGCCGCGGGAGACGCTTCCAGGCCTACCGCGGGATACGATATGCAGAGCCCCCTGTCGGAGCATTGAGGTTTCAGCCACCGAAGTTGAAGTTGCAGTACGAAGGTGTAGTGGACGCGAGCGAGGAGGGCCCAGCCTGTCCTCTGCCGGCGCCTCCCACCTACTACGTTGCCGAGGACTGCCTCACTGTCAACGTCTACACGCCGATACTGAACAGCACCAAACCGCTCCCCGTGATCTTCTTCATCCACCCCGGCGGGTTCTACGCGATGACAGGTCGTAGTGATTTAGCTGGTCCTCACTACCTACTGGATAGAGATGTTGTCCTCGTTACCATCAACTACAGGATTGGATCCTTAGGTTTCATGAGTACTGGTGATGCTTACGCTCCCGGTAACAACGGCATGAAAGACCAAGTAGCCGCCCTCAAGTGGGTGCAGAGGAACATTGCAGCGTTCGGCGGTGACCCGAACCTCGTCACGATCACAGGCTGCAGTGCCGGCTCCATCAGTGTCATCTTGCATATGATATCGCCCATGGCGAAGGGTCTCTTCCATCGCGGTATAGCAATGAGTGCGTCTCCTGTGAGCAAAGAAGTGGAAGCTATAACTCATCAGCGACACCTCGCAGTACGCCAGGCACAGATCCTCAACTGCCCGACAGAGAACTCGTCCGTCATCGTCGACTGTCTCATGACCAAGCCTTGGAAGGAGCTTGGAGATTCTCTGCCTAAATTCTGG

>gene1785

TAAATAGGCCGAGGCATGTGTCGCGGGCTGCAGTGGAGTGTCGGCGGCGTGAGGATGCTGGCGGCAGTAGTGTTGGTGTGTGGTGCGCTGGCCGTCGCCGCGAACCATCACCACCATGTTCATCATGACCTTGATCATTCGACAGCGTCTACACCAGCGGCTCCAGTAGTCCGCAGTGTGTCCGGTCAGTTCCGTGGCTCGTGGATGTCGTCACGACGTGGGAGACAGTTCGAGGCATACCGAGGCGTCCGATACGCTCAGCCACCCGTTGGGGAACTGCGCTTCCAGCCCCCACAGTTGATGGAGAACTACACGAGTGAGGTAGACGCCTCGCAGGACGGGCCCGCCTGCCCGCAGCCCACCTTTAACAACTACCCAGTACACGAGGACTGTCTCCGCCTCAACGTGTACACTCCCGACCACCAATCCAAGAAGCCCCTGCCAGTGGTGGTCTTCATGCACGCGGGCGGGTTCTACTCGGTGTCGGGGCGCAGTGACGTGGCCGGGCCCCAGCACCTGCTGGACCGGGACCTCGTGCTCGTCACCATCAACTACAGGCTCGGATCACTTGGCTTCTTAAGCACAGGAGACAAGTACGCTCCTGGTAACAATGGTTTCAAGGACCAAGTTGCAGCTCTCCGCTGGGTGCAGAGGAACATCGCTGCCTTCGGAGGAGACCCCAACCTGGTCACTATCTCAGGGTACAGCGCTGGATCCTTCAGTGTCATGTTGCATATGATCTCACCCATGTCCAAAGGTTTGTTCCACCGCGCCATTTCAATGAGTGGCTCCCCAATATCTCAAATAGTGATCCCTCGCCACCAGCGCCACCTGGCGGAGAGACAAGCCAAGCTGCTACAGTGCCCTACTGACTCTTCAAAGGCTATCATCGACTGCCTCAAGACTAAAACATCCAAGGAACTGGGAGATTCTTTGGATAAGATGTTTGACTTCGGCTACGATCCAGTATTGCTGTGGGTGCCGATACACGAGCAGGACTTCGGGCAGGAGATGTTCCTGCCGCAGCAGCCGCTGGCGGCCGTGTGTGCGGGGCGGCTGCAGCGCCTGCCGTACATCGTCAGCCAGACACACGACGAGTTCTTCTGGAAGGCACTCGATGTCCTTCGGAACCCGAAAGCGTTCGACAACTGGCGCGCGGACTGGCCTGGATTAGCCAAGATAGCGCTGTACCTGACCGGCGCTGGAGACAACTCCAGCATCACCGCCGCCGCCAACCGACTGAAGCAGGCGTACCTCGGAGGGAAAGACATTAACAATGACACCGCCACTGCTGATGGCTTCGGGAAACTTTACTCCGACGCTATCATTGGGTTCGGAGCTCACAGGCTGGTGAACCTGGCGGCGCGGCAGTCCCCGCGGCCGGTGTACTACTACGAGTTCGGGTACATCGGGAACAGCAGCCACTACGTCGACCCCGACACCAAGCGACCGATCGCTGCGGCCCACCACGACGACCTGCTGTACCTGTTCAACGTGAGCTACAGCTTCCCATCCATCCCGCCGTCCGACAGCAAGGACTCCAAGATGGTGGACAAGATGACTGCAATATTCTACAATTTCGCTAGACATGGGGACCCGAACGACAGGGGCGACACTCCGGAGCTAGCAGACATGTCATGGCCGCAGTTCAAACCAGATGAGAGGAAATACCTCCGTGTCGATACTCCCTTCTCAGTTCGAAGCAACCTCTTCGAAGAGAGGTTCAAGGTCTGGGAGGAACTGTTCCCTCTTGATTATCAGACTTGTAAATAGATTTTTTTACTTAACTAACAATGGCTCCAATATTGGTCCCAGTATTGTTCCCAAGGTAATGCAAACGCGTGGCAGGGGGAGCGTTGTTTAAGGTATCATTGAGGCATGTGCAATGATGTATGTGTGTACCAAAACTAAACATGTATCTAGTACAATAAATTTACAAAATAAAAACATTTTAAC

>gene181

TCGATCATTTCACATGTACAAGCCGTAATGATATACTCAACAAAATGTTTTTAAAAACATTCCTTGATTTTATTTATTTTTTATTAATATTTAATTTGATTCAGTGTCATGAAACTCCTGTTGTTGAAACAAATTATGGACCTATTTCTGGAAAGGTTTTAAAAACGCTGATTAAAAATGTGGAATATTTTGGATTTATGGGAATACCATATGCAGCGCCACCTGTTGGTGAATTGCGATTCATGGCACCTCAACCCATCGACCCTTGGGATGAAACGTTGAAAGCAACACGAGAGAAACCTGCCTGCATACAATTCAACAATGACATCAAAAAAGGACAGGACTTAGGTTGGTACGGAGACGAAGACTGCTTATACCTCGATGTATTCACGCCAGCTATAGATGAAGAATCAAGGCCAGTAATCGTATTCATATACAACGAACATTTCCAAAATTCTTACAACAAATCTAAGGATTATGCTCCAGACTTTTTCATTGAAGAAGACATTATTGTTGTAACTTTAAGTCATCGGTTATCAGCATTTGGATTTTTGTCGCTAGAAAATGATATGGTGCCAGGTAACGCTGGTCTAAAAGACATCGTTGAAGTTTTAGAATGGGTGAGAGACAATATTAACCAGTTTGGTGGAGACCCGCAAAGAGTCACCCTCATGGGACTGGAAGGAGGTGCAGCTGCTATTGATTTACTTATACACTCAAACGCAAAATATCTTTTCCAAGCAGCCATACTGCATAGTGGTTCATCCTGGTCTTCAGCATATTTACAAGAAGAAGTTAGAGATAGAGCATTCAAACTTGGAGAACTAATAGAAAGAACTTCGACTGACGAAGTTAAATTACTGAAGGAGCTCCAAGATATACCAGCTAAAGATTTACTTACAAAAGATGTGCATGCAAGTCCTGATGATTACTTTAAACAGACACAGAAGAGTGTAGTTGCATTCAGTCCAATAGTAGAAAAACATCCAAAGGGCTTAATTAAGGAATACCCGGAAGATTATATGGAAATTATTGAAATGCCAATCATGATTGGTTCTAATTCCAGAGAAGCACTAGAGCCTATGATGCAATATCTGATAGAACCAAGATACATTACTTTTCTTAGGAAAGACTTCCCTCTTCTCTTACCAAGAAGGTTGAGATTCCAATTTGATCCATTGAAAGACGTGTATGATGATGCAACGAAAGACATAAAAGATTTTTACTTTAAGAACGGTGAAATTAATCTGAAGAGTGTGCCAGATCTACTCACATATATGGGGGATGCATCAGTCAATTATGTAGTGGATTATACAGCTAAACTATATTCTGAAAGATCAGAGAAACCAGTGTACTATTACTATTTTGATTACATCAGTAATTTGAACGAGAATAAAAATAATTTGATGAAGTATTCCAATGTGCAGGAAGGAACGTGGGGAACTGCTGCTGGGGATGAAATGTGTTATCTGTTCAAGTGCCCCGCTCTAACAGATGAATATTTAAAACTTGAGAAAAATGTGCCCGAAGAAAAAGTTATTCAAAGGAAAATGATAAAAATGTGGACGAATTTTGCTAAATATGGGAACCCAACACCAGACAACGATGAAGAACTAGGACTTAAATGGCCTGCATATACTACAGACAAGAAGGAATATCTACATATAGACAAACACATGAAAATCAAACAAGATCTGAATAAGAAACGATACGAATTCTGGGATCAATTCATTGATAAATGGGAAGCAATGGCCGTCAACGGAATTATTAGTGAATCAATTAACAAAAAGGACGAATTATGATGAATATAATGTTTTATTAAATTATTTTTTC

>gene2200

ATGTCGTTAAAGTTATTTCTTTTTTTAACGGTCGTAGGTTTAGTGGCTGCTGAGGGCCCTAATCCTACGATCCGTGTGGCTCATGGGTTACTGCAGGGATCTTGGAAGGTGTCCACTAAAGGCAGGTCCTACGCCAGCTTCCAGGGCGTTCCTTATGCAAGGCCACCGATTGGAAAGTATAGATTCAGGGAACCACAGCAGCTGAAGCCATGGATCGGAATATGGGACGCCACCAGACCACTCCCCGGGTGTCTGAAGTACGACCCCTTCGTGAAGGAGATTACAGGTAGCGAGAACTGCTTGTACGTGAACGTATTCACTCCGAAAATGAACCCTGGCGCCAACCTTCCAGTCGTGGTCTTCATCCATGGAGGTGCCTTCATGTATGGTGAAGGAGCGATATATGACCCCAGCAACCTCATGGACCGGGATATGGTTGTAGTCACCTTGAACTACCGACTCGGACCTCTAGGTTTCATAAGCACAGGCGATGAGTTCGCACCAGGCAACATGGGTCTAAAGGATCAGTCGTTTGCCCTGCACTGGATCAAGAATAACATCCTCATGTTTGGAGGCAACCCTGACAGCATCACCTTGACCGGCTGTTCTGCTGGAGGGGCCAGCGTGCACTATCATTACCTGTCACCTTTGTCTAGAGGAACCTTTCACCGAGGCATAGCGTTCAGTGGATCTGCCCTTACGGAATGGACCCATTCCATCAAGCCTGCAGAGAAGGCCAAGGCGCTGTCTGCCATCGTCGGGTGTCCCACCAACAATAACAAGGAAATGATGGACTGCCTCAAGTATAGGCCAGCTGAATCCATCGTCAACGCACAAATTGATATGTTTGAATGGAAAGTGCACATGTTCACGCCGTTCTCTCCAGTGGTGGAGCCCCCAGGAGTCCGGGAGCCGTTCCTGCAGCAGTACCCGTACCATGCTACTAGAGCTGGACAGATGATGAACGTGCCACTTATTGCTAGTGTTACCTCCGAGGAAGGATTGTACCCGGGTGCCGTTTACCAAGAGTCACCAGACCTATTGCCGGATCTAGAAGCCCACTGGAATCAGCTGGCTGCCAACATCTTCGAGTACAATGACACACTGCCACTTAACCGACGCGACGAGGTTGCTCAGAAGATCAAGCAGCACTACTTGGGAGGCAAGCCCGTTAGCCAGGAGACATATCCACAGCTGATTCAGGCTCTAAGCGACCGTCTCTTCGTGGCTGATGTAGGCAAGATGGCGCAGATCCACGCCGCCAAGTCTGGACAGCCGACGTACGTGTACCGGTACGCATTCCGAGGCACTTCCAGTCTGTCCAACTTGATGGCACACAACGAAGAAAATTACGGTGTGAGCCACGGGGACGACGTGCTGAGCATCTTCAGCTACCCTGGAATGGTTCACAATGAAAAGGATTCAGCCATGATTGAAGCGCTCATTGACATGATCTACAGCTACTCCACAACCGGAACTCCCAAGTTAATCAACGGTGGACCTGTATGGGAACCTGTGAAGCCTGGCTCCCCTGAACTAAACTACTTGGATATCCTGTCCCCTACGCAGATGGAAATGAAGTCTTCATCAGATTTTGGACAGAAAGCCTTCTGGGACAGTCTAGGTTTTAACGAGAATGAAAATTACCGCGAGTATCTTAGGGACGAACTTTAAAACCTATTTTAAGAATGTAAAAAGTTCGTATTTTTTTTTAGTATACATTTCAGAGATTTGTTAGCTTTTTCTATTAATTCAGTTGATGAATT

>gene5053

TTAGTATTCAGTAGCGTACACTGTCGCTCTCATCTATTGCTAACAATTTTTTATCTAGCTTATTCATTACCTGTTATGGAATAATCAGATTATAATTATAATACTGATATTATTGTACTTGAAGTAATTATGTCGACAGTATTAAGGATAGCCTTGTTGTGTGTGGTATTGGGTGAAGCGTTAGCGATGGTACAAGTGAGAGTGTCTGACGGTTTGTTAGAGGGAGAGGTTGTACACAACGAGTATGGCGGTACTTACTTTAGTTTTTTGGGAATCCCGTACGCACAGCCTCCACTCGGAGATCTGAGGTTTAAGGCTCCCCAACCACCAAAGCCATGGGATGGGGTCCGCAGCGCCAAAGAATTTGGACCGAAATGCTTCCAATACGATTTGTTCGTGGATAAAGGGCATGTGTCAGGAAGTGAAGATTGTTTGTACTTAAATGTGTACACACCTGACGTCACACCTGTGAAACCGCTTCCAGTAATGGTCTGGATTCATGGAGGAGGCCTAGTATCTGGTGCCGGCGATGACATAGAATACGGTCCCAAGTTCCTAGTAAGACAAGATGTAATCCTTGTTACATTAAATTATCGTCTTGAAGTACTTGGGTTCTTATGTTTAGACACTGAAGATGCGCCTGGAAACGCTGGTATGAAGGATCAGGTAGCAGCCCTCAGGTGGGTGAAGAAAAATATTGCCAGCTTCGGAGGTGACCCAGACAATGTAACTATATTCGGTGAGAGCGCTGGTGGGGTCAGTGTCACATATCACTTGATGTCACCGATGTCCAAGGGACTATTTCAGAGAGCAATAGCTCAGAGTGGTGTCAGTGTCTCGTACTGGGCACAAGCGTATAAACCCCGTGAAAGAGGTTTTGCGTTGGCGAGAAAGTTAGGGTTCTATTCAGATGATGTTAAAGAAGTTTACGAATTCTTAAAACAACAACCTATAGAGAATTTAATAAAAGCCAAAGTACCTATCACTTATTCTGAAAAAGAACGAACAAATGTCGAAGTATACTTTAGTGTGGTTGAAGAAAAACAGTTTGGAGACAACGAGCGATTCTTCTACGGAGACATGGTCGATGCAGTGTCGAATGGTATTCACGAAGGAGTTGACATTATGACAGGATACACGGCGGATGAAGGTATAATGGGCGTAGCTATCTTTGGCGATTACAAAGAAAGTTTAGAACAAGCCAAAAACTTCCCACAATTTTTCGTGTCATACCCAATGTCGCTCTCATTATCGACTAATGACCAATTGGAGCTTGGTAACAGGTTAAAGGAATACTATTTCAAAGAACAGATTCGGGTACCAGACCACTGGGAGAGTCTAAAAAACTTTTACTCAATGGATATATTTGTATTTCCAACCATGAGGTGGATTAAGCTGAGTGCGCGTTCCAAGAAGAACAAGATATATTTGTACAAATTCACTTGTTACACAGAACTAAACATTGTATCCGAGTTGATGGGAGTAGGTCATCTTGTGGGAAGTAAACCAGTTGTCGCTCATTCAGATGATTTAATGTATTTGTTTAGTGCCAGAAACCAGACAAAACTGGACATGAATTCTGAAGAATTCAAACAAATTGAGATAGTGACAAAGCTTTGGACAAATTTTGCTAAATATGGCAATCCCACTCCTGACGACTCTTTTGGCGTAGCATGGGCTCCATACTCCTTAGAACACCAGGATTATCTGGATATCGGCAATGAACTGAAAGCTGGACAGGCACCAGACGACGAAGAAATCAAATTTTGGGAAAATCTATTGACAGAATTTGGACAGGAATTGTATTAAATATGGTTGCTAGTGTTGGTAATAAATGGTAAAAAAGCAAATGTTTA

>gene7097

CAGTGGTATAGAAACCATGTTAAAAATTTTGACTCTTTGTGCTATCCTAGCGCTGGCTTTAGGTCAGCAAGGACCTTCAGTAGAACTAGATGGTCAAGGCACGGTGATAGGTTCATTGTCAACTGAAGGAAATTACTTCGAGTTCCACGGTATACCATACGCGGACGCAACGTCGGGTATCAACCGATTCAAGGCACCACTACCGCCACCTTCATTCCAAACCCCATTAACTGCAAACCGAAAAGACATAAAATGTGTTCGAGCTTTAGGTGTTGGATATGAAGGCACCGAAGATTGCCTGGTAGTTGACATTTATACTCCAACTATAGATAATAATAAGAAACTGCCTGTTATGGTATGGGTTAAAGGAAAAGAATTCGACCGAATTAATAATCCAGAACTATCTTTCCGCAATTTTATTGAAAAGGAGGTTGTTGTAGTATCATTGAATTACCGTGAATCTGTTTTAGGATTCCTATGTCTTGGTACTGAAACCGCACCCGGTAATGCTGGATTAAAAGATATTATTGCAGGATTGAAATGGATCCAGAAAAATATTGCAAGGTTTGGAGGTGATCCAGGCTCAGTTACCATCGTTGGCCATGGAAGTGGTGGAGCTGCCGTTGATTTAGTCACAATGTCTCCAATGGGCAATGGTTTGGTACATAAAGTAATCGCTCAAAGTGGTAACGCATTTTCTCCTTGGGCTGTATCTCGTGATAACTTGAAACATGCCATCGATGTAGCTGAAGGTCTTGGTCATACAATAACCAATATTGAACAGTTATCTGAGGTTTTCACCAGAACTAGTGTCGGAGCTTTGATGGCTGTTATCAATGAACTTGACTTAACTGATAACTCTTTAGCCTTTGCTCCTTGTGTGGAACGTAAAGAATTGGAAGGAGTAGAACCCTTTCTTATAAAAACTCCTGCTGAAGTTGTACATAACAACGAATTTTTAGATATCCCTACCATTATTGGATTTGTAGATTACGAAGGTACTATTCGATCAAAAGAAGCAATAGGTAACGATTGGCTAAACCGAATGGATGAATCGTTCAGTGAATTTATACAGCCCGATCTAAAAATTGAAGATGACCAAGTATCAGTAGCGGATGAAATTAAAAGGTATTATTTCGGGTCAACTACACCCAATTTATATGAACCGTACCTGAGATATCATGGTGATACAATGATTCTCATATCTTCTTTAAGAGAAGTTAGAAATCGAGTATTTGCCACTGAATCACAACAGACCTATTTATATCAGTTTTCATATAAAGGAACTCTCGGAGCAGCATTTATTGGACCCATTAACGTTGATTCAGCACCACACGGTGTAGAAGTGGCTTACTTGTTCAATGGAGGTTCTGAAGCAAATGACCTAGATCGTTTGATTACCAATATCCTCGTTGACAGATGGACTAACTTTGCTAAGAATGGTATCCCTTCTACTGAGCATTCTAATGTAGTATGGCAGCATTATGCTCGACTCAATCCCCATTTCCTGCGTATAGGAACTGTAGACGAAATAGATGGTGAGCGTCAAGGACCTGTAGAAGTAGCGTTAAGGAATCCACATCCACAAACAGCTTCATTTTGGGAACGTATTTACACATCGCATTTCTTAGATGCACAAAGCAATTGGGAAATTATTGACAGGAATGAAGATACTCCTACAACAACCCTGAGCCCAGGTGGTATCGATGTGTGTGATGGGGACGGGGATGACTGTGGTACTGACAATGGCAGTGGTAATGACAATGGCAGTGGTGATGGTAACGATGACTGTGAAGGTGATGACTGCGATGACGGTAATTCAGCCTCAACTGCTGTTGGCTACACTTTCTTAATAATCAGTCTATTTTCAATTTTGAACCATTTCCATTCCTCTCAGATTTTGTCATAGAAACAATTCAATAGATCTAATTCTATTGTTTTTGTTTTCTCAACATACTGTTTATGCATTGACGTCTATGTATGTGTTAAGGCTATGGTCTTGTCCATTTATTTTGAAGACACTAAAATATGAACAAAGCATAAGGCTCCAATTTTATTCTTACCTTTCATTTCTTATAAAAATTAACCAATGTGTTACAGTTTTAAGTAGGTGTAAGTATTTCACAACCTTCTTTGGATTTTGCATTGAAATTCATTCGAAGAACGCTATGTACAGTTTACCTTCTTCTTTTTCTTGTAGTATTAATGTATTGTTAATCAGTTTAAGATAAGTTGGTATTATATCTTAGTAGATGGTTATTTGTTTTAAGATATATTACTGCACTATGGAGTGTAATTTTAATAATATTCTGTTTTAATAACAATTAGAAAACTACCAAGGATACTAATTAGCTAAAACTTGTGTCATTTCTGTATGAATTTTGAACTACACTTTTTTCTTACTGAGTCATTAGATTTTGAACAGACATTTTAATCAACTTTATAATATGAATGCAACCGATTTTTAATGTAATCAAGTTCAATCAGTTTGATTGAGTAGTGGTAGCTGCATCGATGTTATTTCATAAACTTTTAATTTAATATCATCCTTACGTCTACGAAATACAATTACAAATTCATTGCCTCAATAGTTATTTGGAAAGTTGTTAAATTGTTCTGTTTTTTTACTAAGCACATTATGTACCAAATGAACTTTGCTATCATACGTTTGTATGAAAGTTAAGGTTCTGCTTTAAAATAGTTAAGTATTTTATTTTTAATTTTGTAAATACAATAAAAAACCTAAAAACAA

>gene7303

TATTTCAAAAGACATTATGTTCAATTTGTTACGTGTTCAAGTCTGGTGTACATTAGGCTTAAGTATTGTTTTAACCCAAGAACCCGTGACTAATCTCCCACAAGGCCGTATTGTTGGGATAAAAGCTTACATAGAAGGGTCCAACACGCCAATAGAAATATACTATGGTGTTCCGTATGCAACGCCCCCAAAAGGAAGATACAGATTTTCGGCTCCAGAACGGCACACGGGTTGGAGACGAACATTTTTTGCCCATAGGATACCTCCGCATTGCCCCCATATTGGAGACGACGACAAAGACAATTACAGCGAAGATTGTCTTTATTTAAACATTTGGACACCTCGGCGCTCAGATGGTAAAACTTTACCAGTGGTTGTGATATTATTCAGTGAATCGTGGATAAAAGGTGGTGTATCTCTTCCTTGCCAAGAATTAGCAGCCGAGGGGATAGTGGTAGTCACAGTGATGTATCGATTACACCTACTTGCATTTTTTACTTTGCGCACTATAGCTGCTAGGGGTAATCTTGCATTGTTGGATCAATACTTGGCAATGCTCTGGATTCGTGACAACATTGCAGCATTTGGTGGAGATCCTACTGCAATCACTTTACTTGGACACTCCGCCGGTGCTGATAGTGTACTGCATCATATTACTTCACCACGATCTCTTGGTTTATTCCGAAGAGCAATCATTATGTCACCGTTTGATATTTGGAAAATAATTGGCGAAAAGGTTGTTGAAGTTACTCAGGTTGAACGAATGTCTCGTGAAATTGCAAAGGCACTCGGCTGTAACGGTGACACAGATCACGAAATTCTCCATTGTATGCGAGAGCGCCCTTTGTCGGAATTAATGTCATTATATTCGAACGATTCCTGGAATAGATTTATGCAACCGATTTCTGACGATTTTTTACCAGAATCTGAACAATTTTTGCCGAATTCAATAATGTCTGGCTTGACAAATCCAGCAACCAAAATACAACTCGATGTTCTTCTAGGCGCGAATGATCTCGAGGCTTTAAATAATAATGTTATGAAATATGAAGAATTAATGAAGCAAGGAAACCTTTACGCGAAAAACAAAGTAATAATCGAAAGTCTTCGTTTGTTCTCTTTGGATCGATCGGAAATGTTACCCCTGTTAGTGGAAGCCGTACGTTGGGAATATTGGAACAATAAAACAAGAAATGTAAAAGAGGTACTTGGTAATGTGGAATTTCTGGGGCGAGTTGAATCTGCAGCAAAATGGAACTCGGGTATCGTACTGATTGCAGCTCGTTTGGCGAAGAGAGTTAGGCGCCTCTTTGTTTATAGATACTCACAACCAGCTGGAGTTGATCTAAAAGGACAACAGTACAATTTTACTGGAGCAGTCCACGGATCCGATTTAGTGTCTCTATTAGGAGATGCATTAATGCTCCAGGTAGCCCGACGACCCCCTACAAAAGAAGAAAAGAGAGTTTCGTTTCTCTTACGAAGACATTTAATTAACTTCATTCAATTTGGTTCACCTGGAGAAGAATCAATCTGGCAACCCTACAAATCATCGGACGCCAACGTATATGATATCCACGATACGAAAAATTCCTATCCTTACTACCATAGTGCTGAAAGAGATGTCAGATTTTGGCTTCAGTATTTGCCACAACTGAACATTATTTTGGATACTGCAGAGAAAACAGGAAAACTGACAGACGAGAAAGATGAAAATCGACTCAGAGGCGGTGTTCTTGCTATGTGTGGTGTGACAATAGTACTTTTGCTACTTCTCGTTATTTGTGGTATAATATTACATAGACAAAAGTCACGTAGATTTACCGTCGTTGATGAGAATCATCATTAAGCAACGTGCAAGCTGGTACCATATTATTATGGTAGTTGCGCATTAAAATGAGTTTGTTGTTGCTCGATTTTGGTACCTACATATCTACAAAACATACACAATATTATAATCGTACTAATATTATAAATGTGAATGATTGTGAGTTTATTTATTTTTTAATATGGGATGTTTTAGTCTGTTGCTGAATCACGTCAAAACGGCTGTAGAGCCTTCTTATTATTCGGGGCCTTACTATTGGAATAGTTAAAAATTGTATATAAATAGAGGTATATATATATATATATCTAACATTGCTTTAATAATTGCAGATTTGTAAAAATCAAAAAAATAAAACTTGTTAAAATCCATGTAATATGTCCCCAGACGTAGGTAGATCTATTTTTCGGATCGGACATAGGGCCTTGGCGCTTGCAGTATATTTTTGACCATAAAAATGTGAATAATAAAATCTTTTTGTCCTTGTAAAATATCTGATTATTTTACTTTTGTTCATCAGCGCGGTTTATCAGCAGTTTATCAACTCTTGACTGCAACGGTAGTTGCTCAATGTTTATAAAAAAATTTAGTGGTTTATAAATAAACACGTTGAAAAAAGGCAGACGCAATGTTTATAAAGATTATAAAAATATATAATACTTAAAGTTTTTTTTAAACTTACAGACGTGATATACTATTTTGAAATCATCTGGATGACCTTTTTAATCTGATACCCATATTGCAATATTTGAAGAATTTGACATTTTTTTAGGTAGGTTACTTTAGCGTCTGACAGTTAGCATGTTTGTTTAACACATAAACTCGTGTTTCCCGGTTTCCAAGAATTAAATAATCAACAAAAACTATTTTTTATTAATGTTGTAATATGTTTATTGATAAGAATTTAGGATATATAAATAAATATATACAAAACTAAATTTGGTAAACAATTCCAGAAATATTATAAATGCAAAATATTTCCAATTATACAATTCACCCTGCATTGTATGGACGACGTCGTATTGGGTATCGTGACACGTCACGACGTCGCGACGTTTTACGAAGCTCGTCGTAAATTACCACAACGCGATGTCGTATCGTGTTTATGTGTACCGGCCCTTGGACTTGGGTT

>gene8011

ACACGAACACCCACCAATACCGGTACGATCGAACCACGTAACAACAAAACTAAAAAACAAGCAAAACAACATAACGAAACTAATAAACTAAAATAAAAAGTGACTAAAACATAACCGTCAATTAAAAAATTTCAAATATCGAAATGAACGTCCATTTTAAGTTTAACGTATTCGAACACCGGTTTCGTTTTTTAAAAGAACGAATTACATGTTTTAATTAAACCGTGCTGCCAGTTTGCTTGCTCCATTAGTTTTAATAAGTGTTTAAAAATGATCCGTGTGCTTATATTGTTATTGCAAATGACTCTAATTTTATCGGCGAGAGAACCAAAGCAAATAAACTTAGTGAATCAAGGGACGATTTCGGGGATGTACATCACGAGGTTTCGGACAAAAAGAATAGCAGCATACGTTGGAATACCGTATGCTCAACCTCCAATTGAGTTCAGAAGGTTTTTGCCCCCGGAATATACTGATTTACCTCAATGGGAAGGTGTGAGAAACGCGACCATATACGCCCCGGATTGCATGCAGAGTGACCCGAAAAAGGAGGATGTGCAAAACCCTTTGAAGAAACATGATGATCTGTTCATGAAGCTCTTGGATTCTCAAATGGAAGAAAAACGAAAGAAGGAATATTCCGAGGATTGTCTCTTCTTGAACGTGTATGTACCGGACGACTTCAAAGTCGAGGGCTACCCAGTCTTCGTTTGGTTCCACGGCGGTGATTTCGTGCGAGGCAGCCCTAACAGTGTGAACCCCTTCCAATTGGTGATAAAACAAAGGGTAATTTTCGTATCAGTGGCCTACCGGCTAAACATCTTTGGATTCTTCTCAACCCTCGACAATGAAGCTTCAGGCAACTTTGGTCTACACGACCAAGTAGCATCTTTACATTGGGTCAAGAACAATATTGAAAGTTTTGGAGGTGATCCCGAAAACGTTTGCATCATTGGTCATGATGCTGGAGCAGTCAGCGTCACCCTTCATTTAATATCCTCGTACTCAGCAGGACTCTTCCATAAAGCTATAGCAATGAGTGGAAATGCTTTATCACCAGAATCTGTAAACATAGCAAGGAAAGAAATAGTTACTGTCGATAAGGTTGCAGTTGCATTCAGTTGCTTTAGAAAACCAACTTACCAATTACTAGATTGTCTTAGAAGAGTTCCAGATAAAGCACTGTTAGATATAGCTGGGCCAGTAGCTGAATGGAAACCCATAGTGGATGGAGGTTTTAGTAATATAACCAGTCCGTTTTTGCCAGAGCTACCATCAAAACTGTTCAAAGATGAGATTTTCTCCCCAGTCCCTCTTTTAGCAGGCTACACGAACATGGAAGATGGACTTTTGCTAGAAAAAGATGAAAGCGGAGATTCAGGTATTAGTCAAAGAGAGTTTGATTCAATGAGAGAAGAAATTATCCTCGCAGATATCACTGTAGACAACAGCTCGTGTTTCACTAATCAGCATCACATACAAGACGCTGTAGCATTTTTCTACAAACCGATACCTCATACGACAAATGAAACAGTATTGAGAAAGCTTTTCGTGGATTTTTATACCGACAAAACCCATGGAGCTACTACTTATCAGTTAGCGAGGCACATTTGTCCACATGCTCCAGTATATCTCTACAGATTTGACTTAAAACCTTTTTCTGATATTGCAAATGAAGGTCTTCCAGAATGGATTGGTGTTCCACATAATTTCGACCTTATTTACACCTTTGGCATGCCCTATCTAGCCTTACCAGAAGACCTAAGCAAATGGGACTACAGAGACAAAAGCATTTCAGAAATCATAATGAGGATGTGGTCCAATTTCGCCTGGTACTCAAATCCTACGAATTCAGGAGTAATTATAGATTGGCAGACTTATGAACTGGAGAGGCCTGGGTACTTGATCATTGATAGAGCCAATTTCACGATGAGCACTCCAGCAACAATTAATTACAAAGCTTTTGAATTCTGGACGGATTTTTATCCGAAAGTTGTTGAAATTGGGACGAAGTGTTGTAAAGAAATCACAGACGATTCTGGCACGATATCAGTATTTCCATCCAAAATAGTTCATAGTATATTAGTATTGTATTGTAGTTTAATTTTACTGTCATAACGAAGGAAGGGATGTGAGAACACGGTTATTTTTGTATAGTCTATTGAAAGATGTATTTTAAGTACCAGTATTTCTTTAATACCAAATGAAATATTTACCTTCCAGAGAATTAT

>gene8407

GTTAACAAGTTCCCGCGTATGTACCTGTATCTAGCTAGTCTGTTATTTAGTTTATGTGTTCATGGCGTAATGTCGTGCGTGATTATTTGAATTTAATCGTTTTTCCGCCGGCATCCATTGAACGAATTTAATAATAATAACAACGAACAAGTAACAAATAAAGGATTTATAACTTGTATCGTACGAAACAATGTGTTCAACAGTGTTACCGACAACAATAAAAATGGTGGATATACCGGAGGAAGCAAAATGCATAGTCCAAACAGCAGACGGACCCATTTGTGGATATTGTGAAAAGACTGACGAAGGTATTTGTTACAAGTTTAAAAGTATACCTTATGCTAAACCACCACTAGGCCACTTGAGATTCCTGCCCCCATCTCCAATACCACCGTGGACAGAAGTATTAGACTGTACGAAAGATGCTCCGAACCCTGTCTGCCGGTCAGTTTACGATATTATTAGTGGATCAGAAGACTGCCTATACATTGAGCTCTCTACTCCTAGTATAAAGCCTGACAAACCAATACCAGTAATGTTCTGGATAGGAGGATACGGCTACTCATGTGTCTTAGACCCCATCTTAGATGCCACTTTATTCAATTATCAAAATGTTATATTCGTAAGGTGTGGCCATAGAACGGGTCCGTTTGGATACCTCTCCATAAATGACTACGCTGCACCAGGAAATTGCGGACTCAAGGATGTAGTAATGGCCCTAAAATGGGTTCAACGAAATATAAGTACCGTTGGCGGTGATCCAAACAATGTCACAATTTTCGGAAGCTCTTCAGGTGGAGCTATGGTCAACTTTATGATGTTATCGCCAATGGCCACCGGATTGTTTCACAAACTTATCATACAGAGTGCCAGTGTTTTGAATAACTGGTCTTTAGCAAAGAACCCATCACAAGGTGTTATAGAACTGGCAAAAATACTCGGTATAGAGAAGACATCTAAGCAGGAAATCGTTGAGGAGTTACGAAGGATCCCTGCAGTAGATATTGCTGAAGCTTTCAAAAACTTCCACATTGGTTTTAGCGAAGGTGTCGAAAGTGATTTATTTGATTCTGTTTTCAAACCCTGCATTGAAGTAGACCTAGAAGGACAAGCTGCATTTATCACAAAGAGTCCAATAGTCATTCTGAAATCTGGTAATTTTAATAAAGTACCGTGTATCATCGGAAGTAATAATATAGAGGGTGCAGTTTTACAATACGTTGTAGAAAATTTTTGTTCAAATTTTGAAAAATATAACGAGAATATCAAATTGCTCGTGCCTCGAGAACTAGCGAGAAGAGACAGAATGTCTGACAATATAGGGCATCAGATATTGAAGTTTTATTTGGACGGAGAAGAACATTTGAGTGAGAAAAATAGGACTCAATATTTGCAATTGATCAGTGATTATTATTTTTCATATTATGTTAATAAGACTGTGAAATTACTGTGTGAATTTGCACCTTCGTATCCCGTATACTACTACATACTGAACTATGCCGGAGAATGGGCAGTGCCTGAGAAGTTCAATTTTTTCAACTCTACTGGGCATGGTGCGGAGTTACCATTCCTGTTTCGTATAAAAATGCCAGATGTCTGTAAAGGCAGTCCAGATTCTATAACTACTAGGACAAGAGTGATCAAAATGTGGACTAACTTCGCTAAAACAGGAAACCCAACACCAGATCCAGATGACCCACTGCTCTGGATAACTTGGGACCCAGTGGAAAGCAAAGAAAAGCTGAATTATCTCAGTATAGGACAAGAACTGACGAAGGGTAGGAATCCCTTTCAAGAACGAATGAAATTTTGGGATAAACTGCACAAAGAACACATGTTTTTAAGGGCTTTAGTGCATTTTAATGATTCGGGATATTCAGTATAAAACCTTCACTAGCCTGTAACTGTCTTTATGTGCAAAGGCCTCTTCTTGAACGGAGGAGGAATGAGCATTAGTCATCACGCTTGCTTAAGGTGGATTTGCGATATCAAAGTCATTGGAAATTGTACGGTTTTTCTCAAGAAAGAAGAAAGATTGCCAGTTTTTGGCCTCGTACTCTCATTTAAGAAGCAGACACCGAAGGATATTTCCTTACACTATTGTGTTAGGTCTATGTTTGGAAATTTTATTACGATCTGAAATTTTAATTGCGTCCAATATACAATGTAAACACGATGTTGATTTTTATTTGCGCTAACATCATTTTA

>gene9365

GATCTCAATTCATTTAATTAAATATGGCAACATTTTGAAGAAGCGCTAATGATAAGAATATTGTACCATTCTTATCACTTATCACAATAAATGATTGTTCGAGATTTCAACTCACTTGTTAATGGGTATATTTATAGAATAGATCATTTTATCATTCGTCAGTAGAGGTTTGCCTTACTAGTGCAATTCAAGTGCCTTATTGTGCAAAACTTAAGAGAAAATCATAAGTTTTTAACTGTTCAAAAGTAAACTTACAAGGTGACGAAATTTCGGAACAAGTTAGAGCAACCAAAAGACAGAAGATGTGTTCAGTACCAGAAGATGTGAAATGTATTGTTGAAACAAAAGATGGCCCTATATGTGGTTATATTGATAAAAACGATGAAGGAACTTATTATAAATTTAGTAGTATCCCATATGCTAAACCACCCCTTGGTCGCTTGAGATTTATGCCTCCGTCTCCCATCGAACCATGGCAAGAACTACGAGATTGTACTACAAAACAACCCCTTCAGCTATGCTGTGTCTTAAATCGAAGTATAGAAGGTTCAGAAGATTGCCTCTATATTGAAATCTCAACTCCTAGTATACAGCCTGAAACACCTATGCCAGTTATGTTTTGGATTGGAAGTTTTGGATTTACAGGCACTATGGATCAACTATTTGATGCTTCCCTAATCAATAATGAAAACGTCGTATTTGTAAGGTGCGGCTTCCGATTAGGGCCATTTGGCTTCCTTTCTATCAATGACTTTACAGCACCAGGTAATTGTGGGTTAAAGGATATAGTCATGGCTTTAAAATGGGTGCAAAGAAATATATCTGTATTTGGAGGTGATCCAGACAACGTCACCATTTTTGGAAGTTCTTCAGGAGGAGCAATGGTCCATCTCATGATGTTATCCCCAATGGCTACTGGTCTTTTCCACAGAGCAATTATACAAAGCGCAAGTGCACTGAACAATTGGTCTTTAGCCAAGAACCCCTCCCTAGCTGTCATAGAACTAGCAAATGAATTGAACATCCAGAAAACGACTAAAGTAGAAATTATTGAAGAGCTACGGACTTTACCTGCGTTAGATATAATGCAAGCATTTCATAACATGGCACTAAGAACACAAAAGGCTGCCAATCATGATATCATTGACGCAATCTTCAAACCGTGTATCGAAGTTGACTTCGAAGGTCAACCTGCATTTCTTACGAAAAGCCCTCTTCTAATTCTGAAATCTGGAAACTTCAACAAAGTGCCTTTAATCATTGGTAGTAATAATATTGAAGCTGCATTATTGGAATTCATTAAAAAAGATTTTTATTCTGATTATGAGAAATTTAACGAGAATACTAGTCTTATTGTTCCTAGATCGATCGCCAGAGAAGACAAAGTAACAAAAAGTATTGGTCAACAATTACTTAAGTTCTATTTGGGTGGTGAAGAACACCTAACGGCAGAGACTAGAACACAGTACTTACAATTAATAAGTGATTACTATTTCTTATATTATGTCAATAGAACTGTAAGAATGCATAGTCAGTCGGCACCGGAATCCCCAGTGTACTATTATATTGTCAACTATGCTGGCGAGTGGTCAGTACCCACAGACATCAATTTTTTGAACTCTCCAGGGCATTGTGCTGAGCTTCCTTTTATATTCCGTATTAAAGTGCCAGAAATTTGCAAGGGTAGTCGAGATTCGGTGATCACACGAAGCAGAGTAATCAAATTGTGGACAAATTTCGCCAAAACAGGCAATCCAACACCTGATGAAGATGACCCACTTTTACAAATAACTTGGGATCCCGTTGAAAATATGGATAAATTAAACTACTTGAGCATAGGATCAGAACTTACTAAAGGCAGAAATCCATTTTATGAGAGAATGAAGTTTTGGGACTCTCTTCATCAAGAACATGCGTTTCTTAGAACACTCGTTTATTTTAATGATATAGGCATTTCATGGTAGACAATACAACTACAATTTGTAGCTATATTTAGACTCATTCAAACCTCCATAATATAAGAAAAATTATATTATTTGGCCTTCGGTCTTATTGTAACTGTAGGTTATATTTTTGTAAGTAATTTTACACTTTACTTATACAAATAAATATTTTAGTTGTTTTATTGTTTGACAAAAGTTACTCTTTTTATACCAAAGCGAAATTCCCATATCTTTTAATACACAACTTAGCTTCCTCTTGAGTTGGATAAGAACCACAAAAGAAAGGAACTTAGGATTGAAAATTAAATGACATGTTTGTTGGTACCAATGTGTAATATAGCATGAAATGATACTTTCACATAACAATATACAAGTTTAATTATTTTAGTAACAAAAGCATCAAGGCCAACAAATACAATACATACTACAAAATAAATGAACTAGAT

>gene9804

CACAGACAGGATTCAACAAAAATTATCATCGGTTGTTGGGTAGGTATTTTGCAACCCGGTATGACGATTATCACATGTCGTGGGATGTCTAGTTCCGATTGCGAGTGTCATGGGCTTAGTGATGTATCGGCTTTGGATGTGGTGTGTTGCGCTTGCGTTGGCGGCGCTATGGTTTGCGCCAGCACAGGCTGTCGTCGGAGGCGCCCCAGCCTCACCTCCCGAACCTGACGCTGCGGTTGTCTTCACACAGCGGCACGGGTTCAGCGCACGTATCGAAGGAATTAAAGATGATAAACTTGGATATTATAGCTTTTATGGCATCAGATATGCGGAACCACCATTGGGTCCTCAAAGATTCCAGCGGCCCATTCGTCGGTTCCTCGCTGGAGAGATGATGGCCAACCGACACTGCTATCCATGCCCACAACCAGATCCTTACCAGCGAAATCGGATCATAGGCCATGAAGATTGCTTGTGTCTGAACGTGTATGCTCCCAAAATGCCTGCTACTGAAGAAGGAAGCCCAGTTGTCTTCTTCATTCATGGTGGTAACTACAGAACAGGATCAACGGCATCGTATGGCGGTCAACATCTAGCCCAGAAGGACACGATATTAGTGACAGCTCAATATCGACTGGGATCATTAGGATATCTGAGCACGGGACAGAGGGATGCTGGAGGAAATCTTGGGCTTTTTGACCTGCACACTGCCATGGTCTGGATCCAAGATTACATTCAATTCTTCGGTGGCGATCCAAAACGAGTGGTTGTTATGGGCCAAGGTTCTGGTGGTAGTGCGGCTTCTCTCATGGCAATGTCGCCTGAAGGTCGCAGTGCTACAGGTGTGGCTGCCTTGTCTGGAGCCCCGTTGTCTCCAGGCACGGTGAGACCCGATCCGGCCAAACACGCTGAAGCTGTAGCTGAACGCACAGGGTGCCCAAAGAAACCAGCTGAAAGTCTACTCATATGTTTGAGGCAGCTACCCGTTGAGAAACTTGTGCAGGCCGACGAGGATATAAATATGGATATGGTGGATACTCAACGATTTTTGGAAGAGATCTCTGGACGTTCAGGTGCTGGTGCCAGAGTGGAAGGTGCGGATGACCTCCGAGGTCTTCCACCAATAGTAGCCGAGTCTCCAGCTGAGGCTCTCAACAAGAAACAGAAGCGTGTCCCACTGCTCACTGGCGTTACTTCAGCAGAGACCTCAAGAGCTGTATTTGGAAAATTCAATAAGTTCTTAACGAACCAACTTGAAACTGTGAAAGACTTTTTGAAGAAGGACTTGATCGGTGGTCTGCAAAACGTGGTTCATGGTGTAGAGGGTCTGATTCCTCTGGCTTCCAACGTGCAAACCGTGTTGCCTGTAACTGACTACTACGAGGGGTTGCTCAAACAGGCTACCAGTATCGTTGATAGACTCTCTGAAGTTGCTGAAGCCACAGGTGACGCGCTGTTTAACTTCCCCGCTTACCAAAGTGTTCAGAAGTGGAGCTCAGGGGGTCATGCCTTCCTCTATAGCTTCGAACATGTTGGGAACCTCTCGAAAGGATGGCATTTCTTGCCTGGAGTGGCTCTTGCAGTTAAAATAAAAAAACACATTACAGAGAAAGATGACAGTGAAATGGTTAAGCCGAAAAAAGCGCAAGGTCCATCTCATGGTGATGAACTAGCTTACATATTCGAGCCTTTGGGTCCTGACGGCAAGTCTATGGGAGATGAAGTATCCAGCACCGATGCTAGAGTGCGTGATAACTTCGTGGGACTTATCGCCAAATTCGCTCACGGATCTGATAAAACTGATGCCAATAACAACACCCTGTTCGGTTTTGCTGCTAACTTATTACCTAGAAGTAAAGACCAATTTTTGAAAATTGGCGAATCTTTGACAATTGATAAAGATTTCAGATTCTGCCAAATCGGTTTATGGGGTAATATGGCAGACCGACTAACAGGGGCTCTCTGTAAGAATTTACTTGGGGACTTGTTAAACCTGTCGCAGTTGCCCATCGACTTGAAAATACCGCAGCAATTGGGTGGACAGAACAATGGGCCCTTAGGTGGCCTCGGTCTTTTGGGCAACACGGGTACTAGGGCGCCTCAAATACAGAAGCAACCACTGAGAACCACAAAGGCCCCCTTTAGACTACCATTTGATTTTTAGATTCAACAAATAATGTCTTAAGCATACTCCATTAGTAACTTGATTGTTAAACGTTCTTTGTTATACTTACCTATTCAATTTGTGTAAATATTAACATTTTGTACAAGTGTTCACGGTGTACTAGCCTATAGCCATCCTCGATAAATGCGCTATCCAACACAAAAAGAATTATTCAAATCAGACTTGTAGTTACGGATA
