# Supplementary material for: Identification of transcriptome and fluralaner responsive genes in the common cutworm Spodoptera litura Fabricius, based on RNA-seq
Source: BMC Genomics. 2020 Feb 3;21:120. doi: 10.1186/s12864-020-6533-0 (PMC6998375; doi:10.1186/s12864-020-6533-0)
Supplement: Supplementary file 9 — Additional file 9. Nucleotide sequences of annotated insecticide-targeted genes of the S. litura transcriptome. [file 12864_2020_6533_MOESM9_ESM.docx]

**Additional file 9**: Nucleotide sequences of insecticide-targeted genes of the *S. litura* transcriptome

>gene11828

ACGATCCAACAACAATGCTGATCAATTACCTCATCTTGTTAACCATCAGCATTTTACAAGTTGACGCGAAAGGAGCAATCTACTTGACGACTAAAAATGGAACGTTTCCAATATCGCTGACATCCCTGAAGTCCCGAGACGACGCTACATCGCAACACACTCCAAGCATTTTGTCTGCGACCGCTAAAAATCTCTCACACAATACCTCGGAGACGGAACATGACTACTGGGATTCCTCAGAAATGCTGCTCTCCAATTACATAAACGGCACATACGACGAACTCGATGCCTTCACCTTGAAACAAAACGCTAAGCGAAGCATTAATGATGCAGTCTCCAAAAACATCACTATGGTCCTCGAAAATCTACTGAAGAATTACGAAAATTCACAGTTGCCTACACATGGGAAAGGTTATCCTACTGTCGTACAGACTAATATTCTTATACGGAGTATGGGACCTGTGTCTGAACTAGATATGGATTATTCAATGGACTGCTACTTCAGGCAGTATTGGCGTGACACCCGGCTGTCTTTCCTGGGTCCCATCCGCTCCCTGTCACTTTCTATCAAGATGCTCGAAAGGATTTGGCGGCCAGACACCTACTTCTATAACGGAAAACACTCCTATGTGCACACTATTACTGTCCCAAATAAGTTGTTGAGGATTAGTCAGCACGGAGATATTTTATACTCCATGAGGTTAACGATAAAAGCCAAATGTCCGATGGAACTCCGAAATTTTCCGATGGACCGACAATCTTGTCCATTGATTCTTGGTAGCTATGCATACTCAAACCAGCAGTTGGTGTACCAGTGGCAAAACTCTCAGAGCGTCAACTTTGTCCCGGGCATGACCCTCTCACAGTTCGACCTCATCAGCTTCCCTTATAGAAACTTTACGTTCACTCGACGGGAAGGTGAGTTTTCCGTGCTTCAAGTATCGTTCAATCTTCAGCGCCATACAGGCTACTTCCTTATACAAGTGTACGTGCCATGCATACTCATCGTGGTCCTTTCCTGGGTGTCATTTTGGATTCATCGCGAGGCTACGTCGGATCGTGTTGGTCTTGGGATAACAACAGTGCTGACTCTTTCTACAATCAGTCTGGACTCACGCACTGATCTGCCCAAAGTGAGATACGCTACTGCTCTCGACTGGTTCCTACTGATGAGCTTCTTTTATTGCATTGCTACGCTCTTGGAATTTGCAGGCGTGCATTATTTTACCAAGGTTGGCTCCGGTGAGATTGTAATAGATGATGCAGAATGGGAAGAGCTGATAGAAGAAGTTGGAGGTGACGCTTTTGCAGCACGGCAACTAGCGGTGCGCCGGCGCAGTTCTGCTCGATCTACTACGAACTTCAGTTTCACGTTACCTGCTCAAAGTAACCAGGAGGAGAGTGGAGCCCCAGCCGAGCAGACCGCGGTCCGGCTGACCATGGAACGTACCACGCAGACGGAGAGACGTGTCCCACGCTGGCGACAACTACTCTATTGCCTCGCTGGAGACGACCGATACCGTAGACAGAGACAGGTGGAAGCTGGCAGCCGAGGCCACATAAACAGCGTGTCACACATCGACAGGGCTGCCCGCGTCATGTTCCCGGCATCGTTTGCTCTACTCAACTTGTTCTATTGGATGGTCTACGCGTTCTCAAGCGACGACTTTGCCTGGAGTGACAACCCTATGAATTCTTTGTCTCATTAGAAAAAAGTACCTATTTAGAAGAGGGCTTAGACTAAAATTGGTATACCTTGACTACATTGTTACCCATTATGTAGATACATGTAATGAACAATGTAAGAAATTCTCTTTTTTAAG

>gene11829

ATGAGCGCGCCCCGCTACACGCGCTGTCTGCACGCGCACGCGATTTTTCTGCTGTTGCTTCGAGTTCTACCGTTCACAGTGGCACAGAACGAGCGCACCGCTATCGACAGATTGGAAAACGTAACGCATACCGTCACACGAATACTTGACGGATACGACATTCGGTTGCGGCCTAATTTTGGTGGAGACCCGCTCTATGTTGGCATGGATCTAACTATTGCTAGTTTTGATGCAATATCTGAAGTGAATATGGATTACACAATAACCCTTTATCTGAATCAATACTGGAAGGATGAAAGGTTGGCTTTCGGTCTTCCGGATGAGGTATTGACGCTCTCGGGGGACTTCGCCGATAAGATTTGGGTGCCCGATACTTTTTTCGCTAATGACAAGAATAGTTTCCTGCACGATGTGACGGAGCGTAACAAGCTGGTACGCCTCGGCGGAGATGGCAGCATCACGTACGGGATGCGGTTTACCGCGACGCTCGCCTGCATGATGGACCTCCACTATTATCCCTTGGACAGCCAGAACTGCACCGTCGAGATTGAAAGCTATGGGTACACTGTATCGGATGTGGTGATGTACTGGAAGGAGACGCCGGTCAGAGGAGTAGAGGATGCGGAACTCCCACAATTCACTATACTTGGACACGAAACGAATGATAGAAAGGAGAAGTTGGCGACGGGCATCTACCAGCGTCTGTCGCTCAGTTTCAAACTGCGCAGGAACATTGGCTACTTCGTGTTCCAGACGTACCTGCCCAGTATTCTTATTGTGATGCTGTCTTGGGTATCGTTTTGGATTAACCATGAAGCTACGTCCGCTAGAGTTGCGTTAGGTATCACGACGGTACTTACGATGACTACTATAAGTACGGGAGTGCGTAGCAGTTTGCCTCGTATCTCCTATGTCAAAGCTATAGACATCTACCTAGTGATGTGCTTCGTGTTCGTATTTGCGGCGTTGCTCGAGTACGCTGCCGTCAACTACACGTACTGGGGCGCACGAGCACGGAAACGAGCCAAGTTAAAGAACAGAGACCAATTATCCACTAGCGCCAGTGTTGAGAAAGAATTGAAATGTTCAGGTTCCCGTTCCCCAGAAGAGATAATTGCGTTGCGAGAATGTGCAACGACTGCGGGCCGCGTGTCTCCTCTTTTAGGGCTACGGTCGAAACCACTACCAGCCGCTACTGGAGCCCCGCCGTCCCTACGACTACAACGCGACCACACTCAACTACGATACAGAACAAGACCACATTCTAGAAACTCTAGAAACGGATCTACGGGAAAACCTAAAGTGATGCACGCACTTCGTCGAGGCGCGACCGTCATAAAGGCGTCTATGCCAAAGATACGAGATGTGAATGTCATAGACACGTACTCCCGAGTCGTGTTCCCAGTCTGTTTCCTTCTCTTCAATGCCGTCTACTGGGTCTTTTATATTTTCGATTGAATTTTCCACAATGAAAAACATTATCCTCAATAGTGTTCTATGAAATTGTCACCCTACTCGTAATTTTACCTAACGAATTTATGCGATATGTATTTAACATTATTTTAC

>gene14064

TAATTAGGAGGTGAGTACGCGTCGCATCGGCTGCGGCGGCGCGACGGTCGATAGCTGGAGCGACAGGCGGTGCCGGCCCGGGTCACGCCTGCTGCGATCGATACCGGCGCGGCGGCGGGAGCATTCGAGCACTACGCTGCCACCGGACACGACAGCAGCCAGTCCGCCTCGCGACACCGGCGACGAGCGGCGAGCGAGCGAGCGCGGCCGCACATACACGCGCTAGCTCCCATGCGACTCTCGCACGCGTCATGAGCGGCGCCCGCCCCCGCTCCGCGCCGCTCCTGCTGGCGCTCGCGGCCGCCTTCCTACCGCAAGCCAACCATGTCGCGGGCGCCGGTGGGGGAGGGATGTTCGGTGATGTCAATATATCAGCTATTTTGGATTCATTTAGTATAAGTTACGACAAAAGAGTAAGACCAAATTATGGAGGACCACCCGTGGAAGTGGGGGTTACCATGTACGTGCTCTCCATCAGCTCTCTGTCTGAAGTGAAAATGGATTTCACATTGGATTTTTACTTCAGACAATTTTGGACCGACCCCCGATTAGCTTACAAAAAAAGAACTGGAGTTGAGACATTATCTGTGGGTTCGGAGTTCATAAAGAATATATGGGTGCCAGACACGTTTTTTGTAAACGAAAAGCAATCTTATTTCCATATAGCAACAACAAGCAACGAATTCATACGTATTCATTATTCTGGCTCTATCACTAGGAGTATCAGATTGACGATTACAGCTTCGTGCCCTATGAACCTGCAATACTTCCCAATGGATCGCCAGTTGTGTCACATTGAGATAGAAAGTTTCGGCTACACCATGCGGGACATCAGATACAAATGGAACGAGGGGCCCAACTCTGTGGGTGTTTCCAGCGAGGTGTCGCTGCCGCAGTTCAAGGTGCTGGGCCATCGCCAACGAGCTATGGAGATCTCCCTTACTACAGGAAATTACTCAAGATTGGCATGTGAAATTCAATTTGTCCGATCAATGGGATATTACCTGATTCAAATTTATATTCCGTCTGGTTTGATTGTCATCATATCATGGGTATCATTTTGGTTGAACCGGAATGCCACACCGGCTCGAGTGGCTCTAGGCGTTACCACTGTGTTGACGATGACAACGCTCATGTCGTCAACGAATGCGGCTTTACCTAAGATATCGTACGTCAAGTCTATCGACGTATATTTGGGAACCTGTTTCGTGATGGTCTTCGCTAGCTTGCTAGAATATGCTACTGTTGGATATATGGCCAAAAGAATACAGATGAGAAAACAAAGATTTGTAGCTATTCAAAAAATCGCCTCTGAAAAGAAAATCCCAGTTGATTGTCCCCCTGTCGGTGACCCACACACACTGTCTAAAATGGGAACTCTTAGCAGGTGCCCACCTGGAAGACCATCGGAGGTGCGATTTAAAGTACATGACCCCAAAGCTCATTCCAAAGGCGGCACTCTAGAGAATACCATCAACGGTGGCCGCAGTGGAGCAGAAGAAGAAAACCCAGGACCTCCTCCGCATATCTTACATCCTGGCAAGGACATAAGCAAATTGCTCGGCATGACTCCTTCGGACATCGACAAGTACTCTCGCATAGTATTCCCCGTCTGCTTCGTATGTTTTAACCTCATGTACTGGATCATATACCTTCACGTATCCGACGTCGTGGCTGACGATCTGGTTCTACTGGAAGAAGATAAATAGAGGGCGCTGTGTACATAATCCACTTATTTTCTCACATTGCAGTATTGAATAATAATTTGAAACAGTTTATTAAATACTCATATTATTCCCCTCTTTAGTAAAGGAAGAACATAATTGTATTTAGATTTTCAATGTACACGAAACTATCTCGTCGTTTTTCGAAATTGATTTGTAGATAATCTATACTTAATGTACTAAATGTAACGTGATAAATGTTGTATATACGTAAGACTCGGAAATATGAATTACGAACGATTTAGTATAAGATCTACATGTTTTTCTTGGAGTCTAAGGTAGAATCCAAACGACTGAAGTTTTGTGTCATTTAGATGTCACGTGTAACGACGTGATCTACTCTTGGGTTGATAAATGGATTCCAGGTTTAGAAAGTAGGCTTTACTGCTTTACTTAATGTCGGGCTGTAGGCTAAATTTTAATTTAATTATTTCGTATTTGTAAATATTAGGCTCGTTTACTGCACGTAGTTAGTTATAAATGTATGTGCAGCTTATGAGATTGAGTAAAATATGAATTTATGGTTTTCATATAAGTGAATAAATGGACTAGTATGTTATCCTTCATATAGGCACAGATGTGTTCCTAAATTTGGCAAGTGGGGAATAGTATTTTTCTTCTACCGAATTTCAGTAAGGTTTCAAATTATTATTATTATTAAGTGGTAAAATGAGTGGAATTACGATAAATCCGTAGTAATACGAATCTGATGCTCAAATTTCTCAGCTTGAATTATTTAATTAGTTTTAGTAAACTTTTGTAATTATAACATAAACTTAGTGATTCGTTTATTCTTTGCTGATATCGTTGTGATAATATCATTAGGAATTTTATTTTTTAATTGTAGTTGCAGACCATTCCAATGTCCTAAACTATTTTTGTTTGGAACCGTAGTTTTATTTTGTAACTTGTAAATTATTAATGAATGTTTTGAGCAAGTGATTTTGCTTCACATCATTGAATGCGTGTTGAGTTTTGATATTAATATAATTTAATATTGTTTACTATATTATCTCTGATCGATAAGTCATGAAATAAATAAGTCAGAAATCAGCAGCATTCAGAACATTTAGAACATATCCCTACGCTAATCGGAGCAGGTAAATATTAGCAAGTTATCATAAATCTGTTTTTATTTCTATCATTATATCGGAGCAGATCAGTTCCATTAGAGGCATGCAGTCGCCAGATATTAATCTAATCTAAATCCAGCATGTATGTTCCCACTCTTGTCATATTGTACCTTTGCAATACATCGACTTTGTCAAATAAGGCTTCTGGTTTATTATTTCATGTTTTTTGGACTTCGCAAACAAGTTATATAGAAGTGTACAAACGCCAATCATGCGATTGATGCATTTTCAGGGAAAATAAATATTTTCTGCCTTCATACCGCTAATGTTAGAATGGCCGGCAGCACACATAGTTACGCATCCGTGACACTGCTAGACTATGGACCTCTATTTAATAAGAGGGGATTGTAATAATCTTCAAGCATGGTAGGCGCGTTGGAGATTGCAGTTTGATAGGTTCAGGTTTACCAAAGGGCGCTGCTGGCCCCGTCAAATATGTAGTACCTTATTCGGCTCTTACGACAACAACGGGAAGTTCAGCGATAGTGACTAATGTTTTCTATCCGACCGTCACCTCACGACACAGCCAGTTCATAAACTATGTAAAATACCTACACACACCTACATTTTTGTAATTTCATGTTTGAAAACGGTTACTTCGTCACACTATTATCCACTTATTAATATTGTTTATTGAAGATTATTAATTAAAGTGATGTATTTGCTAAGTATTTATTTTTGGAACGTTAAATTAAAAAAAAAATGTTTGATTTAGAATTGCATAGGCAACGGAATCTAAACCGGAATGATCAATTTGAATATTTCAATTACCTAAAATCAACTACTCAGGATATTGCATAGAAATTCTTGTGCGTAGTATTTTGAGGGACACATTAACCATTCAATCTATAAAACATATTCATCAAACAGCGATTTTTTGCTGGAATATCATTCAAGGAACATTCCTGCATTAGTTGACTTACACATTATAGAGCCCATTCATTTGATTCTGATTAATTATCATTAGTACTAATGATTTGTATTTCATCGTAAACAGTATGTAATAGATAAGTAAATACATAGTTTAGTCTTCTATTTAAATAGTTTTACCTCTGAGAATATTTGCTATTTCTTAACTAGCCGCACAATAGTCCATGCAATGCTTTCCTAAATTAGTTCAGTTTAGATGAATTAGCTTTTTTTGCTCGCTAGTACTCTACTAAAGCAGTAGTTGAAGCATAATTGAGCATTAGAAACGCGTTGTCAATATGGGAATCATATTCGTGGCCTGCACTGCTACAGTGACTGGCGTCGGATATCCGTTCCTATCTTCACAACGTTTGGTGACTGTCCTACCCTCGAATATCTGACAAGCAGGGCTCCTGTCTTCAGCTACGACTCGCAATGGATTTGTTGTGCGCAACACGTTTGAGGTTTGAGTTCTTGACATACACATACACCATAAATGATACGTTTAACGTAAAGACTCAAATGAATCAACATATAGGAATTCATTCAAGCATTCGAACATTTGTAGAAATAATAGCAAATGTAATCGAGTAGAAAATCGAGAACCTACTGTGTAATTGTTTAGTCATAGAATAAAACCCAGAGGATGTACTGCTGAATTATTTTCTCAAGATATTTGTTTTATTAAGGGTACAAACGCCGTATTGTACTCATAGCTTCAGCAGTCCACCTTTAATGTTAACTAATTGCATTTTGTTCATAGAGAATAATAACTATTTGCATAAACTGCACGGGGTACAGTGCGGTTAATACTAGAACACGATATCCGCTAGATACAATTTTTACATTACTTTGAGTTTCGTTTAGTGCTACTTACTATATATTTGTTAGCGTTCTAAAGATGTAAATTAGAAAAAAATGAAGTCATTATAATATAGCTATTGAATGTAATCAAATAAAGGCTTCTTTAATAATAATTTAATACCTTCTATACATTTTCATTGCCAAGTACACGTAGATTATGTATGTTTGATTAATCTTTCAGGTAAAAGTGTAGACAATGCATTTTCAATCCAGATTATAGCCATAAATTACGTAGACTAAACATTTGGAATGAACCTGGTTTTAGACAAAGACGCAGTTTAAAAACAAGTTCATTTTGTATACTCCCAAATTTTAGGCGAAACGTAGACTCTGTTTTTGGATAACCCAGATTTGGGCTTAAAAAGTAATACCTTTTAGATATATTTTAGCTCGTAATGTAAATAAAACTTATTGACACTTTCGTATAATAAATAGCACTCAAATATAAGGCACCGTGTAATCTGTGTATTCACAATTTTATTTTATTGCATTTATATTACGACTACGAAAGTCTGTTGTATTACATAATAGAATATAATTATATAATAAGGTAGCACGCTGGTAGAGGAAAATATTGAAAGTAGGGTCTACTATGTGACGTCGGCCACGTCTCTCCGAAACGAAATTCTAAAATATTTGTCTAGTGACTAGTAAATAAGTTCAAGACATTATAGGTCTAAGAAAATGTATCTTAGTTGATCTAAATATAATATTATAATTAAGTAGTTCCGCACTTTCTTAAGTAAGTCACCTTTAATACAAATTACTAGCTGTGACCACATTTTGCTTCAAGTTGATTAAATCAAATCCATGATTATTGCTAAGCAATTTTTTTCCACAAAAGGTTAAAAAGTAATTTATTTGTTGAAGAGTTACACTAAGAATACATAGTAGCAGATGCCAAATGTATCAATACTAATACATCCTGCATTATGCAAAATTTATCATACATTGTCTGATATATCAGATGATGACTAGTATAAATTGGCCTGACCTCGAGTCAAGGTTCCGGTAAAGCAAAAACTCAATTCCTGAAGCCAACTGCTTCTAAAGAATAAATACGCCCAATATTTTTTATAGAATACGTATTAAAAAAATAATTAAGGTAACACTGTTCTCCTAGGAAGTTCTAAAAACTTTCAATCAATATTACTAAACTATCCTACGTGACAATTTATACTAGTCTTTCTCTATCTATCACTATGTACGACTTCAATTCGTTTCTTTGTTTTTCGACAACATGGCTTTCAATTTTCCTTTTTATTTACTGTACTACTAAAGATGAAAAGACTTTAACCACTGTCCTGTATTTCAACTTCTAAACTCTGTATTTCCCTTTAGGATATCGATACTTCAATATATTAAATGAGCACGTTTGTTTCCATATACAATAAATTTTAACGTAGTTGAACAATACACAAAATGAACACCCCTAAAACTGACATTACATGTTTGTATTTTCGTATTCAAATTATACATTGAACTCTAGTAAAAGTAAAGCTTAATGTTATTAGGAATGGATTTTGGATATATTTTGAATCGTGTAGGTTAGTTTACGTGGAACTTAAATAATGTTAACGTCTCCGGGGAAATTAACTTAAAATCGAATGCACAAATATCAGTGAAAGTCTTTTTGTCTGTAGATTTTATTTTGAGAGTATATACCGTTGAGAATTTATAGAAATAGTTACATATATTGCAGATATATAAGATAAAGCACCAAGAATGCCTACAAAGCTGTTATTCCCGTACTGGGCACATAATGTTGTAATAAGATTACTATTATGTCACGACTAGTATCAGTGGATACTGAATCAAAATGACTATATTATGTATTATAACTTATAAGTATTTTATTTTTTGTTAGATATTTGATTTTTTGGTACATTTTAAGAGGTACATTTCAGTTTATTTGTGTTTTCCACGAGTTAATTATACGTTATTTACAACGTTTACGAAATTAATAAATAAACTTACTCACATACAAAAAAGTAATAGGAAAATTCATTTAGAACTTTTATATTTTTATTGTTTATCTATCAATTTATGTAGGGTTATACCTCTAGAATCATTCAGTACTTTGGATATATTCAGGAATTCAATGGAAAGCTAAATCTGCCACTTAATTGAAACTTCGGTCATTCTGCCTATGTCTTGCACTAAAATATTTTTTGTAACCTACTTTCTATAACATACATATAACTACTTTGATTACCGTCAGCAAGTGGTCACACAATTTTATAAATAAAAAATATATTTTTGAAACAGTTTTCTTTACATTGCAGTTTTATAAAATATGTTGTTTTTAAAAATAAATCACCATTTTCTGTTATGTACGACATAGATATAGACATAATATACATTTTGTATATCGTATTCAATAAGGTAATTCTAAACATTAACATTGTATGCAATATTAGTGCATCTACATAATTATGAATAGTGTGAAAGTACCGTAACAATGTACATGAAATAGATTTTCTGAAGAAACGCTAGATAAACTTTGAATGTTGTTACAGAATTGAATGATAAATAATATTGAACGCACGTGTCTTAGATATTATAGAGTTTTATAAATAGATATACAATAGTTATAATATATGATAAATAACAGCAAGCCACGAATGGAGCGAATAGATTATCTCTTTTGAAAACTATTTATACATCATTATACATCACTGGATATAAATATTTGTATGACTGCTCTATTTCTTAAAAATATCTATTATTGAATAAAGTCAAGTTTAAAAAAATGACTAATATCTGCTTCGGCAGTCAAAATGGCACCATCACGGAATAGATGCACGAATAAAATAAACTCGATATGAAAAAATAGATAATTTATTTACAATATTCGTTAGAGTATTTTATAATCACGTTATTTATATAAATTCATGAGTACCTACTATGTTTTTTTTCTATAGGACTAATTTATTTGTTTACATTATTATTTATAAACGAAGGTTTAATTCTACACTTACCAGTGATATAGTTTGTACTGTCCCCAACAGCATAACTGATTAAAAGATGGCAAAAGAAGACTTAACGTCTATTGTATATCTTGTTATTTCTTTTCCAATAGATGTTTGCGTCCCATTCGTTTCTATATTGGTACTAAAGTCCTGATTATATGTTAACTTGTTAATTTTTTCATTCTTATTTATTAAGGAACCGTTTATGCCATAACACTTGGGGACTCAACAAGACACAGATAATAATTATTTGATATACAACACGTTATTCCACGATTCTTCGTTTATCGTAATTAAAACAAATCTAGTAACCATACTCCTATTTATAAATAATTTTAATTTTTAAAAAGCCATCAAAATATATTACGGTGAAGCTTTATAAACAAAGTCAAGAATTAATGTGACATTCTTGTTTCATTTAACTATTTAAAATAAATGTACCTACACATTTTCTGCAAGCACATTTATATGTAACTGTGTCTTGTTAACTTACAAATAATGAACACTGCTAATATATTATGCTCACTCTTCGTCATCCTTCCTACCGTATTCAATTACCTAACCGTCAAATATTTAGAATTATATAATTAGCGTTTATTCAATAAAAGGATAAACCTTTTTTAGGTATAGTAGAATTTTTTGTCACATTTCCTTA

>gene14068

TGCCGGCAGCCAGCGTCCGCAAGGACGCACGCGGACGCGCAAAACATGCATACGTGCCGTCCGCGCGGCGTGCACAGCATCGCGCTTGTGCTGGCTCTCGCCATCGCCTGGTTGCCTCATGCTGACCATGCCGCGGGAGCGGGCGGAGGTGGTATGTTTGGTGACGTCAATATCTCAGCCATTTTGGATTCGCTAAGTGTAAGCTACGATAAAAGAGTGAGGCCGAACTATGGAGGACCGCCCGTGGATGTGGGAGTCACCATGTACGTGCTCTCCATCAGCTCCTTATCTGAAGTGAAAATGGATTTCACCCTGGATTTCTACTTCAGACAGTTTTGGACAGACCCCAGGCTTGCTTACAAAAAAAGGACGGGTGTGGAGACACTTTCCGTCGGCTCAGAATTTATTAGAAACATATGGGTACCCGACACTTTCTTTGTTAACGAAAAACAGTCTTATTTCCACATTGCTACTACAAGCAACGAATTCATCCGCATTCATCATTCTGGATCTATTACTAGGAGTATAAGACTGACTATCACCGCTTCTTGTCCAATGGATCTGCAATACTTTCCGATGGACCGTCAATTATGCAATATTGAAATCGAAAGTTTCGGCTACACCATGCGGGACATCCGATACAAGTGGAACGAGGGGCCCAACTCTGTGGGCGTGTCGAGCGAAGTGTCTCTGCCGCAATTCAAGGTGCTGGGCCATCGGCAGCGAGCCATGGAGATCTCTCTTACGACAGGAAACTATTCACGACTGGCATGCGAGATTCAATTTGTCCGTTCAATGGGATACTACTTAATCCAAATATACATTCCATCTGGCCTAATTGTAATTATATCTTGGGTATCATTCTGGTTGAACCGAAATGCGACACCTGCTAGGGTATCACTAGGTGTTACCACTGTACTTACGATGACTACGCTTATGTCATCCACGAATGCCGCACTGCCCAAGATCTCATATGTCAAGTCTATCGATGTCTACTTGGGAACCTGCTTCGTCATGGTCTTCGCCAGTCTTCTAGAATATGCTACCGTCGGCTATATGGCTAAAAGGATACAGATGAGGAAACAAAGATTTACTGCTGTTCAGAAAATGGCTGCTGAAAAGAAAATGCAAATAGACGGGCCACCAGGTACATCGGAGCCTTTACCACCACCGAGGACGAGTACGCTAACGAGACCACCACCACCGAGTAGATCTTCAGAGGTCCGATTCAAAGTTCATGACCCGAAAGCATATTCCAAGGGCGGCACCTTAGAAAACACTATCAATGGATCTCGAGGTCCCGCTCCGGCACCTGTACCTGCTGCCCCGCAGCCTGATGAAGAAGCTGGACCACCCCCGCACCTTATTCATGCTTCGAAGGGTATCAACAAACTGCTCGGAACGACACCATCGGACATCGACAAGTACTCTCGCATCGTGTTCCCCGTCTGCTTCGTTTGCTTTAACTTAATGTACTGGATCATATACCTCCATGTTTCCGACGTTGTGGCCGACGACTTGGTACTACTAGGCGAAGAAAACTAATTTCTCTCGAATAAACTATACTTACCGGACTTGTTCAAACTTAGGGTGCTCATGATCAACCATCCATCAAGTTTCGGTAATGCGCTTTGGAATCCTAGAAACGCTCGATAATCTATTAGCGTTCTGTAATCTTATAAATATAACAAATAAACATCACTATGATTATAATAGATACGTGTCGCGTATATTGACACTGGTAATATTGAATTCTTTAGAAAATAGAGATAATATAAAGTTCAAAATATAAGTCAATCTGTCTAAGAGACAAAATGTTATATCTTCAACAGTTATCAGTCTTAAATAAATGTGTATGTATACATGAAAATCCGCAGTTATAAAGTAATATACATTTTATGGGATTAAATTAGTTTTTAATAAGAATCCAAGTTTATATACAAATTACTAATTGAAAATCTAAGCGCTTTTTATTGCTAACTTCAAATGATGGCTGTCTTTACTTCTCTAGTTGATTAAGTAGCAATTTTATAGAATATATATCACTAATAACATTGAAAAGAATATACTGAGTGGACAGACGCGTGTATTCAATTTAATCAAACTGTAGTTATTGTCACTTTGATCGGCAAGGCATTGAAAGGATAAATTCAAACGTAAGATTTTCAGTCATTTTAATCCACACAATACATCAGCAAACTGTAATATTAATTAAATAAATATTAATATCCAAAAAATATGGAGTCGATTGTATTAAAAATAGATTATTAAATCTCAATTAACACGACAGTAGTCACAATTTCATAGCCCGGTATCGCCACAAAGATTTTTCATTTATTTAGAAGATGCTGATGTTATAGAACATCATTTGCCCGAATTATGAAATGTATTTCAAATATTCGTATAAAAGTATATCGGTACAAAACAGTAAAGTAACTACAATACACTAAAGGTAACAGGGACTACGTACTTTTTGACGACAACTGGTGTTTAGGAAGTTGGACCACAGTCCCTTACCGATAGTGCCTCTTCATATTACAGTACTAGTCATTAATCTATGAAATTGACATTAGCGCGCGTCACTAGGTACTTAAATAAAGAACTAAAATATAACGACGGAATTTGAAGAGGACTTGATACCGTATTTACGATCATGAGTCCTTTATAGGACAAGATGTAAATAATTATACATAATATTATATTTATACGATTAGGATACACATACACGATTGCTCTAGTTTTTTAAAAAGTTATTCGGTTCAATTTAACATGTATACACCGGTATTACACTTCAAATTAAATCTAGTGCACAGAACACTTTTTCTACGTAGTTTAAAAATTTACATGTATCAATTGAATGTAATGTGTTAGATCTGACGGAGTGACATGATATGTTCTTAAGAGTAAGATTGGTTCATCGATCTTGATTTCTTACTTCTCGGTCTTAATCAACCTTGAATATTGATCGGGTTTGTGTTATATCTAAGGCAACATATATTACGCGGAGAGACTCGGTGAGACCTTTGTAATGTACGTCTTTACACGTATTTCGTAAGTACAATAAACTATAAGATAAAAGAAGAAAGGAAGAGAAGAGTTCGTAGAGTACCGTTATTAGAGCTGTGACTACTCTAAGTTTTGATCTCACAATTAATTGAAAATATACATAAACTTAGAAAAATAAACTTATCTACTAATAATAATGTAAAAGGGAAACAATTCTTAGTTGTGTTTGTGCTCATATAGGCTTGTAGAGGTTTTATTGATATTGTTTATATTTACGTATACATTCCAATTTAACTGGGAAGACATTTTCCCAAGTTTCGTTTTATACGATACTTCCTTGTTGTGACTCCAGTTTTTAGATATTTACAGTATCATTTTGGAGTATTTTAAGATTTGAGCTACAAGCCATTTCTTGTTAGAAAAATCCCTTATTATTTATTTCTGAATTTGCTCAACGTTTTTCAAAGACACTATTTATAAATATGTATAAGGGAGTGCAAATAGTTGATTTGACTAGGAAATGTAATAAATAGGTTAGGTAGTGATATTTTTAAGTAATTATAATTTGCCTCCTATTTTATACCCACATTCTCAGACTGTGCATTAAGACTAGCGTTTATAAATTTTATCATTTAGAAAGTCTATTTCTGTTCTAAAGATATTAAAATTTATTTAAAATACAATTAATCCTAAATATTGTATAAAGACTGAGTCAATATTCTTTTAGTTCCTCATATTTACAGCTATAATGAACATGACAGCCATCTATAAGTTCCCTTGATTACGTAAGTTCTTCTGTTTCTAGATACTGCATTAACTAAAAGTAACAAAAATATATAAGAAATCTATTGCATTACTATCGCAAGATAGTGACATAAATATTCTGTTCTAAAATATATTTATATACATATATATATAATAATGACGATACGTTATAATTAGTAGTTACAATCAGCCAAATTGATAAGTATATCTTTAACGATTAACTATTAGTCGACCTTCAGAATGAACTATGGATTAAATGTACATGATTATGAAAAAAAATATGTAAGATGTGAAATTCGCATTATATTGCACCTAGTTAGTAAGTGAGTAGCGTAGCTTAGCGGACGGGGGAGCGGGCGAGGCGAGCCGAAGGGGATGGCCACAGTTTACTTAGTAAAAGTTTCGGGGCTGGAGTACAGCGGCAGTGGGCCAAGTTATTGGCACGGCTAGCGACTCTTGCTAAGCGGTGACGCTCGAGCCTCTCTCCGCTCAATTCCAATTTAAACTGCATTCTATCTACACTACTACTATGTACACTGAAACATCTAGCGCGCATTGCCCGCAAACCTCTTTCGCGAAACCAAATTCTAAATCACATTTAAATAAAATCTAGCATTATTAAAAAATTATACTGTGTGAGTAAAATCAATCTAGAAAACTAAGATGATGATGATAATTTATTATGATATAGGTATCAAATAAAATTTCAATAATATTAATATATATATATTATATTATCACGTTTCCCAAATTAGTCTACCTACTCTCGTATAACTGATCCACAATGAAATATTTCGCACTAGAAAAACTAATTAATTACTTACACTTGTCAAATATTATGGAGATTTTCTTGCTGAGATTTTAGGGACTGGCAAGTGACTTCGTGTTCAGACGAATTAATAAATAGTAGTGAACGAGTATTAAAAAAACTAAAATCATCCATATAAACAAAGGCTGTCGAAAGTAAATATTCTAAACTAATTCTGACTACTTTAAATTATTGTAAGCAACAAGCATGAATCGCCGTCACTTGCGCGTGCGCCTGTAGGTGGCGCTCTAAGTACTTTGTAATTATTCTTACTTTGCTGCACATTCAGGTATTGGGCCTGCAAGAAGCACATGCGTTCAAATGGTTTTACATAGGATTATACTTAATATGTAGATATTACGTACACATCATAACTATCGGCACTTGATTTCAAAGTAATTCTGCATCATAGACGCATATTATACATACATATAAAATATTTAATAAATAAAGCATAGTAAATGCAGACTTGAATAGTGCCAAATGCGAGGCTTTAAAATTTTATTATTCGCTTTTTATTACGAATATATTTCTACCACATCTGTTTTAATATAATAAACTGTCAATGTCATTAGATGTGTCATATTTGTGTTTCAATTGAAGTATACCCTAGAAATAGATAACATTGTGATGTATATAATAAATTATATACAGTATATGTATACATATATATATCCCTGTAACTTTGAGCAAAGGTTTGTTGTTGAACACGAGTATGTATACCACTTTTGTAAATAAAATCCAACGATCTGTCCTACGAGGAATTACTTCATTTACAGGCAGTGTAATGTATTGCAGTTACTTGTGAATTGTATCTCGATGCTGATTTTTTTACCTATACTTGTATTAAAGTGTTTCGTAATAAGATTTAAAATTTTTGAGGCATATCATGTGTTAGAATTTATTATGCTGTTTCTTTGTTATGTAATTTAATATCCTCTGCACATTCATTTATATGTGAGTGCTTATGAAAATATTTCATCAATGCTTAATGTATTTATTTAATTATTTCTATGACCATGAAGTGATACAAGCGAGAGCAAGGTTCATCTCAAACTTTGATCGAATATTTTCATAAGTAACATTGTGATTGCACCTCGAAGTATGTTTATGTAATTATATTGAGCTTCTGCGTGTTTTATGAACCTGTGTATATTTTATTGACAATTGTATCAGTGTTAAGTAGTGTTCTGTTATCATTATAATATAAGAGAAATAAATTAAAATATCCAGCTAATTATAGTTTTGGTGTTATTAACGTTTGAAACTAATAAAAATTATTAAAATCTTTTGTTTCAACTTGCTGGTAAAATCAGTTACTACTAAGTATATGAATATTTCTTCGACCAAAATATTTATGACAATAATTAATAAACCATTTACCTAATAAAAACGTCAGTATGTCAACATCGAATCGCTTGAACGTAGAAAATTAAAGATCTATGCTGCCGTCAGAAATTACGAGTACTTATCCACGTTGTTACAAGTCAGTACAACATCAATCATTTATCTACACAAATATCGTAACTACTGCACGCCAGTTACTAAACAGCTGGTGATAGGTACGAATTTATTTATCGAAACGAATTTATGCAGACCAGGTGAATGAATTGGTACTTAATGTCGCTGGCTGCCATATAACACACGTATTGGCTGAGAACCCGGAATCTATGAAAAGAGTTATAAGTATCCTCAATTCTCTAAATACTATAGTTACAAACTATTGTCTATTAACCAATGTTCTGAGACAATAATTCATTATTCATACTTTCTGGTCTAAAGAACGGACGACAACTAAAGTATTCCTTAAAACAATCACATTATGGCATATAATATAACCGATTCCTACAAAGCCCTGATTAGTCACAAAACTGAAGAGGTTGTAATTCTAAGTTTCTTGGACACGACTGTGCAGCTTTACATTACACCGTCCGTTTCTTGAAATATCATACTTCTAGAATTTCAGCAGAATATATTATGCACCTTACAGCTTTAACTAGTTAATACCTAATAGACCTATCTTTTTGCTTGTTAAAGTACTACCAAAATTCTAAAAATTAAAATTTGTCTATTATAATTTTAGCACACTCAGTTGAAGTGTGTGTACACGTAGTTCCATTAAACAACATTGAAAAGATATTAGTTTGCAGTTAATATTTGGGTCAACTAACCAACGTTACTAACTAGTCTAGTTGCTTCCTACTACTGGTCACAAGTCAGTATTTCTTGGAATATACAATGTGTTTATCGGTCAGTTTAGGTACTACGACTACCGTCACGTAACTTAGATTCACTTACCTAATTAAGCGTATGCACACTTCTACTGATGAGCACTAAGTTCGTAACTCGGCCAGATTGTAGTTCGTATTCCATATATGAATACTGTGCAGTGCTTCGCTTCCACAGAAATATATCTAATATAATGAAACTGACGTAACAGATTCTAAATATATATAAAATATATAGTTTGTTATTGTTTGAGGCTGAAAGTTTTAAAACTCTCGAACATTATATATTGTCTAACAACGTGTTGTGTCAATGTATGAAATAAAGAAGTTGTGTAATTTATTTAATTTATTGAAATGTCTAATATCTAAGTTGAATATGTAACTAAAGAATCTTCCTCGATGCAAAACTAAATTTTATGGTATTGGTGTGGTTAATGATGTTGCTCAAACTGAATAACTTTAAAAGAAGTAGTTACATTGTACGAAATTTAGTTCTGTGATCCAATAAATTATAATATATGATC

>gene15324

ATGAGCGCGCTCCGGCCGCGCGCCCGCCGCGCGCCGCTGCTGCTCGCGCTGGCCGCCGCTCTGTTGCCGCACGCCGACCGAGTCGCCGGCGGCGGCGGCGGCGGGAGCATGTTCGGGGACGTCAATATATCCTCTATTCTGGACTCCTTCAGCATAAGTTACGATAAGAGGGTTCGGCCAAACTATGGAGGTCCGCCGGTGGAGGTGGGCGTCACCATGTATGTGCTCTCTATCAGCTCCGTGTCGGAAGTGCTCATGGACTTCACGTTGGATTTTTACTTCAGACAATTTTGGACAGACCCCCGGCTCGCGTATAAAAAGCGAGCCGGGGTCGAGACGCTCTCGGTCGGCTCTGAGTTCATCCGCAACATCTGGGTGCCCGACACGTTCTTCGTGAACGAGAAGCAGTCGTACTTCCACACCGCCACTACCAGCAACGAGTTTATCCGAATTCATCACTCGGGTTCCATCACACGCAGCATCAGGCTCACCATCACCGCGTCGTGCCCCATGAACCTCCAGTACTTCCCCATGGACCGCCAGCTGTGCCACATCGAGATCGAGAGCTTCGGCTACACGATGCGGGATATCCGGTACAAATGGAACGAGGGGCCCAACTCGGTGGGCGTGTCTAACGAGGTGTCGCTCCCGCAGTTCAAGGTGTTGGGCCATCGCCAACGCGCCATGGAAATATCTCTGACGACAGGAAACTACTCGCGGTTGGCGTGCGAGATCCAGTTCGTGCGGTCGATGGGCTACTACCTGATCCAGATCTACATCCCGTCCGGCCTGATCGTGATCATATCGTGGGTCTCATTCTGGCTGAACCGCAACGCGACGCCGGCGCGCGTGCAGCTCGGCGTCACCACCGTGCTCACGATGACCACGCTCATGTCTTCCACAAACGCGGCGCTGCCCAAGATCTCCTACGTCAAGTCCATCGACGTCTACCTCGGCACTTGTTTCGTGATGGTGTTCACCAGTCTGCTAGAGTACGCCACGGTGGGGTACATGTCCAAGCGGATACAGATGAGAAAGCAGAGGTTCGTGGCGATACAAAAGATAATGTCGGAGAAGAAGATGCCGGCGGAGTGCGCGCCGCCGTGGGACGCGCACTCGCTCAGCAAGGCGGGCAGCCTCAGTCGCGGCGCGCCCCCGCCCCCGCGCCACTCGCTGGGCGGCGCGCCCCCGCCGCCGCCGCGCCAGCACGGCTCGCTGCACGGCTCCGTGGACCGCTCCGGGGACCGCTGCCGCCACCTGCAGGAGGTGCGCTACAAGGCGCGCGACCCCAAGGCGCACTCCAAGGGCGCCACGCTCGAGAGCTCGCTCGAGGACAACCCCGCGCCCTCGCTGCACACGCTGCACCCCACCAAGGACTACGGCAAGTTCTTGGGCATGACGCCGTCAGACATCGACAAGTACTCGCGCATCGTGTTCCCGGTGTGCTTCGTGTGCTTCAACCTGATGTACTGGATCGTGTACCTGCACGTGTCCGACGTGGTGGCCGACGACCTGGTGCTGCTGGAGGAGAACAAGTGAAGCCTCGTCCGCACTGTACATACCTCATGTATTATAGAACGCTAGTGTCACGCCCCTCCTACACTCAACTGACGGTCTGAACTTCGTACTTCAC

>gene3536

CACTACGGACACATTACCTGCATTTTTATATCCATTTTTTTTAAACAAACACGGATTTTTAAACGGCCATCTTTGTATGAAGTTTTTTTTTTAATAAACAGTTGTGACACACTGTGAGTCGTCTAGAGATGGGACATTATTGAGACGGAGGATGGACATCGTGCGGCCATCATGTGCCCTGGTATTCCTGTTACTGGCTATCATTCACCTCACAGAATGTATGAACGGTGGGAAGATAAATTTTCGAGAGAAAGAGAAGCAGATCCTCGATCAGATCCTGGGTCCAGGGCGTTACGACGCCAGGATCCGACCTTCAGGCATCAATGGCACTGGCTATGCGCCAACGTTAGTCCATGTCAACATGTATCTACGGTCCATCAGCAAAATAGATGATTACAAAATGGAATACTCGGTACAGTTAACGTTCCGAGAACAGTGGTTAGATGAGCGCCTCAAATTCAATAATTTGGGAGGTCGCCTCAAGTACTTAACCCTGACAGAAGCCAACAGAGTCTGGATGCCGGATCTGTTCTTCTCCAACGAGAAGGAAGGCCATTTCCACAACATCATCATGCCCAACGTGTACATTAGGATCTTCCCTAATGGCAATGTGCTGTACAGCATCCGAATCTCCCTCACGCTGTCCTGTCCTATGAACTTGAAGCTGTATCCTCTGGATAAGCAGACCTGCTCGTTGAGAATGGCCAGCTACGGCTGGACGACAGACGACTTAGTGTTCCTGTGGAAGGAGGGCGACCCAGTCCAGGTTGTGAAGAACCTACATCTGCCTCGGTTCACTCTAGAAAAGTTCCTTACTGACTACTGTAACAGCAAAACTAATACTGGTGAATACAGTTGTCTGAAAGTAGACCTGCTGTTCAAGCGGGAGTTCAGTTACTACCTGATCCAGATCTACATCCCATGCTGCATGTTGGTCATCGTGTCCTGGGTGTCCTTCTGGCTGGACCAGGGAGCTGTGCCTGCTAGAGTCTCACTAGGTGTAACGACACTCCTCACGATGGCGACCCAGTCATCAGGTATCAATGCCTCCCTGCCACCAGTGTCCTACACCAAAGCCATCGACGTCTGGACAGGCGTGTGTCTCACCTTCGTGTTCGGAGCACTACTAGAATTCGCTCTAGTCAACTATGCATCTCGATCCGACATGCATAGAGAAAATATGAAGAAAGCAAGAAGGGAGATGGAAGCAGCCAGCATGGACGCTGCCTCCGACCTCCTCGACACAGATAGTAACACCACGTTTGCTATGAAACCCTTGGTGCGCGGCGGCGTGGTGGAATCCAAGATGCGGCAGTGCGAGATCCACATCAACCCTCCGCGCAAGAACTGCTGCCGGCTCTGGATGTCCAAGTTCCCCACACGCTCCAAGAGAATAGACGTCATCTCCAGGATCACCTTCCCACTTGTGTTCGCCTTATTCAATTTGGCTTATTGGTCGACGTACCTATTCCGCGACGAAGACGAAGAGAAGTGATTTTCCGAGTCTCTGGAGAGAGGCGCGGGCCCGCGGCTGCGGCTGGCGGCGGCCGTGGTGGTGCCGTACGTGTTGTTCGTGGCGGCCTACGCGCTCTGCTTCAGAGTCCGCGAGCTGCCGCCCCCGCCGCCCGAGCCGCTGCAGCTGGAG

>gene491

GCGTGGTATCGAAGCGTTTCCGAAAGAACTATATTCACCGGTTCACGGCCACCAGGTCGTTGTTCTGGTACTCCCCGTGGTCTCCAGTCCGAAGAGCTTGCGTCTACCTCTCCACCAACCAGTACTTCGATTACTTTGTCATGGCTACCATACTGCTCAACTGTGTCTTCCTCGCCATGTCGGAGACTATCGAAGAGGCTGAGTACATTTTCCTGGCGATCTACACGGCGGAGATGATAATTAAGTGTATAGCTAAGGGTTTCATATTAAATAAGTACACATACCTACGGAATCCGTGGAATTGGTTGGATTTCGTCGTCATCACGTCAGGGTACGCCACCATCGGCATGGAGGTGGGCAACCTGGCTGGTCTGCGGACCTTTAGAGTGCTAAGAGCATTGAAGACTGTTTCTATAATGCCGGGTCTAAAAACTATCATCAACGCGCTGCTGCACTCGTTCAAGCAGCTCGCAGAAGTAATGACGCTGACCATCTTCTGTCTGATGGTGTTCGCACTATTCGCACTGCAGGTCTACATGGGGGAACTCAGGAATAAATGCGTGAAGAACCTCGTCATTCCACCTGGAGAAAACTTCACTGATGAAGCGTGGTCAGCGTGGATTCAAGAGCCATCAAACTGGATGGTGAATGCGGAGGAGGTGCCTATAATCTGTGGCAACCTGACAGGGGCCCGGCATTGCCCCCCAGAGTGGACCTGTCTCTGTGTGGGCCCCAACCCTAACCATGGGTACACCAACTTCGACAACTTCCTATGGTCCATGCTCACCACATTCCAGCTTATCACCCTCGATTATTGGGAGAATGTATATAATATGGTACTGTCATCATGCGGTCCGATGTCGGTGTCATTCTTCACAGTAGTGGTATTCTTCGGATCGTTCTACCTCATCAACCTGATGCTTGCCGTCGTGGCCCTCAGCTATGAAGAGGAGTCGCAGATCACACAGGAGGAAAGGAAGAAAGACCTGAATGAGCATCGCGACGATTCCACCTTCAGCTTCGACCCCACGTCGCTGGCGGTTCGGACGCTCGCCAAGGACTCGAGAAAGCGCATAGACGCGCGTAAAGGCCTGCTACTGGCCTCTTATTCACGAAAACGGACAAGAAGACGTAAACGGGGCAGAAGTGCTATTCATCATGCAGCTGCAGTTGCTGCTGTTGCTGATGACGCGGGCAAGCGGTCCCGGTCGCGGTCTCGCTCGCGGTCCGTGACCCCGAGCGCATCACCCCCGCCGCTACCGGCGCCGCAGCCCCCGCCCCCGCCCCACACTCTGCACCCAGACAATGCATTAGGACGAGGTGCCCTAAGTGCGGCGGGACGTCAATTAAGTGATCACAGTAACAACAGGGAGTCTTCCCTTGATGACTCGGGTGTGGTGGATGACCACGATGATGGAGAGCATACATCTGACGACCAAGCACCACATCCGCACCCTCGGAGACATCTACTGATACCTCAACTGCCACCGCCGCCAAACCACCCACCTACACCGATGCCAATGCCGATGCAGACCCCCATGCAGATGCCAATGCCAATGCAGATACAGATGCCAATGCCTATGCCTATGCCTACCCCAATGCCTACCCCAATGCCTACACCAATGGTGCCCATGCCCATGCCTCAAATTCAACAGGTTTCTCAAGACAGCCAAACACAAGAATCAAAACACAAACACGCAACAAAAATTGAAAAGCCTGCTTTGAAACCAAAAAAGCCGACAAAAAGCATACAGGACAAGTACCCGTTAAACCCGGACTACCTCAACCAGATCGTCGTGCTAGGGGCGGACTGGCCATACAAGCGCGCGCCCGACCTCCCGCAAGCGGAGACGACGAAAATTAATGAGCTCAGTTTATTGGCAGCCACGCACAAAACTCATATGGGCAATTACGAGCAAAATTGCACTTACTTCACAGATGAGCTCGTGGATAGAAACTGTGAATGTTGCGTGTCTTGCTGTATAGACTATGAAGGCTGGCTCCAGTTCCAGAACTGTTTGTATGGGATTGTGAAAGATCCCTTGTTTGAGTTGTTCATTACAACTTGTATTGTTCTTAATACACTCTTCCTTGCGCTGGAGCATCATGGGATGAGTGAGAACGTTAGATCGGCCTTAGATATAGGAAATAAGGTGTTCACGTCGATATTTACATTGGAATGTATAATGAAAGTGATGGCGATGAGTAAAGACTTCTTTGCGTGTGGTTGGAATATATTCGACTTGATTATAGTGTCAGCAAGCCTTCTAGATCTTATATTCGAGCTCGTCGATGGCCTATCTGTGTTGCGAGGTCTTCGTTTGTTACGAGTGTTAAAATTAGCTCAATCATGGACTACAATGAAAGTGTTATTGAGTATTATAATATCGACAATCGGTGCTCTTGGTAATTTGACGTTCGTGTTAGTGATAGTTATATACATATTTGCTGTGATCGGTATGCAACTATTCTCCAAGTCCTACACGCCGGACAAGTTCGACCCGGATCCAGTACCCAGGTGGAACTTCAACGACTTCTTCCACTCGTTCATGATGATATTCCGGATACTTTGCGGCGAGTGGATCGAGCCTCTGTGGGACTGTATGCGCGCGGAGCAGGAGACAGGGGCAGAGAGTTGCTTCTTCATATTCTTGCCCGCACTCGTTATGGGCAACTTTATGGTGCTCAACCTCTTCCTTGCCTTGCTGCTCAACAGCTTCAATAGTGAGGAACTTAAGAATAAGAAGGAGGAAGTTGGTGAAGATTCAAAATTAGCTAAAAGTTTCGATAGAATACGTTCTATAGTAAGAAAAAAAGGTTTCCTTATATCGAGAAGTAAAGAGACTGACAAAAAATCTAAGTTAGAAGAGCTAGTTAACGAATTTATGACACAACAACGCGCTATAAAGGAGAAAAAGGCGTCAATACCGAGTGAGCAGAGGTACTCAGCATCCATACAGGAGACCATCCTGTTCCCACACGACAATATTTATAGTCATAGTTATCAAGAAGCATTAAATAGGCCAATATCAGTGGGTTCCGACTTCGCGTACCCACATCTTTACCAAGCTACCAACAACTTTAACCACGGTAGTCAAGAAACTTTAAAAGACGTGCGTGACTTGGCGGCCGAACATAAAATCACTAGCATTAGGGAAGATTCCAAGGAAAATCTTGAAGATACATCGCCTACAGAACAAGTTGCTGTTCAAAGGTTATTGAACCAAGTATCCTCTGGCTATCACACGCAGCCATCTGATTCAAGAGATGAGAACGAACATTATGAAATGGCCCAGTTGTCGAGTAGAACAACGCCAGAAAAACCGAAGAGGCAACCCAGGGAACTACCACCATCGAACAACTTAAGTGGGAGAAAAAACTCACAGGGTCAATATATTGATGCGATGGGAAACTTCCCCGGATTTGGGAAACAGAAAGAGCCGAAAGTACCCGAAAATTGTTTCCCTCGCCAATGTTACGATGCGAGTACTTGTTGGGATGCATGTATTGAAACCAACTTGGGTCAGCGTTGGATGTTTCTCCGGACCTATATCCTGCGGTATGTAGACACTCCAGCCTTCGAGTGGTTCGTATTGGTGCTCATCTTTGCATCAAGCATCACGCTCTGCTTCGAAGACATTCACCTCGAGAAAAACAAACCATTGAAGAAAATCCTATACTGGACCAACTTGGGGTTCTGTATGATCTTCATTATCGAGATGTTTCTCAAGTGGATTGCGCTGGGGTTCTTCAGATATTTCACCAGTTTCTGGACTCTATTAGATTTTACTATAGTATTTGTTTCAGTATTCAGTTTGTTAATAGAAGAAAATGAAAACTTAAAAGTGTTAAGATCGTTAAGAACTTTACGTGCGTTGAGGCCATTGCGAGCAATATCGCGCTGGCAGGGAATGCGTATCGTGGTGAACGCGTTAATGTATGCGATACCCAGTATCTTCAACGTATTGTTAGTGTGTTTAGTATTCTGGTTAATATTTTCTATCATGGGTGTTCAATTTTTTGGTGGAAAATTTTATAAATGTGTCGATGATGAAGGCGAATTACTACCATTAGAGATAGTGAACGATAAATGGGAGTGTTATTATAATAACTTCACTTGGATCAATTCAAAAATATCGTTCGACCACGTGGGCATAGCCTACTTGGCATTATTTCAAGTTGCCACGTTCGAAGGCTGGATGGAAGTGATGGCCGATGCAGTCGATGCACGAGGTGTTGATTTGCAGCCAGCGAGAGAAGCTAACATCTACGCTTATATTTATTTTGTCATATTCATCGTATGCGGTTCATTCTTCACTCTTAATCTGTTCATAGGAGTAATTATAGATAACTTTAATATGCTCAAGAAGAAGTACGAAGGCGGAGTTTTAGAAATGTTTCTTACCGAGAGTCAAAAGCATTACTATACAGCAATGAAGAAACTTGGTAGAAAAAAGCCCCAGAAAGTAATAAAGAGGCCAAGGAATCAGTTCTTAGCAATGTTCTACGATCTCTCGAATTCTAGAAGATTTGAGATCGCAATTTTCGTTTTAATATTCCTAAATATGCTCACTATGGGCATAGAACATTATGATCAGCCGCATTCCGTTTTCTTTATATTAGAAGTTAGTAATGCGTTTTTCACGACTGTGTTTGGTTTAGAAGCGATAGTGAAAATAGTAGGTCTTCGATATCATTATTTCACGGTACCATGGAACGTGTTCGACTTCCTGCTGGTGCTGGCGTCTATCCTGGGCATCTTGATGGAGGACATCATGATTGACCTTCCCATCTCGCCAACCTTGCTACGAGTCGTACGAGTGTTCAGGATAGGGAGAATTTTAAGACTGATTAAAGCCGCTAAGGGCATTAGAAAACTACTGTTCGCGCTAGTAGTATCGCTGCCCGCGCTATTCAACATCGGCGCGTTACTAGCTCTCATCACCTTCATCTACGCCATCATCGGCATGTCCGTCTTTGGGCACGTCAAGGAGCAAGGAGCGCTCAACGACATCGTCAACTTTCAAACTTTCGGGAGAAGTATGCAGTTGCTTTTTCGTTTAATGACATCAGCCGGCTGGAACGACGTGTTAGAATCACTTATGATCCAGCCTCCTGAATGCGAGTTAGGCGAACATCCCGGCCAGAATGGCAACTGTGGAAGCCCACTCCTGGCCATCACGTACTTCACTTCGTTCATCATCATTAGCTACATGATTGTCATCAACATGTACATCGCCATCATCCTCGAGAATTTCAATCAAGCGCATCAAGAGGAAGAGATTGGAATCGTTGAAGATGATTTGGAGATGTTCTATATTCGGTGGTCAAAATACGACCCACACGCAACTCAATTCATAAGTTTCGCTCAACTATCAGACTTTATAGCTTCCTTAGACCCTCCGCTAGGCATCTCGAAACCTAATACAGTGGCTTTAGTCAGTTTTAATCTGCCGATAGCTAAAGGCAATAAGATTCACTGTTTAGATATACTTCATGCCCTTGTCAAACATGTGTTGGGGCATGTCGAAGAGACTGATACGTTCAGACAGCTTCAAGAACAAATGGATATTAAGTTCAAGAAGCAGTTCCCTACAAGGAAAGAGCTGGAAATCGTTTCCTCGACGAGGATATGGAAGAGAGAGGACAAGGCAGCCCGGACTATCCAACGGGCGTGGAGAGATTACGTAAAACGAAAAAATCGCAGCCCATCACAAGAAGAAGAGAGTACAAAGTGGTCACCTGGCGGGGGTTGGGGCGGGCGGCTGAGTGCTCTGCTGCACGTGCGGCGCGGCTCGCACGCGTCCAGTCGCAAGTCGTCGCGAGCGTCCGACGCGTCCGACATCAGCGAGTTAGGGGGCGCCTGGCTCAACCTGCCGCTACTGCTGCTACCAGGGGCCGCGCCCGGGGAACAACCGAGTGGTTCTGAGTCAGCGCCGCCTCGAAGGCGCTCGGCCTACGGATTGCCAATCTTCCTGCGGCATCAGGACGCCTTGGACGAAGAGACCGGCCCGAAACGAGCAGGTTCCCTTCGGCTGAGCTCGCGGCGCAGCTCCATGCGTCGCGTGACACCGCCGGCGCGCGCGCGCACTCCGTCGCCACACGCCAACAGTGAGGTCAGCATCCTCGTGACGGAGGCGTCTCCTGACGCAGCTCCAGTGGCCACGTCTGCGCCTCCCGCGCCCCTCACCCCGACTGCGCCCCCCGCGCCCCCGCCCACACTGGTGCGTGTGCTGGTGCACCGAGAGTCGGACGAGCGCCCCAGCTGATCTCCGACTCCCGAGCTCGAGCCCCGAGCTCCTCCTCCGGACGTTCCCCCATCGAACGACCCCGATTGACTTTCCATCCGGCAGTGCTCCGGCGAAAATTGTGATTTTTAAATTTAATTAGCCCCGTTCGCGGATGTCATTATGGTGGATACGTTGAGATAGATGACCTCGTTTCCCTAAACCTAGTAAACGTTAGACACGAGAGTTGTAATTTTGTTATTTACGGGTAGAATTTGAAATGCGCCCATTTATCTCAACAATTAATAATTAATAGATAGTTAATACAGTAATAACCTGAAACTTCCAGAGTCGTTACAATAAAGTTTTCATTAGTATCTCGTCGAATTCAATATAGCTTTACGATACCGAACTGCGCTCGGACGCGCACGCAAGTTGCATTACACGCCGTTAGGTAACATTCGTTTTTAGACGCTTTAATAGAGTAATGTTGCTGAATCCGATTTCTCTGGATCGTAGAAAGCTGGGATTAATTAACAGATTCATAACAATTACCTTATTCAATTATAGACGTAGAGAACCAAAAATGTGCCTAGAAAAATACGTATAAACACAATAATACATAGAAATCGGAAGTCTATGCCAAAAATAATTAAAAATCGAATCTAAGCATAGCATGTTGTTAAGGTTTACTACTTTGAAATACTTACTTCAAACAATTTTGTGATATTTTTTAAATCATTAAAGTACGTATTAATCAATTTATCTTAACTTTAAGAGTTGTAGAGTTCGATTTTACTGTAGATGTTGCCTTATCCACCTTCTACGAACCAGAGTTCGGTAAGACAGCAGTTTGTTTTATTAAAATTAATGATTTAAGTAGTTCGCTATTTGAGACCAAGTGTGTCAAGTTTATATTGTGTTATTGTAAATAGTGTTGTATACGAGGAGAATGACTGATGTCACCGTGTCAATAGAATTGTAAAGAAGCCTATTAAATCAGTCTGTACAAAAGATAAGCATGAGCACGAAAGATAAATGAATCAATATCAATAATTTTGTTTCTATAAATAATACGAGACAATACAATAGTACCTATGAAAATAAAAGGGCCTAAAACACTACCCTGAGGAACACCGTTACAACCGCGCTTTTTTGTCACTTGTATAGTTGATTTAAGAGTGAAAAAAAGAATATAAATTCTGGTGATTTACTTTCCTGTTTGTCTAAAAATGCAATTGTTCTATATATTGCAATATCTAGATATTCCTGCATTTGATTGACATAATCAAAACTAATCAAAAGCTTTAGAAGAACTTAAAAAACAATATTTGTTCAGTATATTACTTTAACATGTCATGTTTCCTTTTGTATAGACTGCAAAAAGTGGTTGTAAAGACATCAAGAGTCAGTTTAGGCTTCTGAATTATTGAATACAGTTCACATGCCATATTATTGTTGAAAATTATTAGAAATTAAGATAACATTTTTGCAAAATATTAGTTCTATGACCATTAAAATGGACGGCATATTGTTATGTATTATATTGTATAAGTATGTTGCCATATTATTTAAGGAAAATGTGCTTGACATATTTAATGTAATATTGGAAAACCCCTACATACTTCCTAATTTATATAATGTCTCTAAACATAATGAAACATGTGTTAATAGATACATAGACATTTGAGCCAGTAAGAATTCAGAATGTTTGGAAAAGACTTTTAGAAACGATAACACCATGACCTCAAACTACATTAATTAATACAAAAAATACACAACGTATTCTTTCGATATTATGTCCTTACAAATCTAAACTTATTTCAACTAACGTGCATATACTAAAAAGTAACTTTCTATATTTCAATGAAACATTATCTACCTACAGTAGATTTGACTGCCTTTCCTTTTATCAATAGACATTGTAGACAATTAAATTCAAGGTATTCTGTAGCTTTTGTTCTAACAGGAATTACAAATCTAGACGTAGTTATTTGTTTTTCAAAACACGTCGCCATTTTCTAAGATTTGCTAGTCAATGATCTCGCTAGAGCTTTTAGATCTTTGGCATGATAATTATTAAGTGGGAGTTTTTTAAATGATTAAAATAAAAACTGTCAGTCATTTTAATATGTTAGTTCAGCTCTAACAAACAATATATTATTATTACTATTAGATTACCTATCAATGTTACTGCTTTCTTCATTTTTTAATTTGTACCTACAAACAATTCTGGATTCGTATTTATGCTTACATATTTCTGGTATTTTATCATACTTAGGATTGTTAAAGTTCCTAGTAGGTACTTTTGCAATAACTAAAAAATATTCTTCTTAATGCACAAATATGTGGCAAACGATGGCATACAGACAAACATGTACAAAATGGGCAAATATTCGCACAAATCTCAATAGATATAATTACGTAGTAAGTCTTGGAGTTGCGTTTAATGTATTTATTTATTGTTAGACGAGTTACGTTGGCTTTGTGATTTTTGTTTAGTTTGTAAAACATTTTTGTATGTATTATTTTCTACTCGCCATTTTTATAATCTTACTGTATTACTGTACTATATTATTATCATCTTCAGGCGAAATTTATTAGTTAATATTTAGCCCGTCCACATGTTCCTTTTGTTGATTGTTTTATAGTTGTAACATTAGCTTCTAATAATGTTAACGAGTTGCTAATATAGGCGCACCAAGTGCTTTCGAATTATATTTTAGCTGCATTTATGTACAATCGGCCCGAAAAGTGATTACTTTTTATTTGTTGGCGAATTAATTAGCGCCATATATCACTTTTGGAACCGATATTACAACTGCGACGAGAATTTACTTAGGAGAATCATAAAACGTTCAATAAATAAAGATGTTGATGCGGTGGTTTTAAATAGTTCTAATAACTATAGAATTAGCTTTTTTTCTAAAAGAAAAGTTTAAAAAGAATTTTAAAATTTCTCTAGAAGTATACTTGAGCAGGTACATTTTTAATCCCCAAATATTTTCCAATTTAGCAAGGACCAAAACTAATTTTTGTTCGAAAAAAATTCCATTTACATACTAATTATATTCTTGACCAACTTTTGAATTAATTTTTACTTAGAATTTTCGAGGCCTGTATCAGATTATGCTAGAGTGTAGATGCTGACACCTTGTAACATATTAGTATTTATACCACCTCGCTTTATGACAAGTTTTACACCATTTGCTTTAAACCCTTTGATATCTCAACAAGCCACACTCACAATTAAATATTTTCAATTCTAAAAGGAGGAAGCATGATAAAATTGTTCTAATAGAATAGATGATATTTCTCAATATTGTTCATTACTATGTACATAATGAAAGATGCAATAATTTTTCATAATTTTATCTATAGTTATATCTTGGAGAGGTTGTTAGTGGTTACTTTTAACTATAATTTATAATATGTACGATGTACATGTAAAACTATTGTAGTTAGTGAAATTGCAATGTTTAAAGCATGTGTTGGGCATGATTCAATTTGTGTAACATATGTTTAAATTGTATGATATGTAAATAAAAAGTTTAAATGAGACCTGCGAAATATGATTTTCTTCCGAGGTACAAAATATAAATTAACCATGTTGAAAACTATGATTA

>gene9046

GGTACTTAACTAACAAAGTTTTCGCTTTCATTTGCAAATCATAGTTTCATTAGATGTGTTGTGTTGTCAGTAGGTTGCTCGTGGTTGGTAGTCCTTTTGAATAAGTAAACTGAAATTAGATTCAATTGTTTTTGTCTACTCGTAAATTGGAATTGGTGCTCTATTCTTTTGTTTCTATTCGCAAATAGAACAAAAACTTGTACTGGGTGTGGCTCGACTTAAGAACAACATTTAACAAGGTCTTGCACGCACGAGAGGAGAATATATAATGGAAATTCAAGATAAGCCGTGAAATATATAGACGCGGCTGAGGGCTGACGCAGATGTTGCGCGCGCGCCAAAACTGGAGAGAGGAGCGCGCGCGGCCAGCGGGCGAGGCGGCCACCATCCCGCGCGCCCTCTAGACCTACACACACCCCTAGGCATTGCACTATATACGGTCGGTCATGAAAAATCTTGCCCATTTGCTGGCTGTCCCTATCAGCGATCGAGTGGTAACTTTCATCTCTCTAGTAGCCGCGGTCACACTGCTCTCGCGGACGACAGAAGGGCGGTCCGCCCGTAGGTGGTCGGCGGGAGAACGCGGGCGTGGAGCGTGTAGCCGCACGCGTTTCCCGCACCACACACACCCGCCCATTCACTGTTAGCAGTAGGCAGTTCACTCGGGGGGAGTTCGATAGACAATGTCCGAGGACTTGGACTCGATCAGCGAGGAAGAACGAAGCTTGTTCCGACCCTTCACAAGAGAGTCACTGGCCGCTATCGAAGCCCGCATAGCTGAGGAGCATGCTAAACAAAAGGAACTCGAGAAAAAACGAGCGGAAGGCGAGAACGATTTGGGGCGGACGAAAAAGAAAAAAGAAGTGCGTTACGATGACGAGGACGAGGACGAAGGTCCTCAGCCGGACGCGACCCTGGAGCAGGGCCTGCCGCTGCCGGTGCGAATGCAGGGCTCCTTTCCGGCGGAGGTGTCCTCCATACCCCTCGAGGACATCGACCCCTTCTATCACAACCAAAGAACATTCGTAGTTATAAGCAAGGGTAAAGATATCTTCAGATTTTCGGCAACCAACGCCCTGTGGATATTAGACCCTTTCAACCCAATAAGAAGAGTTGCGATATACATTCTAGTACATCCTTTGTTCTCGTTGTTTATCATTACGACAATTTTAGTGAACTGTATTCTTATGATAATGCCTACGACCCCGACAGTCGAAAGTACAGAAGTTATCTTTACCGGGATCTACACGTTTGAATCAGCGGTGAAAGTAATGGCACGGGGTTTCATACTTCAGCCATTCACATACCTTAGAGATGCATGGAATTGGCTTGACTTCGTAGTTATAGCTTTAGCTTATGTGACGATGGGCATAGATCTCGGCAACTTGGCCGCTCTCAGAACGTTCAGGGTACTCCGAGCGCTGAAGACTGTGGCCATCATTCCGGGTTTGAAGACTATCGTGGGCGCCGTCATAGAGTCAGTGAAGAATCTGAGAGATGTGATAATTCTGACGATGTTTTCACTATCTGTGTTCGCGCTCATGGGCCTACAGATTTACATGGGAGTGTTAACGCAAAAATGTATAAAAGTGTTCCCAGAAGACGGCTCCTGGGGGAACCTCACCGATGAGAACTGGGAGAGGTTTTGCCAGAATGAGACCAATTGGTATGGGGATGGAGGGGAATATCCACTTTGTGGAAATTCATCAGGAGCTGGTCAATGTGAACCCGGATATGTCTGTCTGCAAGGCTACGGTCCGAACCCCAACTACGGATACACGAGCTTCGACACCTTCGGGTGGGCGTTCCTCTCAGCCTTCAGACTTATGACACAGGATTATTGGGAGAACCTCTATCAGTTGGTATTGCGGTCAGCGGGATCATGGCACGTGCTGTTCTTCGTAGTGATCATCTTCTTGGGCTCCTTCTATCTCGTCAACTTGATCTTGGCCATCGTCGCCATGTCATACGACGAGTTGCAGAAGAAAGCTGAAGAAGAGGAACAGGCCGAAGAGGAAGCTCTAAGGGAAGCAGAGCAAAAAGCGGCAGCGCGAGCAGACAAGGCGGAAGCACGCGAGGCTCATGCGCGGGAGGCGGCGGCGGCGGCGCAGGCGGCGGCTTACGCTGAGGCGCACCCTGCCAAGTCCCCCAGCGACTTTTCGTGTCAGAGCTACGAGCTGTTCGTGAACCAGGAGCGCGGCAACCAGGACGACAATACGCGCGAGCGCATGTCCCTCCGTAGCGACCCCTTCCAGGACTCGGTGAGCACTCAGCCCACGCACAAGCCCACCGCCACCGACCCGCACCACGACGCACGCCGCCCCAGGAAGGTCAGCATGGTCCCCCACCCTGAACGCATAAATAAAATCAGCCAGTTACCATATGGGCCACTGCGCGAGGGCTCACAGGCTTCGTTATCACTGCCTGGCTCGCCGTTCAATTTGCGTCGAGGGTCTCGGGGCTCGCACCAGATGGCGCTGCGGCCGAACGGGCGGTCGCGGTATCCACCGGGTGCGGACCGCAAGCCGCTGGTGCTGTCCACGTACCTGGACGCTCAGGAACACCTGCCTTACGCTGACGACTCCAACGCCGTCACCCCCATGTCTGAAGAGAACGGTGCTATCATTATTCCAGTTTACTACGCTAATTTAGGATCTCGTCACTCATCATACACGTCGCATCAGTCGCGGTTGTCGTACACGTCGCATGGCGACCTGCTGGGCGGTGGTAAGGCGCAGACCAAGGAGGCGCGCCTTAGGAACCGCTCAGCGTCGAGAAACCACAGCGTGACGTCACAACCACACGCCTACCCACTGCCCGGCCAGGACTTCTCGTTAGCGTCCAGGCCACTTAGAGAATATGAAATGAGCACGACTGAATGTACAGACGAAGCTGGGAAAGTATTAAAACCATCGACAGACAATCCCTTCATTGAATCTTCGCAACAACCAAACGTAGTGGACATGAGAGATGTAATGGTATTGAATGAAATCATCGAGCAAGCCGGCCGCCAGAGTCGAGCAAGCGATCAGAACGCGGAAGACGATGAGGATGGGCCCACGTTCAAGGAGAAGCTCTTGGAGTGCGTGATGAAGGCAATAGACTTCTTCTGTGTGTGGGACTGCTGTTGGTTGTGGCTGGAGTTTCAGAAGTACGTGGCTCTGTTGGTGTTCGATCCGTTCGTGGAGTTGTTCATCACCCTTTGTATCGTAGTCAATACGCTGTTCATGGCGCTCGACCACCACGACATGGACAAAGACATGGAGAAGGCGCTCAAAAGTGGCAACTATTTTTTCACTGCTACATTCGGAATAGAAGCCATGTTGAAATTAGTAGCTATGAGCCCAAAGTTCTACTTCCAAGAAGGATGGAACATATTTGACTTTATTATTGTGGCCTTATCACTGTTGGAGTTGGGATTGGAAGGTGTTCAGGGTTTGTCAGTGTTGCGTTCCTTTCGTTTGCTTCGAGTATTCAAATTGGCAAAGTCATGGCCGACACTTAATTTACTCATCTCCATAATGGGTAGGACAATGGGTGCCTTGGGCAACCTGACCTTCGTATTGTGCATCATTATTTTCATATTTGCGGTGATGGGTATGCAACTATTCGGGAAAAATTACGTGGATTACGTAGACCGTTTCCCGGACGGTGACCTCCCGCGATGGAATTTCACGGATTTCATGCACAGTTTCATGATTGTATTCCGAGTGCTCTGCGGAGAATGGATTGAGAGTATGTGGGACTGTATGTTGGTCGGAGACGTATCTTGTATACCATTCTTCCTGGCTACAGTCGTCATTGGCAATCTTGTGGTACTCAACCTCTTCTTGGCCCTGTTACTGTCCAATTTTGGTTCATCGAGTTTATCAACACCAACCGCTGATCAGGATACTAATAAAATAGCGGAGGCCTTCAACAGAATATCGAGGTTCATTGATTGGGTGAAACGTAACGTAGCCGACGTAATGAAGCTACTGAAGAACAAATTGACCAATCAGATTGCGATTCACGCTCCCGAGCGAGTCGACAACGAACTGGAACTCGGCACAGACCTGGACGACGCCGTATTGTACAAAGACAAGAAACTAAAAGACCAAGTAGAAGTAGCTATAGGTGATGGTATGGAATTCACTATACCAGGTGACAATAAATACAAAAAGGGAAAAATTTTGTTAAATAATATAAATGCGATAACAGATAATCATAGAGACAACCGGTTAGATTGTGAATTAAATCATCATGGGTACCCTATACAGGACGATGACACAATAAGTCAAAAGTCGTACGGCAGTCACAAAATCAGATCATTCAAGGATGAAAGTCATAAAGGTTCAGCCGACACGATAGATGGCGAAGAGAAGAAAGATGCCAGTAAAGAAGAATTAGGATTAGAAGAAGAAATGGTTGAAGAAGAAGAAGATGGGAAGTTAGATGGAGGTCTGGGCAAAACAGACATCATAGTAGCAGCAGACGAAGAAGTCGTTGACGACAGTCCCGCTGACTGCTGCCCAGAACCGTGCTACGCGAAGTTTCCATTCCTCGCTGGCGACGACGAGTCTCCCTTCTGGCAAGGCTGGGGCATGCTACGGTTGAAGACTTTCAAACTCATTGAGAACACGTACTTTGAAACGGCTGTGATTACAATGATTTTGCTCAGTAGTTTGGCTTTGGCTTTAGAAGACGTAAACTTACCACATCGACCCATCCTTCAGGACATCTTATATTATATGGATCGAATCTTCACCGTCATATTCTTCATTGAGATGTTGATCAAATGGCTTGCCCTTGGCTTCCAAAAATATTTCACAAACGCGTGGTGCTGGCTCGACTTCATCATTGTCATGGTCTCGCTTATAAACTTCGTAGCGGCGCTTTGTGGCGCCGGCGGCATTCAGGCGTTCAAAACGATGAGAACGCTGCGCGCACTGCGCCCGCTCAGAGCCATGAGCCGCATGCAGGGCATGAGGGTGGTGGTAAACGCGCTGGTACAGGCGATCCCGTCCATCTTCAACGTGTTACTGGTCTGTCTTATCTTCTGGCTTATCTTTGCCATCATGGGTGTACAGCTTTTCGCTGGAAAGTATTTCAAGTGCGTGGATCTAAACCACACAACACTAAGCCACGAGATCATCCCGGACCGGAACGCGTGCATCTTGGAGAACTACACATGGGAAAACTCGCCGATGAATTTCGACCACGTGGGCAAGGCGTACCTGTGCCTCTTCCAGGTGGCCACTTTCAAAGGATGGATACAGATTATGAACGATGCTATTGATTCACGAGAGGTGGGACGGCAACCAATAAGAGAGACGAACATCTACATGTACCTGTACTTTGTATTCTTCATTATATTCGGCTCATTCTTCACCCTCAACCTATTCATCGGTGTGATCATTGACAACTTTAACGAACAAAAGAAGAAAGCTGGTGGAAGTCTCGAGATGTTCATGACTGAAGATCAGAAGAAATACTACAATGCCATGAAGAAAATGGGTTCTAAGAAACCGCTGAAAGCTATTCCGAGGCCCAAGTGGCGACCACAAGCCATCGTGTTCGAGATAGTGACGGACAAGAAGTTCGACATGATCATCATGTTGTTCATCGGGCTGAACATGTTGACGATGACTCTGGACCACTACCAGCAGTCCGAGACCTTCAGCACAGTGCTCGACTACCTCAACATGATATTCATCGTGATATTCAGTTCAGAGTGCCTACTAAAAATGTTCGCCTTACGCTATCATTACTTCGTTGAGCCTTGGAACTTATTCGATTTCGTAGTAGTCAATTTCTCAATTCTTAGTTTGGTATTGAGTGATATTATAGAAAAATATTTTGTATCTCCGACTTTACTGAGAGTCGTGAGAGTGGCGAAGGTGGGTCGTGTGTTGCGTCTCGTGAAGGGCGCGAAAGGTATCAGGACGTTATTGTTCGCGTTGGCGATGTCACTGCCAGCCCTGTTCAACATCTGTCTGTTGCTCTTCCTTGTCATGTTCATCTTCGCCATCTTCGGCATGTCATTCTTCATGCACGTCAAGGACAAAGGAGGTCTCGATGACGTCTACAACTTCAAGACTTTCGTGCAGAGTATGATCCTGCTATTTCAGATGTCTACATCAGCCGGATGGGACGGTGTACTGGATGGTATCATCAATGAGGAAGAGTGTGATCTGCCGGACAACGAGAGAGGGTACCCCGGCAACTGTGGCTCGGCCACCATCGGCATCACATACTTGCTGTCCTACCTCGTCATCTCTTTCCTCATTGTTATTAACATGTACATCGCCGTCATTCTCGAGAATTACTCACAGGCGACAGAAGACGTTCAAGAGGGTCTCACGGACGACGATTACGATATGTATTACGAGATCTGGCAGCGGTTCGACCCGGATGGCACACAGTACATTCGCTACGATCAGCTGTCAGACTTCCTGGACGTGTTGGAACCGCCGTTGCAGATCCACAAGCCCAACAAGTACAAGATTATATCTATGGACATCCCGATATGTCGTGGCGACATGATGTTCTGCGTCGACATCCTCGATGCACTCACAAAAGACTTCTTCGCGCGGAAAGGCAACCCCATCGAGGACCCCGGCGACCTGGAGGTGGGGCGGCCCGACGAGGTGGGCTACGAGCCCGTGTCGTCCACGCTGTGGCGCCAGCGCGAGGAATACTGCGCGCGCCTCATACAGCACGCCTGGCGCCGCCACCGCCGCGCGCACGACGCGCCGGGCTCCCGTCTAGCGGCGGCAGAGCGCCGCGGCCGGGCGGCGCGCGCCCGCGCCCGCCTGGACCGACTTCTTCGCGTTCTAGAGACGTTCGACGTGTTTACTGGAGTGTTTTGTATTCGCGTGGCCGAAGCTTAG

>gene1694

TAAACATAGCCGGCCTCAATGAAAGCAATCGCAGTGGGCGGAGACCGATCATGTTACTCACTGAACCGAACAACTGTCAACATTGTCCAGCAGTACTGCACAAACCATGGCCCTGTTTACATATATACTCCTGACCGCCCTACACGCGACCCTCGCACAGGACTGCGTCATCAACAACATACCCGAGCGCATGGCATTCGAGAAACAACTCCGGAAAGACCTAAAATGCAACTACAAAATATTCGAACATCCTCCACAAAACAGCTCCAGTTTTCCAGTCAAAGTCAAATTCGTGATGAAAAAATTCAACTTCAACAGTGAAGAAGACACATTCTCAGTTCAAACGAGGATGTCTGTGTCTTGGAACGACCCTCGCCTACAATGGAACCCAGAGAAATACTACGGGATTGATGTAACAGAAATGTCAATAATGAACATTTGGGCTCCAACCATAACTCTGGTGAATTCAGTAAGTCCTGATGACTTCAACGACAGGTACTACTTCGCTCGCTGCAACATCTATAGTGATGGGGACGTGACCTGCATGCGCAGGGTGACGCACGATGCGGCCTGTAACGTCAACCTCACACATTGGCCTTATGACCAGCAAGAGTGTAGCCTCATCTTCGAGGCCGCGGATTCAAGATCGCTGAACATACAAATTAACTCGTCTTCACGTGTATTTAGCATGTGGGGTGCAGAATATGGAGCCGAGTGGACCATGACAGATTATAAACAGGAGAGTAACTACACATCTAACAAACGGTTGAAGTTGACGTTTGTTGTGGAGCGGGAAGCGATGGGTTTGGCGGCGATCGTGTTGTATCCAGCTATGATTTTGACATTTTTGAATGTGACGATTCTTTTTATGGATGTGCGGCGTCCTGCTCGACTGGGTCTGGCTAGCTTCTGTCTTCTATGTCATCATTACTTCTTGTGTGAATTAGCAGAGAACATACCGAAGAAGAATATGAATCCCCCAAATCTACTTTTATACTACCGAGGTTCGGTTGTGCTGTCGTTGTTATTAATGCTACTCTCGTGTGTGTTAGGTAAAATATGTAGCCTTAAGTCTGCTCCACAGAATTATATCATATCTTTTAACAATTCAGTGTTCGAAAGTTTTGGGAAATATATGGTATTTCCTCAATGGGAGCTCGAGTGTGACTCCGATGGTAAACCGTTTAGTCAAGACTGGACAAAGTTTTCAAATATTATTAATACTGTTTTTATTATTGTCGTAATGGTCACTTATATTTGTCTATATCTTATTTTGATGCCGCAACCTATTCCTATTACATATTGAT

>gene7327

CAGGATGCGCCCCGCGCGGCAGTGCCCGCCGCCATGTCGGGCGGGTCGCGCGCCTTCCTCCTGCTCACCGCGATCCTCACACTACTCTACTCAGGATGGTGTTCCGAAGACGAGGAGCGTTTGGTCCGCGACCTGTTCCGTGGTTACAACAAACTGATCCGCCCAGTACAAAATATGACTCAGAAAGTCGACGTGCGATTCGGATTAGCCTTCGTACAGCTTATTAATGTTAACGAGAAGAATCAGATCATGAAGTCGAACGTATGGCTCCGGTTAGTTTGGATGGACTACCAGCTGATGTGGGACGAGGCTGACTATGGCGGCATAGGAGTACTGCGGCTGCCGCCCGACAAAGTCTGGAAACCAGACATTGTACTCTTCAACAACGCCGACGGTAACTACGAGGTGCGGTACAAGTCGAACGTTCTAATCTATCCCAACGGTGAAGTGCTGTGGGTACCACCAGCTATTTATCAGAGCTCGTGCACAATTGACGTCACGTACTTCCCCTTCGATCAACAAACTTGCATCATGAAATTCGGATCTTGGACCTTCAACGGCGATCAAGTATCGCTCGCTTTATACAACAACAAGAACTTCGTCGATCTGTCCGACTACTGGAAGTCGGGTACTTGGGACATTATCGAAGTGCCAGCTTATCTGAATATTTATGAAGGGAACCACCCTACTGAAACTGATATTACATTTTACATTATAATCAGGAGAAAAACTTTGTTCTACACTGTCAACTTGATCCTGCCAACAGTATTGATTTCATTCCTCTGCGTCTTAGTATTCTATTTACCCGCCGAAGCTGGTGAAAAGGTAACGTTGGGTATTAGCATTTTGCTGTCACTGGTTGTGTTCCTGCTACTGGTGTCGAAGATTCTCCCGCCGACCTCTCTGGTGCTACCCCTGATTGCCAAGTACCTCCTCTTTACTTTCATCATGAACACCGTCAGTATTCTAGTCACGGTCATCATTATCAACTGGAACTTTAGAGGTCCAAGAACACACCGAATGCCGCTATGGATCCGAAGTGTCTTCCTTCATTACCTACCGACGGGGCTCCTCATGCGTCGTCCACGTAAAACGAGACTGCGATGGATGATGGAAATGCCTGGTATGGGCGCGCCGCCCCACGCAGCCGCCCCGCACGACCTGCCTAAACACATCAGTGCGATTGGTGCAAAACAAAGCAAAATGGAGGCGATGGAGATGTCGGACCTTCACCACCCGAACTGCAAGATAAACCGAGCAGCCGGCGGCGGTGAGGTCGGCGCCTTGGGCGGTCTCGGCGCCCTCGGGGGCCTCGGTCTTGGAGGAGAGAGGAGGGAGTCGGAGAGCTCCGACTCTTTGCTGCTGTCGCCCGAGGCTGCGAAGGCTACGGAAGCAGTAGAGTTCATCGCAGAACATTTACGTAATGAAGATCTTTACATTCAGACACGTGAAGATTGGAAGTACGTAGCGATGGTGATCGACAGGCTGCAGCTGTACATCTTTTTCATCGTGACGACTGCAGGCACGGTCGGCATCCTCATGGACGCGCCACACATCTTCGAGTACGTCGACCAGGATCGCATCATAGAAATATATCGAGGAAAATAAATCAAACCCTTAGTTTTAAGTACAAACAGAATTGCTTCAAAACGGCTTCCATAATACTCATTAGATACCTATTTGTATTTTTTCTTAATAATAGGTGACGTTTTATAATAAGTGATGCTAATACGTAGACCGATTCAGCACATATTTACATGAATTTACCATTGAGTTGTTGTGTCGAAGTATAGTGACGTATGAGTAAAATATGTTGCGGCAAACTTACTGAAAATAACCTAATAATAAATTGATCAGGCTTAGTTCGTAAGTATTAAGTAATATAAATAAAATGGCTGTGTTCGAGGTTTAAAAGCACAATGGTTTGTATTTTGTAAGTGTCCTAAGATTAATATACCTCTAGAGAGTAATGTAGATAGTAGGTTTGTAATTATTTATGTTCTCATCCTAAGAAATGAAACTCTTCCATTAATTTATCGATATCACTTTTTATCTTTGTAATTCTTAATTATAAACCCCAAAAACATTTCAATGCCCCAAGTAATTTGTTACTTTTTTTACTTAACTATTTGAAATTCTTAAGATAATTGTAGTATGT

>gene7328

ATGGGCGGGCGGGCGCGCCGCTCGCTGTTGGCGGCGCCCGCGGGCCTGCTGCTGCTGCTGTGCCTGCTCTGGCCGAGGGGGGCACGTTGCGGGTACCACGAGAAGCGGCTTCTGCACCACCTACTGGACCACTACAACGTACTAGAGAGGCCCGTCGTCAACGAGAGCGACCCGCTGCAGCTCTCCTTCGGCCTCACGCTCATGCAGATCATCGACGTGGACGAGAAGAACCAGCTTTTAATAACAAACATCTGGCTTAAACTAGAGTGGAATGATATGAACTTGAGATGGAACACTTCGGATTTCGGCGGGGTCAAAGATTTACGAGTACCGCCCCACAGACTATGGAAACCAGACGTCCTTATGTACAACAGCGCGGACGAAGGGTTCGACAGCACGTATCCAACGAACGTGGTGGTGAGGAACAACGGCTCGTGCCTGTACGTGCCACCTGGCATCTTCAAGAGCACATGCAAAATCGACATCACCTGGTTTCCCTTCGACGACCAACGTTGCGAAATGAAGTTTGGCAGCTGGACTTATGATGGATATCAGTTGGATCTACAGCTACAAGATGAGGGGGGTGGAGATATAAGCAGTTTTGTCACGAATGGCGAATGGGAGTTAATAGGAGTGCCCGGCAAGCGCAATGAGATTTATTACAACTGCTGTCCGGAGCCGTACATCGACATCACGTTCGCGGTGGTGATCCGGAGGAAGACGCTCTACTACTTCTTTAATCTGATCGTGCCCTGCGTGCTCATTGCCTCCATGGCTTTGTTGGGGTTCACCTTACCTCCAGATTCTGGCGAAAAATTGTCATTAGGTGTAACGATATTGCTGTCGTTGACGGTGTTCCTCAACATGGTAGCGGAGACAATGCCAGCGACATCAGATGCTGTGCCCTTACTCGGCACTTACTTCAACTGCATCATGTTTATGGTGGCGTCATCCGTCGTCTCCACCATCCTAATTCTCAATTACCATCATAGGCACGCAGACACACACGAGATGAGTGATTGGATTCGATGCGTGTTCCTGTACTGGCTGCCGTGGGTGCTGCGCATGTCTCGGCCGGGCTCCGCCACGACGCCCCCGCCGGCGCGCGTGCCTCCGCCGCCCGACCTAGAGCTGCGCGAGCGCTCTTCCAAGTCGCTCCTTGCGAACGTACTCGATATCGATGACGACTTCCGGCACCCGCAAGCACAGCAGCCCCAATGCTGTCGATACTACAGGTCGGGTGAAGAGAACGGCGCGGGATTGGCCGCACACAGTTGTTTCGGTGTCGACTACGAGCTCTCCCTCATCCTGAAGGAGATCCGAGTCATCACAGACCAGATGCGCAAGGACGACGAGGATGCGGACATTTCGCGCGACTGGAAGTTCGCCGCCATGGTCGTGGACAGACTGTGCCTTATTATCTTTACCCTGTTCACAATCATCGCCACGCTAGCCGTGCTGCTGTCCGCGCCGCACATCATGGTGTCGTAGCGACCCGCCCGCTTGCGGATGCGCATGCGAAAAGTTCTGTGATACCGCGAATATTTGTTAAGTTGTGATGAGCGAAGTGGCGCGGACGTTGACGCCGCGGCGCTGGAGTTGCCGCCGCCTGCCTCGTCGCCCGCGCCCCCCTGTAGACATAAGTTACCGCTGACTGCCAACCCTGTACGTTCAAGAAATAACTGCCCATCTGACTAATGTCTTCTTATCCCTTGAGAACTTCAGCGATTGTATACCTTTTCTTCCAAGAATACTATGACAAACGGTCGTCACGCTCAGTGGAATCAATCCCGTACTCTAGACCCGGTTGATCCCTTAGGGTATGTACTCGAGTTGAACGAGCGTTTCCTTGACAGACGTTCCGTCTCCGGAACGATCCCCTAGCGATAAAGTGGCAGTACGTGCTACACAGGCACCAGCGCCGCCACGCCGCCGCGGTGCAAGCTCTGCGCTAGAATAGTCACCGCAGCAAGTGGCCACCCACTAGACAAGACTGCGGCAGAAAATATTTGCACAAATACGTCTTCCTTCTTACCGTTGAACGACCTGAATTCGCATTTAAATTTAAAACTTTGTTAGAACTTCTTCGATTCTTGAAATCTATTGTACAGTTTAGAGTTTGGGCGGTGAAACAATGGCCCTTTGTTTCCTTCTTGTTCGATTCCATGAATCGTGGTTATAATCCCTAGTTTTATTTTCGGATATATTTGTGTCAGTAGCTAGTATAGAACTTTACAAACAATGTTGATTCAATTGGTACAGGTTGTGATATGCCTCGTTGTGAACGGGTCGATATTGTTATAAATGGTAAAATACCCATGGCTATAGCTTAATAATCGTTCGTTAAAAGTTGTAGTTAAACAAATATTATTTTAATAAAGTCATATCTGGGTCTTTCGGAACGACTTTTACAAATAATAATTAAATTACATATAAATATCACGTTTGTACTTCTTTCCATACAGTTACAGTAATTCGTATGCTGGAAATAATGTTAGCTTCTAAAATTTTCTTCTTCGAAAATTAATTCAAAATGTACGACCATCGTATCAGACGATTACATGTAATATATAACTCATTTTGTAAGATATACAACATTTTATAAGTACAAAAATTGTAACATGAACCGGTTTTTCGTTACATAGAGGGTATACACAAAGGTGCCTATTTATTAACAGAAGAGACGATCAGTTGATAATACAGGTAGACTATATCCTGAGCATCTGACCAGTCGACAGTCCTGCCGCTCGTCTGCCACACTCCGACACATACTTGCAACACGTTCAGTTATCGGCTCCGTCATCATCTATTGTTGCGTTTAGTTATTCTAAGTCATCGATGTTCATTTCACCCAGAAACGTTGTGAACCTCCTAAAAGGAAAACGTAGTGTAAACAGCGAGAATGTGCGGGTACACCCGACAAGCGAATGTACCCTCGCAAAGCTCCTGTAATGTTTTCCTTTTTCGAGGCAGTGCTGTGAGTAATCTTAGACGGTCCGATGGAAGTTGCGGACCGGATATGATTATAAGTCAATGTTTCTGCTCATCCTTTTATTTACTGTTATATCTTCTTACCATTCGCTAGAGGTTGTATGACGACCCGGACGGTGGAGCCGCAGCCCTGCAAACGCGGGCTTCATCTTTGTACTAGATGGAATTTTGTGCGGCAGCCCTCCGTCGGCAATGGGACAACCCATTGTCCCCAAGATTTGTTCAATTGTTAGGGTTAACTCTGAATTGCACTTTGTTTATTAAATATAAACGAATGA

>gene7363

ATGTCGCCTATGTGTGCGGACCCAAACTATCGGTCAGCGGTGATCAACACCAACGTGATAGTGAAACACACGGGAGAAGTCACGTGGCTGAGCCATGGGATCTATGTGTCTGTGTGTGACATCAACGTCGAGCAGTTCCCGTTCGATGTCCAGCTGTGCACCATGAAGTGGGCGTCTTGGACGTATGATGGATTTCAGTTGGATTTGAAGAAGCAATTTGACGAAGGGGACACAACGAATTACCAGACGAACGGGGAGTTCGACCTAGTGAGCTTTGAGGCCATTCGCCATGACCAATATTACTCCTGCTGTGTGGAACCCTACCCTGACATCACATACATCATCAAATTGAGGAGAAGACCCATGTTCTATGTCTTCAACCTTATTCTTCCCTGTCTCCTCATCAATGGTATTGCACTTCTAGTGTTTTACGTGCCGTCGGAGTCAGGTGAAAAAGTGACCCTGGGCATAAGTGCACTTCTGTCTATGACCGTCTTCTTGATGACTATAAGGGACACGTTGCCGCCTACAGAGAAAACCCCACTTATTAGTTTGTATTATGGAGTTAGTACGTGTTTAGTGTCATTCTCGGCTTCTCTATCAGTTGTCACGCTCAATATATCTTACAGAGGAGTGAGAGGGCAGCCGGTGCCGGCGGTGCTGCGCGAGCTGGTGCTGCAGCGCCTCGCGCGCCTGCTCTTCATCAACCTTGACACTGACGGTGCAAAGGGTGACGCCGGCACGGTGACATCAGCTGGGATGCAGGTGAACCCGCGCACAGGCTCCAGGCTCAAGGCGGAGGTGCGCTGCGATGGAGCCCCACTCACACCTGCCTCACCACGCTTCGCTCGCCACAACCACAACCATTTAGGTGGGACACAAGCCAATATGGCGTCTGTGCCAGGAGGTGGCGCGGGTGGCGGTGCAGCGAACACGCCAGTAGAGACGGGCTGCGCAAACACATGTTCACGGTGCGCGTGTTGCGGTTGCACGTTGCGCGAAGCTGCGGCCCGCCACGAACAGCGAGTCGCCGCCAACGAACGATTGGAACGTGCTAATTTGGAGTGGAAACAGGTGGCAGTGGTAGCGGACCGGGCACTCCTAGCTGTCTTCGTGCTCGTCACAGCAATATCCACAGCGGCCATCTTGTTACCACAGTTGAACAACTTGCACGTCGGCAACACGCCTCTTAAACCTCCCATTTAGAGGCACTACAGAACCCTAGAATAAATGGAAACAAAGTCTTCTGCCAACGAATGTGTTAAATGAAATGTGCCAAACTTCAATTGAGTCACGCACTGTTGTAGATGATAAAATGTAAGTGATTTATAAAAGGTTTATACCACACTGGTATGAGGCCAAAGGAAATAAGTACACATTGGTAAGAAAATAAGGTTTATTTTTTCATTTGCTTGCTGATGTGGAAGAGAGTGTCATGGAAGAATGTGAGTGGTAAATTAAGCGATGTTGATATCCTTGATGTTATAGGCGATTGATTGTCTTAGAGCTCAGTAAATACTTTACTCGATATCTGTTACTATTTCGTTTGAAGCTTTAGGAAGGAAATTACATAAGGGATAATATTTCCGTACAGAAACGACTCTTATTGTTCCAATTTTATTGTTGCTAAGAATAAAATTAGTTCATTTTACATACTTACTTTGAGCATAATTTTCAGTACATTCAATGATGAAAACATATGTGTTTTTAAAACAATTATTGTTTTAGAATAATTCAGAAAAGTCACTAAAGATCTATAATGAGGAGAAATTAAGACAACTGTTAGTGTAGTATCGGTAATTCTGAATGGAAATTACTAAGGTTTAATTACAGTCAGTAAAATAATTTGGTCAGCACTGCATGGGTACGTATTGCTGTAGTAAACTGTAAGCTTTAATGTTAATTTACTTCCTATGTGAACTGCTGTGAACTAAAGGGATTTTTGACTATCTATATTTTTAAACTCATATTTCCATTAGTTGCGGGATCTTGTTGTTTCTTATACCTTGAAGAAAACTATTAAGAGATAGAGGTAAGGCTAAGTAAAGGGTTCAA

>gene7613

CGCTCGGGCCGGCCGCACGTTGTCTCAGGCCGCATGAGCGCGCCGGCGTGCTAGCGCAGCGTGCGCGGGTGTGGTATGCCCGCGCGTCGCCGCTATGGCCCCTATGTTGGTGGCCTTGGCGCTGCTGGCTTTGCTGCCCGTATCGGAGCAAGGTCCCCACGAGAAGCGGCTGCTGAACGCTTTGCTCGCAAACTACAACACGCTGGAACGACCAGTCGCGAACGAGAGCGAGCCGCTGGAAGTCAGATTCGGCCTCACGCTGCAGCAGATCATTGATGTGGACGAGAAGAATCAACTACTTATAACCAATATATGGCTGTCGTTGGAGTGGAATGACTACAACCTGAGGTGGAACGACAGCGACTATGGCGGGGTCAAGGACCTCAGGATCACACCCAACAAGCTGTGGAAGCCGGACGTGCTTATGTACAATAGTGCTGACGAGGGTTTTGACGGGACATACCAGACCAACGTGGTGGTCAGAAACAACGGCAGTTGCCTGTACGTGCCACCTGGCATATTCAAGAGCACATGCAAGATAGACATCACGTGGTTTCCCTTCGACGACCAACACTGTGATATGAAGTTCGGTAGCTGGACATATGACGGGAATCAGTTGGATCTGGTGCTAAAAGATGAGGCAGGCGGTGATCTATCGGACTTCATAACAAATGGGGAGTGGTATTTAATAGGAATGCCAGGCAAAAAGAACACAATAACTTACGCGTGCTGCCCAGAGCCTTACGTAGATGTCACGTTCACCATTATGATCAGGAGACGGACGCTGTACTACTTCTTCAACCTCATCGTCCCCTGCGTGCTGATCTCATCCATGGCTCTCCTCGGTTTCACTTTACCGCCAGACTCTGGAGAAAAACTTACTCTTGGAGTCACTATTCTTCTCTCGCTGACGGTGTTCCTCAACCTGGTAGCTGAGACCCTGCCACAGGTCTCCGACGCTATCCCCCTGTTAGGGACATACTTCAACTGCATCATGTTCATGGTAGCGTCGTCAGTGGTATTGACAGTCGTGGTACTCAACTATCATCACCGGACCGCAGACATACATGAGATGCCGCAGTGGATAAAATCAGTGTTCCTGCAATGGTTACCATGGATCCTGCGAATGTCGAGACCAGGGAAGAAAATCACAAGAAAGACAATCATGATGAATACGAGGATGAGGGAGTTGGAGCTTAAAGAGCGGTCGTCGAAGTCATTACTGGCCAACGTACTTGACATAGATGATGACTTCAGGCACGCTCCTCCACCTCCTAACAGTACAGCATCCACTGGGAATTTAGGGCCAGGGTGCTCAATATTCCGCACGGATTTCCGTCGGTCCTTCGTCCGTCCGTCCACTATGGAGGATGTGGGCGGCGGGCTGGCCAGCCACCACAGGGAACTTCATCTCATCCTGAGGGAGCTGCAGTTCATCACTGCAAGGATGAAGAAAGCAGACGAAGAAGCTGAGCTGATCAGCGACTGGAAGTTCGCTGCTATGGTTGTTGATAGGTTTTGCCTGTTCGTGTTCACACTTTTCACAATCATCGCAACAGTGGCTGTCCTGCTATCGGCACCGCATATCATCGTGCAATGAACCAACTACTAGGTGCCTCGGGTGTACCGGCACAAGTATGAGAGAACTAATTATTAGTATGCCGATTTGTAATTATAATTAATAATGTAATTAAAATAAATACGTGTTTGAAGCGCACACGTCTCCTTAAAAGTCTTAAGACATTAAATTACGGTAACTTTACATTATTATAGTTAAGTCAAATGCTAATGGAAATTTCAGCCGGAGCTAGGAATTACATTATGAAGGTCGTTCTGTTGTTCGTGTTGTACTAGTGTATTGTTTGTAGCAAATAAAAGAAAATATAAAGTTTTGTTTCTTCGAATTTACAAAGCTCCTTGAGCTCAGACTCAAATTCCCATTAGTAGTTATTAAAAAAAATGTGATGTTGACTAGGATGTTATACAGATAAATGTTGACGTGTCTAATTTGTTAAAAAACAATATTAATAACTTTATCTAAACGGTTTGTATAAACGAAATATTAATGAGGGTTAATTTGATGGAAAGGAATGCTTTGCTAGCACAGAGTTGCATTAATTTAGAAAAAGACATCACAAAGGTTTGTCTGGACATTGGACTCTTTGAATGAAAAATGAGGTTTGGTCAAAAAAGTTTGTAAAACAGAAAAGTTTGGTTCTGCGTCTGTGCTGGAATTAAAATAATTGTCTTAAATTGAAGGAAAAGAATGTCCTTTGAAAAATTAAATTTCAATCGGTATCCTTTTTACATGTAATGAGGGAACACAGATGATGACAAAACCTTAGGGTATTAAGTAATGTACATAATGGATCAAATATCGGTAGAGTGAAGAATAGCTTAACGATTTAAGATTATTCCATTCGATATTAAAATTCGATTAGCGAAAATCGCTACGTTTACATCGTTACATATCGATTTGAATCGATATGTATTATATAAATTGGGACGTTAGTGGAGATTGTGAAAGTAAATATTGTAATTATTAAATGAATATACTACGAAAATTTAATCCAGGAATTGTTAAACAGTCATGGAATTGATAAGAATTCAACATTAAATACGGAACCAAGGATAGACTAGGTCTAGCAGCACGGGGTTGAATAAAAAATAATTAGATCCGACCGCGGAAGGTAACAAGTATTCAGTAGAGAGTTTTTGTTAATAAACGTAATATCAATTAAACTGAAAACTGTTAATATCTCGTATCGATAAATGCTCATTTAATATGAATTGTGTAAAATTATGAAAGTGTTAGTATATCGATATGAGAATAAGTCCCTAGCAACAGAAATAGCAACTGATATTAGACGTCGGTTTGTGTTAATCTAATGCTTAGAAAATATTACATCGCCGTTAATATCACTAACCACCACGTAATATGATATATATAATGTACATACAGAATAATGCAGTGTGTATGTATTTGTATATAAATTTTAGAAAAAAATCGGAAAAAAGACTAAAATATCGCATTTTTCACTCTAGTATTTATAACAACCAGTCTTATTGGGCAGGAATAGTACACTATTACACGTAAGTACTAGATACTGTCTTTATATTGATATCATCTCGATAAGTCAAGCGTTATCTGATTCATTTCAAACGTAGATCGATGGGAGGCGATCCGAAATATTTTCGCGCCCAATAAGACGGCAGAATGTCACTTCGACGAATGTTCAATTGAAAAACTTTTTTGATTGAAATTGATTGTTATCGATTCCAATTCGTCATTGTCTACTCAATTAGAACCTTTTAGCACATACTGTAGCATCTATATAATAGTCGTATTATTTAAAAAATAAACAATTAAAGTCTTTATCTATGTCCGTTCCTCGATTTCACTCTTATAGATGTCGTGGTAGCCTAGTGAATGTTGTGTCCCTTGATTTAAGCTAAACTTATAATGAAATAACTATTGTTAAGGAAGCTCATTAAAACTAGATATAAAATTTACGCGATCTGAGTGTTTTGCATAATTATTTAATATTCTTTTTGATATAGAACAACATGAAAATGTGTTTACACGAGCAGTTGTAGTATTTTCTTGGATTCATAATTACGAGTCTTGACGGGCTCAGAATGATTGAGTCTCAAGAGAAAAATACAGCGTGTTCTGAAATTGTATTTATCAGAATAATGTATCATTATAACTTAATTATGTTAATGATTCCTGAGATTATGAACGTTGTCGTTTTGCAGAATTTATTTCAAACAGTGTGGTGCTGAATAATATCAGGTGTCGACATTTTCTTTTCGTATTTATAATTGTTATAAGTATAATTTAAAATTTCAT

>gene8227

GGAGTGGGCAAGCAAAGTTCGTTCAGTAAAGCAGAGGTGCACATTACGACACTTATTGCCACTGTACAATGTCCCACCCACTTTTCACTATTTATGTTATGAGTCCCATGTAATAAGGGGTGACTCTATCACTTCCCGTTCCTGTAGCATTTTGTCCGGGAATCGAACCCGAGACCCCTTGCCAGGCAGTCGCACTTATGACCACTCGGCCAACGAGGCGGTCACACACATACTTACCTATAGTAACATACAAGATTAACAAGTGTATTGTATTACACCTAGAGTATAAAAACCTCTTTTGTATTCACAAAAGGTGGTTCTGTATTATGTATTCACAATTCACAACTTTAGCAACCGTTCTATGAAAAATCAATAAAAAAAATACATTTGCAACGCTTATCTCAGTGGGCGTTTTCATATTATATGACATAACAATTGATATTGAAAGCAGTATCGTAGAAACCATGACGAAGTTCACTTATATTTTATTACTAATGTATCTTTTAAAATTTAGTCTGGCCAAAGACTGTGCCAATAATAAAAGAGAACCAGAGTATGTCACGTGGCTTAAAAACCTTTTAGAAGATATGTGTCCCAGTTCAAAAAAGCCTCCGGCAGGAAACAATACAGTCTATGTCTTTATGGATGTAGGAAGAATCAGTTTCGAAGCCGATAAAGAAGTATTCACTGCTCAAACCACTTTAAATATTACTTGGAGAGATGAACGACTAAAGTGGGATCCAGACAAATACCACGGTATCAAGGACACAGAAATAGGAGAATATTCATCTTGGATTCCAAAATTTGAACTAATCAATGTGGAAGATTTCTTCAACTTTGAAATATCGACTTTATATTCTTGCAAAATCTATAACACGGGGGTCGTTACATGTCGACACAGAATGCTGCATGAAACATGGTGTGGCGTCAAACTCACTAACTGGCCTTACGATGTTCAAGAATGCAGTTTGGAATTTGGTCATGAGTACATGACAGTAAACCTTAGAACAGCCATATCTGACGAATATACAGCTGGATGGCACGTTTCTGAAATAAAACAAGAGAATAATGCGAGTGGCAAGCCAGGAGTGACAGTGACTTTGGTGCTGGTGAGGGAAGCGGCTGGATTGTCTGCGATCTTCATATACCCTGCTTTAGTGTTGACTGTGCTCAATATTGTGACGCTGTTCTTAGACGTGAGACGCAATGTAAGGCTAGCAGTTGGCTGCTTCACTTTGTTTGGACATTATTATTTACTGATTCAGTTAGACATTGACATGCCTAAAGGTAACGCCTTTACTCCTACTATACTGTTGTACTATCGCGATTCTATTATTTTGACTATTATAACCATTCTATTGACTTTCTTTCTGACCAAACTTTGCAGTGTAAGTTCAAAGCCACATAATTATATCAAGATAATTAATAATTTTGTGTGTGACAACAAGTATTCTGACTATTTAGTGTTTCCTAAATGGGAATCTGATGATGTCACCGTTGATTTAAAATCTTCTAATGAAGATTATGTAATTTTTGCAAACGTAGTGAATAGTGTTTTTATGTTTTTGATTGTTTTCGCTTATTTTGGTTTATTTATAAGCAAGATTCCGAAATAGATTATACGAATAATGTAAGTAATAATAAAGTTCGTTATTGATATCATTTTTTAGTTTAATAAAAAAAGTAAAAGATGTCGATGCCTCCATCTCTATTATGTTAGTAATATCAGAATATTCAGAATGTAAGGCCGCTGGATATGACACAAAACTGTGTTTAGCGATATAAAACTAGGTAGAGAGTAAAGCGACACATCAAATACAGGTAAAGGTTAAAAATAAAAGCACATTGCTAGTGATAAGTGCGCTAGACCGACTAAATCAAGACTGTAATCAAAGGATGACTGTAATGAAGGAGGAATGTAATCATTCCGTGATTGCAGAAATAAGGTACAAGAAAGACGATGTAATATATCAACCGCACAGAAGGCCGCATCAATGCAGCGCAAGGCGGTAGGCCGTAAGATATTTGTCATTAAATATTTTAACGAATA

>gene8228

GTTCACTTACATTTTATTACTAATGTATTTTTTGACATTTAGTCTGGCCAAAGGCTGTGCCAATAATAATAAAGAACCAGAATATGTCACGCGGCTAAAGAAACTTTTAGAAGATCTGTGTCCCAGTTCAAAAAAGCCTCCGGCAGGAAACAATACAGTTTATGTCTTTATGGATGTAAGAAGAATCAGTTTCGAAGCCGATAAAGAAGTGTTCACTACTCAAACCAATTTCAATGTTACTTGGAGAGATGAACGACTAAAGTGGGATCCAGACAAATACCACGGTATCAAGGAAACTGAAATAGGACACTTTTTAGGTTGGATTCCAAAATTTGAACTAATCAATGTGGCAAAATTCCTCAACCAGGTATCGACCTTTCATTCTTGTAAAATCTATAACACGGGAGTCGTTACATGTCGATACAGAATGTTGTATGAAATACGGTGTGGCGTCAAACTCACTAACTGGCCTTACGATGTTCAAGAATGCAGTTTGGAATTTAGTTATGAGTACATGACAGTAAACCTTAGAACCGGCATATCTGGCGAATATACAGCTGGATGGCACGTTTCTGAAATAAAACAAGAGAATAATGCGAGTGGCAAGCCAGGAGTGACAGTGACTTTGGTGCTGGTGAGGGAAGCGGCTGGATTGTCTGCGATCTTCATATACCCTGCTTTAGTGTTGACTGTGCTCAATATTGTGACGCTGTTCTTAGACGTGAGACGCAATGTAAGGCTAGCAGTTGGCTGCTTCACTTTGTTTGGACATTATTATTTACTGATGCAGTTAGACATTGACATGCCTAAAGGTAACGCCTTTACTCCTACTATACTGTTGTACTACCGCGATTCTATTATTTTGACTATTATAACCATTCTATTGACTTTCTTTCTGACCAAACTTTGCAGTGTAAGTTCAAAGCCTCATAATTATATCAAGATAATTAAGAATTTTGTGTGTGATAACAAGTATTCTAACTATTTTGTGTTTCCTAAATGGGAATCTGATGATGTCACCGTTGATTTTAAATCTTCTAATGAAGATTATGTAATTTTTGCAAACGTAGTGAATAGTGTTTTTATGTTTTTGATTGTTTTTGCTTATTTTGGTTTATTTATAAGCAAGATTCCGAAATAGATTATAAGAATAATGTAAGTAATAATAAAGTTCGTTATTGATATCGTTTTTAAGTTTAATATAAAAGTAGAAGATGTCGATGCCTCCATCTCAATTATGTTAGTAATATCAGAATGTAATTATATTCAGAATGTAAGGATACTGAATATGACACAAAGCTAGAGGTATATTTAACGATATAAAACTAGATAAAGAGTAAAGCGACTCATCAAATACAGGTAAAGGTTAAAAATAAAAGCACGTTGCTAGTGATAAGTGCGCTAGACCGACTAAATCAAGACTGTAATCAAAGGTCCATTGCATGATTGCAGAAATAAGGTACAAAACAGACGATGTAATTTGCAGCTGACATGACAACCAGCACCACCCTGCCTGGACAGTGGTGATGAAGAAATGTTCCAATATCAAACCATTGGACAGAATTTTCGATTGAACAAATATTGAACTGTACACTTGTCGTTTATTGTTATCTTATGAGCAGAACAACCAATAAATTCAAAATCAAATCATTCATTTCAATCAAACCATATACTAGATCGAGCTCAGTAGCATGCCAATGCCAAGGAAAGGCCTATGTCCAACAGTGTACGAGAACAGGTTGTTGATGATATACTACATACATACATGACGAAAACGATAAATATTTTAACG

>gene863

GATGAGGGCCTTCATAACTGCTGTGAGCATGGCGCCGCGCGGTACTTTCTACGCACTGTGTGCACTGTTAGCACTAGCTGCGCCCCTAGTAACTTGTGACTGCCCACCGGAACGAGAAGGAGCTACGCATGATGAAGGCAAACTTCACAAGGATTTGCTTTGTGCGTATAATTACGACTTCCGGCCAGTGAAGGATCACAAACAATCCCTTACCGTGAAAGTAAGATTCGCAATAAAGTATCTTAGTTTCGATTCTTTAGAAGAAACGTTTACTTTGCATAGCTGGGTAGCGATGATGTGGAAAGATGAGTTTTTATCTTGGAGACCCGAAGATTACGGCGGTATAAAAGAAACACAGATGGAAAGTCATGAAATCTGGACTCCGAGGATGGCGCTCTTCAACGCCGACGCCTCTATGTATCAGTCCGATCAGATCTACACGACCTGCATGGTGTCGAGCGAGGGCGTAGTGACATGCGTACCTCACGTCGCTCACTCCGGGATTTGCCGGACGTCACTGCGTAGCTGGCCCTACGACGTACAAAACTGTACACTATATTTTGGGTCGTGGATGAACACTGGGGAACAAGTCAACTTTACCTTTTACAATAAACAACCCATCGTAAGGGAAGACTATCAAGATGGTCCAGGCTGGAAATTGCTGAACGTGGTAAACGAACGTTTACCAGGGAAATACAGCTGTTGTCCGAACAGCACGTACCCAATGTTAAAATATACATTCGTAATGAAGAGGATAGCAGCGGGACCAGCAGCTATCGTCGTGGTGCCTTCCATTGTGATCGTACTTCTGACTCTGGTGGCACTTGCAATGGACGCTAAAGACAATACTCGACTCATGTTAATTTGTTTCAGTCTGTACGGACATTTCATGTTTCTCACAGAAATTGGTTATGATATTCCGAAACACAGTGCGGACACACCGATCATATTACTGTTCCTTCGCGATTCGATGATAGTGACATTGGTTGGGATCATAGAAACCATATTTTTAATGTATTTGAGACGACGAAAGGTGCCAGCGCCAGGTTTTATAGTGTCAGTGAACCGGTTAGTGGCCAATGGTCCAGGTAAGTATGTTATTTTCACCGAGTTTGACCCAAGTGAGATGGATACAAAAGAACTGGCCGGTAATAGCTCTGATATCACTACCGACAAACCGCCTGTTACATTCGACTGGGTACAATTTGCTATTATATTGAACAGAATTAATTTCATGGTAATCGTCCTCGTTTATATTATACTAGTTGGCACCTACATTCCACACGATGATTAAATTTTTATAACACCACCCACCAACCACCATGTTAATAAGTTTGTGCCAGAGCCATTAAAGTCATTCACATTCCTAATTTCTGTATTCTAAAGTTAAAATAAATAAATACATAAAACCAGA

>gene8842

GCAACGACGCGCGTGTCGCCTCCGACACTTCCGCCCCGCGCACCTAATCCGCTCATTTAGCCAGCAAACGCCGCATACGGTGTGGTGGCGGTGGCAGCCCTGCGGCTCCGAGCCCCCGGGGCTGATGCTCTGACGAGCCGGGCGCCTCCGCCCGAGCTCCTTCGATCCGCGTCCGACCGGCATGTGATATCGCGCCGCGCCACTCGACAAAATGATTGTGTGTTCAATTGTTTACGTTAACGATAGTTGATAACCGGGAGGCCGACGCTGGCATGCTAATTCGGACTGTTGGTGGGGAGTCGTGGACTGGTTGTCGTGGTCGCGCGGGCGTTGGACGCGCGGTTTTCTCGCGCGCGGGCTGCACATCGCGCGGTTCTGGCGGGCCAGCGCCATGCGGTAGCGCGGCGGCGCGGCCGAGCGGCGGGCGAGCACTCGTGCGCTCTCGGCTATGTGCCCCCCTTGCGCTACACTGTAAGTCTCGATCTGTCGATGACGCAACCAAGGAGGACCCATGTCTTACTGGGGTGGTGTTAGCCTAAGATGGTGTTGGCGCTGGTGGTCGGAGTGTTGTGCGTGTGGGGCCGCCTGAGCGACGCAAACCCAGAAGCGAAGCGGCTATATGACGACCTGCTTTCCAACTACAACCGCCTCATCCGGCCCGTTGGCAACAATTCTGATCGACTCACTGTCAAGATGGGACTTAGGCTCAGTCAGCTCATCGACGTTAATCTTAAGAATCAAATCATGACAACGAACGTGTGGGTAGAACAGGAATGGAACGACTACAAATTGAAGTGGAACCCTGACGACTACGGCGGTGTGGAGACTCTGCATGTACCTTCTGAACACATATGGTTGCCTGATATTGTATTGTACAATAACGCCGACGGCAACTACGAAGTGACAATTATGACGAAAGCAATTCTTCATCACGACGGTAAAGTAGTGTGGAAACCACCTGCCATTTACAAGTCCTTCTGTGAGATTGACGTGGAGTACTTCCCTTTTGATGAGCAGACCTGCTTCATGAAGTTCGGCTCTTGGAGTTACGATGGCTACATGGTGGACTTGCGGCACTTGAAACAGACGCCAGACTCAGACCGCATCGGAATGGGGATTGATCTGTCGGAGTACTACATCTCAGTGGAATGGGATATCATGCGAGTCCCTGCCACGAGGAACGAAAAATTTTACTCCTGCTGCGAAGAACCGTATCCTGACATCATCTTCAACATCACTCTCAGAAGAAAAACTTTGTTCTACACGGTCAATCTCATCATCCCTTGCGTTGGGATCTCATTCCTATCAGTCCTTGTATTCTATCTTCCTTCAGACTCCGGAGAGAAAATCTCGCTCTGTATTTCGATTCTTCTATCATTGACCGTGTTCTTCTTACTGTTGGCAGAAATTATTCCTCCAACTTCGCTAACAGTTCCCTTGTTAGGAAAATATTTGTTGTTCACTATGATGCTAGTTACATTATCAGTAGTTGTCACTATCGTGGTGCTAAACATAAATTTTAGGTCGCCAGTTACCCACCATATGGCCCCGTGGGTGCGGAAAGTCTTTATAGATTTTTTACCCAAAATATTATGTATACAGAGGCCAGAAAAACCGCCCGATGACGACGATGATAACGATAAACCAAGCGAGATACTCACGGATGTCTTCGGCCCGGATGACATGGATGGAAAGTTCAAGGAATGGGGTTGTGAGGAATATGAACTGCCAGGAATGCCTCCTTCCCCTCCGCCTCCACCAGGAGGTGACGACGAACTATTCTCCCCGCCTCCTGGATCCCCGTGCCGCTTAGACTTAGATGATGGTAGCCCATCTCTAGAAAAACCGTACGTTAGAGAGATGGAAAAGACAATAGAAGGTTCTAGATTTATTGCACAGCATGTCAAAAATAAGGATAAGTTTGAAAGTGTGGAGGATGACTGGAAGTACGTAGCGATGGTGTTGGATAGAATCTTCCTGTTCCTGTTCACGATAGCCTGCGTACTGGGTACGGCGCTCATTATATTCAGGGCGCCTACCTTCTACGACAACACGAAGCCGATAGACATTCTGTACTCAAAGATCGCGAAGAAAAAGTTGGAGCTCCTGAAGATGGGCTCTGAAGGTGACCCGGGTCTCTGAAATTCGTCTTACGTCTTCGGCCACTCGCCGCCGAAGACGTTCTGGGAGTTGATCGTCATGTGGTGGCACGGATGATCTATTTAAGACTCCCAGATGGGAATTACCAGGCGAGAACTATGTGTTTCTGTGCTGTGTGTGGTGCCAAGCGCCCGGTGCGCCAATGGACGTGTACACGCCGTAAGCGTATCCTAAGCCGTCTTTTTATATAGAATCATTAACTCGATGTCAAAACTGTCCGTCGATGTTCGATTGTCGCAGCGACCTCTAGTAACGGCCGTTTTGTGTTGACACCGGATCTGCCGGATCTGCCATAACTATTTTTATATTAGAATCGCTGTATTGAACGTTTATACTTTAAGCGAAACGTTTTCTTTTATAATAATCAACGGAACGATTGTTTTTCGAGTAATAATTTGATACGAGCGCACAAACATTGTACAGTTTATCTTTAGGTGGTGGAGTTAATTTCTTGCGATAGTCGGATTCATTTTCGAGATGGAAACTGACATGATAATGTAATAGATGACTAAATGTAAGATTTACTTCGAATAAATCGAGCTCAAATTCGTAATATGTAAGTATTGTAAAGTGGTAATATTCAATGAAACGTCGATTAGAATCAAACATTTATAATAATAGTTACGTGCAAACGTCCTCGATGAGAGCCGGACTATGTAGTATATGTATTAAATTATTATGAAATAAATTTGAATTATACATTATATAATATTTGATAAACTGCGTACTCGCGTAAAAATCTTAAGGACAATATCGATACTGACGAAATGTAATAATTTCCCTTTAGTAGTATGTCATAAGGGATTGTAGATACAGTTAGGTTAAAAGCCTCAATCATCAGTACTGTAAGACACTTATCATAATGCCAGATAGTTAGTCACATCGCGATCGTGATAATTCTATGATTAGATGTATTTGTGTAAAACAATTGACTGCCAATTGTAAAAATTAAACATTTTTATAAAGATCGGGTATTCTTAGGTAGTTCGATAACTTGTTTTATTAGATTTTTTAAACGGAGTGCCAATCCCACCGACGTGACAGATGTACAAGTTTGCAATTCTACAATGTTCTGTTTCCACGTGCGCTGACTTCACTGTCGTTAGAAGTATCTTTTTGATTCGCTTGAAGATATTCTGATGTATTTATAAGTAAAACGAATGCTTGCCGAAAATGTAATGACTGATCAATACACATCTTAAAGCTTAAGTTTGGAAGCGCCCGCTTGTCCGCGAACTACCGCAGCGAGGCGTGATGTCACACTTCTTCCGTAACCTTAAATATAAACAGTATTTTCACTATAAAACCTAGAGTACAGGGTAAATTTAGTACACGAATGTCTCCTGATTTATATTTCTCACAAATTTTCACTGAATGTAAAGTCTTTACTCACTTATCAGCACTTTACCACTAACACTATTAGATATAATAAATCATTGTTGAGGTTTGTGACTTTAGTACCTAAGTAATTTAGGTAACATTATATTAATTTGTACCTAGATTAGTCTCGTATTAGATGTTCCATTTCGTTCTTGAAATCATGACTCACGATTCATTTGATAGTCATTTATGATTTGAGACGAACGCATCAAGCGTTGTTACCTCACTTCAATCCATTCTGTAGGATATTTGTAGCAGTCTAACGTAATAAAAAAAAAATATTTCGAGCCGCGTATTTGATATGTCAAAGTAAATCATGATAATGTTTATTAGGTAATTACTTAGATAAAATTCAAAGTTATTGATATTAATGATGAATAAAATCCACAGCGAGCAGTTAGGAAAACAGGGATGGATAATGTAAAATAAATTAAAGAAAATCAAATTGTTTTGTGTGGTTCGTCTTCGTAATTATAAGTGTTATTGAATGTGTATAGCTGTTCGTGTCGAGGGTAACGCATAAAAATGGCTAGTTTAGAATTAGATCATACGTAATAAATTAAAGAAAACGGTCTAACGTATTAGACGTCGTGGAAGCTCGCTAAAGGTTGAAACATGTTAAATATTAGCAAGATACGGCAAACTGCTCTCACACACGACCTGGCTCTAAAGGAATTATCGGAAGGAATTGTAAGTTGTCCTCAACTATGCTGTTAATTTTTCTAAATGAATCACTCACGTGGATTAATAAAAAGTAGGATCGTGCTATTGCACCACCGAAATACACCAATTTAGTGTAATAATAACTATTTATCAAATAATGTTTAATTAAGTCCACCGGCTTGGCACACTATCCCGTATAAATCATCCGAACTTTAATTGTAGATATTTGCTAGATAAAACGGTCTGTTGTTATTTTTATATGAATGCAAAATGATGAATAGTTAGATTTTCTATAACACCATAGGGTAAAGTGGTAGGTTGGAGTTTGACGTTCAGTTTGAGGTGTTAGGGGTTAAGTGAACAATTGTATATATTTGGAGCGTGAACTATGGAGCTAAGTGTAGATTGGATCGGGTTCTATTTGCAACGACACAGTATAAACCGACTGTAAACCTTCTGTTGGTGTACTGCAACATTTTCAATGGTTTAATGTTGAATTTAATGAGCTATGTCGATACAACGATTTATGGCCCATCTTGTGGTGTTATTTCATTCAGTGTTCGTGGCGGACTCTGATTTTTGTACTGGTTTATACATTTTGTATTTATGTATTTATTTACGCATATTTATATGATACGAAGTTAGTTTGAGTACTAACTACCAGGTGGCATGCATAGCACATTAATTAACTGCACTGTTAACATGCTTTGAGCATTATGCCAATTAGACTACGAAAATAAATATTCAAGATATAACTTTCGGGCTATGATCCGCCTGGAAAAGGCACCATTTTATACTGTTCCTCAAAATACCTTAAATCTACGTATATTGTATATTTCGAATTGCATTTATTTATTGGAAAAAATAATATT

>gene8844

CGCTGCAGCCTCTCGGGCGTAGCTGTCAAAATTACGTTTACGAAAAAATTGCGATTCCCTTACTTATTGCCGATTTCCGATTGTTATAATTGAATATATTGGATAACAATATGCAGATTTAAGTTTGTAATAGTTGTATTCGGTATTCGGTAGCCGATTAAAACCTAACCTATTTCATTTCGGTCAGCGTCGGTGGTATATGGTGACAATAATCTTCAGTGTTATTGCGAAATGTCCCGGACCTTTCTTCTTGTCTTTTTGTTTATTTTTGGCGTTTGTTATTGTAATCCTGATGCGAAACGCTTATATGACGACCTACTGAGTAACTACAATAGGTTGATACGTCCTGTTGATAAAAACAACAATACTGTGTTGGTGAAACTTGGCTTGCGGCTATCACAACTGATTGACTTGAATCTAAAAGATCAAATTCTGACGACGAACGTGTGGTTGGAACACGAATGGGAGGACCACAAGTTCAAATGGGACCCCTTGGAGTACGGGGGCGTGAAGGAGCTCTACGTACCTTCGGAACACATCTGGTTGCCTGACATAGTACTTTATAACAATGCTGACGGAGAGTACGTAGTGACAACAATGACGAAAGCAGTTCTGCATCACACTGGCAAAGTGCTATGGACTCCTCCAGCCATCTTCAAGTCCTCGTGTGAGATCGACGTGCGCTACTTCCCCTTTGACCAGCAAACTTGCTTCTTGAAGTTCGGGTCTTGGAGCTATGATGGTGATCAGATTGACTTGAAGCACATCAATCAGAAGAAAGGTGACATGGTGGACGTCGGCATCGACCTTCGGGAGTACTATCCTTCAGTGGAGTGGGACATCCTTGGAGTGCCAGCTGAGAGGCATGAGAGGTACTACCCTTGCTGTCAGGAACCTTATCCTGATATCTTCTTCAACATCACGCTAAGAAGAAAAACCTTATTCTACACCGTCAACCTGATCGTTCCATGCGTCGGCATATCATACCTCTCGGTCCTTGTCTTCTATCTACCTGCTGACTCTGGAGAGAAAATTGCACTTAGCATTTCTATATTATTGTCCCAAACTATGTTTTTCTTGCTAATTTCCGAAATTATACCTTCGACATCCCTTGCATTACCATTGCTGGGAAAGTACCTACTGTTCACTATGTTGTTAGTGGGATTGTCAGTAGTGATAACCATTATAATACTGAACGTGCACTATCGGAAACCAAGTACGCATAAGATGGCTCCATGGGTTCGAAAATTCTTTATAACAAAACTTCCGAAGTTACTACTGATGAGGGTACCAAAGGACTTGCTTAGAGACTTGGCAGCGCAGAAGATAGCTGGGAGAAGTATGAAGAACAAGAATAAGTTTAAAGATGCTCTCGCAGCGGCTGAGCAGACGAATTCAAATGCCTCTAGCCCTGATTCCCTGCGTCATCATCTGCCTGGTGGATGTAATGGACTACATTCTACTACCGCTACCAACAGGTTTAGCGGTCTCGTGGGTGCTCTTGGCAGCCTCGGTGCTGGTTACAATGGCCTACCATCAGTCATGTCGGGTCTGGATGACTCTTTAAGCGATGTAGCTCCTCGGAAGAAGTACCCATTCGAACTGGAGAAGGCAATCCATAACGTTATGTTTATACAACACCACATGCAGAGGCAGGATGAGTTTAATGCTGAAGATCAAGACTGGGGCTTCGTGGCTATGGTGTTGGACCGTCTGTTTCTATGGATCTTCACTATCGCTTCCATAGTTGGCACCTTTGCTATCCTCTGTGAAGCACCATCTCTGTACGATGACACCAAACCTATTGACATGATGCTCTCATCCGTAGCCCAGCAGCAATTCTTACCTGTGGACAGTGGGGACTCATAGGTATATTCATAAAAGAAAGTTGATGTCCATGTGACGTCACTGATTTAGACGCGTGAGATTATACAAACTCAAGGAGTGTTGAGATGGTTATAATCAATGGTTGTTTTGACAGAATTTTTCTTAAACATTGTTGATAGTTTAAGTAGTTTTTATTGATACAGCAGTACTATGTCTGCCTTCTTGTTCATTAAAAGTTTTAATATACCGAAAACGTTCATTTGAACAATCTAGATTTCTAGAATCATTATGATCTTGTTGTATTCTTTAGAACTAAGCTTGTTTTCAAAATCTAATATTTAAAATTATTAAGGTCTGAAACTTCAAAGCTCCTGAAATTGCCAAGATAGGTCGGTCGACAATCTATCAAACAAATTAGTACTAAAATAAGACTTTTCCTCTTAAACATTAATTATCTTAATTAGACGTTGTAAAGCCAAAATAGGCTGTGAATTGTATCGTAACATAATGTAAGTAAGTTAAGGTTTCAAGTAAAAAGTTAGTGTTTAAGTTGGATGAAGTAGGAGGGTAATAAATTGTTCTTTCCCTCAGTTTGATACGGTACACTAATAAATTATTGTCTTATCTTATTTTTCTCGCTTTTAGATAACATTTTAAAAGAGTAAGGGACGGTAAATGAAGATGTTGAATCGATAGACGTTGGCGATTCTGAATTATTCATAAGAAAAGTTATATTGGTTTTCGATTTTTCCCAGAATTTTTTTCGGTCTATTAGTGGGGAAGAATCTTGTATTTACTCACACAATTGTATCTTCTGCGTAATCTTGGATTAAGCTAATCGAATTTAGACGAGAAACTTTTTCACTACTTGTAAGGTAAATCTCATACATGATAGAAGAAAATATGTTGATGGATATGAAACAATTGTATTAACTTTCGAATGCAAAAGTATTCCTATTGAATATTAAGGTCCCAAAACTGAACCTTGTGGGACACCACACCAAAATGTTTATAGGACGACTTTTGTAAAGCGCTTGGTTGCCAAAGTGGGTTATAAAGGTGGGGTAACACCATACGTTTTGGACGTAACATTTTCTTTGACAAAACATGGCTGTGTTAGTAATTAATAAAATATTGTAGTAATGAGGAATCATAATGCTCACCATTTATAGGATTAGATATTAATGACGTATATACACATTACGATTTTTTATGTATTATTATTATCGTGTTATTCAATTTTTAGTACAATTATTCATGTTATAGAATTTAAATTAAATCGAAAGCCATGATTCTGTGTAGCTCAGTCGTAGACAAATATATCAATTTTACTTTTTTTATCTTGTAATAGCAATAGAATATTAACTATTAATCTATCATATCTTTGAATGCCTGCTTAAACAACATAATTAATATTTTATGGCAACATATAAGATAATAGTCATACGATAGTATATGCCAAAATAGACTTTTAACTTTGTTATTAAAATATTAAACATTTCCTTTATTGTCAGATTATAAATTCAAACCTCATTTTGAAACAACGTTATAGTTTCTAATAACGTAAAACAAAACTATGTCATAGAGCTTTAAAGAATTTGAAATATAAACAGCAATTTTGCGACGATAGATGGTAATAAAGATAAGTTTATTAAATTGTGCTTTATCAAACTGTTAGAGTCCGCAGAAACCATTAACCATTTTGCCAAAATAAACAGAGTAGTAAAATTAGTTATATACATAAGTAAACTCATACATATAAGTTTAGTTTAACCGACTTTGTAGCAGTTAGTGTTATCCAAACCATGTTTATAAAGTAAACTTTGACTTAATAAATT

>gene8875

TTGTAAACATGTTCCTTTAAAAATATACGAAAAATAGTATTTTTACAATAGAAACATGAAGTTCGGATTTGTGTTCATATTCTTAATTTATTTTAGATATAGTGCTGGAGTAAAACTTCTTGAAGCGAATCCAGATGTAAAGCGGTTATATGACGACCTCCTAAGTAATTACAATCGTCTGATACGACCAGTGACCAATGTCAGCGACATCTTGACTGTAAGACTTGGTTTAAAACTGTCACAACTGATGGAAGTCAACCTCAAGAACCAGGTCATGACAACCAACCTTTGGGTGGAACAAAAATGGTACGACTACAAGCTGCAATGGAACCCTGAAGACTATGGAGGAGTAGAGATGTTGTATGTACCTTCAGAACACATCTGGTTGCCAGATATCGTGCTTTACAACAATTGGGATGGAAATTATGAAGTGACATTGATGACGAAGGCCACGTTAAAATACACTGGAGAGGTGAACTGGAAGCCACCAGCCATCTACAAGTCCTCATGTGAGATCAACGTGGAGTACTTCCCCTTTGATGAGCAGACCTGCTTCATGAAGTTCGGATCGTGGACTTATAATGGAGCTCAGGTGGACCTAAAGCATATGGACCAGTCTCCAGGCAGTAGTTTGGTGCATGTTGGTATTGATTTGAGCGAGTTCTACCTCTCAGTGGAATGGGACATTTTGGAAGTACCAGCTACGAGGAATGAAGAATACTATCCTTGCTGTCCAGAGCCGTTTTCAGATATAACATTTAAGCTGACAATGCGACGAAAGACATTGTTTTACACAGTGAACCTAATCATACCCTGCGTTGGTCTTACGTTCCTGACTGTACTCGTCTTCTATCTACCTTCAGACTCTGGTGAAAAGATATCGCTCTGCATCTCCATCCTGGTGTCCCTCACTGTGTTCTTCCTTGGTCTGGCTGAGATCATTCCGCCAACATCCCTGGCAATCCCCTTGCTTGGGAAATATCTACTATTCACGATGATACTCGTCTCGCTCAGCGTTTGGGTGACTGTTTGCATACTTAACGTGCATTTCAGGTCTCCATCAACGCATACGATGTCTCCGTGGATGAAGAAGCTGTTCTTGCAGTTGATGCCGAAACTCCTCATGATGAGGAGGACTAAGTACTCTTTACCGGATTATGACGATACCTTCGTGTCTAACGGCTATACCAATGAGTTGGAGATGAGCAGGGACAGCTTAACGGATGCCTTTGGTGATTCCAAAGATAATGGTGATTATCGTAAATCACCAGCTCCAGAAGATGATATGCTGTCTGCTGGAGGTGCTCATCAAAGACCTTCAGTGACTGAATCAGAGAACATGTTGCCGCGTCACCTCTCGCCTGAAGTGGCTGCTGCATTGAAGAGTGTCAGGTTTATAGCGCAACACATCAAGGATGCTGATAAAGATAATGAGGTGGTAGAAGACTGGAAATTCATGTCGATGGTACTAGATCGCTTCTTCCTATGGCTGTTCACCATCGCTTGCTTCGTCGGCACCTTCGGCATCATCTTCCAATCGCCTTCACTCTACGACACCAGGGTACCAGTAGACCAGCAGATCTCGTCCATACCGATGAAAAAGAACAATTTCTTCTACCCTAAAGATATTGAAACTATTGGTATTATCAGTTAAATGATAAACTATCATAAATTAGTTTAAAAACAACCGATTTCAAGCTTTTTCTTTTGATTGAAGGCGGTTAGTATACTAACATGTGCTGTTAAGAACAAACGCTTCGACATGAATGGGCCAGCTCGACCGGAGTGATACCACGGCCTCACAGAAGA

>gene9375

GCGCCCGGGCCACCCGCCCCTGTACCCGAGTTTTACGTAACGATGCCCGCGCCGAGCTGATGGCCGCGATCGTGTGGTGGCTGGCGGCCACCTGCCTGCTCCGCGCCGCCACCGCCGGCAACCCCGACGCCAAGCGACTCTACGATGACCTCCTCTCCAACTACAACAAGCTCGTGCGACCCGTCGTCAACACCACCGACGTGCTGCGAGTCTGCATCAAACTCAAACTCAGTCAACTCATCGACGTCAATCTAAAGAATCAGATAATGACGACCAATTTGTGGGTGGAGCAGTCGTGGTATGACTACAAGTTGCGCTGGGAGCCGCGCGAGTACGGCGGCGTGCACATGCTGCACGTACCCTCCGACCACATCTGGAGACCAGACATCGTCCTCTACAACAATGCCGACGGCAACTTCGAGGTAACGTTGGCGACCAAGGCGACCATCTATCATCAGGGGCTGGTGGAATGGAAACCACCCGCCATTTACAAGTCTTCCTGCGAAATAGACGTGGAATATTTTCCTTTCGACGAGCAAACATGCGTTCTTAAGTTCGGTAGCTGGACGTACGACGGATTCAAGGTGGATCTGAGACACATGGACGAGCAGGCCGGCAGTAACGTGGTGTCGGTCGGGGTCGACCTGTCGGAGTTCTACATGTCTGTCGAGTGGGATATACTCGAGGTGCCAGCTGTCAGGAATGAGAAATTCTACACTTGCTGCGACGAGCCTTACTTGGACATAACATTCAACATCACGATGCGAAGAAAAACGCTGTTTTACACAGTGAACATCATCATCCCTTGTATGGGGATCTCTTTCCTTACCGTCCTCACATTTTACCTGCCTTCAGACAGTGGAGAGAAGGTAACGCTGTCGATATCAATATTGATTAGTCTCCACGTGTTTTTCCTGCTGGTTGTCGAGATCATACCTCCTACGTCACTGGTCGTACCGCTATTGGGAAAGTATCTTATATTCGCCATGATTCTCGTTTCAATAAGTATATGTGTGACGGTAGTGGTCCTAAACGTCCACTTCCGTTCGCCGCAGACACACCGGATGGCGCCTTGGGTGAAACGAGTTTTCATACATATTCTACCACGACTGCTGTTTATGAAACGGCCGCAGTACAAGTTTGACACAACGAGTATCGGGTACCACAGATCGCGCTACACGGCGTGTGGTGTGGTGGTGCGGTGTGGAGGGGCAGCTCGGCCTCTCTACCCGTACCGGCTGGCAGCGGCTGACGACGACTGCTGCGCACCCGGTGCATGGCCGCCGCCTGCTGCACCACCACCCAGACCTCTCACGAGAACACCAAGCAAAGAAGATCTCACACACACCACCTACCTTAACTCAGGGATGGGTGATAGCAACAGGTTTGGTGGTAGCTGTCTGGTGCATGGCTCGAGTGATGGCGGTGCGGGACTAGGTAGCGGGTTAGCAGGCGGCTTGGCCGGTGGGCTGGCCAACGCTGAAGACGAGACCCTCAGTCCCGTCCCCGACCCGCCGCCTGGCTTCAGTCACTCCGCCTGCCCACCAGAAGTACACAAAACATGCTTCTGTGTCCGATTTATTGCAGAGCACACTAGAATGCTCGAGGACTCTACTAAGGTTAAAGAAGATTGGAAATACGTAGCGATGGTGCTGGACCGTTTGTTCTTGTGGATCTTTACGCTGGCTGTGCTAGTGGGCACCGCCGGTATCATCCTGCAGGCGCCAACGTTGTACGACGACCGAGTGCCCATTGACAAGCACTTCAACCAATACGCGACCTCGTCGCTCGTGCGGTGTCCACCACCCTCGTAACTTGATAATATTGACTACATTTATGTTTACACAAGTATGACGTCACAGTACCAACTTTTATTTACAGACAAATACTGTTGCTACAACATTTACTACTGAAAACGTTTCATACTGCAATGTTTGCTGTTCAAAATTGTAATCTGAGACTGGGACACATTCATTTTTATTAAAACTCTGACGCCATACTCGTGTAGCCATGTCTATTTGTATGGTTATTCAATAATTATACAAACAACGGTGTCTTCAAAGGTGCAGTGATTTGTTTGAGAAAATCTGTTTAGGTTATAATCTAAAAAAACTTAAAACCTTGTACTGGCCTTATGACTCAAGGTCTACTTATTTGAACGTTTGTAACGATTTTCATGAACAAGTTGCAGTAACACCGTATGACATTATTCTTATTGCGTCGCCGTTGTATTTCAAGACCCAACGCACACCCGTCCCACCCGGAACGTGTTCTATTCCACGACACGTAAATATATCTATCTAATAAAGTTTTCGTAATAATAATCATAAGAACTTTCATATCTTTTCATATTATCTATTCTTGCACTCATTGTTAGTATAAACATTGATTCACTCATTCTGAGCTCCCACGCAAGAGTTTGAGCATTGTTTGGCTCATACAACGTCTTATGTGACAATAAATTGTAGCAATTGTTTCCTGTAGTTATCTTATTGCAGCGCAGTATCAAAATCCAAAGCGACTTACGATTCACAAAATCATCTAAGAATACACTAGTCTCTATGAATAGATATAAGATAATGAACGAAACACCACCATGTTAAATGTATCTGTGTTCAAAAACGTCATTTAGTAAATTAGCATACATAATATCGTAGCTAGCACCTTTAACTAAGCTCATCCAAAGCGATAGACCAACAAGTCTGCTTATTACACCTCGCTTATTCATATAGGAATTTAATAACTGATTTTCATACAAATTGTCAACATTAGCTCTAATGTAGACATCGAAATCGAATGTAATTTTAAGGAATTCATAGGCACTAGACGTAGTGTAAGTCTGTCTTAAAATCATGCTGAAACTATCGACTAGAGATTCTTCCAACTCTTCTATTTCTAAGAACATTACTATAATTTGTCGTAGAATAGAACATGTACTTTAAAATAAGAATATCGATTTTATTATTTATAAGAAAAAAATAGTATTTGAAACCTATAAAATACAGTAACGTATTTATAAAACGATTTATTGTTGGCTTTGAAAGAACGAAATTCAGTTAATAGACTCGATGAATATCGTAAAGATTTACAAGACCAGAGTAATGAAAAATATCCTTATGATAATAAGTACAACATAATGATTTACTTTAAGAAGATAATTGCTCAATTTTAAAGTCCGTACCTTCTTTAGTTTTGCTACTGTTTATTAATACGCCGAGTGATTATGAACTGACCGTAACCTAATATGAACTAAGTAGGTACTTATTGATTGATTTATGTTATACTCCTACATTCGCTTAATCAAAATGGTGTCACTATATTACGTTGGTATATAATTTACATAATATTCCAAAAAATGTACTATGTTACATATTATAGTGTAGAATCACTGTAAATAAAATTAATGTAATTATATTTGAGGCACGCGTTTGAGCAATGGCCGTGCCAAACTTTTGGACAGGAATTGAGATTATTTATTAATTGAAAATGAACAATACACAATAAATAAATTACCAAAAATTAAAAAAGAGTGTACAAAAATAAATAACGAATATAAAACATTAAGAGTAAAAGCTTATTGTACTGAGATGAAAAAAAATCATGACAAACGTGATTATGATAGTGGTCATAGTCATGTCATATTATAATTAAATATCGTAAATTGTAAATTCACTATATGTATAATAAAATGCGAGGCTATAGTCGAATATTAAAATGCTGCATAGTCATAGACTATTACATTATATCACAAGACTATTAAAATACTAAACATCAATTTAATTGTTCCACGTAAACGCAGTCAAGCGTTCTACGTCTTATCACCAACAATCATATTTTATTGGCGTTTAAATATTCGGCTATATGTACAATATTATTGTCAATAAACGAATAGAAAGTACTGGTGCATAGTATAGTACCCCATAAAAGTAATCTAATCTAATAAATAAGATATATACCTAATACACATTGACTGCTCGCAGTAGAGTAGCGCAGCTCAATTGTAGAAATATAAGTTTGTAAGTACTTATTTATATTGTTAATAAAATCGTTATATATAGTCTTCTCTTTTCTTTCCGTCGACAATTTATTACTAAACACATGTTTATTGTGATAGGGGCCTATAAGGACGCTCTTAAAGTAATACTAACTTATGTTATTGCGCGCTATGACGCGTACAACAGCTGCCGATGAGGTCATAC

>gene9676

CGCCGCGACCGCTCGCCGCTGATGCCGCGCGCCGCCTGACGCTCCCTCTGCCGCGACACAGCATGCCCGCCGGAGGAGCGTGAGCCTACACCGAGTGATCACTTGCGAACACTGTGTCCTTCTAATGATGAATTAACGTGCACACTTTATCAACTAAACTCTCGTTAAGGATTTGCTTGGTCCCGAATTGAGTCCGGGCAGGCGGCGCGCGGTGGCGGCGCGATGCGCTGACGCCATGCGCGGGCGGAGATGCCGCGCCGTGCCGCGCCGCACCCCGCGGCCCCGCTGCTGCTGCTGGCCGCGCTGGCCGCGCTCGCCGGCTGCGCCGCCAACCCCGACGCCAAGCGGCTCTACGACGACCTGCTCAGCAACTACAACAAACTCGTGCGGCCCGTGCTCAACGTCAGCGACGCTCTCACCGTGCGCATCAAGCTCAAGCTGAGCCAGCTCATCGACGTGAACCTGAAGAATCAGATCATGACGACTAACTTGTGGGTGGAGCAGAGCTGGTACGACTACAAGCTGTCGTGGGAGCCGCGGGAATACGGCGGAGTCGAGATGCTGCATGTACCCTCAGACCACATCTGGCGACCCGATATAGTTCTCTATAACAATGCGGATGGCAATTTCGAGGTGACGCTGGCGACAAAGGCGACCCTCAACTACACGGGTCGCGTGGAGTGGCGTCCGCCTGCAATCTACAAGTCCTCCTGTGAGATCGACGTCGAATATTTCCCCTTCGACCAACAGACTTGTGTCATGAAGTTCGGCTCATGGACGTACGACGGCTTCCAGGTGGACCTTCGGCATATCGACGAAGCGCGTGGCACGAATGTCGTGGAGCTGGGTGTCGACCTGTCCGAGTTCTACACTTCCGTGGAGTGGGACATTCTCGAAGTACCAGCCGTCAGAAATGAAAAATTCTACACGTGTTGCGACGAGCCGTACCTGGACATCACGTTCAATATCACGATGCGCCGCAAGACTCTCTTCTACACGGTGAACCTCATAATCCCCTGTATGGGCATCTCCTTCCTCACCGTGCTGGTCTTCTACCTTCCTTCCGACAGCGGCGAGAAGGTGTCTCTCTCGATCTCCATCCTTCTATCGCTCACCGTGTTCTTCCTGCTGCTGGCTGAGATCATCCCGCCAACGTCTCTCGTCGTACCTCTGCTCGGCAAGTTCGTGCTGTTCACCATGATCCTCGACACGTTCAGTATATGCGTGACGGTGGTAGTGTTGAACGTGCACTTCCGGTCACCGCAAACCCATACGATGGCGCCGTGGGTCCGGCGTGTGTTCATCCACGTCCTGCCGCGCCTACTCGTGATGCGCCGCCCGCACTACCGCGTCGATCCGCACCGCAGTCGCTTTGCAGGGCTGGTGACTGCGGTGAGCGAGAGCACGCCGTGGGACGAGAGCTCGCCGCTGGGCGGCGTGGGCGCCGTGCCGGGCGTGCCGGCGTGTGCCACGTGCCGCTCGTGCCATCTGCACGACGCGCCCGCGCTGTGTGATGCACTGCGGCGCTGGCATCGCTGTCCCGAGTTAAACAAGGCCATCGACGGCATCAACTACATTGCCGAACAGACGCGGAAGGAGGAGGAGTCTACTAGGGTGAAAGAGGATTGGAAATATGTGGCGATGGTGCTGGATCGACTGTTCCTGTGGATCTTTACGCTGGCCGTGGTGGTGGGGTCGGCCGGCATCATCCTCCAAGCGCCGACTCTGTACGATGAGCGCGCGCCTATAGACGTGCGTCTGTCGGAGATTGCGTATGCGACTGCGAAGCCCCGGCCTCCGCCGCCACGCTAGGGGTGGCGTCCTGAGCTAGTCTTTTTATAATTTATTTGTTACGGTGATCCAAAGATGTCATAAATGTCCGTTTGTATTTTCCGGCGTCTTGGATCCCCACAAAACGTAATTTTAATGATATTGTAAATAATATACATACATATTATAGTTTATACGACGAAACGACACGATCGAAAAAATGTCGAATTTCTTTTGATA

>gene1175

CAGCAGCCGCACGCGACCGCGAGTGCATCAGTGCCCGTACACTACAAACACTGACACTCCGACCTTCAATCCGTGGGAACGAGACATCTATATCAGGTGGTGACGAATAGGATGTGAAGTGGTGTTCCCTGCTGCACACGCGCCGCTAGCAGCGGGCATGGCGACCCGGAGATAGTGCCAGGTGGTGTCAGTGGGAGCGGGAGGAGCGGCGGCATGCGCGTGGTGTTGGCAGCGCTGACGGCGCTGGCGGCGCGCGCGCTCGCCGGGCCTCACGAGCACCGCGCGCGCCACCACGCGCCCGAGCACCCTCCACACTTCCCCGCGCCGGCGCCGCCACAGCCCTACCGCGGACACGGCGAGGCCGTCCGATACAACCCCGAACTAGACACTATACTACCCCGACTTGAAGATCACGAGACTTCCTCCAAACGAGCAAAGTTTGAAGATGCAGAAACCTCTTCTAAGAGAGCCAAATACGATGAAAGATTCTACTCGAATCATGAACGAGCTGATGAAGAGCCTATGGCTGATGAGCCACAGCTCGGGCCAGAGGACGACGATCCACTCGTCGTTCGTACGAGAAAGGGAAGAGTAAGAGGCATCACATTAACTGCTGCAACTGGGAAAAAAGTCGACGCATGGTTCGGCATCCCATACGCTCAAAAGCCTGTAGGTGACTTGAGATTCAGGCACCCTAGACCGGCAGAGAGCTGGGGAGATGAAATATTGAATACTACGACACTGCCACATTCATGCGTTCAAATTATAGATACTGTGTTTGGTGATTTTCCAGGAGCGATGATGTGGAATCCCAACACAGACATGCAGGAAGACTGTCTCTTTATTAATATAGTCACACCCAGGCCACGACCCAAGAACGCTGCTGTCATGCTGTGGGTCTTCGGCGGTGGGTTTTACTCAGGAACTGCTACTCTAGATGTTTACGACGCAAAAATACTCGTATCAGAAGAGAAAGTAGTCTATGTTTCAATGCAATATAGAGTTGCGTCCCTAGGTTTTCTATTCTTTGATACTCCTGATGTACCGGGTAATGCTGGTCTTTTTGATCAGCTCATGGCTTTACAATGGGTGAAAGACAACATAGCTTACTTCGGAGGAAACCCGCACAATATAACATTATTTGGTGAGTCGGCGGGGGCTGTGTCGGTTTCATTGCATTTGTTGTCGCCTTTATCGAGAAATTTGTTCTCTCAGGCGATAATGCAGTCAGGAGCAGCTACTGCACCGTGGGCCATTATATCAAGAGAAGAAAGCATTTTGAGAGGAATCCGATTAGCAGAAGCTGTCCACTGTCCACATTCCAGAACGGATATGGGCCCTATGATTGAGTGTCTCAGAAAGAAGAGTCCAGATGAACTAGTCAATAATGAATGGGGAACTCTTGGTATTTGTGAATTTCCCTTTGTGCCCATCATAGATGGTTCTTTCTTAGATGAGTTACCTGCTCGATCATTAGCTCATCAGAACTTTAAGAAGACCAACCTTTTGATGGGATCCAATACGGAGGAAGGTTACTACTTTATACTTTATTATCTTACTGAACTATTCCCCAAGGAAGAGAATGTAGGGATAAGTAGGGAGCAGTACTTGCAAGCAGTCAGAGAGTTGAATCCGTACGTGAATGACGTTGGTAGACAGGCTATTGTGTTCGAGTACACGGACTGGTTGAACCCAGACGATCCAATAAGGAACCGAAATGCTTTGGATAAGATGGTGGGAGATTATCACTTTACTTGTGGAGTGAATGAAATGGCGCATCGTTACGCAGAAACTGGAAACAACGTCTTTACATATTACTACAAGCATCGCAGCAAGAACAACCCCTGGCCATCATGGACAGGAGTGATGCATGCTGATGAAATCAATTATGTATTCGGGGAGCCATTGAATCCAGGAAAGAACTATTCGCCGGAGGAGGTGGAATTCAGCAAGCGCCTAATGAGATATTGGGCTAACTTCGCGAGAACCGGCAACCCATCGATAAACCCGAATGGAGAATCGACGAAGATCTACTGGCCAGTCCACTCGGCTACCGGCAGAGAGTACTTGTCGCTGGCAGTCAACTCCAGTACAGTAGGCCACGGGCTGAGAGTCAAGGAGTGTGCGTTCTGGCAGAAGTATCTGCCACAATTGATGTCTGCTACCAATAAGCCAGAACCTCCAAAGAACTGTACGAGCAGTGCGGCACCAATCAAAGTGCCGTATGAAATCATTGGAGTGGGTGTAGTCATCGCCACTGGTTTGGCCAAGACCACTATGTTCAAGTATATCGTGTAACTGTATCCTGTGGTCTTCGAGTATCTTAGATTTATTTGCTTCGTCTACTGTTTCTTCATTCATAAGATTCAGATTGTTAGAAATATCGTCAAAGTTTATGAAAGTACCATACGTTAGGGACATTATTTTTGAATGAATTATTATTATATTTTTCAATCATGGGAGGCGAATAAAGGTGTTTAGAAATGAAATTGTAATTGATTTGAAGTTGGTCAAAATGTTCATGTAATTTCGATTATACCTACATAATCATTTGATTATACATAACTAATCTAACACATAACACACCAACAACCGAAACCTACATAAT

>gene9347

TCGATGGGGACGCATCGCGGTCGGTCTCTCCCGAAATATTATTACTTTATTACCATTCAAAACGCGAGTGAGTGTCTGTGAACTTCTATCAACTTTAAGTGTCAGATTAATAAGACGCTTCCCTTCCACGACCGGTCCACTCAAGGTCATCGATCAGCAATTTGACACCTGTTGTGTTTATATACTATATGTATGTAATTTCATATGTAATATAAAATAAACAGTAATATTGTGTAACATTGTACACCCGCAGCAGCATATCGAGTGCCACTAGAAATGTGTTTCAATAAGTATCTTATCAAGCTGGCGGACTACTTCAGATTGTACTCTGACACGTCTCGTTAAGGCTCGTGTGAATAAAAATTATTCGAGATGATCAGCAACACGAAGATTGTGTTCACCAAGCTCCTCCTCTGCTGCATCGTGTCAGGAGCCTGGACGCGGTCATGGGCAAACCACCACGATACCACTACCTCAACCACACAAACAACCCCCACCACGAGTCAAGCACCAAAGAATTTCCACAATGATCCACTCATCGTCGAGACGAAAAGTGGGCTCGTCAAAGGTTACGCTAAGACAGTAATGGGGAGAGAGGTACACATCTTCACCGGTATTCCGTTCGCGAAGCCTCCGCTAGGACCGTTAAGATTCCGCAAACCGGTACCCATCGACCCGTGGCACGGAGTACTTGAAGCCACAGCGATGCCAAATAGCTGTTATCAAGAACGGTACGAATATTTTCCCGGTTTTGAGGGAGAAGAAATGTGGAATCCAAATACAAACATATCAGAAGACTGTCTCTATCTAAACATTTGGGTTCCGCAACATTTACGAGTTCGTCATCATCAAGATAAACCTCTTACGGAGAGGCCCAAAGTGCCAATACTAGTGTGGATTTACGGTGGCGGCTACATGAGTGGCACGGCAACACTCGACCTATATAAAGCAGACATAATGGCTTCTTCCAGTGATGTAATAGTAGCATCTATGCAGTATAGGGTGGGCGCGTTCGGATTTTTATACTTGAATAAATATTTTTCGCCGGGCAGTGAAGAGGCTCCGGGAAATATGGGCTTATGGGATCAACAACTTGCGATTCGTTGGATTAAAGATAATGCGCGCGCTTTTGGAGGTGACCCCGAATTGATAACATTATTTGGTGAGTCGGCTGGAGGGGGGAGCGTGAGTTTGCATATGCTTTCCCCAGAAATGAAAGGATTATTCAAAAGAGGAATATTGCAATCTGGGACGCTCAACGCTCCGTGGAGTTGGATGACAGGAGAGAGAGCACAAGACATAGGAAAAGTTTTAGTAGACGATTGTAATTGCAACAGTAGTCTTCTTGCCGCTGACCCAAGTTTAGTAATGGACTGCATGCGTGGAGTGGATGCAAAAACTATATCAGTACAGCAGTGGAATTCGTACACTGGCATTTTGGGATTCCCGTCAGCGCCGACAGTGGACGGCGTGTTTTTGCCCAAAGATCCGGACACAATGATGAAAGAAGGCCATTTCCATAATACCGAAGTGCTTCTTGGAAGTAATCAAGACGAAGGAACCTATTTCTTGCTCTACGATTTCCTCGACTATTTTGAAAAAGACGGACCGAGTTTTCTGCAGCGGGAGAAATTTCTTGAAATCGTCGACACTATCTTCAAGGACTTTTCCAAAATTAAGAGGGAGGCTATCGTTTTTCAATATACGGACTGGGAGGAAATTACCGATGGTTATCTGAACCAAAAAATGATTGCAGACGTGGTGGGAGACTACTTCTTCGTGTGCCCCACTAATTACTTTGCGGAAGTGTTGGCGGATTCGGGCGTCGAAGTTTACTACTATTACTTTACCCACCGTACCAGCACAAGTCTGTGGGGCGAATGGATGGGCGTGATGCATGGCGATGAGATGGAGTACGTCTTCGGGCATCCATTGAACATGTCTCTCCAGTATCACACCAGGGAGAGAGACCTGGCTGCTCACATCATGCAGTCATTCACCAGATTCGCACTGACTGGTAAGCCTCATAAGCCTGATGAGAAATGGCCGCTGTACTCTCGTTCGTCGCCTCACTACTACACATACACTGCGGATGGCACTAGCGGACCTGCTGGACCTCGAGGACCTCGAGCCTCCGCCTGCGCTTTTTGGAATGACTTCCTCAACAAGCTCAATGAATTGGAGCACATGCCGTGTGATGGAGCTGTGACAGGGCCATACAGCAGCGTAGCTGGCACCACACTACCCATAGTGCTGCTGACAACGCTTGCCACAACCGTCGCGTTGTAAACGCAGCATATCACCGCTTGAATGATTCAAGCGAATATTTAAGCATTAGGAAAAGAGTAGAAAAAATATTTATACAAACTAGAACAATAACAATGCAAATTAATGTTTTCCTTCACAAATAATTATAAAATATGAGTTTCATTGCGATAATGTCATAAAATCATGTATCAAATTGTCTCAAGAAAATTTATATTTTATACTTATTTGTGAAGATCTAGGCGAGTCAGCAAAAGGCGTTTCGAATTTTAATATTTGCTACTAAAGTACTAGAGGTAATTGTTGTATTAATTAAATATATTTTATCGAAATCTTACATATCACTGGCGTGTGGAACGTGAAACGAAAGACAAGGACAATGGATCGAAAATTATGAAAAAGGAACTTTAAAAAGTGAAATCGTGATGTGAATTTGTACGAGATTATGAAGAAGGACGTTATTTAGAAGATGTTATATTTCTTTTCTTGTATTATAATAATATTGTAGAAAAAGTATGTTGAATGTTAAATAATTTAGTTACAATGTTCCTGTAACTAGTTGTTTCGTATGTTTGATGTTTCATGTATTTAGTTAATGTGTATTCCATGTTATGAGTATGAATATTATTATATAAATGTCGGCGGATTATTATAATACCTATGCGTATTGAATAATTTATTATTATACTTAGAGAAAGTACTTGTACGTCGGCTTTCATTGTTAAAGCGAAGCTTTCACACACGACAGTTTGATAAATCACTATACATATCAAAGTGTGCCTCTGCTTAAATAGCAAAATGACAACTTATTCGTAGCAAAATTTTACGATTTTATATATTTAATGGTAATATAGAAGTCGAGTGTATACTGTGAGATGAATTTTTATCAATATATTTACTACTATTGTATTTAGGACGCTAATATTCGTACCACTTGAGGATCTTATGAACTCCAAATTCTTATGACTTCGAACAGATAATAGTATATTTTGTGAATATTCCCTTCTTTAGTATAGATGCGATATTCTGAACGCTGAGTAGACTTATGTGTACCTTACTTATTGTATTTGATAGGCAAAGCACAAGATACCGTAAATCAATTTAGTACAATTAAGTCACATGATTCTGACACTTGTTTCTTAACACGGTTGAGTACATTTTCTCTTGACACTATCGAAATCGTATACATATCATTAATGTAAACATACATATCAAATATATTTATGAAGAAATTATATACAGGTGTCTATTCGTCTTCACAATAGCAGTGAATTCCGAAACAGTACGTAATTCGTAGCTTAATAACGCTTTAAGTGAAAAAATATCTTATGAACTTAGTAACAATTTTTATACTTTTAACATTATATTTGTACTAAAAAACGTTAGCGGTAATCAAATATTTATACCTATGCGAACGTCAATTACTATCCTTGAACAAATTGGAAGGCGGCGGGAGTATTAAAAGAAAGTGGCCATGCTTACTCAAACCATCAAGAAAAATATTGATTTAGTACTAAATTGTATCTATTTTCACCTTTCCTCATATTTAAAACTGTCCTTGAAACACGGTATTCCCCCTTTTGTGAGCACGAAGTAGTTTTATAATTTAACAACGGACTTTTTATACAAAATTTAACTGTATTCCCTAAAGAATATAGAGTTTATTGTAACATTGGCAAAGATGCACAACACTCGCTTTCTTGTTGAATTAAGCACAATTTGAGAAGTTGTATGGGATAGCTTTAATTAGTAAGAATAAGATGTAAAGTGATCTAAATAAGAAATGTATTATAAGTAGCAATTTCAAATAATGTTAGGATCAGTTTCAACCAAAATGTTAATTTAAATCATGTTGTAATAATGCTTCAACAAATTAATGTAAAACTAAATACAGACATTATATATTTTGTAGTTATATTATTAAATAGTTGATACATTACGTTCACGTTTATTAAAATAAAAGGAATATTGCACTTAATTTTAATACAGTTATCTTTTCGATATTATTCGAAATCTATTTATTATAAGGAATTCTTGTTACTTACTTGTGTCAAACTTATAGTACGTGCAATCAGCCTTGACTATTTTATATAACCCTTTTGCGAACGTTAATTCCACTGTTATATACTTTTGACTACCTATCTGATAGTTTGGTTACAATAAAGAGTGTAATTTTAATTAAAACAAAACCAGCTTGGTGATTTGGTACGCAAGTAACAAAATAAAGATTTATTCAGCTCTCGTTATAGTAAGTCGTATGTACACGAAGCTAAGCGTTGTACAACCACGATTGCTTGACTCAGTAAGTACCGATGATTACTGTGTGGATGTTCTGCAATGAATCAATCTGTTTTTTTACCTGCAAATCAAATTATTTGGCTGATTTAAAAAAACTCAACATATATATTATAATGAACAAAATAAATACATAATAATATTGTATTATGAAACCATTTACTTTATTTTTTTAACGGGCCTAAAACTTGATTTCCACACATATAGATCTATGTTATGGTAAAGGTAGGATTAGTCCTGCATACTATCGCTGTACGAGACGATATCAATACGTACCGTTGGTATATATTTGTTATATAACCTACTCATTAGGCGTAGTATATAGTTTAGTACGTAAGTCTTAGTATTGATGTGATTGCCATTAGAATTTTACGTAACTAACTGGACTATCTTATTTTCTCTCTGCAATTGTGACTATGTAGGCAGATTATCCATACTAATTAATCAAAATATATGCGTAAACAATAGTTTTTAACAATGTATGTATGTAAAACAGCTCTTAAATGTGTAGTCAATTAGAGAAAATCTAAAATGGTATTATTTGTTAGATAAGTTAAATGTTAAATGTTATTTGTGTAAGCTTACATCTGTTATGCAAACGATAATAGTAGTTACTGCTTTACAAATTAGGTGCTAATTTAATGTGTGTATATATGAGTGTGCACATTCGTTTTATCTAAAACTAAATTGCAGATGACGTCGACAATATGTGTTTTATATGTTATATCCAGTGTCGTGTGCTATCAATGCTTTCAGTAGATGAAAAAAAAATAGCTGTATGACTTGATTTCCTTCACAGGTTTATATAATATTACGCTAAAATTACTATCAACATTAGTTATTATTGTAATAGATTTTGAATGTCGATATATTTAGTATATTTTTATTATTAAAGTAAATTCGTTTGTACAGTTGCACGAATATTTATTTCACTGTAGCGGAATAAACTATTTTTGTCCAAATACTGTTACACAATAGTATATTGCTTTTAAATAAGTACTATTTGTTTATTATCTCTATCCAAAAAATTAC

>gene11613

CGCTCGCGCAGCCCGGACGTTTGAGAATCGGATTTTTTAAATCCAAATTTCGAAATTCCAATCCCGACTCCGACTGGAAAGGCCACGTGATTGGGTTGATTTTGTAAAAGGAACGTGTGTTAATGAAATTAGCATCTCTAGTGTTGTGAACGGTGAAAAATGTGTGAACAGTGCGTGCAGCGTTGATTCCAGAAAAAAGGATTTATTTGTGCGTTTTTGGCGCATGTGTGATGCATTTCGAGTGTTATGGCAACGTTGCAGTAACGTACTGACAGTTAACTGAGAATTCAAAATGGCGGAAGCGGAGGGAGGAGCAAGCGAGCAAGATGATGTTTCATTCTTGCGTACGGAAGACATGGTCTGCCTGTCATGCACGGCGACTGGCGAGAGAGTGTGTTTGGCGGCCGAGGGGTTCGGTAACCGACACTGTTTCCTCGAGAACATCGCCGATAAGAACATACCGCCAGATCTATCACAGTGTGTGTTTGTCATCGAACAGGCTTTATCAGTAAGAGCGTTGCAAGAGTTGGTGACGGCTGCAGGGTCTGAAACTGGAAAAGAAAACTTAGGTAAAGGCACCGGTTCTGGTCATCGTACCCTGTTGTACGGCAATGCTATCCTACTACGACATCTCAACAGTGACATGTACCTGGCCTGTCTGTCGACTTCTTCATCTCAGGACAAGCTGGCCTTTGACGTGGGTCTGCAAGAGCATTCGCAGGGTGAAGCCTGCTGGTGGACGCTGCACCCTGCCAGCAAACAAAGATCTGAGGGTGAGAAAGTCCGAGTCGGTGATGACTTGATTCTGGTATCTGTGGCTACTGAAAGATACTTGCACACTACAAAAGAAAATGAAGTATCGATAGTGAACGCTTCATTCCACGTGACTCACTGGTCAGTGCAACCATACGGTACTGGCATCTCTCGTATGAAGTACGTGGGTTATGTCTTCGGAGGTGATGTCCTGAGGTTCTTCCACGGAGGAGACGAGTGTCTCACCATACCCAGTACGTGGACTAAAGATGGGGGACAGAATATTGTTGTCTACGAGGGCGGTTCCGTGATGTCACAAGCTCGTTCACTATGGCGCTTAGAACTAGCTCGCACCAAGTGGGCAGGAGGCTTCATCAACTGGTACCACCCAATGCGAATCCGACACATCACGACTGGCAGATACCTCGGAGTCAATGACCAGAATGAGCTGTATTTAGTTAGCAGAGAGGAAGCCACAACAGCGTCCTGCGCGTTCTGCCTCCGCCAAGAGAAGGACGATCAGAAGCAGGTGCTGGAAGACAAGGACCTGGAAGTTATAGGCGCTCCGATCATCAAGTATGGTGACTCCACTGTCATTGTACAGCATTCAGAGACTGGGTTGTGGCTCTCTTATAAGTCGTACGAGACAAAGAAGAAAGGAGTAGGTAAAGTAGAAGAGAAGCAAGCGATCCTTCACGAGGAAGGCAAGATGGACGACGGACTAGACTTCTCAAGGTCACAAGAGGAGGAGTCCAGGACTGCTAGAGTCATTAGGAAGTGTTCGTCTCTGTTCACCAAGTTTATCAATGGTCTTGAAACTCTGCAAGAGAACCGTCGTCATTCGATGTTCTTCGCGTCAGTTAATCTTGGGGAGATGGTGATGTGCCTCGAGGATCTGATCAACTACTTCGCTCAGCCAGATGAGGATATGGAGCACGAAGAAAAACAGAACAAATTCCGCGCGCTCCGAAACCGTCAGGACCTGTTCCAAGAGGAAGGTATCCTCAACCTGATCCTCGAAGCCATCGACAAGATCAACGTCATCACATCGCAGGGTTTCTTGGCTGGGTTCCTGGCTGGAGACGAGTCTGGACAGAGCTGGGAGATGATCTCGGGGTACCTGTATCAGTTGCTTGCGGCCATAATAAAGGGCAACCACACGAACTGCGCGCAGTTCGCCAACTCGAACCGCCTGAACTGGTTGTTCTCCCGGCTCGGCTCGCAGGCGTCGGGCGAGGGGACCGGCATGTTGGACGTACTACACTGCGTGCTCATTGACTCGCCTGAAGCTTTGAATATGATGAGGGATGAACACATAAAAGTAATTATCTCCCTCCTTGAGAAGCACGGTCGTGACCCGAAAGTACTAGATGTCCTATGTTCACTCTGCGTCGGTAACGGGGTGGCGGTCCGTTCGTCACAGAACAATATCTGCGACTATTTACTGCCTGGCAAGAACTTGCTATTGCAGACTGCCTTAGTAGACCATGTGTCCAGTGTTCGTCCAAATATCTTCGTGGGACGTGTAGAAGGGTCGGCAGTGTACCGTAAATGGTACTTTGAAGTGACCATGGACCACATCGAGAAGACCACACATATGATGCCGCATCTTAGAATCGGATGGGCTAATACTACTGGCTACGTTCCCTACCCTGGTGGTGGTGAGAAGTGGGGAGGTAACGGTGTGGGAGATGATCTATACTCGTATGGTTTCGATGGAGCATACCTCTGGTCTGGTGGCAGGAAGACGCAGGTTAACAGGACCCACGCTGAAGAACCTTATATTAGGAAAGGTGATGTCATCGGCTGCGCCTTAGACCTGACAGTGCCTATCATCAACTTCATGTTCAACGGCGTGAGAGTGACAGGCTCCTTCACGAACTTCAACCTGGAGGGCATGTTCTTCCCTGTCATCAGCTGTTCGAGCAAGCTGAGTTGTCGTTTCCTCCTGGGAGGCGAGCACGGTCGGCTCCGTTACGCGGCCCCGGAGGGGTACTCTCCCCTGGTGGAGTCGCTCCTTCCTCAACAGATCCTCAGCCTGGAGCCATGCTTCTACTTCGGGAACTTGTCCAAGCGAGCTCTGGCTGGTCCACCGCTAGTACAGGATGATACAGCTTTCGTGCCAACTCCTGTTGATACGCTGCAGATACCATTACCTTCATACGTGGAACAGATCAGAGACAAGCTAGCTGAAAATATTCATGAAATGTGGGCCATGAACAAGATCGAAGCAGGTTGGATGTACGGAGACCAGCGCGATGACTTGCACAAGATCCACCCCTGCCTGGTCCCCTTCGAGCGCCTGCCTCCCGCGGAGAAGAGATACGACATACAACTTGCTGTGCAAACACTCAAGACAATCCTGGCCCTAGGCTACTACATCAGCTTAGACAAGCCGCCGGCGCGCATACGCAACGTTCGTCTGCCGAACGAACCGTTCATGCAGTCAAACGGCTACAAGCCAGCACCCCTGGACCTGAGTGCTGTCACCCTGACACCCAAGATGGACGAGCTCGTGGACCAACTCGCTGAGAACACTCACAATCTATGGGCCAGGGAGAGGATCCAGCAGGGCTGGACATATGGACTTAATGAGGACCCCGACATGCACCGTTCCCCCCACCTGGTGCCCTACCCGAAGGTAGACGATGCCATAAAGAAGGCCAACAGAGATACTGCCTCGGAGACTGTGAGGACTTTACTAGTCTACGGATACAACCTGGACCCACCCACTGGAGAACAGCATGAAGCCCTCTTGTTAGAAGCATCAAAACAGAAGCAAGCAGAGTTCAGAACGTACAGAGCTGAAAAGAACTATGCTGTCGGCTCCGGAAAGTGGTACTTCGAGTTCGAAATCCTTACAGCTGGGCCGATGAGGGTGGGTTGGGCCCATGCCGATATGGCTCCAGGCATGATGCTTGGCCAAGACGAAAACTCCTGGGCATTCGATGGTTACAATGAGGAAAAAGTGTTCAGCGGTAGCACAGAATCGTTCGGCAAGCAATGGTCTGTGGGAGACGTAGTCGGCGTGTTCCTAGATCTCATTGATAAGACGATAAGTTTCTCTCTGAACGGAGAGCTGTTGATGGACGCTCTCGGTGGAGAGACCACATTCGCAGATGTTCAGGGCGATAACTTCGTACCAGCTTGCACTTTGGGAGTGGGACAGAAAGCCAGATTAACATACGGTCAAGATGTGAACACCCTGAAATATTTCACGACGTGCGGTCTTCAGGAGGGCTATGAGCCATTCTGTGTCAACATGAAACGCGACGTGACACACTGGTACACGAAGGACCAGCCCATCTTCGAGAACACGGACGAGATGATCGACACCAGGATTGATGTTACTAGGATACCTGCTGGATCTGACACGCCACCGTGCTTGAAAATATCTCACAACACATTCGAGACTATGGAAAAAGCGAACTGGGAGTTCTTGCGACTGTCACTACCCGTCATCTGCCACAACGAGTTTATCGATGAAGCAGAAAAAGCCAGGCGCTGGGTGGAAATCAAGGAACGCCAGCAGATTCTGATGAAGGAGGCCGTGGAAGCTCAGATGCCTGCTCACATCGACCAGATCATGAGGAGTGGCTTCACCATGAATGATATTAAAGGTTTGCACTACGAGGACAATCAGGAAGAGGTCCCAAGTTCGAAGGTCAAGCGGCAACCGTCGAGACCTCCACGTAAGGGTTCGATGACCAGAGGCGTGTCTATACAAAATTACAATAATTTACAACCAGGTCAAGTAAACGGTATGCATCGGTCGACCAGTGAGGCTGAGATGGCTAAATACGACTTGGGAGCACAGAACTTGAGTCCTGATGATAAGAAGGATAAACGAGGACGGTCACCTTTCAAGTTCTTCAGAAGCAAACGAGGTGAAAGCAGCGACCGTGCCAAGAGTCGTAAGTCCAAGACACCAGATCCATTCAGTGACACCGAAGTATCCCCTGAACGTGGACCTCGAAGACCAAACCCTCAGATCAAAGTGTCACAGGCGAATCAAAGATATAACGGCATGAACGCGCGCCCTAGTCGGACCAACTTGTACGGAAGCCAAGTTGGTCTCAACATGGCGACGCCTACGCAAGACAGGAAACAAATGACTACGAGCACTCTTGCGCAGTCTACGACGGAAACTGTTGGCAATGAGATCTTCGACGCTGAGTGCTTGAAGCTCATCAATGAATACTTTTATGGAGTTAGGATATTCCCCGGTCAAGATCCGACACATGTGTACATAGGTTGGGTGACGACCCAGTACCATCTTCACTCCAAGGACTTCAACCAGAGCAAGGTGACCAAGTCATCGGTTATCATCACCGATGATTACGACAGGGTTATCGAAAATGTAAACCGCCAATCCTGCTACATGGTGCGCGCTGACGAGCTGTACAACGAGGTGATGGCTGAAGCCACAGCTAAGGGAGCGTCCCAAGGCATGTTCATTGGGTGCTCCGTGGATACCTCCACTGGCACTGTCTCCTTTACTTGTGAGGGCAAGGATACTAGCTTTAAATTCAAGATGGAACCAGAAACGAAGCTGTTCCCAGCAATCTTCGTGGAGGCGACGTCGAAGGAGATCCTCCAGATAGAACTGGGAAGGTCTACCACCAGCCTGCCTCTGTCGGCCGCAGTGCTCCCGACCAGTGACAAGCACGTCATCCCCCAGTTCCCCCCCAGGTTGAAAGTGCAGTGCTTGAAGCCACATCAGTGGGCTAGAGTACCGAACCAATCCTTACAAGTACACGCTCTGAAGTTGTCAGACATCCGCGGTTGGTCCATGCTGTGTGAGGATGCAGTGTCTATGCTAGCGTTGCATATACCAGAGGAAGACCGTTGCATAGACATTCTAGAACTAATAGAAATGGACAAACTTCTAAGTTTCCACTCACACACACTCACGCTGTATGCCGCATTGTGTTACCAGAGTAACTATAGGGCGGCGCACGCGCTGTGCACGCACGTGGACCAGAAGCAGTTGCTGTACGCGATCCAGTCGCAGTACATGTCGGGCCCGCTGCGACAAGGCTTCTACGATCTACTCATCGCGCTGCATCTCGAGTCACATGCTACCACCATGTATGTATCACTGCAATTTCTATTTTCCCGTTTCAGTGAAAGCATAACAGACATTAGCAACCTATACTCGCCGTACTTCCCGCTGGAGGTGGTCCGTGAGTTCGTGATGCAAGCTCTGGCGGAGGCCGTGGAGACCAACCAGGTCCATAACAGGGATCCTGTAGGAGGCAGCAATGAGAACCTGTTCCTGCCATTAATAAAGCTAGTAGACCGACTACTCTTAGTCGGAATGATGCGCGATGAAGATGTTGAGAAGCTGCTCATCATGACTAACCCAGAAACGTGGGACCCTTCGTTTGACAAAGAAGGTAAAGACGAGCACCGCAAAGGTCTACTTCACATGAAAATGGCCGAAGGCGCAAAGTTACAGATGTGTTACCTACTACAACATCTGAACGACATACAGCTGAGACACAGGGTCGAAGCTATCATTGCGTTTGCGCATGACTTCGTTGGAGATCTACAGACTGATCAGTTGAGACGTTACACAGAGATCAAGCAGTCAGACCTGCCCAGCGCGGTAGCAGCTAAGAAGACGAGAGAGTTCCGTTGCCCACCCAGAGAACAAATGAACGCAATCTTGAGTTTCAAGCACTTGGAAGAAGAAGACAAAGAGAACTGTCCATGTGGAGAGGAACTTATCGCCAGGATGAACGAGTTCCACGAGAGTCTGATGGCACATGTGTCGCTTAATGCTTTACAGGAACCGGATGGCACGGAGAACCAAGAACCTGAAGCGAAACCAGGTGCCTTTGGCAAGTTGTACAACATCATCAACACTGTGAAGGAACTTGAAGAAGAACCCAAGGCTATCGATGAGCCACCAAAGAAGACCCCAGAAGAGAAGTTCCGTAAGGTCTTAATCCAGACCATCGTCAACTGGGCTGAGGAGTCGCAGATTGAAACTCCCAAACTGGTCAGGGAGATGTTCAGTCTCCTAGTCCGTCAATACGACGCAGTGGGCGAGTTGATCCGTGCCCTGGAGAAGACGTACGTCATCAATGCGAAGACCAAGTTGGACGTGGCAGAGATGTGGGTCGGACTCAGTCAGATCCGAGCTCTTCTACCGGTACAGATGAGTCAGGAGGAAGAGGAACTTATGCGGAAGAGGCTTTGGAAACTCGTCAACAACCACACGTTTTTCCAACATCCAGACTTGATAAGAGTGCTCCGCGTGCACGAGAACGTGATGGCGGTGATGATGAACACGCTGGGCCGCCGCGCGCAGGCGCAGTCCGACGCGCAGCCCGCCTCGCCGCCCGTCGCAGAGGACAACAAGGAGAAGGACACATCCCACGAGATGGTAGTAGCGTGTTGTCGTTTCCTCTGCTACTTCTGTCGTACGGGACGACAGAACCAGAAGGCAATGTTCGATCACTTCGACTTCCTGCTGGAGAACTCCAACATCTTGCTGTCCAGACCCTCGTTGAGAGGTTCCACGCCTCTAGACGTGGCGTATTCCAGTTTGATGGAGAATACTGAGCTGGCTTTGGCTTTGAGGGAGCACTACCTAGAAAAGATAGCAGTGTACCTTTCTCGCTGTGGACTACAGAGCAACTCGGAGCTCGTCGAGAAAGGATACCCGGACCTTGGGTGGGATCCAGTCGAGGGCGAACGTTACCTGGACTTCCTACGATTCTGTGTCTGGGTTAACGGTGAAAGCGTAGAAGAGAACGCGAACTTAGTGATCCGTCTTCTGATCCGTCGTCCAGAGTGCTTGGGTCCCGCCCTCAGAGGAGAGGGTGAGGGTCTACTGAAGGCCATCGTAGACGCTAATAAAATGAGTGAGAGGATCGCAGACAGGAGGAAACTGAGGGAGATGGAGCAAGAAGGGGATATTAATTTCAGTCACCCGCTCCCCGAGTCCGACGAGGACGAGGACTACATCGACACTGGCGCCGCCATACTTAACTTCTACTGTACTCTAGTGGACTTGCTGGGACGGTGCGCGCCTGATGCAGGGGTTATTGCACTGGGCAAAAACGAGTCCCTCCGAGCCCGCGCCATCCTCCGTTCCTTGGTGCCCCTGGAGGACCTGCAGGGAGTGCTGAGTCTGAGGTTCACGCTCAACAACCCCGCTGCAGGAGAGGAGAGACCTAAGTCTGACATGCCGTCAGGGCTGATCCCCGGCCACAAGCAGAGCGTGGGTCTGTTCCTGGAGCGAGTGTACGGCATAGAGACCCAGGAACTGTTCTACAAGTTGTTGGAGGAAGCGTTCCTACCTGACCTCAGGGCGGCTACCATGCTGGATAGGAACGACGGTTGCGAGTCCGACATGGCTCTCTCGATGAACCGCTACATCGGGAACTCCATCCTGCCTCTACTGATCAAACACGCCTACTTCTACAACGAGGCCGAGAACTACGCCAGTCTCCTGGACGCCACGCTACATACTGTTTATAGGTTATCAAAGAACCGCATGTTAACCAAGGGTCAGCGTGAGGCGGTGTCAGACTTCCTAGTCGCCCTAACATCGGCCATGCAGCCTTCTATGTTGTTGAAACTATTGAGGAAACTGACAGTGGATGTGTCCAAGCTGTCGGAGTACACGACTGTCGCTCTTAGGCTTCTAACCCTTCACTACGAGCGTTGTGCCAAGTACTACGGTAGTACAGGAGCCGGCCAAGGCGTGTACGGAGCATCATCAGACGAGGAGAAGAGGCTCACCATGATGCTATTCTCAAACATCTTCGATTCCCTCAGCAAGATGGACTATGAGCCTGAACTCTTTGGGAAGGCGTTGCCATGTTTGATTGCTATTGGATGCGCTTTACCACCCGATTATTCGCTTTCCAAGAACTATGACGATGAATTCTACGGCAAAGAACAAGTAGCTGGTGACCTAGACAATCCTCAATACGACCCTCAGCCGATCAACACATCATCAGTAGCCCTCAACAATGACTTAAACACAATAGTCCAGAAGTTCTCAGAACATTACCACGATGCCTGGGCTTCTAGGAAGATCGAGAACGGATGGGTCTACGGAGAAGGATGGTCTGATAGCCAGAAGACGCATCCACGACTGAAACCGTATAATATGCTTAATGACTATGAAAAGGAGCGTTACAAGGAACCAGTTCGTGAATCTTTGAAAGCCCTTCTAGCCATCGGATGGTCAGTGGAACATTCAGAAGTGGACATACCAAGCACCAACCGCAGTTCCATGAGAAGGCAATCCAAATCGGGAGGCCGCCCTCCCGATATTGTGACGGACTCAGCAACTCCCTTCAACTACAACCCGCACCCAGTAGACATGACCAACTTGACGCTATCAAGAGAGATGCAGAATATGGCAGAAAGACTGGCTGATAATGCCCACGATATCTGGGCTAAGAAGAAGAAAGAAGAGCTTGTTACTAATGGAGGAGGTATCCACCCACAACTCGTACCCTATGATCTCTTAACAGACAAAGAGAAGAAGAAGGACAGAGAGAGATCGCAAGAGTTCCTCAAATATTTACAATACCAGGGTTACAAACTACACAGGCCAAGCAAGGCACCACAGAGTGATACGGAACAGACCACTACGGGCGTAGCAATAGAGCTCAGATTCGCATACTCGTTACTGGAGAAGTTGATACAGTATATAGATAGAGCGACCATCAATATGAAGCTCTTGAAACCTTCTACTACTTTCAGTAGGAGAACTAGTTTTAAAACTAGTACGAGGGATATTAAGTTCTTTTCTAAGGTGGTGCTGCCTCTGATGGAGAAGTACTTCTCAACGCATAGAAACTACTTCATAGCAGTAGCTACTGCTACCAACAATGTGGGTGCTGCCAGTTTAAAGGAGAAAGAGATGGTTGCAGCCCTATTCTGTAAACTGGCCAGTTTACTTAGATCTAGACTCGCAGCTTTCGGTCCTGATGTCCGCATCACCGTCCGTTGTCTCCAAGTGCTGGTCAAAGGCATCGACGCCAAGTCTCTGGTGAAGAACTGCCCCGAGTTCATCAGGACATCCATGTTGACCTTCTTCAATAATGTCGCTGATGATGTAGGGCATACCATCATGAATCTTCAGGATGGTAAATACGCACATCTCCGAGGTACACACTTGAAGACTTCCACTTCACTTGGCTACATCAACGGAGTTCTACTACCAATACTGACGGCCAAGTTTGATCACTTGGCCAACTGCGAGTACGGAGCTGACCTACTATTGGATGAGATCCAAGTGGCTTCATACAAAATGCTGGGTTCACTGTACGCTTTAGGCACTGACGCGTCCCTCACACACGACAGAAAATACCTCAAGACTGAAATCGAGAGACATAAACCTGCTTTGGGTTCGTGCCTCGGTGCGTTCAGCTCGACATTCCCAGTGGCGTTCTTGGAGCCCCACTTGAACAAACACAATCAGTTCTCCTTGCTGAACAGGATCGCTGACCACTCTTTGGAGGCTCAAGATATAATGCAGAAAATGGAACAATGCATGCCGACACTAGAAACGATCTTGGGTGAAGTGGACCAGTTCGTGGAGTCTGATAAGACTTACAACGAAGCTCCTCATATTATTGATGTGGTGTTGCCTTTACTCTGCTCTTACTTGCCGTTCTGGTGGGCACAGGGACCTGATAACGTTACTCCTACTGGAGGCAACCATGTAACAATGGTGACAGCAGAACACATGAACCAACTTCTAAAGAACGTTCTGAAGCTTATCAAGAAGAACATAGGCAATGAGAACGCTCCATGGATGACGCGCATAGCTACCTACACCCAACAGATCATCATCAACAGTTCAGAGGAACTCCTCAGGGACTCCTTCCTTCCTCTCGCTGAGAGGGTCCGAAAGAGGACCGATAATATGTTCCATAAGGAGGAGAGCTTGAGAGGATTTATTAAGTCATCAACTGACGATACATCTCAAGTAGAATCTCAAATCCAAGAAGACTGGCAGCTGTTGGTTCGTGACATTTATTCCTTCTACCCACTGCTCATCAAATATGTAGACTTACAAAGAAACCACTGGCTCAGGAACAACGTGCCAGAGGCTGAAGAGCTTTACAACCACGTAGCTGAAATTTTCAACATCTGGTCCAAGAGTCAGTACTTCTTGAAAGAAGAGCAGAACTTCATATCTGCTAATGAGATTGATAACATGGTACTGATCATGCCAACAGCAACTAGAAGGGTAACAGCAGTTGTAGACGGTACTCCACAAGCCGGTGGCAAGAAAAAGAAGAAGCACCGCGATAAGAAGCGAGATAAAGACAAAGAAGTCCAAGCCTCTCTAATGGTGGCCTGTCTCAAAAGACTTCTGCCAGTAGGCCTGAACCTCTTCGCAGGTAGAGAGCAGGAACTGGTACAGCATTGCAAAGACAGGTTCCTGAAGAAAATGTCTGAACAAGATGTAGCTGAATTTGCGAAAACACAATTGACATTGCCTGATAAAATTGACCCAGCTGATGAAATGTCTTGGCAACATTATTTGTATAGCAAACTGGGTTCTAAGAGTAAAACGAATATAACTGTTGAGACTGCTGAGAATAAAGCTAAAATAATTGATGACACCGTGGAAAGGATTGTTGCTATGAGCAAAGTGCTGTTTGGATTACATATGATTGACCATCCCCAACAAATGAGTAAGAATGTCTACCGTTCAGTGGTGTCTATCCAACGTAAGCGAGCTGTCATCGCTTGCTTCAGACAAACGTCGCTACATTCTCTGCCGAGACATCGAGCGTGTAATATATTCGCTCGAACATATTACGAGCTGTGGCTGGAAGAAGAGAACGTTGGCCAAGAAGTCATGATTGAAGATCTTACGCAATCGTTCGAAGACGCGGAGCTGAAGAAGAGCGACGGTGAGGAGGAGGGAGAGAAGCCTGATCCTCTGACACAGCTAGTCACCACCTTCTGCAGGGGAGCCATGACTGAGAGATCTGGAGCGTTGCAGGAGGATCCCCTCTACATGTCTTACGCACACATCATAGCGAAGTCCTGTGGAGAAGAAGAGGAAGAAGGGGGCGGGGAAGAAGAAGAGGGCGGGGGTGAAGCCGAGGCAGAAGATGAGGGCAGAGCCAGTATACATGAACAAGAAATGGAAAAACAGAAGCTCCTCTTCCATCAAGCTCGTCTAGCGAACAGGGGTGTGGCAGAGATGGTGCTCCTCCATATATCTGCGTCCAAGGGGCTACCTAGCGAGATGGTGATGAAGACTCTACAGCTGGGAATCTCTATACTTAGGGGAGGCAATATTGATATACAGATGGGTATGCTGAACCATCTGAAAGACAAAAAAGACGTCGGTTTCTTCACTTCGATAGCTGGTCTCATGAACTCCTGTTCCGTGCTCGATCTGGACGCCTTCGAGAGGAATACTAAGGCTGAAGGTCTGGGAGTGGGCCTGGAAGGCGCGGCAGGTGAGAAGAACATGCACGACGCGGAGTTCACGTGCGCACTGTTCCGGTTCATACAACTTACTTGTGAAGGACACAACTTGGACTGGCAGAACTATCTCAGAACGCAGGCTGGTAACACGACGACAGTGAACGTCGTCATCTGTACCGTCGACTATCTGCTGCGGCTACAGGAGTCCATCATGGACTTCTACTGGCATTACTCCAGCAAGGAGTTAATCGATCCAGCTGGCAAGGCGAACTTCTTCAAGGCGATAGGTGTCGCGTCCCAAGTGTTCAACACTCTCACTGAGGTCATCCAGGGACCTTGTACACAGAACCAACAGGCTCTGGCACACTCTAGGTTATGGGACGCAGTAGGTGGTTTCCTGTTCCTATTCTCCCACATGCAGGACAAGTTGTCGAAACACTCGTCCCAGGTGGACCTGCTGAAGGAACTCCTCAACCTGCAGAAGGATATGATCACTATGATGCTGTCTATGCTGGAAGGAAACGTTGTTAACGGCACTATCGGTAAGCAGATGGTGGATACGCTAGTAGAATCTGCATCCAACGTGGAGCTGATCCTGAAATACTTCGACATGTTCCTGAAACTGAAGGACCTGACATCTAGCGCCAGCTTCCAGGAGATTGATGCTAATAATGATGGATGGGTGCTGCCTAAGGACTTTAAGGAGAAGATGGAACAGCAGAAGAGTTATACACCTGAAGAAATCGAGTTCCTCCTAGCATGCTGTGAGACGAACCACGATGGGAAACTGGACTACATCGGCTTCTGTGACCGGTTCCATGAACCTGCCAAGGAGATCGGGTTCAACTTGGCTGTACTGCTCACCAATTTGTCTGAACATATGCCTAATGAACCCAGGTTGGCCCGTTTCCTAGAGACAGCTGGTTCTGTCCTGAACTACTTCGAGCCATTCCTCGGTCGCATCGAGATCATGGGCGGCTCCAAGCGGATAGAGCGAGTCTACTTCGAGATCAAGGAGTCTAACATCGAACAGTGGGAGAAACCACAGATTAAGGAATCCAAACGCGCATTCTTCTACAGCATAGTAACAGAAGGAGGAGACAAGGAGAAGTTGGAGGCCTTCGTCAACTTCTGTGAGGACGCCATCTTTGAAATGACTCACGCGTCCGGACTCATGGCCGCGTCCGAGGAGTCTGTCGGGGGAACCAAGAATAGGGAGGCTAGCTACATGTATATGGGAGATGATGATGATGAGAGAGCCGGCAAAGATCCATTCCGTCGTGGTCTCCAATCAGTAAAAGACGGCATCTACACAGCATTCTCATCCTTATCCCCATCGAACATAAAGGCGAAAATCGCAGATCTCCAACAAATGCCGCCAGCAGAACTTGCAGTGGGCTTCTTCAAAATGTTCTTCCTATTATTCTATTACTTGGGCTACGGAATGCTCGTCGTGATCAGGTACATATTCGGCGTCCTTCTCGGTCTGATGAGAGGTCCACAGACGGACGAACCGCCGCCCGAACCGACAGAGGAGGAGAAAATCGGTCAGTTAAGACACAGGTTGCTAGCCACACAAAGTTCTCGACATCTACCAGCGTTACCACCAGCTGACGACACTGGGCAGATGCAGGTCTCCGCATTCGGACTGGATATCACTAAAGAAGACAATGGACAGATACAGGTAAAACCGCACGAGTCACCGAGCACATCAACGCCATCTTCCGGCGAGGAGGCTGAAGTATCTCCTGATGAGAACGCCGACCATCCTGAGGAACAGAGACCACCGTCACTCATCGATCTACTTGGAGGAGAACAAGCGAAGAAGCAGGCTCAAGAACGTATGGAAGCGCAGGCTGCCCAACAAGCTGCTATGTCCGCTATTGAAGCTGAGAGCAAAAAGGCAGTCCAAGGTCCGGCTCCATCAGCGTTATCACAAGTGGACCTGTCCCAGTACACCAGGCGTGCGGTCTCCTTCTTGGCTCGAAACTTCTATAATTTGAAATACGTCGCCCTCGTCTTGGCTTTCTGTATCAACTTTGTACTGCTGTTCTATAAGGTGTCTACGTTGGACGCGGAAGGTGGCGAGGGTTCTGGTCTGGGCGATATCATCGCTGGATCTGGATCGGGCTCAGGATCTGGCAGTGGAGATGGTGGTAGCGGTGAATCAGGTGAAGACGACGATGCTTTAGAAGTGGTACATATAGACGAGGACTTCTTCTACATGGAGCATGTCATCAAAGTGGCCGCCGTACTGCACTCCATCGTGTCTCTGGCTATACTTATTGGATATTACCATCTTAAGGTGCCACTAGCTATCTTCAAGCGTGAGAAAGAGATCGCCCGTAAGCTGGAGTTTGATGGTCTTTACATCGCTGAGCAGCCAGAAGACGACGACCTCAAGAGCCACTGGGATAAACTTGTTATATCTGCCAAATCGTTCCCGGTGAACTATTGGGATAAGTTCGTGAAGAAGAAGGTTCGTGCCAAGTACTCCGAGACTTATGACTTCGACTCCATATCCAACATGCTGGGCATGGAGAAGACTTCCTTCTCTGCTCAAGAAGAAGAAGGCAGCAAGGGACTGATTCATTACATAATCAATATAGACTGGCGTTACCAAGTATGGAAGGCGGGAGTGACGATCACAGACAACTCCTTCCTCTATTCTCTATGGTACTTCTCATTCTCCGTGATGGGTAACTTCAACAACTTCTTCTTCGCTGCCCATCTACTAGATGTCGCTGTTGGATTCAAAACTTTGAGAACCATTCTACAGTCCGTCACGCATAATGGAAAACAGCTGGTCCTGACGGTGATGCTGCTTACCATCATAGTGTATATCTATACCGTGATAGCGTTCAACTTCTTCCGCAAGTTCTACGTGCAGGAGGAAGACGACGAGGTCAATAGGAACTGCCATGATATGCTTACGTGCTTCGTATTCAACCTGTATAAAGGAGTACGAGCCGGCGGCGGTATTGGCGACGAGCTGGAGCCGCCCGATGGAGACGACTCCGAGGTCTACCGCATCATATTCGACATCACATTCTTCTTCTTCATCATCGTCATCCTGCTGGCTATTCTGCAGGGTTTGATCATCGACGCGTTCGGTGAACTGCGTGATCAGTTGGAGTCGGTGAAGGAAGACATGGAGTCCAACTGCTTCATCTGTGGCATTAATAAAGACTATTTTGATAAAGTGCCGCACGGGTTCGACACTCACGTCCAGAGAGAGCATAATCTAGCGAACTACATGTTCTTCCTTATGCATCTCATTAATAAGCCCGATACGGAATATACAGGTCAAGAAACGTATGTATGGAATATGTACACGCAACGATGCTGGGACTTCTTCCCTGTCGGAGACTGCTTCAGGAAACAGTATGAAGATCTAATGGGAGAATAGACAACTACACCATAAACAATCTCGCTTAAACGTAGCAATTTCAAATTTATTGCTCAAAGTGAACACCAATGAATAGAAATAATCTATTGCAACTTTCTTACACGTCGGTACAATAATTTTGTAAATAAATCTGTATTTTTTTCTTCGTATTTATGTTTGAATAAACATGGCGTGTTAATGTTTATATAATCTATGTATGTACGATAAAGAATTACGGTTGTGGATTACAGAGGAAATTAAAACAAAAATCCAGTATTTAATTGAGAAAATTGTCCTTGAAAGAAATAGCTTCAACATAAATTGTATTATTCTTGCGATTTCTCGTCGAAAATCCTAAATAAAACTTTAGAAACAGACAAACGGTTTTATCTATATCTTTAAATAAGTCTAACATAAGGTGGCCTTTCCATGTAAACTTTTCTATTTTTAACTATTTCGATATAATAATATGTATAAGGTAAATAA
