# Supplementary material for: Identification of transcriptome and fluralaner responsive genes in the common cutworm Spodoptera litura Fabricius, based on RNA-seq
Source: BMC Genomics. 2020 Feb 3;21:120. doi: 10.1186/s12864-020-6533-0 (PMC6998375; doi:10.1186/s12864-020-6533-0)
Supplement: Supplementary file 12 — Additional file 12. Functional classification of DEGs according to COG database. Note: A represented the DEGs and all genes after the exposure of LC30 fluralaner, B represented the DEGs and all genes after the exposure of LC50 fluralaner, respectively. [file 12864_2020_6533_MOESM12_ESM.docx]

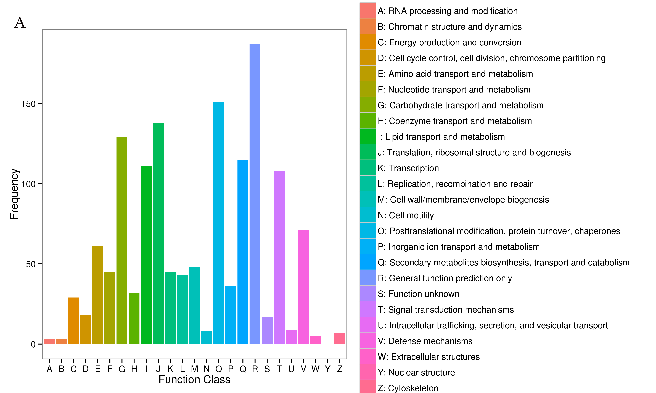

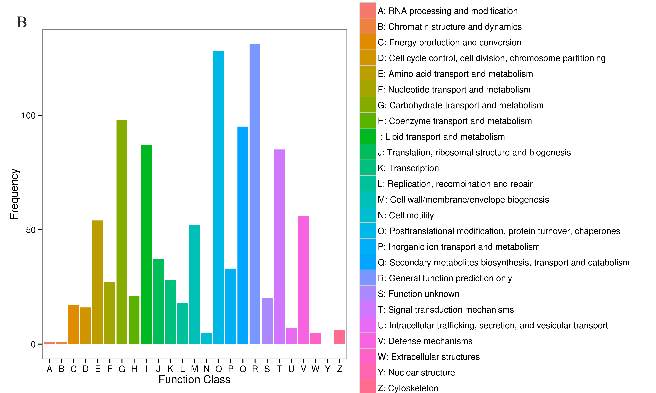


**Additional file 12** Functional classification of DEGs according to COG database

Note: A represented the DEGs and all genes after the exposure of LC_30_ fluralaner, B represented the DEGs and all genes after the exposure of LC_50_ fluralaner, respectively.
