# Supplementary material for: Identification of transcriptome and fluralaner responsive genes in the common cutworm Spodoptera litura Fabricius, based on RNA-seq
Source: BMC Genomics. 2020 Feb 3;21:120. doi: 10.1186/s12864-020-6533-0 (PMC6998375; doi:10.1186/s12864-020-6533-0)
Supplement: Supplementary file 14 — Additional file 14. Changes of DEGs related to detoxification and development of S. litura after exposure of fluralaner. [file 12864_2020_6533_MOESM14_ESM.docx]

**Additional file 14**: Changes of DEGs of *S. litura* after exposure of fluralaner

| Gene ID | Annotation | Regulation | Log_2_ FC of treatment | |
| --- | --- | --- | --- | --- |
|  |  |  | LC_30_ | LC0_50_ |
| gene9333 | probable cytochrome P450 6A13 | Up | 3.03 | 3.94 |
| gene13430 | cytochrome P450 4G15-like | Up | 3.30 | 3.04 |
| gene4502 | cytochrome P450 6B7-like | Up | 2.05 | 2.60 |
| gene17041 | cytochrome P450 9E2-like, partial | Up | 2.32 | 2.24 |
| gene1218 | cytochrome P450 9E2-like | Up | 2.23 | 2.14 |
| gene11881 | cytochrome P450 4D2-like | Up | 1.75 | 2.04 |
| gene11879 | cytochrome P450 CYP12A2-like | Up | 2.20 | 1.95 |
| gene2558 | probable cytochrome P450 49A1 | Up | 2.23 | 1.95 |
| gene15894 | cytochrome P450 9E2-like | Up | 1.30 | 1.94 |
| gene3595 | cytochrome P450 4C1-like | Up | 1.95 | 1.94 |
| gene9175 | cytochrome P450 4C1-like | Up | 1.64 | 1.64 |
| gene9509 | probable cytochrome P450 304A1 | Up | 1.68 | 1.60 |
| gene10859 | cytochrome P450 4V2-like | Up | 1.52 | 1.54 |
| gene9174 | cytochrome P450 4C1-like | Up | 1.37 | 1.30 |
| gene3598 | cytochrome P450 4V2-like | Up | 1.66 | 1.17 |
| gene12726 | cytochrome P450 9E2-like | Up | 1.42 | 1.12 |
| gene7753 | glutathione *S*-transferase 2-like | Up | 2.35 | 2.21 |
| gene8407 | venom carboxylesterase-6-like | Up | 1.40 | - |
| gene5053 | carboxylesterase 1C-like | Up | 2.02 | - |
| gene15885 | liver carboxylesterase B-1-like | Up | 1.36 | - |
| gene6005 | probable chitinase 10 | Down | -4.59 | -4.64 |
| gene1042 | juvenile hormone esterase-like | Down | -1.05 | -1.13 |
| gene2891 | juvenile hormone acid *O*-methyltransferase-like juvenile | Up | 2.02 | 1.77 |
| gene2887 | hormone acid *O*-methyltransferase-like isoform X2 | Up | 1.30 | 1.24 |
| gene8419 | torso-like protein | Down | -1.07 | -1.63 |
