# Supplementary material for: Identification of transcriptome and fluralaner responsive genes in the common cutworm Spodoptera litura Fabricius, based on RNA-seq
Source: BMC Genomics. 2020 Feb 3;21:120. doi: 10.1186/s12864-020-6533-0 (PMC6998375; doi:10.1186/s12864-020-6533-0)
Supplement: Supplementary file 15 — Additional file 15. Primers for RT-qPCR validation. [file 12864_2020_6533_MOESM15_ESM.docx]

**Additional file 15: Primers for RT-qPCR validation.**

| Gene ID | Primer name | Primer sequence (5’-3’) | product size (bp) |
| --- | --- | --- | --- |
| gene11879 | qgene11879-F | CCGCTCTTCCAGTTGATA | 84 |
|  | qgene11879-R | CAAGGCTTCTGTCTTCAC |  |
| gene1218 | qgene1218-F | CTCGTAGCACAAGCAGTA | 146 |
|  | qgene1218-R | CCGTTCTTAGCATCATTCTC |  |
| gene9333 | qgene9333-F | AGATAAGGTGCTGAGTGAG | 175 |
|  | qgene9333-R | GTCTGGGTCTGGATACAAT |  |
| gene13430 | qgene13430-F | AACAAGGAGTGTGAGGAG | 83 |
|  | qgene13430-R | CTTCTGAGGACATCTTCGTA |  |
| gene4060 | qgene4060-F | GCGAATGGTATCCTGAAC | 148 |
|  | qgene4060-R | GCTCATCCTCTTCCTTGTA |  |
| gene7753 | qgene7753-F | CTAAGAACAACGGTCACG | 95 |
|  | qgene7753-R | CATACGCATCATAGCACTC |  |
| gene5810 | qgene5810-F | GATTCTGTTGCGTCATAGTC | 104 |
|  | qgene5810-R | CAGGTTCTCCAGTCTCAG |  |
| gene11398 | qgene11398-F | CGTGTTCCTCGTCTACTAT | 180 |
|  | qgene11398-R | CTTCCACCACTTCTTGATG |  |
| gene9409 | qgene9409-F | TCAGTGGTTGGTGGTATG | 140 |
|  | qgene9409-R | CGAGTTCTCTTGGTAGTCT |  |
| gene6042 | qgene6042-F | GAATAACGCTGCCAGAAC | 136 |
|  | qgene6042-R | CCATTGAAGGAGAGTGCTA |  |
| gene13600 | qgene13600-F | CCCGATGGCAAGTATGTA | 185 |
|  | qgene13600-R | CCGTATTTCAGGAGACCA |  |
| gene13631 | qgene13631-F | CAGGCTAACCAGAATAACC | 148 |
|  | qgene13631-R | ACTGTCCATGAGCTTGAG |  |
| gene1073 | qgene1073-F | GGACCTTGACACACTGAA | 97 |
|  | qgene1073-R | TGGCACTATGAGGACTTG |  |
| gene8052 | qgene8052-F | GCCTTCAATGATGCTTACC | 140 |
|  | qgene8052-R | GACTCCCAATCTTCACTTTC |  |
| gene6203 | qgene6203-F | CCGAGGTTCATCAAGTCT | 160 |
|  | qgene6203-R | TGAAGTCGCCTGTGTATC |  |
| gene6209 | qgene6209-F | TGGAGGATGTGGAGAATG | 75 |
|  | qgene6209-R | CTGATCGTTGGTATGTGAC |  |
| gene334 | qgene334-F | TTAGGTGAGGTGCAGATG | 89 |
|  | qgene334-R | GCGGAGTATGTGAGGTAT |  |
| gene6005 | qgene6005-F | AACGACCTGCTACTACTAC | 134 |
|  | qgene6005-R | CATACCGCCTTCACATTG |  |
| gene7031 | qgene7031-F | TCACCAAGCATCTCCTAC | 158 |
|  | qgene7031-R | CCTACACCACGAGCATAA |  |
| gene4202 | qgene4202-F | ATGTCTGCCTACCTTCTC | 185 |
|  | qgene4202-R | CTCCTGTTCCTTGCCTAT |  |
| gene10548 | qgene10548-F | ACCTGCTTCTGGAGTATG | 91 |
|  | qgene10548-R | CTGTGGCTCGTAGTAGTG |  |
| gene5961 | qgene5961-F | CTTGAGAGAAGTCGTAGCA | 145 |
|  | qgene5961-R | CCATTCCGCAGTATTGAC |  |
| gene11038 | qgene11038-F | CCTGGTATGGGTTAAGAAGA | 99 |
|  | qgene11038-R | TGAAGTGTTACGGCTGAG |  |
| gene1041 | qgene1041-F | GGTCGCAGTATATGTTCAC | 178 |
|  | qgene1041-R | GTCCTTCATTCCAGCATTG |  |
| gene13788 | qgene13788-F | ACTCCACTCTTCTCCTCT | 127 |
|  | qgene13788-R | CTGTCCTTCTCACTTCAATG |  |
| gene10843 | qgene10843-F | GGTATATGTCGCAGTTCCA | 81 |
|  | qgene10843-R | CTTCCCGCTCTCAATGTA |  |
| EF-1α | qSlEF-1a-F | CCCATACAGCGAATCCCGT | 124 |
|  | qSlEF-1a-R | AATGTTGTCTCCGTGCCAGC |  |
